# Catalyst‐Free Direct Hydrocarbonation of Terminal Alkynes Toward *E*‐Alkene Substituted Stabilized Sulfoxonium Ylides

**DOI:** 10.1002/advs.202417362

**Published:** 2025-04-02

**Authors:** Haiting Wu, Yougen Xu, An Lin, Jingyuan Liu, Huanjun Chen, Shimin Xie, Lebin Su

**Affiliations:** ^1^ School of Pharmaceutical Sciences Guangzhou Laboratory Guangzhou Medical University Guangzhou 511436 China; ^2^ Bioland Laboratory Guangzhou 510005 China; ^3^ State Key Laboratory of Chemo/Biosensing and Chemometrics College of Chemistry and Chemical Engineering Hunan University Changsha 410082 China

**Keywords:** hydrocarbonation, terminal alkynes, amide‐sulfoxonium ylide, high‐throughput experimentation, 1,5‐dicarbonyl compounds

## Abstract

Amide and alkene moieties are frequently found in natural products and are privileged structures in pharmaceuticals and agrochemicals. Moreover, vinyl sulfoxonium ylide can be converted into a broad range of high‐value compounds, thus they have been widely employed in organic synthesis. However, the synthesis of alkene‐substituted amide‐sulfoxonium ylides via intermolecular hydrocarbonation of alkynes remains underexplored. This study describes the development of a high‐throughput approach to provide diverse functionalized *E*‐alkene substituted (hetero)amide‐sulfoxonium ylides. The reaction occurs under mild metal‐free conditions, employing amide‐sulfoxonium ylides as highly effective nucleophiles, which participate in Michael addition reactions with various substituted alkynes, such as esters, thioesters, ketones, amides, and sulfones. This low‐cost, operationally simple approach has a broad substrate scope, high functional group compatibility, and excellent regio‐ and stereoselectivity, making it suitable for the transformation of structurally complex molecules. Furthermore, the obtained stabilized sulfoxonium ylide products are directly useful for the synthesis of diverse valuable 1,5‐dicarbonyl and thiabenzene 1‐oxide compounds.

## Graphical abstract

The authors introduce a high‐throughput synthesis using amide‐sulfoxonium ylides as new nucleophilic reagents, enabling a catalyst‐ and base‐free hydrocarbonation of terminal alkynes under mild conditions, resulting in various *E*‐alkene substituted (hetero)amide‐sulfoxonium ylides with excellent regio‐ and stereoselectivity. The late‐stage modifications and diverse transformations of the products demonstrate their utility and generality.

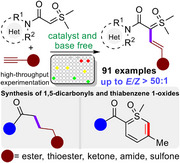

## 1 Introduction

Sulfur ylides hold great potential for synthetic applications in the fields of organic chemistry, pharmaceutical chemistry, and materials chemistry.^[^ ^1^, ^2^ ^]^ Over the past decade, sulfoxonium ylide compounds have received increasing interest due to their bench‐stable and operational safety, which are regarded as the important versatile reagents in organic synthesis.^[^ ^3^ ^]^ Similar to diazo metal carbenes, sulfoxonium ylides can produce metal carbenes to participate in many chemical transformations,^[^ ^3^, ^4^ ^]^ such as cyclopropanation, various X─H bond insertions, cycloaddition, C─H functionalization, cross‐coupling, and others in the presence of a suitable metal catalyst. Traditionally, sulfur ylides are prepared by the deprotonation of sulfonium salts with strong bases or the reaction of sulfides/sulfoxides with metal carbenes (i.e., using α‐diazo carbonyls as starting materials).^[^ ^5^ ^]^ The tedious operation (i.e., diazo compound synthesis) and the use of stoichiometric amounts of metal salts have motivated and inspired chemists to pursue more direct and efficient alternatives. In recent years, numerous methods have been developed for the synthesis of bis‐substituted sulfonium/sulfoxonium ylide compounds (e.g., dicarbonyl sulfonium/sulfoxonium ylides, tricarbonyl sulfoxonium ylides, α‐cyanato carbonyl sulfoxonium ylides, α‐aryl/alkyl carbonyl sulfoxonium ylides, and indolyl ketosulfoxonium ylides) through transition‐metal/light catalyzed cross‐coupling reactions using α‐keto sulfoxonium ylides as substrates.^[^ ^6^, ^7^ ^]^ Despite significant advancements, the synthesis of polyfunctional vinyl sulfur ylide compounds remains underexplored. Therefore, the development of new general, efficient, and green strategies to selectively access the structurally and functionally diverse sulfur ylide compounds from readily available starting materials would be highly desired.

**Figure 1:**
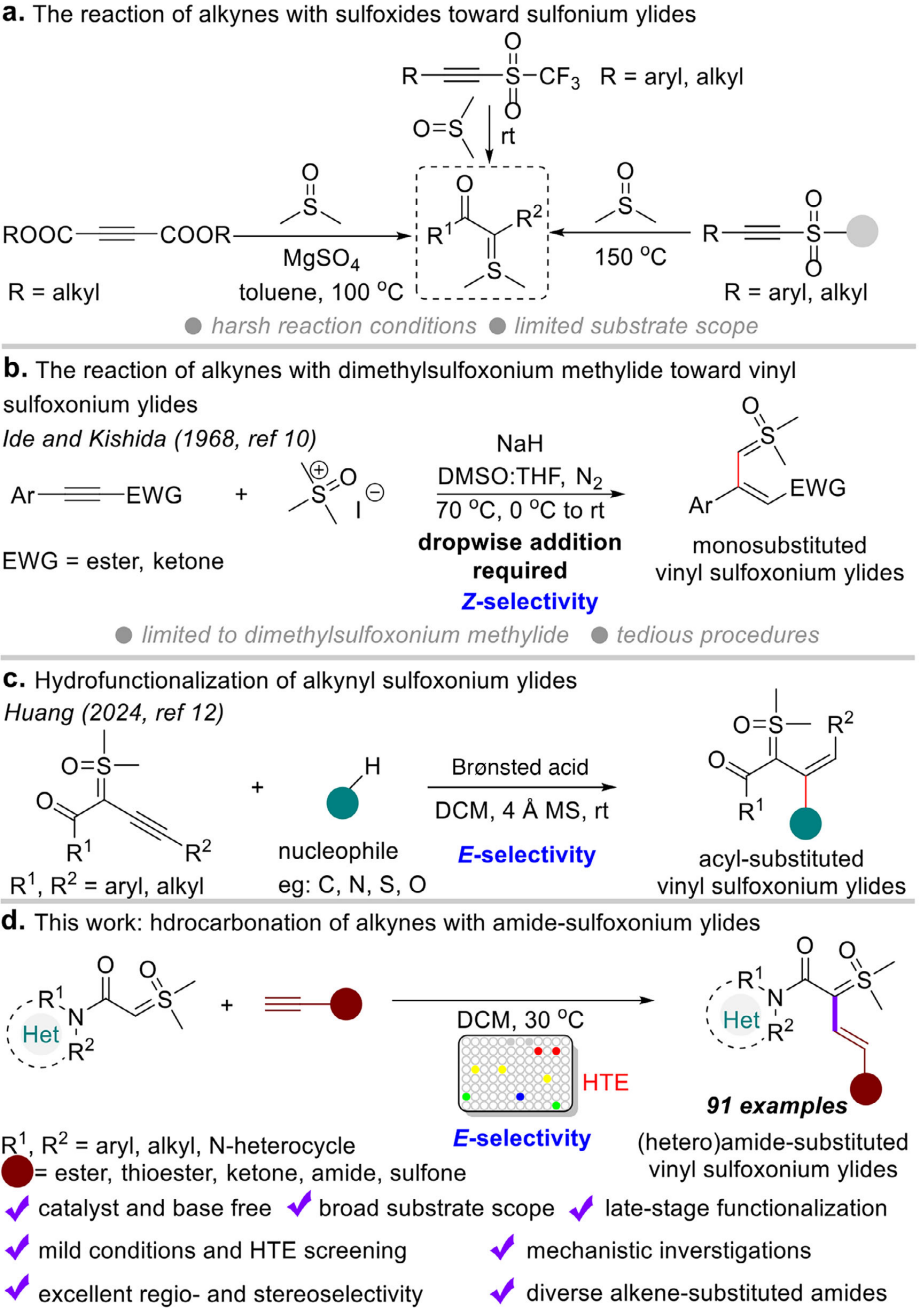
Approaches to functionalized sulfur ylides from alkynes. a) The reaction of alkynes with sulfoxides toward sulfonium ylides. b) The reaction of alkynes with dimethylsulfoxonium methylide toward vinyl sulfoxonium ylides. c) Hydrofunctionalization of alkynyl sulfoxonium ylides. d) This work: hydrocarbonation of alkynes with amide‐sulfoxonium ylides.

Alkynes, due to their abundance and economic accessibility,^[^ ^8^ ^]^ have emerged as promising starting materials for the synthesis of sulfur ylides. In 1989, Hanack and co‐workers first reported the reaction of acetylenic trifluoromethylsulfones with dimethyl sulfoxide (DMSO) for the synthesis of α‐acyl‐α‐sulfonyl sulfonium ylides at room temperature for 1 to 2 day(s).^[^ ^9a^ ^]^ After three decades, Xu and co‐workers developed an efficient protocol for synthesizing sulfonium α‐acyl‐α‐sulfonyl methylides using acetylenic sulfones with sulfoxides.^[^ ^9b^ ^]^ Subsequently, Mo and co‐workers achieved MnSO_4_‐promoted S─O bond cleavage, enabling the preparation of diacyl sulfonium ylides from acetylenedicarboxylates (**Figure** **1**a).^[^ ^9c^ ^]^ However, obvious shortcomings remain. High‐temperature and/or pre‐prepared substrates are required. Moreover, the above methods are primarily limited to the synthesis of dicarbonyl sulfonium ylides. Besides, an interesting work was reported by Ide and Kishida in 1968, in which the authors synthesized mono‐substituted *Z*‐alkene sulfoxonium ylides in a one‐pot protocol that involved in situ formation of active dimethylsulfoxonium methylide, which then underwent nucleophilic addition with ethyl 3‐phenyl‐2‐propynoate derivatives (Figure 1b);^[^ ^10^ ^]^ however, this method was restricted to dimethylsulfoxonium methylide, and a dropwise addition procedure was tedious. Importantly, the application of vinyl sulfoxonium ylides in constructing various heterocyclic and carbocyclic skeletons have recently received significant attention from the Hopmann group, Vaitla group, and Zhang group.^[^ ^11^ ^]^ In 2024, Huang and co‐workers reported an efficient strategy for the synthesis of acyl‐substituted *E*‐alkene sulfoxonium ylides via hydrofunctionalization reaction between alkynyl sulfoxonium ylides and various nucleophiles (e.g., C, N, S, O), with the aid of a Brønsted acid (Figure 1c).^[^ ^12^ ^]^ With the addition of a chiral phosphoric acid (CPA), the authors also demonstrated an asymmetric hydroamination of electron‐rich alkynes with pyrazole derivatives in chloroform at −60 °C, leading to axially chiral vinyl sulfoxonium ylide products in high yield and excellent enantioselectivity. This strategy is effective, but the preparation of alkynyl sulfoxonium ylide precursors requires multiple steps, which limits its application. Thus, the development of novel and practical synthesis of alkene‐substituted sulfoxonium ylides through hydrocarbonation reaction of alkynes is highly warranted.

Amide and alkene moieties are present in many natural products, pharmaceuticals, and fine chemicals.^[^ ^13^ ^]^ Considering the importance of alkene‐substituted amides and our ongoing interest in alkyne and sulfoxonium ylide chemistry,^[^ ^14^ ^]^ herein, we present an efficient and environmentally friendly route of catalyst‐ and base‐free hydrocarbonation of terminal alkynes with amide‐sulfoxonium ylides (Figure 1d). This method enables the preparation of various *E*‐alkene substituted (hetero)amide‐sulfoxonium ylide compounds in high yields with excellent regio‐ and stereoselectivities. A broad range of functional groups (e.g., ester, thioester, ketone, amide, sulfone, heterocycle, etc.) were directly incorporated onto bis‐substituted vinyl amide‐sulfoxonium ylide skeletons from readily available and economical starting materials under mild conditions. This straightforward method is amenable to a wide scope of substrates and can be used in the late‐stage modification of structurally bioactive molecules and natural products. It is worth highlighting that alkenyl (hetero)amide‐sulfoxonium ylides undergo subsequent reduction‐hydrogenation reactions through Pd/C catalysis providing a flexible platform for the synthesis of 1,5‐dicarbonyl compounds, which were difficult to obtain by previous methods.

## 2 Results and Discussion

**Figure 2:**
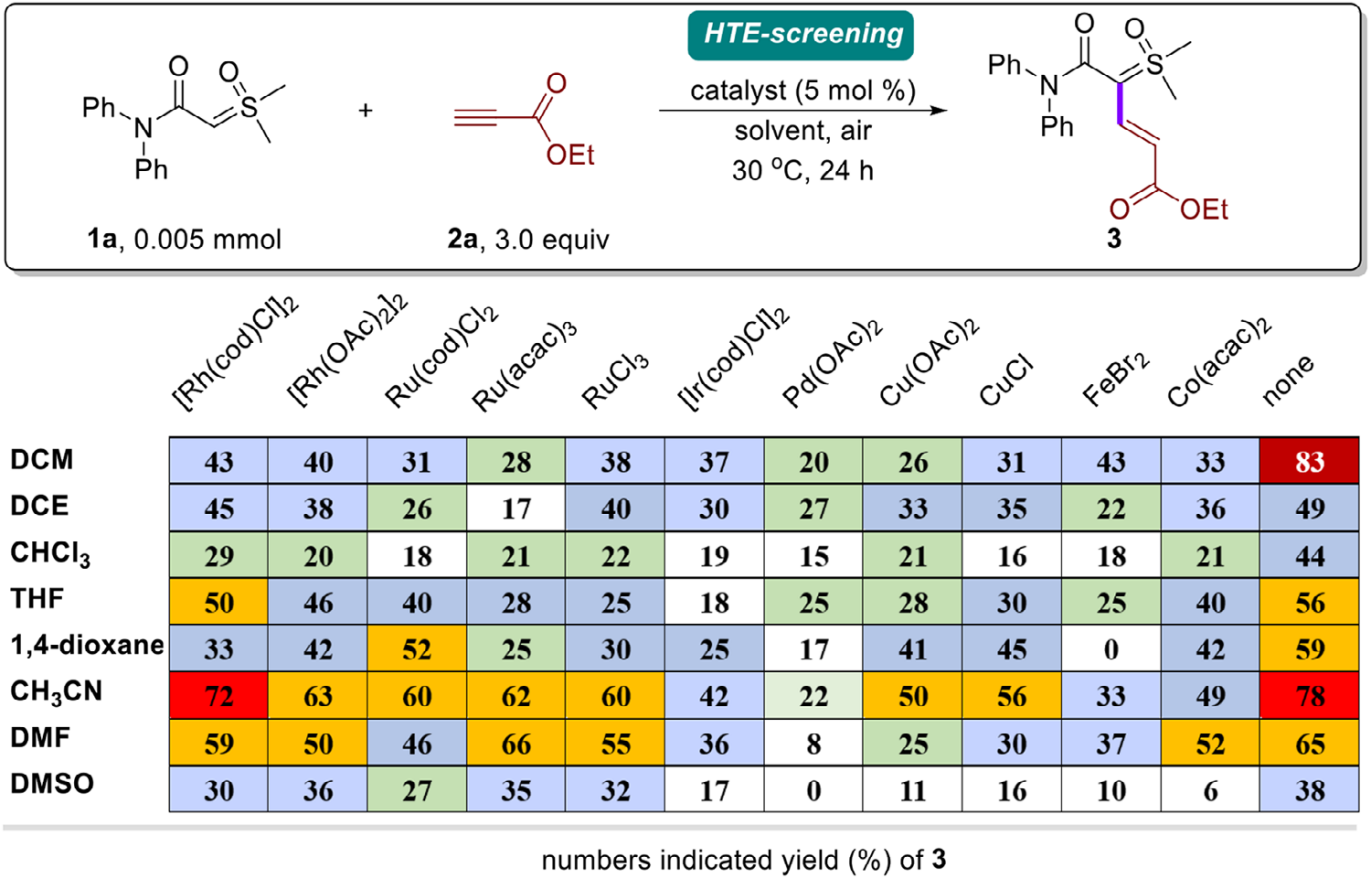
Reaction conditions screening by automated HTE platform.

We commenced our studies by exploring the selective hydrocarbonation of amide‐sulfoxonium ylide (**1a**) with ethyl propiolate (**2a**). Inspired by sulfoxonium ylide as a versatile reagents in organic synthesis for a number of chemical transformations, a range of metal catalysts and solvents were screened through an automated high‐throughput experimentation (HTE) platform (**Figure** **2**, see the Supporting Information for details of the HTE‐screening, Table S1, Supporting Information). After extensive screening of the reaction parameters, we found that a metal catalyst was not necessary for this reaction. To our delight, using DCM as solvent at 30 °C under air, we obtained the corresponding product **3** in 83% yield with high regio‐ and stereoselectivity. In addition, [Rh(cod)Cl]_2_, [Rh(OAc)_2_]_2_, Ru(cod)Cl_2_, Ru(acac)_3_, and RuCl_3_ also displayed good efficiencies in this reaction. For other noble and non‐noble metal catalysts, such as Ir, Pd, Cu, Fe, and Co demonstrated lower efficiency for the reaction. Although the reaction could proceed effectively under metal‐free conditions, side reactions occurred in the presence of metal catalysts, such as homo‐dimerization, intramolecular carbene C─H insertion, and other unknown reactions.^[^ ^14b,c^ ^]^ In regard to the solvent, this hydrocarbonation of electron‐deficient alkyne reaction also proceeded efficiently in CH_3_CN and DMF, but poorly in DCE, CHCl_3_, THF, 1,4‐dioxane, and DMSO. To our delight, scale‐up of the reaction from 5 µmol (microscale‐HTE experiment) to 0.1 mmol scale was demonstrated in a similar 81% yield. Notably, the reaction was inhibited when Na_2_CO_3_, *^t^* BuOK, or Et_3_N was added. These results indicate that the reaction between amide‐sulfoxonium ylides with electron‐deficient alkynes is different from the classical Michael addition process. Moreover, changing the reaction temperature from 30 °C to 20 or 40 °C did not influence the regio‐ and stereoselectivities, but a lower yield of addition product **3** was observed (see details in, Table S2, Supporting Information).

**Figure 3:**
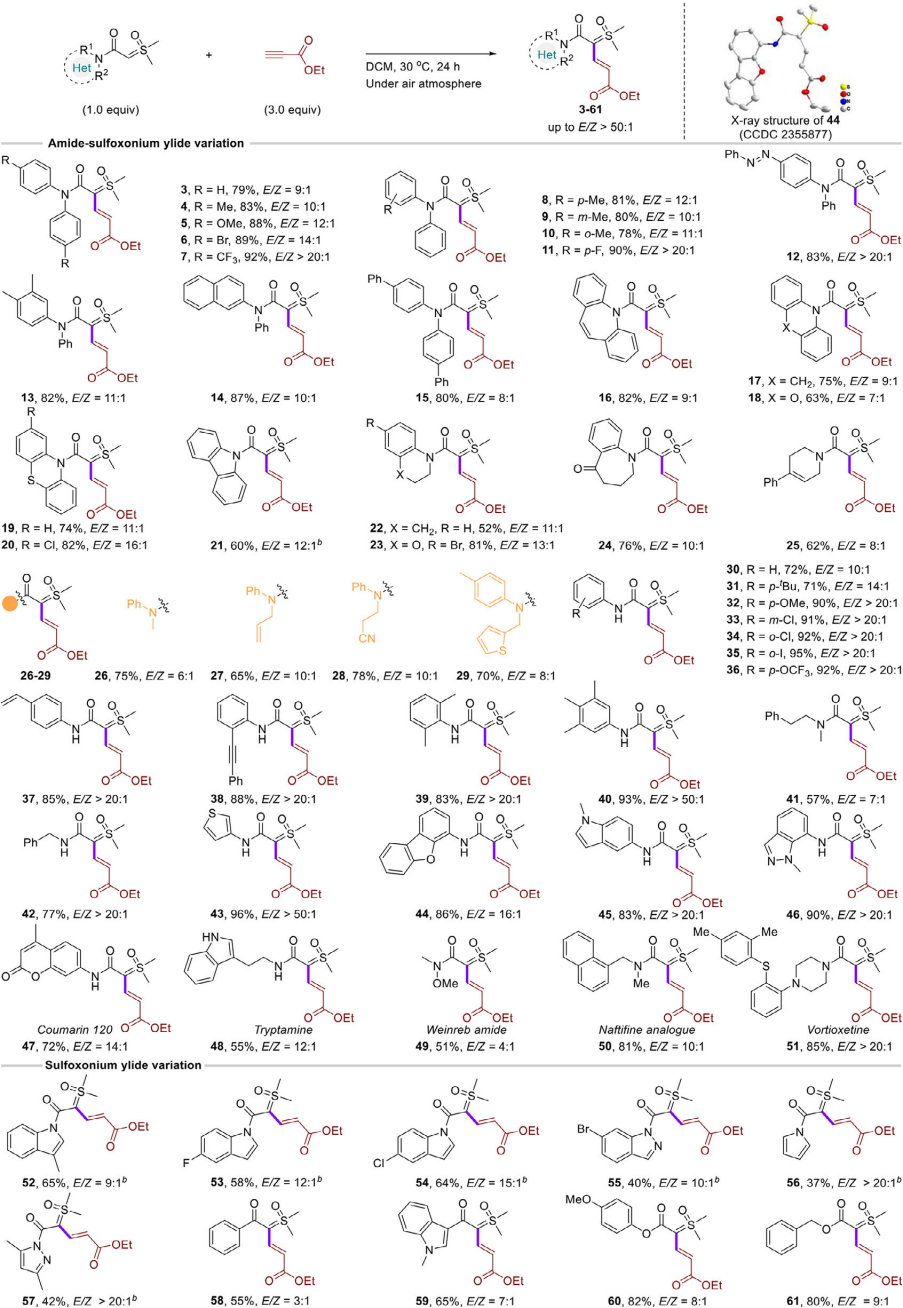
Scope of amide‐sulfoxonium ylides. *^a^* Reaction conditions: amide‐sulfoxonium ylide (0.1 mmol), ethyl propiolate (0.3 mmol), DCM (0.5 mL) at 30 °C for 24 h under air atmosphere. The *E*/*Z* ratios were determined by UPLC analysis of the crude reaction mixture, isolated yields were given. *^b^* 50 °C.

After establishing the optimized conditions, we set out to investigate the scope of this catalyst‐free hydrocarbonation of alkynes protocol. As shown in **Figure** **3**, a variety of symmetric and asymmetric diarylamide‐sulfoxonium ylides bearing a functionality, such as methyl, methoxyl, bromo, trifluoromethyl, fluoro, and azo, on the aryl ring at position *para*, *meta*, or *ortho* were compatible with the optimized conditions, furnishing the desired product (**4–12**) in good to excellent yields with high regio‐ and stereoselective control. It is worth noting that the electron withdrawing‐groups have higher reactivity than electron donating‐groups, resulting in excellent stereoselectivity (with *E*/*Z* ratios > 20:1). The steric hindrance has little effect on the reaction (**8–10**). The reaction of a disubstituted benzene ring or polycyclic arylamide‐sulfoxonium ylides also proceeded smoothly and delivered the corresponding products (**13**–**15**). Moreover, various seven/six/five (hetero)cycle amide‐sulfoxonium ylides were also suitable for this reaction, and good yields with high regioselectivities were achieved (**16**–**21**). The cyclic amide‐sulfoxonium ylides that contained tetrahydroquinoline, benzomorpholine, tetrahydro‐benzo[*b*]azepin‐5‐one, or tetrahydropyridine were also tolerated to give **22–25** in moderate to high yields. The alkyl group of disubstituted amide‐sulfoxonium ylides could bear a range of functional groups including methyl, alkenyl, cyano, and thiophene, which all worked well under the present reaction conditions, furnishing the corresponding target products (**26–29**) in 65–78% yields with good chemical selective control. To our delight, a variety of NH‐amide‐sulfoxonium ylides serve as suitable reaction partners in this metal‐free hydrocarbonation to generate highly regio‐ and stereoselective *E*‐alkene substituted amide‐sulfoxonium ylide products (**30–38**). Various functional groups such as *tert*‐butyl, methoxyl, chloro, iodine, trifluoromethoxy, alkenyl, and alkynyl were compatible with the standard reaction conditions. Interestingly, di‐ and trisubstituted phenyl ring NH‐amide‐sulfoxonium ylides could efficiently access target products (**39** and **40**) in 83% and 93% yields, respectively. It is worth mentioning that alkyl di‐ or mono‐substituted amide‐sulfoxonium ylides (**41** and **42**) were also applicable substrates, which further confirms the universality of this reaction system. Heterocyclic substrates such as thiophene, dibenzofuran, indole, and indazole were suitable for efficiently constructing target products (**43–46**) with excellent regio‐ and stereoselectivities. Importantly, drug fragments and natural product derivatives were successfully incorporated into the metal‐free hydrocarbonation process with high selectivity (**47–51**). X‐ray crystallographic analysis determined the absolute configuration of **44** (CCDC no. 2355877) and those of other products were assigned by analogy. Subsequently, various N‐heterocyclic sulfoxonium ylides were examined. For example, the reaction between Me‐, F‐, or Cl‐substituted indole sulfoxonium ylides and ethyl propiolate (**2a**) afforded the desired functionalized vinyl sulfoxonium ylide products (**52–54**) in good yields, with *E*/*Z* ratios up to 15:1. It should be noted that other N‐heterocyclic sulfoxonium ylides (i.e., indazole, pyrrole, and pyrazole) were also applicable substrates in the current system, and the desired products (**55–57**) were obtained moderate outcomes. Furthermore, when keto and ester sulfoxonium ylide substrates were employed under the same reaction conditions, surprisingly, furnishing the corresponding alkene products (**58–61**) in 55–82% yields with good selectivity.^[^ ^15^ ^]^ Notably, the separation of alkenyl sulfoxonium ylide in the previous report has failed due to low yield.^[^ ^7j^ ^]^ Moreover, while we were preparing our manuscript, the Burtoloso group reported a related method for synthesizing α‐keto/ester vinyl sulfoxonium ylides; however, this method required argon gas protection and heating to 60 °C.^[^ ^15^ ^]^

**Figure 4:**
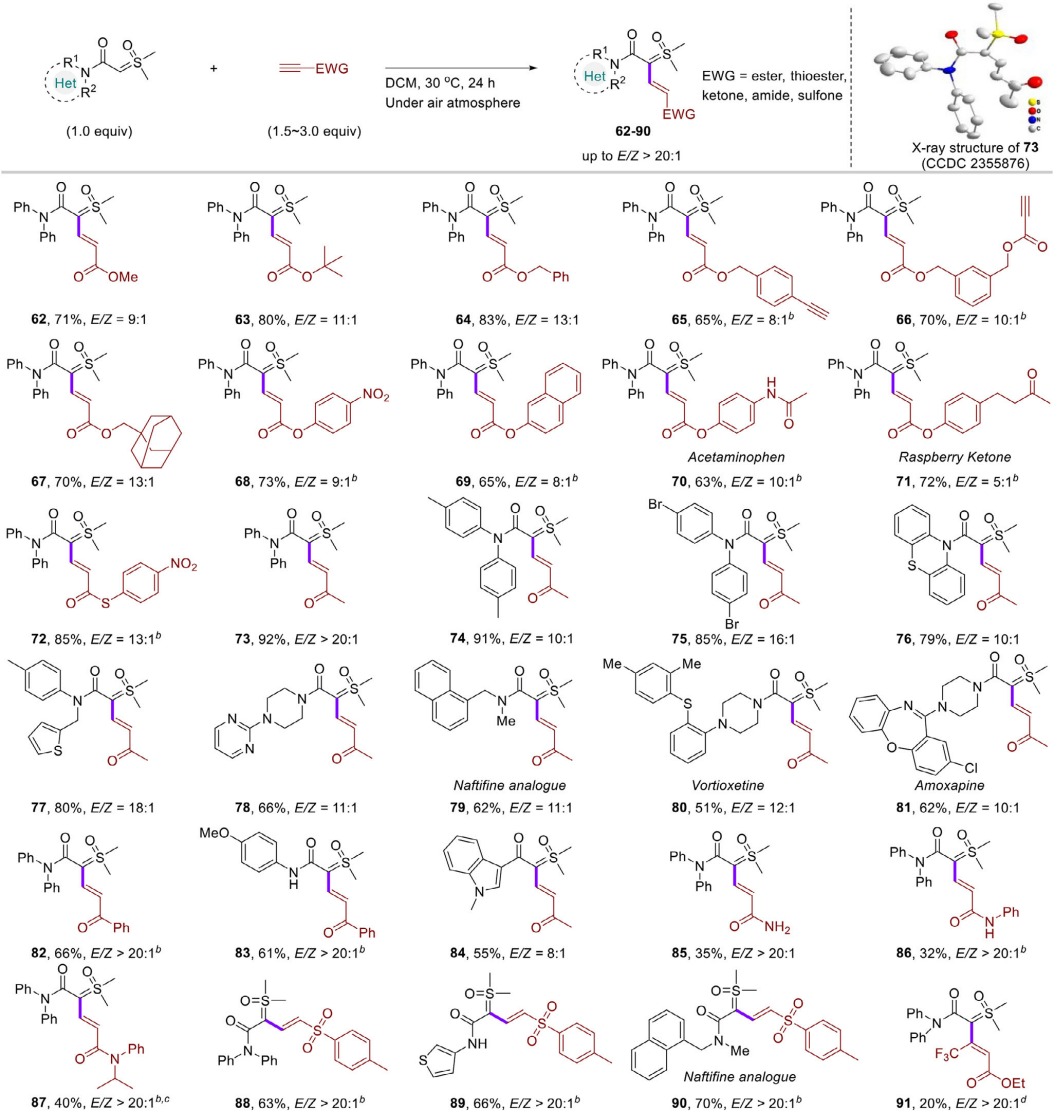
Scope of terminal alkynes. *^a^* Reaction conditions: amide‐sulfoxonium ylide (0.1 mmol), terminal alkyne (0.3 mmol), DCM (0.5 mL) at 30 °C for 24 h under air atmosphere. The *E*/*Z* ratios were determined by UPLC analysis of the crude reaction mixture, isolated yields were given. *^b^* terminal alkyne (0.15 mmol). *^c^* 60 °C. *^d^* DCE (0.5 mL), 60 °C.

Next, we investigated the scope of alkynes conjugated to esters, ketones, or amides under the standard reaction conditions (**Figure** **4**). The alkyl‐substituted propargylic carboxylates including methyl, *tert*‐butyl, benzyl, and adamantyl, which exhibited excellent compatibility with the reaction system (**62**–**67**), and similarly, the aryl‐substituted propargylic carboxylates effectively produced the desired products **68** and **69** with great regio‐ and stereoselectivities. Notably, exclusive chemoselectivities were observed when the substrate contained an unactivated or reactive terminal alkynes (**65** and **66**). The high functional group tolerance renders this approach applicable for late‐stage functionalizations (LSFs) of natural and medicinal molecules such as acetaminophen (**70**) and raspberry ketone (**71**). Thioester substituted terminal alkyne gave the product (**72**) in 85% yield with high regio‐ and stereoselectivity. Alkyl and aryl enones could react well with a variety of (hetero)amide or keto sulfoxonium ylides under present reaction conditions, and afforded the corresponding target products (**73**–**84**) in 51–92% yields with excellent selectivity. X‐ray crystallographic analysis determined the absolute configuration of **73** (CCDC no. 2355876) and those of other products were assigned by analogy. Interestingly, acetylenic amides were well tolerated with alkene products containing primary amide (**85**), secondary amide (**86**), and tertiary amide (**87**) formed effectively. Furthermore, when ethynyl *p*‐tolyl sulfone was employed, the target products (**88**–**90**) were obtained in good yields with excellent regio‐ and stereoselectivities. Perhaps due to steric hindrance from the substituents, when the internal alkyne was used as a reaction substrate, it yielded the corresponding target product (**91**) in moderate yield at 60 °C. Thus, this new metal‐free hydrocarbonation strategy provides a concise and selective access to various functionalized vinyl substituted amide‐sulfoxonium ylides and could be further used for late‐stage functionalization of target molecules.

**Figure 5:**
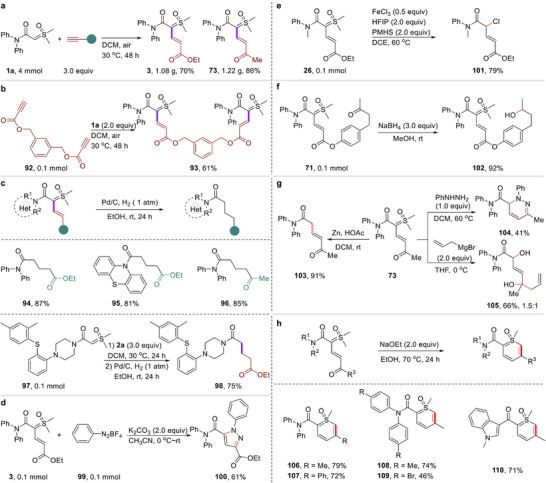
Scale‐up reactions and diverse transformations of the products. a) Gram‐scale reaction for the synthesis of **3** and **73**. b) Synthesis of bis‐alkene amide‐sulfoxonium ylide. c) Pd‐catalyzed hydrogenation reaction for the synthesis of 1,5‐dicarbonyls. d) Transformation of product **3**. e) Transformation of product **26**. f) Transformation of product **71**. g) Transformations of product **73**. h) Synthesis of polyfunctional thiabenzene 1‐oxide compounds.

To further demonstrate the synthetic value of this general, convenient, and easily handled strategy, the gram‐scale syntheses of **3** and **73** were conducted under present reaction conditions. These gram‐scale reactions smoothly provided desired alkenyl amide‐sulfoxonium ylide products **3** and **73** in good yields with maintained chemo‐, stereo‐, and regioselectivities, as shown in **Figure** **5**a. The reaction of the bis‐benzyl propionate **92** with **1a** was carried out; interestingly, and furnished the corresponding bis‐alkene substituted product **93** in 61% yield (Figure 5b). 1,5‐Dicarbonyl compounds are frequently used for the Paal–Knorr type synthesis to construct pyridines, exists widely in a large number of natural products and active drug molecules (e.g., antitumor, anti‐HIV, antibacterial, and etc.).^[^ ^16^ ^]^ However, approaches to construct 1,5‐dicarbonyls mainly rely on oxidative coupling of the α‐carbonyl alkyl radicals with alkenes or homologation of 1,3‐dicarbonyls with alkenes,^[^ ^17^ ^]^ which critically limits the substituent diversity. To this end, our method offers a straightforward and green protocol to access such compounds, as shown in Figure 5c, by Pd/C‐catalyzed reduction‐hydrogenation reactions,^[^ ^18^ ^]^ delivering the corresponding 1,5‐dicarbonyl products (**94**–**96**) in high yields. Importantly, one‐pot reaction for the preparation of complex target product **98** could also be achieved by continuous hydrocarbonation and reduction‐hydrogenation reactions. The simplicity and efficiency of the reaction were further highlighted, providing a convenient and flexible platform for subsequent molecular modifications, thus demonstrating the practical significance of this method. As shown in Figure 5d, the reaction of **3** with phenyl diazonium tetraffuoroborate gave the corresponding pyrazole product **100** in 61% yield.^[^ ^19^ ^]^ Interestingly, when the reaction of compound **26** was performed in the presence of FeCl₃ (50 mol %) in DCE at 60 °C, an α‐chloro‐substituted amide **101** was produced in 79% yield (Figure 5e). In addition, selective reduction of the ketone group in the compound **71**, giving the corresponding alcohol product **102** in 92% yield (Figure 5f).^[^ ^20^ ^]^ Moreover, the ketone‐substituted vinyl sulfoxonium ylide product **73** can readily undergo several transformations to access a variety of functional molecules (Figure 5g). For example, the compound **73** was further transformed into 5‐oxo‐*N,N*‐diphenylhex‐3‐enamide (**103**) in 91% yield in the reduction with zinc and acetic acid.^[^ ^7h^ ^]^ The reaction of **73** with phenylhydrazine afforded cyclized product **104** in 41% yield. Additionally, the addition reaction of **73** with allylMgBr, followed by hydrolysis, generated the corresponding vinyl‐substituted amide bearing dihydroxyl groups (**105**) in 66% yield. As shown in Figure 5h, the ketone and methylide moieties of alkene sulfoxonium ylide could be selectively dehydration‐cyclization with NaOEt to generate the corresponding thiabenzene 1‐oxide products (**106–110**) in 46–79% yields.^[^ ^21^ ^]^

**Figure 6:**
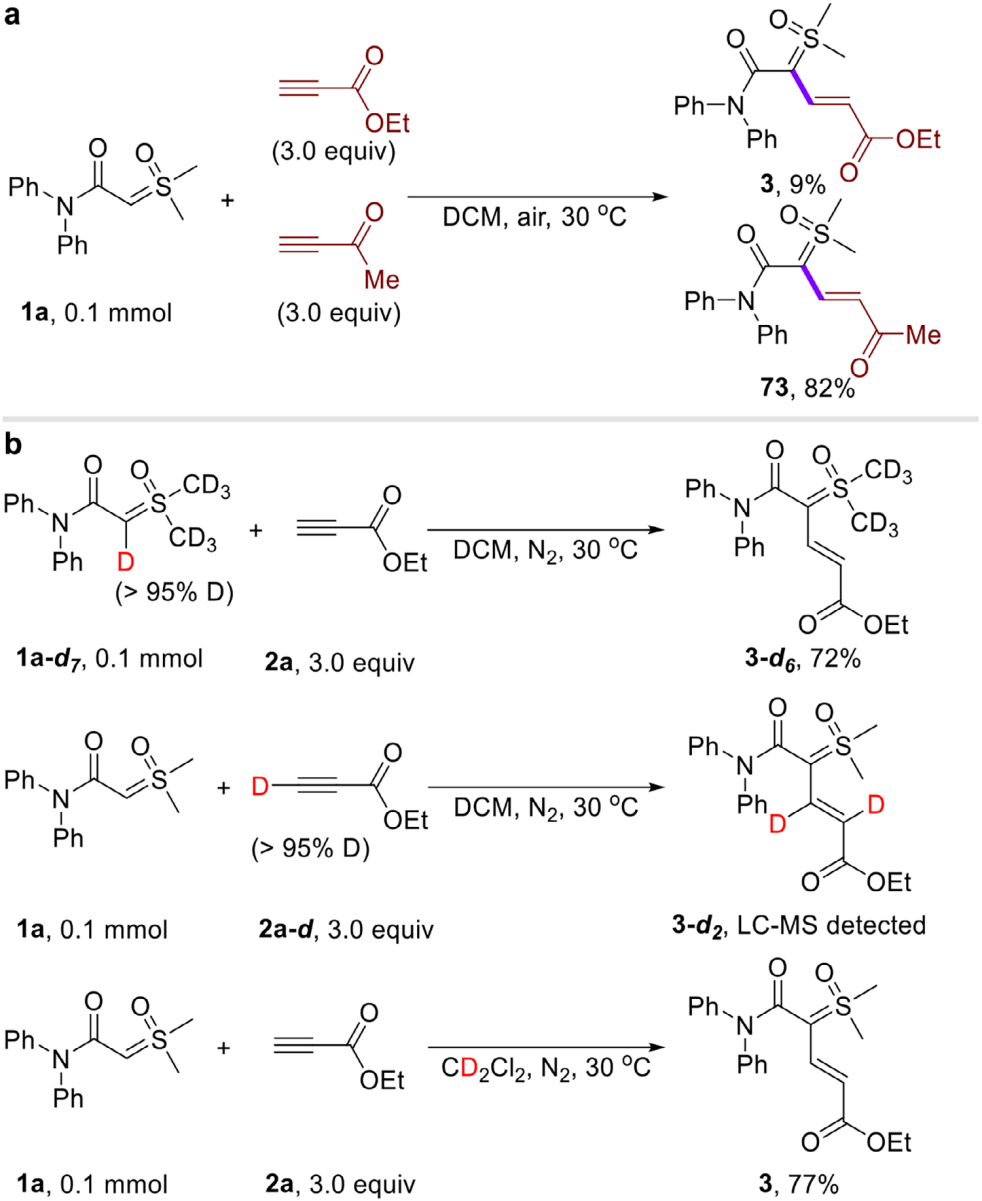
Mechanism investigations. a) Competition experiment. b) Deuterium labeling experiments.

To explore the mechanism of this reaction, several control experiments were carried out. First, when acetylenic ester (**2a**) and acetylenic ketone were employed to react with amide‐sulfoxonium ylide (**1a**) in one‐pot procedure, **73** was produced in 82% yield, while the product **3** was only obtained in 9% yield (**Figure** **6**a), indicating that acetylenic ketones show higher reactivity in this reaction. Under a nitrogen atmosphere, the reaction was conducted by replacing amide‐sulfoxonium ylide (**1a**) with its deuterated counterpart, amide‐sulfoxonium ylide‐*d_7_* (**1a‐*d_7_***, D content: >95%), yielding the deuterated product (**3‐*d_6_***) in 72% yield (Figure 6b). Notably, the position of the alkene remained undeuterated. Then, the D atom of ethyl propiolate‐*d* (**2a‐*d***, D content: >95%) could be smoothly converted into the corresponding deuterated product (**3**‐***d_2_***) under the reaction conditions (LC‐MS detected, see Figure S5. Supporting Information). Finally, conducting the reaction in CD₂Cl₂ resulted in the formation of the corresponding product (**3**) with a yield of 77%. These results indicate that the D atom on C‐2 of the alkene originated from another molecule of terminal alkyne in this hydrocarbonation reaction (vide infra).

**Figure 7:**
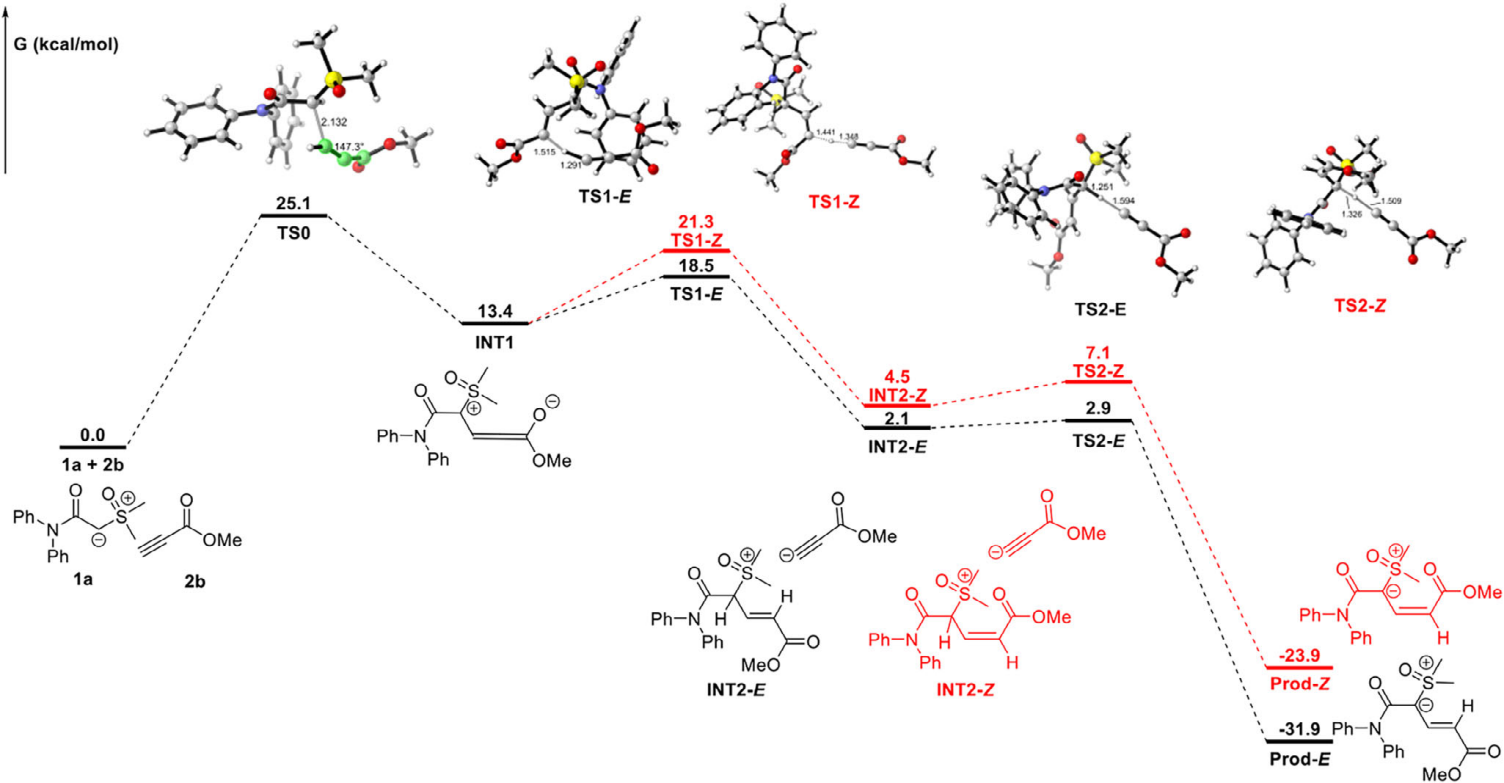
Free energy changes of the possible reaction pathways. Calculations were performed at the PWPB95‐D3(BJ)/def2‐TZVP:SMD(DCM) // wB97XD/6‐31+G^**^:IEFPCM(DCM) level of theory.

To better understand the reaction mechanism, we conducted density functional theory (DFT) calculations (for computational details, see Table S6, Supporting Information). As shown in **Figure** **7**, the amide‐sulfoxonium ylide (**1a**) underwent nucleophilic addition with methyl propiolate (**2b**), thereby generating an allene intermediate (**INT1**) through a transition state (**TS0**) that has an energy barrier of 25.1 kcal mol^−1^. It then reacted with another molecule of methyl propiolate (**2b**) via an intermolecular proton transfer process, which through a transition state (**TS1‐*E***) with an energy barrier of 5.1 kcal mol^−1^, resulting in the formation of an alkene intermediate (**INT2‐*E***). In contrast, the formation of alkene intermediate (**INT2‐*Z***) with **2b** requires a higher‐energy transition state (7.9 kcal mol^−1^). Finally, the acetylene anion rapidly captured hydrogen on the α‐carbon of intermediate (**INT2‐*E***) to give the desired product (**Prod‐*E***) via a transition state (**TS2‐*E***) with an energy barrier of 0.8 kcal mol^−1^. Furthermore, a possible reaction mechanism was proposed and was presented in Scheme S1 (Supporting Information) based on the above results and literature reports.^[^ ^7^, ^22^ ^]^

## 3 Conclusion

In summary, using HTE we have developed a novel, catalyst‐ and base‐free method for direct hydrocarbonation of terminal alkynes with amide‐sulfoxonium ylides. A diverse array of easily accessible ester, thioester, ketone, amide, and sulfone substituted alkynes were successfully converted into the corresponding alkenyl stabilized sulfoxonium ylides in high yields under mild conditions. Notably, various valuable 1,5‐dicarbonyl compounds and thiabenzene 1‐oxides were also successfully synthesized via product transformation. This metal‐free reaction process, which is operationally simple and environmentally friendly, is characterized by its broad substrate scope, good functional group compatibility, and excellent regio‐ and stereoselectivity. The combined experimental and computational studies shed light on the reaction mechanism and the origin of these high selectivities. Furthermore, late‐stage functionalization of complex biologically active molecules and natural products demonstrates the potential applications of this approach in drug development.

## Conflict of Interest

The authors declare no conflict of interest.

## Supporting information



Supporting Information

## Data Availability

The data that support the findings of this study are available in the supplementary material of this article.
