# Supplementary material for: Catalyst‐Free Direct Hydrocarbonation of Terminal Alkynes Toward E‐Alkene Substituted Stabilized Sulfoxonium Ylides
Source: Adv Sci (Weinh). 2025 Apr 2;12(22):2417362. doi: 10.1002/advs.202417362 (PMC12165105; doi:10.1002/advs.202417362)

ADVANCED SCIENCE
Open Access

Supporting Information

for *Adv. Sci.*, DOI 10.1002/advs.202417362

Catalyst-Free Direct Hydrocarbonation of Terminal Alkynes Toward *E*-Alkene Substituted Stabilized Sulfoxonium Ylides

Haiting Wu, Yougen Xu, An Lin, Jingyuan Liu, Huanjun Chen, Shimin Xie and Lebin Su**

Supporting Information

Catalyst-Free Direct Hydrocarbonation of Terminal Alkynes toward *E*-Alkene Substituted Stabilized Sulfoxonium Ylides

Haiting Wu,[a,b,†] Yougen Xu,[b,†] An Lin,[b] Jingyuan Liu,[b] Huanjun Chen,[b] Shimin Xie,[c,*] and Lebin Su[a,b,*]

[a]School of Pharmaceutical Sciences, Guangzhou Laboratory, Guangzhou Medical University, Guangzhou 511436, China

[b]Bioland Laboratory, Guangzhou 510005, China

[c]State Key Laboratory of Chemo/Biosensing and Chemometrics, College of Chemistry and Chemical Engineering, Hunan University, Changsha 410082, China

E-mail: su_lebin@gzlab.ac.cn; xsm2020@hnu.edu.cn

Table of Contents

1. General Information S2
2. General Procedure for the Preparation of Sulfoxonium Ylides S3
3. General Experimental Procedure S8
4. X-ray Crystallographic Data of **44** S16
5. X-ray Crystallographic Data of **73** S17
6. Control Experiments S18
7. Computational Details S21
8. Characterization Data for the Products S44
9. Characterization Data for Amide-Sulfoxonium Ylides S97
10. References S114
11. Copies of ^{1}H, ^{13}C and ^{19}F NMR Spectra of the Products S117

1. General Information

Materials. Reactions were carried out under an air atmosphere in oven-dried 4 mL glass tubes unless otherwise specified. The heat source is IKA magnetic stirrer with RCT Basic. Reagents were purchased from commercial suppliers (Energy, Aladdin, Bidepharm, Alfa Aesar, Sigma-Aldrich, and J&K Scientific) and used with no further purification. Anhydrous solvents in sure-seal bottle were purchased from Energy and used with no further purification. Organic solutions were concentrated under reduced pressure on a Heidolph rotary evaporator using a water bath.

Instruments. ^{1}H and ^{13}C NMR spectra were recorded at 600 MHz (^{13}C at 150 MHz) on Bruker Avance III 600 MHz spectrometer, as indicated. NMR spectra run in solutions of deuterated chloroform ($CDCl_3$) with residual chloroform as internal standard (7.26 ppm for ^{1}H, and 77.00 ppm for ^{13}C) or in solutions of deuterated dimethyl sulfoxide (DMSO-d_6) with residual dimethyl sulfoxide as internal standard (2.50 ppm for ^{1}H, and 39.50 ppm for ^{13}C), and chemical shifts were reported in parts per million (ppm). ^{19}F NMR spectra were recorded on a Bruker Avance III 600 MHz (^{19}F at 564 MHz), and were reported unreferenced. Abbreviations for signal multiplicity are as follow: s = singlet, d = doublet, t = triplet, q = quartet, m = multiplet, dd = doublet of doublet, etc. Coupling constants (*J* values) were calculated directly from the spectra. Reactions were analyzed using a Waters Acquity UPLC. Column: Acquity UPLC BEH C18 1.7 µm 2.1 × 50 mm (Part No. 186002350), Mobile Phase A: 2.0 mL formic acid + 3998 mL Water, Mobile Phase B: 4000 mL MeCN, Weak Wash: 100 mL MeCN + 900 mL Water. The instrument was equipped with an SQ Detector 2 with electrospray ionization (ESI) source in the positive mode. Flash column chromatography was performed on silica gel (300–400 mesh) with the solvents given in the procedures. Thin layer chromatographic (TLC) analysis was performed with glass-backed silica gel plates, visualizing with UV light (254 nm). Liquid was handled with TECAN EVO-200 equipped with 96-channel disposable tips (Air LiHa) pipetting device. The high resolution mass spectra (HRMS) were measured on a Waters Xevo G2-XS using electrospray ionization time-of-flight (ESI-TOF). The single-crystal X-ray diffraction was conducted on a D8 Quest X-ray single-crystal diffractometer.

2. General Procedure for the Preparation of Sulfoxonium Ylides

A) Synthesis of Amide-Sulfoxonium Ylides

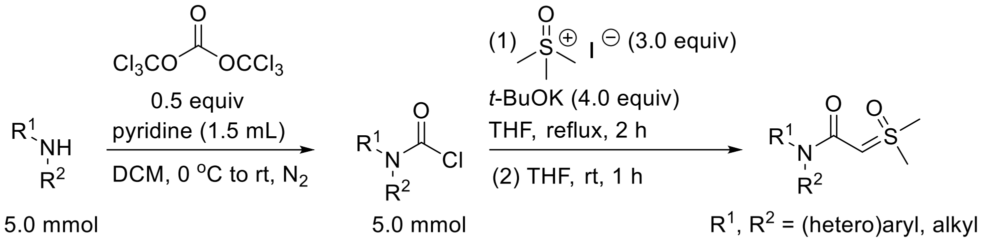

Method 1:

The preparation of intermediate acyl chloride compound was performed according to a literature reference.[1] A 100 mL two-necked flask, equipped with a magnetic stir bar, was evacuated and purged with nitrogen gas three times. Triphosgene (2.5 mmol) and anhydrous DCM (20 mL) were added to the flask. The mixture was cooled at 0 °C and anhydrous pyridine (1.5 mL) was slowly added to the flask. After stirring for 15 min at 0 °C, secondary amine (5 mmol) was slowly added to the mixture. The mixture was warmed to room temperature and stirred for 6 h at room temperature. The reaction mixture was carefully quenched by 1.0 M HCl (5 mL) and was extracted with DCM (3 × 10 mL). The organic layer was washed with water and brine, then dried over Na_2SO_4 and concentrated in vacuo. This material was then used in the next step without further purification.

The preparation of amide-sulfoxonium ylides was performed according to a literature reference.[2] The *t*-BuOK (4.0 equiv) was suspended in THF (1.0 M solution) and trimethylsulfoxonium iodide (3.0 equiv) at once in a three-necked flask fitted with a condenser. The mixture was refluxed for 2 h resulting in a yellow cloudy suspension. The reaction was cooled down to 0 °C and carbamoyl chloride (1.0 equiv) was added dropwise as a solution in THF (1.0 M solution). After warming up to room temperature the mixture was stirred for 3 h. Afterwards volatiles were removed under reduced pressure and the residue was dissolved in equal amounts of ethyl acetate and H_2O. The crude product was with ethyl acetate (3 × the volume of the aqueous phase). The organic layer was then dried over anhydrous Na_2SO_4 and concentrated in vacuo. The crude product was purified by flash column chromatography.

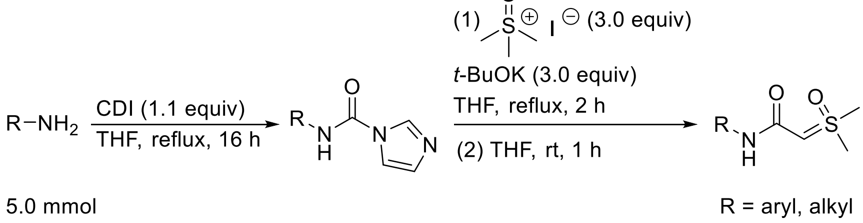

Method 2:

The preparation of this compound was performed according to a literature reference.[3] To a flame-dried 2-necked round bottomed flask, containing 1,1'-carbonyldiimidazole (CDI, 5.5 mmol, 1.1 equiv) solution in dry THF (20 mL)

was added the primary amine (5 mmol, 1.0 equiv). The mixture was stirred at reflux for 16 h. After cooling to room temperature, the solvent was evaporated under vacuum and the crude was purified by flash chromatography to give the carboxamide intermediate. Under N_2 atmosphere, trimethylsulfoxonium iodide (3.0 equiv) was suspended in dry THF (10 mL/mmol) in a flame-dried round bottom flask that was protected from light with aluminium foil. The *t*-BuOK (3.0 equiv) was added and the mixture was stirred at reflux for 2 h. After cooling to room temperature, a solution of the carboxamide intermediate (1.0 equiv) in THF (2.0 mL/mmol) was added dropwise to the mixture. After stirring at room temperature for 1~2 h, the mixture was filtered (elution DCM) and the solvent evaporated. Purification by flash chromatography gave the desired amide-sulfoxonium ylides.

The following amide-sulfoxonium ylides were used in this study and were prepared according to the previous literature.[1–4] Compounds **S1–S3**, **S6**, **S12**, **S14-17**, **S19–S20**, **S24**, and **S28** have been our previous reported,[4] and their spectroscopic data matched those reported in the literature. The remaining compounds are new compounds. All new compounds have been characterized by ^{1}H NMR and ^{13}C NMR.

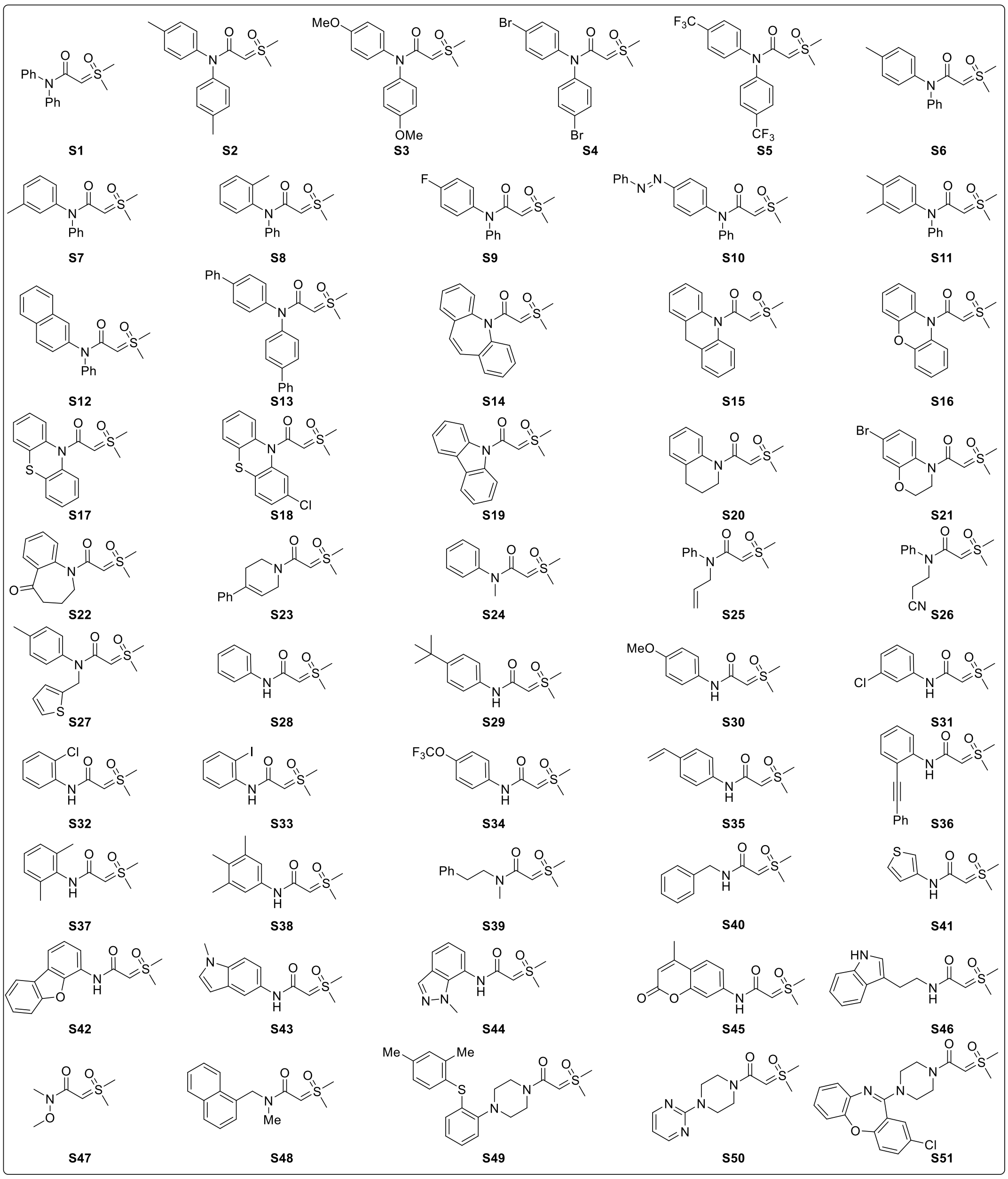

B) Synthesis of N-Heterocyclic Sulfoxonium Ylides

The following N-heterocyclic sulfoxonium ylides were used in this study and were prepared according to the previous literature.[4] Compounds **S52–S57** have been our previous reported,[4] and their spectroscopic data matched those reported in the literature.

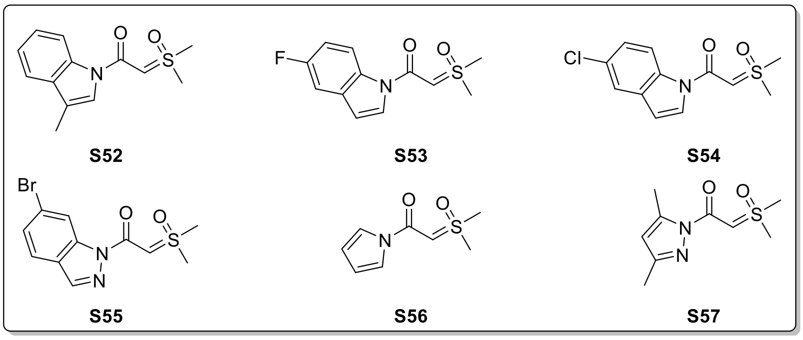

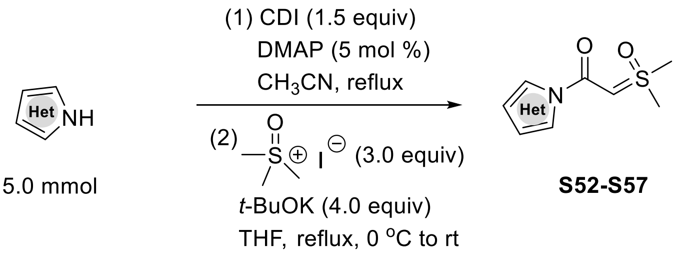

Preparation of methylide solution: The reaction was carried out under air. The *t*-BuOK (4.0 equiv) was suspended in THF (1.0 M solution) and trimethylsulfoxonium iodide (3.0 equiv) at once in a three-necked flask fitted with a condenser. The mixture was heated under reflux for 2 h, resulting in formation of a yellow cloudy suspension. The reaction solution was cooled down to 0 °C.

Preparation of N-heterocyclic sulfoxonium ylide: To a 100 mL round bottle charged with a magnetic stir bar, was added N-heterocycle (5.0 mmol, 1.0 equiv), 1,1'-carbonyldiimidazole (CDI, 7.5 mmol, 1.5 equiv), and 4-dimethylaminepyridine (DMAP, 5 mol %). Then, anhydrous CH_3CN (30 mL) was added to bottle under the protection of N_2 atmosphere. The system was refluxed at 85 °C for 10 h. After cooled to room temperature, methylide solution (3.0 M in THF, 2.0 equiv) was added and then stirred at room temperature for another 3 h (when the most of indole was consumed detected by TLC). Afterwards volatiles were removed under reduced pressure and the residue was dissolved in equal amounts of ethyl acetate and H_2O. The crude product was extracted with ethyl acetate (3 × the volume of the aqueous phase). The organic layer was then dried over anhydrous Na_2SO_4 and concentrated in vacuo. The crude product was purified by flash column chromatography.

C) Synthesis of Other Sulfoxonium Ylides

The following sulfoxonium ylides were used in this study and were prepared according to the previous literature.[3,5] Compounds **S58-S61** have been our previous reported,[4,5] and their spectroscopic data matched those reported in the literature.

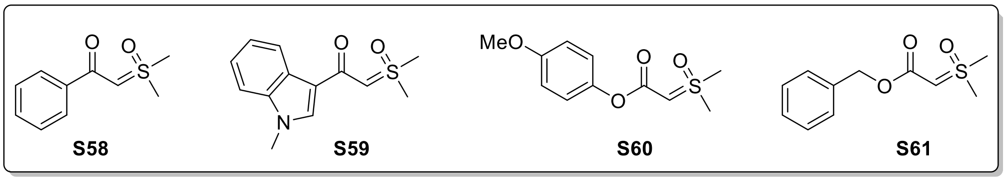

a) Synthesis of sulfoxonium ylides from carboxylic acids

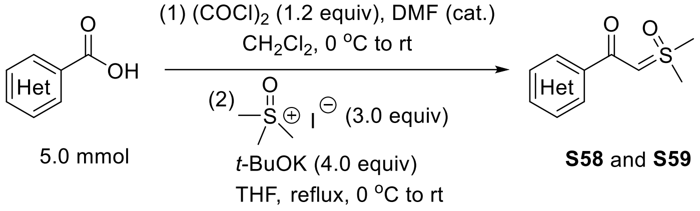

A 100 mL oven-dried round bottom flask, equipped with a magnetic stir bar, was purged with nitrogen gas and charged with carboxylic acid (5.0 mmol) in anhydrous DCM (20 mL), which was added DMF (3 drops) at 0 °C and $(COCl)_2$ (1.2 equiv) was subsequently added dropwise by syringe. After 15 min, the reaction mixture was allowed to warm to room temperature overnight. After removal of the volatiles under vacuum, the crude product was used directly without any further purification.

The *t*-BuOK (4.0 equiv) was suspended in THF (1.0 M solution) and trimethylsulfoxonium iodide (3.0 equiv) at once in a three-necked flask fitted with a condenser. The mixture was refluxed for 2 h resulting in a yellow cloudy suspension. The reaction was cooled down to 0 °C and acyl chloride was added dropwise as a solution in THF (1.0 equiv, 1.0 M). After warming up to room temperature the mixture was stirred for 3 h. Afterwards volatiles were removed under reduced pressure and the residue was dissolved in equal amounts of ethyl acetate and H_2O. The crude product was extracted with ethyl acetate (3 × the volume of the aqueous phase), and the organic layer was then dried over anhydrous Na_2SO_4 and concentrated in vacuo. The crude product was purified by flash column chromatography on silica gel to give the desired sulfoxonium ylides.

b) Synthesis of sulfoxonium ylides from acyl chlorides

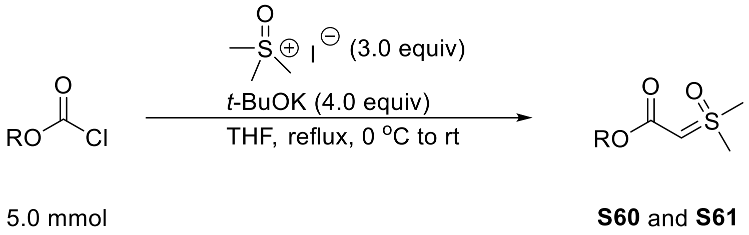

The *t*-BuOK (4.0 equiv) was suspended in THF (1.0 M solution) and trimethylsulfoxonium iodide (3.0 equiv) at once in a three-necked flask fitted with a condenser. The mixture was refluxed for 2 h resulting in a yellow cloudy suspension. The reaction was cooled down to 0 °C and acyl chloride was added dropwise as a solution in THF (1.0

equiv, 1.0 M). After warming up to room temperature the mixture was stirred for 3 h. Afterwards volatiles were removed under reduced pressure and the residue was dissolved in equal amounts of ethyl acetate and H_2O. The crude product was extracted with ethyl acetate (3 × the volume of the aqueous phase), and the organic layer was then dried over anhydrous Na_2SO_4 and concentrated in vacuo. The crude product was purified by flash column chromatography on silica gel to give the desired sulfoxonium ylides.

D) Synthesis of Propionate Compounds

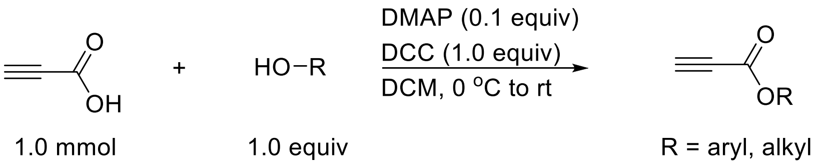

The preparation of this compound was performed according to a literature reference.[6] In a 100 mL two-necked round-bottom flask, 4-dimethylaminopyridine (DMAP, 12.2 mg, 0.1 mmol), dicyclohexylcarbodiimide (DCC, 206.3 mg, 1.0 mmol) and in DCM (3.0 mL) was cooled to 0 °C under a nitrogen atmosphere. A solution of a propiolic acid (61.5 µL, 1.0 mmol) and alcohol or phenol (1.0 mmol) in DCM (3.0 mL) was added dropwise slowly over 15 min. Then the reaction was allowed to warm to room temperature and stirred for 4 h. Upon completion, the mixture was filtered through a layer of Celite, the filtrate concentrated in vacuo, and purification by silica column chromatography afforded the corresponding propiolate compounds.

3. General Experimental Procedure

A) Reaction Optimization

Table S1. HTE screening of catalyst and solvent for hydrocarbonation reaction of 1a and 2a[a]

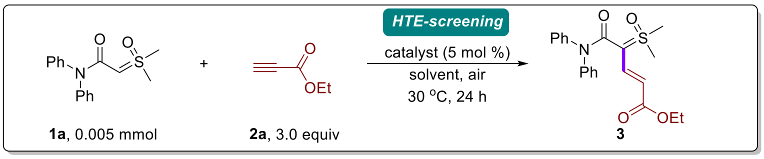

	$[Rh(cod)Cl]_2$	$[Rh(OAc)_2]_2$	$Ru(cod)Cl_2$	$Ru(acac)_3$	$RuCl_3$	$[Ir(cod)Cl]_2$	$Pd(OAc)_2$	$Cu(OAc)_2$	CuCl	$FeBr_2$	$Co(acac)_2$	none
DCM	43	40	31	28	38	37	20	26	31	43	33	83
DCE	45	38	26	17	40	30	27	33	35	22	36	49
$CHCl_3$	29	20	18	21	22	19	15	21	16	18	21	44
THF	50	46	40	28	25	18	25	28	30	25	40	56
1,4-dioxane	33	42	52	25	30	25	17	41	45	0	42	59
CH_3CN	72	63	60	62	60	42	22	50	56	33	49	78
DMF	59	50	46	66	55	36	8	25	30	37	52	65
DMSO	30	36	27	35	32	17	0	11	16	10	6	38

numbers indicated yield (%) of **3**

[a]Reaction conditions: **1a** (0.005 mmol), **2a** (0.015 mmol), catalyst (5 mol %) in solvent (100 µL) at 30 °C under air for 24 h. Yields were determined by UPLC-MS analysis on the reaction crude using indole as internal standard.

Table S2. Study of the reaction conditions[a]

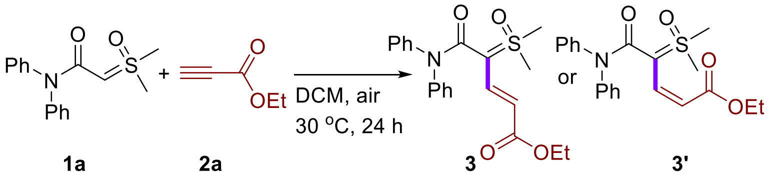

entry	variation from the standard conditions	yield (%)[b]	3:3'[c]
1	none	81	9:1
2	with 1.0 equiv Na_2CO_3	62	8:1
3	with 1.0 equiv ^{t}BuOK	6	-
4	with 1.0 equiv Et_3N	10	-
5	20 °C, instead of 30 °C	61	10:1
6	40 °C, instead of 30 °C	75	9:1
7	N_2, instead of air	80	9:1

[a]Standard reaction conditions: **1a** (0.1 mmol), **2a** (0.3 mmol), DCM (0.5 mL) at 30 °C for 24 h under air. [b]Yield of isolated product. [c]Determined by UPLC analysis of the crude reaction mixture.

To our delight, scale-up of the reaction from 5 µmol (microscale-HTE experiment) to 0.1 mmol scale was demonstrated in a similar 81% yield (Table S2, entry 1). Notably, the reaction was inhibited when Na_2CO_3, ^{t}BuOK, or Et_3N was added (entries 2–4). These results indicate that the reaction between amide-sulfoxonium ylides with electron-deficient alkynes is different from the classical Michael addition process. Moreover, changing the reaction temperature from 30 °C to 20 or 40 °C did not influence the regio- and stereoselectivities, but a lower yield of addition product **3** was observed (entries 5 and 6). The reaction could also proceed smoothly under N_2 atmosphere, producing the target product **3** in 80% yield (entry 7).

B) General Experimental Procedure for Hydrocarbonation of Terminal Alkynes with Amide-Sulfoxonium Ylides

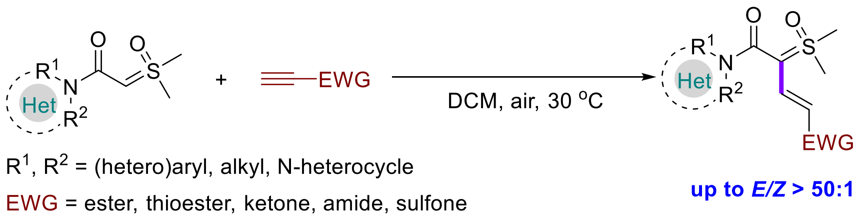

An oven dried glass tube of 4 mL equipped with a magnetic stir bar and was charged with amide-sulfoxonium ylide (0.10 mmol, 1.0 equiv) in DCM (0.5 mL); then alkyne (0.30 mmol, 3.0 equiv) was added. The reaction mixture was sealed and stirred (820 r/min in IKA RCT basic) at 30 ºC for 24 h under air atmosphere. After completion, the reaction mixture was recovered to room temperature. The crude product was extracted with ethyl acetate (3 × 3.0 mL), and the solution was washed with saturated solution of NaCl (3.0 mL), and the organic layer was dried over anhydrous Na_2SO_4 and concentrated in vacuo. The crude product was purified by flash column chromatography on silica gel (eluent: petroleum ether/ethyl acetate) to give the corresponding products.

Table S3. The unsuccessful substrates

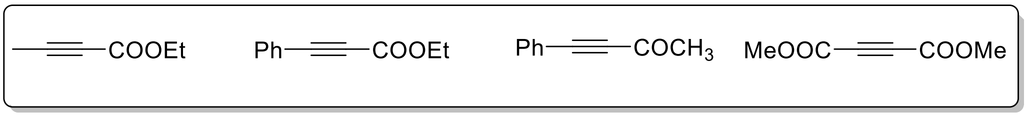

C) Gram-Scale Reaction for Synthesis of 3

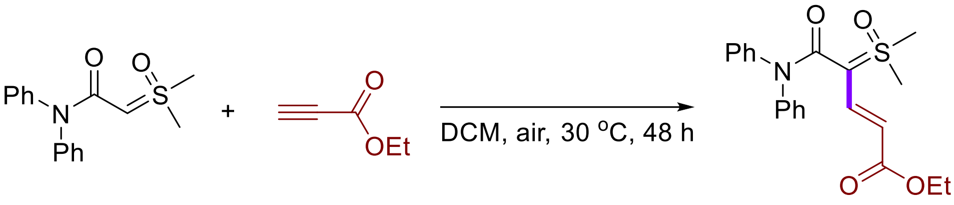

1a, 4.0 mmol **2a**, 3.0 equiv **3**, 1.08 g, 70%

An oven dried Schlenk tube of 100 mL equipped with a magnetic stir bar was charged with amide-sulfoxonium ylide **1a** (4.0 mmol, 1.0 equiv) in DCM (20 mL); then ethyl propiolate **2a** (12.0 mmol, 3.0 equiv) was added. The reaction mixture was sealed and stirred (820 r/min in IKA RCT basic) at 30 ºC for 48 h under air atmosphere. After completion of the reaction, the reaction mixture was cooled to room temperature. The crude product was extracted with ethyl acetate (3 × 15 mL), and the solution was washed with saturated solution of NaCl (15 mL), and the organic layer was dried over anhydrous Na_2SO_4 and concentrated in vacuo. The crude product was purified by flash column chromatography on silica gel to afford the desired product **3** in 70% yield (1.080 g).

D) Gram-Scale Reaction for Synthesis of 73

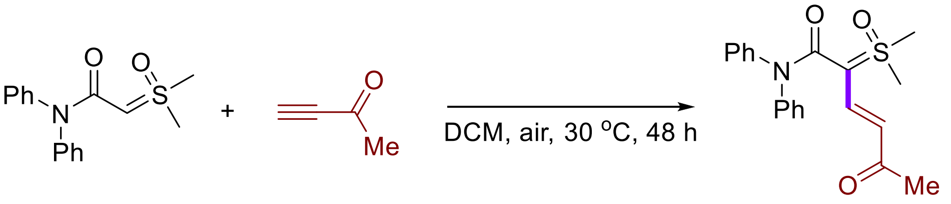

1a, 4.0 mmol 3.0 equiv **73**, 1.22 g, 86%

An oven dried Schlenk tube of 100 mL equipped with a magnetic stir bar was charged with amide-sulfoxonium ylide **1a** (4.0 mmol, 1.0 equiv) in DCM (20 mL); then 3-butyn-2-one (12.0 mmol, 3.0 equiv) was added. The reaction mixture was sealed and stirred (820 r/min in IKA RCT basic) at 30 ºC for 48 h under air atmosphere. After completion of the reaction, the reaction mixture was cooled to room temperature. The crude product was extracted with ethyl acetate (3 × 15 mL), and the solution was washed with saturated solution of NaCl (15 mL), and the organic layer was dried over anhydrous Na_2SO_4 and concentrated in vacuo. The crude product was purified by flash column

chromatography on silica gel to afford the desired product **73** in 86% yield (1.220 g).

E) Synthesis of Bis-alkene Amide-Sulfoxonium Ylide 93

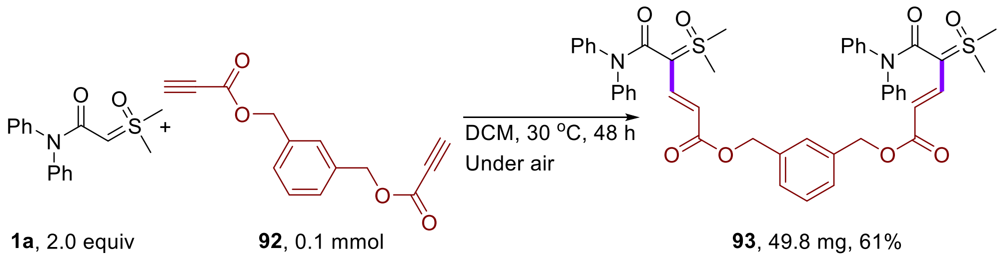

An oven dried glass tube of 4 mL equipped with a magnetic stir bar was charged with amide-sulfoxonium ylide **1a** (0.2 mmol, 2.0 equiv) in DCM (1.0 mL); then 1,3-phenylenebis(methylene) dipropiolate **92** (0.1 mmol, 1.0 equiv) was added. The reaction mixture was stirred (820 r/min in IKA RCT basic) at 30 °C for 48 h under air atmosphere. After completion of the reaction, the reaction mixture was cooled to room temperature. The crude product was extracted with ethyl acetate (3 × 3.0 mL), and the solution was washed with saturated solution of NaCl (3.0 mL), and the organic layer was dried over anhydrous Na_2SO_4 and concentrated in vacuo. The crude product was purified by flash column chromatography on silica gel to afford double alkene substituted product **93** in 61% yield (49.8 mg).

F) Pd-Catalyzed Hydrogenation Reaction for the Synthesis of 1,5-Dicarbonyls 94–96

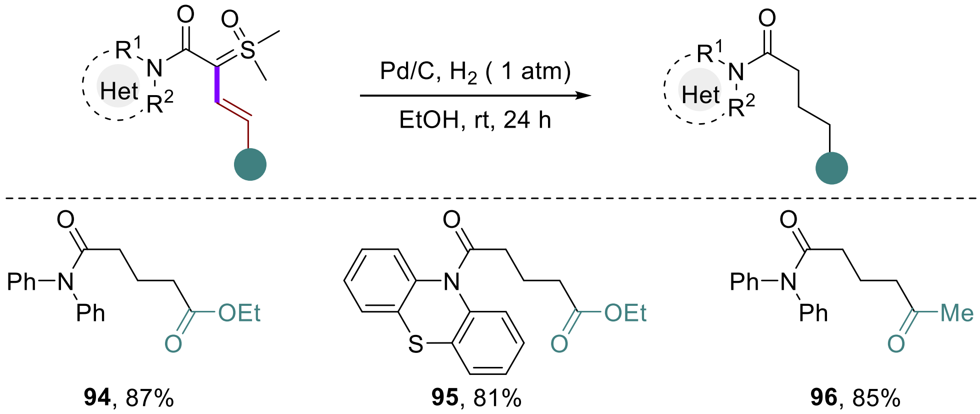

The preparation of this compound was performed according to a literature reference.[7] After three vacuum/H_2 cycles to replace air inside reaction tube with hydrogen, the mixture of the substrate (0.1 mmol), and 50% Pd/C (50 wt % of the substrate), in EtOH (2.0 mL) was vigorously stirred at room temperature under ordinary hydrogen pressure (balloon) for 24 h. After completion of the reaction, the reaction mixture was filtered using a membrane filter (Millipore, Millex®-LH, 0.45 µm), the filtrate was concentrated in vacuo. The crude product was purified by flash column chromatography on silica gel to afford the corresponding products (**94–96**) in 81–87% yields.

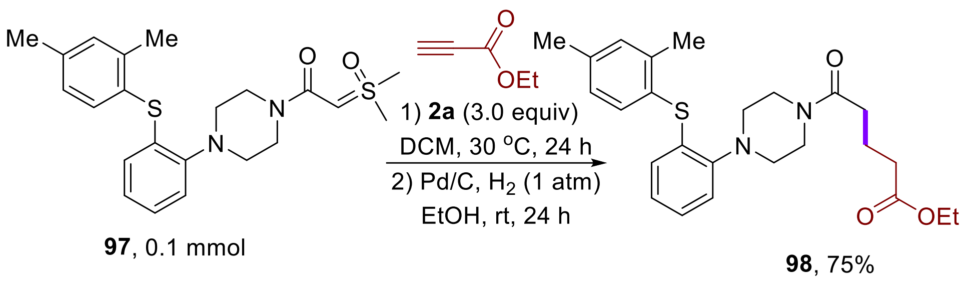

An oven dried glass tube of 4 mL equipped with a magnetic stir bar and was charged with amide-sulfoxonium ylide **97** (0.10 mmol, 1.0 equiv) in DCM (0.5 mL); then ethyl propiolate **2a** (0.30 mmol, 3.0 equiv) was added. The reaction mixture was sealed and stirred (820 r/min in IKA RCT basic) at 30 ºC for 24 h under air atmosphere. After completion, the reaction mixture was recovered to room temperature. The crude product was extracted with ethyl acetate (3 × 3.0 mL), and the solution was washed with saturated solution of NaCl (3.0 mL), and the organic layer was dried over anhydrous Na_2SO_4 and concentrated in vacuo. The mixture of the crude and 50% Pd/C (50 wt % of the substrate), in EtOH (2.0 mL) was vigorously stirred at room temperature under ordinary hydrogen pressure (balloon) for 24 h. After completion of the reaction, the reaction mixture was filtered using a membrane filter (Millipore, Millex®-LH, 0.45 µm), the filtrate was concentrated in vacuo. The crude product was purified by flash column chromatography on silica gel to afford the corresponding product (**98**) in 75% yields (33.0 mg).

G) Synthesis of Pyrazole 100

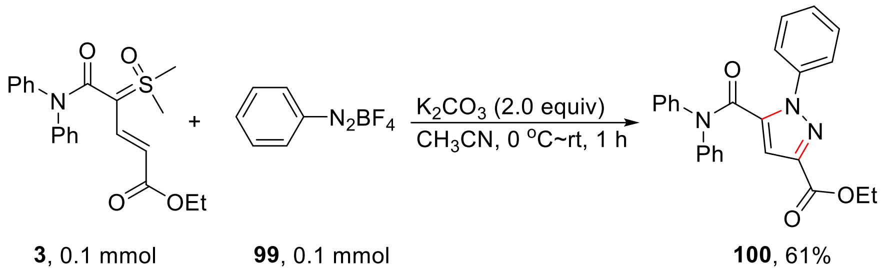

The preparation of this compound was performed according to a literature reference.[8] An oven dried Schlenk tube of 25 mL equipped with a magnetic stir bar was charged with phenyl diazonium tetrafluoroborate **99** (0.1 mmol, 1.0 equiv) in CH_3CN (1.0 mL) at 0 °C, then K_2CO_3 (0.2 mmol, 2.0 equiv), and alkene amide-sulfoxonium ylide **3** (0.1 mmol, 1.0 equiv) was added. The reaction mixture was stirred (820 r/min in IKA RCT basic) at room temperature for 1 h under N_2. After completion of the reaction, the mixture was treated with water and extracted with ethyl acetate (3 × 3.0 mL). The solution was washed with saturated solution of NaCl (3.0 mL), and the organic layer was dried over anhydrous Na_2SO_4 and concentrated in vacuo. The crude product was purified by flash column chromatography on silica gel to afford the corresponding product **100** in 61% yield (25.1 mg).

H) Synthesis of Compound 101

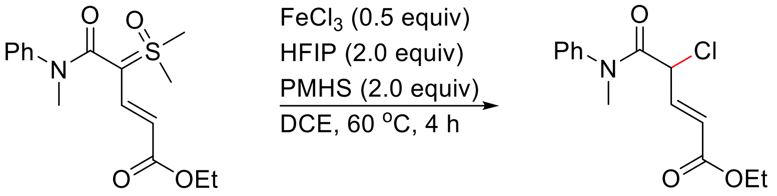

26, 0.1 mmol **101**, 79%

The preparation of this compound was performed according to a literature reference.[9] An oven dried glass tube of 4 mL equipped with a magnetic stir bar and was charged with alkene amide-sulfoxonium ylide **26** (0.1 mmol, 1.0 equiv), and $FeCl_3$ (0.2 mmol, 2.0 equiv) in DCE (1.0 mL); then polymethylhydrosiloxane (0.2 mmol, 2.0 equiv) and 1,1,1,3,3,3-Hexafluoro-2-propanol (0.01 mmol, 10 mol%) was added. The reaction mixture was sealed and stirred (820 r/min in IKA RCT basic) at 60 °C for 4 h. After completion of the reaction, the reaction mixture was extracted with ethyl acetate (3 × 3.0 mL). The solution was washed with saturated solution of NaCl (3.0 mL), and the organic layer was dried over anhydrous Na_2SO_4 and concentrated in vacuo. The crude product was purified by flash column chromatography on silica gel to afford the corresponding product **101** in 79% yield (22.2 mg).

J) Synthesis of Compound 102

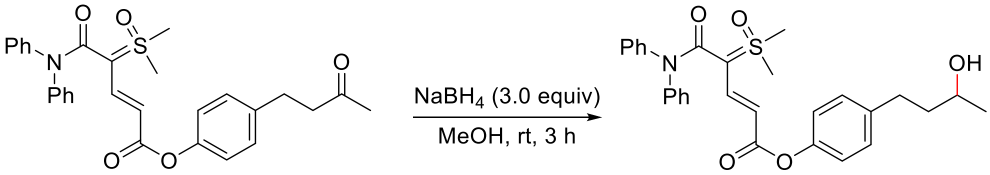

71, 0.1 mmol **102**, 92%

The preparation of this compound was performed according to a literature reference.[10] An oven dried glass tube of 4 mL equipped with a magnetic stir bar and was charged with alkene amide-sulfoxonium ylide **71** (0.1 mmol, 1.0 equiv), and $NaBH_4$ (0.3 mmol, 3.0 equiv) in MeOH (1.0 mL). The reaction mixture was sealed and stirred (820 r/min in IKA RCT basic) at room temperature for 3 h. After completion of the reaction, the mixture was treated with NH_4Cl and extracted with ethyl acetate (3 × 3.0 mL). The solution was washed with saturated solution of NaCl (3.0 mL), and the organic layer was dried over anhydrous Na_2SO_4 and concentrated in vacuo. The crude product was purified by flash column chromatography on silica gel to afford the corresponding product **102** in 92% yield (46.5 mg).

K) Synthesis of Compound 103

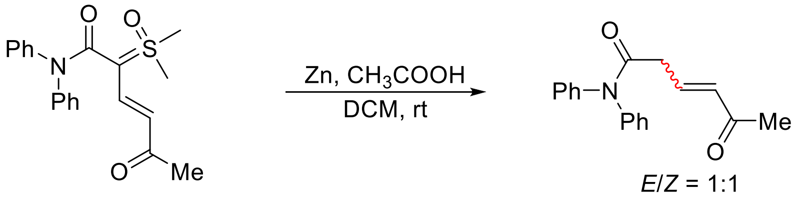

73, 0.1 mmol **103**, 25.4 mg, 91%

The preparation of this compound was performed according to a literature reference.[11] An oven dried glass tube of 4 mL equipped with a magnetic stir bar was charged with alkene amide-sulfoxonium ylide **73** (0.1 mmol), and Zn dust (2.0 mmol) in DCM (2.0 mL); then CH_3COOH (2.0 mmol) was added. The reaction mixture was sealed and stirred (820 r/min in IKA RCT basic) at room temperature for 24 h. After completion of the reaction, the reaction mixture was filtered. The filtrate was washed with saturated $NaHCO_3$ solution and concentrated in vacuo. The crude product was purified by flash column chromatography on silica gel to afford the corresponding product **103** in 91% yield (25.4 mg).

L) Synthesis of Compound 104

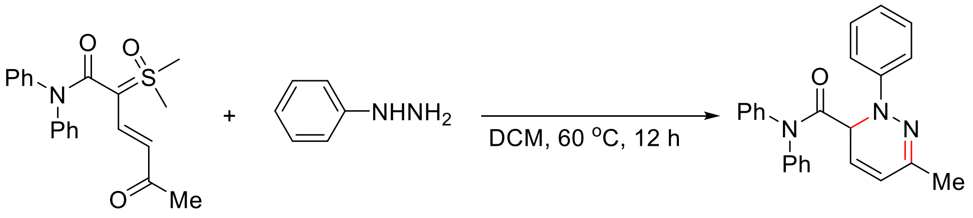

73, 0.1 mmol 0.1 mmol **104**, 41%

An oven dried glass tube of 4 mL equipped with a magnetic stir bar was charged with alkene amide-sulfoxonium ylide **73** (0.1 mmol, 1.0 equiv) in DCM (1.0 mL); then phenylhydrazine (0.2 mmol, 2.0 equiv) was added. The reaction mixture was sealed and stirred (820 r/min in IKA RCT basic) at 60 °C for 12 h. After completion of the reaction, the reaction mixture was extracted with ethyl acetate (3 × 3.0 mL). The solution was washed with saturated solution of NaCl (3.0 mL), and the organic layer was dried over anhydrous Na_2SO_4 and concentrated in vacuo. The crude product was purified by flash column chromatography on silica gel to afford the corresponding product **104** in 41% yield (15.0 mg).

M) Synthesis of Compound 105

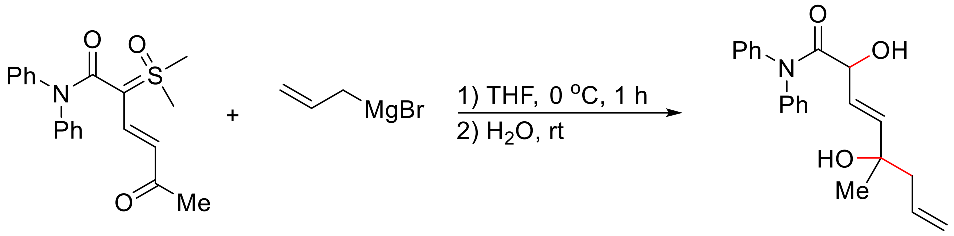

73, 0.1 mmol 0.2 mmol **105**, 66%, 1.5:1

The preparation of this compound was performed according to a literature reference.[12] An oven dried Schlenk tube of 25 mL equipped with a magnetic stir bar was charged with alkene amide-sulfoxonium ylide **73** (0.1 mmol, 1.0 equiv) in THF (1.0 mL) at 0 °C; then allylMgBr (1.0 M in diethyl ether, 0.2 mmol, 2.0 equiv) was added. The reaction mixture was sealed and stirred (820 r/min in IKA RCT basic) at 0 °C for 1 h. After completion of the reaction, the reaction mixture was extracted with ethyl acetate (3 × 3.0 mL). The solution was washed with saturated solution of NH_4Cl (3.0 mL), and the organic layer was dried over anhydrous Na_2SO_4 and concentrated in vacuo. The crude product was purified by flash column chromatography on silica gel to afford the corresponding product **105** in 66% yield (22.2 mg).

N) Synthesis of Polyfunctional Thiabenzene 1-oxide Compounds 106–110

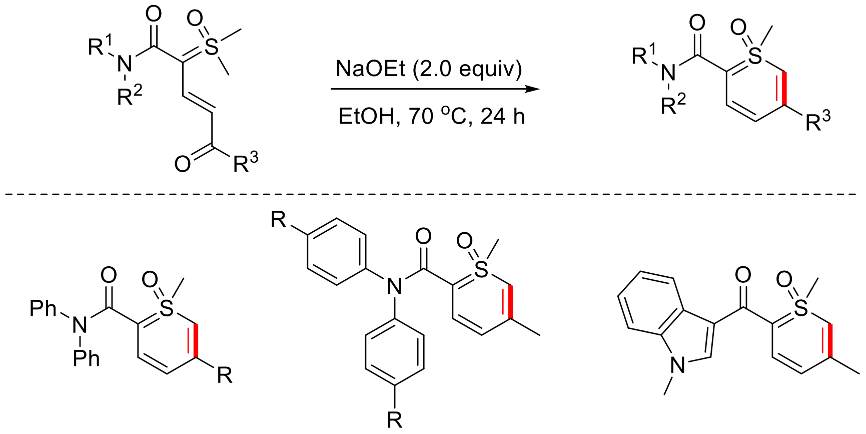

106, R = Me, 79%
107, R = Ph, 72%
108, R = Me, 74%
109, R = Br, 46%
110, 71%

The preparation of this compound was performed according to a literature reference.[13] An oven dried Schlenk tube of 25 mL equipped with a magnetic stir bar was charged with alkene amide-sulfoxonium ylide (0.1 mmol), and EtONa (0.2 mmol, 2.0 equiv) in EtOH (2.0 mL). The reaction mixture was sealed and stirred (820 r/min in IKA RCT basic) at 70 °C for 24 h. After completion of the reaction, the reaction mixture was cooled to room temperature. The crude product was extracted with ethyl acetate (3 × 3.0 mL), and the solution was washed with saturated solution of NaCl (3.0 mL), and the organic layer was dried over anhydrous Na_2SO_4 and concentrated in vacuo. The crude product

was purified by flash column chromatography on silica gel to afford the corresponding products (**106–110**) in 46–79% yields.

4. X-ray Crystallographic Data of 44

The preparation of crystal **44**: 12.0 mg of **44** was dissolved in dichloromethane (1.0 mL), it was filtered through a plug of silica gel. The filtrate was transferred to a tube (diameter, 0.6 cm), and 2.0 mL of petroleum ether was slowly added. Light yellow crystals were grown from standing it for a week.

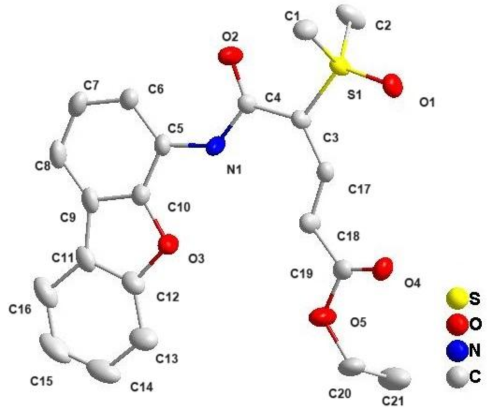

Figure S1. Molecular structure of compound **44** (CCDC number: 2355877). Thermal ellipsoids are drawn at 50% probability. H atoms are omitted for clarity.

Table S4. Summary of X-ray crystallographic data for compound 44

formula	$C_{21}H_{21}NO_5S$
Fw.	399.46
T/K	273
crystal system	orthorhombic
space group	P212121
a/Å	5.4123(8)
b/Å	11.280(2)
c/Å	32.226(7)
α/deg	90.00
β/deg	90.00
γ/deg	90.00
V/Å^3	1967.4(6)
Z	4
D/g cm^{-3}	1.349
cryst size/mm	0.14×0.13×0.12
reflns collected	11348
ind reflns, *R*int	4846, 0.0308
goodness-of-fit on F^2	1.046
$R1$, $wR2$ [$I > 2\sigma(I)$]	0.0349, 0.0877
$R1$, $wR2$ (all data)	0.0385, 0.0913

5. X-ray Crystallographic Data of 73

The preparation of crystal **73**: 11.0 mg of **73** was dissolved in dichloromethane (1.0 mL), it was filtered through a plug of silica gel. The filtrate was transferred to a tube (diameter, 0.6 cm), and 2.0 mL of petroleum ether was slowly added. Light yellow crystals were grown from standing it for a week.

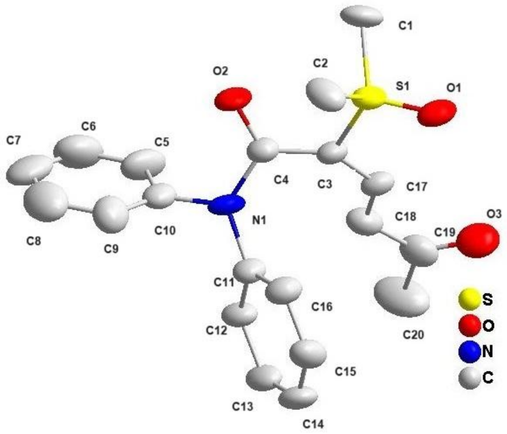

Figure S2. Molecular structure of compound **73** (CCDC number: 2355876). Thermal ellipsoids are drawn at 50% probability. H atoms are omitted for clarity.

Table S5. Summary of X-ray crystallographic data for compound 73

formula	$C_{20}H_{21}NO_3S$
Fw.	355.45
T/K	273
crystal system	monoclinic
space group	I1a1
a/Å	9.839(4)
b/Å	14.413(4)
c/Å	13.903(4)
α/deg	90.00
β/deg	107.91(2)
γ/deg	90.00
V/$Å^3$	1876.0(11)
Z	4
D/g cm^{-3}	1.258
cryst size/mm	0.14×0.13×0.12
reflns collected	8057
ind reflns, *R*int	4352, 0.1321
goodness-of-fit on F^2	1.021
*R*1, *wR*2 [$I > 2\sigma(I)$]	0.0952, 0.2414
*R*1, *wR*2 (all data)	0.1830, 0.3065

6. Control Experiments

A) Competition Experiment between Ethyl Propiolate and 3-Butyn-2-one

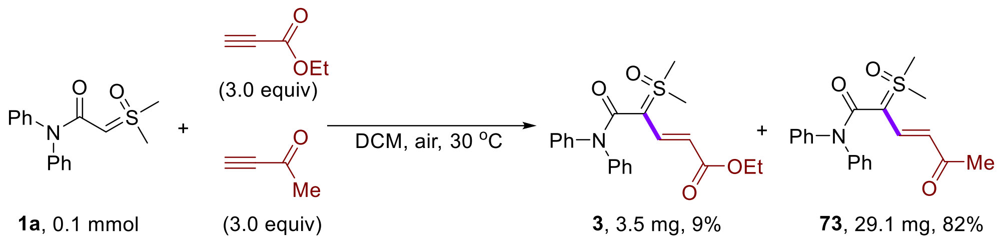

An oven dried glass tube of 4 mL, equipped with a magnetic stir bar was charged with amide-sulfoxonium ylide **1a** (0.1 mmol) in DCM (1.0 mL); then ethyl propiolate **2a** (0.3 mmol, 3.0 equiv) and 3-butyn-2-one (0.3 mmol, 3.0 equiv) were added. The reaction mixture was stirred (820 r/min in IKA RCT basic) at 30 °C for 24 h under air atmosphere. After completion, the reaction mixture was returned to room temperature. The crude product was extracted with ethyl acetate (3 × 3.0 mL), and the solution was washed with saturated solution of NH_4Cl (3.0 mL), and the organic layer was dried over anhydrous Na_2SO_4 and concentrated in vacuo. The crude product was purified by flash column chromatography on silica gel to afford the desired products **3** and **73** in 9% and 82% yields, respectively.

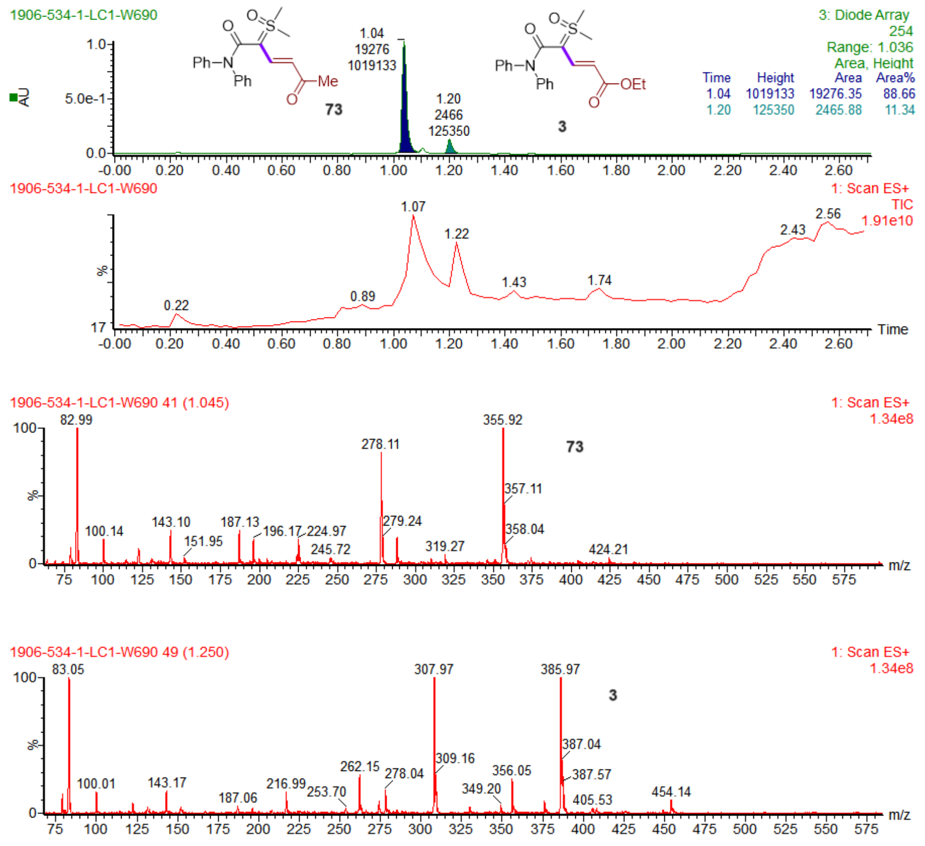

Figure S3. The UPLC-MS chart of the crude reaction mixture of A.

B) Deuterium Labeling Experiments

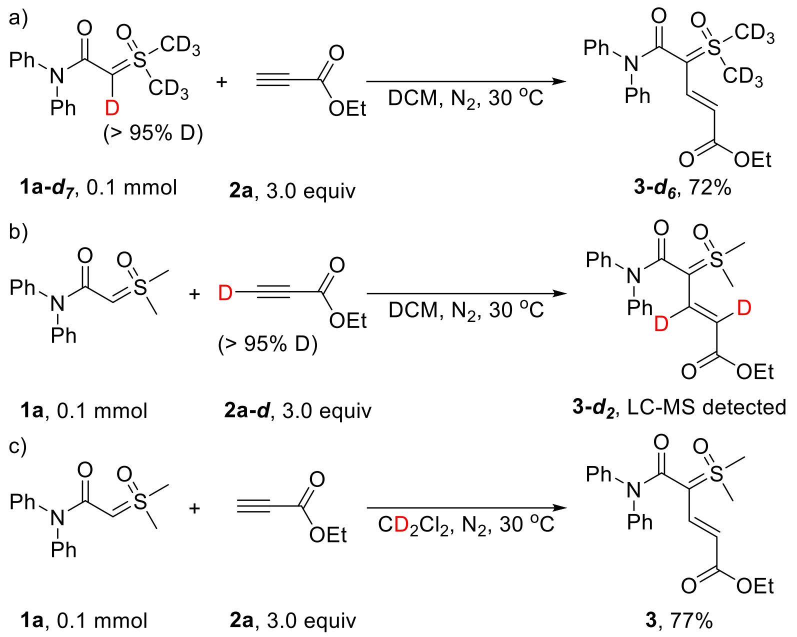

a) A Schlenk tube of 25 mL equipped with a magnetic stir bar, and was charged with amide-sulfoxonium ylide **1a-*d*₇** (0.10 mmol, 1.0 equiv) in DCM (0.5 mL); then ethyl propiolate **2a** (0.30 mmol, 3.0 equiv) was added. The reaction mixture was sealed and stirred (820 r/min in IKA RCT basic) at 30 ºC for 24 h under N_2 atmosphere. After completion, the reaction mixture was recovered to room temperature. The crude product was extracted with ethyl acetate (3 × 3.0 mL), and the solution was washed with saturated solution of NaCl (3.0 mL), and the organic layer was dried over anhydrous Na_2SO_4 and concentrated in vacuo. The crude product was purified by flash column chromatography on silica gel (eluent: petroleum ether/ethyl acetate) to give the corresponding product **3-d_6** in 72% yield (28.2 mg).

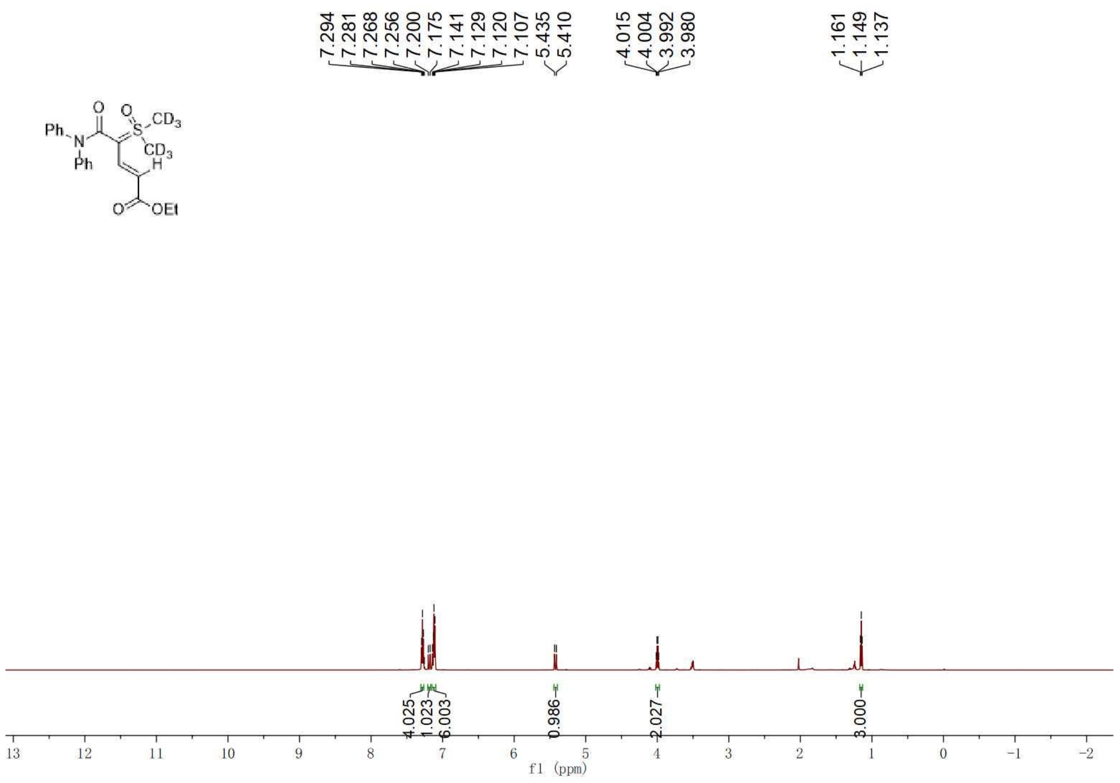

Figure S4. ^{1}H NMR (600 MHz, $CDCl_3$) Spectrum of 3-*d*$_6$.

b) A Schlenk tube of 25 mL equipped with a magnetic stir bar, and was charged with amide-sulfoxonium ylide **1a** (0.10 mmol, 1.0 equiv) in DCM (0.5 mL); then ethyl propiolate **2a-*d*** (0.30 mmol, 3.0 equiv) was added. The reaction mixture was sealed and stirred (820 r/min in IKA RCT basic) at 30 ºC for 24 h under N_2 atmosphere. After completion, the reaction mixture was recovered to room temperature. The reaction was monitored by UPLC-MS, and the results are summarized in **Figure S5**. Notably, the desired product **3-*d*$_2$** could be detected by LC-MS.

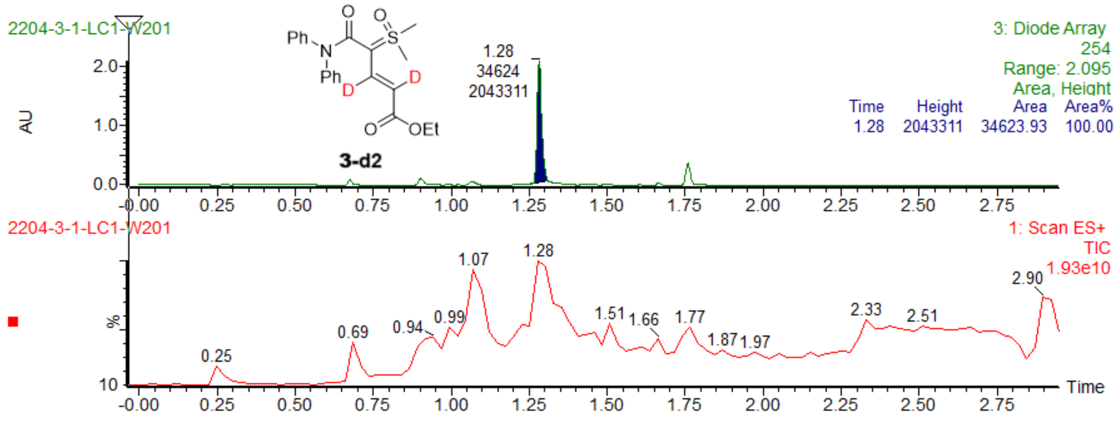

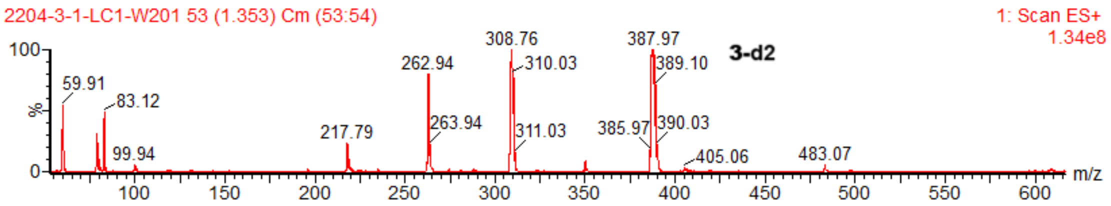

Figure S5. The UPLC-MS chart of the crude reaction mixture of b.

c) A Schlenk tube of 25 mL equipped with a magnetic stir bar, and was charged with amide-sulfoxonium ylide **1a** (0.10 mmol, 1.0 equiv) in CD_2Cl_2 (0.5 mL); then ethyl propiolate **2a** (0.30 mmol, 3.0 equiv) was added. The reaction mixture was sealed and stirred (820 r/min in IKA RCT basic) at 30 ºC for 24 h under N_2 atmosphere. After completion, the reaction mixture was recovered to room temperature. The crude product was extracted with ethyl acetate (3 × 3.0 mL), and the solution was washed with saturated solution of NaCl (3.0 mL), and the organic layer was dried over anhydrous Na_2SO_4 and concentrated in vacuo. The crude product was purified by flash column chromatography on silica gel (eluent: petroleum ether/ethyl acetate) to give the corresponding product **3** in 77% yield (29.7 mg).

7. Computational Details

In our calculations, Gaussian 16 program[14] was used to carry out total density functional theory (DFT) calculations. Geometry optimizations were performed by wB97XD functional[15] with 6-31+G**[16-20] basis set for all elements. The vibrational frequencies calculations were conducted at the same level of theory to be sure whether every optimized stationary point is an energy minimum or a transition state and evaluate the zero-point vibrational energy and thermal corrections at 298 K. The single point energies and solvent effects based on the gas-phase optimized structures were calculated by the PWPB95 functional[21] with dispersion correction of D3(BJ),[22] and def2-TZVP basis set[23,24] was used for all elements by using ORCA 5.0.1 packages.[25–28] The solvation energies were evaluated by self-consistent reaction field by SMD implicit solvent model.[29] The conformations of intermediates and transition states were generated by Molclus 1.10[30] and initially optimized and screened by CREST (version 2.10.2),[31] and then fully optimized by Gaussian 16. The calculated structures were displayed with the CYLview software.[32] In order to adjust the Gibbs free energies from 1 atm to 1 mol/L, a correction of RTln(Csol/Cgas) (1.89 kcal/mol) is added to energies of all species. Csol represents the standard molar concentration in solution (1 mol/L), Cgas represents the standard molar concentration in gas phase (0.0446 mol/L), and R represents the gas constant.

Table S6. Zero-point correction (ZPE), thermal correction to enthalpy (ΔH), thermal correction to Gibbs free energy (ΔG), single point energy (SPE), energies (E), Gibbs free energies (G), and enthalpies (H) (in Hartree) of the structures calculated at the PWPB95-D3(BJ)/def2-TZVP:SMD(DCM) // wB97XD/6-31+G:IEFPCM(DCM) level of theory**

	ZPE	ΔH	ΔG	SPE	E	H	G
**1a**	-1222.978655	0.318353	0.249517	-1222.978655	-2445.957311	-1222.660302	-1222.729138
**2b**	-305.1341864	0.079325	0.042151	-305.1341864	-610.2683728	-305.0548614	-305.0920354
**TS0-A**	-1528.084438	0.39889	0.313807	-1528.084438	-3056.168877	-1527.685548	-1527.770631
**TS0-B**	**-1528.095102**	**0.398942**	**0.313854**	**-1528.095102**	**-3056.190204**	**-1527.69616**	**-1527.781248**
**TS0-C**	-1528.082998	0.398759	0.316281	-1528.082998	-3056.165996	-1527.684239	-1527.766717
**TS0-D**	-1528.08363	0.39909	0.314729	-1528.08363	-3056.167259	-1527.68454	-1527.768901
**INT1-A**	-1528.119777	0.401598	0.319929	-1528.119777	-3056.239555	-1527.718179	-1527.799848
**INT1-B**	-1528.116001	0.401658	0.318592	-1528.116001	-3056.232001	-1527.714343	-1527.797409
**TS1-E-A**	-1833.244273	0.478585	0.373811	-1833.244273	-3666.488547	-1832.765688	-1832.870462
**TS1-E-B**	**-1833.258271**	**0.478589**	**0.374612**	**-1833.258271**	**-3666.516542**	**-1832.779682**	**-1832.883659**
**TS1-Z**	-1833.250884	0.478972	0.371667	-1833.250884	-3666.501769	-1832.771912	-1832.879217
**INT2-E**	-1833.288314	0.484475	0.378526	-1833.288314	-3666.576627	-1832.803839	-1832.909788
**INT2-Z**	-1833.289093	0.485146	0.383092	-1833.289093	-3666.578187	-1832.803947	-1832.906001
**TS2-E**	-1833.28385	0.479791	0.375185	-1833.28385	-3666.567701	-1832.804059	-1832.908665
**TS2-Z**	-1833.278674	0.479549	0.376726	-1833.278674	-3666.557348	-1832.799125	-1832.901948
**Prod-E**	-1528.190987	0.402672	0.318998	-1528.190987	-3056.381975	-1527.788315	-1527.871989
**Prod-Z**	-1528.179768	0.402386	0.320492	-1528.179768	-3056.359536	-1527.777382	-1527.859276

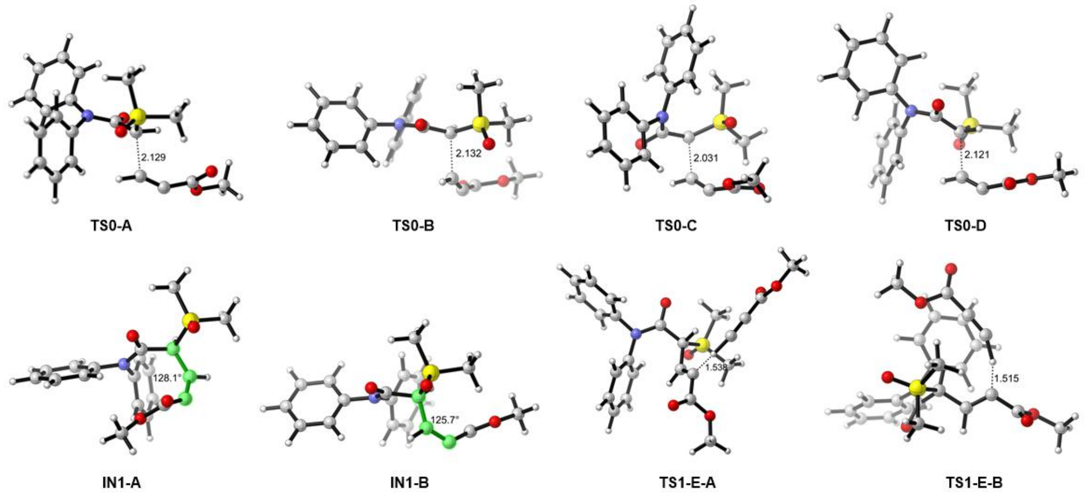

Figure S6. Conformational search for **TS0**, **INT1** and **TS1-E**.

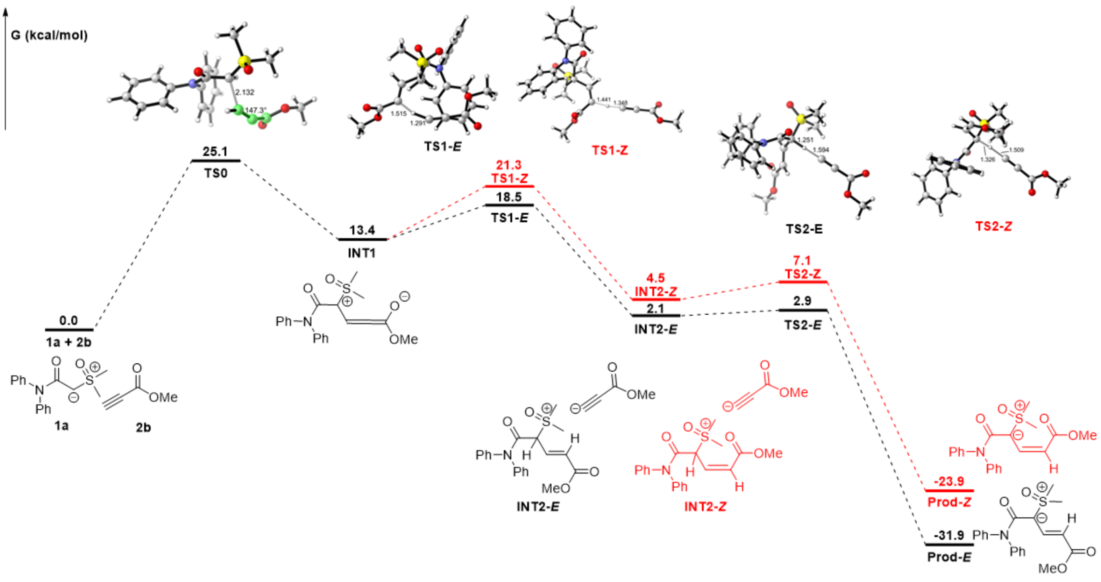

Figure S7. Free energy changes of the possible reaction pathway.

Scheme S1. Proposed Reaction Mechanism

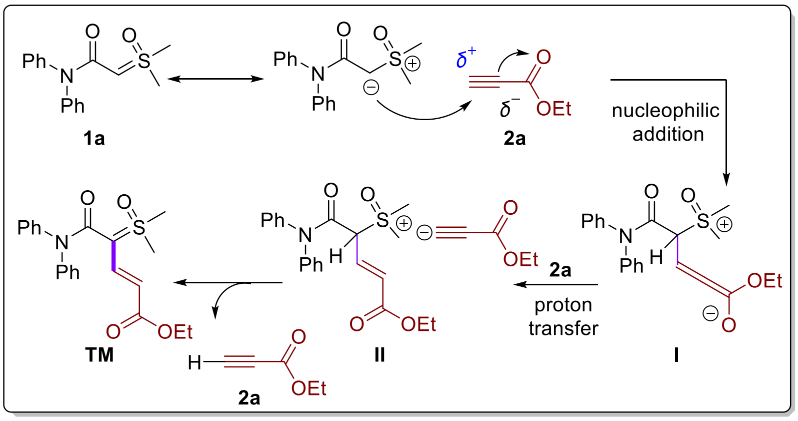

Cartesian coordinates of the optimized structure

INT1-A

C	4.12799400	0.76295700	2.35774000
C	3.30927000	1.67799000	1.69725700
C	2.09468100	1.27602300	1.15037600
C	1.68994200	-0.05377700	1.27035600
C	2.50378300	-0.97466100	1.93161100
C	3.72048300	-0.56433300	2.47091500
N	0.46048100	-0.50755500	0.68410900
C	-0.66842100	0.24795500	0.75291600
C	0.48081800	-1.79253400	0.03316000
C	1.11872100	-1.92033500	-1.19789800
C	1.16873400	-3.16790300	-1.81085500
C	0.58055100	-4.27644000	-1.19968000
C	-0.05228800	-4.14018900	0.03421700
C	-0.09424600	-2.89519500	0.66005900
C	-1.87524300	-0.27308200	-0.02993900
O	-0.76408500	1.28491700	1.40196200
S	-3.34145200	0.72067200	0.51299700
O	-3.38199800	2.12479000	0.09882200
C	-4.69524800	-0.18691100	-0.20658700
C	-3.55506200	0.51528900	2.27325700
C	-1.80940000	-0.24599200	-1.55024900
C	-1.12232800	0.58994000	-2.32824900
C	-0.25912500	1.68216400	-2.06713500
O	1.05799400	1.29727300	-1.96868900
C	2.03066200	2.32919600	-2.09670900
O	-0.56173800	2.88128200	-2.07333100
H	5.07640200	1.08155900	2.77786800
H	3.61812600	2.71363300	1.59704900
H	1.46457800	1.98822000	0.63582000
H	2.19537200	-2.01105300	2.01675500
H	4.34808200	-1.28803500	2.98087800
H	1.53780300	-1.03601700	-1.66648300
H	1.65723200	-3.27313800	-2.77394900
H	0.61793300	-5.24636500	-1.68501100
H	-0.50182300	-5.00150900	0.51725300
H	-0.55991300	-2.77938100	1.63472700
H	-2.15037600	-1.27616200	0.31410000
H	-4.64036300	-1.23485000	0.09130200
H	-5.61347200	0.27888200	0.15240700
H	-4.60734500	-0.07917300	-1.28791900
H	-3.50886900	-0.54815600	2.51340400

H	-4.53213900	0.93182800	2.52198700
H	-2.75073500	1.06908900	2.75391200
H	-2.41689500	-1.04063900	-1.98674100
H	2.01786200	2.74949400	-3.10700500
H	1.86195700	3.13329200	-1.37651100
H	2.99218700	1.85315000	-1.90270000

INT1-B

C	4.24807100	-1.69501100	-1.37579200
C	3.34477400	-2.20024300	-0.44067900
C	2.16761700	-1.51239000	-0.16081600
C	1.89442900	-0.32169300	-0.83057700
C	2.78959300	0.19052600	-1.76592300
C	3.96999000	-0.49879700	-2.03491700
N	0.69759500	0.41431100	-0.52144400
C	-0.51234200	-0.17022000	-0.72185400
C	0.86791700	1.75175000	-0.02126900
C	1.42359200	1.94037400	1.24128800
C	1.64311000	3.23381900	1.70423800
C	1.30092700	4.32965900	0.91303900
C	0.74493500	4.13332400	-0.34962200
C	0.53727700	2.84080700	-0.82571100
C	-1.72491900	0.52004600	-0.10352300
O	-0.65822100	-1.24953900	-1.28946400
S	-3.19087300	0.06850600	-1.13577700
O	-3.76028900	-1.25500100	-0.86279100
C	-4.35630200	1.36080200	-0.78006200
C	-2.78093600	0.29534800	-2.85768100
C	-1.96210400	0.02836500	1.32076400
C	-2.38090900	0.77800000	2.34448700
C	-2.68952100	2.16812800	2.33267600
O	-4.03577800	2.40100500	2.17055800
C	-4.46209400	3.75555300	2.29771300
O	-1.91887700	3.10535000	2.55667000
H	5.16767000	-2.23092700	-1.58708700
H	3.55937200	-3.12824500	0.07906400
H	1.46224400	-1.89284900	0.57043100
H	2.56686200	1.12436700	-2.27228400
H	4.67023200	-0.10019900	-2.76168900
H	1.67548500	1.07857700	1.85045600
H	2.06903700	3.38570700	2.69017000
H	1.46658800	5.33668000	1.28136400
H	0.48377300	4.98389700	-0.97060800
H	0.13280100	2.67242500	-1.81954500
H	-1.69257400	1.61105600	-0.14866700

H	-3.95016900	2.31265400	-1.12549400
H	-5.27413200	1.10600800	-1.31108100
H	-4.51066000	1.37988100	0.30209700
H	-2.32764200	1.28068600	-2.98379200
H	-3.71352800	0.21937300	-3.41897600
H	-2.08875500	-0.50075600	-3.12755800
H	-1.73499300	-1.03202700	1.43579800
H	-4.22677500	4.14649100	3.29047600
H	-3.98966500	4.39012600	1.54316000
H	-5.54183700	3.73915500	2.14952200
**INT2-E**			
C	-4.92858900	-3.45867900	-1.74675200
C	-3.90020300	-2.96067700	-2.54680600
C	-2.94341000	-2.10687700	-2.00591800
C	-3.01603900	-1.76425300	-0.65780900
C	-4.03776600	-2.25605100	0.14945600
C	-4.99684200	-3.10362900	-0.40069300
N	-2.04316700	-0.85657900	-0.10448800
C	-0.74708100	-1.26390300	-0.04327100
C	-2.55462400	0.32890400	0.52165700
C	-2.50483400	0.45639000	1.90734800
C	-3.00615400	1.61161600	2.50564600
C	-3.57568800	2.61261900	1.72261900
C	-3.64633700	2.46182600	0.33638700
C	-3.13644700	1.31900800	-0.26837800
C	0.38054600	-0.23388300	0.19868100
O	-0.39648100	-2.41560800	-0.26712500
S	1.29337100	-0.72706300	1.69847700
O	0.45115100	-0.76833400	2.90217900
C	2.60544700	0.46380100	1.80547800
C	2.10098100	-2.27119300	1.36029900
C	0.13006100	1.24444000	0.23373500
C	0.45047800	2.00282300	-0.81504700
C	0.18525900	3.46384500	-0.78116600
O	0.44138200	4.02238200	-1.96478700
O	-0.21042100	4.07963700	0.18943800
C	0.22583100	5.43733400	-2.06297200
H	-5.67604600	-4.12003400	-2.17265800
H	-3.84533200	-3.23104100	-3.59616000
H	-2.14221300	-1.70943600	-2.62029800
H	-4.08436000	-1.97440100	1.19647600
H	-5.79511200	-3.48782500	0.22576700
H	-2.05788600	-0.33103600	2.50567400
H	-2.95482400	1.72287400	3.58355100

H	-3.96550600	3.51090400	2.18975100
H	-4.08846700	3.24187300	-0.27434900
H	-3.16858700	1.19441200	-1.34595400
H	3.27661500	0.12345400	2.59432200
H	2.15936700	1.42747800	2.05097800
H	3.11176500	0.47964200	0.83601200
H	2.66498200	-2.14622200	0.43199000
H	2.75957600	-2.45806600	2.20943700
H	1.32336600	-3.02628000	1.25959900
H	-0.30868600	1.70357500	1.11550700
H	-0.81938100	5.67433400	-1.85618900
H	0.86984900	5.96595100	-1.35791500
H	0.48295900	5.69943100	-3.08690700
H	1.11574400	-0.44317800	-0.61357100
C	3.97624600	-0.74132400	-1.23363000
C	5.18816900	-1.00539400	-0.51911700
O	6.27758800	-1.01264000	-1.30422600
O	5.24741800	-1.20594400	0.68906600
C	7.52640500	-1.27089700	-0.65213900
C	2.86573300	-0.52739700	-1.74828100
H	7.72994100	-0.50736300	0.10155800
H	7.51561700	-2.25571300	-0.18061000
H	0.89614200	1.58483800	-1.71267600
H	8.27676100	-1.23572100	-1.43987300

INT2-Z

C	4.79588700	2.37563300	-1.18377600
C	4.50991500	1.39727000	-0.23181500
C	3.21372100	0.90695200	-0.10214700
C	2.21354700	1.39596200	-0.93690100
C	2.48562100	2.37312200	-1.88928400
C	3.78386800	2.86417500	-2.00933100
N	0.86561600	0.91212400	-0.78095700
C	0.57654400	-0.35221300	-1.16902000
C	-0.10678200	1.83131600	-0.25229500
C	-0.02460400	2.21222700	1.08550500
C	-0.97401000	3.08525100	1.60230400
C	-1.99543900	3.57526600	0.78643200
C	-2.05884700	3.20340100	-0.55434400
C	-1.10475400	2.33497000	-1.08217600
C	-0.83119200	-0.86865700	-0.81282900
O	1.36117900	-1.07848400	-1.76845100
S	-0.94332400	-2.60768200	-1.39912400
O	-0.03236900	-3.54881900	-0.74660400
C	-2.63992100	-3.04123800	-1.08877700

C	-0.78893600	-2.57426900	-3.17528100
C	-1.13253100	-0.83670400	0.66044000
C	-2.26443200	-0.39120500	1.21126400
C	-3.42613900	0.14235200	0.45883900
O	-4.23199100	0.83090800	1.25726500
C	-5.35948900	1.46714200	0.63867400
O	-3.63249200	-0.01522400	-0.73555800
H	5.80615200	2.76008700	-1.27951500
H	5.29349100	1.01988000	0.41690600
H	2.96521200	0.15263700	0.63599900
H	1.68854700	2.74651800	-2.52447200
H	4.00355200	3.62729900	-2.74873200
H	0.74267600	1.78519100	1.72341800
H	-0.92901700	3.36552700	2.64903400
H	-2.74268100	4.24580800	1.19816000
H	-2.84626300	3.58854900	-1.19383800
H	-1.13706800	2.04670400	-2.12895600
H	-1.61312500	-0.35603700	-1.38346900
H	-3.29727900	-2.27109900	-1.49464500
H	-2.79946900	-4.01213600	-1.55896200
H	-2.75112500	-3.11560600	-0.00655700
H	-1.41791300	-1.77429800	-3.57003100
H	-1.12070500	-3.54954400	-3.53401600
H	0.26259000	-2.39820800	-3.39189400
H	-0.32378300	-1.14105700	1.31991500
H	-5.01509200	2.19279600	-0.10100100
H	-5.99821200	0.72326200	0.15972300
H	-5.88867200	1.96618500	1.44710500
C	1.10646400	-0.85249900	3.37885900
C	2.14356500	-1.62831500	2.77869400
O	1.90904000	-1.85699600	1.46132800
O	3.14458500	-2.06181800	3.33236300
C	2.86368200	-2.67167800	0.77255400
C	0.19050300	-0.19484300	3.90542800
H	3.85593800	-2.21553600	0.81001900
H	2.90536400	-3.66778500	1.21855500
H	-2.31423700	-0.33444100	2.29411200
H	2.50392900	-2.72370000	-0.25301300

Prod-E

C	5.14979400	-1.10351600	-0.96528900
C	4.17245100	-0.86871600	-1.93242000
C	2.88583700	-0.49756600	-1.55260000
C	2.57969300	-0.34354100	-0.20094200
C	3.55318400	-0.57346000	0.76972900

C	4.83546300	-0.95813300	0.38514000
N	1.24947800	0.01981200	0.18578900
C	0.75553800	1.24843200	-0.22931100
C	0.61465300	-0.76807900	1.19449000
C	0.09255700	-0.16848500	2.34091500
C	-0.52487500	-0.95341900	3.31144400
C	-0.59693500	-2.33727900	3.15832400
C	-0.04646700	-2.93513300	2.02517900
C	0.55263100	-2.15398800	1.04205300
C	-0.69847300	1.43194500	-0.19642000
O	1.52124000	2.14211400	-0.60717900
S	-1.12709000	3.04506700	0.16618600
O	-0.54650800	3.58101500	1.41537700
C	-2.90831300	3.14244800	0.22438300
C	-0.71607100	4.16639500	-1.16903300
C	-1.69466700	0.48358200	-0.55802400
C	-1.51522600	-0.75058900	-1.09697100
C	-2.67157700	-1.59153900	-1.35723700
O	-2.30828300	-2.77855400	-1.88969600
O	-3.84534000	-1.31038000	-1.14206000
C	-3.36533000	-3.69362000	-2.18270100
H	6.15022600	-1.40066900	-1.26289000
H	4.40805500	-0.98549600	-2.98542500
H	2.11699700	-0.32394300	-2.29882300
H	3.30330300	-0.45546800	1.81940600
H	5.59083500	-1.13807200	1.14344400
H	0.16546100	0.90648200	2.46900000
H	-0.93478800	-0.48070800	4.19836700
H	-1.07180300	-2.94645200	3.92063400
H	-0.09333600	-4.01196700	1.89816900
H	0.97153200	-2.61503300	0.15312600
H	-3.12137000	4.17783800	0.49337200
H	-3.26303200	2.47276200	1.00779600
H	-3.34393900	2.90193000	-0.74473000
H	-1.19492500	3.80653200	-2.08036100
H	-1.07129100	5.15931700	-0.88935000
H	0.36832000	4.13443300	-1.26196300
H	-2.73534500	0.75405100	-0.39966100
H	-3.92184800	-3.94201900	-1.27627100
H	-4.04871100	-3.27004900	-2.92221500
H	-2.88053000	-4.58254000	-2.58345300
H	-0.53997800	-1.15225000	-1.33840000

Prod-Z

C	-2.29235100	1.74012100	4.88034300

C	-3.26430000	1.39564500	3.94005000
C	-2.90480000	1.12586600	2.62373700
C	-1.56260200	1.17892500	2.24010700
C	-0.58785800	1.51783100	3.17962200
C	-0.95550500	1.80376200	4.49307000
N	-1.20703500	0.91416100	0.88351900
C	-1.59215300	-0.32343100	0.32560800
C	-0.15742200	1.69490800	0.30905800
C	-0.30705500	3.07938900	0.20864100
C	0.70249500	3.84823100	-0.36064700
C	1.86241500	3.23997600	-0.84284200
C	2.01404200	1.85948400	-0.72905600
C	1.01584100	1.08847300	-0.13911100
C	-1.65193100	-0.38463300	-1.10974400
O	-1.84553500	-1.28712900	1.06380500
S	-2.03899500	-1.94520900	-1.67850500
O	-3.33116800	-2.52394500	-1.24213800
C	-2.04013000	-1.92409200	-3.45985100
C	-0.71911300	-3.07722900	-1.26049300
C	-1.67135300	0.80668900	-1.95301700
C	-0.91479800	1.13596100	-3.02099200
C	0.29956100	0.43482200	-3.45275000
O	1.01091800	1.20225300	-4.29381900
C	2.27232600	0.67789600	-4.71972900
O	0.68533000	-0.66883300	-3.09440500
H	-2.57618000	1.95896400	5.90471300
H	-4.30946100	1.34744700	4.22935500
H	-3.66015300	0.86749000	1.88872100
H	0.45496100	1.56197400	2.88220500
H	-0.19012500	2.06893500	5.21581400
H	-1.22123200	3.54289800	0.56657100
H	0.57683500	4.92331300	-0.44195700
H	2.64277300	3.83903000	-1.30098600
H	2.91534200	1.37720100	-1.09450700
H	1.13215200	0.01313300	-0.05396400
H	-1.02924600	-1.77753400	-3.83213600
H	-2.43739000	-2.89974000	-3.74188900
H	-2.71420800	-1.13242400	-3.78558200
H	0.19164400	-2.69379600	-1.72116300
H	-0.99092300	-4.06356800	-1.63828800
H	-0.65162100	-3.07927800	-0.17315100
H	-2.36529700	1.58055300	-1.62187800
H	2.93255700	0.53416100	-3.86172900
H	2.13640000	-0.27296200	-5.23900200

H	2.68525900	1.42498300	-5.39482700
H	-1.09091600	2.09118600	-3.50295000

1a

C	4.27200300	-2.31064200	0.09105000
C	3.23323800	-2.68659600	-0.76116200
C	2.03990300	-1.97071500	-0.77915700
C	1.87028500	-0.87742200	0.07412100
C	2.90777300	-0.50074200	0.92934700
C	4.10565800	-1.21214000	0.93205700
N	0.67014500	-0.10680300	0.04778900
C	-0.57865300	-0.72633400	0.11083900
C	0.80559800	1.31352400	-0.05176100
C	0.42928700	2.13411200	1.01240400
C	0.57289400	3.51552200	0.90791700
C	1.10799200	4.07864300	-0.25049900
C	1.49603500	3.25532000	-1.30732200
C	1.34154600	1.87441500	-1.21126100
C	-1.72011000	0.09707800	-0.14764300
O	-0.66231600	-1.93617500	0.38965600
S	-3.22709800	-0.61710200	-0.14566600
O	-3.63378100	-1.55888800	-1.22065600
C	-4.35294800	0.76966400	-0.13067900
C	-3.52193300	-1.43146600	1.42174400
H	5.20345800	-2.86769600	0.09628200
H	3.35494000	-3.53671500	-1.42529000
H	1.23615700	-2.25876200	-1.44676900
H	2.77730800	0.35328200	1.58670800
H	4.90659000	-0.90843000	1.59895400
H	0.02231800	1.68391000	1.91229100
H	0.27680700	4.15115700	1.73642900
H	1.22554700	5.15477400	-0.32790900
H	1.91421900	3.68824100	-2.21046100
H	1.63596300	1.22393200	-2.02913300
H	-1.69170400	1.10137600	-0.54436000
H	-5.36313600	0.35958900	-0.13589500
H	-4.17327300	1.37903100	0.75431200
H	-4.17542600	1.34072100	-1.04258900
H	-3.41419600	-0.69850000	2.22085700
H	-4.52769600	-1.85096800	1.38861100
H	-2.76785700	-2.21308200	1.50387000

2b

C	1.89509800	-0.34124900	0.00010100
C	0.66980400	0.43652700	0.00031200
O	-0.40432100	-0.34663800	0.00024600

O	0.64671500	1.64953300	-0.00003500
C	-1.67656200	0.32536400	0.00003600
C	2.94256300	-0.93877700	-0.00012100
H	-1.77317400	0.94405000	0.89360900
H	-1.77287500	0.94409600	-0.89353700
H	3.86948100	-1.47170500	-0.00019500
H	-2.41903500	-0.46925400	-0.00010900

TS0-A

C	5.17022100	-2.66956400	-0.39080600
C	4.33884200	-2.76941600	0.72513900
C	3.19033000	-1.98978000	0.81629500
C	2.85947400	-1.11492900	-0.21935600
C	3.68587500	-1.01320600	-1.33727600
C	4.84142600	-1.78781600	-1.41809600
N	1.68721100	-0.29466400	-0.10869800
C	0.45079300	-0.92753300	0.01806000
C	1.82194900	1.07360500	-0.50709700
C	1.19638300	1.53265200	-1.66535200
C	1.35012000	2.86130900	-2.05688800
C	2.15616900	3.71972200	-1.31293200
C	2.81867600	3.24356000	-0.18091800
C	2.65269600	1.92394800	0.22329900
C	-0.73384500	-0.17378700	0.47270300
O	0.33441500	-2.12938800	-0.22115000
S	-0.63405900	0.95425900	1.77498900
O	-0.40735200	2.36682400	1.42108600
C	0.59071800	0.45764200	2.98366400
C	-2.18935700	0.74658300	2.62053300
C	-1.94587900	0.90921500	-0.90214100
C	-3.13818100	0.54156300	-1.00802300
C	-4.13209200	-0.33802800	-0.48996200
O	-4.38539700	-1.37977100	-1.30268800
O	-4.72156400	-0.16701600	0.57434900
C	-5.37755700	-2.30723900	-0.84986500
H	6.06913000	-3.27384800	-0.45694900
H	4.58898700	-3.45061000	1.53216600
H	2.54492500	-2.06433200	1.68533500
H	3.42932900	-0.32780600	-2.13854300
H	5.48111700	-1.70331600	-2.29076100
H	0.61248200	0.84244400	-2.26611200
H	0.85215000	3.21742000	-2.95289300
H	2.27991300	4.75271400	-1.62093300
H	3.46052400	3.90404800	0.39282900
H	3.17252200	1.54799300	1.09931000

H	-1.52036800	-0.87936100	0.72093200
H	0.36806100	-0.55731900	3.31440800
H	0.54632300	1.16969500	3.80878600
H	1.56156500	0.50015100	2.49064000
H	-2.23244300	-0.25424400	3.05092000
H	-2.22748900	1.51455500	3.39362800
H	-2.98740600	0.88390800	1.88660100
H	-1.20463500	1.62699000	-1.20027500
H	-5.43631800	-3.06888200	-1.62530700
H	-5.08017100	-2.75601500	0.10031500
H	-6.34232600	-1.80941400	-0.73230600

TS0-B

C	-5.61863100	-0.21584200	-0.97559800
C	-4.63454900	-0.66952600	-1.85414500
C	-3.29202200	-0.62744100	-1.48981900
C	-2.93101900	-0.14322200	-0.23182000
C	-3.91102800	0.31123700	0.65023600
C	-5.25223900	0.27834000	0.27480800
N	-1.55328100	-0.06255800	0.14656300
C	-0.73866100	-1.17216500	0.06413700
C	-1.07197100	1.20370700	0.61589600
O	-1.19295400	-2.29861400	-0.18148900
C	-0.95200400	2.26631000	-0.27733900
C	-0.50272900	3.50236700	0.17942100
C	-0.16730600	3.67367900	1.52201300
C	-0.28909900	2.60747400	2.41256200
C	-0.75033900	1.37268900	1.96260200
S	1.60094800	-2.35057900	0.58755700
O	1.83649300	-3.33467600	-0.48413500
C	3.13865800	-1.67827000	1.18331800
C	0.89246400	-3.18856500	2.00151000
C	1.31466700	-0.54820100	-1.82148900
C	2.19722000	0.31681600	-2.00819000
C	3.08199700	1.26940900	-1.42807100
O	4.23333100	0.69170000	-1.00540600
O	2.87493100	2.47083100	-1.32532700
H	-6.66381100	-0.24391500	-1.26634000
H	-4.91097800	-1.04901700	-2.83271900
H	-2.52365600	-0.96989600	-2.17399100
H	-3.62265200	0.69544000	1.62362100
H	-6.01021300	0.63597500	0.96442500
H	-1.20264800	2.11548300	-1.32246700
H	-0.40117300	4.32780300	-0.51721500
H	0.18905300	4.63642100	1.87369500

H	-0.03491700	2.73867200	3.45945300
H	-0.86187300	0.53626200	2.64633900
H	3.78857900	-2.52505300	1.40601800
H	2.95228300	-1.07831600	2.07469500
H	3.55751100	-1.07031400	0.37732000
H	-0.08993800	-3.53926000	1.68599700
H	1.54648800	-4.02362300	2.25511400
H	0.81275600	-2.47581400	2.82290800
C	0.69649800	-0.94986500	0.17895600
H	1.09528600	-0.06853200	0.66482700
C	5.21620200	1.57512900	-0.45477600
H	6.05866800	0.93966300	-0.18661600
H	5.52210700	2.31718700	-1.19512700
H	4.82605600	2.08302900	0.42988600
H	0.65980000	-1.27331100	-2.27108800
**TS0-C**			
C	3.54441500	-2.20524800	1.77383400
C	3.69923600	-2.17557900	0.38927900
C	2.92693400	-1.30641800	-0.38036900
C	1.98267300	-0.48259800	0.23660200
C	1.83449500	-0.50234100	1.62470300
C	2.61671100	-1.36115800	2.38910100
N	1.19884600	0.41646700	-0.54714900
C	0.61710100	-0.05495500	-1.73508100
C	1.48556600	1.81290100	-0.45719200
C	0.44593200	2.72446600	-0.66151500
C	0.69315200	4.08913500	-0.56801200
C	1.96955600	4.55590400	-0.24867500
C	2.99883200	3.64348200	-0.03070300
C	2.76365700	2.27348800	-0.14121900
C	-0.40400800	-1.10674500	-1.69761300
O	0.81957300	0.49482700	-2.81600000
S	-0.85305700	-2.06998300	-0.32495200
O	-1.09783500	-1.31753800	0.91253700
C	-2.31564600	-2.93158100	-0.87895500
C	0.27425700	-3.43080300	-0.02198300
C	-1.97369400	0.11801700	-2.10051300
C	-2.87750200	0.46914100	-1.29734300
C	-3.47643000	0.32446300	-0.01919000
O	-3.09326200	1.28659900	0.84437500
C	-3.63112100	1.19441000	2.16505400
O	-4.29891900	-0.54211200	0.27677700
H	4.14674300	-2.87918100	2.37413100
H	4.42561800	-2.82068500	-0.09393800

H	3.05642500	-1.26271400	-1.45760600
H	1.09666000	0.14398800	2.08640700
H	2.49448100	-1.38163100	3.46705300
H	-0.55031100	2.35658400	-0.88878200
H	-0.11901300	4.79060500	-0.73105500
H	2.15703300	5.62166500	-0.16667600
H	3.99543800	3.99437900	0.21832400
H	3.57172400	1.56781600	0.02102500
H	-0.42669500	-1.73662000	-2.58379600
H	-2.10741900	-3.42048000	-1.83156200
H	-2.55403900	-3.66334200	-0.10629300
H	-3.11361800	-2.19346000	-0.96403000
H	0.49580500	-3.91871700	-0.97260900
H	-0.23093100	-4.11099900	0.66552900
H	1.17715000	-3.02608600	0.43052000
H	-1.60588700	0.32987000	-3.09256700
H	-3.20650800	2.03589500	2.71043300
H	-4.72089500	1.26712300	2.14625400
H	-3.33620700	0.25264700	2.63334400

TS0-D

C	5.34231200	0.00315600	-1.55216900
C	4.87872300	-1.21405400	-1.05789700
C	3.56458500	-1.33755500	-0.61137300
C	2.71208900	-0.23545500	-0.65135300
C	3.17524000	0.98815700	-1.13705900
C	4.48453700	1.10269500	-1.59285700
N	1.36938800	-0.33973600	-0.15672000
C	0.32014300	-0.04887900	-1.02955100
C	1.18640800	-1.08202300	1.05338200
C	1.74617400	-0.60968500	2.24105300
C	1.60913000	-1.34734300	3.41109700
C	0.91132700	-2.55581900	3.40186300
C	0.37603300	-3.03798900	2.20991700
C	0.52871200	-2.31102100	1.03048400
C	-1.03196400	0.22411500	-0.50122400
O	0.51190100	0.01668500	-2.24287200
S	-1.24211200	1.23803100	0.88066100
O	-1.34189100	0.57166200	2.19124400
C	-2.73156900	2.14609300	0.51287000
C	0.02578900	2.49927700	0.96671600
C	-2.40062900	-1.33834700	-0.07084600
C	-3.47167800	-1.34918500	-0.71947600
C	-4.24157700	-0.72439900	-1.74164900
O	-4.98556700	0.29676900	-1.25139000

O	-4.27167100	-1.05315900	-2.92004000
C	-5.83163100	0.96743200	-2.19125900
H	6.36534600	0.09651100	-1.90179000
H	5.53776400	-2.07561500	-1.02305900
H	3.20390200	-2.28653200	-0.22808300
H	2.50851500	1.84383800	-1.16572500
H	4.83757400	2.05608900	-1.97277300
H	2.29439600	0.32751800	2.24356500
H	2.04095000	-0.97469900	4.33418200
H	0.79787800	-3.12503500	4.31858300
H	-0.14666200	-3.98875200	2.18963000
H	0.15929300	-2.70228100	0.08776600
H	-2.95768000	2.74165100	1.39777200
H	-3.51946800	1.41500900	0.31874300
H	-2.56410800	2.77856700	-0.35942900
H	0.03799000	3.05039800	0.02571900
H	-0.21313000	3.14749300	1.81066200
H	0.97461600	1.98866000	1.12946800
H	-1.83582600	-1.80106700	0.71709800
H	-5.23872200	1.42154800	-2.98806500
H	-6.55067200	0.26964500	-2.62533700
H	-6.34902600	1.73562600	-1.61885000
H	-1.64022100	0.63581700	-1.30038800
**TS1-E-A**			
C	-4.68368400	-3.82800600	-1.43040700
C	-3.63032800	-3.41154900	-2.24452000
C	-2.75337600	-2.42457100	-1.80391500
C	-2.93193100	-1.86464700	-0.54126100
C	-3.97989900	-2.27278700	0.27866400
C	-4.85801600	-3.25651300	-0.17114500
N	-2.04477900	-0.81895300	-0.09745600
C	-0.74935600	-1.14293500	0.14916200
C	-2.65200500	0.45159700	0.18264700
C	-2.80806000	0.86719400	1.50181900
C	-3.38818500	2.10771100	1.76234700
C	-3.82707000	2.90840000	0.71001000
C	-3.69282800	2.46749700	-0.60785100
C	-3.10693600	1.23534700	-0.87525600
C	0.32774800	-0.04469700	0.32925600
O	-0.35130400	-2.30448200	0.16036500
S	0.97228500	-0.21099500	2.05093700
O	-0.06985100	-0.06611300	3.07927100
C	2.23654300	1.03112700	2.17388000
C	1.84814500	-1.75262100	2.19036000

C	0.13037300	1.40746600	-0.01885700
C	0.95117700	1.96478600	-0.91966300
C	0.76925500	3.35179900	-1.31691400
O	1.53888900	4.19705000	-0.59340100
O	0.08213100	3.74023500	-2.25356600
C	1.52016700	5.57146500	-0.99139800
H	-5.36866900	-4.59422700	-1.77863300
H	-3.49390200	-3.85041500	-3.22743800
H	-1.93344300	-2.08846300	-2.43013200
H	-4.10929800	-1.82088400	1.25693400
H	-5.67667900	-3.57635100	0.46516700
H	-2.45085300	0.23814700	2.31014700
H	-3.49688400	2.44454200	2.78798400
H	-4.27637000	3.87476400	0.91429100
H	-4.03216300	3.09065300	-1.42839300
H	-2.97479000	0.88931300	-1.89503900
H	2.74669300	0.86390300	3.12364100
H	1.74681000	2.00387100	2.14784900
H	2.91384700	0.91237200	1.32403000
H	2.68469500	-1.75734700	1.48264700
H	2.20476300	-1.79887300	3.22096500
H	1.13727900	-2.54776200	1.96968700
H	-0.66059400	1.94286300	0.50214900
H	1.86053000	5.67772300	-2.02376900
H	0.51461100	5.98724400	-0.89691400
H	2.20392900	6.08136600	-0.31433000
H	1.17018100	-0.43167800	-0.25547600
C	3.98790400	-0.50077400	-0.97022600
C	4.84616200	-1.55960700	-0.50478600
O	5.84909400	-1.81789200	-1.34230500
O	4.67020500	-2.15498500	0.54672400
C	6.74929600	-2.86660400	-0.95097400
C	3.21996400	0.39122800	-1.30436900
H	7.23532500	-2.61412400	-0.00693500
H	6.20868300	-3.80906000	-0.84842000
H	2.21843100	1.18881200	-1.31584000
H	7.48108500	-2.93013900	-1.75329800

TS1-E-B

C	-5.65969500	-2.14632100	-0.32343500
C	-4.79914400	-2.35462900	-1.39998200
C	-3.48049600	-1.91003900	-1.33803300
C	-3.03209400	-1.25237600	-0.19554900
C	-3.88649700	-1.03319400	0.88274900
C	-5.20080800	-1.48707100	0.81683600

N	-1.68235800	-0.75662200	-0.14978100
C	-0.83065900	-1.22152900	0.79836600
C	-1.29509600	0.18234200	-1.16992900
C	-0.44532300	-0.21610000	-2.19887000
C	-0.10884400	0.69373100	-3.19847000
C	-0.63034500	1.98449700	-3.17255600
C	-1.48445800	2.37320800	-2.14063900
C	-1.81810800	1.47341100	-1.13329700
C	0.56108000	-0.58970500	0.84499200
O	-1.11053700	-2.10861200	1.59937000
S	0.80063800	-0.08378600	2.59849900
O	-0.36935000	0.59512600	3.17427900
C	1.28490700	-1.52152600	3.52841200
C	2.22905800	0.97046300	2.55824200
C	1.64688500	-1.54136900	0.40267400
C	2.71969000	-1.09726100	-0.26129100
C	3.78268800	-2.00915000	-0.65575800
O	3.64581400	-2.44481700	-1.92414500
C	4.71079600	-3.25105600	-2.43949600
O	4.75577000	-2.29206000	0.03337800
H	-6.68629900	-2.49431900	-0.37341800
H	-5.15118000	-2.86614300	-2.28979200
H	-2.80433100	-2.06742600	-2.17221900
H	-3.52079800	-0.51414100	1.76228900
H	-5.86850500	-1.31900900	1.65549500
H	-0.04706200	-1.22556600	-2.20910900
H	0.56408100	0.39165100	-3.99377400
H	-0.36346000	2.69350300	-3.94909500
H	-1.88232700	3.38238600	-2.11487000
H	-2.47615600	1.76298400	-0.32005900
H	0.62534600	0.36909300	0.32215900
H	2.18531200	-1.94136300	3.07829100
H	1.46924300	-1.18261200	4.54884600
H	0.44921000	-2.21872600	3.47457900
H	3.02443600	0.46440600	2.00809900
H	2.51205400	1.16668100	3.59291400
H	1.92036600	1.88406600	2.04716300
H	1.45998800	-2.58572700	0.66578600
H	5.65110900	-2.69570600	-2.42911000
H	4.82150100	-4.16484500	-1.85185900
H	4.42553600	-3.49198500	-3.46223600
C	1.83665100	2.62667100	-0.61324900
C	0.99128800	3.75972800	-0.34855900
O	0.28608300	3.56304600	0.77832200

O	0.90221000	4.76263800	-1.03096100
C	-0.65025200	4.58974900	1.12899100
C	2.50003300	1.61560500	-0.80347700
H	-0.13580100	5.53984800	1.28441600
H	-1.39869200	4.70145500	0.34142900
H	2.78224000	0.36577200	-0.64942300
H	-1.11695200	4.25160700	2.05202800

TS1-Z

C	4.34518700	4.55866300	-0.39220000
C	2.97600000	4.47595000	-0.64467700
C	2.31139200	3.26001500	-0.51228100
C	3.02446700	2.13141100	-0.11510300
C	4.39114300	2.20319000	0.14035900
C	5.05095800	3.42156800	-0.00244800
N	2.35158900	0.86380300	-0.00533700
C	1.35120500	0.71985700	0.90112000
C	2.82594100	-0.20497500	-0.84382200
C	2.60286400	-0.14210400	-2.21695000
C	3.07506600	-1.16654500	-3.03229300
C	3.76053100	-2.24623500	-2.47587200
C	3.98304400	-2.29913100	-1.10070600
C	3.52483700	-1.27128700	-0.27939100
C	0.50386100	-0.55551100	0.80494800
O	1.05178000	1.56516400	1.73704400
S	0.63610300	-1.36766400	2.44650100
O	2.02090600	-1.43125600	2.93565400
C	-0.44741800	-0.51103100	3.56556500
C	-0.07334900	-2.98139900	2.22504800
C	-0.93308800	-0.23838500	0.46469600
C	-1.69063900	-0.90672300	-0.40905500
C	-1.23623200	-2.03833200	-1.20064900
O	-1.52013000	-3.22281800	-0.61106200
C	-1.20397000	-4.40110100	-1.36136200
O	-0.73299000	-1.96894400	-2.31318800
H	4.86100000	5.50705400	-0.50157300
H	2.42352500	5.35763400	-0.95235500
H	1.24791600	3.18309200	-0.71274200
H	4.93272000	1.31286600	0.44332500
H	6.11633700	3.48066400	0.19447200
H	2.05627700	0.69701400	-2.63464600
H	2.89636300	-1.12670100	-4.10144000
H	4.12134700	-3.04572800	-3.11451400
H	4.52079000	-3.13493600	-0.66544300
H	3.69569300	-1.29409700	0.79277700

H	0.92480800	-1.30240500	0.12835200
H	-1.46735200	-0.57091500	3.18449400
H	-0.34962800	-1.01565400	4.52754700
H	-0.09755400	0.52064600	3.60783000
H	-1.03556600	-2.88634900	1.71892600
H	-0.17538500	-3.42225600	3.21751400
H	0.62995100	-3.55027500	1.61524900
H	-1.33490500	0.63598500	0.97948000
H	-1.75463800	-4.41366100	-2.30391600
H	-0.13200500	-4.44991400	-1.56523600
H	-1.50909100	-5.23680100	-0.73371700
C	-5.32634700	0.76347600	-0.28298500
C	-6.60441200	1.39454600	-0.08748600
O	-7.28033900	1.53544100	-1.23721500
O	-7.03957500	1.76467400	0.98996400
C	-8.56899400	2.15570700	-1.13919100
C	-4.22919600	0.22978800	-0.39844600
H	-9.22735800	1.56658200	-0.49761900
H	-8.47552500	3.16682700	-0.73805400
H	-3.02693100	-0.37284700	-0.48913800
H	-8.95389200	2.18361800	-2.15672800

TS2-E

C	2.93520000	4.61673200	-1.75959200
C	1.68930800	4.07643700	-2.07874600
C	1.25873500	2.90140100	-1.46995400
C	2.07452600	2.27656900	-0.52873800
C	3.31881200	2.80988200	-0.20238600
C	3.74889100	3.98013500	-0.82393400
N	1.64004300	1.04903800	0.08008000
C	0.51209000	1.08042100	0.86001000
C	2.54346200	-0.05852700	0.02868200
C	3.00406800	-0.64369800	1.20510900
C	3.86932000	-1.73605600	1.13675500
C	4.29607600	-2.21505400	-0.09799300
C	3.85537500	-1.60018300	-1.27250100
C	2.97835100	-0.52492400	-1.21279000
C	-0.24065800	-0.21284400	1.13220800
O	0.02301700	2.13340400	1.25615100
S	-0.53514700	-0.44581700	2.86953900
O	0.63862200	-0.70906800	3.71846400
C	-1.70263600	-1.78958500	2.88988100
C	-1.48485200	0.94523300	3.44035900
C	0.12796100	-1.50025800	0.47522500
C	-0.20871000	-1.73633300	-0.79461600

C	0.21613900	-2.99459700	-1.44976000
O	-0.05817300	-2.97187000	-2.75717900
O	0.74798300	-3.93424700	-0.88879500
C	0.31766300	-4.13498100	-3.50657700
H	3.27129700	5.52934100	-2.24100900
H	1.05380500	4.56500500	-2.81014200
H	0.29488900	2.46868600	-1.71815800
H	3.94628400	2.30823700	0.52755600
H	4.71927400	4.39539500	-0.57193900
H	2.67815600	-0.26029100	2.16711100
H	4.21524500	-2.20159300	2.05374200
H	4.97197700	-3.06219200	-0.14931600
H	4.18648000	-1.96956100	-2.23762400
H	2.61347700	-0.05372300	-2.11992000
H	-2.06347400	-1.88772300	3.91394400
H	-1.18203100	-2.69461500	2.57754300
H	-2.51143200	-1.53503400	2.19800800
H	-2.31371400	1.08805200	2.74181400
H	-1.82970500	0.68788700	4.44280900
H	-0.82875200	1.81300600	3.44237600
H	0.69014800	-2.26072500	1.01425600
H	1.39618900	-4.29204900	-3.44334900
H	-0.20446200	-5.01486100	-3.12621400
H	0.02059500	-3.92827800	-4.53258100
H	-1.40175800	0.04988900	0.74773800
C	-4.07603800	0.41595500	-0.08602000
C	-5.44614500	0.58441500	-0.48530400
O	-5.55614000	1.01156600	-1.75365200
O	-6.41347600	0.37011600	0.22747100
C	-6.88972900	1.20992800	-2.23917500
C	-2.91617300	0.26117400	0.29660200
H	-7.45153800	0.27425200	-2.20580300
H	-7.40402500	1.96670200	-1.64334800
H	-0.75028800	-1.01123100	-1.39364400
H	-6.77548400	1.54856400	-3.26719400

TS2-Z

C	5.42865200	-2.54581700	-0.93535800
C	5.41489400	-1.70540900	0.17792000
C	4.28414000	-0.94714700	0.46881900
C	3.16381400	-1.04182000	-0.35368000
C	3.16854700	-1.87919900	-1.46612600
C	4.30577200	-2.62945500	-1.75716500
N	2.00163800	-0.23870400	-0.08520600
C	1.28564500	-0.47867200	1.05563100

C	1.64298500	0.72641400	-1.08641000
C	2.50536000	1.79538900	-1.32946500
C	2.15943900	2.75590300	-2.27314900
C	0.95574600	2.64869300	-2.97154500
C	0.11381300	1.56273100	-2.74276000
C	0.46308300	0.58715500	-1.81165500
C	0.10637800	0.42679800	1.34113000
O	1.55862700	-1.42202800	1.79790200
S	-0.43426400	0.10943000	3.02176600
O	0.48673800	0.47414800	4.11382200
C	-1.95397400	1.02467800	3.14394200
C	-0.99255100	-1.57986100	3.13796700
C	0.42122200	1.89810300	1.22738800
C	-0.30101100	2.89264200	0.70100800
C	-1.63312400	2.78534800	0.05963200
O	-1.82629700	3.81201100	-0.77515800
C	-3.06121300	3.81117100	-1.50283900
O	-2.45869600	1.91642400	0.25280100
H	6.31298700	-3.13254400	-1.16215200
H	6.28770700	-1.63530900	0.81899200
H	4.26342200	-0.28798700	1.33042600
H	2.28803300	-1.93817700	-2.09819600
H	4.31083400	-3.28199500	-2.62417200
H	3.42845200	1.87820700	-0.76416100
H	2.82276600	3.59519800	-2.45509600
H	0.67949000	3.40743300	-3.69655700
H	-0.81796600	1.46758300	-3.29089700
H	-0.19366300	-0.25588800	-1.62336300
H	-0.88034400	-0.13846600	0.65953400
H	-2.57625100	0.77388400	2.28272500
H	-2.41354300	0.74098100	4.09117100
H	-1.70269800	2.08491900	3.13913900
H	-1.59718700	-1.79028600	2.25079800
H	-1.58054700	-1.63946800	4.05474100
H	-0.10816900	-2.20973300	3.17199200
H	1.40058000	2.18310200	1.60974500
H	-3.13493500	2.91175900	-2.11743300
H	-3.90743100	3.85505900	-0.81460100
H	-3.03036200	4.70061300	-2.12865300
C	-2.68512200	-1.74018300	-0.63268000
C	-3.60627100	-2.52759100	-1.40350000
O	-4.52846000	-3.12821100	-0.63280500
O	-3.57515900	-2.65194600	-2.61691200
C	-5.49238200	-3.93675100	-1.31884900

C	-1.87797300	-1.04312800	-0.02076100
H	-6.06497400	-3.33175100	-2.02472500
H	-4.99740600	-4.75068000	-1.85240200
H	0.14235400	3.88270000	0.67076000
H	-6.14362800	-4.33224100	-0.54160000

8. Characterization Data for the Products

ethyl (*E*)-4-(dimethyl(oxo)-λ^6-sulfanylidene)-5-(diphenylamino)-5-oxopent-2-enoate (3)

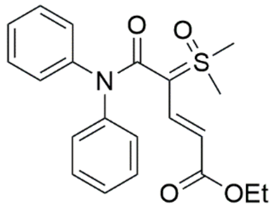

The title compound was prepared according to the general procedure and purified by column chromatography on silica gel and eluted with petroleum ether/ethyl acetate (1/2) to afford a colorless oil in 79% yield (30.6 mg). ^{1}H NMR (600 MHz, $CDCl_3$): δ 7.28 (dd, J = 8.4, 7.5 Hz, 4H), 7.19 (d, J = 15.4 Hz, 1H), 7.14–7.09 (m, 6H), 5.42 (d, J = 15.4 Hz, 1H), 3.99 (q, J = 7.1 Hz, 2H), 3.52 (s, 6H), 1.14 (t, J = 7.1 Hz, 3H). ^{13}C NMR (150 MHz, $CDCl_3$): δ 168.3, 168.2, 144.1, 135.1, 128.9, 126.3, 125.4, 102.7, 77.9, 59.0, 43.4, 14.4. HRMS (ESI-TOF) *m/z* calcd. for $C_{21}H_{24}NO_4S^+$ ([M+H]$^+$) 386.1421. found, 386.1425.

ethyl (*Z*)-4-(dimethyl(oxo)-λ^6-sulfanylidene)-5-(diphenylamino)-5-oxopent-2-enoate (3')

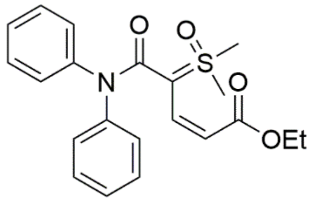

The title compound was prepared according to the general procedure and purified by column chromatography on silica gel and eluted with petroleum ether/ethyl acetate (1/1) to afford a colorless oil in 8% yield (3.0 mg). ^{1}H NMR (600 MHz, $CDCl_3$): δ 7.66 (d, J = 14.9 Hz, 1H), 7.32 (t, J = 7.6 Hz, 5H), 7.27–7.23 (m, 3H), 7.18 (t, J = 6.9 Hz, 2H), 6.08 (d, J = 14.9 Hz, 1H), 3.96 (q, J = 7.1 Hz, 2H), 3.42 (s, 6H), 0.89 (t, J = 7.1 Hz, 3H). ^{13}C NMR (150 MHz, $CDCl_3$): δ 168.6, 165.8, 143.8, 133.4, 128.9, 127.9, 125.9, 105.7, 74.2, 59.8, 43.1, 13.9. HRMS (ESI-TOF) *m/z* calcd. for $C_{21}H_{24}NO_4S^+$ ([M+H]$^+$) 386.1421. found, 386.1427.

ethyl (*E*)-5-(di-*p*-tolylamino)-4-(dimethyl(oxo)-λ^6-sulfaneylidene)-5-oxopent-2-enoate (4)

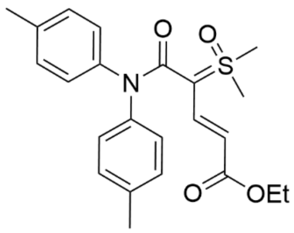

The title compound was prepared according to the general procedure and purified by column chromatography on silica gel and eluted with petroleum ether/ethyl acetate (1/2) to afford a colorless oil in 83% yield (29.3 mg). ^{1}H NMR (600 MHz, $CDCl_3$): δ 7.21 (d, *J* = 15.4 Hz, 1H), 7.08 (d, *J* = 8.1 Hz, 4H), 7.00 (d, *J* = 8.3 Hz, 4H), 5.41 (d, *J* = 15.4 Hz, 1H), 4.01 (q, *J* = 7.1 Hz, 2H), 3.53 (s, 6H), 2.29 (s, 6H), 1.16 (t, *J* = 7.1 Hz, 3H). ^{13}C NMR (150 MHz, $CDCl_3$): δ 168.4, 141.7, 135.4, 135.2, 129.6, 126.2, 102.5, 77.7, 59.1, 43.7, 20.9, 14.4. HRMS (ESI-TOF) *m/z* calcd. for $C_{23}H_{28}NO_4S^+$ ([M+H]$^+$) 414.1734. found, 414.1731.

ethyl (*E*)-5-(bis(4-methoxyphenyl)amino)-4-(dimethyl(oxo)-λ^6-sulfaneylidene)-5-oxopent-2-enoate (5)

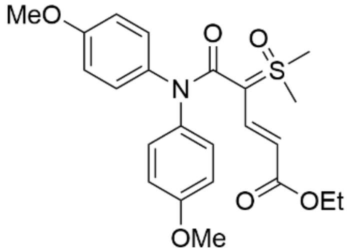

The title compound was prepared according to the general procedure and purified by column chromatography on silica gel and eluted with petroleum ether/ethyl acetate (1/2) to afford a colorless oil in 88% yield (39.2 mg). ^{1}H NMR (600 MHz, $CDCl_3$): δ 7.21 (d, *J* = 15.4 Hz, 1H), 7.04 (d, *J* = 9.0 Hz, 4H), 6.81 (d, *J* = 9.0 Hz, 4H), 5.39 (d, *J* = 15.4 Hz, 1H), 4.02 (q, *J* = 7.1 Hz, 2H), 3.75 (s, 6H), 3.53 (s, 6H), 1.17 (t, *J* = 7.1 Hz, 3H). ^{13}C NMR (150 MHz, $CDCl_3$): δ 168.4, 168.3, 157.2, 137.3, 135.3, 127.4, 114.3, 102.5, 77.4, 59.1, 55.4, 43.8, 14.5. HRMS (ESI-TOF) *m/z* calcd. for $C_{23}H_{28}NO_6S^+$ ([M+H]$^+$) 446.1632. found, 446.1635.

ethyl (*E*)-5-(bis(4-bromophenyl)amino)-4-(dimethyl(oxo)-λ⁶-sulfaneylidene)-5-oxopent-2-enoate (6)

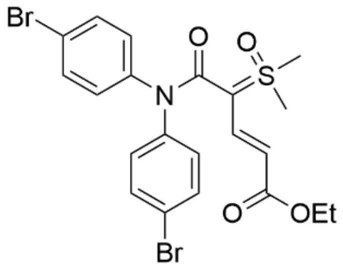

The title compound was prepared according to the general procedure and purified by column chromatography on silica gel and eluted with petroleum ether/ethyl acetate (1/2) to afford a yellow oil in 89% yield (50.2 mg). ^{1}H NMR (600 MHz, $CDCl_3$): δ 7.41 (d, *J* = 8.8 Hz, 4H), 7.16 (d, *J* = 15.5 Hz, 1H), 6.98 (d, *J* = 8.8 Hz, 4H), 5.40 (d, *J* = 15.5 Hz, 1H), 4.04 (q, *J* = 7.1 Hz, 2H), 3.55 (s, 6H), 1.19 (t, *J* = 7.1 Hz, 3H). ^{13}C NMR (150 MHz, $CDCl_3$): δ 167.92, 167.89, 142.8, 134.5, 132.2, 127.7, 119.0, 103.9, 78.0, 59.3, 43.4, 14.4. HRMS (ESI-TOF) *m/z* calcd. for $C_{21}H_{22}Br_2NO_4S^+$ ($[M+H]^+$) 541.9631. found, 541.9634.

ethyl (*E*)-5-(bis(4-(trifluoromethyl)phenyl)amino)-4-(dimethyl(oxo)-λ⁶-sulfaneylidene)-5-oxopent-2-enoate (7)

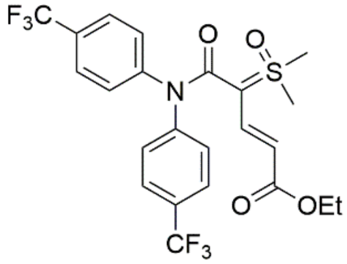

The title compound was prepared according to the general procedure and purified by column chromatography on silica gel and eluted with petroleum ether/ethyl acetate (1/2) to afford a yellow oil in 92% yield (47.9 mg). ^{1}H NMR (600 MHz, $CDCl_3$): δ 7.56 (d, *J* = 8.5 Hz, 4H), 7.21 (d, *J* = 8.4 Hz, 4H), 7.13 (d, *J* = 15.5 Hz, 1H), 5.42 (d, *J* = 15.5 Hz, 1H), 4.00 (q, *J* = 7.1 Hz, 2H), 3.58 (s, 6H), 1.14 (t, *J* = 7.1 Hz, 3H). ^{13}C NMR (150 MHz, $CDCl_3$): δ 168.1, 167.7, 146.7, 134.0, 127.6 (d, *J* = 32.9 Hz), 126.4 (q, *J* = 3.6 Hz), 126.1, 123.8 (d, *J* = 271.9 Hz), 105.1, 78.5, 59.4, 43.3, 14.3. ^{19}F NMR (564 MHz, $CDCl_3$): δ -62.4 (s, 6F). HRMS (ESI-TOF) *m/z* calcd. for $C_{23}H_{22}F_6NO_4S^+$ ($[M+H]^+$) 522.1168. found, 522.1162.

ethyl *(E)*-4-(dimethyl(oxo)-λ⁶-sulfaneylidene)-5-oxo-5-(phenyl(p-tolyl)amino)pent-2-enoate (8)

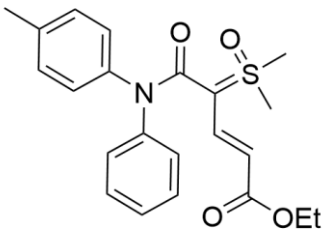

The title compound was prepared according to the general procedure and purified by column chromatography on silica gel and eluted with petroleum ether/ethyl acetate (1/2) to afford a yellow oil in 81% yield (32.3 mg). ^{1}H NMR (600 MHz, $CDCl_3$): δ 7.27 (d, *J* = 7.8 Hz, 2H), 7.20 (d, *J* = 15.4 Hz, 1H), 7.12–7.07 (m, 5H), 7.00 (d, *J* = 8.3 Hz, 2H), 5.41 (d, *J* = 15.4 Hz, 1H), 4.00 (q, *J* = 7.1 Hz, 2H), 3.52 (s, 6H), 2.29 (s, 3H), 1.15 (t, *J* = 7.1 Hz, 3H). ^{13}C NMR (150 MHz, $CDCl_3$): δ 168.3, 168.2, 144.3, 141.5, 135.3, 135.2, 129.6, 128.9, 126.3, 126.1, 125.2, 102.6, 77.8, 59.0, 43.5, 20.8, 14.4. HRMS (ESI-TOF) *m/z* calcd. for $C_{22}H_{26}NO_4S^+$ ($[M+H]^+$) 400.1577. found, 400.1573.

ethyl (*E*)-4-(dimethyl(oxo)-λ⁶-sulfaneylidene)-5-oxo-5-(phenyl(m-tolyl)amino)pent-2-enoate (9)

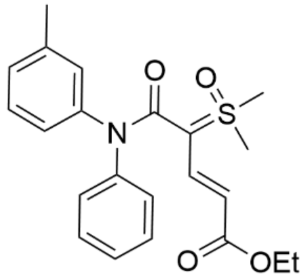

The title compound was prepared according to the general procedure and purified by column chromatography on silica gel and eluted with petroleum ether/ethyl acetate (1/1) to afford a yellow oil in 80% yield (31.9 mg). ^{1}H NMR (600 MHz, $CDCl_3$): δ 7.28 (t, *J* = 7.8 Hz, 2H), 7.18 (dd, *J* = 15.7, 10.5 Hz, 2H), 7.11 (d, *J* = 8.2 Hz, 3H), 6.96 (d, *J* = 7.6 Hz, 1H), 6.92 (d, *J* = 6.4 Hz, 2H), 5.42 (d, *J* = 15.4 Hz, 1H), 4.00 (q, *J* = 7.1 Hz, 2H), 3.53 (s, 6H), 2.28 (s, 3H), 1.16 (t, *J* = 7.1 Hz, 3H). ^{13}C NMR (150 MHz, $CDCl_3$): δ 168.4, 168.3, 144.2, 144.1, 138.9, 135.1, 128.9, 128.8, 127.3, 126.5, 126.2, 125.5, 123.6, 102.9, 77.9, 59.1, 43.6, 21.3, 14.4. HRMS (ESI-TOF) *m/z* calcd. for $C_{22}H_{26}NO_4S^+$ ($[M+H]^+$) 400.1577. found, 400.1575.

ethyl (*E*)-4-(dimethyl(oxo)-λ^6-sulfaneylidene)-5-oxo-5-(phenyl(o-tolyl)amino)pent-2-enoate (10)

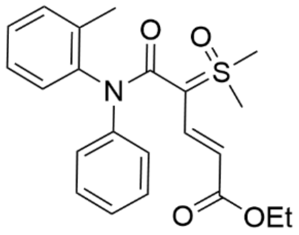

The title compound was prepared according to the general procedure and purified by column chromatography on silica gel and eluted with petroleum ether/ethyl acetate (1/1) to afford a white solid in 78% yield (30.3 mg). ^{1}H NMR (600 MHz, $CDCl_3$): δ 7.29 (q, *J* = 7.7 Hz, 4H), 7.21 (t, *J* = 4.7 Hz, 2H), 7.11 (q, *J* = 7.5 Hz, 4H), 5.43 (d, *J* = 15.5 Hz, 1H), 4.04 (q, *J* = 7.1 Hz, 2H), 3.59 (s, 6H), 2.24 (s, 3H), 1.20 (t, *J* = 7.1 Hz, 3H). ^{13}C NMR (150 MHz, $CDCl_3$): δ 168.2, 167.6, 143.7, 142.8, 135.8, 135.1, 131.2, 128.8, 128.3, 126.84, 126.81, 125.0, 124.7, 101.9, 77.8, 59.0, 43.6, 18.5, 14.4. HRMS (ESI-TOF) *m/z* calcd. for $C_{22}H_{26}NO_4S^+$ ([M+H]$^+$) 4001577. found, 400.1573.

ethyl (*E*)-4-(dimethyl(oxo)-λ^6-sulfaneylidene)-5-((4-fluorophenyl)(phenyl)amino)-5-oxopent-2-enoate (11)

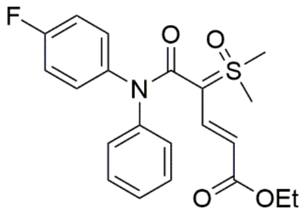

The title compound was prepared according to the general procedure and purified by column chromatography on silica gel and eluted with petroleum ether/ethyl acetate (1/1) to afford a yellow solid in 90% yield (36.3 mg). ^{1}H NMR (600 MHz, $CDCl_3$): δ 7.28 (dd, *J* = 8.3, 7.5 Hz, 2H), 7.17 (d, *J* = 15.4 Hz, 1H), 7.12 (t, *J* = 7.4 Hz, 1H), 7.10–7.07 (m, 4H), 6.99–6.95 (m, 2H), 5.40 (d, *J* = 15.4 Hz, 1H), 4.00 (q, *J* = 7.1 Hz, 2H), 3.52 (s, 6H), 1.15 (t, *J* = 7.1 Hz, 3H). ^{13}C NMR (150 MHz, $CDCl_3$): δ 168.2, 168.1, 160.17 (d, *J* = 245.8 Hz), 144.0, 140.1 (d, *J* = 2.9 Hz), 134.9, 129.0, 127.9 (d, *J* = 8.3 Hz), 126.1, 125.5, 115.8 (d, *J* = 22.7 Hz), 103.0, 77.7, 59.1, 43.4, 14.4. ^{19}F NMR (564 MHz, $CDCl_3$): δ -116.4 (s, 1F). HRMS (ESI-TOF) *m/z* calcd. for $C_{21}H_{23}FNO_4S^+$ ([M+H]$^+$) 404.1326. found, 404.1331.

ethyl (*E*)-4-(dimethyl(oxo)-λ^6-sulfaneylidene)-5-oxo-5-(phenyl(4-((*E*)-phenyldiazenyl)phenyl)amino)pent-2-enoate (12)

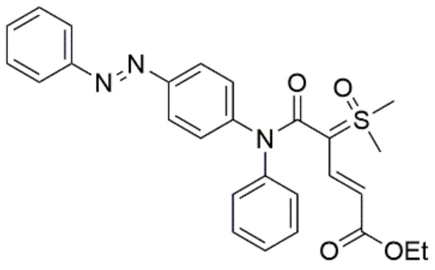

The title compound was prepared according to the general procedure and purified by column chromatography on silica gel and eluted with petroleum ether/ethyl acetate (1/2) to afford a brown oil in 83% yield (40.6 mg). ^{1}H NMR (600 MHz, CDCl$_3$): δ 7.87 (t, *J* = 8.5 Hz, 4H), 7.49 (t, *J* = 7.5 Hz, 2H), 7.44 (t, *J* = 7.2 Hz, 1H), 7.33 (t, *J* = 7.9 Hz, 2H), 7.25–7.20 (m, 3H), 7.20–7.15 (m, 3H), 5.49 (d, *J* = 15.5 Hz, 1H), 4.00 (q, *J* = 7.1 Hz, 2H), 3.57 (s, 6H), 1.14 (t, *J* = 7.1 Hz, 3H). ^{13}C NMR (150 MHz, CDCl$_3$): δ 168.3, 168.1, 152.6, 149.4, 146.7, 143.7, 134.8, 130.8, 129.2, 129.0, 126.7, 126.03, 126.00, 123.6, 122.7, 103.7, 78.4, 59.2, 43.5, 14.4. HRMS (ESI-TOF) *m/z* calcd. for $C_{27}H_{28}N_3O_4S^+$ ([M+H]$^+$) 490.1795. found, 490.1824.

ethyl (*E*)-4-(dimethyl(oxo)-λ^6-sulfaneylidene)-5-((3,4-dimethylphenyl)(phenyl)amino)-5-oxopent-2-enoate (13)

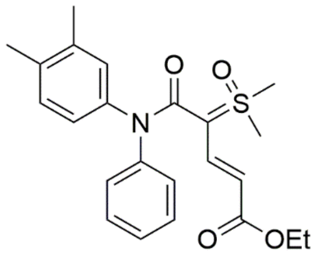

The title compound was prepared according to the general procedure and purified by column chromatography on silica gel and eluted with petroleum ether/ethyl acetate (1/1) to afford a yellow oil in 82% yield (31.6 mg). ^{1}H NMR (600 MHz, CDCl$_3$): δ 7.45–7.40 (m, 2H), 7.37 (d, *J* = 15.4 Hz, 1H), 7.28 (d, *J* = 8.5 Hz, 3H), 7.22 (d, *J* = 8.0 Hz, 1H), 7.06 (s, 1H), 7.02 (d, *J* = 8.0 Hz, 1H), 5.58 (d, *J* = 15.4 Hz, 1H), 4.17 (q, *J* = 7.1 Hz, 2H), 3.69 (s, 6H), 2.37 (s, 3H), 2.35 (s, 3H), 1.32 (t, *J* = 7.1 Hz, 3H). ^{13}C NMR (150 MHz, CDCl$_3$): δ 168.3, 168.2, 144.3, 141.7, 137.3, 135.2, 134.2, 130.0, 128.8, 127.8, 125.9, 125.1, 124.0, 102.5, 77.8, 59.0, 43.5, 19.7, 19.2, 14.4. HRMS (ESI-TOF) *m/z* calcd. for $C_{23}H_{28}NO_4S^+$ ([M+H]$^+$) 414.1734. found, 414.1739.

ethyl (*E*)-4-(dimethyl(oxo)-λ⁶-sulfaneylidene)-5-(naphthalen-2-yl(phenyl)amino)-5-oxopent-2-enoate (14)

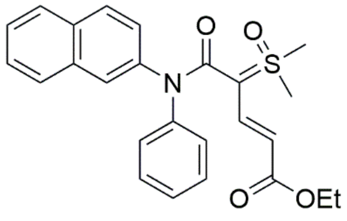

The title compound was prepared according to the general procedure and purified by column chromatography on silica gel and eluted with petroleum ether/ethyl acetate (1/2) to afford a colorless oil in 87% yield (37.8 mg). ^{1}H NMR (600 MHz, $CDCl_3$): δ 7.78 (d, *J* = 8.8 Hz, 2H), 7.71–7.66 (m, 1H), 7.50 (d, *J* = 2.0 Hz, 1H), 7.43–7.39 (m, 2H), 7.33–7.28 (m, 3H), 7.23 (d, *J* = 15.4 Hz, 1H), 7.17 (dd, *J* = 14.0, 7.4 Hz, 3H), 5.49 (d, *J* = 15.4 Hz, 1H), 3.95 (q, *J* = 7.1 Hz, 2H), 3.53 (s, 6H), 1.09 (t, *J* = 7.1 Hz, 3H). ^{13}C NMR (150 MHz, $CDCl_3$): δ 168.4, 168.1, 144.1, 141.7, 135.0, 133.5, 131.1, 129.0, 128.6, 127.5, 127.4, 126.3, 126.2, 125.6, 125.5, 125.2, 123.8, 102.9, 78.2, 59.0, 43.3, 14.3. HRMS (ESI-TOF) *m/z* calcd. for $C_{25}H_{26}NO_4S^+$ ($[M+H]^+$) 436.1577. found, 436.1572.

ethyl (*E*)-5-(di([1,1'-biphenyl]-4-yl)amino)-4-(dimethyl(oxo)-λ⁶-sulfaneylidene)-5-oxopent-2-enoate (15)

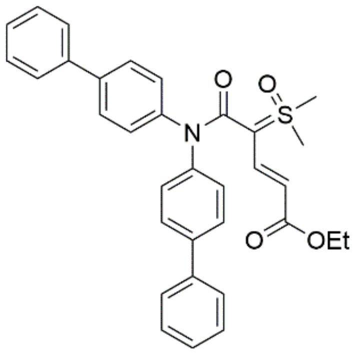

The title compound was prepared according to the general procedure and purified by column chromatography on silica gel and eluted with petroleum ether/ethyl acetate (1/1) to afford a yellow solid in 80% yield (42.9 mg). ^{1}H NMR (600 MHz, $CDCl_3$): δ 7.56 (t, *J* = 8.3 Hz, 8H), 7.43 (t, *J* = 7.6 Hz, 4H), 7.34 (t, *J* = 7.3 Hz, 2H), 7.29 (d, *J* = 15.5 Hz, 1H), 7.26–7.23 (m, 4H), 5.51 (d, *J* = 15.4 Hz, 1H), 4.00 (q, *J* = 7.1 Hz, 2H), 3.58 (s, 6H), 1.14 (t, *J* = 7.1 Hz, 3H). ^{13}C NMR (150 MHz, $CDCl_3$): δ 168.3, 168.1, 143.3, 140.2, 138.3, 134.9, 128.7, 127.7, 127.2, 126.9, 126.5, 103.3, 78.1, 59.1, 43.5, 14.4. HRMS (ESI-TOF) *m/z* calcd. for $C_{33}H_{32}NO_4S^+$ ($[M+H]^+$) 538.2047. found, 538.2044.

ethyl (*E*)-5-(5H-dibenzo[*b*,*f*]azepin-5-yl)-4-(dimethyl(oxo)-λ^6-sulfaneylidene)-5-oxopent-2-enoate (16)

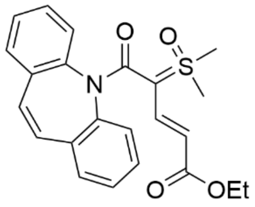

The title compound was prepared according to the general procedure and purified by column chromatography on silica gel and eluted with petroleum ether/ethyl acetate (1/2) to afford a yellow oil in 82% yield (33.5 mg). ^{1}H NMR (600 MHz, $CDCl_3$): δ 7.72 (dd, *J* = 8.0, 1.1 Hz, 2H), 7.67–7.63 (m, 2H), 7.62 (dd, *J* = 7.8, 1.4 Hz, 2H), 7.54–7.51 (m, 2H), 7.34 (s, 2H), 7.26 (d, *J* = 15.3 Hz, 1H), 5.68 (d, *J* = 15.3 Hz, 1H), 4.33 (q, *J* = 7.1 Hz, 2H), 3.77 (s, 6H), 1.51 (t, *J* = 7.1 Hz, 3H). ^{13}C NMR (150 MHz, $CDCl_3$): δ 168.1, 166.3, 141.1, 137.3, 134.3, 131.0, 129.0, 128.7, 127.9, 126.7, 101.3, 74.7, 58.9, 43.4, 14.5. HRMS (ESI-TOF) *m/z* calcd. for $C_{23}H_{24}NO_4S^+$ ([M+H]$^+$) 410.1421. found, 410.1426.

ethyl (*E*)-5-(acridin-10(9*H*)-yl)-4-(dimethyl(oxo)-λ^6-sulfaneylidene)-5-oxopent-2-enoate (17)

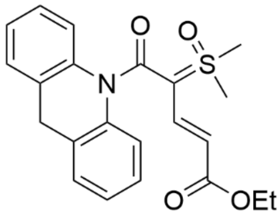

The title compound was prepared according to the general procedure and purified by column chromatography on silica gel and eluted with petroleum ether/ethyl acetate (1/2) to afford a yellow oil in 75% yield (29.7 mg). ^{1}H NMR (600 MHz, $CDCl_3$): δ 7.68 (dd, *J* = 8.1, 0.9 Hz, 2H), 7.26 (s, 1H), 7.25 (s, 1H), 7.18–7.15 (m, 2H), 7.11 (d, *J* = 13.5 Hz, 1H), 7.10–7.06 (m, 2H), 5.30 (d, *J* = 15.5 Hz, 1H), 3.98 (q, *J* = 7.1 Hz, 2H), 3.96 (s, 2H), 3.64 (s, 6H), 1.15 (t, *J* = 7.1 Hz, 3H). ^{13}C NMR (150 MHz, $CDCl_3$): δ 168.1, 166.4, 140.0, 136.3, 131.2, 127.5, 126.4, 124.7, 121.7, 102.6, 78.6, 59.2, 43.5, 33.9, 14.4. HRMS (ESI-TOF) *m/z* calcd. for $C_{22}H_{24}NO_4S^+$ ([M+H]$^+$) 398.1421. found, 398.1417.

ethyl (*E*)-4-(dimethyl(oxo)-λ^6-sulfaneylidene)-5-oxo-5-(10*H*-phenoxazin-10-yl)pent-2-enoate (18)

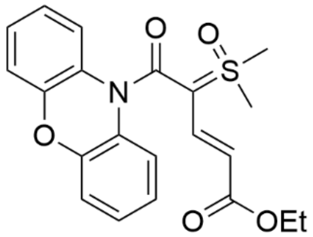

The title compound was prepared according to the general procedure and purified by column chromatography on silica gel and eluted with petroleum ether/ethyl acetate (1/2) to afford a yellow oil in 63% yield (25.1 mg). ^{1}H NMR (600 MHz, $CDCl_3$): δ 7.48–7.45 (m, 2H), 7.19 (d, *J* = 15.5 Hz, 1H), 7.01–6.97 (m, 4H), 6.95–6.90 (m, 2H), 5.44 (d, *J* = 15.5 Hz, 1H), 4.01 (q, *J* = 7.1 Hz, 2H), 3.59 (s, 6H), 1.16 (t, *J* = 7.1 Hz, 3H). ^{13}C NMR (150 MHz, $CDCl_3$): δ 168.1, 165.3, 148.9, 133.8, 130.5, 125.1, 123.5, 120.3, 116.6, 104.3, 79.7, 59.2, 43.1, 14.4. HRMS (ESI-TOF) *m/z* calcd. for $C_{21}H_{22}NO_5S^+$ ([M+H]$^+$) 400.1213. found, 400.1210.

ethyl (*E*)-4-(dimethyl(oxo)-λ^6-sulfaneylidene)-5-oxo-5-(10*H*-phenothiazin-10-yl)pent-2-enoate (19)

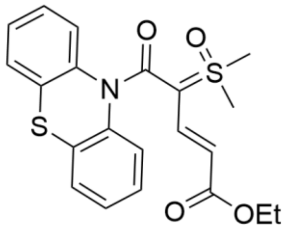

The title compound was prepared according to the general procedure and purified by column chromatography on silica gel and eluted with petroleum ether/ethyl acetate (1/2) to afford a yellow oil in 74% yield (30.7 mg). ^{1}H NMR (600 MHz, $CDCl_3$): δ 7.67 (dd, *J* = 8.1, 1.3 Hz, 2H), 7.38 (dd, *J* = 7.8, 1.5 Hz, 2H), 7.23–7.20 (m, 2H), 7.12 (td, *J* = 7.6, 1.3 Hz, 2H), 7.07 (d, *J* = 15.4 Hz, 1H), 5.51 (d, *J* = 15.5 Hz, 1H), 4.00 (q, *J* = 7.1 Hz, 2H), 3.60 (s, 6H), 1.18 (t, *J* = 7.1 Hz, 3H). ^{13}C NMR (150 MHz, $CDCl_3$): δ 168.0, 165.6, 140.1, 136.1, 132.2, 127.8, 126.9, 125.8, 125.3, 103.8, 76.2, 59.1, 43.5, 14.5. HRMS (ESI-TOF) *m/z* calcd. for $C_{21}H_{22}NO_4S_2^+$ ([M+H]$^+$) 416.0985. found, 416.0981.

ethyl (*E*)-5-(2-chloro-10*H*-phenothiazin-10-yl)-4-(dimethyl(oxo)-λ^6-sulfaneylidene)-5-oxopent-2-enoate (20)

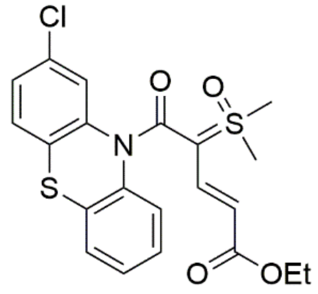

The title compound was prepared according to the general procedure and purified by column chromatography on silica gel and eluted with dichloromethane/ethyl acetate (2/1) to afford a yellow oil in 82% yield (36.8 mg). ^{1}H NMR (600 MHz, $CDCl_3$): δ 7.85 (d, *J* = 2.1 Hz, 1H), 7.50 (d, *J* = 8.0 Hz, 1H), 7.34 (d, *J* = 7.7 Hz, 1H), 7.26 (t, *J* = 4.2 Hz, 1H), 7.18 (t, *J* = 7.2 Hz, 1H), 7.12–7.07 (m, 3H), 5.55 (d, *J* = 15.5 Hz, 1H), 4.00 (q, *J* = 7.1 Hz, 2H), 3.59 (s, 6H), 1.18 (t, *J* = 7.1 Hz, 3H). ^{13}C NMR (150 MHz, $CDCl_3$): δ 167.9, 165.4, 141.0, 140.1, 135. 8, 132.4, 131.5, 130.2, 128.2, 127.8, 127.2, 125.9, 125.7, 125.3, 124.8, 104.0, 76.7, 59.1, 43.2, 14.4. HRMS (ESI-TOF) *m/z* calcd. for $C_{21}H_{21}ClNO_4S_2^+$ ([M+H]$^+$) 450.0595. found, 450.0593.

ethyl (*E*)-5-(9*H*-carbazol-9-yl)-4-(dimethyl(oxo)-λ^6-sulfaneylidene)-5-oxopent-2-enoate (21)

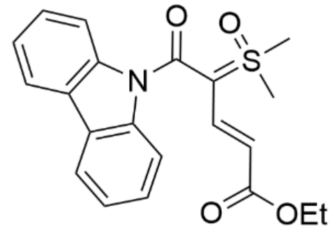

The title compound was prepared according to the general procedure and purified by column chromatography on silica gel and eluted with petroleum ether/ethyl acetate (1/2) to afford a colorless oil in 60% yield (23.0 mg). ^{1}H NMR (600 MHz, $CDCl_3$): δ 8.02 (d, *J* = 7.7 Hz, 2H), 7.74 (d, *J* = 8.3 Hz, 2H), 7.46 (d, *J* = 15.7 Hz, 1H), 7.42 (t, *J* = 7.5 Hz, 2H), 7.31 (t, *J* = 7.4 Hz, 2H), 5.19 (d, *J* = 15.7 Hz, 1H), 3.98 (q, *J* = 7.1 Hz, 2H), 3.65 (s, 6H), 1.09 (t, *J* = 7.1 Hz, 3H). ^{13}C NMR (150 MHz, $CDCl_3$): δ 168.2, 164.1, 138.0, 134.7, 126.5, 125.3, 122.2, 119.9, 114.3, 105.4, 82.3, 59.5, 43.1, 14.2. HRMS (ESI-TOF) *m/z* calcd. for $C_{21}H_{22}NO_4S^+$ ([M+H]$^+$) 384.1264. found, 384.1259.

ethyl (*E*)-5-(3,4-dihydroquinolin-1(2*H*)-yl)-4-(dimethyl(oxo)-λ^6-sulfaneylidene)-5-oxopent-2-enoate (22)

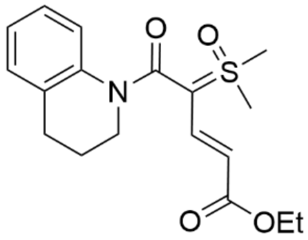

The title compound was prepared according to the general procedure and purified by column chromatography on silica gel and eluted with petroleum ether/ethyl acetate (1/2) to afford a yellow oil in 52% yield (18.1 mg). ^{1}H NMR (600 MHz, $CDCl_3$): δ 7.48 (d, *J* = 15.2 Hz, 1H), 7.28 (d, *J* = 8.0 Hz, 1H), 7.10 (d, *J* = 7.5 Hz, 1H), 7.07 (t, *J* = 7.5 Hz, 1H), 6.97 (t, *J* = 7.3 Hz, 1H), 5.30 (d, *J* = 15.2 Hz, 1H), 4.09 (q, *J* = 7.1 Hz, 2H), 3.73–3.67 (m, 2H), 3.48 (s, 6H), 2.83 (t, *J* = 6.7 Hz, 2H), 1.93–1.89 (m, 2H), 1.21 (t, *J* = 7.1 Hz, 3H). ^{13}C NMR (150 MHz, $CDCl_3$): δ 168.8, 168.4, 138.3, 135.3, 129.4, 128.7, 125.6, 123.7, 123.6, 101.8, 78.0, 59.2, 47.1, 43.7, 26.6, 23.8, 14.4. HRMS (ESI-TOF) *m/z* calcd. for $C_{18}H_{24}NO_4S^+$ ([M+H]$^+$) 350.1421. found, 350.1420.

ethyl (*E*)-5-(7-bromo-2,3-dihydro-4*H*-benzo[*b*][1,4]oxazin-4-yl)-4-(dimethyl(oxo)-λ^6-sulfaneylidene)-5-oxopent-2-enoate (23)

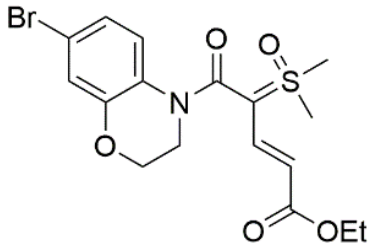

The title compound was prepared according to the general procedure and purified by column chromatography on silica gel and eluted with petroleum ether/ethyl acetate (1/1) to afford a white solid in 81% yield (34.7 mg). ^{1}H NMR (600 MHz, $CDCl_3$): δ 7.48 (d, *J* = 15.3 Hz, 1H), 7.22 (d, *J* = 8.8 Hz, 1H), 7.02 (d, *J* = 2.2 Hz, 1H), 6.93 (dd, *J* = 8.8, 2.2 Hz, 1H), 5.34 (d, *J* = 15.3 Hz, 1H), 4.21–4.14 (m, 2H), 4.10 (q, *J* = 7.1 Hz, 2H), 3.80–3.71 (m, 2H), 3.48 (s, 6H), 1.21 (t, *J* = 7.1 Hz, 3H). ^{13}C NMR (150 MHz, $CDCl_3$): δ 168.5, 167.0, 146.9, 134.9, 124.7, 124.4, 123.2, 120.2, 116.7, 103.0, 73.30, 65.6, 59.4, 45.05, 43.4, 14.4. HRMS (ESI-TOF) *m/z* calcd. for $C_{18}H_{23}BrNO_4S^+$ ([M+H]$^+$) 428.0526. found, 428.0521.

ethyl (*E*)-4-(dimethyl(oxo)-λ⁶-sulfaneylidene)-5-oxo-5-(5-oxo-2,3,4,5-tetrahydro-1*H*-benzo[*b*]azepin-1-yl)pent-2-enoate (24)

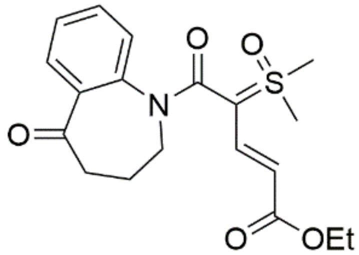

The title compound was prepared according to the general procedure and purified by column chromatography on silica gel and eluted with petroleum ether/ethyl acetate (1/2) to afford a colorless oil in 76% yield (28.7 mg). ^{1}H NMR (600 MHz, $CDCl_3$): δ 7.83 (d, *J* = 9.1 Hz, 1H), 7.43 (t, *J* = 6.9 Hz, 1H), 7.29 (d, *J* = 15.4 Hz, 1H), 7.29 (t, *J* = 6.6 Hz, 1H), 7.20 (d, *J* = 7.9 Hz, 1H), 5.29 (d, *J* = 15.3 Hz, 1H), 4.03 (q, *J* = 7.1 Hz, 2H), 3.84 (t, *J* = 6.5 Hz, 2H), 3.48 (s, 6H), 2.76–2.68 (m, 2H), 2.01 (p, *J* = 6.6 Hz, 2H), 1.16 (t, *J* = 7.1 Hz, 3H). ^{13}C NMR (150 MHz, $CDCl_3$): δ 201.2, 168.3, 168.2, 143.9, 135.9, 134.1, 133.1, 129.3, 127.2, 126.5, 101.7, 76.9, 59.3, 48.7, 43.5, 39.8, 24.5, 14.4. HRMS (ESI-TOF) *m/z* calcd. for $C_{19}H_{24}NO_5S^+$ ($[M+H]^+$) 378.1370. found, 378.1385.

ethyl (*E)*-4-(dimethyl(oxo)-λ⁶-sulfaneylidene)-5-oxo-5-(4-phenyl-3,6-dihydropyridin-1(2*H*)-yl)pent-2-enoate (25)

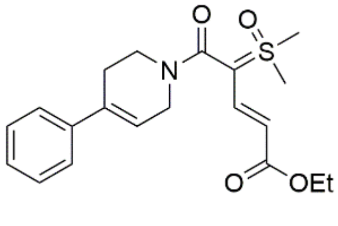

The title compound was prepared according to the general procedure and purified by column chromatography on silica gel and eluted with petroleum ether/ethyl acetate (1/2) to afford a yellow oil in 62% yield (23.2 mg). ^{1}H NMR (600 MHz, $CDCl_3$): δ 7.57 (d, *J* = 15.0 Hz, 1H), 7.38 (d, *J* = 7.2 Hz, 2H), 7.33 (t, *J* = 7.7 Hz, 2H), 7.25 (d, *J* = 7.6 Hz, 1H), 6.08 (s, 1H), 5.21 (d, *J* = 15.0 Hz, 1H), 4.16 (q, *J* = 7.1 Hz, 2H), 4.14–4.09 (m, 2H), 3.68 (t, *J* = 5.6 Hz, 2H), 3.44 (s, 6H), 2.61 (s, 2H), 1.27 (t, *J* = 7.1 Hz, 3H). ^{13}C NMR (150 MHz, $CDCl_3$): δ 169.0, 167.7, 140.2, 136.3, 135.4, 128.4, 127.4, 124.9, 121.0, 100.9, 76.2, 59.3, 43.9, 40.9, 27.5, 14.6. HRMS (ESI-TOF) *m/z* calcd. for $C_{20}H_{26}NO_4S^+$ ($[M+H]^+$) 376.1577. found, 376.1571.

ethyl (*E*)-4-(dimethyl(oxo)-λ⁶-sulfaneylidene)-5-(methyl(phenyl)amino)-5-oxopent-2-enoate (26)

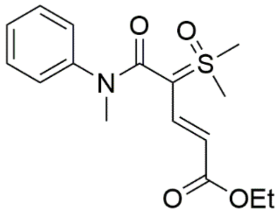

The title compound was prepared according to the general procedure and purified by column chromatography on silica gel and eluted with petroleum ether/ethyl acetate (1/1) to afford a yellow oil in 75% yield (24.2 mg). ^{1}H NMR (600 MHz, $CDCl_3$): δ 7.31–7.27 (m, 2H), 7.25 (d, *J* = 13.1 Hz, 1H), 7.20 (d, *J* = 7.4 Hz, 2H), 7.11 (t, *J* = 7.4 Hz, 1H), 5.21 (d, *J* = 15.2 Hz, 1H), 4.02 (q, *J* = 7.1 Hz, 2H), 3.48 (s, 6H), 3.30 (s, 3H), 1.17 (t, *J* = 7.1 Hz, 3H). ^{13}C NMR (150 MHz, $CDCl_3$): δ 168.5, 168.4, 144.4, 135.8, 128.9, 125.5, 124.9, 101.5, 76.7, 59.0, 43.6, 38.5, 14.4. HRMS (ESI-TOF) *m/z* calcd. for $C_{16}H_{22}NO_4S^+$ ($[M+H]^+$) 324.1264. found, 324.1271.

ethyl (*E*)-5-(allyl(phenyl)amino)-4-(dimethyl(oxo)-λ⁶-sulfaneylidene)-5-oxopent-2-enoate (27)

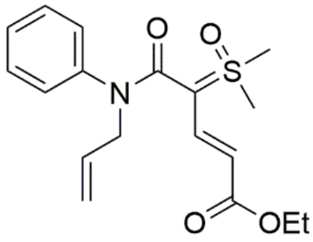

The title compound was prepared according to the general procedure and purified by column chromatography on silica gel and eluted with petroleum ether/ethyl acetate (1/1) to afford a yellow oil in 65% yield (22.7 mg). ^{1}H NMR (600 MHz, $CDCl_3$): δ 7.35 (q, *J* = 6.9, 5.9 Hz, 2H), 7.30–7.25 (m, 3H), 7.18 (t, *J* = 7.4 Hz, 1H), 6.00–5.94 (m, 1H), 5.34 (d, *J* = 15.3 Hz, 1H), 5.23–5.15 (m, 2H), 4.42 (d, *J* = 5.5 Hz, 2H), 4.10 (q, *J* = 7.1 Hz, 2H), 3.57 (s, 6H), 1.24 (t, *J* = 7.1 Hz, 3H). ^{13}C NMR (150 MHz, $CDCl_3$): δ 168.5, 168.0, 143.5, 135.9, 134.4, 128.9, 125.6, 125.5, 116.8, 101.4, 76.7, 59.1, 53.2, 43.8, 14.5. HRMS (ESI-TOF) *m/z* calcd. for $C_{18}H_{24}NO_4S^+$ ($[M+H]^+$) 350.1421. found, 350.1418.

ethyl (*E*)-5-((2-cyanoethyl)(phenyl)amino)-4-(dimethyl(oxo)-λ^6-sulfaneylidene)-5-oxopent-2-enoate (28)

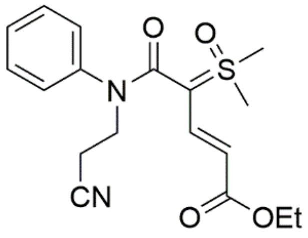

The title compound was prepared according to the general procedure and purified by column chromatography on silica gel and eluted with petroleum ether/ethyl acetate (1/1) to afford a yellow oil in 78% yield (28.2 mg). ^{1}H NMR (600 MHz, $CDCl_3$): δ 7.31 (t, *J* = 7.8 Hz, 2H), 7.22 (d, *J* = 7.5 Hz, 2H), 7.17 (t, *J* = 7.4 Hz, 1H), 7.08 (d, *J* = 15.3 Hz, 1H), 5.17 (d, *J* = 15.3 Hz, 1H), 3.97 (p, *J* = 6.8 Hz, 4H), 3.50 (s, 6H), 2.52 (t, *J* = 6.6 Hz, 2H), 1.13 (t, *J* = 7.1 Hz, 3H). ^{13}C NMR (150 MHz, $CDCl_3$): δ 168.1, 167.6, 141.4, 135.5, 129.3, 126.7, 126.5, 118.0, 101.8, 76.3, 59.0, 45.9, 43.5, 16.8, 14.4. HRMS (ESI-TOF) *m/z* calcd. for $C_{18}H_{23}N_2O_4S^+$ ([M+H]$^+$) 363.1373. found, 363.1371.

ethyl (*E*)-4-(dimethyl(oxo)-λ^6-sulfaneylidene)-5-oxo-5-((thiophen-2-ylmethyl)(p-tolyl)amino)pent-2-enoate (29)

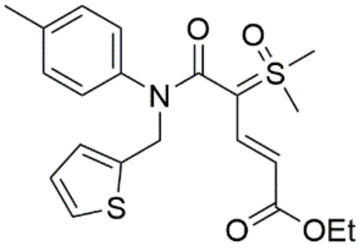

The title compound was prepared according to the general procedure and purified by column chromatography on silica gel and eluted with petroleum ether/ethyl acetate (1/1) to afford a yellow oil in 70% yield (29.3 mg). ^{1}H NMR (600 MHz, $CDCl_3$): δ 7.18 (d, *J* = 15.3 Hz, 1H), 7.14 (d, *J* = 5.1 Hz, 1H), 7.03 (d, *J* = 8.1 Hz, 2H), 6.97 (d, *J* = 8.4 Hz, 2H), 6.80 (dd, *J* = 5.1, 3.4 Hz, 1H), 6.69 (d, *J* = 3.4 Hz, 1H), 5.28 (d, *J* = 15.3 Hz, 1H), 4.99 (s, 2H), 4.02 (q, *J* = 7.1 Hz, 2H), 3.50 (s, 6H), 2.25 (s, 3H), 1.17 (t, *J* = 7.1 Hz, 3H). ^{13}C NMR (150 MHz, $CDCl_3$): δ 168.4, 167.8, 140.8, 140.1, 135.9, 135.8, 129.5, 126.7, 126.2, 126.1, 125.4, 101.4, 76.4, 59.0, 49.4, 43.8, 20.9, 14.5. HRMS (ESI-TOF) *m/z* calcd. for $C_{21}H_{26}NO_4S_2^+$ ([M+H]$^+$) 420.1298. found, 420.1395.

ethyl (*E*)-4-(dimethyl(oxo)-λ^6-sulfaneylidene)-5-oxo-5-(phenylamino)pent-2-enoate (30)

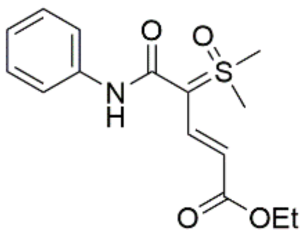

The title compound was prepared according to the general procedure and purified by column chromatography on silica gel and eluted with petroleum ether/ethyl acetate (1/1) to afford a yellow oil in 72% yield (22.2 mg). ^{1}H NMR (600 MHz, $CDCl_3$): δ 7.70 (d, J = 15.5 Hz, 1H), 7.39 (d, J = 8.4 Hz, 3H), 7.33–7.28 (m, 2H), 7.07 (t, J = 7.4 Hz, 1H), 5.53 (d, J = 15.5 Hz, 1H), 4.17 (q, J = 7.1 Hz, 2H), 3.53 (s, 6H), 1.27 (t, J = 7.1 Hz, 3H). ^{13}C NMR (150 MHz, $CDCl_3$): δ 168.9, 163.7, 137.8, 136.6, 128.9, 123.8, 120.4, 99.6, 76.1, 59.6, 44.3, 14.5. HRMS (ESI-TOF) *m/z* calcd. for $C_{15}H_{20}NO_4S^+$ ([M+H]$^+$) 310.1108. found, 310.1105.

ethyl (*E*)-5-((4-(tert-butyl)phenyl)amino)-4-(dimethyl(oxo)-λ^6-sulfaneylidene)-5-oxopent-2-enoate (31)

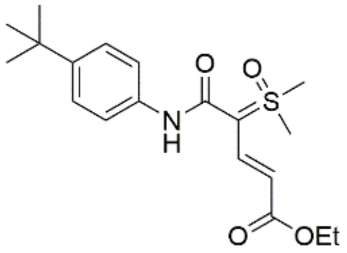

The title compound was prepared according to the general procedure and purified by column chromatography on silica gel and eluted with petroleum ether/ethyl acetate (2/1) to afford a yellow solid in 71% yield (25.9 mg). ^{1}H NMR (600 MHz, $CDCl_3$): δ 7.70 (d, J = 15.5 Hz, 1H), 7.35 (s, 1H), 7.34–7.28 (m, 4H), 5.52 (d, J = 15.5 Hz, 1H), 4.17 (q, J = 7.1 Hz, 2H), 3.53 (s, 6H), 1.29 (s, 9H), 1.27 (t, J = 7.1 Hz, 3H). ^{13}C NMR (150 MHz, $CDCl_3$): δ 168.9, 163.8, 146.9, 136.7, 135.0, 125.8, 120.4, 99.4, 76.0, 59.5, 44.3, 34.3, 31.3, 14.5. HRMS (ESI-TOF) *m/z* calcd. for $C_{19}H_{28}NO_4S^+$ ([M+H]$^+$) 366.1734. found, 366.1730.

ethyl (*E*)-4-(dimethyl(oxo)-λ⁶-sulfaneylidene)-5-((4-methoxyphenyl)amino)-5-oxopent-2-enoate (32)

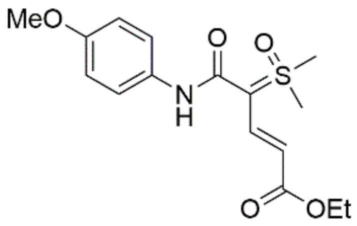

The title compound was prepared according to the general procedure and purified by column chromatography on silica gel and eluted with petroleum ether/ethyl acetate (1/1) to afford a yellow oil in 90% yield (30.5 mg). ^{1}H NMR (600 MHz, $CDCl_3$): δ 7.67 (d, *J* = 15.5 Hz, 1H), 7.32 (s, 1H), 7.28 (d, *J* = 9.0 Hz, 2H), 6.84 (d, *J* = 9.0 Hz, 2H), 5.49 (d, *J* = 15.5 Hz, 1H), 4.15 (q, *J* = 7.1 Hz, 2H), 3.76 (s, 3H), 3.51 (s, 6H), 1.25 (t, *J* = 7.1 Hz, 3H). ^{13}C NMR (150 MHz, $CDCl_3$): δ 168.9, 163.8, 156.2, 136.7, 130.6, 122.6, 114.1, 99.1, 75.9, 59.5, 55.4, 44.2, 14.5. HRMS (ESI-TOF) *m/z* calcd. for $C_{16}H_{22}NO_5S^+$ ($[M+H]^+$) 340.1213. found, 340.1210.

ethyl (*E*)-5-((3-chlorophenyl)amino)-4-(dimethyl(oxo)-λ⁶-sulfaneylidene)-5-oxopent-2-enoate (33)

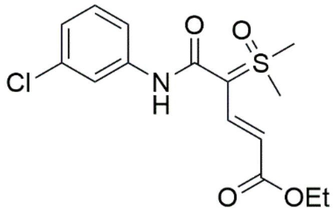

The title compound was prepared according to the general procedure and purified by column chromatography on silica gel and eluted with petroleum ether/ethyl acetate (1/1) to afford a white solid in 91% yield (31.2 mg). ^{1}H NMR (600 MHz, $CDCl_3$): δ 8.17 (dd, *J* = 8.3, 1.5 Hz, 1H), 7.93 (s, 1H), 7.72 (d, *J* = 15.7 Hz, 1H), 7.39 (dd, *J* = 8.0, 1.4 Hz, 1H), 7.27–7.22 (m, 1H), 7.02–6.99 (m, 1H), 5.75 (d, *J* = 15.7 Hz, 1H), 4.19 (q, *J* = 7.1 Hz, 2H), 3.57 (s, 6H), 1.29 (t, *J* = 7.1 Hz, 3H). ^{13}C NMR (150 MHz, $CDCl_3$): δ 168.8, 163.4, 135.8, 135.1, 129.2, 127.3, 123.9, 123.0, 121.5, 100.7, 76.6, 59.6, 44.2, 14.5. HRMS (ESI-TOF) *m/z* calcd. for $C_{15}H_{19}ClNO_4S^+$ ($[M+H]^+$) 344.0718. found, 344.0715.

ethyl (*E*)-5-((2-chlorophenyl)amino)-4-(dimethyl(oxo)-λ^6-sulfaneylidene)-5-oxopent-2-enoate (34)

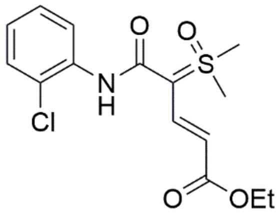

The title compound was prepared according to the general procedure and purified by column chromatography on silica gel and eluted with petroleum ether/ethyl acetate (1/1) to afford a white solid in 92% yield (31.6 mg). ^{1}H NMR (600 MHz, $CDCl_3$): δ 7.70 (d, *J* = 15.5 Hz, 1H), 7.63 (d, *J* = 28.8 Hz, 2H), 7.32–7.26 (m, 2H), 7.09 (d, *J* = 7.6 Hz, 1H), 5.57 (d, *J* = 15.5 Hz, 1H), 4.23 (q, *J* = 7.1 Hz, 2H), 3.60 (s, 6H), 1.33 (t, *J* = 7.1 Hz, 3H). ^{13}C NMR (150 MHz, $CDCl_3$): δ 168.9, 163.6, 139.3, 136.5, 134.5, 129.8, 123.6, 120.1, 118.0, 100.2, 76.1, 59.7, 44.1, 14.5. HRMS (ESI-TOF) *m/z* calcd. for $C_{15}H_{19}ClNO_4S^+$ ($[M+H]^+$) 344.0718. found, 344.0715.

ethyl (*E*)-4-(dimethyl(oxo)-λ^6-sulfaneylidene)-5-((2-iodophenyl)amino)-5-oxopent-2-enoate (35)

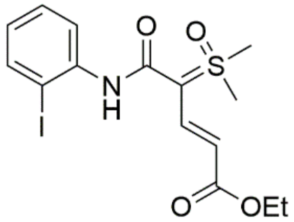

The title compound was prepared according to the general procedure and purified by column chromatography on silica gel and eluted with petroleum ether/ethyl acetate (1/1) to afford a white solid in 95% yield (41.3 mg). ^{1}H NMR (600 MHz, $CDCl_3$): δ 7.95 (dd, *J* = 8.2, 1.5 Hz, 1H), 7.78 (dd, *J* = 8.0, 1.4 Hz, 1H), 7.73–7.67 (m, 2H), 7.32–7.27 (m, 1H), 6.82–6.77 (m, 1H), 5.86 (d, *J* = 15.7 Hz, 1H), 4.16 (q, *J* = 7.1 Hz, 2H), 3.54 (s, 6H), 1.26 (t, *J* = 7.1 Hz, 3H). ^{13}C NMR (150 MHz, $CDCl_3$): δ 168.7, 163.5, 139.0, 138.9, 135.7, 128.7, 125.4, 122.4, 101.3, 90.6, 76.3, 59.5, 44.2, 14.5. HRMS (ESI-TOF) *m/z* calcd. for $C_{15}H_{19}INO_4S^+$ ($[M+H]^+$) 436.0074. found, 436.0081.

ethyl (*E*)-4-(dimethyl(oxo)-λ^6-sulfaneylidene)-5-oxo-5-((4-(trifluoromethoxy)phenyl)amino)pent-2-enoate (36)

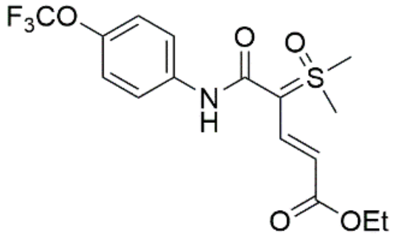

The title compound was prepared according to the general procedure and purified by column chromatography on silica gel and eluted with petroleum ether/ethyl acetate (1/2) to afford a white solid in 92% yield (36.2 mg). ^{1}H NMR (600 MHz, $CDCl_3$): δ 7.65 (d, J = 15.5 Hz, 1H), 7.61 (s, 1H), 7.46 (d, J = 9.0 Hz, 2H), 7.15 (d, J = 8.6 Hz, 2H), 5.52 (d, J = 15.5 Hz, 1H), 4.16 (q, J = 7.1 Hz, 2H), 3.53 (s, 6H), 1.26 (t, J = 7.1 Hz, 3H). ^{13}C NMR (150 MHz, $CDCl_3$): δ 169.0, 163.8, 144.8, 136.8, 136.5, 121.7, 121.4, 120.4 (q, J = 256.7 Hz), 100.2, 76.0, 59.6, 44.1, 14.5. ^{19}F NMR (564 MHz, $CDCl_3$): δ -58.13 (s, 3F). HRMS (ESI-TOF) *m/z* calcd. for $C_{16}H_{19}F_3NO_5S^+$ ([M+H]$^+$) 394.0931. found, 394.0928.

ethyl (*E*)-4-(dimethyl(oxo)-λ^6-sulfaneylidene)-5-oxo-5-((4-vinylphenyl)amino)pent-2-enoate (37)

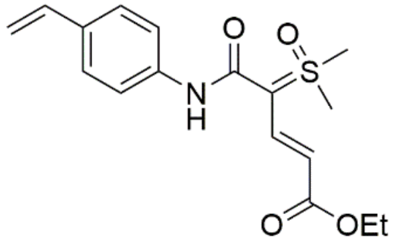

The title compound was prepared according to the general procedure and purified by column chromatography on silica gel and eluted with petroleum ether/ethyl acetate (2/1) to afford a yellow oil in 85% yield (28.5 mg). ^{1}H NMR (600 MHz, $CDCl_3$): δ 7.67 (d, J = 15.5 Hz, 1H), 7.48 (s, 1H), 7.39–7.33 (m, 4H), 6.65 (dd, J = 17.6, 10.9 Hz, 1H), 5.65 (d, J = 17.6 Hz, 1H), 5.52 (d, J = 15.5 Hz, 1H), 5.16 (d, J = 10.9 Hz, 1H), 4.16 (q, J = 7.1 Hz, 2H), 3.52 (s, 6H), 1.26 (t, J = 7.1 Hz, 3H). ^{13}C NMR (150 MHz, $CDCl_3$): δ 168.9, 163.6, 137.5, 136.6, 136.1, 133.1, 126.7, 120.1, 112.7, 99.6, 76.2, 59.6, 44.1, 14.5. HRMS (ESI-TOF) *m/z* calcd. for $C_{17}H_{22}NO_4S^+$ ([M+H]$^+$) 336.1264. found, 336.1259.

ethyl (*E*)-4-(dimethyl(oxo)-λ^6-sulfaneylidene)-5-oxo-5-((2-(phenylethynyl)phenyl)amino)pent-2-enoate (38)

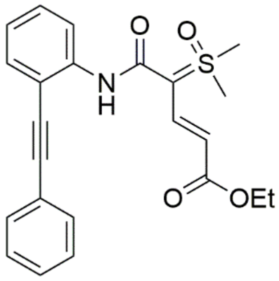

The title compound was prepared according to the general procedure and purified by column chromatography on silica gel and eluted with petroleum ether/ethyl acetate (1/1) to afford a white solid in 88% yield (36.0 mg). ^{1}H NMR (600 MHz, CDCl$_3$): δ 8.37 (s, 1H), 8.18 (d, *J* = 8.3 Hz, 1H), 7.72 (d, *J* = 15.6 Hz, 1H), 7.51–7.47 (m, 3H), 7.32–7.27 (m, 4H), 7.00 (t, *J* = 7.5 Hz, 1H), 5.80 (d, *J* = 15.6 Hz, 1H), 3.91 (q, *J* = 7.1 Hz, 2H), 3.53 (s, 6H), 1.01 (t, *J* = 7.1 Hz, 3H). ^{13}C NMR (150 MHz, CDCl$_3$): δ 168.8, 163.4, 139.4, 136.0, 131.9, 131.6, 129.2, 128.4, 128.1, 122.5, 122.2, 119.0, 111.8, 100.0, 96.3, 84.6, 76.9, 59.2, 43.9, 14.1. HRMS (ESI-TOF) *m/z* calcd. for $C_{23}H_{24}NO_4S^+$ ([M+H]$^+$) 410.1421. found, 410.1417.

ethyl (*E*)-4-(dimethyl(oxo)-λ^6-sulfaneylidene)-5-((2,6-dimethylphenyl)amino)-5-oxopent-2-enoate (39)

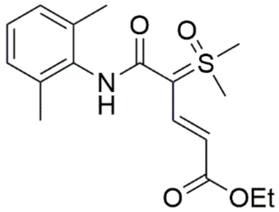

The title compound was prepared according to the general procedure and purified by column chromatography on silica gel and eluted with petroleum ether/ethyl acetate (1/1) to afford a yellow oil in 83% yield (27.9 mg). ^{1}H NMR (600 MHz, CDCl$_3$): δ 7.77 (d, *J* = 15.6 Hz, 1H), 7.08 (s, 3H), 6.90 (s, 1H), 5.56 (d, *J* = 15.5 Hz, 1H), 4.17 (q, *J* = 7.1 Hz, 2H), 3.50 (s, 6H), 2.23 (s, 6H), 1.27 (t, *J* = 7.1 Hz, 3H). ^{13}C NMR (150 MHz, CDCl$_3$): δ 168.9, 164.0, 137.0, 135.3, 133.9, 128.1, 127.1, 99.3, 75.8, 59.5, 44.4, 18.6, 14.5. HRMS (ESI-TOF) *m/z* calcd. for $C_{17}H_{24}NO_4S^+$ ([M+H]$^+$) 338.1421. found, 338.1426.

ethyl (*E*)-4-(dimethyl(oxo)-λ^6-sulfaneylidene)-5-oxo-5-((3,4,5-trimethylphenyl)amino)pent-2-enoate (40)

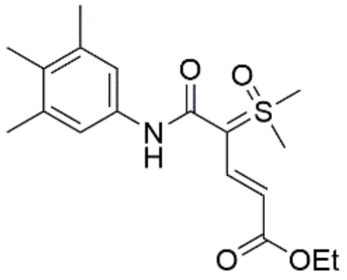

The title compound was prepared according to the general procedure and purified by column chromatography on silica gel and eluted with petroleum ether/ethyl acetate (1/1) to afford a yellow solid in 93% yield (32.6 mg). ^{1}H NMR (600 MHz, $CDCl_3$): δ 7.68 (d, J = 15.5 Hz, 1H), 7.27 (s, 1H), 7.04 (s, 2H), 5.49 (d, J = 15.5 Hz, 1H), 4.16 (q, J = 6.8 Hz, 2H), 3.52 (s, 6H), 2.25 (s, 6H), 2.10 (s, 3H), 1.26 (t, J = 6.9 Hz, 3H). ^{13}C NMR (150 MHz, $CDCl_3$): δ 168.9, 163.6, 136.9, 136.7, 134.6, 130.7, 119.7, 99.0, 76.1, 59.4, 44.2, 20.6, 14.8, 14.6. HRMS (ESI-TOF) *m/z* calcd. for $C_{18}H_{26}NO_4S^+$ ([M+H]$^+$) 352.1577. found, 352.1583.

ethyl (*E*)-4-(dimethyl(oxo)-λ^6-sulfaneylidene)-5-(methyl(phenethyl)amino)-5-oxopent-2-enoate (41)

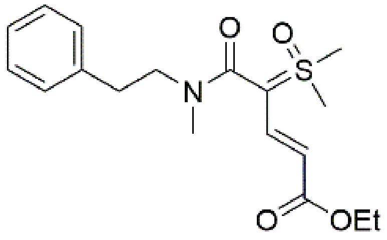

The title compound was prepared according to the general procedure and purified by column chromatography on silica gel and eluted with petroleum ether/ethyl acetate (1/1) to afford a colorless oil in 57% yield (20.0 mg). ^{1}H NMR (600 MHz, $CDCl_3$): δ 7.48 (d, J = 14.9 Hz, 1H), 7.29–7.24 (m, 2H), 7.19 (d, J = 7.3 Hz, 3H), 5.00 (d, J = 14.9 Hz, 1H), 4.16 (q, J = 7.1 Hz, 2H), 3.64 (t, J = 6.7 Hz, 2H), 3.09 (s, 6H), 2.99 (s, 3H), 2.87 (t, J = 6.7 Hz, 2H), 1.28 (t, J = 7.1 Hz, 3H). ^{13}C NMR (150 MHz, $CDCl_3$): δ 169.0, 168.0, 138.9, 136.2, 129.2, 128.5, 126.4, 100.0, 76.3, 59.2, 51.5, 43.2, 34.0, 29.7, 14.6. HRMS (ESI-TOF) *m/z* calcd. for $C_{18}H_{26}NO_4S^+$ ([M+H]$^+$) 352.1577. found, 352.1571.

ethyl (*E*)-5-(benzylamino)-4-(dimethyl(oxo)-λ⁶-sulfaneylidene)-5-oxopent-2-enoate (42)

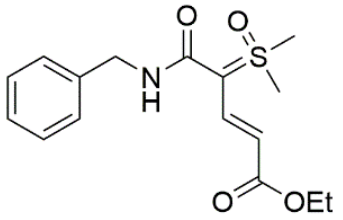

The title compound was prepared according to the general procedure and purified by column chromatography on silica gel and eluted with petroleum ether/ethyl acetate (1/1) to afford a yellow oil in 77% yield (24.9 mg). ^{1}H NMR (600 MHz, $CDCl_3$): δ 7.67 (d, *J* = 15.5 Hz, 1H), 7.37–7.32 (m, 2H), 7.29 (dd, *J* = 15.4, 7.1 Hz, 3H), 6.05 (s, 1H), 5.38 (d, *J* = 15.5 Hz, 1H), 4.51 (d, *J* = 5.9 Hz, 2H), 4.15 (q, *J* = 7.1 Hz, 2H), 3.51 (s, 6H), 1.26 (t, *J* = 7.1 Hz, 3H). ^{13}C NMR (150 MHz, $CDCl_3$): δ 169.0, 165.4, 138.7, 136.8, 128.6, 127.23, 127.22, 98.4, 75.4, 59.4, 44.5, 43.1, 14.5. HRMS (ESI-TOF) *m/z* calcd. for $C_{16}H_{22}NO_4S^+$ ($[M+H]^+$) 324.1264. found, 324.1267.

ethyl (*E*)-4-(dimethyl(oxo)-λ⁶-sulfaneylidene)-5-oxo-5-(thiophen-3-ylamino)pent-2-enoate (43)

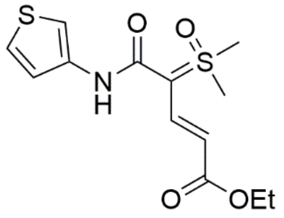

The title compound was prepared according to the general procedure and purified by column chromatography on silica gel and eluted with petroleum ether/ethyl acetate (1/1) to afford a yellow oil in 96% yield (30.2 mg). ^{1}H NMR (600 MHz, $CDCl_3$): δ 7.84 (s, 1H), 7.67 (d, *J* = 15.5 Hz, 1H), 7.34 (s, 1H), 7.26–7.22 (m, 1H), 7.09 (d, *J* = 4.9 Hz, 1H), 5.52 (d, *J* = 15.5 Hz, 1H), 4.18 (q, *J* = 7.1 Hz, 2H), 3.55 (s, 6H), 1.29 (t, *J* = 7.1 Hz, 3H). ^{13}C NMR (150 MHz, $CDCl_3$): δ 169.0, 163.1, 136.6, 135.7, 124.4, 121.7, 109.2, 99.4, 75.7, 59.6, 44.2, 14.5. HRMS (ESI-TOF) *m/z* calcd. for $C_{13}H_{18}NO_4S_2^+$ ($[M+H]^+$) 316.0672. found, 316.0669.

ethyl (*E*)-5-(dibenzo[*b,d*]furan-4-ylamino)-4-(dimethyl(oxo)-λ^6-sulfaneylidene)-5-oxopent-2-enoate (44)

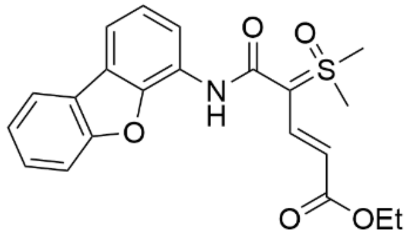

The title compound was prepared according to the general procedure and purified by column chromatography on silica gel and eluted with petroleum ether/ethyl acetate (1/1) to afford a yellow solid in 86% yield (34.3 mg). ^{1}H NMR (600 MHz, $CDCl_3$): δ 8.04 (d, *J* = 7.9 Hz, 1H), 7.96 (s, 1H), 7.92 (d, *J* = 7.6 Hz, 1H), 7.79 (d, *J* = 15.7 Hz, 1H), 7.63 (d, *J* = 7.7 Hz, 1H), 7.59 (d, *J* = 8.2 Hz, 1H), 7.45 (t, *J* = 7.8 Hz, 1H), 7.34 (t, *J* = 7.2 Hz, 1H), 7.30 (t, *J* = 7.8 Hz, 1H), 5.85 (d, *J* = 15.7 Hz, 1H), 4.24 (q, *J* = 7.1 Hz, 2H), 3.58 (s, 6H), 1.32 (t, *J* = 7.1 Hz, 3H). ^{13}C NMR (150 MHz, $CDCl_3$): δ 168.9, 163.6, 155.7, 146.1, 136.2, 127.2, 124.5, 124.4, 123.7, 123.1, 123.0, 120.8, 118.2, 115.4, 111.8, 100.4, 76.4, 59.6, 44.2, 14.6. HRMS (ESI-TOF) *m/z* calcd. for $C_{21}H_{22}NO_5S^+$ ($[M+H]^+$) 400.1213. found, 400.1219.

ethyl (*E*)-4-(dimethyl(oxo)-λ^6-sulfaneylidene)-5-((1-methyl-1*H*-indol-5-yl)amino)-5-oxopent-2-enoate (45)

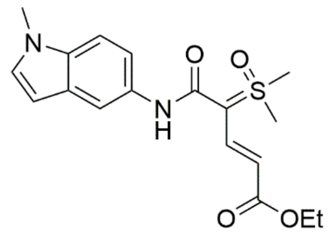

The title compound was prepared according to the general procedure and purified by column chromatography on silica gel and eluted with petroleum ether/ethyl acetate (1/1) to afford a colorless oil in 83% yield (30.0 mg). ^{1}H NMR (600 MHz, $CDCl_3$): δ 7.76 (d, *J* = 15.5 Hz, 1H), 7.67 (s, 1H), 7.42 (s, 1H), 7.29–7.24 (m, 1H), 7.18 (d, *J* = 7.1 Hz, 1H), 7.04 (d, *J* = 2.9 Hz, 1H), 6.43 (s, 1H), 5.58 (d, *J* = 15.5 Hz, 1H), 4.20 (q, *J* = 7.1 Hz, 2H), 3.77 (s, 3H), 3.54 (s, 6H), 1.30 (t, *J* = 7.1 Hz, 3H). ^{13}C NMR (150 MHz, $CDCl_3$): δ 169.0, 164.0, 136.9, 134.2, 129.6, 129.5, 128.6, 116.8, 113.5, 109.3, 100.9, 98.8, 76.0, 59.5, 44.5, 32.9, 14.5. HRMS (ESI-TOF) *m/z* calcd. for $C_{18}H_{23}N_2O_4S^+$ ($[M+H]^+$) 363.1373. found, 363.1377.

ethyl (*E*)-4-(dimethyl(oxo)-λ⁶-sulfaneylidene)-5-((1-methyl-1*H*-indazol-7-yl)amino)-5-oxopent-2-enoate (46)

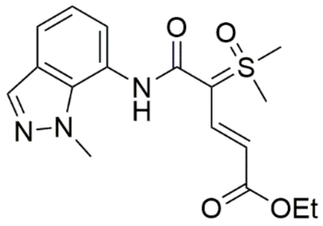

The title compound was prepared according to the general procedure and purified by column chromatography on silica gel and eluted with dichloromethane/ethyl acetate (1/1) to afford a colorless oil in 90% yield (32.7 mg). ^{1}H NMR (600 MHz, $CDCl_3$): δ 7.93 (s, 1H), 7.74 (d, *J* = 15.6 Hz, 1H), 7.58 (d, *J* = 8.0 Hz, 1H), 7.49 (s, 1H), 7.13 (d, *J* = 7.1 Hz, 1H), 7.07 (t, *J* = 7.6 Hz, 1H), 5.64 (d, *J* = 15.6 Hz, 1H), 4.16–4.12 (m, 2H), 4.12 (s, 3H), 3.47 (s, 6H), 1.24 (t, *J* = 7.0 Hz, 3H). ^{13}C NMR (150 MHz, $CDCl_3$): δ 168.8, 165.7, 136.4, 136.1, 132.8, 126.3, 124.6, 120.7, 120.5, 112.0, 99.9, 75.9, 59.6, 44.0, 37.7, 14.4. HRMS (ESI-TOF) *m/z* calcd. for $C_{17}H_{22}N_3O_4S^+$ ([M+H]$^+$) 364.1326. found, 363.1332.

ethyl (*E*)-4-(dimethyl(oxo)-λ⁶-sulfaneylidene)-5-((4-methyl-2-oxo-2*H*-chromen-7-yl)amino)-5-oxopent-2-enoate (47)

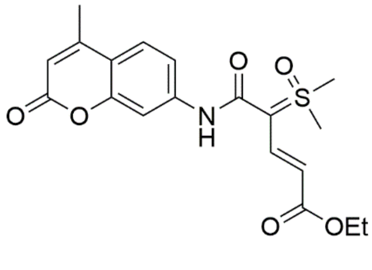

The title compound was prepared according to the general procedure and purified by column chromatography on silica gel and eluted with petroleum ether/ethyl acetate (1/1) to afford a yellow solid in 72% yield (28.2 mg). ^{1}H NMR (600 MHz, $CDCl_3$): δ 7.77 (s, 1H), 7.67 (d, *J* = 15.7 Hz, 2H), 7.49 (d, *J* = 8.6 Hz, 1H), 7.29–7.25 (m, 1H), 6.16 (s, 1H), 5.56 (d, *J* = 15.5 Hz, 1H), 4.18 (q, *J* = 7.1 Hz, 2H), 3.57 (s, 6H), 2.39 (d, *J* = 1.2 Hz, 3H), 1.30–1.26 (m, 3H). ^{13}C NMR (150 MHz, $CDCl_3$): δ 168.9, 163.6, 161.2, 154.3, 152.2, 141.8, 136.4, 125.0, 115.7, 115.5, 113.0, 107.2, 100.7, 76.5, 59.8, 44.1, 18.5, 14.5. HRMS (ESI-TOF) *m/z* calcd. for $C_{19}H_{22}NO_6S^+$ ([M+H]$^+$) 392.1162. found, 362.1158.

ethyl (*E*)-5-((2-(1*H*-indol-3-yl)ethyl)amino)-4-(dimethyl(oxo)-λ^6-sulfaneylidene)-5-oxopent-2-enoate (48)

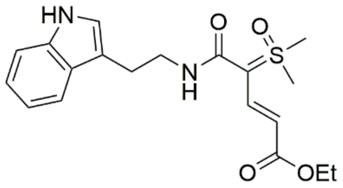

The title compound was prepared according to the general procedure and purified by column chromatography on silica gel and eluted with petroleum ether/ethyl acetate (1/1) to afford a yellow oil in 55% yield (20.7 mg). ^{1}H NMR (600 MHz, $CDCl_3$): δ 8.41 (s, 1H), 7.61 (d, *J* = 7.8 Hz, 1H), 7.57 (d, *J* = 15.6 Hz, 1H), 7.36 (d, *J* = 8.1 Hz, 1H), 7.18 (t, *J* = 7.6 Hz, 1H), 7.13–7.08 (m, 2H), 5.68 (s, 1H), 5.16 (d, *J* = 15.5 Hz, 1H), 4.16 (q, *J* = 7.1 Hz, 2H), 3.62 (q, *J* = 6.6 Hz, 2H), 3.40 (s, 6H), 3.02 (t, *J* = 6.6 Hz, 2H), 1.28 (t, *J* = 7.1 Hz, 3H). ^{13}C NMR (150 MHz, $CDCl_3$): δ 169.1, 165.3, 136.9, 136.6, 127.0, 122.6, 122.1, 119.3, 118.7, 112.7, 111.4, 98.1, 75.5, 59.4, 44.5, 39.4, 25.6, 14.6. HRMS (ESI-TOF) *m/z* calcd. for $C_{19}H_{25}N_2O_4S^+$ ($[M+H]^+$) 377.1530. found, 377.1537.

ethyl (*E*)-4-(dimethyl(oxo)-λ^6-sulfaneylidene)-5-(methoxy(methyl)amino)-5-oxopent-2-enoate (49)

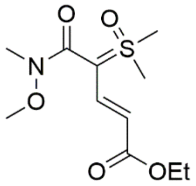

The title compound was prepared according to the general procedure and purified by column chromatography on silica gel and eluted with dichloromethane/ethyl acetate (1/1) to afford a colorless oil in 51% yield (14.1 mg). ^{1}H NMR (600 MHz, $CDCl_3$): δ 7.56 (d, *J* = 15.3 Hz, 1H), 5.44 (d, *J* = 15.3 Hz, 1H), 4.14 (q, *J* = 7.1 Hz, 2H), 3.67 (s, 3H), 3.47 (s, 6H), 3.13 (s, 3H), 1.25 (t, *J* = 7.1 Hz, 3H). ^{13}C NMR (150 MHz, $CDCl_3$): δ 169.4, 169.0, 135.7, 102.3, 75.6, 61.1, 59.4, 43.8, 37.7, 14.5. HRMS (ESI-TOF) *m/z* calcd. for $C_{11}H_{20}NO_5S^+$ ($[M+H]^+$) 278.1057. found, 278.1055.

ethyl (*E*)-4-(dimethyl(oxo)-λ^6-sulfaneylidene)-5-(methyl(naphthalen-1-ylmethyl)amino)-5-oxopent-2-enoate (50)

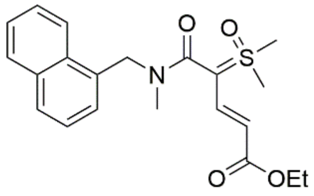

The title compound was prepared according to the general procedure and purified by column chromatography on silica gel and eluted with dichloromethane/ethyl acetate (1/2) to afford a colorless oil in 81% yield (31.3 mg). ^{1}H NMR (600 MHz, CDCl$_3$):δ 8.01 (dd, *J* = 6.1, 3.4 Hz, 1H), 7.85 (dd, *J* = 6.1, 3.3 Hz, 1H), 7.78 (d, *J* = 8.2 Hz, 1H), 7.56 (d, *J* = 15.0 Hz, 1H), 7.48 (dd, *J* = 6.3, 3.2 Hz, 2H), 7.43–7.39 (m, 1H), 7.31 (d, *J* = 6.9 Hz, 1H), 5.10 (d, *J* = 15.0 Hz, 1H), 5.00 (s, 2H), 4.11 (q, *J* = 7.1 Hz, 2H), 3.45 (s, 6H), 2.84 (s, 3H), 1.23 (t, *J* = 7.1 Hz, 3H). ^{13}C NMR (150 MHz, CDCl$_3$): δ 168.8, 168.2, 136.3, 133.7, 132.5, 131.4, 128.7, 128.2, 126.2, 126.0, 125.9, 125.1, 123.2, 100.9, 76.5, 59.2, 50.4, 43.5, 35.6, 14.5. HRMS (ESI-TOF) *m/z* calcd. for $C_{21}H_{26}NO_4S^+$ ([M+H]$^+$) 388.1577. found, 388.1584.

ethyl (*E*)-4-(dimethyl(oxo)-λ^6-sulfaneylidene)-5-(4-(2-((2,4-dimethylphenyl)thio)phenyl)piperazin-1-yl)-5-oxopent-2-enoate (51)

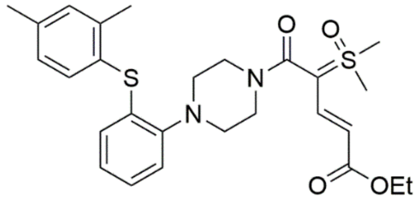

The title compound was prepared according to the general procedure and purified by column chromatography on silica gel and eluted with petroleum ether/ethyl acetate (1/2) to afford a colorless oil in 85% yield (43.7 mg). ^{1}H NMR (600 MHz, CDCl$_3$): δ 7.55 (d, *J* = 15.0 Hz, 1H), 7.35 (d, *J* = 7.8 Hz, 1H), 7.13 (s, 1H), 7.05 (t, *J* = 7.5 Hz, 1H), 7.01 (d, *J* = 7.7 Hz, 2H), 6.85 (t, *J* = 7.5 Hz, 1H), 6.50 (d, *J* = 8.9 Hz, 1H), 5.25 (d, *J* = 15.0 Hz, 1H), 4.14 (q, *J* = 7.1 Hz, 2H), 3.65 (s, 4H), 3.47 (s, 6H), 3.05 (t, *J* = 4.8 Hz, 4H), 2.34 (s, 3H), 2.30 (s, 3H), 1.26 (t, *J* = 7.1 Hz, 3H). ^{13}C NMR (150 MHz, CDCl$_3$): δ 168.9, 167.7, 148.4, 142.3, 139.2, 136.1, 136.0, 134.6, 131.6, 127.7, 127.5, 126.1, 125.3, 124.6, 119.8, 100.5, 76.5, 59.2, 52.0, 45.9, 43.8, 21.1, 20.5, 14.5. HRMS (ESI-TOF) *m/z* calcd. for $C_{27}H_{35}N_2O_4S_2^+$ ([M+H]$^+$) 515.2033. found, 515.2025.

ethyl (*E*)-4-(dimethyl(oxo)-λ⁶-sulfaneylidene)-5-(3-methyl-1*H*-indol-1-yl)-5-oxopent-2-enoate (52)

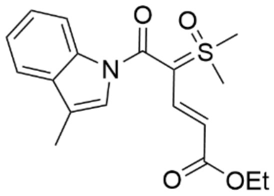

The title compound was prepared according to the general procedure and purified by column chromatography on silica gel and eluted with petroleum ether/ethyl acetate (2/1) to afford a yellow solid in 65% yield (22.6 mg). ^{1}H NMR (600 MHz, $CDCl_3$): δ 7.90 (d, *J* = 8.1 Hz, 1H), 7.59 (d, *J* = 15.5 Hz, 1H), 7.50 (d, *J* = 7.7 Hz, 1H), 7.28 (t, *J* = 7.1 Hz, 1H), 7.23 (t, *J* = 7.4 Hz, 1H), 7.18 (s, 1H), 5.45 (d, *J* = 15.5 Hz, 1H), 4.11 (q, *J* = 7.1 Hz, 2H), 3.61 (s, 6H), 2.27 (s, 3H), 1.21 (t, *J* = 7.1 Hz, 3H). ^{13}C NMR (150 MHz, $CDCl_3$): δ 168.5, 164.4, 135.7, 135.5, 131.5, 124.2, 124.1, 122.4, 119.0, 116.5, 114.9, 104.4, 78.7, 59.6, 43.3, 14.4, 9.7. HRMS (ESI-TOF) *m/z* calcd. for $C_{18}H_{22}NO_4S^+$ ($[M+H]^+$) 348.1264. found, 348.1267.

ethyl (*E*)-4-(dimethyl(oxo)-λ⁶-sulfaneylidene)-5-(5-fluoro-1*H*-indol-1-yl)-5-oxopent-2-enoate (53)

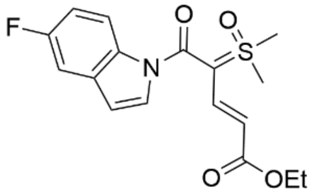

The title compound was prepared according to the general procedure and purified by column chromatography on silica gel and eluted with petroleum ether/ethyl acetate (1/1) to afford a yellow solid in 58% yield (20.4 mg). ^{1}H NMR (600 MHz, $CDCl_3$): δ 7.87 (dd, *J* = 9.0, 4.6 Hz, 1H), 7.56 (d, *J* = 15.6 Hz, 1H), 7.45 (d, *J* = 3.6 Hz, 1H), 7.22 (dd, *J* = 8.9, 2.5 Hz, 1H), 7.02–6.99 (m, *J* = 9.1, 2.5 Hz, 1H), 6.55 (d, *J* = 4.3 Hz, 1H), 5.43 (d, *J* = 15.6 Hz, 1H), 4.11 (q, *J* = 7.1 Hz, 2H), 3.63 (s, 6H), 1.21 (t, *J* = 7.1 Hz, 3H). ^{13}C NMR (150 MHz, $CDCl_3$): δ 168.3, 164.2, 159.1 (d, *J* = 238.5 Hz), 135.5, 131.5, 131.1 (d, *J* = 10.1 Hz), 128.7, 115.5 (d, *J* = 9.2 Hz), 111.7, 106.8, 106.2 (d, *J* = 23.7 Hz), 104.8, 78.9, 59.7, 43.1, 14.4. ^{19}F NMR (564 MHz, $CDCl_3$): δ -120.9 (s, 1F). HRMS (ESI-TOF) *m/z* calcd. for $C_{17}H_{19}FNO_4S^+$ ($[M+H]^+$) 352.1013. found, 352.1013.

ethyl (*E*)-5-(5-chloro-1*H*-indol-1-yl)-4-(dimethyl(oxo)-λ^6-sulfaneylidene)-5-oxopent-2-enoate (54)

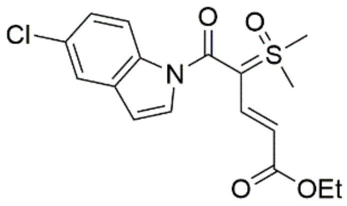

The title compound was prepared according to the general procedure and purified by column chromatography on silica gel and eluted with petroleum ether/ethyl acetate (1/2) to afford a yellow oil in 64% yield (23.5 mg). ^{1}H NMR (600 MHz, CDCl$_3$): δ 7.83 (d, *J* = 8.8 Hz, 1H), 7.53 (dd, *J* = 8.7, 6.8 Hz, 2H), 7.42 (d, *J* = 3.6 Hz, 1H), 7.21 (dd, *J* = 8.8, 2.0 Hz, 1H), 6.52 (d, *J* = 3.5 Hz, 1H), 5.40 (d, *J* = 15.6 Hz, 1H), 4.09 (q, *J* = 7.1 Hz, 2H), 3.60 (s, 6H), 1.20 (t, *J* = 7.1 Hz, 3H). ^{13}C NMR (150 MHz, CDCl$_3$): δ 168.2, 164.0, 135.3, 133.4, 131.5, 128.4, 128.0, 124.0, 120.4, 115.7, 106.3, 105.0, 79.1, 59.7, 43.0, 14.4. HRMS (ESI-TOF) *m/z* calcd. for $C_{17}H_{19}ClNO_4S^+$ ([M+H]$^+$) 368.0718. found, 368.0724.

ethyl (*E*)-5-(6-bromo-1*H*-indazol-1-yl)-4-(dimethyl(oxo)-λ^6-sulfaneylidene)-5-oxopent-2-enoate (55)

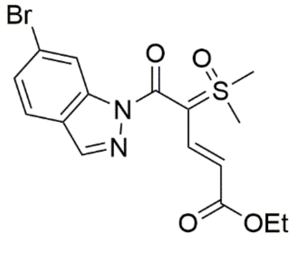

The title compound was prepared according to the general procedure and purified by column chromatography on silica gel and eluted with petroleum ether/ethyl acetate (1/1) to afford a white solid in 40% yield (16.5 mg). ^{1}H NMR (600 MHz, CDCl$_3$): δ 8.36 (s, 1H), 8.12 (s, 1H), 8.10 (d, *J* = 15.7 Hz, 1H), 7.59 (d, *J* = 8.4 Hz, 1H), 7.40 (dd, *J* = 8.5, 1.6 Hz, 1H), 5.87 (d, *J* = 15.7 Hz, 1H), 4.14 (q, *J* = 7.1 Hz, 2H), 3.69 (s, 6H), 1.24 (t, *J* = 7.1 Hz, 3H). ^{13}C NMR (150 MHz, CDCl$_3$): δ 168.5, 163.2, 140.6, 137.9, 137.6, 127.0, 124.3, 123.0, 121.9, 117.6, 104.4, 79.6, 59.7, 43.4, 14.4. HRMS (ESI-TOF) *m/z* calcd. for $C_{16}H_{18}BrN_2O_4S^+$ ([M+H]$^+$) 413.0165. found, 413.0159.

ethyl (*E*)-4-(dimethyl(oxo)-λ^6-sulfaneylidene)-5-oxo-5-(1*H*-pyrrol-1-yl)pent-2-enoate (56)

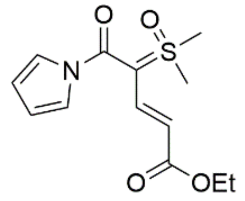

The title compound was prepared according to the general procedure and purified by column chromatography on silica gel and eluted with petroleum ether/ethyl acetate (1/1) to afford a colorless solid in 37% yield (10.5 mg). ^{1}H NMR (600 MHz, CDCl$_3$): δ 7.60 (d, J = 15.5 Hz, 1H), 7.13 (s, 2H), 6.25 (s, 2H), 5.66 (d, J = 15.6 Hz, 1H), 4.13 (q, J = 7.1 Hz, 2H), 3.58 (s, 6H), 1.23 (t, J = 7.1 Hz, 3H). ^{13}C NMR (150 MHz, CDCl$_3$): δ 168.4, 163.8, 136.2, 120.2, 111.6, 104.7, 78.5, 59.7, 43.0, 14.4. HRMS (ESI-TOF) *m/z* calcd. for $C_{13}H_{18}NO_4S^+$ ([M+H]$^+$) 284.0951. found, 284.0943.

ethyl (*E*)-4-(dimethyl(oxo)-λ^6-sulfaneylidene)-5-(3,5-dimethyl-1*H*-pyrazol-1-yl)-5-oxopent-2-enoate (57)

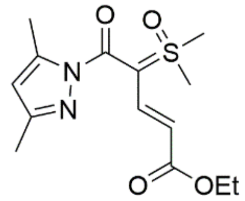

The title compound was prepared according to the general procedure and purified by column chromatography on silica gel and eluted with petroleum ether/ethyl acetate (1/1) to afford a colorless oil in 42% yield (13.1 mg). ^{1}H NMR (600 MHz, CDCl$_3$): δ 7.75 (d, J = 15.8 Hz, 1H), 5.94 (s, 1H), 5.56 (d, J = 15.8 Hz, 1H), 4.11 (q, J = 7.1 Hz, 2H), 3.62 (s, 6H), 2.37 (s, 3H), 2.24 (s, 3H), 1.22 (t, J = 7.1 Hz, 3H). ^{13}C NMR (150 MHz, CDCl$_3$): δ 168.4, 163.7, 150.5, 142.1, 137.2, 109.2, 104.4, 81.3, 59.5, 43.0, 14.4, 13.6, 12.7. HRMS (ESI-TOF) *m/z* calcd. for $C_{14}H_{21}N_2O_4S^+$ ([M+H]$^+$) 313.1217. found, 313.1215.

ethyl (*E*)-4-(dimethyl(oxo)-λ^6-sulfaneylidene)-5-oxo-5-phenylpent-2-enoate (58)

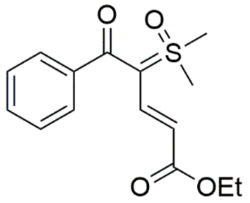

The title compound was prepared according to the general procedure and purified by column chromatography on silica gel and eluted with petroleum ether/ethyl acetate (1/1) to afford a yellow oil in 55% yield (16.2 mg). ^{1}H NMR (600 MHz, $CDCl_3$): δ 7.62 (d, *J* = 15.7 Hz, 1H), 7.54–7.50 (m, 2H), 7.44 (t, *J* = 7.3 Hz, 1H), 7.39 (t, *J* = 7.2 Hz, 2H), 5.59 (d, *J* = 15.7 Hz, 1H), 4.06 (q, *J* = 7.1 Hz, 2H), 3.61 (s, 6H), 1.18 (t, *J* = 7.1 Hz, 3H). ^{13}C NMR (150 MHz, $CDCl_3$): δ 187.6, 168.5, 139.5, 137.8, 130.7, 128.4, 128.0, 103.2, 88.4, 59.5, 43.2, 14.3. HRMS (ESI-TOF) *m/z* calcd. for $C_{15}H_{19}O_4S^+$ ([M+H]$^+$) 295.0999. found, 295.0996.

ethyl (*E*)-4-(dimethyl(oxo)-λ^6-sulfaneylidene)-5-(1-methyl-1*H*-indol-3-yl)-5-oxopent-2-enoate (59)

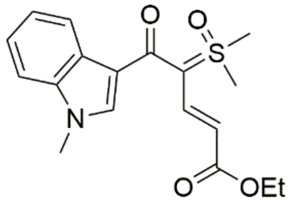

The title compound was prepared according to the general procedure and purified by column chromatography on silica gel and eluted with petroleum ether/ethyl acetate (1/2) to afford a yellow solid in 65% yield (22.6 mg). ^{1}H NMR (600 MHz, $CDCl_3$): δ 7.99 (d, *J* = 7.9 Hz, 1H), 7.96 (d, *J* = 15.5 Hz, 1H), 7.53 (s, 1H), 7.32 (d, *J* = 8.2 Hz, 1H), 7.29–7.24 (m, 1H), 7.21 (t, *J* = 7.5 Hz, 1H), 5.92 (d, *J* = 15.5 Hz, 1H), 4.11 (q, *J* = 7.1 Hz, 2H), 3.81 (s, 3H), 3.62 (s, 6H), 1.21 (t, *J* = 7.1 Hz, 3H). ^{13}C NMR (150 MHz, $CDCl_3$): δ 182.3, 169.0, 139.5, 137.0, 133.5, 126.7, 122.7, 121.6, 121.5, 114.7, 109.6, 100.7, 87.5, 59.3, 43.8, 33.3, 14.4. HRMS (ESI-TOF) *m/z* calcd. for $C_{18}H_{22}NO_4S^+$ ([M+H]$^+$) 348.1264. found, 348.1258.

1-ethyl 5-(4-methoxyphenyl) (*E*)-4-(dimethyl(oxo)-λ^6-sulfaneylidene)pent-2-enedioate (60)

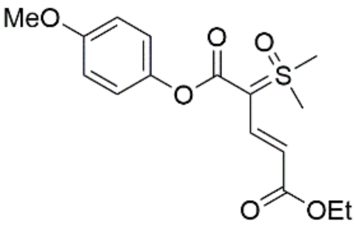

The title compound was prepared according to the general procedure and purified by column chromatography on silica gel and eluted with petroleum ether/ethyl acetate (1/1) to afford a colorless oil in 82% yield (27.9 mg). ^{1}H NMR (600 MHz, $CDCl_3$): δ 7.61 (d, *J* = 15.4 Hz, 1H), 7.03 (d, *J* = 9.0 Hz, 2H), 6.88 (d, *J* = 9.0 Hz, 2H), 6.12 (d, *J* = 15.4 Hz, 1H), 4.15 (q, *J* = 7.1 Hz, 2H), 3.78 (s, 3H), 3.48 (s, 6H), 1.25 (t, *J* = 7.1 Hz, 3H). ^{13}C NMR (150 MHz, $CDCl_3$): δ 169.2, 157.1, 143.7, 134.5, 122.9, 114.3, 104.4, 73.4, 59.4, 55.5, 42.9, 14.5. HRMS (ESI-TOF) *m/z* calcd. for $C_{16}H_{21}O_6S^+$ ($[M+H]^+$) 341.1053. found, 341.1058.

5-benzyl 1-ethyl (*E*)-4-(dimethyl(oxo)-λ^6-sulfaneylidene)pent-2-enedioate (61)

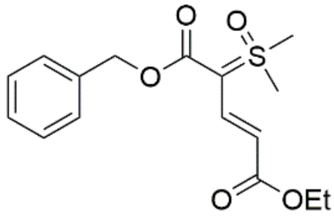

The title compound was prepared according to the general procedure and purified by column chromatography on silica gel and eluted with petroleum ether/ethyl acetate (1/1) to afford a colorless oil in 80% yield (25.9 mg). ^{1}H NMR (600 MHz, $CDCl_3$): δ 7.54 (d, *J* = 15.3 Hz, 1H), 7.38–7.32 (m, 4H), 7.28 (t, *J* = 7.0 Hz, 1H), 6.03 (d, *J* = 15.3 Hz, 1H), 5.22 (s, 2H), 4.13 (q, *J* = 7.1 Hz, 2H), 3.43 (s, 6H), 1.24 (t, *J* = 7.1 Hz, 3H). ^{13}C NMR (150 MHz, $CDCl_3$): δ 169.3, 136.4, 135.1, 128.5, 127.9, 127.6, 103.4, 73.3, 65.4, 59.3, 43.1, 14.5. HRMS (ESI-TOF) *m/z* calcd. for $C_{16}H_{21}O_5S^+$ ($[M+H]^+$) 325.1104. found, 325.1101.

methyl (*E*)-4-(dimethyl(oxo)-λ^6-sulfaneylidene)-5-(diphenylamino)-5-oxopent-2-enoate (62)

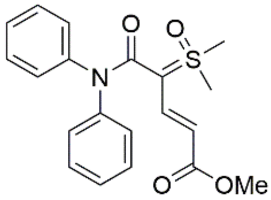

The title compound was prepared according to the general procedure and purified by column chromatography on silica gel and eluted with petroleum ether/ethyl acetate (1/2) to afford a yellow oil in 71% yield (26.3 mg). ^{1}H NMR (600 MHz, $CDCl_3$): δ 7.27 (q, *J* = 6.3, 4.8 Hz, 4H), 7.19 (d, *J* = 15.4 Hz, 1H), 7.12 (dd, *J* = 16.3, 7.7 Hz, 6H), 5.40 (d, *J* = 15.4 Hz, 1H), 3.53 (s, 3H), 3.52 (s, 6H). ^{13}C NMR (150 MHz, $CDCl_3$): δ 168.6, 168.3, 144.1, 135.1, 128.9, 126.3, 125.5, 102.2, 78.2, 50.6, 43.5. HRMS (ESI-TOF) *m/z* calcd. for $C_{20}H_{22}NO_4S^+$ ([M+H]$^+$) 372.1264. found, 372.1258.

***tert*-butyl (*E*)-4-(dimethyl(oxo)-λ^6-sulfaneylidene)-5-(diphenylamino)-5-oxopent-2-enoate (63)**

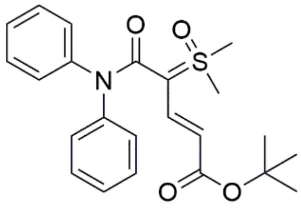

The title compound was prepared according to the general procedure and purified by column chromatography on silica gel and eluted with petroleum ether/ethyl acetate (1/2) to afford a yellow oil in 80% yield (33.0 mg). ^{1}H NMR (600 MHz, CDCl): δ 7.50–7.44 (m, 5H), 7.30 (q, *J* = 9.4, 8.0 Hz, 6H), 5.56 (d, *J* = 15.5 Hz, 1H), 3.70 (s, 6H), 1.54 (s, 9H). ^{13}C NMR (150 MHz, $CDCl_3$): δ 168.4, 167.6, 144.2, 134.1, 128.9, 126.3, 125.3, 105.4, 78.3, 77.2, 43.4, 28.2. HRMS (ESI-TOF) *m/z* calcd. for $C_{23}H_{28}NO_4S^+$ ([M+H]$^+$) 414.1743. found, 414.1751.

benzyl (*E*)-4-(dimethyl(oxo)-λ^6-sulfaneylidene)-5-(diphenylamino)-5-oxopent-2-enoate (64)

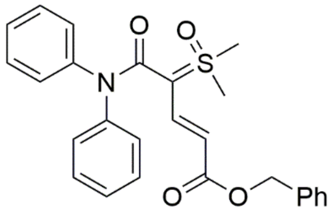

The title compound was prepared according to the general procedure and purified by column chromatography on silica gel and eluted with petroleum ether/ethyl acetate (1/2) to afford a yellow oil in 83% yield (37.1 mg). ^{1}H NMR (600 MHz, $CDCl_3$): δ 7.33 (t, J = 7.2 Hz, 2H), 7.30–7.25 (m, 8H), 7.16–7.11 (m, 6H), 5.49 (d, J = 15.4 Hz, 1H), 5.04 (s, 2H), 3.51 (s, 6H). ^{13}C NMR (150 MHz, $CDCl_3$): δ 168.2, 168.0, 144.1, 137.1, 135.6, 128.9, 128.2, 127.6, 127.5, 126.3, 125.4, 102.1, 78.4, 64.8, 43.4. HRMS (ESI-TOF) *m/z* calcd. for $C_{26}H_{26}NO_4S^+$ ($[M+H]^+$) 448.1577. found, 448.1581.

4-ethynylbenzyl (*E*)-4-(dimethyl(oxo)-λ^6-sulfaneylidene)-5-(diphenylamino)-5-oxopent-2-enoate (65)

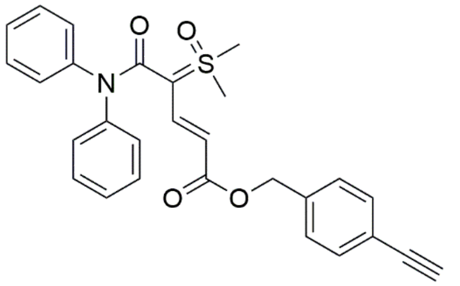

The title compound was prepared according to the general procedure and purified by column chromatography on silica gel and eluted with petroleum ether/ethyl acetate (1/1) to afford a yellow oil in 65% yield (30.6 mg). ^{1}H NMR (600 MHz, $CDCl_3$): δ 7.44 (d, J = 8.2 Hz, 2H), 7.29–7.26 (m, 4H), 7.25 (d, J = 7.7 Hz, 1H), 7.19 (d, J = 8.1 Hz, 2H), 7.15–7.10 (m, 6H), 5.46 (d, J = 15.4 Hz, 1H), 5.02 (s, 2H), 3.54 (s, 6H), 3.07 (s, 1H). ^{13}C NMR (150 MHz, $CDCl_3$): δ 168.2, 167.8, 144.1, 138.0, 135.8, 132.0, 129.0, 127.4, 126.3, 125.5, 121.2, 101.9, 83.4, 78.5, 77.2, 64.2, 43.6. HRMS (ESI-TOF) *m/z* calcd. for $C_{28}H_{26}NO_4S^+$ ($[M+H]^+$) 472.1577. found, 472.1572.

3-((propioloyloxy)methyl)benzyl (*E*)-4-(dimethyl(oxo)-λ⁶-sulfaneylidene)-5-(diphenylamino)-5-oxopent-2-enoate (66)

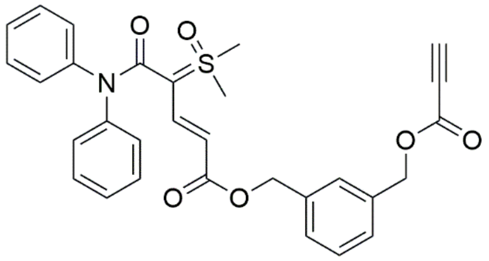

The title compound was prepared according to the general procedure and purified by column chromatography on silica gel and eluted with petroleum ether/ethyl acetate (1/2) to afford a yellow oil in 70% yield (37.0 mg). ^{1}H NMR (600 MHz, $CDCl_3$): δ 7.31 (q, *J* = 6.2, 5.0 Hz, 2H), 7.24–7.22 (m, 6H), 7.13–7.10 (m, 7H), 5.45 (d, *J* = 15.4 Hz, 1H), 5.18 (s, 2H), 5.01 (s, 2H), 3.50 (s, 6H), 2.88 (s, 1H). ^{13}C NMR (150 MHz, $CDCl_3$): δ 168.2, 167.9, 152.4, 144.0, 137.7, 135.7, 134.4, 128.9, 128.6, 127.9, 127.7, 127.6, 126.3, 125.5, 101.8, 78.6, 75.3, 74.3, 67.6, 64.4, 43.4. HRMS (ESI-TOF) *m/z* calcd. for $C_{30}H_{28}NO_6S^+$ ($[M+H]^+$) 530.1632. found, 530.1626.

adamantan-1-ylmethyl (*E*)-4-(dimethyl(oxo)-λ⁶-sulfaneylidene)-5-(diphenylamino)-5-oxopent-2-enoate (67)

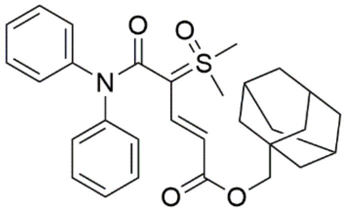

The title compound was prepared according to the general procedure and purified by column chromatography on silica gel and eluted with petroleum ether/ethyl acetate (1/1) to afford a yellow solid in 70% yield (35.4 mg). ^{1}H NMR (600 MHz, $CDCl_3$): δ 7.28 (t, *J* = 7.7 Hz, 4H), 7.19 (d, *J* = 15.4 Hz, 1H), 7.12 (d, *J* = 7.8 Hz, 6H), 5.41 (d, *J* = 15.4 Hz, 1H), 3.58 (s, 2H), 3.53 (s, 6H), 1.94 (s, 3H), 1.70 (d, *J* = 12.0 Hz, 3H), 1.62 (d, *J* = 11.7 Hz, 3H), 1.45 (s, 6H). ^{13}C NMR (150 MHz, $CDCl_3$): δ 168.4, 168.3, 144.1, 134.5, 128.9, 126.3, 125.4, 103.3, 78.0, 72.7, 43.5, 39.1, 36.9, 33.3, 28.0. HRMS (ESI-TOF) *m/z* calcd. for $C_{30}H_{36}NO_4S^+$ ($[M+H]^+$) 506.2360. found, 506.2358.

4-nitrophenyl (*E*)-4-(dimethyl(oxo)-λ⁶-sulfaneylidene)-5-(diphenylamino)-5-oxopent-2-enoate (68)

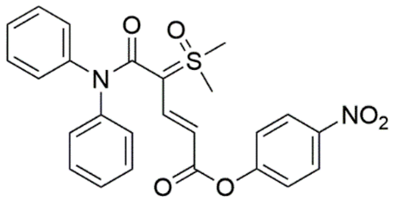

The title compound was prepared according to the general procedure and purified by column chromatography on silica gel and eluted with petroleum ether/ethyl acetate (1/1) to afford a yellow solid in 73% yield (34.9 mg). ^{1}H NMR (600 MHz, $CDCl_3$): δ 8.17 (d, *J* = 9.1 Hz, 2H), 7.43 (d, *J* = 15.2 Hz, 1H), 7.32 (t, *J* = 7.9 Hz, 4H), 7.19–7.13 (m, 8H), 5.53 (d, *J* = 15.2 Hz, 1H), 3.60 (s, 6H). ^{13}C NMR (150 MHz, $CDCl_3$): δ 167.9, 165.4, 156.6, 144.3, 143.9, 138.6, 129.1, 126.4, 125.8, 124.8, 122.3, 98.8, 80.4, 43.6. HRMS (ESI-TOF) *m/z* calcd. for $C_{25}H_{23}N_2O_6S^+$ ([M+H]$^+$) 479.1271. found, 479.1266.

naphthalen-2-yl (*E*)-4-(dimethyl(oxo)-λ⁶-sulfaneylidene)-5-(diphenylamino)-5-oxopent-2-enoate (69)

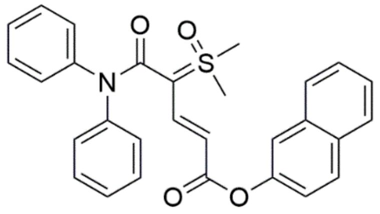

The title compound was prepared according to the general procedure and purified by column chromatography on silica gel and eluted with petroleum ether/ethyl acetate (1/2) to afford a yellow oil in 65% yield (31.4 mg). ^{1}H NMR (600 MHz, $CDCl_3$): δ 7.81 (t, *J* = 8.2 Hz, 2H), 7.77 (d, *J* = 8.0 Hz, 1H), 7.4–7.44 (m, 3H), 7.43 (t, *J* = 8.0 Hz, 1H), 7.35 (t, *J* = 7.9 Hz, 4H), 7.20 (dd, *J* = 15.4, 7.9 Hz, 6H), 7.16 (dd, *J* = 8.8, 2.3 Hz, 1H), 5.65 (d, *J* = 15.3 Hz, 1H), 3.54 (s, 6H). ^{13}C NMR (150 MHz, $CDCl_3$): δ 168.2, 166.8, 149.0, 144.0, 137.4, 133.7, 130.9, 129.1, 128.8, 127.6, 127.4, 126.4, 126.1, 125.7, 125.1, 121.8, 118.4, 100.6, 79.4, 43.5. HRMS (ESI-TOF) *m/z* calcd. for $C_{29}H_{26}NO_4S^+$ ([M+H]$^+$) 484.1577. found, 484.1583.

4-acetamidophenyl (*E*)-4-(dimethyl(oxo)-λ^6-sulfaneylidene)-5-(diphenylamino)-5-oxopent-2-enoate (70)

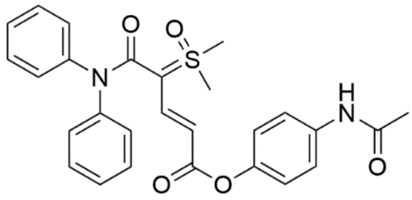

The title compound was prepared according to the general procedure and purified by column chromatography on silica gel and eluted with dichloromethane/ethyl acetate (1/3) to afford a white solid in 63% yield (30.9 mg). ^{1}H NMR (600 MHz, $CDCl_3$): δ 7.84 (s, 1H), 7.38 (d, *J* = 15.3 Hz, 1H), 7.33–7.28 (m, 6H), 7.16 (dd, *J* = 19.1, 7.4 Hz, 6H), 6.81 (d, *J* = 8.9 Hz, 2H), 5.55 (d, *J* = 15.3 Hz, 1H), 3.57 (s, 6H), 2.01 (s, 3H). ^{13}C NMR (150 MHz, $CDCl_3$): δ 168.6, 168.2, 167.3, 147.2, 144.0, 137.5, 135.1, 129.1, 126.4, 125.8, 121.9, 120.8, 100.6, 79.5, 43.8, 24.2. HRMS (ESI-TOF) *m/z* calcd. for $C_{27}H_{27}N_2O_5S^+$ ([M+H]$^+$) 491.1635. found, 491.1641.

4-(3-oxobutyl)phenyl (*E*)-4-(dimethyl(oxo)-λ^6-sulfaneylidene)-5-(diphenylamino)-5-oxopent-2-enoate (71)

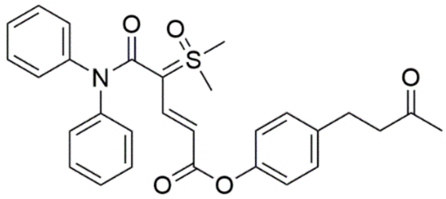

The title compound was prepared according to the general procedure and purified by column chromatography on silica gel and eluted with petroleum ether/ethyl acetate (1/2) to afford a colorless oil in 72% yield (36.2 mg). ^{1}H NMR (600 MHz, $CDCl_3$): δ 7.37 (d, *J* = 15.3 Hz, 1H), 7.31 (t, *J* = 7.9 Hz, 4H), 7.15 (dd, *J* = 16.9, 7.5 Hz, 6H), 7.10 (d, *J* = 8.4 Hz, 2H), 6.87 (d, *J* = 8.4 Hz, 2H), 5.56 (d, *J* = 15.3 Hz, 1H), 3.56 (s, 6H), 2.84 (t, *J* = 7.6 Hz, 2H), 2.71 (t, *J* = 7.6 Hz, 2H), 2.11 (s, 3H). ^{13}C NMR (150 MHz, $CDCl_3$): δ 207.9, 168.2, 166.7, 149.5, 144.0, 137.4, 137.2, 129.0, 128.8, 126.4, 125.7, 121.7, 100.9, 79.1, 45.1, 43.6, 30.0, 29.0. HRMS (ESI-TOF) *m/z* calcd. for $C_{29}H_{30}NO_5S^+$ ([M+H]$^+$) 504.1839. found, 504.1844.

S-(4-nitrophenyl) (*E*)-4-(dimethyl(oxo)-λ⁶-sulfaneylidene)-5-(diphenylamino)-5-oxopent-2-enethioate (72)

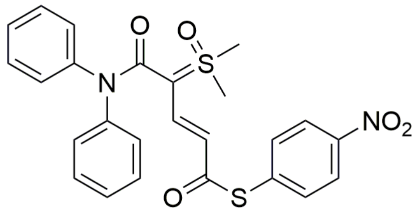

The title compound was prepared according to the general procedure and purified by column chromatography on silica gel and eluted with petroleum ether/ethyl acetate (1/2) to afford a yellow solid in 85% yield (42.0 mg). ^{1}H NMR (600 MHz, $CDCl_3$): δ 8.13 (d, *J* = 8.9 Hz, 2H), 7.53 (d, *J* = 8.9 Hz, 2H), 7.35–7.30 (m, 5H), 7.19 (t, *J* = 7.4 Hz, 2H), 7.13 (d, *J* = 8.5 Hz, 4H), 5.70 (d, *J* = 14.8 Hz, 1H), 3.57 (s, 6H). ^{13}C NMR (150 MHz, $CDCl_3$): δ 182.6, 167.7, 147.1, 143.6, 139.2, 134.2, 134.1, 129.1, 126.3, 126.0, 123.3, 107.7, 82.0, 43.6. HRMS (ESI-TOF) *m/z* calcd. for $C_{25}H_{23}N_2O_5S_2^+$ ($[M+H]^+$) 495.1043. found, 495.1038.

(*E*)-2-(dimethyl(oxo)-λ⁶-sulfaneylidene)-5-oxo-*N,N*-diphenylhex-3-enamide (73)

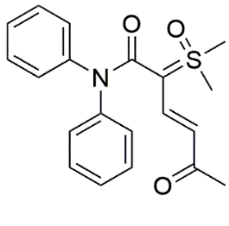

The title compound was prepared according to the general procedure and purified by column chromatography on silica gel and eluted with petroleum ether/ethyl acetate (1/1) to afford a yellow solid in 92% yield (32.7 mg). ^{1}H NMR (600 MHz, $CDCl_3$): δ 7.47 (t, *J* = 7.5 Hz, 4H), 7.33–7.28 (p, *J* = 7.3 Hz, 7H), 5.97 (d, *J* = 15.5 Hz, 1H), 3.73 (s, 6H), 2.02 (s, 3H). ^{13}C NMR (150 MHz, $CDCl_3$): δ 196.4, 167.8, 143.9, 135.0, 128.9, 126.3, 125.5, 113.7, 79.0, 43.5, 26.4. HRMS (ESI-TOF) *m/z* calcd. for $C_{20}H_{22}NO_3S^+$ ($[M+H]^+$) 356.1315. found, 356.1309.

(*E*)-2-(dimethyl(oxo)-λ^6-sulfaneylidene)-5-oxo-*N*,*N*-di-*p*-tolylhex-3-enamide (74)

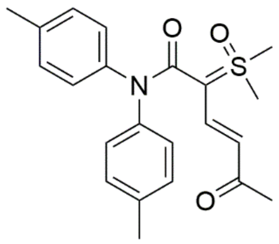

The title compound was prepared according to the general procedure and purified by column chromatography on silica gel and eluted with petroleum ether/ethyl acetate (1/1) to afford a yellow solid in 91% yield (34.9 mg). ^{1}H NMR (600 MHz, $CDCl_3$): δ 7.12–7.05 (m, 5H), 6.99 (d, *J* = 8.3 Hz, 4H), 5.77 (d, *J* = 15.5 Hz, 1H), 3.53 (s, 6H), 2.27 (s, 6H), 1.85 (s, 3H). ^{13}C NMR (150 MHz, $CDCl_3$): δ 196.4, 167.8, 141.4, 135.3, 129.5, 129.0, 126.1, 113.4, 78.9, 43.6, 26.3, 20.8. HRMS (ESI-TOF) *m/z* calcd. for $C_{22}H_{26}NO_3S^+$ ([M+H]$^+$) 384.1628. found, 384.1632.

(*E*)-*N*,*N*-bis(4-bromophenyl)-2-(dimethyl(oxo)-λ^6-sulfaneylidene)-5-oxohex-3-enamide (75)

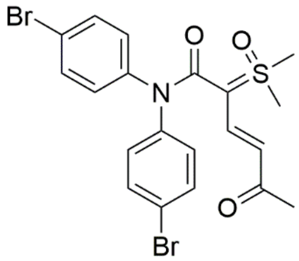

The title compound was prepared according to the general procedure and purified by column chromatography on silica gel and eluted with petroleum ether/ethyl acetate (1/1) to afford a yellow solid in 85% yield (43.6 mg). ^{1}H NMR (600 MHz, $CDCl_3$): δ 7.39 (d, *J* = 8.7 Hz, 4H), 7.04 (d, *J* = 15.5 Hz, 1H), 6.96 (d, *J* = 8.7 Hz, 4H), 5.73 (d, *J* = 15.5 Hz, 1H), 3.55 (s, 6H), 1.89 (s, 3H). ^{13}C NMR (150 MHz, $CDCl_3$): δ 196.4, 167.6, 142.6, 134.2, 132.2, 127.7, 119.1, 114.7, 79.0, 43.4, 26.6. HRMS (ESI-TOF) *m/z* calcd. for $C_{20}H_{20}Br_2NO_3S^+$ ([M+H]$^+$) 511.9525. found, 511.9527.

(*E*)-2-(dimethyl(oxo)-λ^6-sulfaneylidene)-1-(10*H*-phenothiazin-10-yl)hex-3-ene-1,5-dione (76)

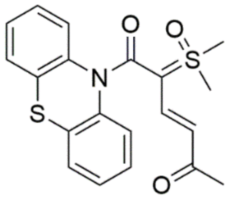

The title compound was prepared according to the general procedure and purified by column chromatography on silica gel and eluted with petroleum ether/ethyl acetate (1/1) to afford a yellow solid in 79% yield (30.4 mg). ^{1}H NMR (600 MHz, CDCl$_3$): δ 7.68 (d, *J* = 8.1 Hz, 2H), 7.37 (dd, *J* = 7.8, 1.4 Hz, 2H), 7.23–7.18 (m, 2H), 7.13–7.08 (m, 2H), 6.99 (d, *J* = 15.5 Hz, 1H), 5.87 (d, *J* = 15.5 Hz, 1H), 3.60 (s, 6H), 1.83 (s, 3H). ^{13}C NMR (150 MHz, CDCl$_3$): δ 196.5, 165.2, 139.9, 135.8, 131.8, 127.6, 127.0, 125.9, 125.3, 113.8, 78.0, 43.4, 26.5. HRMS (ESI-TOF) *m/z* calcd. for $C_{20}H_{20}NO_3S_2^+$ ([M+H]$^+$) 386.0879. found, 386.0885.

(*E*)-2-(dimethyl(oxo)-λ^6-sulfaneylidene)-5-oxo-*N*-(thiophen-2-ylmethyl)-*N*-(*p*-tolyl)hex-3-enamide (77)

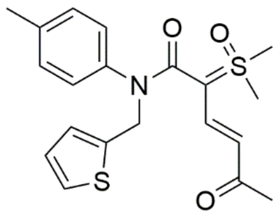

The title compound was prepared according to the general procedure and purified by column chromatography on silica gel and eluted with petroleum ether/ethyl acetate (1/1) to afford a yellow solid in 80% yield (31.2 mg). ^{1}H NMR (600 MHz, CDCl$_3$): δ 7.13 (d, *J* = 6.0 Hz, 1H), 7.06–7.00 (m, 3H), 6.96 (d, *J* = 8.3 Hz, 2H), 6.80–6.77 (m, 1H), 6.68 (d, *J* = 2.9 Hz, 1H), 5.65 (d, *J* = 15.4 Hz, 1H), 4.99 (s, 2H), 3.52 (s, 6H), 2.23 (s, 3H), 1.85 (s, 3H). ^{13}C NMR (150 MHz, CDCl$_3$): δ 196.3, 167.2, 140.4, 139.9, 136.1, 136.0, 129.5, 126.7, 126.1, 126.0, 125.4, 112.6, 77.8, 49.3, 43.9, 26.2, 20.8. HRMS (ESI-TOF) *m/z* calcd. for $C_{20}H_{24}NO_3S_2^+$ ([M+H]$^+$) 390.1192. found, 390.1191.

(*E*)-2-(dimethyl(oxo)-λ⁶-sulfaneylidene)-1-(4-(pyrimidin-2-yl)piperazin-1-yl)hex-3-ene-1,5-dione (78)

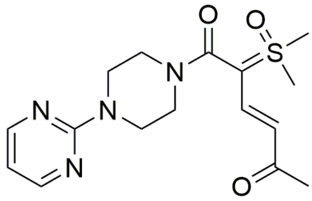

The title compound was prepared according to the general procedure and purified by column chromatography on silica gel and eluted with dichloromethane/ethyl acetate (1/2) to afford a yellow oil in 66% yield (23.1 mg). ^{1}H NMR (600 MHz, $CDCl_3$): δ 8.30 (d, *J* = 4.7 Hz, 2H), 7.47 (d, *J* = 15.0 Hz, 1H), 6.52 (t, *J* = 4.7 Hz, 1H), 5.63 (d, *J* = 15.0 Hz, 1H), 3.83 (s, 4H), 3.51 (t, *J* = 5.1 Hz, 4H), 3.48 (s, 6H), 2.15 (s, 3H). ^{13}C NMR (150 MHz, $CDCl_3$): δ 196.3, 167.5, 161.5, 157.7, 136.0, 112.3, 110.5, 78.0, 45.4, 44.0, 40.9, 26.9. HRMS (ESI-TOF) *m/z* calcd. for $C_{16}H_{23}N_4O_3S^+$ ([M+H]$^+$) 351.1485. found, 358.1488.

(*E*)-2-(dimethyl(oxo)-λ⁶-sulfaneylidene)-*N*-methyl-*N*-(naphthalen-1-ylmethyl)-5-oxohex-3-enamide (79)

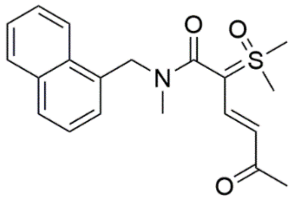

The title compound was prepared according to the general procedure and purified by column chromatography on silica gel and eluted with dichloromethane/ethyl acetate (1/3) to afford a colorless oil in 62% yield (22.1 mg). ^{1}H NMR (600 MHz, $CDCl_3$): δ 7.99 (dd, *J* = 6.2, 3.5 Hz, 1H), 7.86 (dd, *J* = 6.0, 3.5 Hz, 1H), 7.80 (d, *J* = 8.2 Hz, 1H), 7.50–7.46 (m, 3H), 7.42 (dd, *J* = 8.2, 7.0 Hz, 1H), 7.33 (d, *J* = 6.9 Hz, 1H), 5.42 (d, *J* = 14.9 Hz, 1H), 5.01 (s, 2H), 3.49 (s, 6H), 2.82 (s, 3H), 1.98 (s, 3H). ^{13}C NMR (150 MHz, $CDCl_3$): δ 196.1, 167.8, 136.2, 133.8, 132.4, 131.3, 128.8, 128.4, 126.3, 125.9, 125.1, 123.3, 112.1, 78.2, 50.4, 43.7, 35.7, 26.9. HRMS (ESI-TOF) *m/z* calcd. for $C_{20}H_{24}NO_3S^+$ ([M+H]$^+$) 358.1471. found, 358.1466.

(*E*)-2-(dimethyl(oxo)-l6-sulfanylidene)-1-(4-(2-((2,4-dimethylphenyl)thio)phenyl)piperazin-1-yl)hex-3-ene-1,5-dione (80)

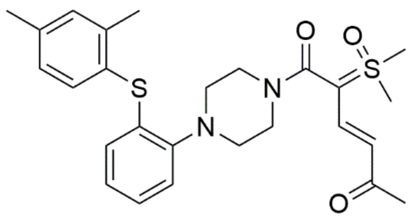

The title compound was prepared according to the general procedure and purified by column chromatography on silica gel and eluted with dichloromethane/ethyl acetate (1/1) to afford a yellow oil in 51% yield (24.7 mg). ^{1}H NMR (600 MHz, $CDCl_3$): δ 7.51 (d, *J* = 15.0 Hz, 1H), 7.36 (d, *J* = 7.8 Hz, 1H), 7.15 (s, 1H), 7.06 (t, *J* = 6.8 Hz, 1H), 7.02 (t, *J* = 7.3 Hz, 2H), 6.87 (t, *J* = 7.5 Hz, 1H), 6.51 (dd, *J* = 7.9, 1.3 Hz, 1H), 5.69 (d, *J* = 15.0 Hz, 1H), 3.66 (s, 4H), 3.50 (s, 6H), 3.07 (s, 4H), 2.35 (s, 3H), 2.31 (s, 3H), 2.19 (s, 3H). ^{13}C NMR (150 MHz, $CDCl_3$): δ 196.3, 148.4, 142.4, 139.3, 136.2, 136.1, 134.7, 131.7, 127.8, 127.6, 126.2, 125.4, 124.8, 119.8, 112.2, 78.1, 52.1, 45.9, 44.1, 27.0, 21.2, 20.6. HRMS (ESI-TOF) *m/z* calcd. for $C_{26}H_{33}N_2O_3S_2^+$ ([M+H]$^+$) 484.1927. found, 484.1939.

(*E*)-1-(4-(2-chlorodibenzo[*b*,*f*][1,4]oxazepin-11-yl)piperazin-1-yl)-2-(dimethyl(oxo)-λ^6-sulfaneylidene)hex-3-ene-1,5-dione (81)

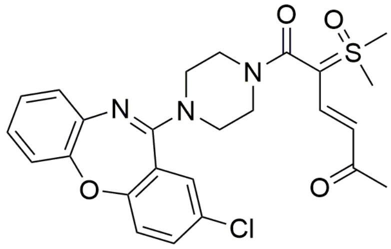

The title compound was prepared according to the general procedure and purified by column chromatography on silica gel and eluted with dichloromethane/ethyl acetate (1/2) to afford a yellow solid in 62% yield (30.9 mg). ^{1}H NMR (600 MHz, $CDCl_3$): δ 7.47 (d, *J* = 15.1 Hz, 1H), 7.39 (d, *J* = 8.4 Hz, 1H), 7.32 (s, 1H), 7.18 (d, *J* = 8.7 Hz, 1H), 7.14 (d, *J* = 7.7 Hz, 1H), 7.08 (t, *J* = 8.0 Hz, 2H), 7.00 (t, *J* = 7.6 Hz, 1H), 5.66 (d, *J* = 15.1 Hz, 1H), 3.66–3.51 (m, 8H), 3.48 (s, 6H), 2.17 (s, 3H). ^{13}C NMR (150 MHz, $CDCl_3$): δ 196.4, 167.6, 159.3, 158.9, 151.7, 139.7, 135.9, 132.8, 130.5, 128.9, 127.1, 125.8, 125.0, 124.8, 122.8, 120.12 112.4, 77.9, 47.8, 45.2, 44.0, 40.9, 29.6, 26.9. HRMS (ESI-TOF) *m/z* calcd. for $C_{25}H_{27}ClN_3O_4S^+$ ([M+H]$^+$) 500.1405. found, 500.1411.

(*E*)-2-(dimethyl(oxo)-λ^6-sulfaneylidene)-5-oxo-*N*,*N*,5-triphenylpent-3-enamide (82)

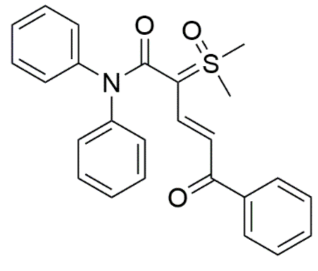

The title compound was prepared according to the general procedure and purified by column chromatography on silica gel and eluted with dichloromethane /ethyl acetate (1/1) to afford a yellow solid in 66% yield (27.6 mg). ^{1}H NMR (600 MHz, $CDCl_3$): δ 7.65 (d, J = 7.1 Hz, 2H), 7.53 (d, J = 14.8 Hz, 1H), 7.41 (t, J = 7.3 Hz, 1H), 7.34 (t, J = 7.6 Hz, 2H), 7.30 (t, J = 7.9 Hz, 4H), 7.20–7.11 (m, 6H), 6.45 (d, J = 14.8 Hz, 1H), 3.59 (s, 6H). ^{13}C NMR (150 MHz, $CDCl_3$): δ 189.0, 168.0, 144.0, 139.7, 136.3, 131.1, 129.1, 128.0, 127.8, 126.3, 125.8, 108.1, 82.4, 43.7. HRMS (ESI-TOF) *m/z* calcd. for $C_{25}H_{24}NO_3S^+$ ([M+H]$^+$) 418.1471. found, 418.1473.

(*E*)-2-(dimethyl(oxo)-λ^6-sulfaneylidene)-*N*-(4-methoxyphenyl)-5-oxo-5-phenylpent-3-enamide (83)

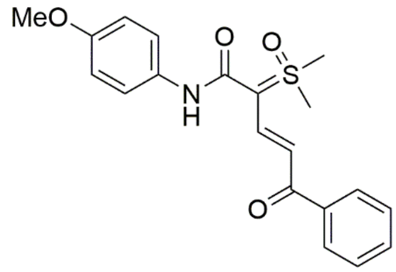

The title compound was prepared according to the general procedure and purified by column chromatography on silica gel and eluted with petroleum ether/ethyl acetate (1/2) to afford a yellow oil in 61% yield (22.6 mg). ^{1}H NMR (600 MHz, $CDCl_3$): δ 8.03–7.95 (m, 2H), 7.82 (d, J = 7.3 Hz, 2H), 7.43 (d, J = 8.5 Hz, 2H), 7.41–7.37 (m, 1H), 7.32 (t, J = 7.2 Hz, 2H), 6.88 (d, J = 8.4 Hz, 2H), 6.70 (d, J = 14.6 Hz, 1H), 3.78 (s, 3H), 3.55 (s, 6H). ^{13}C NMR (150 MHz, $CDCl_3$): δ 188.8, 163.7, 156.2, 139.8, 138.8, 131.1, 131.0, 128.1, 127.7, 122.4, 114.1, 104.5, 80.9, 55.4, 44.0. HRMS (ESI-TOF) *m/z* calcd. for $C_{20}H_{22}NO_4S^+$ ([M+H]$^+$) 372.1264. found, 372.1257.

(*E*)-2-(dimethyl(oxo)-λ⁶-sulfaneylidene)-1-(1-methyl-1*H*-indol-3-yl)hex-3-ene-1,5-dione (84)

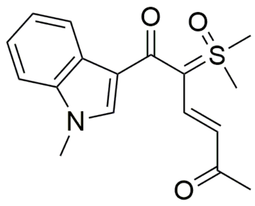

The title compound was prepared according to the general procedure and purified by column chromatography on silica gel and eluted with dichloromethane/ethyl acetate (1/1) to afford a yellow solid in 55% yield (17.4 mg). ^{1}H NMR (600 MHz, $CDCl_3$): δ 7.95 (d, *J* = 7.9 Hz, 1H), 7.88 (d, *J* = 15.5 Hz, 1H), 7.54 (s, 1H), 7.33 (d, *J* = 8.2 Hz, 1H), 7.27 (d, *J* = 8.0 Hz, 1H), 7.21 (t, *J* = 7.8 Hz, 1H), 6.32 (d, *J* = 15.5 Hz, 1H), 3.82 (s, 3H), 3.64 (s, 6H), 2.07 (s, 3H). ^{13}C NMR (150 MHz, $CDCl_3$): δ 197.2, 182.6, 139.1, 137.0, 133.8, 126.5, 122.8, 121.6, 121.5, 114.9, 111.3, 109.7, 88.4, 43.9, 33.3, 27.7. HRMS (ESI-TOF) *m/z* calcd. for $C_{17}H_{20}NO_3S^+$ ($[M+H]^+$) 318.1158. found, 318.1165.

(*E*)-4-(dimethyl(oxo)-λ⁶-sulfaneylidene)-*N⁵*,*N⁵*-diphenylpent-2-enediamide (85)

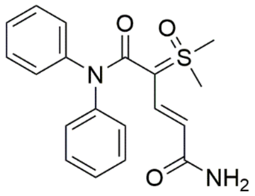

The title compound was prepared according to the general procedure and purified by column chromatography on silica gel and eluted with ethyl acetate/methanol (20/1) to afford a yellow oil in 35% yield (12.5 mg). ^{1}H NMR (600 MHz, $CDCl_3$): δ 7.31–7.27 (m, 5H), 7.15–7.09 (m, 6H), 5.41 (d, *J* = 15.2 Hz, 1H), 4.91 (br s, 2H), 3.56 (s, 6H). ^{13}C NMR (150 MHz, $CDCl_3$): δ 169.5, 168.4, 144.3, 132.8, 129.1, 127.8, 126.3, 125.6, 104.9, 43.8. HRMS (ESI-TOF) *m/z* calcd. for $C_{19}H_{21}N_2O_3S^+$ ($[M+H]^+$) 357.1267. found, 357.1273.

(*E*)-4-(dimethyl(oxo)-λ⁶-sulfaneylidene)-*N¹*,*N⁵*,*N⁵*-triphenylpent-2-enediamide (86)

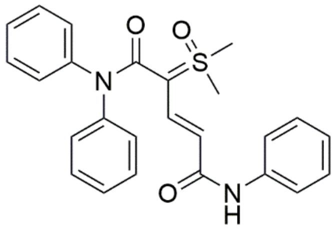

The title compound was prepared according to the general procedure and purified by column chromatography on

silica gel and eluted with petroleum ether/ethyl acetate (1/2) to afford a white solid in 32% yield (13.8 mg). ^{1}H NMR (600 MHz, DMSO-d_6): δ 9.33 (s, 1H), 7.55 (d, J = 7.7 Hz, 2H), 7.28 (t, J = 7.9 Hz, 4H), 7.22–7.18 (m, 2H), 7.14 (d, J = 7.5 Hz, 4H), 7.10 (t, J = 7.4 Hz, 2H), 6.98 (d, J = 14.9 Hz, 1H), 6.91 (t, J = 7.3 Hz, 1H), 5.68 (d, J = 14.9 Hz, 1H), 3.67 (s, 6H). ^{13}C NMR (150 MHz, DMSO-d_6): δ 168.7, 165.8, 145.1, 140.9, 131.5, 129.1, 128.9, 126.7, 125.2, 122.3, 119.1, 106.5, 80.6, 42.2. HRMS (ESI-TOF) *m/z* calcd. for $C_{25}H_{25}N_2O_3S^+$ ([M+H]$^+$) 433.1580. found, 433.1573.

(*E*)-4-(dimethyl(oxo)-λ^6-sulfaneylidene)-*N*^{1}-isopropyl-*N*^{1},*N*^{5},*N*^{5}-triphenylpent-2-enediamide (87)

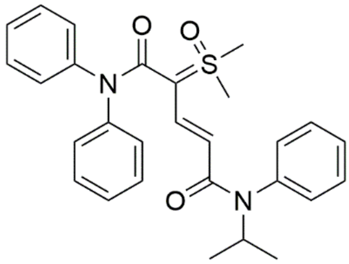

The title compound was prepared according to the general procedure and purified by column chromatography on silica gel and eluted with dichloromethane/ethyl acetate (1/3) to afford a colorless oil in 40% yield (18.9 mg). ^{1}H NMR (600 MHz, $CDCl_3$): δ 7.37 (t, J = 7.6 Hz, 2H), 7.29 (t, J = 7.8 Hz, 1H), 7.20 (t, J = 7.7 Hz, 4H), 7.17 (d, J = 14.9 Hz, 1H), 7.10 (t, J = 7.2 Hz, 2H), 7.03 (d, J = 7.9 Hz, 2H), 6.75 (d, J = 8.0 Hz, 4H), 5.10 (d, J = 14.9 Hz, 1H), 5.00–4.95 (m, 1H), 3.46 (s, 6H), 0.99 (d, J = 6.8 Hz, 6H). ^{13}C NMR (150 MHz, $CDCl_3$): δ 168.6, 167.0, 144.1, 139.1, 131.2, 131.1, 128.8, 128.6, 127.6, 126.3, 125.3, 106.3, 78.5, 45.5, 43.3, 21.2. HRMS (ESI-TOF) *m/z* calcd. for $C_{28}H_{31}N_2O_3S^+$ ([M+H]$^+$) 475.2050. found, 475.2043.

(*E*)-2-(dimethyl(oxo)-λ^6-sulfaneylidene)-*N*,*N*-diphenyl-4-tosylbut-3-enamide (88)

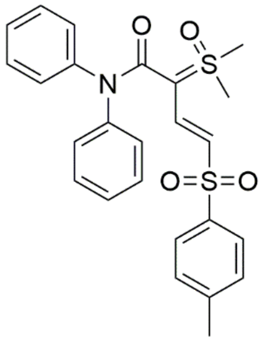

The title compound was prepared according to the general procedure and purified by column chromatography on silica gel and eluted with dichloromethane/ethyl acetate (2/1) to afford a colorless oil in 63% yield (29.4 mg). ^{1}H NMR (600 MHz, $CDCl_3$): δ 7.30 (d, *J* = 8.2 Hz, 2H), 7.22 (d, *J* = 14.9 Hz, 1H), 7.20–7.17 (m, 4H), 7.15 (d, *J* = 8.0 Hz, 2H), 7.06–7.02 (m, 6H), 5.73 (d, *J* = 14.9 Hz, 1H), 3.56 (s, 6H), 2.39 (s, 3H). ^{13}C NMR (150 MHz, $CDCl_3$): δ 168.2, 144.1, 142.2, 140.3, 133.7, 129.2, 129.1, 126.9, 126.3, 125.8, 109.3, 77.3, 43.8, 21.5. HRMS (ESI-TOF) *m/z* calcd. for $C_{25}H_{26}NO_4S_2^+$ ([M+H]$^+$) 468.1298. found, 468.1305.

(*E*)-2-(dimethyl(oxo)-λ^6-sulfaneylidene)-*N*-(thiophen-3-yl)-4-tosylbut-3-enamide (89)

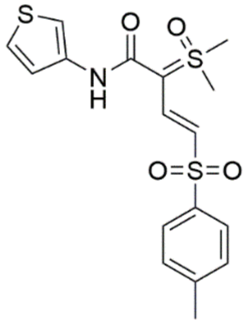

The title compound was prepared according to the general procedure and purified by column chromatography on silica gel and eluted with petroleum ether/ethyl acetate (1/1) to afford a yellow solid in 66% yield (26.2 mg). ^{1}H NMR (600 MHz, $CDCl_3$): δ 7.77 (d, *J* = 8.3 Hz, 2H), 7.58 (d, *J* = 15.0 Hz, 1H), 7.44 (s, 1H), 7.30–7.26 (m, 3H), 7.24 (dd, *J* = 5.1, 3.2 Hz, 1H), 7.01 (d, *J* = 5.1 Hz, 1H), 5.89 (d, *J* = 15.0 Hz, 1H), 3.56 (s, 6H), 2.40 (s, 3H). ^{13}C NMR (150 MHz, $CDCl_3$): δ 162.9, 142.9, 140.6, 135.3, 134.2, 129.6, 126.8, 124.8, 121.8, 110.0, 107.2, 74.9, 44.5, 21.5. HRMS (ESI-TOF) *m/z* calcd. for $C_{17}H_{20}NO_4S_3^+$ ([M+H]$^+$) 398.0549. found, 398.0557.

(*E*)-2-(dimethyl(oxo)-λ^6-sulfaneylidene)-*N*-methyl-*N*-(naphthalen-1-ylmethyl)-4-tosylbut-3-enamide (90)

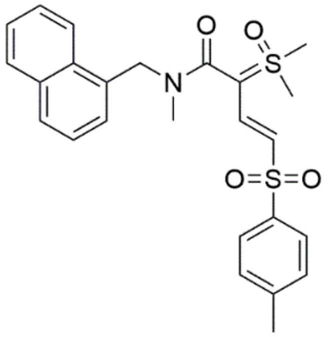

The title compound was prepared according to the general procedure and purified by column chromatography on silica gel and eluted with petroleum ether /ethyl acetate (1/2) to afford a white solid in 70% yield (32.8 mg). ^{1}H NMR (600 MHz, $CDCl_3$): δ 7.87 (d, J = 8.1 Hz, 1H), 7.80 (d, J = 8.2 Hz, 1H), 7.77 (d, J = 8.5 Hz, 1H), 7.49 (dd, J = 16.5, 8.1 Hz, 3H), 7.44–7.39 (m, 2H), 7.37 (t, J = 7.7 Hz, 1H), 7.29 (d, J = 6.9 Hz, 1H), 7.13 (d, J = 8.0 Hz, 2H), 5.39 (d, J = 14.5 Hz, 1H), 4.90 (s, 2H), 3.48 (s, 6H), 2.75 (s, 3H), 2.36 (s, 3H). ^{13}C NMR (150 MHz, $CDCl_3$): δ 168.3, 142.2, 141.1, 134.3, 133.8, 132.2, 131.2, 129.3, 128.9, 128.4, 126.31, 126.29, 125.9, 125.2, 122.9, 108.2, 75.5, 50.7, 43.7, 35.7, 21.4. HRMS (ESI-TOF) *m/z* calcd. for $C_{25}H_{28}NO_4S_2^+$ ([M+H]$^+$) 470.1454. found, 470.1447.

ethyl (*E*)-4-(dimethyl(oxo)-λ^6-sulfaneylidene)-5-(diphenylamino)-5-oxo-3-(trifluoromethyl)pent-2-enoate (91)

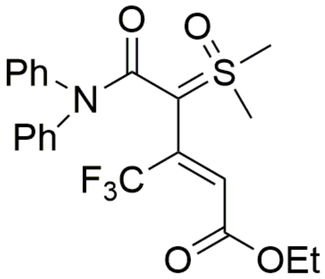

The title compound was prepared according to the general procedure and purified by column chromatography on silica gel and eluted with petroleum ether /ethyl acetate (1/1) to afford a colorless oil in 20% yield (9.1 mg). ^{1}H NMR (600 MHz, $CDCl_3$): δ 77.38 (t, J = 7.3 Hz, 2H), 7.33 (q, J = 7.4 Hz, 3H), 7.29–7.25 (m, 4H), 7.21 (t, J = 7.0 Hz, 1H), 6.46 (s, 1H), 3.99 (q, J = 7.1 Hz, 2H), 3.34 (s, 6H), 1.15 (t, J = 7.1 Hz, 3H). ^{13}C NMR (150 MHz, $CDCl_3$): δ 165.9, 164.1, 141.5, 141.3, 138.6, 129.7, 128.9, 128.7, 128.1, 126.6, 126.1, 126.0, 125.9, 122.8 (q, J = 275.0 Hz), 59.0, 42.4, 14.5. ^{19}F NMR (564 MHz, $CDCl_3$) δ -62.89. HRMS (ESI-TOF) *m/z* calcd. for $C_{22}H_{23}F_3NO_4S^+$ ([M+H]$^+$) 454.1294. found, 454.1297.

3-((((*E*)-4-(dimethyl(oxo)-λ⁶-sulfaneylidene)-5-(diphenylamino)-5-oxopent-2-enoyl)oxy)methyl)benzyl (*E*)-4-(dimethyl(oxo)-λ⁶-sulfaneylidene)-5-(diphenylamino)-5-oxopent-2-enoate (93)

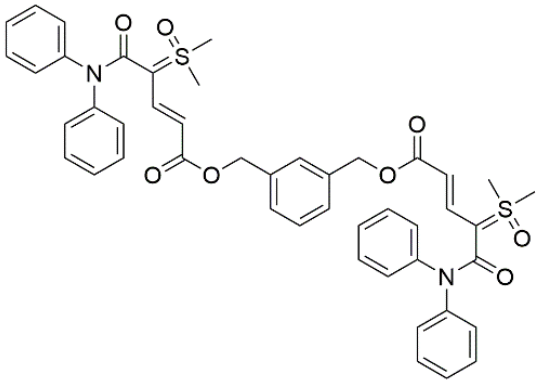

The title compound was prepared according to the general procedure and purified by column chromatography on silica gel and eluted with dichloromethane/ethyl acetate (1/2) to afford a yellow oil in 61% yield (49.8 mg). ^{1}H NMR (600 MHz, $CDCl_3$): δ 7.31–7.26 (m, 10H), 7.20 (d, *J* = 7.6 Hz, 2H), 7.18–7.11 (m, 14H), 5.48 (d, *J* = 15.4 Hz, 2H), 5.04 (s, 4H), 3.54 (s, 12H). ^{13}C NMR (150 MHz, $CDCl_3$): δ 168.3, 168.0, 144.1, 137.2, 135.6, 129.0, 128.3, 127.1, 127.0, 126.3, 125.5, 102.1, 78.5, 64.7, 43.5. HRMS (ESI-TOF) *m/z* calcd. for $C_{46}H_{45}N_2O_8S_2^+$ ([M+H]$^+$) 817.2612. found, 817.2605.

ethyl 5-(diphenylamino)-5-oxopentanoate (94)

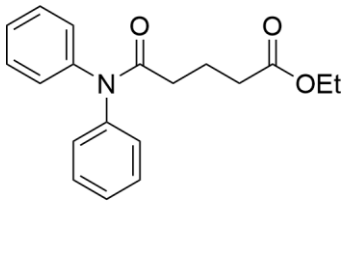

The title compound was prepared according to the general procedure and purified by column chromatography on silica gel and eluted with petroleum ether/ethyl acetate (5/1) to afford a yellow oil in 87% yield (27.1 mg). ^{1}H NMR (600 MHz, $CDCl_3$): δ 7.35 (s, 5H), 7.25 (d, *J* = 7.3 Hz, 5H), 4.06 (q, *J* = 7.1 Hz, 2H), 2.34 (t, *J* = 7.3 Hz, 2H), 2.31 (t, *J* = 7.2 Hz, 2H), 1.97 (p, *J* = 7.3 Hz, 2H), 1.19 (t, *J* = 7.1 Hz, 3H). ^{13}C NMR (150 MHz, $CDCl_3$): δ 173.1, 172.2, 142.6, 129.8, 128.9, 128.6, 127.8, 126.4, 60.2, 34.1, 33.4, 20.6, 14.1. HRMS (ESI-TOF) *m/z* calcd. for $C_{19}H_{22}NO_3^+$ ([M+H]$^+$) 312.1594. found, 312.1598.

ethyl 5-oxo-5-(10*H*-phenothiazin-10-yl)pentanoate (95)

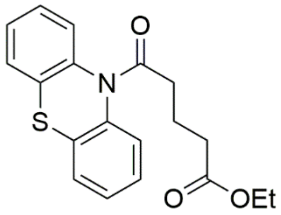

The title compound was prepared according to the general procedure and purified by column chromatography on silica gel and eluted with petroleum ether/dichloromethane (1/1) to afford a yellow oil in 81% yield (27.6 mg). ^{1}H NMR (600 MHz, $CDCl_3$): δ 7.49 (d, J = 7.3 Hz, 2H), 7.43 (dd, J = 7.8, 1.3 Hz, 2H), 7.32–7.30 (m, 2H), 7.23–7.20 (m, 2H), 4.06 (q, J = 7.1 Hz, 2H), 2.51 (s, 2H), 2.31 (t, J = 7.3 Hz, 2H), 1.94 (q, J = 7.2 Hz, 2H), 1.19 (t, J = 7.1 Hz, 3H). ^{13}C NMR (150 MHz, $CDCl_3$): δ 173.0, 171.3, 138.6, 133.2, 127.9, 127.2, 126.9, 126.8, 60.2, 33.2, 33.1, 20.5, 14.1. HRMS (ESI-TOF) *m/z* calcd. for $C_{19}H_{20}NO_3S^+$ ($[M+H]^+$) 342.1158. found, 342.1153.

5-oxo-*N*,*N*-diphenylhexanamide (96)

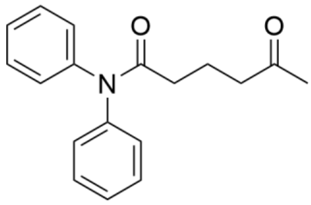

The title compound was prepared according to the general procedure and purified by column chromatography on silica gel and eluted with petroleum ether/ethyl acetate (5/1) to afford a yellow oil in 85% yield (23.9 mg). ^{1}H NMR (600 MHz, $CDCl_3$): δ 7.35 (s, 5H), 7.27–7.23 (m, 5H), 2.50 (t, J = 7.2 Hz, 2H), 2.28 (t, J = 7.1 Hz, 2H), 2.10 (s, 3H), 1.90 (p, J = 7.1 Hz, 2H). ^{13}C NMR (150 MHz, $CDCl_3$): δ 208.6, 172.5, 142.7, 129.8, 129.1, 128.8, 128.6, 127.9, 126.3, 42.7, 34.1, 29.8, 19.4. HRMS (ESI-TOF) *m/z* calcd. for $C_{18}H_{20}NO_2^+$ ($[M+H]^+$) 282.1489. found, 282.1485.

ethyl 5-(4-(2-((2,4-dimethylphenyl)thio)phenyl)piperazin-1-yl)-5-oxopentanoate (98)

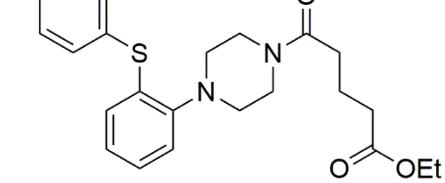

The title compound was prepared according to the general procedure and purified by column chromatography on silica gel and eluted with petroleum ether/ethyl acetate (3/1) to afford a yellow oil in 75% yield (33.0 mg). ^{1}H NMR (600 MHz, $CDCl_3$): δ 7.35 (d, *J* = 7.8 Hz, 1H), 7.15 (s, 1H), 7.09–7.05 (m, 1H), 7.04–6.98 (m, 2H), 6.90–6.86 (m, 1H), 6.53 (d, *J* = 7.9 Hz, 1H), 4.13 (q, *J* = 7.1 Hz, 2H), 3.80 (s, 2H), 3.68–3.60 (m, 2H), 3.06–3.01 (m, 4H), 2.47–2.39 (m, 4H), 2.35 (s, 3H), 2.31 (s, 3H), 1.99 (p, *J* = 7.1 Hz, 2H), 1.26 (t, *J* = 7.1 Hz, 3H). ^{13}C NMR (150 MHz, $CDCl_3$): δ 173.4, 170.9, 148.5, 142.3, 139.3, 136.1, 134.6, 131.7, 127.8, 127.6, 126.3, 125.5, 124.8, 119.9, 60.4, 52.0, 51.4, 46.0, 42.0, 33.5, 32.3, 21.2, 20.6, 20.5, 14.2. HRMS (ESI-TOF) *m/z* calcd. for $C_{25}H_{33}N_2O_3S^+$ ([M+H]$^+$) 441.2206. found, 441.2213.

ethyl 5-(diphenylcarbamoyl)-1-phenyl-1*H*-pyrazole-3-carboxylate (100)

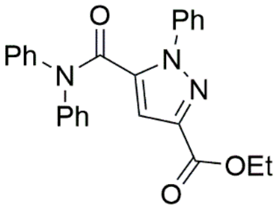

The title compound was prepared according to the general procedure and purified by column chromatography on silica gel and eluted with petroleum ether/ethyl acetate (5/1) to afford a yellow oil in 61% yield (25.1 mg). ^{1}H NMR (600 MHz, $CDCl_3$): δ 7.38 (d, *J* = 7.5 Hz, 1H), 7.34 (t, *J* = 7.3 Hz, 6H), 7.27–7.21 (m, 6H), 7.12 (s, 1H), 7.06 (d, *J* = 7.1 Hz, 2H), 4.21 (q, *J* = 7.1 Hz, 2H), 1.22 (t, *J* = 7.1 Hz, 3H). ^{13}C NMR (150 MHz, $CDCl_3$): δ 163.0, 158.6, 147.0, 143.43, 139.6, 133.5, 129.1, 128.8, 128.3, 127.5, 126.6, 125.7, 114.7, 61.4, 13.9. HRMS (ESI-TOF) *m/z* calcd. for $C_{25}H_{22}N_3O_3^+$ ([M+H]$^+$) 412.1656. found, 412.1658.

ethyl (*E*)-4-chloro-5-(methyl(phenyl)amino)-5-oxopent-2-enoate (101)

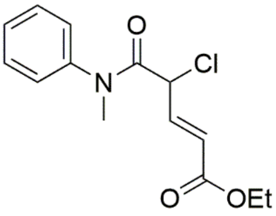

The title compound was prepared according to the general procedure and purified by column chromatography on silica gel and eluted with petroleum ether/ethyl acetate (5/1) to afford a white solid in 79% yield (22.2 mg). ^{1}H NMR (600 MHz, $CDCl_3$): δ 7.46 (t, *J* = 7.6 Hz, 2H), 7.40 (t, *J* = 7.4 Hz, 1H), 7.33 (d, *J* = 7.4 Hz, 2H), 6.61–6.45 (m, 1H),

5.98 (d, J = 9.5 Hz, 1H), 5.86 (d, J = 11.3 Hz, 1H), 4.12–3.92 (m, 2H), 3.30 (s, 3H), 1.17 (t, J = 7.1 Hz, 3H). ^{13}C NMR (150 MHz, $CDCl_3$): δ 167.3, 164.8, 142.2, 142.0, 129.8, 128.5, 127.6, 122.1, 60.5, 48.4, 38.2, 14.0. HRMS (ESI-TOF) *m/z* calcd. for $C_{14}H_{17}ClNO_3^+$ ([M+H]$^+$) 282.0891. found, 282.0887.

4-(3-hydroxybutyl)phenyl (*E*)-4-(dimethyl(oxo)-λ^6-sulfaneylidene)-5-(diphenylamino)-5-oxopent-2-enoate (102)

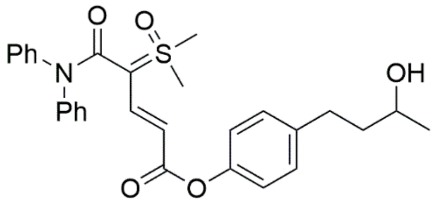

The title compound was prepared according to the general procedure and purified by column chromatography on silica gel and eluted with petroleum ether/ethyl acetate (1/1) to afford a yellow oil in 92% yield (46.5 mg). ^{1}H NMR (600 MHz, $CDCl_3$): δ 7.37 (d, J = 15.3 Hz, 1H), 7.31 (t, J = 7.9 Hz, 4H), 7.14 (td, J = 16.2, 7.9 Hz, 8H), 6.87 (d, J = 8.4 Hz, 2H), 5.57 (d, J = 15.3 Hz, 1H), 3.80–3.71 (m, 1H), 3.55 (s, 6H), 2.69 (ddd, J = 15.3, 9.6, 6.0 Hz, 1H), 2.63–2.57 (m, 1H), 1.85 (s, 1H), 1.77–1.63 (m, 2H), 1.18 (d, J = 6.2 Hz, 3H). ^{13}C NMR (150 MHz, $CDCl_3$): δ 168.2, 166.8, 149.3, 144.0, 138.5, 137.1, 129.0, 128.9, 126.4, 125.7, 121.6, 101.0, 79.1, 67.2, 43.6, 40.7, 31.4, 23.5. HRMS (ESI-TOF) *m/z* calcd. for $C_{29}H_{32}NO_5S^+$ ([M+H]$^+$) 506.1996. found, 506.1987.

ethyl 5-(4-(2-((2,4-dimethylphenyl)thio)phenyl)piperazin-1-yl)-5-oxopentanoate (103)

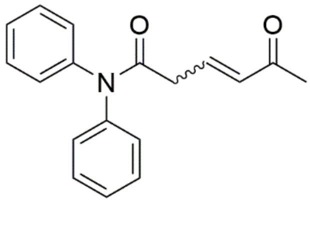

E/*Z* = 1:1

The title compound was prepared according to the general procedure and purified by column chromatography on silica gel and eluted with petroleum ether/ethyl acetate (3/1) to afford a colorless oil in 91% yield (25.4 mg). ^{1}H NMR (600 MHz, $CDCl_3$): δ 7.35 (s, 6H), 7.26 (d, J = 9.8 Hz, 2H), 7.22 (d, J = 7.5 Hz, 2H), 7.06 (dt, J = 15.0, 7.4

Hz, 0.5H), 6.95 (dt, J = 16.2, 6.9 Hz, 0.5H), 5.97 (d, J = 10.4 Hz, 0.5H), 5.94 (d, J = 9.4 Hz, 0.5H), 3.23 (dd, J = 6.9, 1.5 Hz, 1H), 3.22–3.20 (m, 1H), 2.26 (s, 1.5H), 2.14 (s, 1.5H). ^{13}C NMR (150 MHz, $CDCl_3$): δ 204.6, 198.4, 169.2, 165.1, 142.7, 142.4, 142.2, 140.8, 137.5, 133.7, 130.0, 129.2, 129.0, 128.6, 126.5, 126.1, 46.8, 38.7, 26.5, 18.3. HRMS (ESI-TOF) *m/z* calcd. for $C_{18}H_{18}NO_2^+$ ([M+H]$^+$) 280.1332. found, 280.1330.

6-methyl-*N*,*N*,2-triphenyl-2,3-dihydropyridazine-3-carboxamide (104)

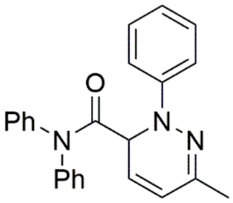

The title compound was prepared according to the general procedure and purified by column chromatography on silica gel and eluted with petroleum ether /ethyl acetate (10/1) to afford a red oil in 41% yield (15.0 mg). ^{1}H NMR (600 MHz, $CDCl_3$): δ 7.95 (dd, J = 14.7, 12.3 Hz, 1H), 7.77 (d, J = 7.1 Hz, 2H), 7.50–7.36 (m, 8H), 7.29–7.26 (m, 4H), 7.17 (d, J = 12.3 Hz, 1H), 6.31 (d, J = 14.7 Hz, 1H), 3.66 (d, J = 4.0 Hz, 1H), 2.18 (s, 3H). ^{13}C NMR (150 MHz, $CDCl_3$): δ 165.8, 157.9, 152.7, 138.1, 137.4, 130.9, 129.0, 128.9, 127.7, 122.7, 122.0, 114.3, 56.3, 21.2. HRMS (ESI-TOF) *m/z* calcd. for $C_{24}H_{22}N_3O^+$ ([M+H]$^+$) 368.1757. found, 368.1762.

(*E*)-2,5-dihydroxy-5-methyl-*N*,*N*-diphenylocta-3,7-dienamide (105)

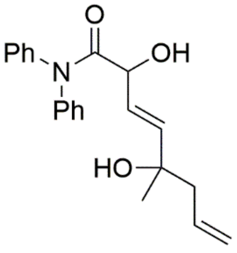

The title compound was prepared according to the general procedure and purified by column chromatography on silica gel and eluted with petroleum ether /ethyl acetate (5/1) to afford a red oil in 66% yield (22.2 mg). ^{1}H NMR (600 MHz, $CDCl_3$): δ 7.35 (s, 4H), 7.29–7.16 (m, 6H), 7.05–6.91 (m, 1H), 6.21–6.11 (m, 1H), 5.89–5.76 (m, 1H), 5.17–5.03 (m, 2H), 4.01 (dd, J = 17.1, 5.5 Hz, 1H), 2.94 (s, 1H), 2.58 (s, 1H), 2.33–2.21 (m, 1H), 2.09–2.04 (m, 1H), 1.15 (s, 1.8H), 1.01 (s, 1.2H). ^{13}C NMR (150 MHz, $CDCl_3$): δ 166.0, 143.6, 143.5, 142.4, 133.4, 133.1, 129.1, 126.6,

124.1, 123.9, 120.0, 119.0, 77.5, 76.3, 74.5, 74.3, 43.2, 41.4, 23.4, 21.9. HRMS (ESI-TOF) *m/z* calcd. for $C_{21}H_{24}NO_3^+$ ([M+H]$^+$) 338.1751. found, 338.1757.

1,5-dimethyl-*N*,*N*-diphenyl-1λ^6-thiopyran-2-carboxamide 1-oxide (106)

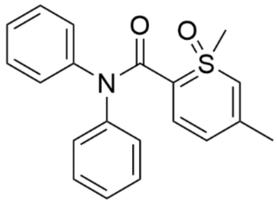

The title compound was prepared according to the general procedure and purified by column chromatography on silica gel and eluted with petroleum ether/ethyl acetate (1/1) to afford a white solid in 79% yield (26.6 mg). ^{1}H NMR (600 MHz, $CDCl_3$): δ 7.31 (t, J = 7.7 Hz, 4H), 7.20–7.16 (m, 6H), 6.71 (d, J = 9.2 Hz, 1H), 5.70 (s, 1H), 5.22 (d, J = 9.2 Hz, 1H), 3.88 (s, 3H), 2.09 (s, 3H). ^{13}C NMR (150 MHz, $CDCl_3$): δ 168.2, 147.2, 144.5, 132.3, 129.2, 127.0, 125.8, 103.5, 95.3, 92.9, 49.4, 22.9. HRMS (ESI-TOF) *m/z* calcd. for $C_{20}H_{20}NO_2S^+$ ([M+H]$^+$) 338.1209. found, 338.1215.

1-methyl-*N*,*N*,5-triphenyl-1λ^6-thiopyran-2-carboxamide 1-oxide (107)

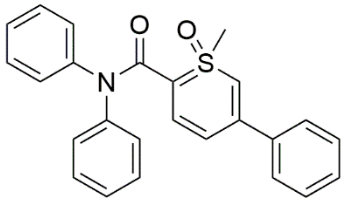

The title compound was prepared according to the general procedure and purified by column chromatography on silica gel and eluted with petroleum ether/ethyl acetate (2/1) to afford a yellow oil in 72% yield (28.7 mg). ^{1}H NMR (600 MHz, $CDCl_3$): δ 7.46 (dd, J = 6.7, 3.0 Hz, 2H), 7.39–7.36 (m, 3H), 7.36–7.31 (m, 4H), 7.26–7.22 (m, 4H), 7.20 (t, J = 7.4 Hz, 2H), 6.91 (d, J = 9.3 Hz, 1H), 6.03 (s, 1H), 5.67 (dd, J = 9.3, 1.5 Hz, 1H), 3.98 (s, 3H). ^{13}C NMR (150 MHz, $CDCl_3$): δ 167.9, 148.1, 144.5, 139.0, 132.9, 129.3, 129.2, 128.7, 127.2, 127.0, 126.0, 101.9, 94.4, 93.0, 49.6. HRMS (ESI-TOF) *m/z* calcd. for $C_{25}H_{22}NO_2S^+$ ([M+H]$^+$) 400.1366. found, 400.1371.

1,5-dimethyl-*N*,*N*-di-p-tolyl-1λ⁶-thiopyran-2-carboxamide 1-oxide (108)

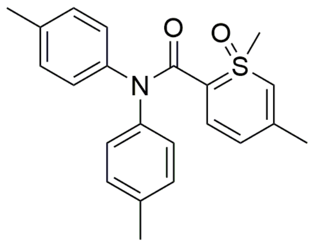

The title compound was prepared according to the general procedure and purified by column chromatography on silica gel and eluted with petroleum ether/ethyl acetate (2/1) to afford a yellow solid in 74% yield (27.0 mg). ^{1}H NMR (600 MHz, $CDCl_3$): δ 7.10 (d, *J* = 8.3 Hz, 4H), 7.08–7.05 (m, 4H), 6.72 (d, *J* = 9.2 Hz, 1H), 5.67 (s, 1H), 5.23 (dd, *J* = 9.2, 1.3 Hz, 1H), 3.87 (s, 3H), 2.31 (s, 6H), 2.10 (s, 3H). ^{13}C NMR (150 MHz, $CDCl_3$): δ 168.0, 146.9, 142.1, 135.6, 132.4, 129.8, 126.7, 103.3, 95.0, 93.1, 49.7, 23.0, 21.0. HRMS (ESI-TOF) *m/z* calcd. for $C_{22}H_{24}NO_2S^+$ ([M+H]$^+$) 366.1522. found, 366.1517.

***N*,*N*-bis(4-bromophenyl)-1,5-dimethyl-1λ⁶-thiopyran-2-carboxamide 1-oxide (109)**

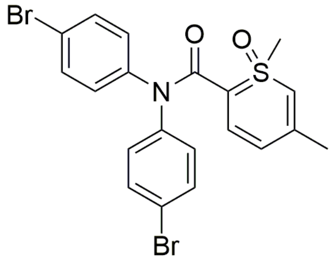

The title compound was prepared according to the general procedure and purified by column chromatography on silica gel and eluted with petroleum ether/ethyl acetate (2/1) to afford a yellow solid in 46% yield (22.6 mg). ^{1}H NMR (600 MHz, $CDCl_3$): δ 7.45–7.40 (m, 4H), 7.05–7.02 (m, 4H), 6.67 (d, *J* = 9.2 Hz, 1H), 5.73 (s, 1H), 5.29 (dd, *J* = 9.3, 1.3 Hz, 1H), 3.86 (s, 3H), 2.12 (s, 3H). ^{13}C NMR (150 MHz, $CDCl_3$): δ 168.1, 147.8, 143.3, 132.5, 132.0, 128.4, 119.5, 104.1, 95.9, 92.2, 49.3, 23.0. HRMS (ESI-TOF) *m/z* calcd. for $C_{20}H_{18}Br_2NO_2S^+$ ([M+H]$^+$) 493.9420. found, 493.9413.

(1,5-dimethyl-1-oxido-1λ⁶-thiopyran-2-yl)(1-methyl-1*H*-indol-3-yl)methanone (110)

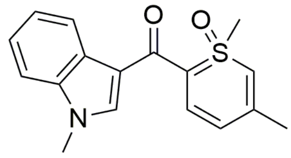

The title compound was prepared according to the general procedure and purified by column chromatography on silica gel and eluted with petroleum ether/ethyl acetate (2/1) to afford a yellow solid in 71% yield (21.2 mg). ^{1}H NMR (600 MHz, $CDCl_3$): δ 8.17 (d, *J* = 8.0 Hz, 1H), 7.50 (d, *J* = 9.4 Hz, 2H), 7.37–7.33 (m, 1H), 7.33–7.29 (m, 1H), 7.28–7.21 (m, 1H), 5.89 (s, 1H), 5.67 (dd, *J* = 9.1, 1.3 Hz, 1H), 3.95 (s, 3H), 3.84 (s, 3H), 2.24 (s, 3H). ^{13}C NMR (150 MHz, $CDCl_3$): δ 184.9, 148.0, 137.1, 134.5, 132.9, 127.1, 123.2, 122.1, 121.9, 114.9, 109.5, 105.0, 101.3, 97.1, 46.2, 33.4, 23.0. HRMS (ESI-TOF) *m/z* calcd. for $C_{17}H_{18}NO_2S^+$ ($[M+H]^+$) 300.1053. found, 300.1061.

9. Characterization Data for Amide-Sulfoxonium Ylides

N,*N*-bis(4-bromophenyl)-2-(dimethyl(oxo)-λ^6-sulfaneylidene)acetamide (S4)

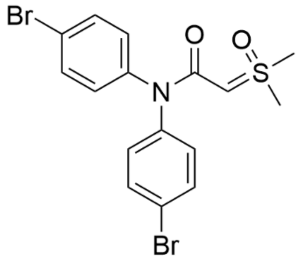

The title compound was prepared according to the general procedure and purified by column chromatography on silica gel and eluted with ethyl acetate/methanol (20/1) to afford a yellow solid in 73% yield (1.625 g). ^{1}H NMR (600 MHz, CDCl$_3$): δ 7.42 (d, *J* = 8.7 Hz, 4H), 7.11 (d, *J* = 8.7 Hz, 4H), 3.55 (s, 1H), 3.38 (s, 6H). ^{13}C NMR (150 MHz, CDCl$_3$): δ 166.7, 142.3, 132.1, 129.2, 119.3, 57.9, 43.0. HRMS (ESI-TOF) *m/z* calcd. for $C_{16}H_{15}Br_2NNaO_2S^+$ ([M+Na]$^+$) 465.9082. found, 465.9085.

2-(dimethyl(oxo)-λ^6-sulfaneylidene)-*N*,*N*-bis(4-(trifluoromethyl)phenyl)acetamide (S5)

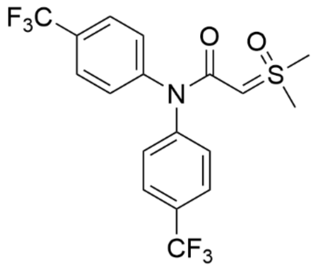

The title compound was prepared according to the general procedure and purified by column chromatography on silica gel and eluted with ethyl acetate/methanol (20/1) to afford a yellow solid in 68% yield (1.440 g). ^{1}H NMR (600 MHz, CDCl$_3$): δ 7.57 (d, *J* = 8.1 Hz, 4H), 7.34 (d, *J* = 8.1 Hz, 4H), 3.60 (s, 1H), 3.40 (s, 6H). ^{13}C NMR (150 MHz, CDCl$_3$): δ 166.6, 146.3, 127.62 (q, *J* = 32.8 Hz), 127.57, 126.2 (q, *J* = 3.6 Hz), 123.9 (q, *J* = 271.9 Hz), 58.9, 42.8. ^{19}F NMR (564 MHz, CDCl$_3$): δ -62.3 (s, 6F). HRMS (ESI-TOF) *m/z* calcd. for $C_{18}H_{15}F_6NNaO_2S^+$ ([M+Na]$^+$) 446.0620. found, 446.0622.

2-(dimethyl(oxo)-λ⁶-sulfaneylidene)-*N*-phenyl-*N*-(m-tolyl)acetamide (S7)

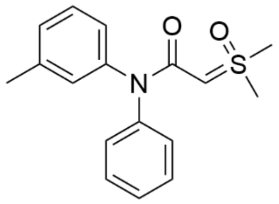

The title compound was prepared according to the general procedure and purified by column chromatography on silica gel and eluted with ethyl acetate/methanol (10/1) to afford a yellow solid in 67% yield (1.010 g). ^{1}H NMR (600 MHz, $CDCl_3$): δ 7.21–7.28 (m, 4H), 7.19 (t, J = 7.7 Hz, 1H), 7.15 (t, J = 7.0 Hz, 1H), 7.10–7.05 (m, 2H), 6.99 (d, J = 7.5 Hz, 1H), 3.55 (s, 1H), 3.37 (s, 6H), 2.30 (s, 3H). ^{13}C NMR (150 MHz, $CDCl_3$): δ 167.2, 143.6, 143.5, 138.8, 128.8, 128.7, 128.5, 127.6, 126.7, 125.5, 125.1, 56.9, 43.1, 21.3. HRMS (ESI-TOF) *m/z* calcd. for $C_{17}H_{19}NNaO_2S^+$ ([M+Na]$^+$) 324.1029. found, 324.1033.

2-(dimethyl(oxo)-λ⁶-sulfaneylidene)-*N*-phenyl-*N*-(o-tolyl)acetamide (S8)

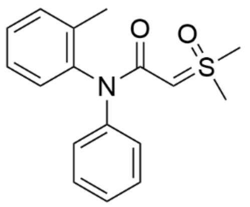

The title compound was prepared according to the general procedure and purified by column chromatography on silica gel and eluted with ethyl acetate/methanol (10/1) to afford a yellow solid in 67% yield (1.010 g). ^{1}H NMR (600 MHz, $CDCl_3$): δ 7.25–7.23 (m, 6H), 7.22–7.19 (m, 2H), 7.06–7.03 (m, 1H), 3.45 (s, 1H), 3.37 (s, 6H), 2.24 (s, 3H). ^{13}C NMR (150 MHz, $CDCl_3$): δ 167.0, 142.6, 142.0, 137.1, 131.3, 130.1, 128.4, 127.6, 127.0, 125.3, 124.1, 56.8, 43.1, 18.1. HRMS (ESI-TOF) *m/z* calcd. for $C_{17}H_{19}NNaO_2S^+$ ([M+Na]$^+$) 324.1029. found, 324.1033.

2-(dimethyl(oxo)-λ⁶-sulfaneylidene)-*N*-(4-fluorophenyl)-*N*-phenylacetamide (S9)

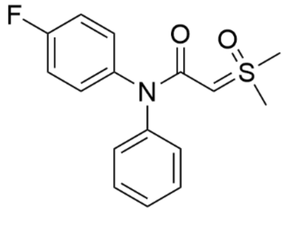

The title compound was prepared according to the general procedure and purified by column chromatography on

silica gel and eluted with ethyl acetate/methanol (20/1) to afford a yellow solid in 71% yield (1.084 g). ^{1}H NMR (600 MHz, $CDCl_3$): δ 7.33–7.29 (m, 2H), 7.25–7.21 (m, 4H), 7.19–7.14 (m, 1H), 6.99 (t, J = 8.7 Hz, 2H), 3.54 (s, 1H), 3.37 (s, 6H). ^{13}C NMR (150 MHz, $CDCl_3$): δ 167.2, 160.4 (d, J = 245.3 Hz), 143.5, 139.5, 129.3 (d, J = 8.3 Hz), 129.0, 127.7, 125.8, 115.6 (d, J = 22.5 Hz), 57.2, 43.0. ^{19}F NMR (564 MHz, $CDCl_3$): δ -116.6 (s, 1F). HRMS (ESI-TOF) *m/z* calcd. for $C_{16}H_{16}FNNaO_2S^+$ ([M+Na]$^+$) 328.0778. found, 328.0783.

(*E*)-2-(dimethyl(oxo)-λ^6-sulfaneylidene)-*N*-phenyl-*N*-(4-(phenyldiazenyl)phenyl)acetamide (S10)

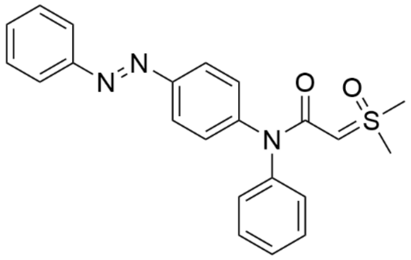

The title compound was prepared according to the general procedure and purified by column chromatography on silica gel and eluted with ethyl acetate/methanol (10/1) to afford a yellow solid in 57% yield (1.116 g). ^{1}H NMR (600 MHz, $CDCl_3$): δ 7.89–7.85 (m, 4H), 7.50 (t, J = 7.1 Hz, 2H), 7.45 (d, J = 6.7 Hz, 1H), 7.41–7.36 (m, 4H), 7.32–7.24 (m, 3H), 3.63 (s, 1H), 3.41 (s, 6H). ^{13}C NMR (150 MHz, $CDCl_3$): δ 167.1, 152.7, 149.3, 146.2, 143.3, 130.7, 129.3, 129.0, 128.5, 126.8, 126.5, 123.3, 122.7, 58.2, 43.0. HRMS (ESI-TOF) *m/z* calcd. for $C_{22}H_{21}N_3NaO_2S^+$ ([M+Na]$^+$) 414.1247. found, 414.1243.

2-(dimethyl(oxo)-λ^6-sulfaneylidene)-*N*-(3,4-dimethylphenyl)-*N*-phenylacetamide (S11)

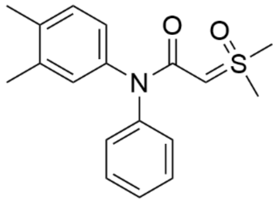

The title compound was prepared according to the general procedure and purified by column chromatography on silica gel and eluted with ethyl acetate/methanol (10/1) to afford a yellow solid in 63% yield (0.994 g). ^{1}H NMR (600 MHz, $CDCl_3$): δ 7.28–7.23 (m, 4H), 7.12–7.10 (m, 1H), 7.06 (d, J = 8.0 Hz, 1H), 7.03 (s, 1H), 6.99 (d, J = 9.8 Hz, 1H), 3.54 (s, 1H), 3.35 (s, 6H), 2.21 (s, 3H), 2.19 (s, 3H). ^{13}C NMR (150 MHz, $CDCl_3$): δ 167.3, 143.7, 141.2, 137.3, 134.5, 130.0, 129.2, 128.7, 127.2, 125.6, 125.1, 56.8, 43.0, 19.8, 19.2. HRMS (ESI-TOF) *m/z* calcd. for

$C_{18}H_{21}NNaO_2S^+$ ([M+Na]$^+$) 338.1185. found, 338.1188.

N,*N*-di([1,1'-biphenyl]-4-yl)-2-(dimethyl(oxo)-λ^6-sulfaneylidene)acetamide (S13)

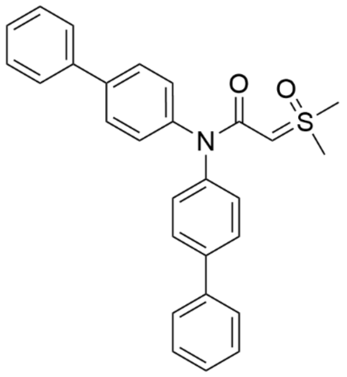

The title compound was prepared according to the general procedure and purified by column chromatography on silica gel and eluted with ethyl acetate/methanol (10/1) to afford a yellow solid in 69% yield (1.517 g). ^{1}H NMR (600 MHz, $CDCl_3$): δ 7.56 (t, J = 7.6 Hz, 8H), 7.43 (t, J = 7.7 Hz, 4H), 7.38 (d, J = 8.5 Hz, 4H), 7.33 (t, J = 7.4 Hz, 2H), 3.67 (s, 1H), 3.43 (s, 6H). ^{13}C NMR (150 MHz, $CDCl_3$): δ 167.2, 142.8, 140.5, 138.6, 128.8, 128.0, 127.7, 127.2, 127.0, 57.2, 43.2. HRMS (ESI-TOF) *m/z* calcd. for $C_{28}H_{25}NNaO_2S^+$ ([M+Na]$^+$) 462.1498. found, 462.1499.

1-(2-chloro-10*H*-phenothiazin-10-yl)-2-(dimethyl(oxo)-λ^6-sulfaneylidene)ethan-1-one (S18)

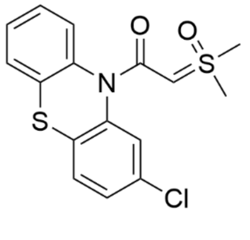

The title compound was prepared according to the general procedure and purified by column chromatography on silica gel and eluted with ethyl acetate/methanol (10/1) to afford a yellow solid in 51% yield (0.898 g). ^{1}H NMR (600 MHz, $CDCl_3$): δ 7.61 (s, 1H), 7.53 (d, J = 7.9 Hz, 1H), 7.32 (d, J = 7.7 Hz, 1H), 7.25–7.20 (m, 2H), 7.14 (t, J = 7.5 Hz, 1H), 7.10 (d, J = 8.3 Hz, 1H), 3.98 (s, 1H), 3.33 (s, 6H). ^{13}C NMR (150 MHz, $CDCl_3$): δ 165.1, 140.3, 139.0, 132.8, 132.3, 131.4, 128.2, 127.9, 127.8, 127.6, 126.8, 126.3, 126.1, 56.2, 42.8. HRMS (ESI-TOF) *m/z* calcd. for $C_{16}H_{14}ClNNaO_2S_2^+$ ([M+Na]$^+$) 374.0047. found, 374.0050.

1-(9*H*-carbazol-9-yl)-2-(dimethyl(oxo)-λ^6-sulfaneylidene)ethan-1-one (S19)[4]

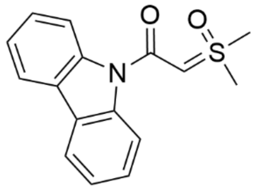

The title compound was prepared according to the general procedure and purified by column chromatography on silica gel and eluted with ethyl acetate. ^{1}H NMR (600 MHz, DMSO-d_6): δ 8.16 (d, *J* = 4.2 Hz, 2H), 8.14 (d, *J* = 3.7 Hz, 2H), 7.56–7.38 (m, 2H), 7.31–7.27 (m, 2H), 5.21 (s, 1H), 3.70 (s, 6H). ^{13}C NMR (150 MHz, DMSO-d_6): δ 162.9, 138.3, 126.3, 123.8, 121.1, 120.0, 114.3, 68.5, 40.6.

1-(7-bromo-2,3-dihydro-4*H*-benzo[*b*][1,4]oxazin-4-yl)-2-(dimethyl(oxo)-λ^6-sulfaneylidene)ethan-1-one (S21)

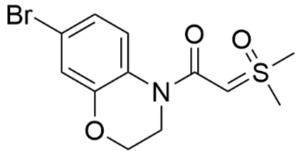

The title compound was prepared according to the general procedure and purified by column chromatography on silica gel and eluted with ethyl acetate/methanol (20/1) to afford a white solid in 43% yield (0.715 g). ^{1}H NMR (600 MHz, $CDCl_3$): δ 7.41 (d, *J* = 8.7 Hz, 1H), 6.99 (s, 1H), 6.92 (d, *J* = 8.7 Hz, 1H), 4.25 (s, 1H), 4.20–4.15 (m, 2H), 3.80–3.75 (m, 2H), 3.39 (s, 6H). ^{13}C NMR (150 MHz, $CDCl_3$): δ 165.8, 147.1, 126.7, 125.0, 122.7, 119.9, 116.3, 66.2, 57.8, 42.8, 39.8. HRMS (ESI-TOF) *m/z* calcd. for $C_{12}H_{14}BrNNaO_3S^+$ ($[M+Na]^+$) 353.9770. found, 353.9768.

1-(2-(dimethyl(oxo)-λ^6-sulfaneylidene)acetyl)-1,2,3,4-tetrahydro-5*H*-benzo[*b*]azepin-5-one (S22)

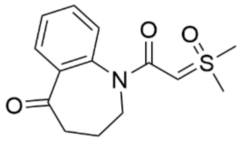

The title compound was prepared according to the general procedure and purified by column chromatography on silica gel and eluted with ethyl acetate/methanol (10/1) to afford a yellow solid in 41% yield (0.571 g). ^{1}H NMR (600 MHz, $CDCl_3$): δ 7.85 (d, *J* = 7.8 Hz, 1H), 7.49 (t, *J* = 7.5 Hz, 1H), 7.41 (d, *J* = 7.9 Hz, 1H), 7.32 (t, *J* = 7.4 Hz,

1H), 3.75 (s, 1H), 3.36 (s, 6H), 2.67 (t, J = 6.2 Hz, 2H), 2.60 (s, 1H), 2.04 (s, 2H), 1.80 (s, 1H). ^{13}C NMR (150 MHz, $CDCl_3$): δ 202.3, 167.1, 143.9, 134.9, 133.0, 129.5, 129.0, 126.9, 56.2, 45.1, 43.0, 40.9, 40.2. HRMS (ESI-TOF) *m/z* calcd. for $C_{14}H_{17}NNaO_3S^+$ ($[M+Na]^+$) 302.0821. found, 302.0816.

2-(dimethyl(oxo)-λ^6-sulfaneylidene)-1-(4-phenyl-3,6-dihydropyridin-1(2*H*)-yl)ethan-1-one (S23)

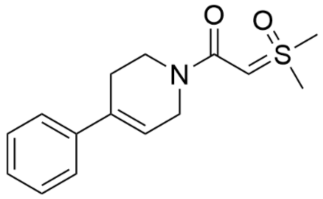

The title compound was prepared according to the general procedure and purified by column chromatography on silica gel and eluted with ethyl acetate/methanol (10/1) to afford a yellow solid in 20% yield (0.277 g). ^{1}H NMR (600 MHz, $CDCl_3$): δ 7.37 (d, J = 7.6 Hz, 2H), 7.32 (t, J = 7.6 Hz, 2H), 7.24 (d, J = 7.1 Hz, 1H), 6.04 (s, 1H), 4.03 (s, 2H), 3.88 (s, 1H), 3.63 (s, 2H), 3.43 (s, 6H), 2.54 (s, 2H). ^{13}C NMR (150 MHz, $CDCl_3$): δ 167.4, 140.7, 135.8, 128.4, 127.2, 124.9, 120.8, 54.1, 43.5, 41.0, 39.4, 27.6. HRMS (ESI-TOF) *m/z* calcd. for $C_{15}H_{19}NNaO_2S^+$ ($[M+Na]^+$) 300.1029. found, 300.1035.

***N*-allyl-2-(dimethyl(oxo)-λ^6-sulfaneylidene)-*N*-phenylacetamide (S25)**

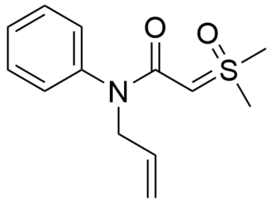

The title compound was prepared according to the general procedure and purified by column chromatography on silica gel and eluted with ethyl acetate/methanol (10/1) to afford a yellow solid in 36% yield (0.451 g). ^{1}H NMR (600 MHz, $CDCl_3$): δ 7.35 (t, J = 7.6 Hz, 2H), 7.24 (dd, J = 15.0, 7.6 Hz, 3H), 5.92–5.85 (m, 1H), 5.11–5.02 (m, 2H), 4.29 (d, J = 5.9 Hz, 2H), 3.46 (s, 1H), 3.35 (s, 6H). ^{13}C NMR (150 MHz, $CDCl_3$): δ 167.4, 143.6, 134.9, 129.2, 128.5, 126.8, 116.5, 55.6, 50.8, 43.2. HRMS (ESI-TOF) *m/z* calcd. for $C_{13}H_{17}NNaO_2S^+$ ($[M+Na]^+$) 274.0872. found, 274.0867.

N-(2-cyanoethyl)-2-(dimethyl(oxo)-λ⁶-sulfaneylidene)-N-phenylacetamide (S26)

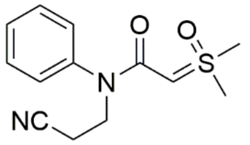

The title compound was prepared according to the general procedure and purified by column chromatography on silica gel and eluted with ethyl acetate/methanol (10/1) to afford a yellow solid in 61% yield (0.806 g). ^{1}H NMR (600 MHz, DMSO-d_6): δ 7.41 (t, J = 7.6 Hz, 2H), 7.29 (t, J = 7.3 Hz, 1H), 7.26 (d, J = 7.7 Hz, 2H), 3.82 (t, J = 6.6 Hz, 2H), 3.57 (s, 1H), 3.34 (s, 6H), 2.64 (t, J = 6.6 Hz, 2H). ^{13}C NMR (150 MHz, DMSO-d_6): δ 166.7, 143.1, 129.4, 128.4, 126.8, 119.3, 58.7, 43.4, 41.2, 17.0. HRMS (ESI-TOF) *m/z* calcd. for $C_{13}H_{16}N_2NaO_2S^+$ ([M+Na]$^+$) 287.0825. found, 287.0828.

2-(dimethyl(oxo)-λ⁶-sulfaneylidene)-N-(thiophen-2-ylmethyl)-N-(p-tolyl)acetamide (S27)

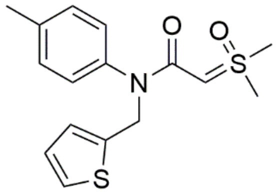

The title compound was prepared according to the general procedure and purified by column chromatography on silica gel and eluted with ethyl acetate/methanol (10/1) to afford a yellow solid in 45% yield (0.724 g). ^{1}H NMR (600 MHz, $CDCl_3$): δ 7.16 (d, J = 6.2 Hz, 1H), 7.09 (d, J = 8.0 Hz, 2H), 6.98 (d, J = 8.2 Hz, 2H), 6.86–6.84 (m, 1H), 6.78 (d, J = 4.2 Hz, 1H), 4.97 (s, 2H), 3.40 (s, 1H), 3.35 (s, 6H), 2.32 (s, 3H). ^{13}C NMR (150 MHz, $CDCl_3$): δ 167.3, 142.1, 140.5, 136.9, 129.8, 128.4, 126.2, 126.1, 125.0, 55.4, 46.4, 43.1, 21.1. HRMS (ESI-TOF) *m/z* calcd. for $C_{16}H_{19}NNaO_2S_2^+$ ([M+Na]$^+$) 344.0749. found, 344.0752.

N-(4-(tert-butyl)phenyl)-2-(dimethyl(oxo)-λ⁶-sulfaneylidene)acetamide (S29)

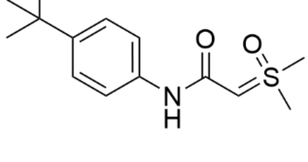

The title compound was prepared according to the general procedure and purified by column chromatography on silica gel and eluted with ethyl acetate/methanol (10/1) to afford a yellow solid in 43% yield (0.574 g). ^{1}H NMR

(600 MHz, DMSO-d_6): δ 8.64 (s, 1H), 7.37 (d, J = 8.7 Hz, 2H), 7.18 (d, J = 8.7 Hz, 2H), 4.14 (s, 1H), 3.43 (s, 6H), 1.24 (s, 9H). ^{13}C NMR (150 MHz, DMSO-d_6): δ 165.8, 142.7, 138.7, 124.9, 117.8, 58.8, 41.3, 33.8, 31.3. HRMS (ESI-TOF) m/z calcd. for $C_{14}H_{21}NNaO_2S^+$ ([M+Na]$^+$) 290.1185. found, 290.1192.

2-(dimethyl(oxo)-λ^6-sulfaneylidene)-*N*-(4-methoxyphenyl)acetamide (S30)

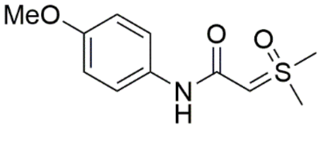

The title compound was prepared according to the general procedure and purified by column chromatography on silica gel and eluted with ethyl acetate/methanol (20/1) to afford a yellow solid in 53% yield (0.640 g). ^{1}H NMR (600 MHz, DMSO-d_6): δ 8.56 (s, 1H), 7.36 (d, J = 9.0 Hz, 2H), 6.76 (d, J = 9.0 Hz, 2H), 4.06 (s, 1H), 3.67 (s, 3H), 3.42 (s, 6H). ^{13}C NMR (150 MHz, DMSO-d_6): δ 165.8, 153.6, 134.6, 119.6, 113.7, 58.5, 55.1, 41.4. HRMS (ESI-TOF) m/z calcd. for $C_{11}H_{15}NNaO_3S^+$ ([M+Na]$^+$) 264.0665. found, 264.0667.

***N*-(3-chlorophenyl)-2-(dimethyl(oxo)-λ^6-sulfaneylidene)acetamide (S31)**

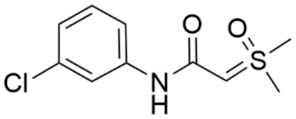

The title compound was prepared according to the general procedure and purified by column chromatography on silica gel and eluted with ethyl acetate/methanol (10/1) to afford a yellow solid in 39% yield (0.479 g). ^{1}H NMR (600 MHz, $CDCl_3$): δ 7.51 (s, 1H), 7.16 (d, J = 5.7 Hz, 2H), 6.97–6.95 (m, 1H), 6.48 (s, 1H), 4.00 (s, 1H), 3.43 (s, 6H). ^{13}C NMR (150 MHz, $CDCl_3$): δ 165.7, 140.6, 134.5, 129.8, 122.7, 119.8, 117.7, 55.9, 43.1. HRMS (ESI-TOF) m/z calcd. for $C_{10}H_{12}ClNNaO_2S^+$ ([M+Na]$^+$) 268.0169. found, 268.0173.

***N*-(2-chlorophenyl)-2-(dimethyl(oxo)-λ^6-sulfaneylidene)acetamide (S32)**

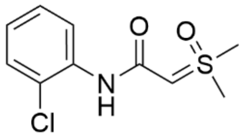

The title compound was prepared according to the general procedure and purified by column chromatography on silica gel and eluted with ethyl acetate/methanol (10/1) to afford a white solid in 37% yield (0.453 g). ^{1}H NMR (600

MHz, $CDCl_3$): δ 8.17 (d, J = 7.6 Hz, 1H), 7.31 (d, J = 7.7 Hz, 1H), 7.19 (t, J = 7.3 Hz, 1H), 6.98 (s, 1H), 6.92 (t, J = 7.3 Hz, 1H), 4.07 (s, 1H), 3.46 (s, 6H). ^{13}C NMR (150 MHz, $CDCl_3$): δ 165.4, 136.0, 128.9, 127.4, 123.0, 122.4, 121.5, 56.8, 42.9. HRMS (ESI-TOF) *m/z* calcd. for $C_{10}H_{12}ClNNaO_2S^+$ ([M+Na]$^+$) 268.0169. found, 268.0175.

2-(dimethyl(oxo)-λ^6-sulfaneylidene)-*N*-(2-iodophenyl)acetamide (S33)

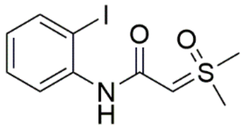

The title compound was prepared according to the general procedure and purified by column chromatography on silica gel and eluted with ethyl acetate/methanol (10/1) to afford a yellow solid in 38% yield (0.641 g). ^{1}H NMR (600 MHz, DMSO-d_6): δ 7.77 (d, J = 9.3 Hz, 1H), 7.71 (d, J = 9.7 Hz, 1H), 7.64 (s, 1H), 7.26 (t, J = 8.4 Hz, 1H), 6.79–6.72 (m, 1H), 4.40 (s, 1H), 3.44 (s, 6H). ^{13}C NMR (150 MHz, DMSO-d_6): δ 165.2, 140.9, 138.6, 128.2, 124.5, 124.1, 92.6, 59.3, 41.2. HRMS (ESI-TOF) *m/z* calcd. for $C_{10}H_{12}INNaO_2S^+$ ([M+Na]$^+$) 359.9526. found, 359.9527.

2-(dimethyl(oxo)-λ^6-sulfaneylidene)-*N*-(4-(trifluoromethoxy)phenyl)acetamide (S34)

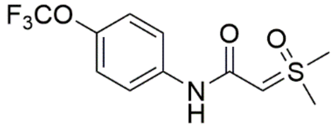

The title compound was prepared according to the general procedure and purified by column chromatography on silica gel and eluted with ethyl acetate/methanol (10/1) to afford a white solid in 41% yield (0.606 g). ^{1}H NMR (600 MHz, DMSO-d_6): δ 8.94 (s, 1H), 7.56 (d, J = 7.8 Hz, 2H), 7.16 (d, J = 8.3 Hz, 2H), 4.15 (s, 1H), 3.45 (s, 6H). ^{13}C NMR (150 MHz, DMSO-d_6): δ 165.7, 141.6, 140.7, 121.3, 120.2 (q, J = 254.8 Hz), 118.7, 59.4, 41.2. ^{19}F NMR (564 MHz, DMSO-d_6): δ -57.1 (s, 3F). HRMS (ESI-TOF) *m/z* calcd. for $C_{11}H_{12}F_3NNaO_3S^+$ ([M+Na]$^+$) 318.0382. found, 318.0385.

2-(dimethyl(oxo)-λ^6-sulfaneylidene)-*N*-(4-vinylphenyl)acetamide (S35)

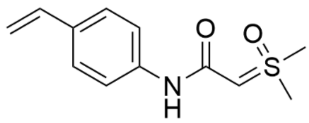

The title compound was prepared according to the general procedure and purified by column chromatography on

silica gel and eluted with ethyl acetate/methanol (10/1) to afford a yellow solid in 45% yield (0.534 g). ^{1}H NMR (600 MHz, DMSO-d_6): δ 8.83 (s, 1H), 7.46 (d, J = 8.1 Hz, 2H), 7.28 (d, J = 8.0 Hz, 2H), 6.63–6.58 (m, 1H), 5.62 (d, J = 17.6 Hz, 1H), 5.06 (d, J = 10.9 Hz, 1H), 4.20 (s, 1H), 3.45 (s, 6H). ^{13}C NMR (150 MHz, DMSO-d_6): δ 165.6, 141.2, 136.5, 129.5, 126.4, 117.7, 111.1, 59.3, 41.2. HRMS (ESI-TOF) *m/z* calcd. for $C_{12}H_{15}NNaO_2S^+$ ([M+Na]$^+$) 260.0716. found, 260.0711.

2-(dimethyl(oxo)-λ^6-sulfaneylidene)-*N*-(2-(phenylethynyl)phenyl)acetamide (S36)

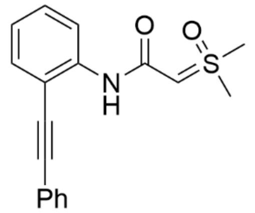

The title compound was prepared according to the general procedure and purified by column chromatography on silica gel and eluted with ethyl acetate/methanol (10/1) to afford a yellow solid in 41% yield (0.637 g). ^{1}H NMR (600 MHz, $CDCl_3$): δ 8.23 (d, J = 8.4 Hz, 1H), 7.54–7.47 (m, 2H), 7.41 (d, J = 7.6 Hz, 1H), 7.37–7.31 (m, 3H), 7.24 (d, J = 7.6 Hz, 2H), 6.91 (t, J = 7.5 Hz, 1H), 3.99 (s, 1H), 3.38 (s, 6H). ^{13}C NMR (150 MHz, $CDCl_3$): δ 165.6, 140.6, 131.6, 131.4, 129.4, 128.5, 128.4, 122.6, 121.5, 118.8, 110.9, 95.5, 85.0, 57.1, 42.6. HRMS (ESI-TOF) *m/z* calcd. for $C_{18}H_{17}NNaO_2S^+$ ([M+Na]$^+$) 334.0872. found, 334.0865.

2-(dimethyl(oxo)-λ^6-sulfaneylidene)-*N*-(2,6-dimethylphenyl)acetamide (S37)

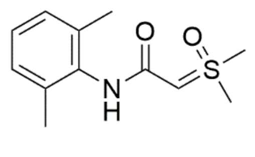

The title compound was prepared according to the general procedure and purified by column chromatography on silica gel and eluted with ethyl acetate/methanol (10/1) to afford a white solid in 37% yield (0.442 g). ^{1}H NMR (600 MHz, DMSO-d_6): δ 7.77 (s, 1H), 7.00 (s, 3H), 3.39 (s, 6H), 2.54 (s, 1H), 2.15 (s, 6H). ^{13}C NMR (150 MHz, DMSO-d_6): δ 165.9, 137.0, 135.7, 127.5, 125.3, 57.0, 41.4, 18.5. HRMS (ESI-TOF) *m/z* calcd. for $C_{12}H_{17}NNaO_2S^+$ ([M+Na]$^+$) 262.0872. found, 262.0865.

2-(dimethyl(oxo)-λ⁶-sulfaneylidene)-*N*-(3,4,5-trimethylphenyl)acetamide (S38)

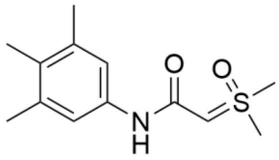

The title compound was prepared according to the general procedure and purified by column chromatography on silica gel and eluted with ethyl acetate/methanol (10/1) to afford a white solid in 90% yield (1.138 g). ^{1}H NMR (600 MHz, $CDCl_3$): δ 6.98 (s, 2H), 6.28 (s, 1H), 3.85 (s, 1H), 3.41 (s, 6H), 2.23 (s, 6H), 2.10 (s, 3H). ^{13}C NMR (150 MHz, $CDCl_3$): δ 166.6, 137.0, 136.2, 130.2, 120.3, 55.1, 43.2, 20.6, 14.8. HRMS (ESI-TOF) *m/z* calcd. for $C_{13}H_{19}NNaO_2S^+$ ($[M+Na]^+$) 276.1029. found, 276.1037.

2-(dimethyl(oxo)-λ⁶-sulfaneylidene)-*N*-methyl-*N*-phenethylacetamide (S39)

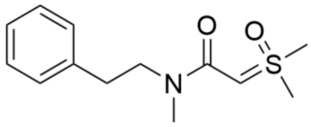

The title compound was prepared according to the general procedure and purified by column chromatography on silica gel and eluted with ethyl acetate/methanol (20/1) to afford a yellow oil in 61% yield (0.771 g). ^{1}H NMR (600 MHz, $CDCl_3$): δ 7.27 (t, *J* = 7.1 Hz, 2H), 7.19 (d, *J* = 7.6 Hz, 3H), 3.78 (s, 1H), 3.44 (s, 2H), 3.34 (s, 6H), 2.82 (s, 2H), 2.81 (s, 3H). ^{13}C NMR (150 MHz, $CDCl_3$): δ 167.7, 139.4, 128.8, 128.3, 126.0, 53.8, 50.1, 43.1, 34.4. HRMS (ESI-TOF) *m/z* calcd. for $C_{13}H_{19}NNaO_2S^+$ ($[M+Na]^+$) 276.1029. found, 276.1023.

***N*-benzyl-2-(dimethyl(oxo)-λ⁶-sulfaneylidene)acetamide (S40)**

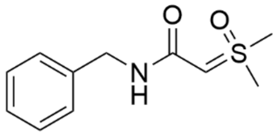

The title compound was prepared according to the general procedure and purified by column chromatography on silica gel and eluted with ethyl acetate/methanol (10/1) to afford a white solid in 40% yield (0.451 g). ^{1}H NMR (600 MHz, DMSO-d_6): δ 7.29 (t, *J* = 7.5 Hz, 2H), 7.23 (d, *J* = 7.1 Hz, 2H), 7.19 (t, *J* = 7.2 Hz, 1H), 6.92 (br s, 1H), 4.19 (d, *J* = 6.1 Hz, 2H), 3.93 (s, 1H), 3.37 (s, 6H). ^{13}C NMR (150 MHz, DMSO-d_6): δ 167.3, 141.4, 128.1, 127.1, 126.3, 56.6, 41.5, 40.0. HRMS (ESI-TOF) *m/z* calcd. for $C_{11}H_{15}NNaO_2S^+$ ($[M+Na]^+$) 248.0716. found, 248.0719.

2-(dimethyl(oxo)-λ^6-sulfaneylidene)-*N*-(thiophen-3-yl)acetamide (S41)

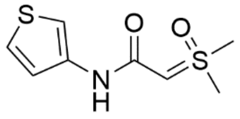

The title compound was prepared according to the general procedure and purified by column chromatography on silica gel and eluted with ethyl acetate/methanol (10/1) to afford a yellow solid in 57% yield (0.618 g). ^{1}H NMR (600 MHz, DMSO-d_6): δ 9.06 (s, 1H), 7.30 (dd, J = 5.1, 3.2 Hz, 1H), 7.22 (dd, J = 3.2, 1.3 Hz, 1H), 6.94 (dd, J = 5.1, 1.3 Hz, 1H), 4.06 (s, 1H), 3.43 (s, 6H). ^{13}C NMR (150 MHz, DMSO-d_6): δ 165.0, 138.8, 123.6, 121.3, 104.0, 58.3, 41.3. HRMS (ESI-TOF) *m/z* calcd. for $C_8H_{11}NNaO_2S_2^+$ ([M+Na]$^+$) 240.0123. found, 240.0129.

***N*-(dibenzo[*b*,*d*]furan-4-yl)-2-(dimethyl(oxo)-λ^6-sulfaneylidene)acetamide (S42)**

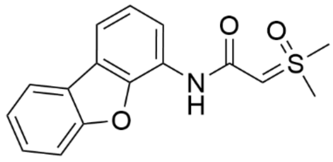

The title compound was prepared according to the general procedure and purified by column chromatography on silica gel and eluted with ethyl acetate/methanol (10/1) to afford a yellow solid in 33% yield (0.497 g). ^{1}H NMR (600 MHz, CDCl$_3$): δ 8.13 (d, J = 7.9 Hz, 1H), 7.92 (d, J = 7.7 Hz, 1H), 7.58–7.53 (m, 2H), 7.43 (t, J = 7.7 Hz, 1H), 7.34 (t, J = 7.4 Hz, 1H), 7.27 (d, J = 8.1 Hz, 1H), 7.15 (s, 1H), 4.31 (s, 1H), 3.49 (s, 6H). ^{13}C NMR (150 MHz, CDCl$_3$): δ 165.6, 155.6, 145.8, 127.0, 125.1, 124.7, 124.2, 123.4, 122.9, 120.8, 118.1, 114.3, 111.6, 56.4, 43.0. HRMS (ESI-TOF) *m/z* calcd. for $C_{16}H_{15}NNaO_3S^+$ ([M+Na]$^+$) 324.0665. found, 324.0669.

2-(dimethyl(oxo)-λ^6-sulfaneylidene)-*N*-(1-methyl-1*H*-indol-5-yl)acetamide (S43)

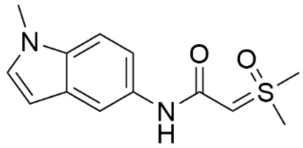

The title compound was prepared according to the general procedure and purified by column chromatography on silica gel and eluted with ethyl acetate/methanol (10/1) to afford a yellow solid in 43% yield (0.567 g). ^{1}H NMR (600 MHz, CDCl$_3$): δ 7.56 (s, 1H), 7.21 (d, J = 8.6 Hz, 1H), 7.13 (d, J = 10.3 Hz, 1H), 7.02 (d, J = 3.0 Hz, 1H), 6.76 (s, 1H), 6.40 (d, J = 2.9 Hz, 1H), 3.75 (s, 3H), 3.47 (s, 1H), 3.40 (s, 6H). ^{13}C NMR (150 MHz, CDCl$_3$): δ 167.5,

150.0, 134.4, 131.0, 129.5, 128.6,118.6, 109.3, 100.9, 55.2, 42.9, 32.9. HRMS (ESI-TOF) *m/z* calcd. for $C_{13}H_{16}N_2NaO_2S^+$ ($[M+Na]^+$) 287.0825. found, 287.0832.

2-(dimethyl(oxo)-λ^6-sulfaneylidene)-*N*-(1-methyl-1*H*-indazol-7-yl)acetamide (S44)

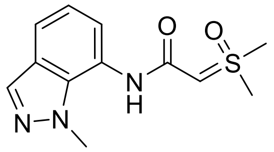

The title compound was prepared according to the general procedure and purified by column chromatography on silica gel and eluted with ethyl acetate/methanol (10/1) to afford a yellow solid in 35% yield (0.463 g). ^{1}H NMR (600 MHz, $CDCl_3$): δ 7.95 (s, 1H), 7.61 (dd, J = 8.1, 0.9 Hz, 1H), 7.18 (dd, J = 7.2, 0.9 Hz, 1H), 7.06 (dd, J = 8.0, 7.3 Hz, 1H), 6.68 (s, 1H), 4.89 (s, 1H), 4.26 (s, 3H), 3.37 (s, 6H). ^{13}C NMR (150 MHz, $CDCl_3$): δ 169.1, 136.7, 132.8, 126.5, 126.4, 121.9, 120.9, 120.2, 55.8, 43.0, 38.1. HRMS (ESI-TOF) *m/z* calcd. for $C_{12}H_{15}N_3NaO_2S^+$ ($[M+Na]^+$) 288.0777. found, 288.0785.

2-(dimethyl(oxo)-λ^6-sulfaneylidene)-*N*-(4-methyl-2-oxo-2*H*-chromen-7-yl)acetamide (S45)

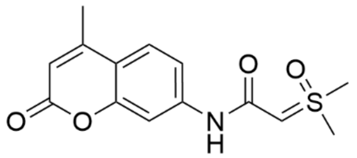

The title compound was prepared according to the general procedure and purified by column chromatography on silica gel and eluted with ethyl acetate/methanol (10/1) to afford a yellow solid in 39% yield (0.572 g). ^{1}H NMR (600 MHz, DMSO-d_6): δ 11.81 (s, 1H), 7.79 (d, J = 8.6 Hz, 1H), 7.76 (d, J = 1.8 Hz, 1H), 7.55 (d, J = 10.4 Hz, 1H), 6.33 (s, 1H), 5.63 (s, 1H), 4.06 (s, 6H), 2.42 (s, 3H). ^{13}C NMR (150 MHz, DMSO-d_6): δ 159.8, 158.8, 153.5, 153.0, 140.8, 126.3, 116.2, 115.6, 113.1, 106.5, 57.1, 38.7, 18.0. HRMS (ESI-TOF) *m/z* calcd. for $C_{14}H_{15}NNaO_4S^+$ ($[M+Na]^+$) 316.0614. found, 316.0618.

N-(2-(1H-indol-3-yl)ethyl)-2-(dimethyl(oxo)-λ^6-sulfaneylidene)acetamide (S46)

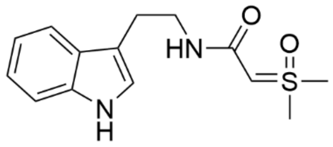

The title compound was prepared according to the general procedure and purified by column chromatography on silica gel and eluted with ethyl acetate/methanol (10/1) to afford a yellow solid in 41% yield (0.571 g). ^{1}H NMR (600 MHz, DMSO-d_6): δ 10.78 (br s, 1H), 7.53 (d, J = 7.8 Hz, 1H), 7.32 (d, J = 8.0 Hz, 1H), 7.12 (s, 1H), 7.05 (t, J = 7.4 Hz, 1H), 6.97 (t, J = 7.3 Hz, 1H), 6.51 (s, 1H), 4.03 (s, 1H), 3.37 (s, 6H), 3.28–3.23 (m, 2H), 2.76 (t, J = 7.3 Hz, 2H). ^{13}C NMR (150 MHz, DMSO-d_6): δ 167.4, 136.2, 127.3, 122.5, 120.8, 118.3, 118.1, 112.3, 111.3, 56.5, 41.5, 40.0, 26.3. HRMS (ESI-TOF) *m/z* calcd. for $C_{14}H_{18}N_2NaO_2S^+$ ([M+Na]$^+$) 301.0981. found, 301.0983.

2-(dimethyl(oxo)-λ^6-sulfaneylidene)-N-methoxy-N-methylacetamide (S47)

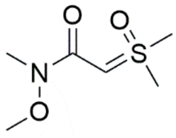

The title compound was prepared according to the general procedure and purified by column chromatography on silica gel and eluted with ethyl acetate/methanol (10/1) to afford a yellow solid in 32% yield (0.286 g). ^{1}H NMR (600 MHz, DMSO-d_6): δ 4.55 (s, 1H), 3.54 (s, 3H), 3.42 (s, 6H), 2.88 (s, 3H). ^{13}C NMR (151 MHz, DMSO-d_6): δ 171.0, 60.6, 58.8, 42.1, 41.1. HRMS (ESI-TOF) *m/z* calcd. for $C_6H_{13}NNaO_3S^+$ ([M+Na]$^+$) 202.0508. found, 202.0515.

2-(dimethyl(oxo)-λ^6-sulfaneylidene)-N-methyl-N-(naphthalen-1-ylmethyl)acetamide (S48)

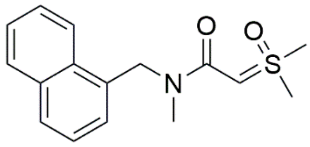

The title compound was prepared according to the general procedure and purified by column chromatography on silica gel and eluted with ethyl acetate/methanol (10/1) to afford a yellow solid in 78% yield (1.127 g). ^{1}H NMR (600 MHz, $CDCl_3$): δ 8.07 (s, 1H), 7.86 (d, J = 7.9 Hz, 1H), 7.77 (d, J = 8.2 Hz, 1H), 7.50 (p, J = 6.7 Hz, 2H), 7.43 (t, J = 7.6 Hz, 1H), 7.32 (d, J = 7.0 Hz, 1H), 4.96 (s, 2H), 3.83 (s, 1H), 3.43 (s, 6H), 2.83 (s, 3H). ^{13}C NMR (150

MHz, $CDCl_3$): δ 168.3, 133.7, 133.4, 131.4, 128.6, 127.7, 126.1, 125.7, 125.3, 123.3, 54.4, 48.7, 43.1, 33.8. HRMS (ESI-TOF) *m/z* calcd. for $C_{16}H_{19}NNaO_2S^+$ ($[M+Na]^+$) 312.1029. found, 312.1038.

2-(dimethyl(oxo)-λ^6-sulfaneylidene)-1-(4-(2-((2,4-dimethylphenyl)thio)phenyl)piperazin-1-yl)ethan-1-one (S49)

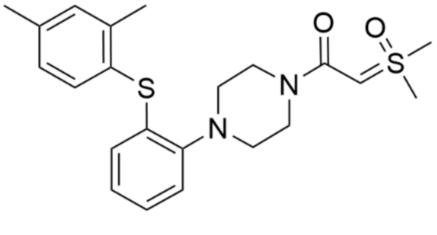

The title compound was prepared according to the general procedure and purified by column chromatography on silica gel and eluted with ethyl acetate/methanol (10/1) to afford a yellow solid in 68% yield (1.414 g). ^{1}H NMR (600 MHz, $CDCl_3$): δ 7.36 (d, J = 7.7 Hz, 1H), 7.14 (s, 1H), 7.06 (t, J = 7.4 Hz, 1H), 7.02 (d, J = 7.4 Hz, 2H), 6.86 (t, J = 7.4 Hz, 1H), 6.52 (d, J = 7.8 Hz, 1H), 3.89 (s, 1H), 3.56 (s, 4H), 3.42 (s, 6H), 3.02 (s, 4H), 2.35 (s, 3H), 2.31 (s, 3H). ^{13}C NMR (150 MHz, $CDCl_3$): δ 167.5, 149.0, 142.3, 139.2, 136.1, 134.6, 131.7, 127.8 127.7, 126.3, 125.5, 124.5, 119.9, 54.1, 51.6, 43.4, 21.2, 20.6. HRMS (ESI-TOF) *m/z* calcd. for $C_{22}H_{28}N_2NaO_2S_2^+$ ($[M+Na]^+$) 439.1484. found, 439.1475.

2-(dimethyl(oxo)-λ^6-sulfaneylidene)-1-(4-(pyrimidin-2-yl)piperazin-1-yl)ethan-1-one (S50)

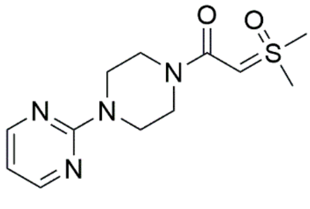

The title compound was prepared according to the general procedure and purified by column chromatography on silica gel and eluted with ethyl acetate/methanol (5/1). ^{1}H NMR (600 MHz, $CDCl_3$): δ 8.26 (d, J = 4.7 Hz, 2H), 6.46 (t, J = 4.7 Hz, 1H), 3.87 (s, 1H), 3.80–3.73 (m, 4H), 3.42 (s, 4H), 3.38 (s, 6H).^{13}C NMR (150 MHz, $CDCl_3$): δ 167.4, 161.5, 157.6, 110.0, 54.5, 43.4, 43.2, 42.7. HRMS (ESI-TOF) *m/z* calcd. for $C_{12}H_{18}N_4NaO_2S^+$ ($[M+Na]^+$) 305.1043. found, 305.1048.

1-(4-(2-chlorodibenzo[*b,f*][1,4]oxazepin-11-yl)piperazin-1-yl)-2-(dimethyl(oxo)-λ^6-sulfaneylidene)ethan-1-one (S51)

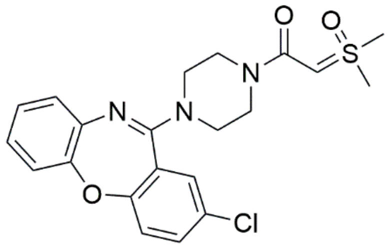

The title compound was prepared according to the general procedure and purified by column chromatography on silica gel and eluted with ethyl acetate/methanol (10/1). ^{1}H NMR (600 MHz, $CDCl_3$): δ 7.39 (dd, *J* = 8.7, 2.6 Hz, 1H), 7.31 (d, *J* = 2.6 Hz, 1H), 7.18 (d, *J* = 8.7 Hz, 1H), 7.14 (dd, *J* = 7.8, 1.5 Hz, 1H), 7.11–7.05 (m, 2H), 6.99 (td, *J* = 7.7, 1.7 Hz, 1H), 3.86 (s, 1H), 3.51 (s, 8H), 3.40 (s, 6H).^{13}C NMR (150 MHz, $CDCl_3$): δ 167.6, 159.3, 158.8, 151.8, 140.0, 132.6, 130.3, 129.0, 127.0, 125.8, 124.9, 124.7, 122.7, 120.1, 54.3, 47.3, 43.4, 42.9. HRMS (ESI-TOF) *m/z* calcd. for $C_{21}H_{22}ClN_3NaO_3S^+$ ([M+Na]$^+$) 454.0963. found, 454.0968.

2-(dimethyl(oxo)-λ^6-sulfaneylidene)-1-(1*H*-pyrrol-1-yl)ethan-1-one (S56)[4]

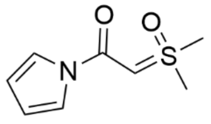

The title compound was prepared according to the general procedure and purified by column chromatography on silica gel and eluted with ethyl acetate. ^{1}H NMR (600 MHz, DMSO-d_6): δ 7.26 (t, *J* = 2.4 Hz, 2H), 6.13 (t, *J* = 2.4 Hz, 2H), 5.31 (s, 1H), 3.55 (s, 6H). ^{13}C NMR (150 MHz, DMSO-d_6): δ 161.0, 117.8, 110.2, 62.8, 40.7.

2-(dimethyl(oxo)-λ^6-sulfaneylidene)-1-(3,5-dimethyl-1*H*-pyrazol-1-yl)ethan-1-one (S57)[4]

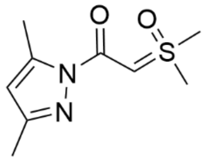

The title compound was prepared according to the general procedure and purified by column chromatography on silica gel and eluted with ethyl acetate. ^{1}H NMR (600 MHz, $CDCl_3$): δ 5.83 (s, 1H), 5.37 (s, 1H), 3.44 (s, 6H), 2.51 (s, 3H), 2.17 (s, 3H). ^{13}C NMR (150 MHz, $CDCl_3$): δ 164.5, 149.8, 142.7, 109.3, 61.7, 41.9, 14.2, 13.6.

2-(dimethyl(oxo)-λ^6-sulfaneylidene)-1-(1-methyl-1*H*-indol-3-yl)ethan-1-one (S59)[4]

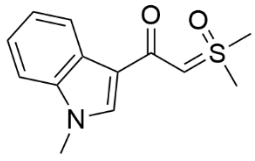

The title compound was prepared according to the general procedure and purified by column chromatography on silica gel and eluted with ethyl acetate/methanol (5/1). ^{1}H NMR (600 MHz, DMSO-d_6): δ 8.16 (d, J = 7.9 Hz, 1H), 7.79 (s, 1H), 7.43 (d, J = 8.2 Hz, 1H), 7.17 (t, J = 8.0 Hz, 1H), 7.11 (t, J = 7.4 Hz, 1H), 5.27 (s, 1H), 3.79 (s, 3H), 3.54 (s, 6H). ^{13}C NMR (150 MHz, DMSO-d_6): δ 179.1, 136.8, 132.0, 125.9, 121.5, 121.4, 120.3, 115.9, 110.0, 70.2, 41.1, 32.7.

2-(bis(methyl-d$_3$)(oxo)-λ^6-sulfaneylidene)-*N*,*N*-diphenylacetamide-*d* (1a-*d*$_7$)

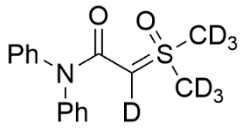

The title compound was prepared according to the general procedure and purified by column chromatography on silica gel and eluted with ethyl acetate/methanol (5/1). ^{1}H NMR (600 MHz, $CDCl_3$): δ 7.30 (t, J = 7.7 Hz, 4H), 7.26 (d, J = 7.7 Hz, 4H), 7.16 (t, J = 7.1 Hz, 2H). ^{13}C NMR (150 MHz, $CDCl_3$: δ 167.2, 143.6, 128.9, 127.8, 125.7, 56.9, 43.0.

10. References

1. Iwai, T.; Fujihara, T.; Terao, J.; Tsuji, Y. Iridium-Catalyzed Annulation of *N*-Arylcarbamoyl Chlorides with Internal Alkynes. *J. Am. Chem. Soc.* **2010**, *132*, 9602–9603.
2. Zhu, S.; Shi, K.; Zhu, H.; Jia, Z.-K.; Xia, X.-F.; Wang, D.; Zou, L.-H. Copper-Catalyzed Annulation or Homocoupling of Sulfoxonium Ylides: Synthesis of 2,3-Diaroylquinolines or α,α,β-Tricarbonyl Sulfoxonium Ylides. *Org. Lett.* **2020**, *22*, 1504–1509.
3. Caiuby, C. A.; Vidal, L.; Burtoloso, A. C.; Aïssa, C. Cyclic Sulfoxonium Ylides: Synthesis and Chemospecific Reactivity in the Catalytic Alkylation of Indoles. *ChemCatChem*. **2023**, *15*, e202201643.
4. Xu, Y.; Gao, Y.; Su, L.; Wu, H.; Tian, H.; Zeng, M.; Xu, C.; Zhu, X.; Liao, K. High-Throughput Experimentation and Machine Learning-Assisted Optimization of Iridium-Catalyzed Cross-Dimerization of Sulfoxonium Ylides. *Angew. Chem. Int. Ed.* **2023**, *62*, e202313638.
5. Xu, Y.; Ren, F.; Su, L.; Xiong, Z.; Zhu, X.; Lin, X.; Qiao, N.; Tian, H. Tian, C.; Liao, K. HTE and machine learning-assisted development of iridium (I)-catalyzed selective O–H bond insertion reactions toward carboxymethyl ketones. *Org. Chem. Front.* **2023**, *10*, 1153–1159.
6. Liu, L.; Sun, K.; Su, L.; Dong, J.; Cheng, L.; Zhu, X.; Au, C.-T.; Zhou, Y.; Yin, S.-F. Palladium-Catalyzed Regio-and Stereoselective Coupling–Addition of Propiolates with Arylsulfonyl Hydrazides: A Pattern for Difunctionalization of Alkynes. *Org. Lett.* **2018**, *20*, 4023–4027.
7. Mori, A.; Miyakawa, Y.; Ohashi, E.; Haga, T.; Maegawa, T.; Sajiki, H. Pd/C-catalyzed chemoselective hydrogenation in the presence of diphenylsulfide. *Org. Lett.* **2006**, *8*, 3279–3281.
8. Vishwakarma, R. K.; Sen, R.; Deshwal, S.; Vaitla, J. Regioselective Synthesis of N-Aryl Pyrazoles from Alkenyl Sulfoxonium Ylides and Aryl Diazonium Salts. *J. Org. Chem.* **2024**, *89*, 18535–18549.
9. Wang H.; Chen L.; Chen Y.; Zhang Y. Highly efficient iron-catalyzed conjugate reduction of α,β-unsaturated ketones with polymethylhydrosiloxane. *Org. Biomol. Chem*. **2024**, *22*, 5097–5100.
10. Gong, Y.; Xu, X.-C.; Meng, Y. X.; Zhao, Y.-L. Silver-Catalyzed Tandem Cyclization of Sulfur Ylides with Terminal Alkynes: Direct Synthesis of Vinylcyclopropanes. *Org. Chem. Front.* **2025**. DOI: 10.1039/d5QO00012b.
11. Dong, Y.; Tian, Y.; Zhang, Z.; Wang, T. Blue Light-Promoted Reaction of α-Diazoketones and Sulfoxonium Ylides: Synthesis of 1, 3-Dicarbonyl Sulfoxonium Ylides. *Adv. Synth. Catal.* **2022**, *364*, 4026–4030.
12. Liao, H.; Dong, J.; Zhou, X.; Jiang, Q.; Lv, Z.; Lei, F.; Xue, D. Silver-mediated formal [4π+ 2 σ] cycloaddition reactions of bicyclobutanes with nitrile imines: access to 2, 3-diazobicyclo [3.1. 1] heptenes. *Chem. Sci*. **2025**,

16, 4654–4660.

13. Watanabe, M.; Kinoshita, T.; Furukawa, S. Stable Sulfur Ylides. III. Reaction of Stable Sulfonium Allylides and Oxosulfonium Allylides with Alkoxides. *Chem. Pharm. Bull.* **1975**, *23*, 258–263.
14. Gaussian 16, Revision C.01, Frisch, M. J.; Trucks, G. W.; Schlegel, H. B.; Scuseria, G. E.; Robb, M. A.; Cheeseman, J. R.; Scalmani, G.; Barone, V.; Petersson, G. A.; Nakatsuji, H.; Li, X.; Caricato, M.; Marenich, A. V.; Bloino, J.; Janesko, B. G.; Gomperts, R.; Mennucci, B.; Hratchian, H. P.; Ortiz, J. V.; Izmaylov, A. F.; Sonnenberg, J. L.; Williams-Young, D.; Ding, F.; Lipparini, F.; Egidi, F.; Goings, J.; Peng, B.; Petrone, A.; Henderson, T.; Ranasinghe, D.; Zakrzewski, V. G.; Gao, J.; Rega, N.; Zheng, G.; Liang, W.; Hada, M.; Ehara, M.; Toyota, K.; Fukuda, R.; Hasegawa, J.; Ishida, M.; Nakajima, T.; Honda, Y.; Kitao, O.; Nakai, H.; Vreven, T.; Throssell, K.; Montgomery, J. A., Jr.; Peralta, J. E.; Ogliaro, F.; Bearpark, M. J.; Heyd, J. J.; Brothers, E. N.; Kudin, K. N.; Staroverov, V. N.; Keith, T. A.; Kobayashi, R.; Normand, J.; Raghavachari, K.; Rendell, A. P.; Burant, J. C.; Iyengar, S. S.; Tomasi, J.; Cossi, M.; Millam, J. M.; Klene, M.; Adamo, C.; Cammi, R.; Ochterski, J. W.; Martin, R. L.; Morokuma, K.; Farkas, O.; Foresman, J. B.; Fox, D. J. Gaussian, Inc., Wallingford CT, 2019.
15. Chai, J.-D.; Head-Gordon, M. Long-range corrected hybrid density functionals with damped atom-atom dispersion corrections. *Phys. Chem. Chem. Phys.* **2008**, *10*, 6615–6620.
16. Pople, J. A.; Krishnan, R.; Schlegel, H. B.; Binkley, J. S. Derivative studies in Hartree-Fock and Møller-Plesset theories. *Int. J. Quantum Chem*. **1979**, *16*, 225–241.
17. Hehre, W. J.; Ditchfield, R.; Pople, J. A. Self—Consistent Molecular Orbital Methods. XII. Further Extensions of Gaussian—Type Basis Sets for Use in Molecular Orbital Studies of Organic Molecules. *J. Chem. Phys.* **1972**, *56*, 2257–2261.
18. Hariharan, P. C.; Pople, J. A. The influence of polarization functions on molecular orbital hydrogenation energies. *Theor. Chim. Acta* **1973**, *28*, 213–222.
19. Clark, T.; Chandrasekhar, J.; Spitznagel, G. W. Efficient diffuse function-augmented basis sets for anion calculations. III. The 3-21+G basis set for first-row elements, Li–F. *J. Comput. Chem.* **1983**, *4*, 294–301.
20. Frisch, M. J.; Pople, J. A.; Binkley, J. S. Self-consistent molecular orbital methods 25. Supplementary functions for Gaussian basis sets. *J. Chem. Phys.* **1984**, *80*, 3265–3269.
21. Goerigk, L.; Grimme, S. Efficient and Accurate Double-Hybrid-Meta-GGA Density Functionals—Evaluation with the Extended GMTKN30 Database for General Main Group Thermochemistry, Kinetics, and Noncovalent Interactions. *J. Chem. Theory Comput*. **2011**, *7*, 291–309.

22. Grimme, S.; Ehrlich, S.; Goerigk, L. Effect of the Damping Function in Dispersion Corrected Density Functional Theory. *J. Comput. Chem.*, **2011**, *32*, 1456–1465.

23. Weigend, F.; Ahlrichs, R. Balanced basis sets of split valence, triple zeta valence and quadruple zeta valence quality for H to Rn: Design and assessment of accuracy. *Phys. Chem. Chem. Phys.* **2005**, *7*, 3297–3305.

24. Weigend, F. Accurate Coulomb-fitting basis sets for H to Rn. *Phys. Chem. Chem. Phys.* **2006**, *8*, 1057-1065.

25. Neese, F. The ORCA program system. *WIREs Comput. Mol. Sci*. **2012**, *2*, 73–78.

26. Neese, F. Software update: the ORCA program system, version 4.0. *WIREs Comput. Mol. Sci*. **2017**, *8*, e1327.

27. Neese, F.; Wennmohs, F.; Becker, U.; Riplinger, C. The ORCA quantum chemistry program package. *J. Chem. Phys*. **2020**, *152*, 224108.

28. Neese, F. Software update: The ORCA program system—Version 5.0. *WIREs Comput. Mol. Sci*. **2022**, *12*, e1606.

29. Marenich, A. V.; Cramer, C. J.; Truhlar, D. G. Universal solvation model based on solute electron density and on a continuum model of the solvent defined by the bulk dielectric constant and atomic surface tensions. *J. Phys. Chem. B* **2009**, *113*, 6378–6396.

30. Lu, T. Molclus program, Version 1.10, http://www.keinsci.com/research/molclus.html (accessed July 10, 2023)

31. Pracht, P.; Bohle, F.; Grimme, S. Automated exploration of the low-energy chemical space with fast quantum chemical methods. *Phys. Chem. Chem. Phys*. **2020**, *22*, 7169–7192.

32. Legault, C. Y. CYLView, 1.0b; Universitéde Sherbrooke: Québec, Montreal, Canada, **2009**; (http://www.cylview.org).

11. Copies of ^{1}H, ^{13}C and ^{19}F NMR Spectra of the Products

^{1}H NMR (600 MHz, $CDCl_3$) Spectrum of **3**

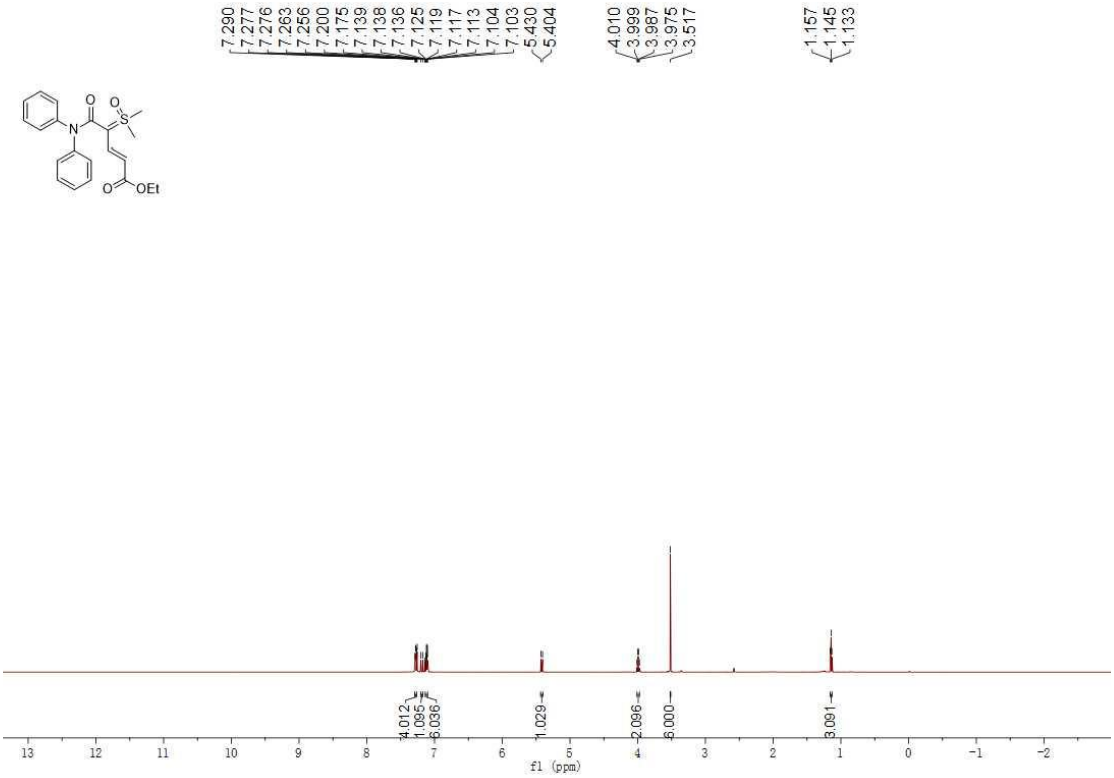

^{13}C NMR (150 MHz, $CDCl_3$) Spectrum of **3**

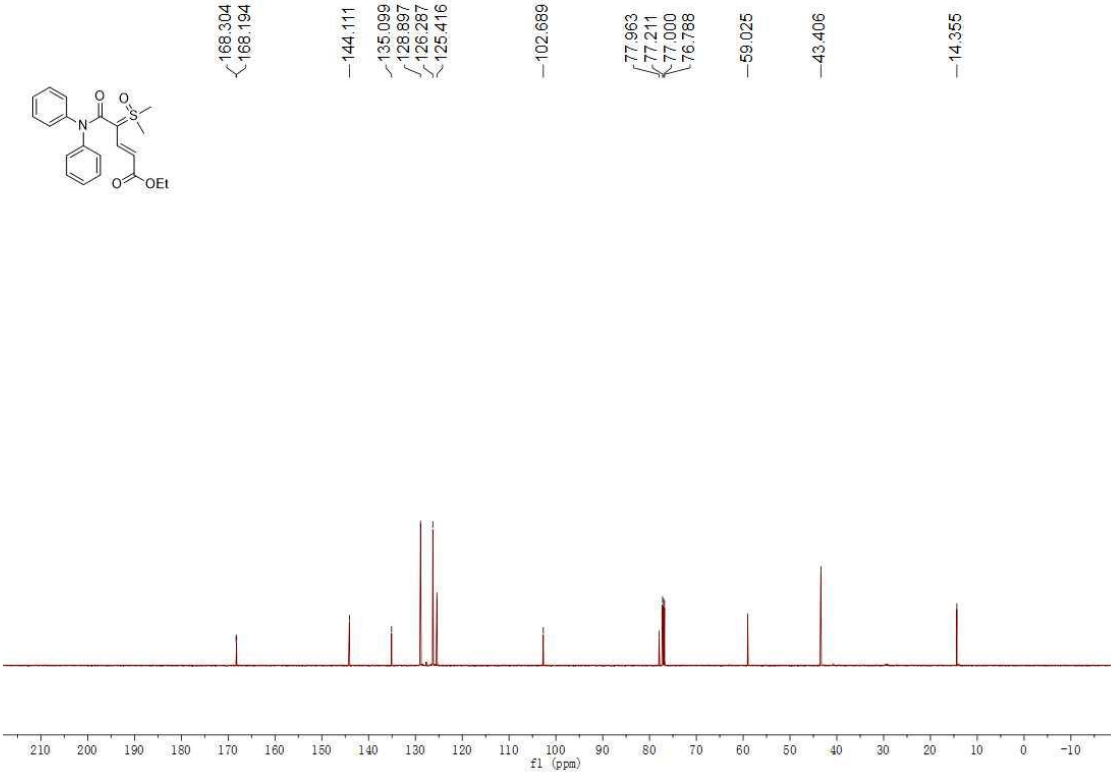

^{1}H NMR (600 MHz, $CDCl_3$) Spectrum of **3'**

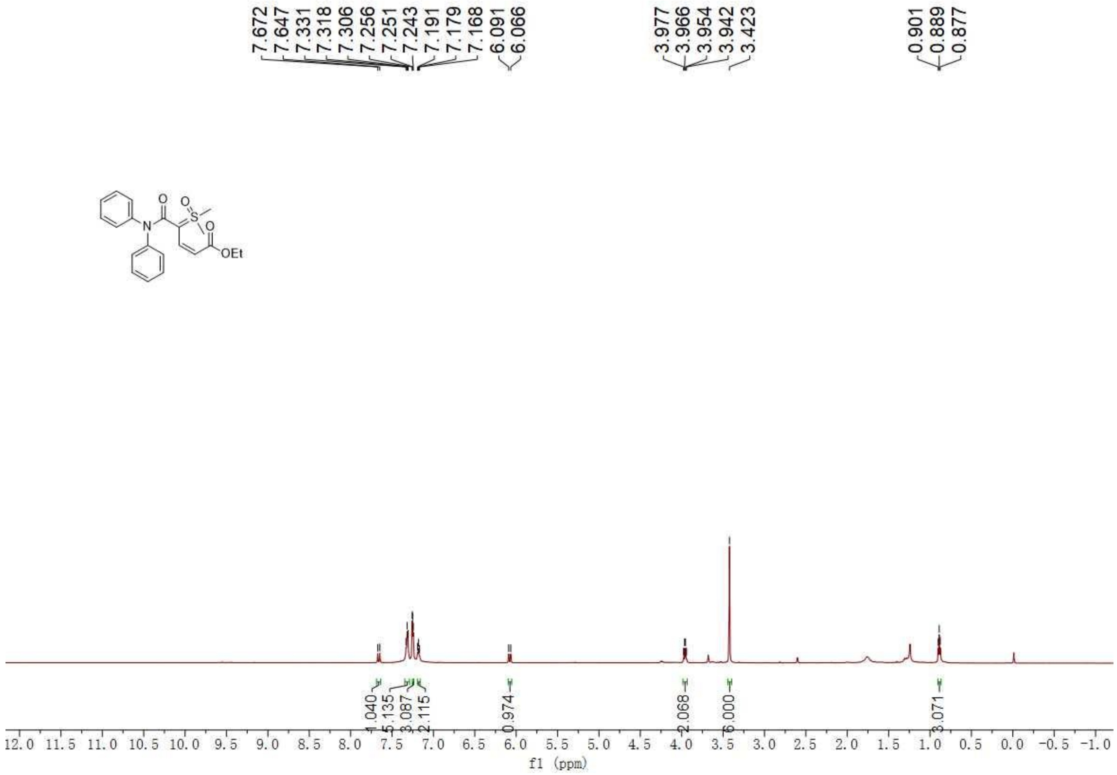

^{13}C NMR (150 MHz, $CDCl_3$) Spectrum of **3'**

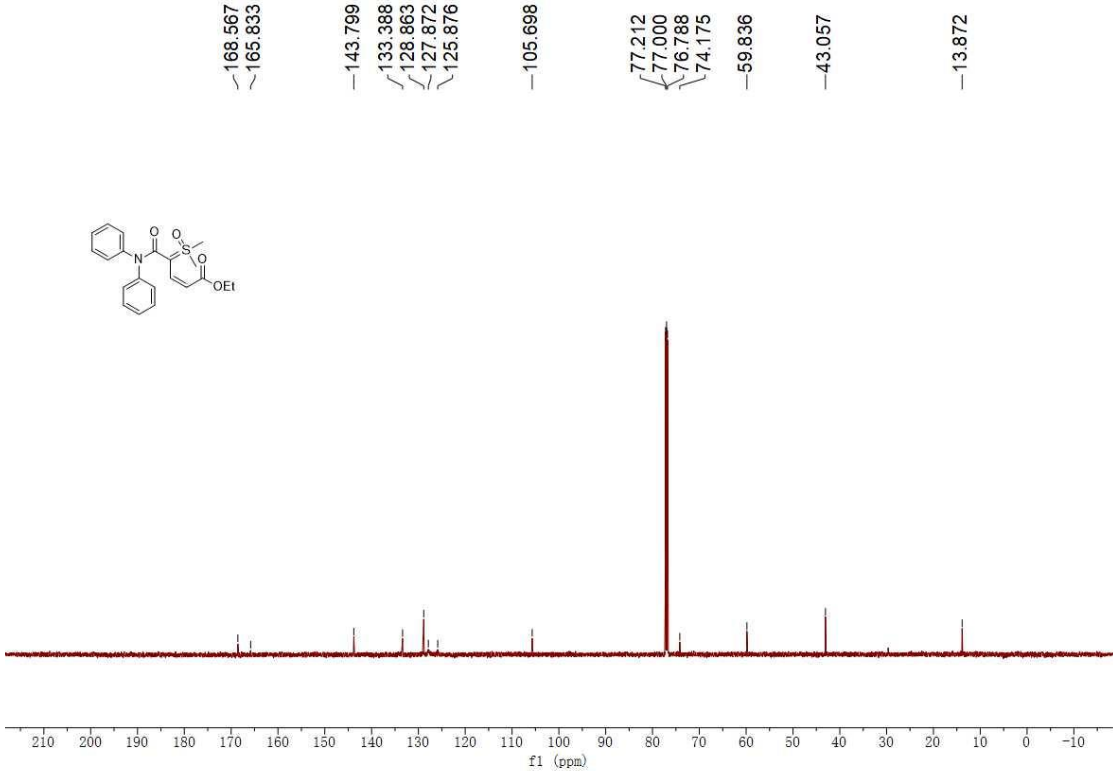

^{1}H NMR (600 MHz, $CDCl_3$) Spectrum of **4**

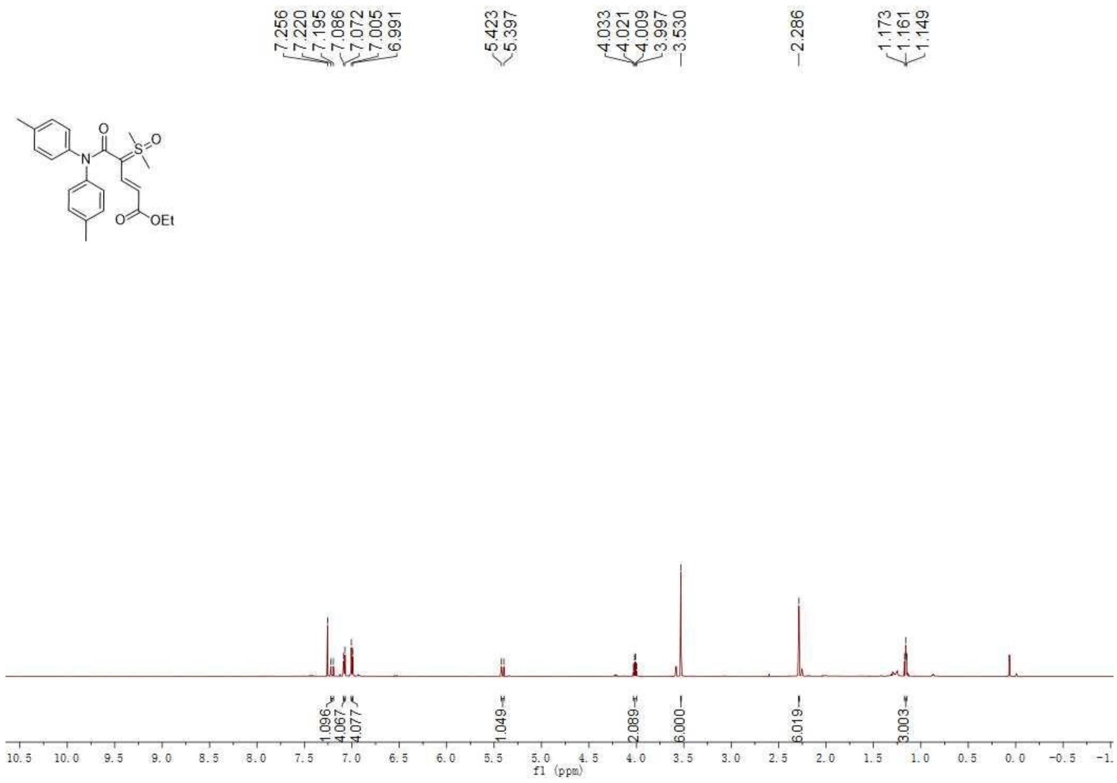

^{13}C NMR (150 MHz, $CDCl_3$) Spectrum of **4**

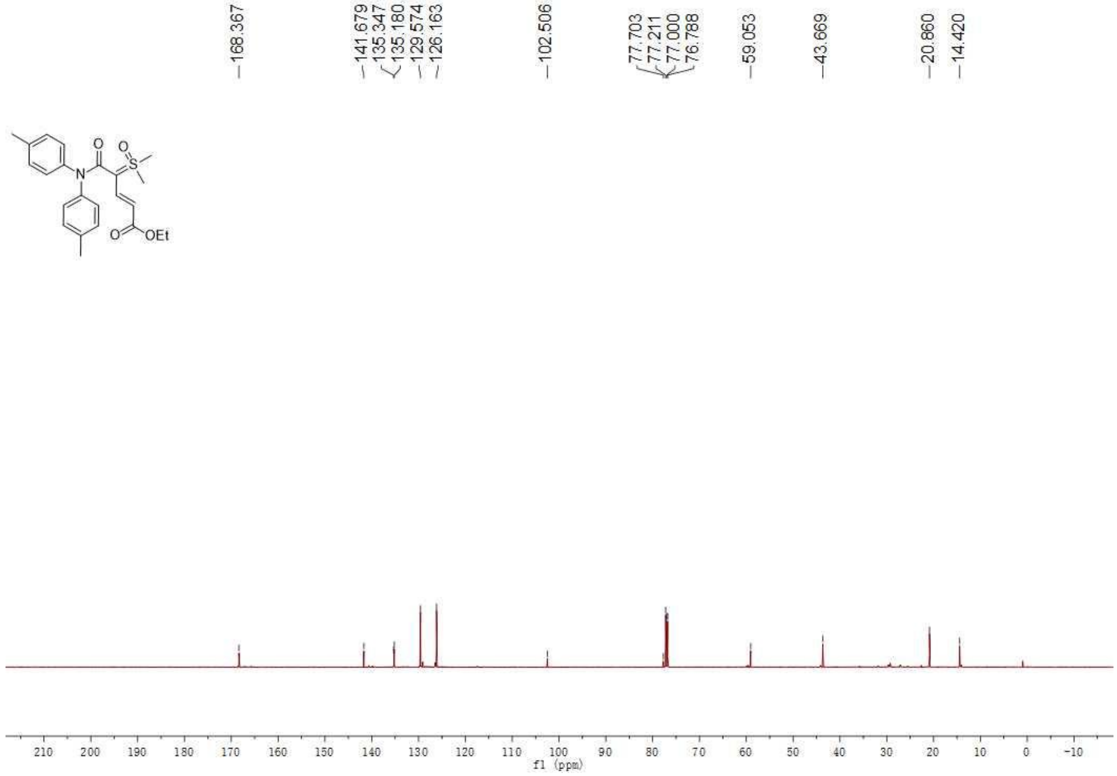

^{1}H NMR (600 MHz, $CDCl_3$) Spectrum of **5**

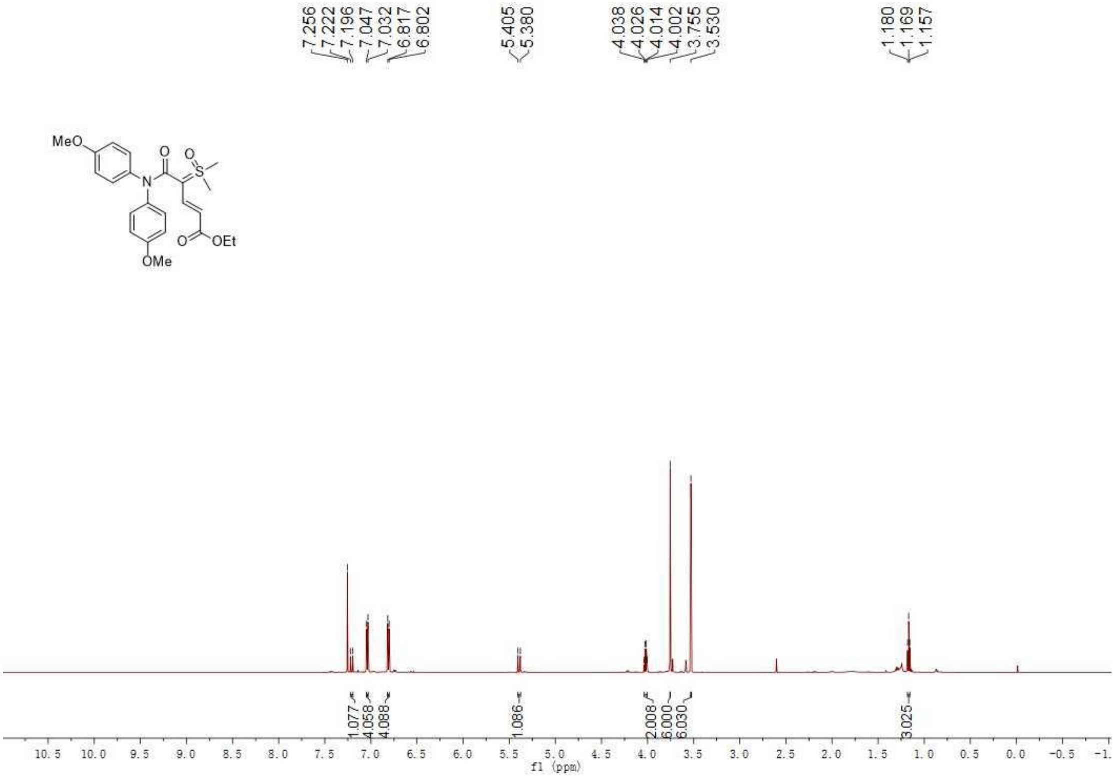

^{13}C NMR (150 MHz, $CDCl_3$) Spectrum of **5**

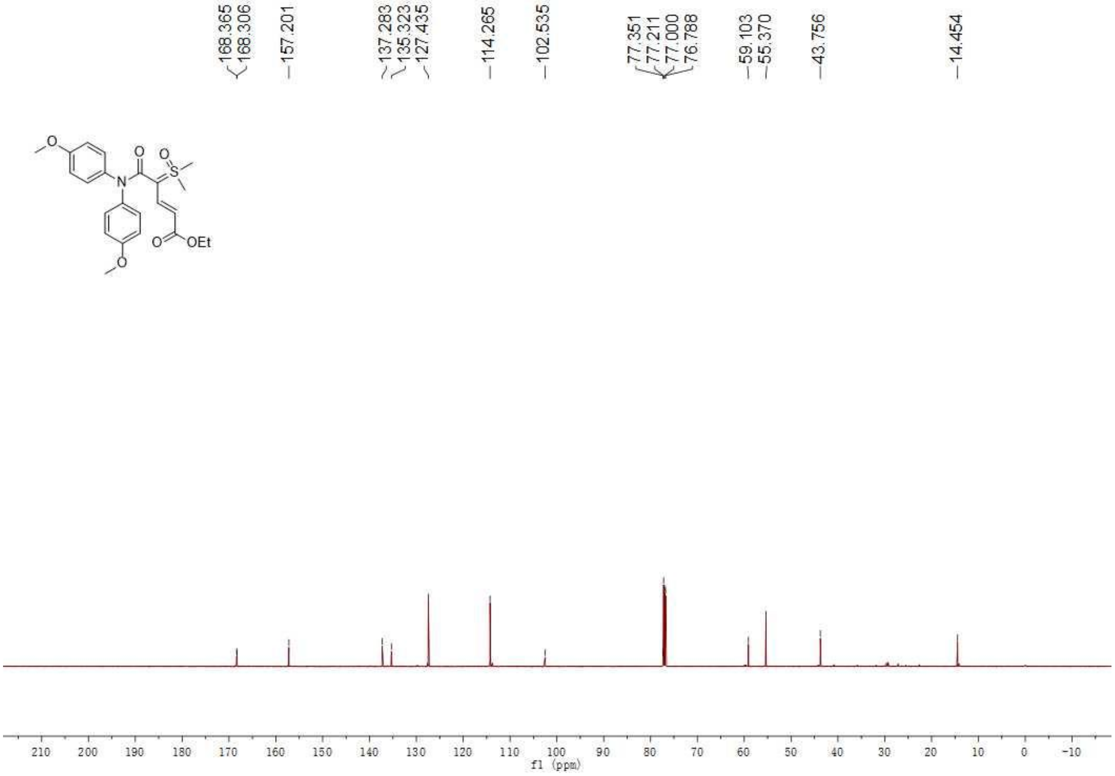

^{1}H NMR (600 MHz, $CDCl_3$) Spectrum of **6**

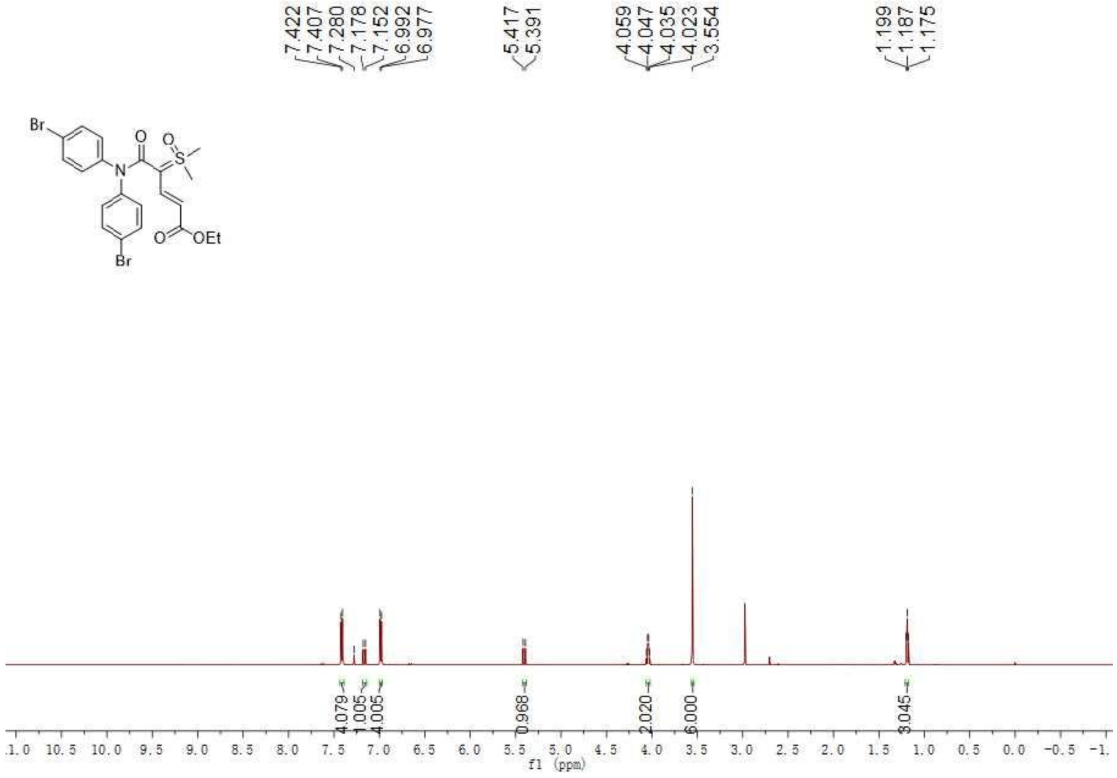

^{13}C NMR (150 MHz, $CDCl_3$) Spectrum of **6**

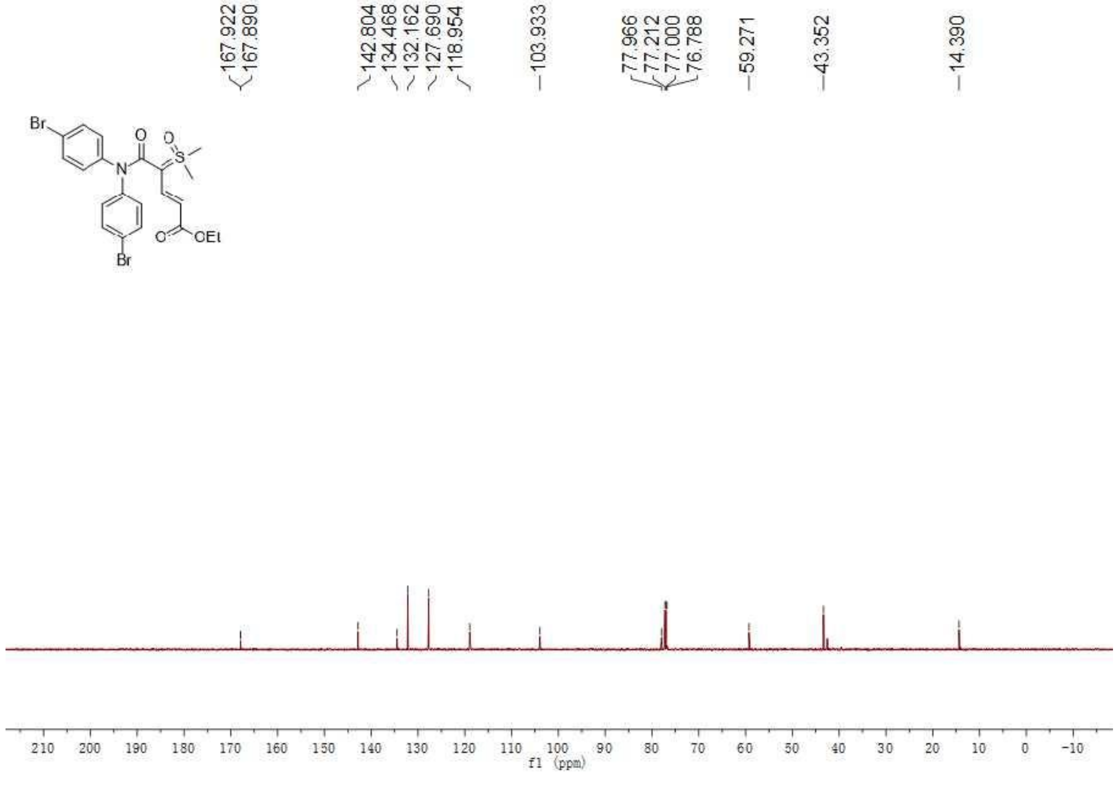

^{1}H NMR (600 MHz, $CDCl_3$) Spectrum of **7**

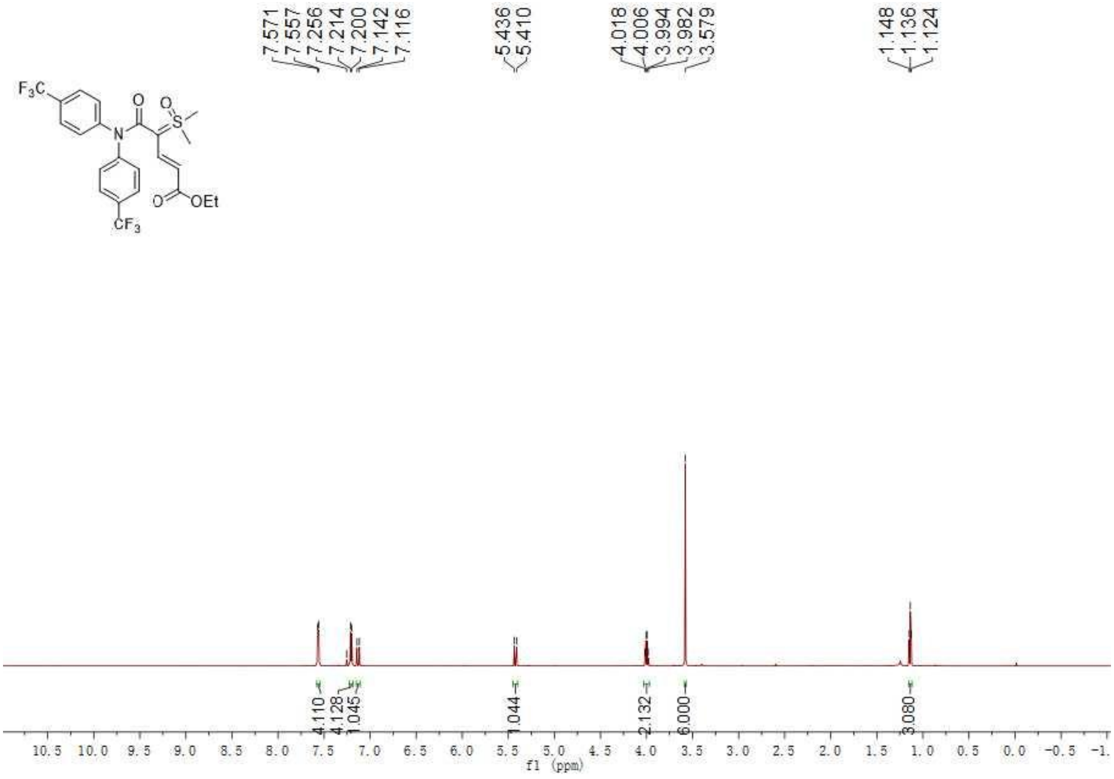

^{13}C NMR (150 MHz, $CDCl_3$) Spectrum of **7**

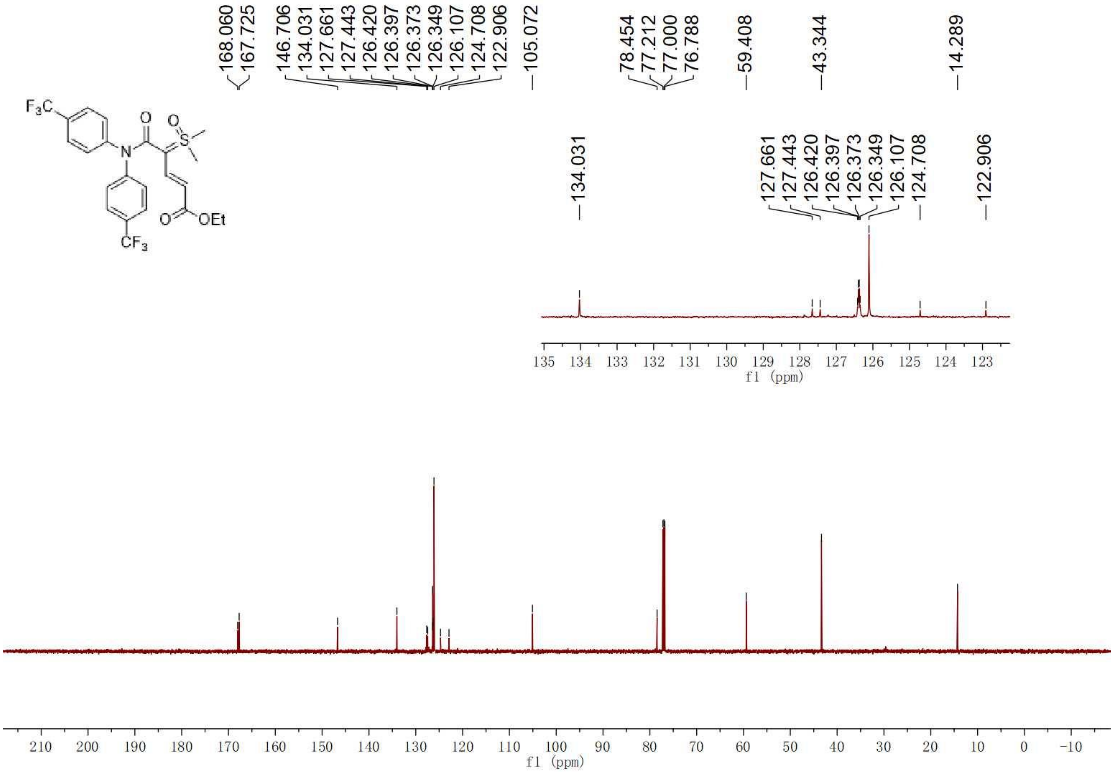

^{19}F NMR (564 MHz, $CDCl_3$) Spectrum of **7**

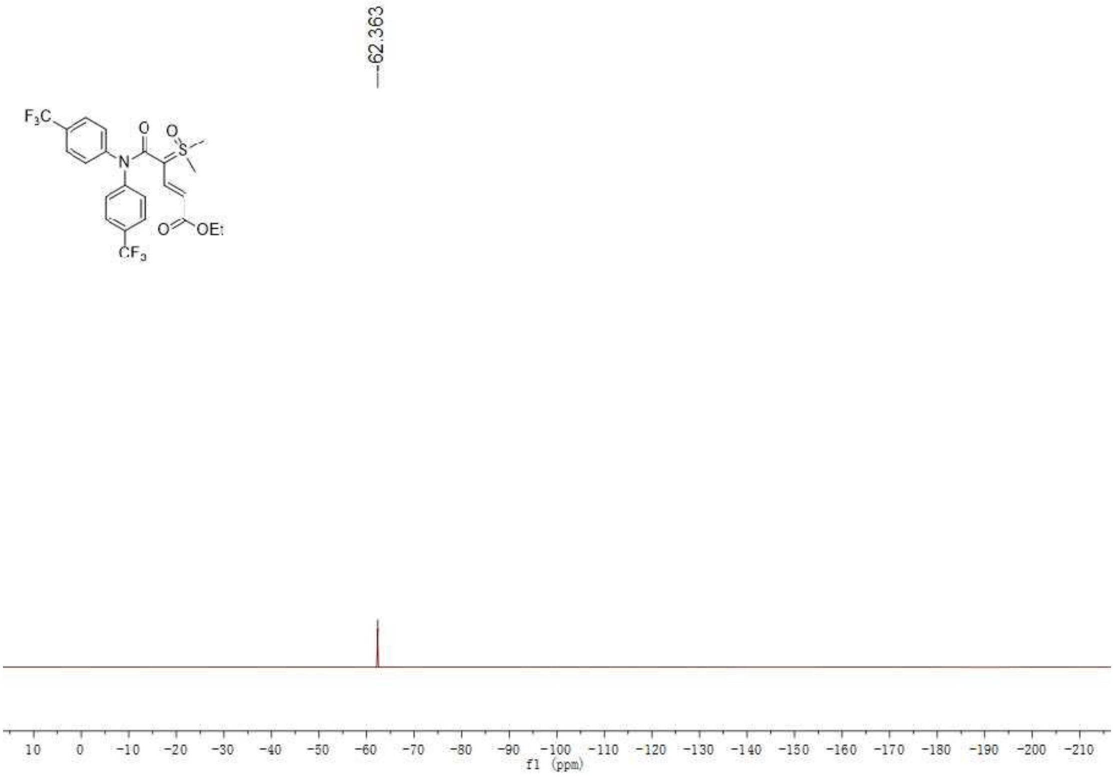

^{1}H NMR (600 MHz, $CDCl_3$) Spectrum of **8**

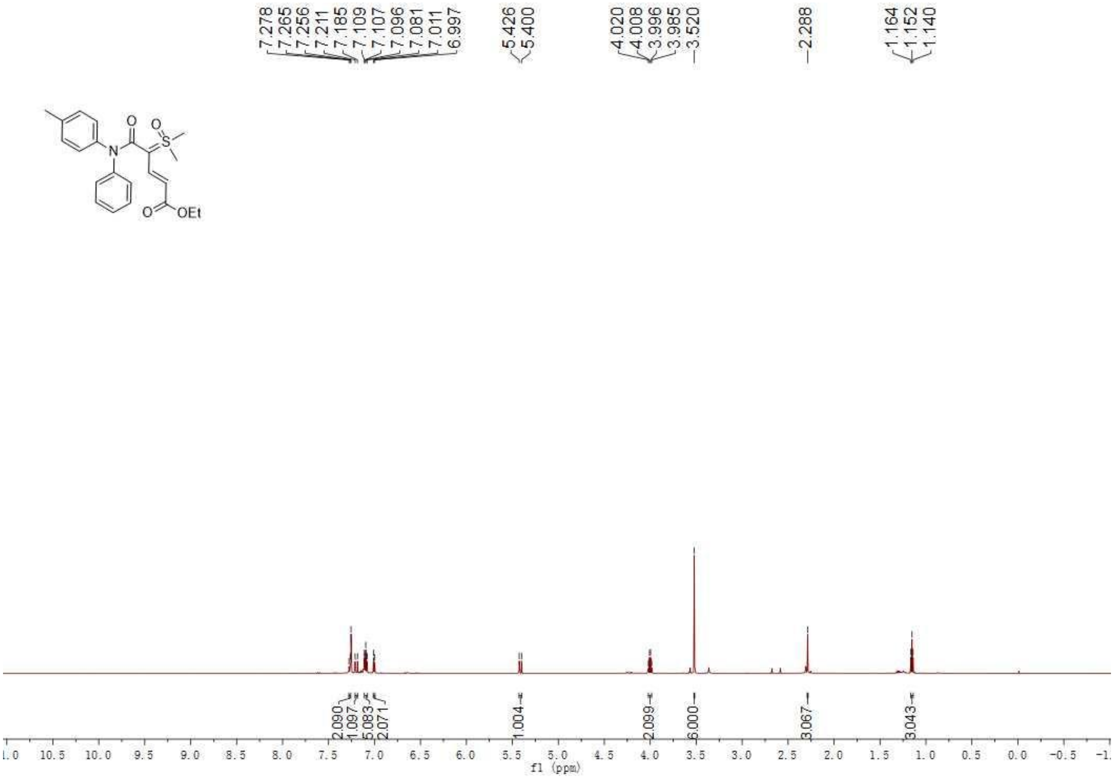

^{13}C NMR (150 MHz, $CDCl_3$) Spectrum of **8**

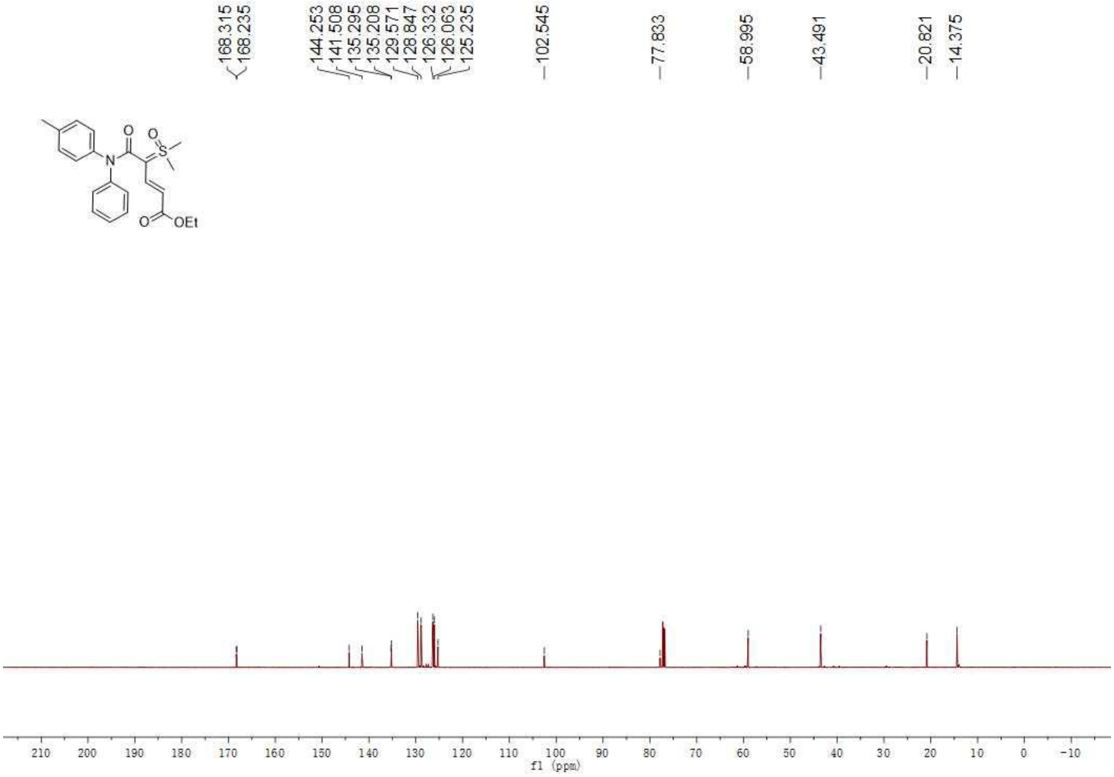

^{1}H NMR (600 MHz, $CDCl_3$) Spectrum of **9**

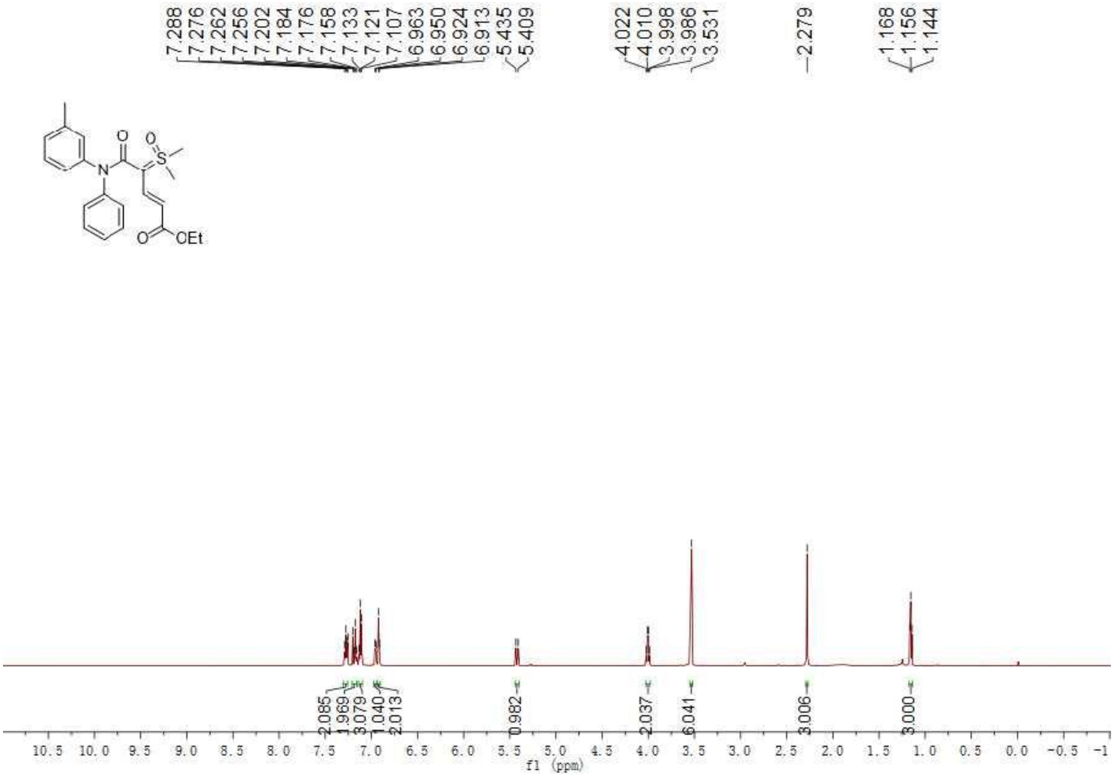

^{13}C NMR (150 MHz, $CDCl_3$) Spectrum of **9**

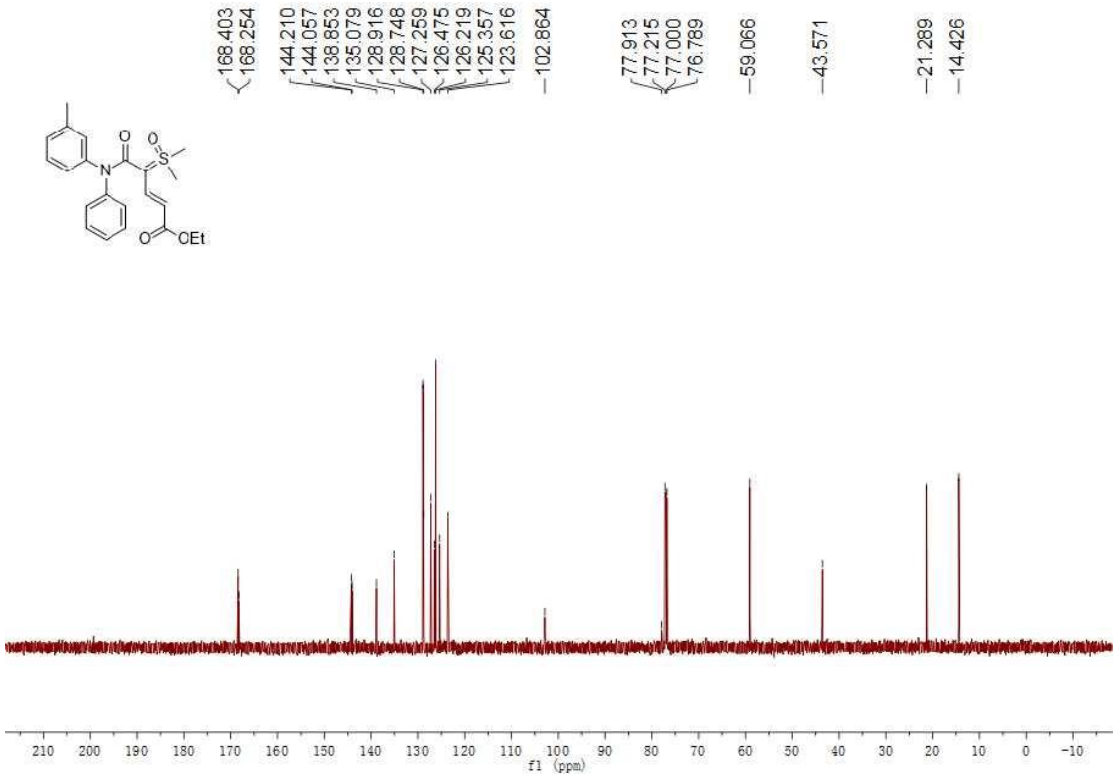

^{1}H NMR (600 MHz, $CDCl_3$) Spectrum of **10**

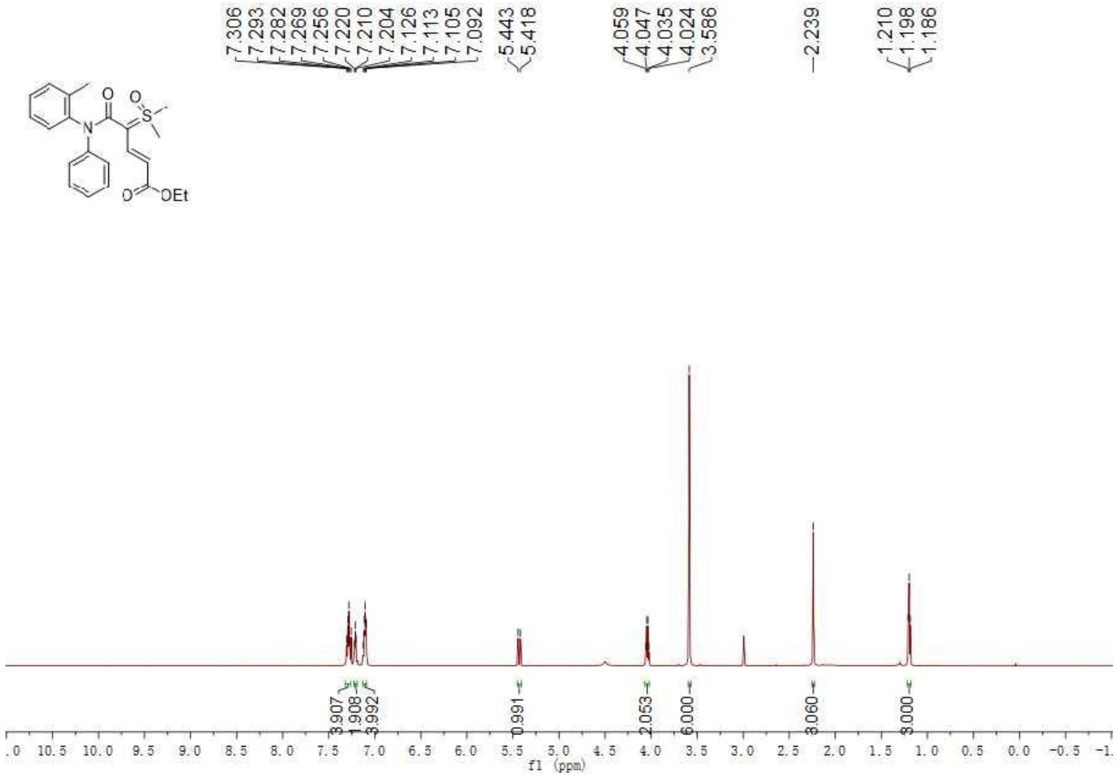

^{13}C NMR (150 MHz, $CDCl_3$) Spectrum of **10**

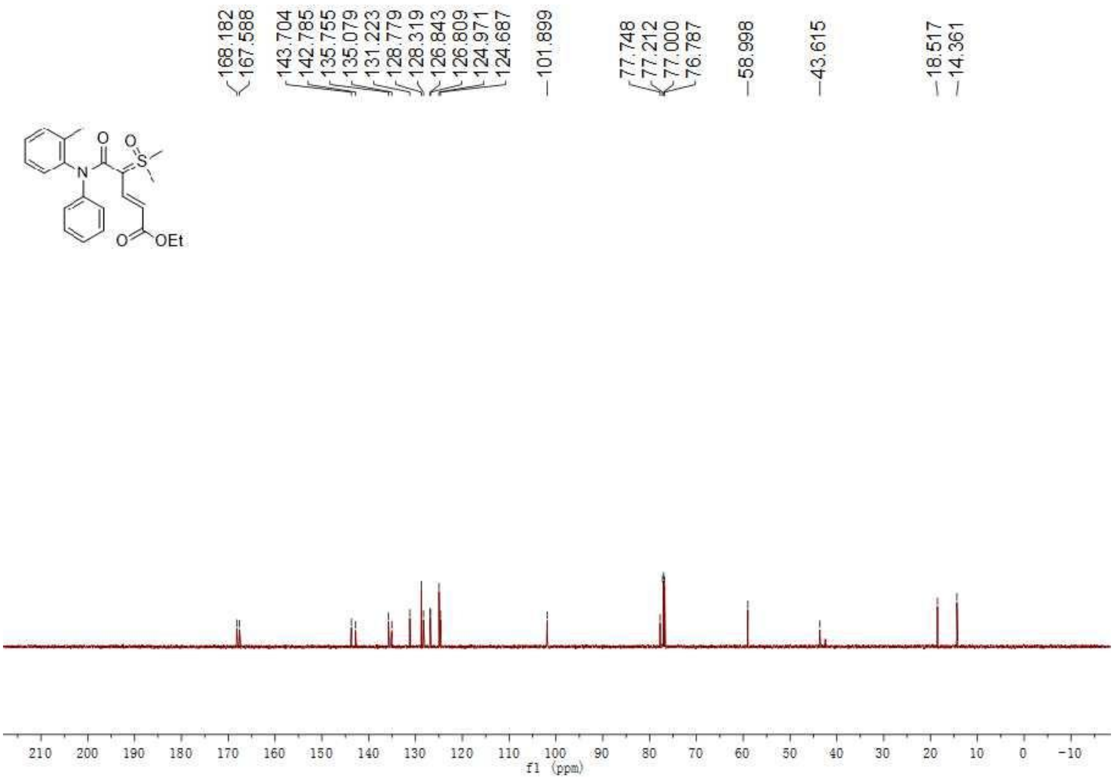

^{1}H NMR (600 MHz, $CDCl_3$) Spectrum of **11**

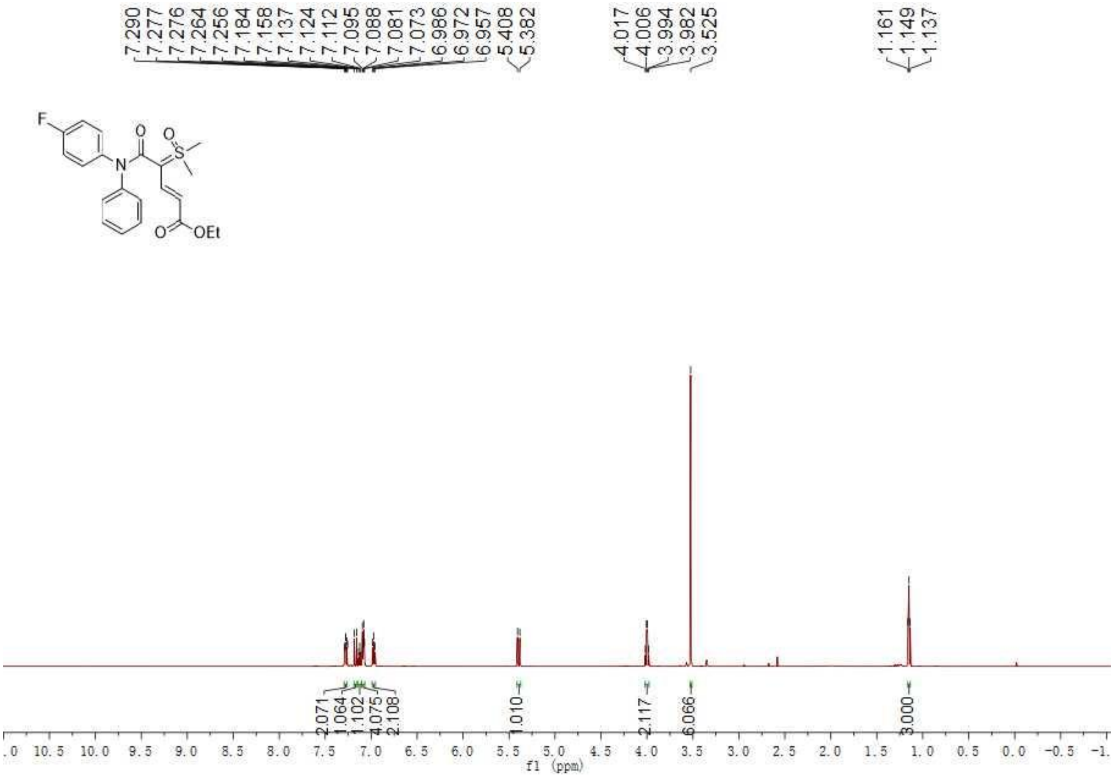

^{13}C NMR (150 MHz, $CDCl_3$) Spectrum of **11**

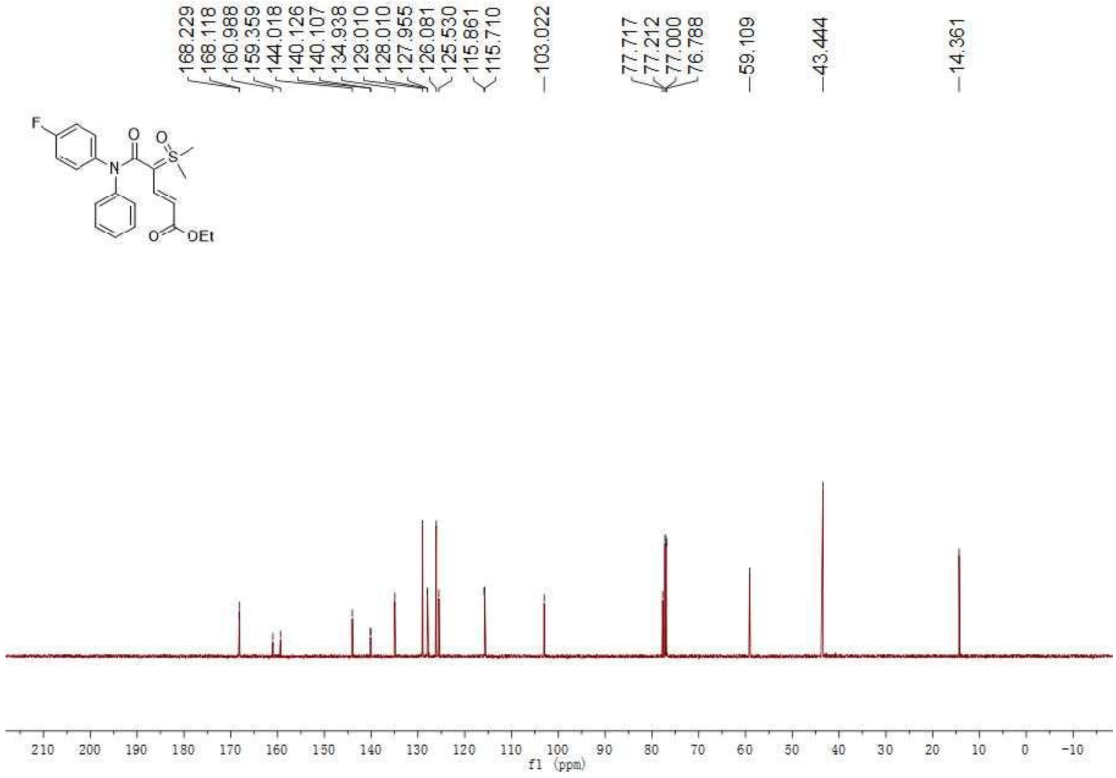

^{19}F NMR (564 MHz, $CDCl_3$) Spectrum of **11**

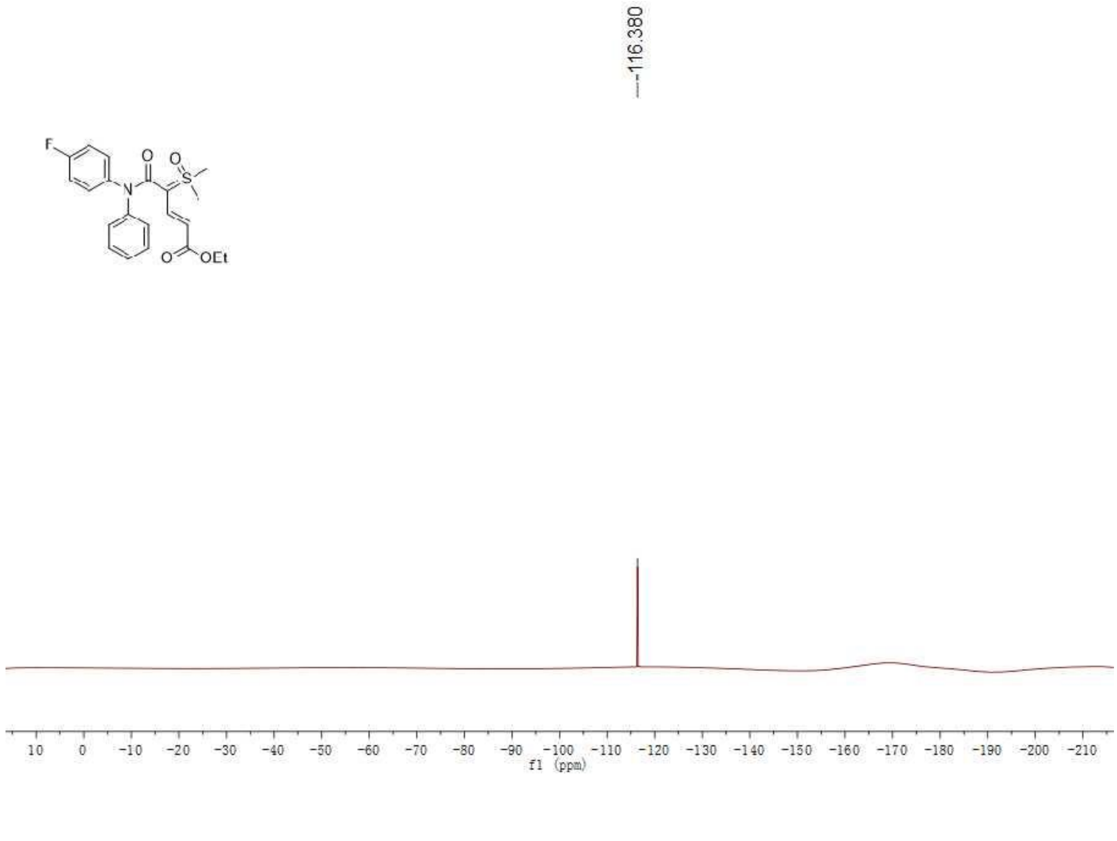

^{1}H NMR (600 MHz, $CDCl_3$) Spectrum of **12**

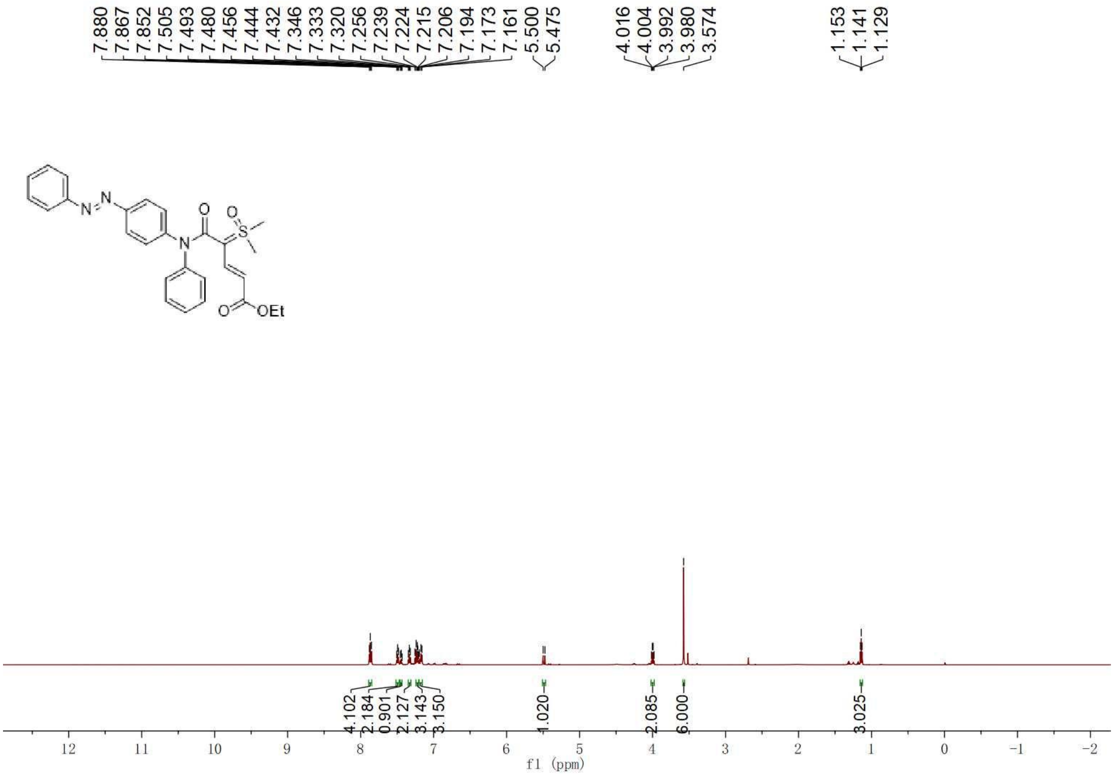

^{13}C NMR (150 MHz, $CDCl_3$) Spectrum of **12**

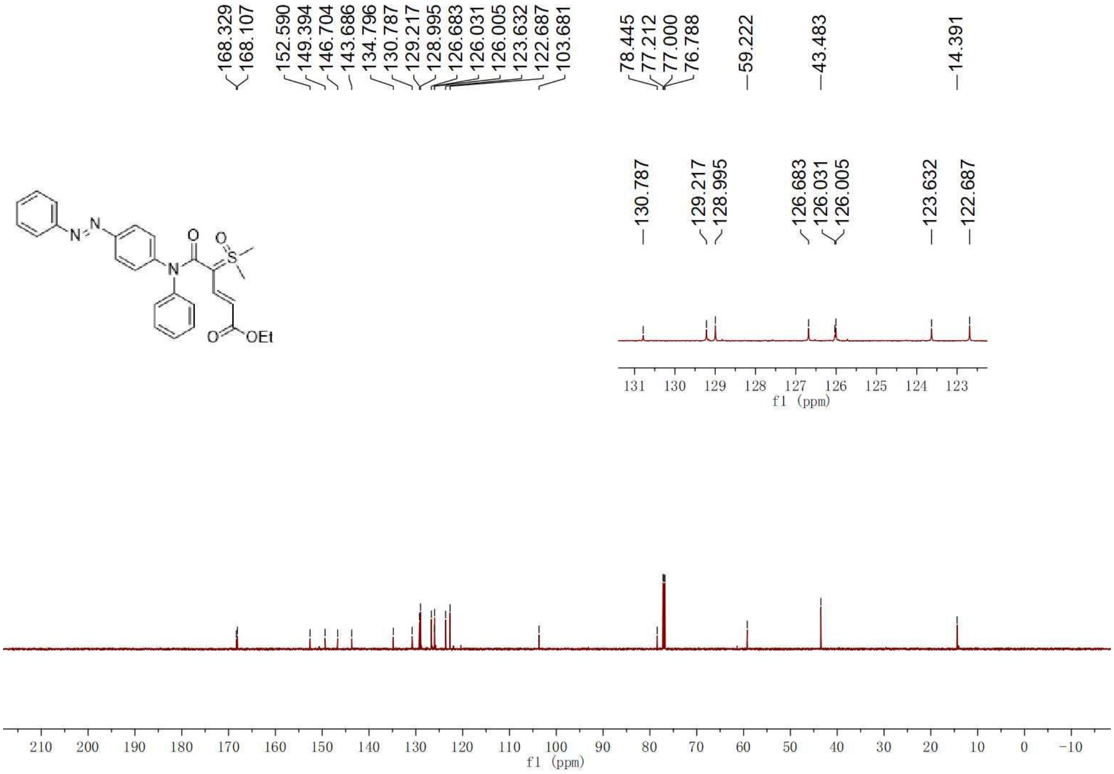

^{1}H NMR (600 MHz, $CDCl_3$) Spectrum of **13**

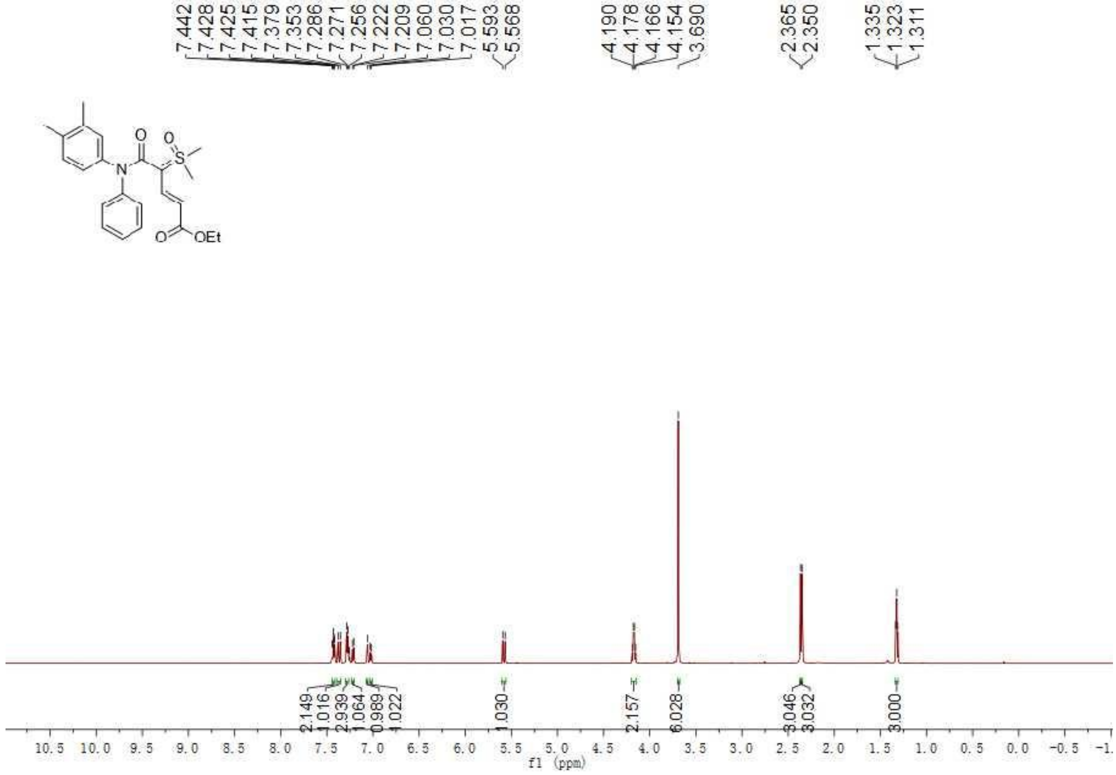

^{13}C NMR (150 MHz, $CDCl_3$) Spectrum of **13**

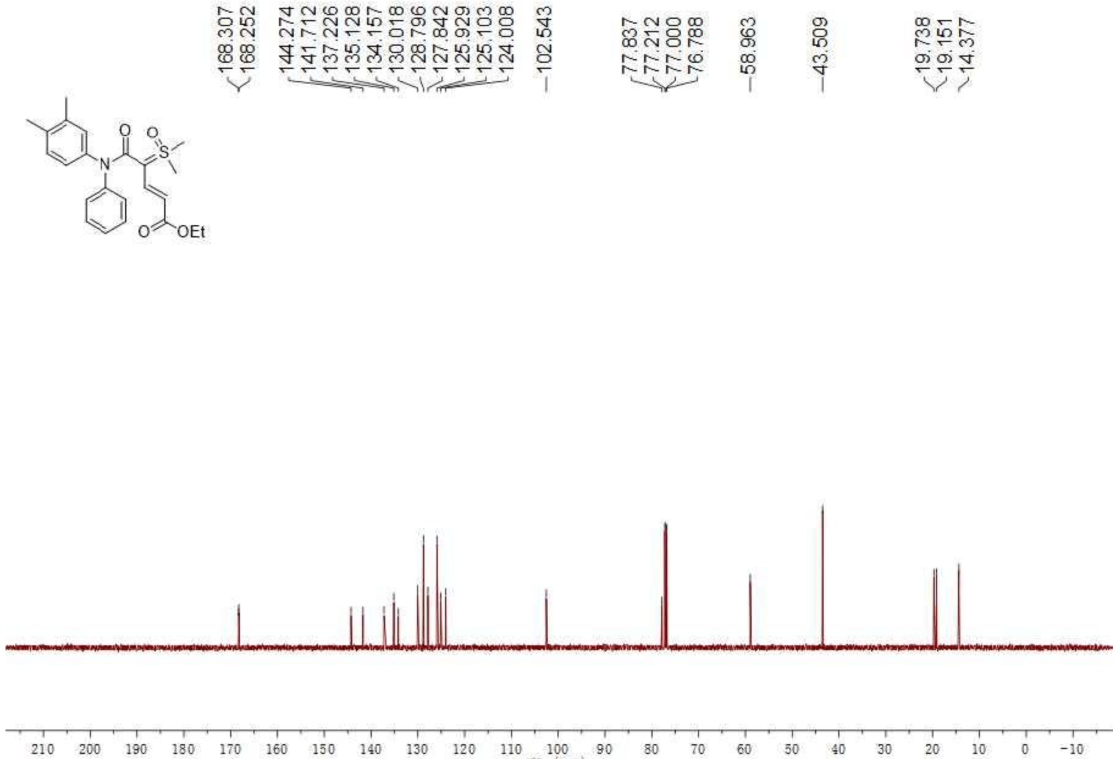

^{1}H NMR (600 MHz, $CDCl_3$) Spectrum of **14**

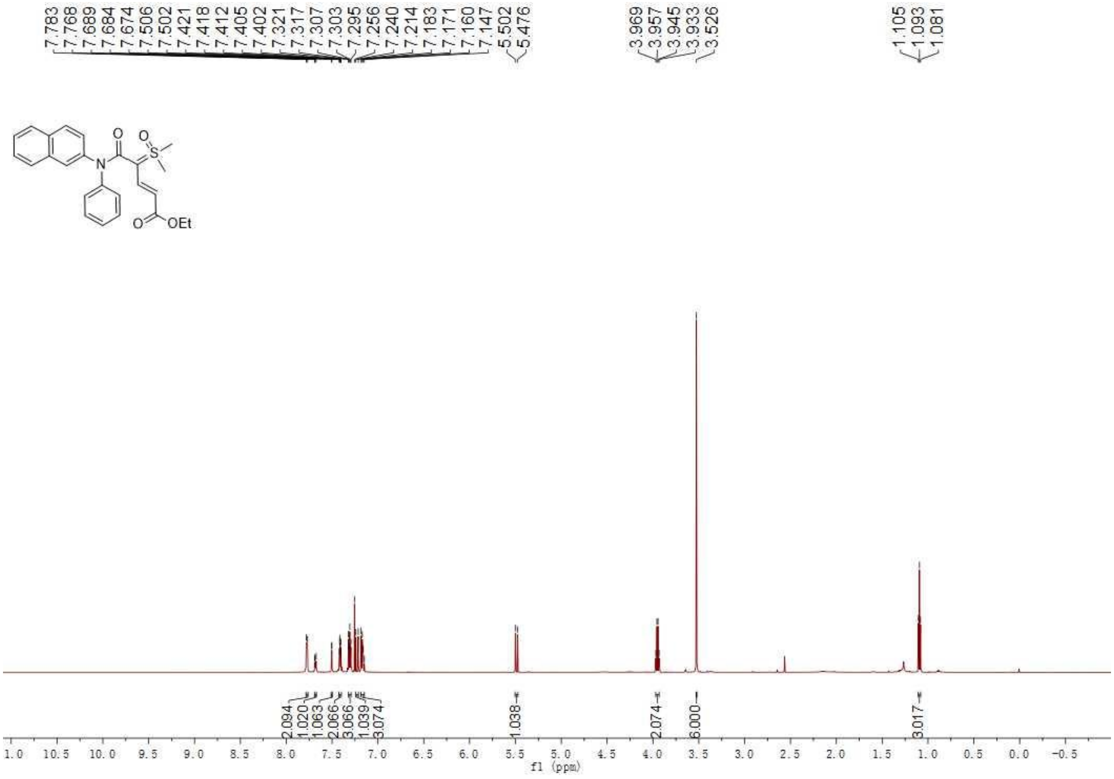

^{13}C NMR (150 MHz, $CDCl_3$) Spectrum of **14**

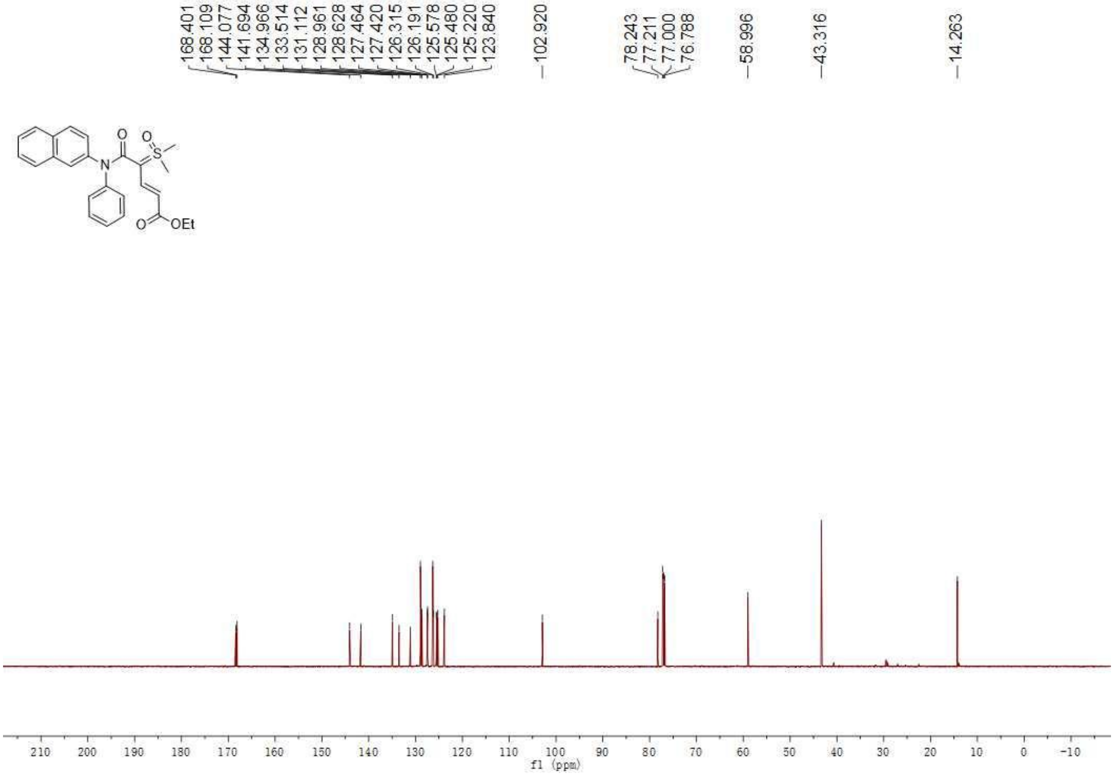

^{1}H NMR (600 MHz, $CDCl_3$) Spectrum of **15**

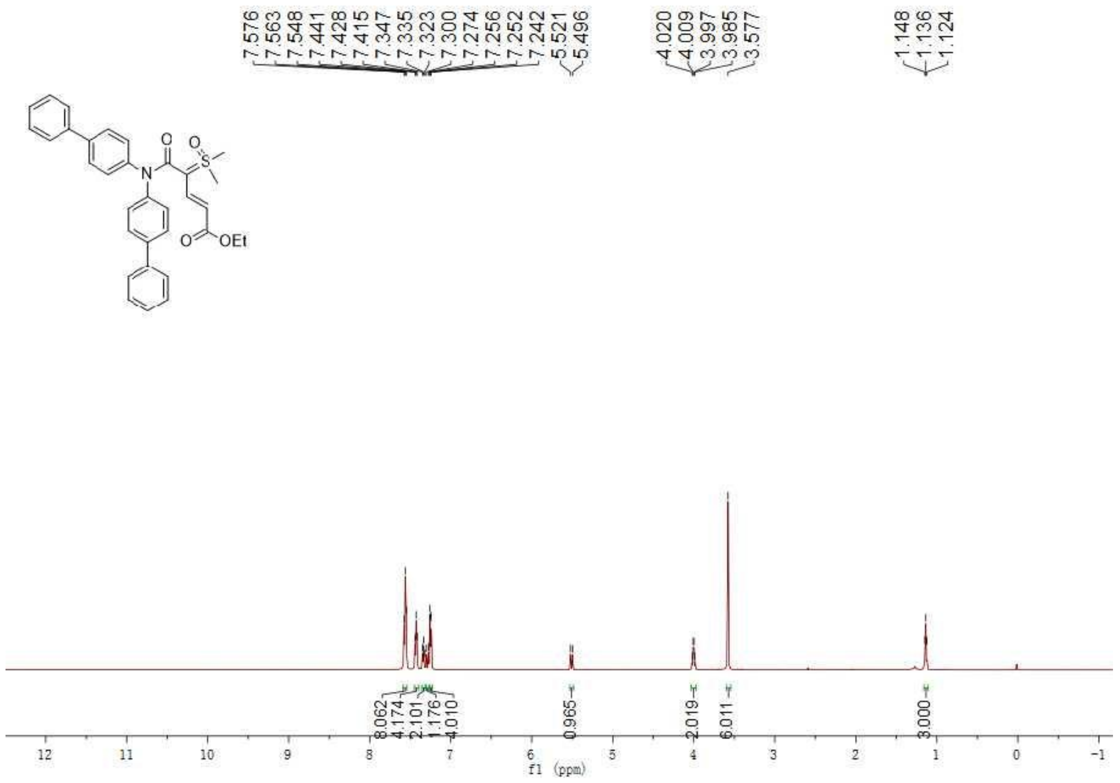

^{13}C NMR (150 MHz, $CDCl_3$) Spectrum of **15**

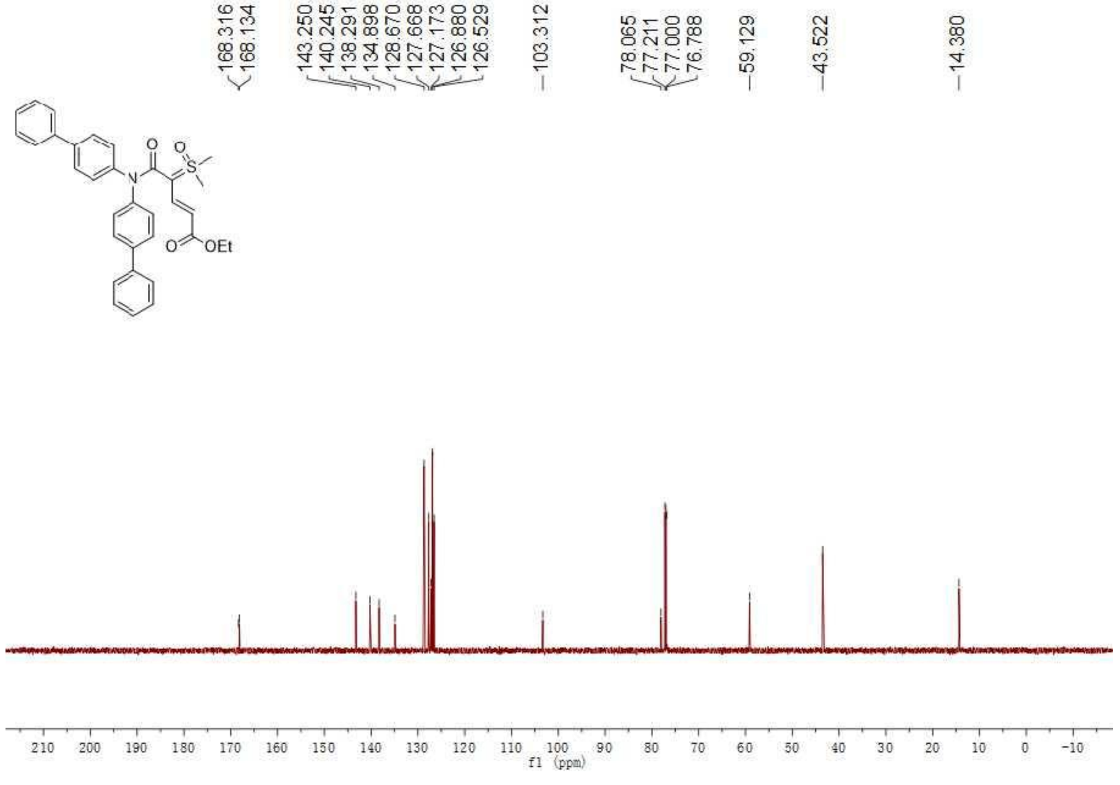

^{1}H NMR (600 MHz, $CDCl_3$) Spectrum of **16**

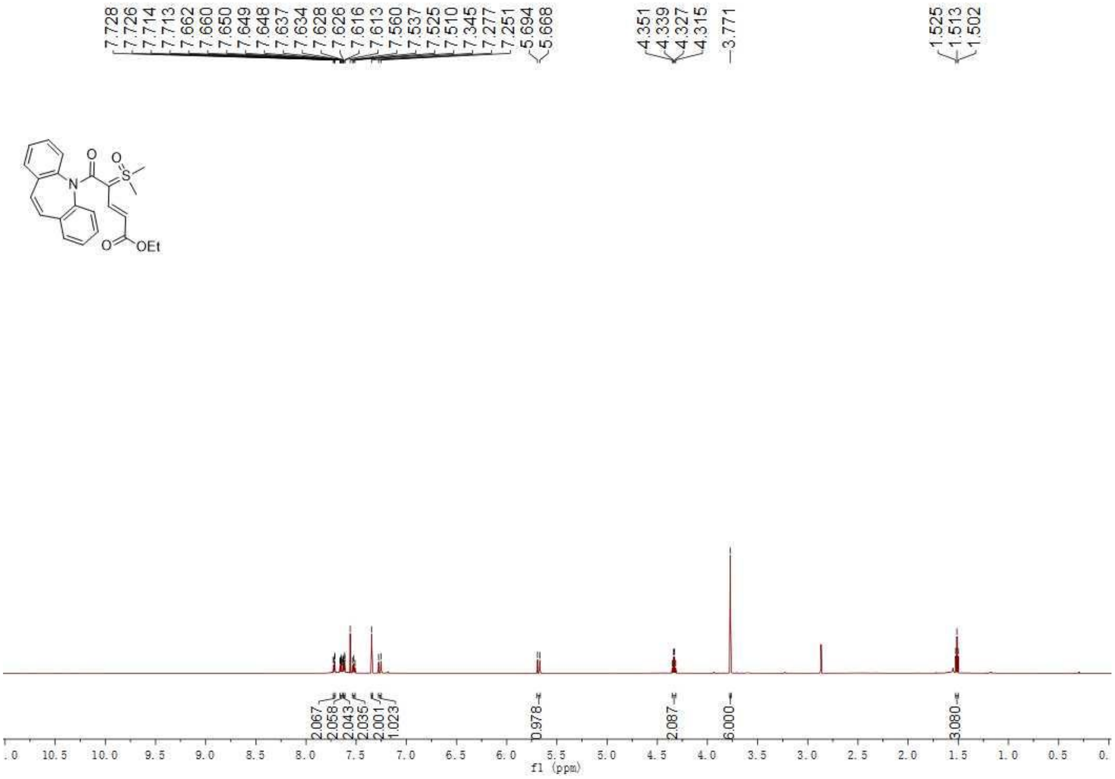

^{13}C NMR (150 MHz, $CDCl_3$) Spectrum of **16**

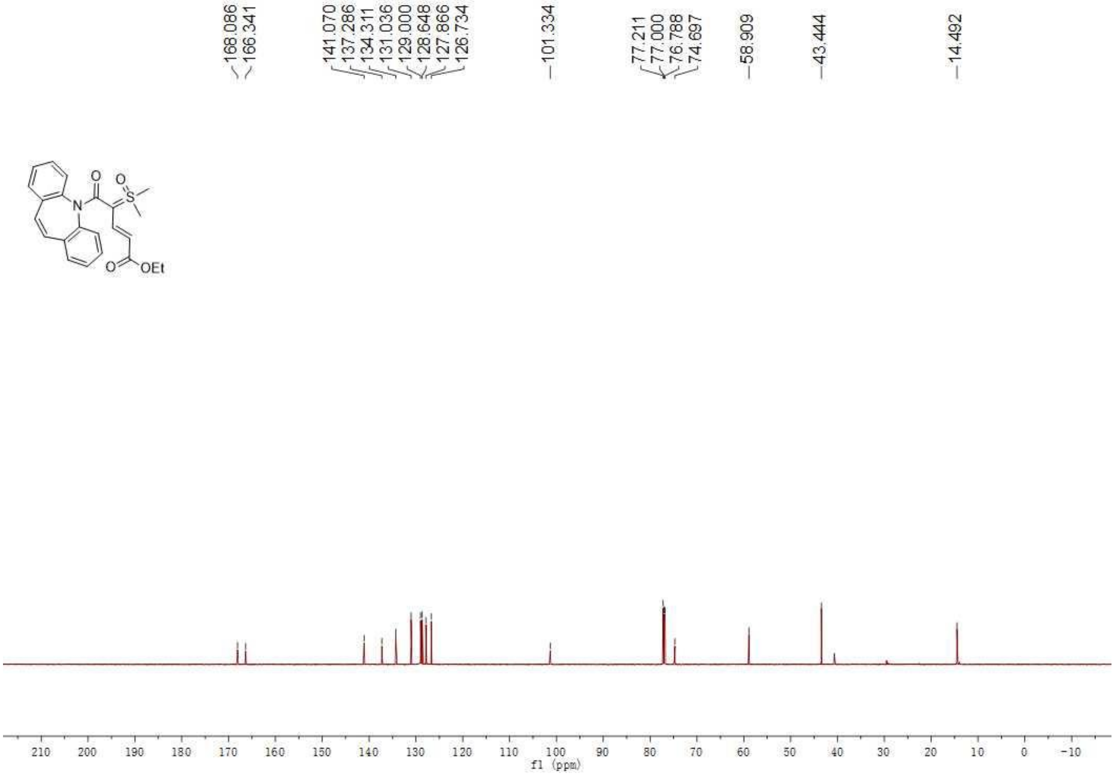

^{1}H NMR (600 MHz, $CDCl_3$) Spectrum of **17**

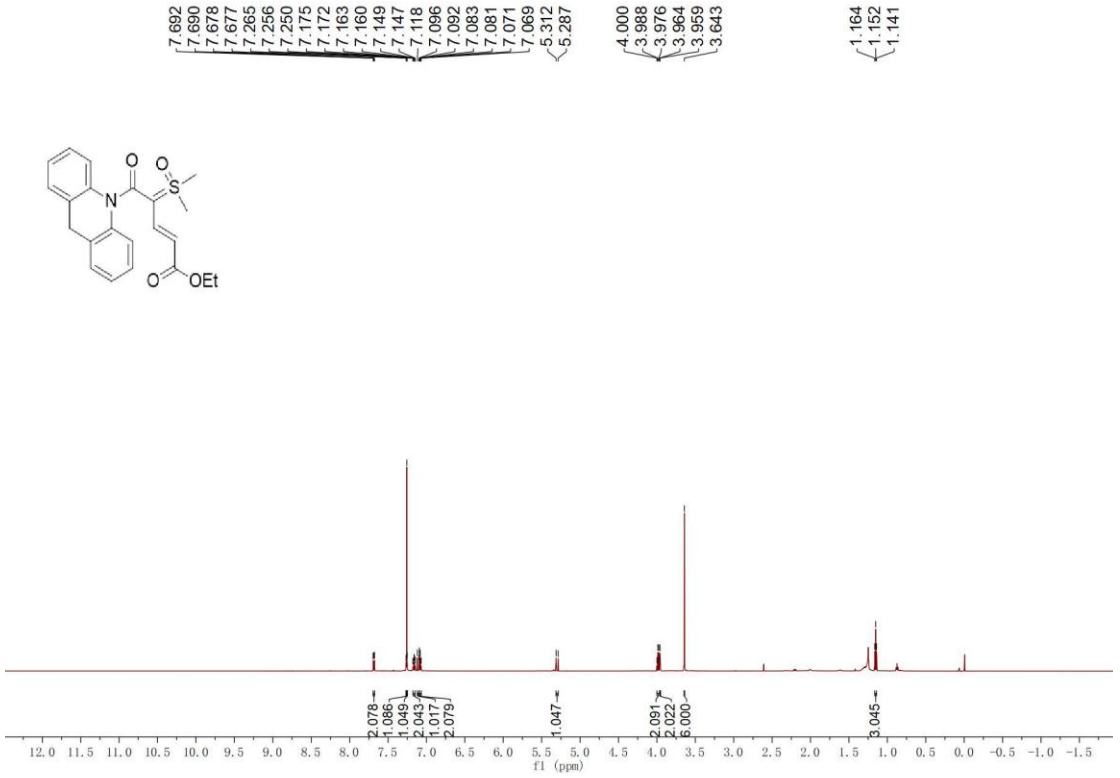

^{13}C NMR (150 MHz, $CDCl_3$) Spectrum of **17**

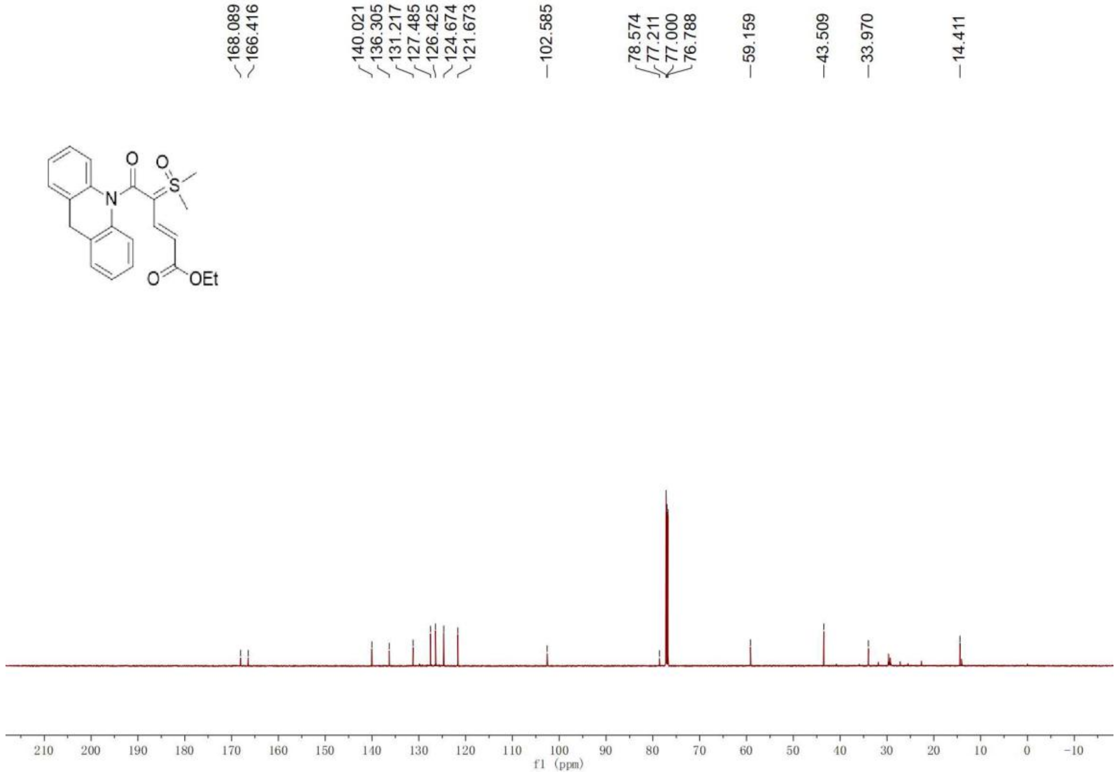

^{1}H NMR (600 MHz, $CDCl_3$) Spectrum of **18**

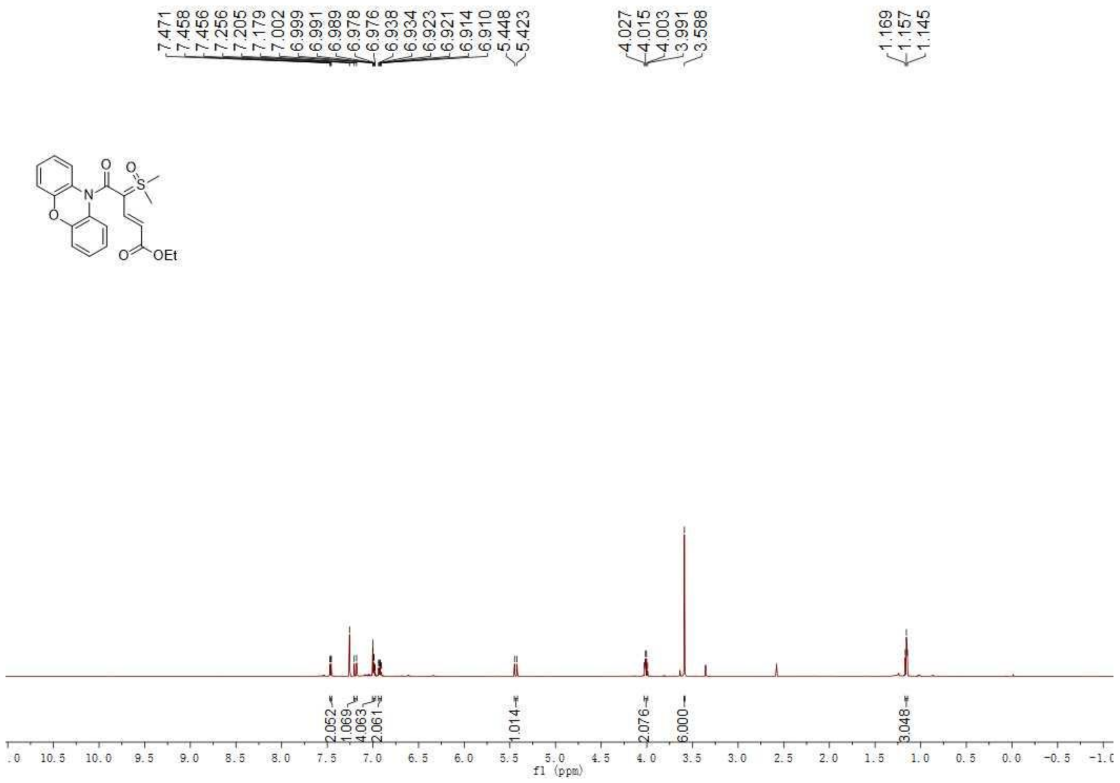

^{13}C NMR (150 MHz, $CDCl_3$) Spectrum of **18**

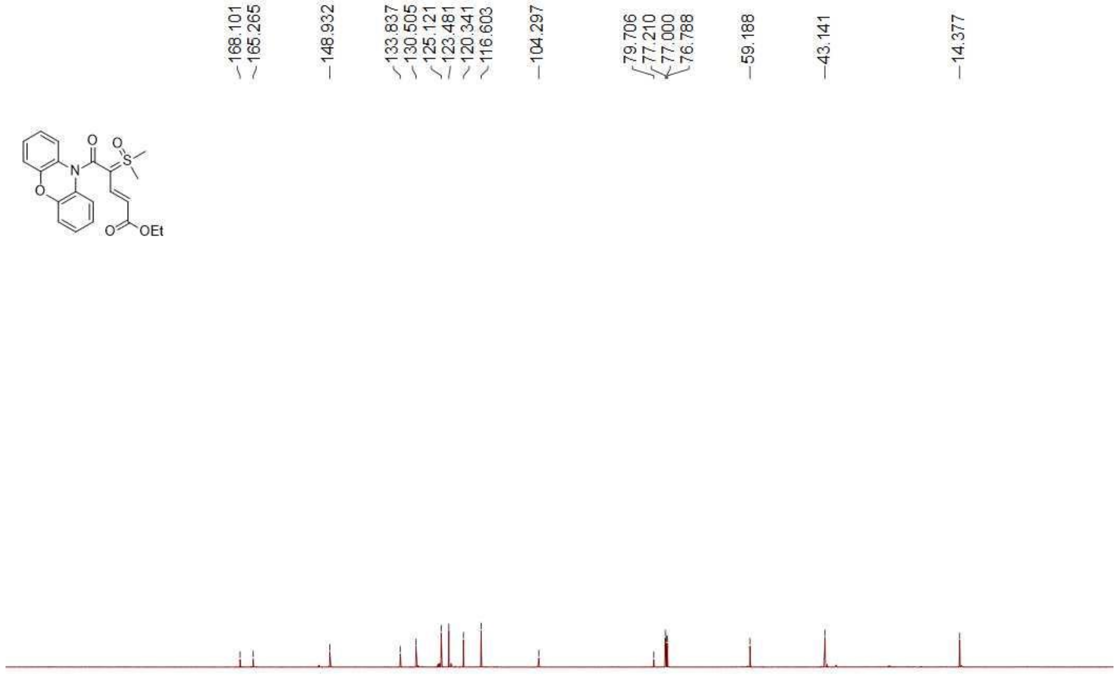

^{1}H NMR (600 MHz, $CDCl_3$) Spectrum of **19**

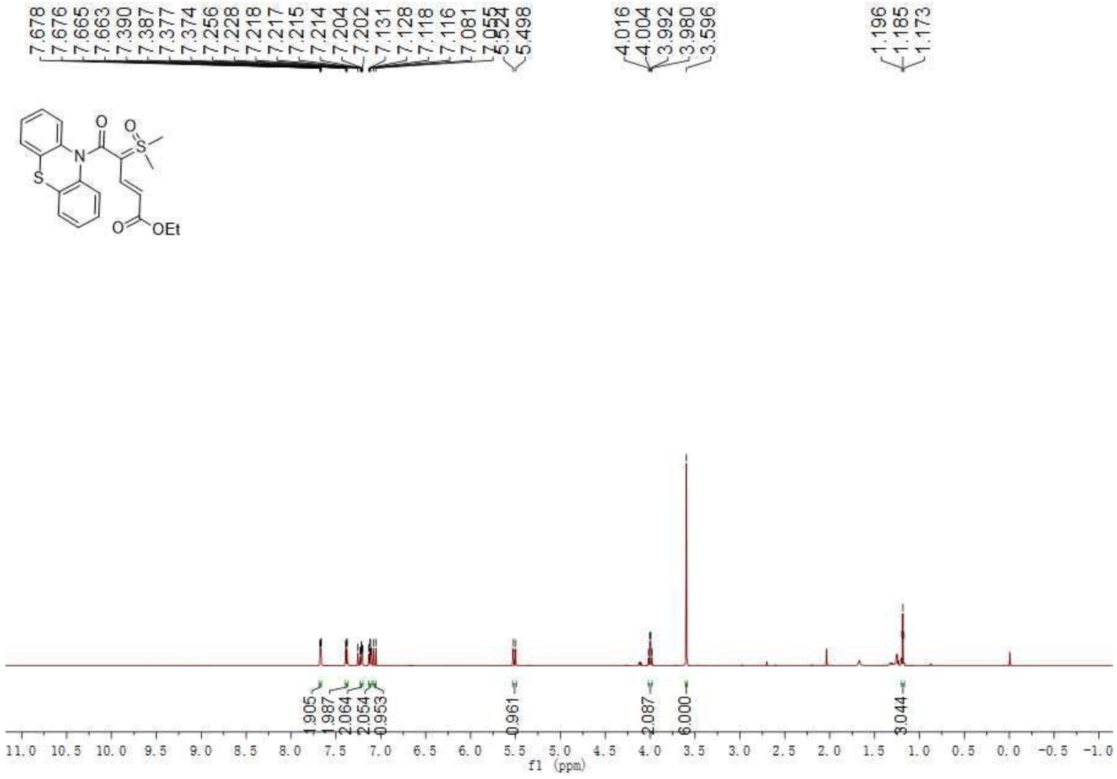

^{13}C NMR (150 MHz, $CDCl_3$) Spectrum of **19**

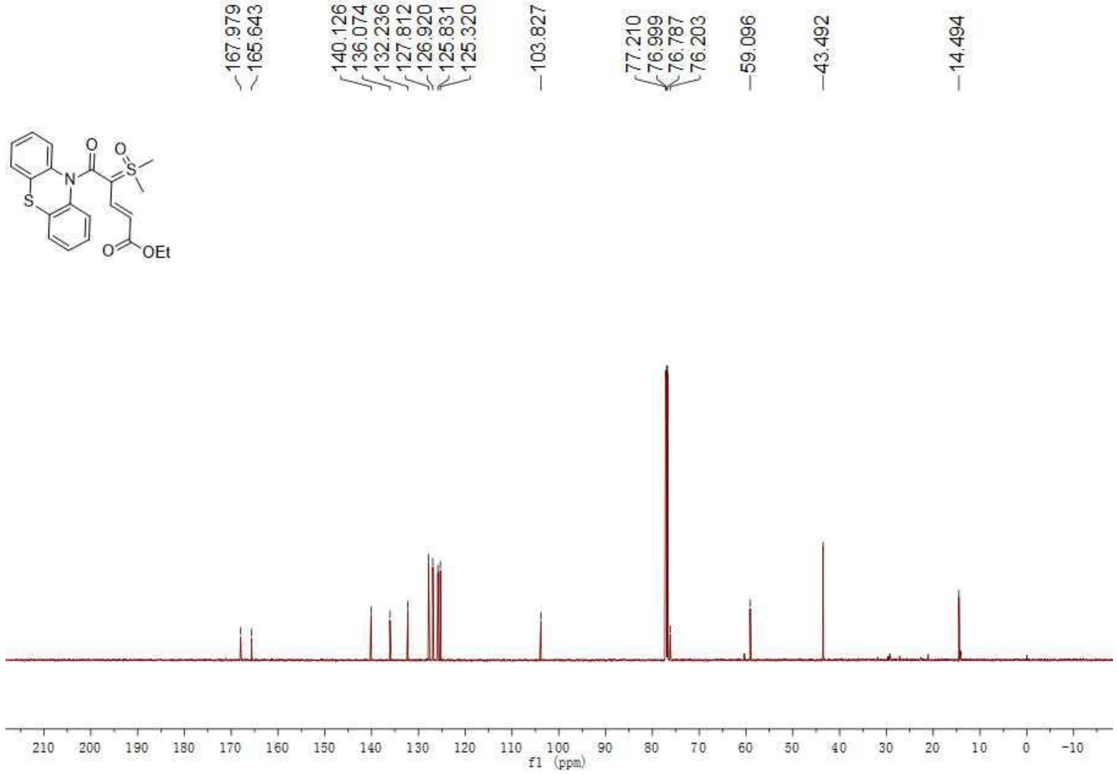

^{1}H NMR (600 MHz, $CDCl_3$) Spectrum of **20**

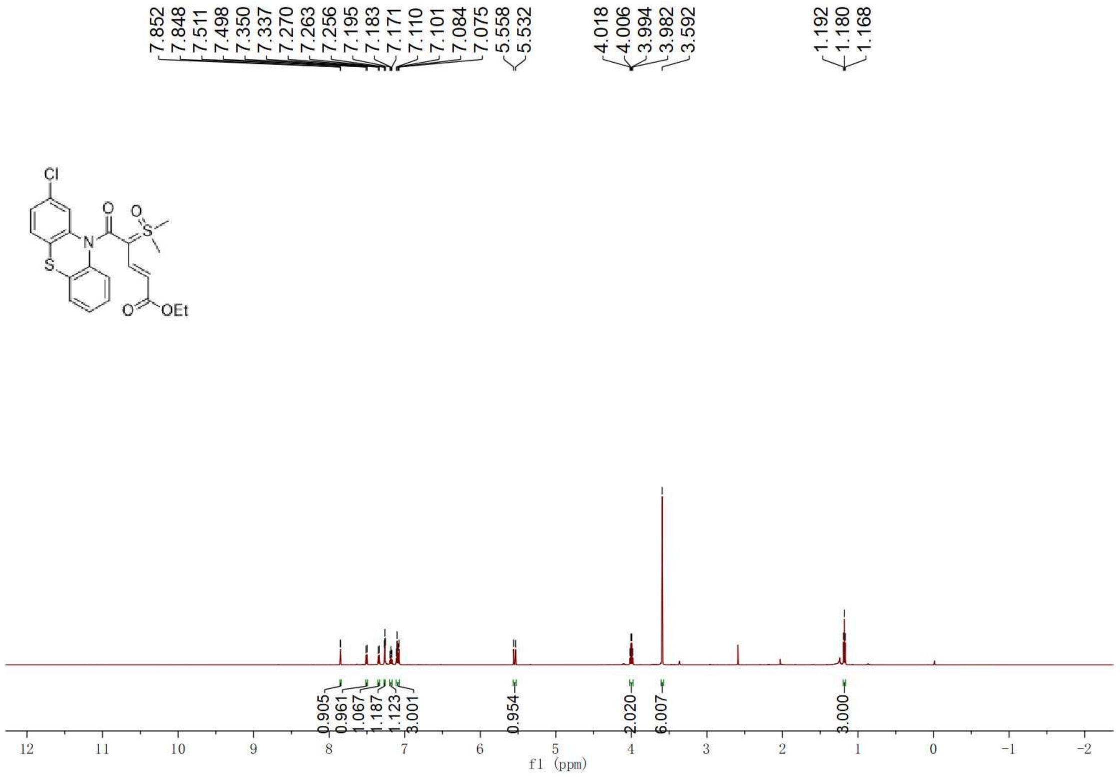

^{13}C NMR (150 MHz, $CDCl_3$) Spectrum of **20**

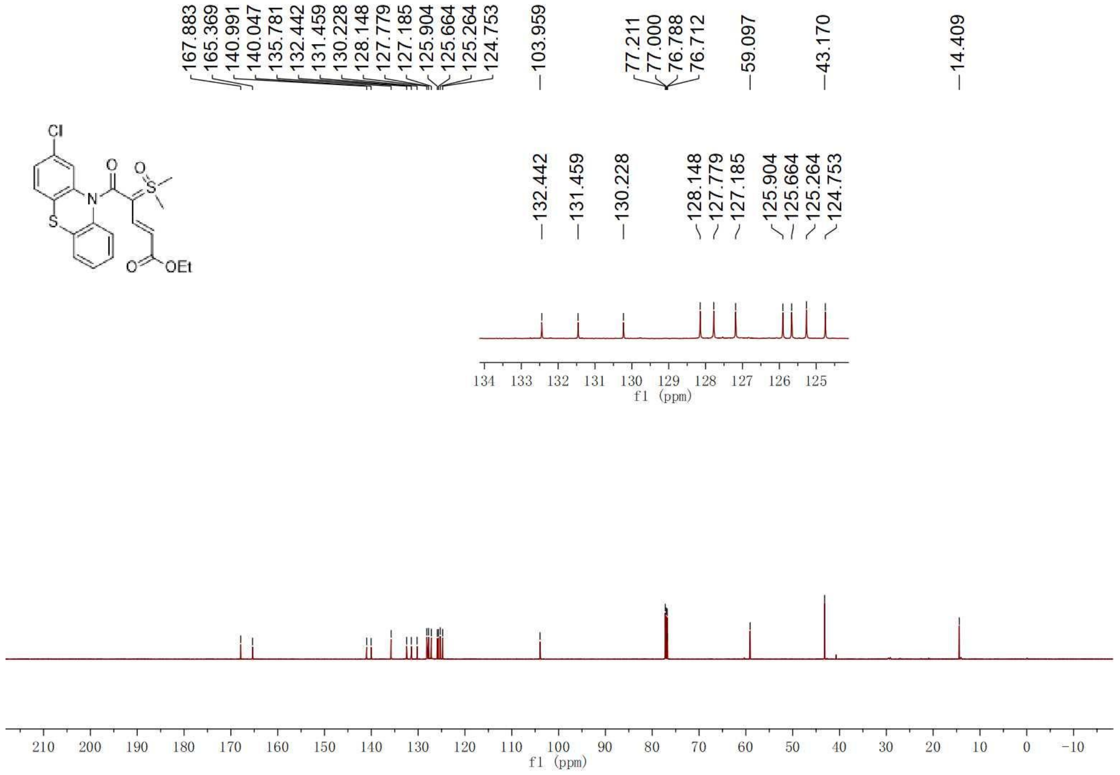

^{1}H NMR (600 MHz, $CDCl_3$) Spectrum of **21**

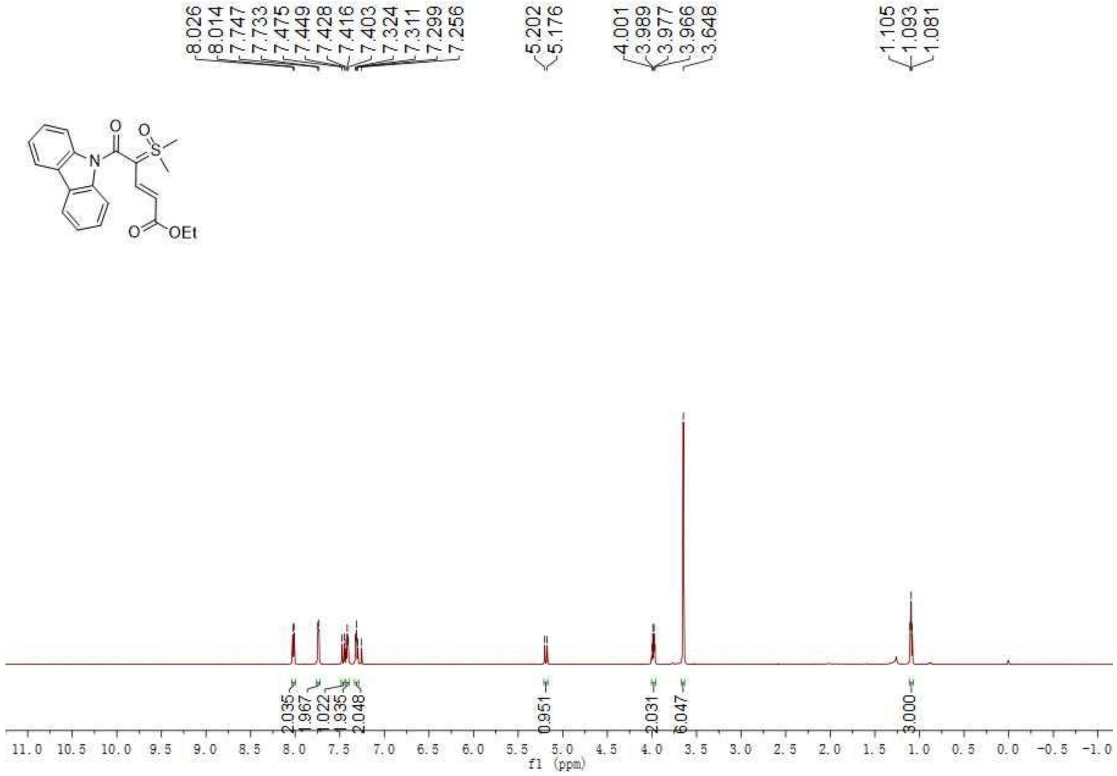

^{13}C NMR (150 MHz, $CDCl_3$) Spectrum of **21**

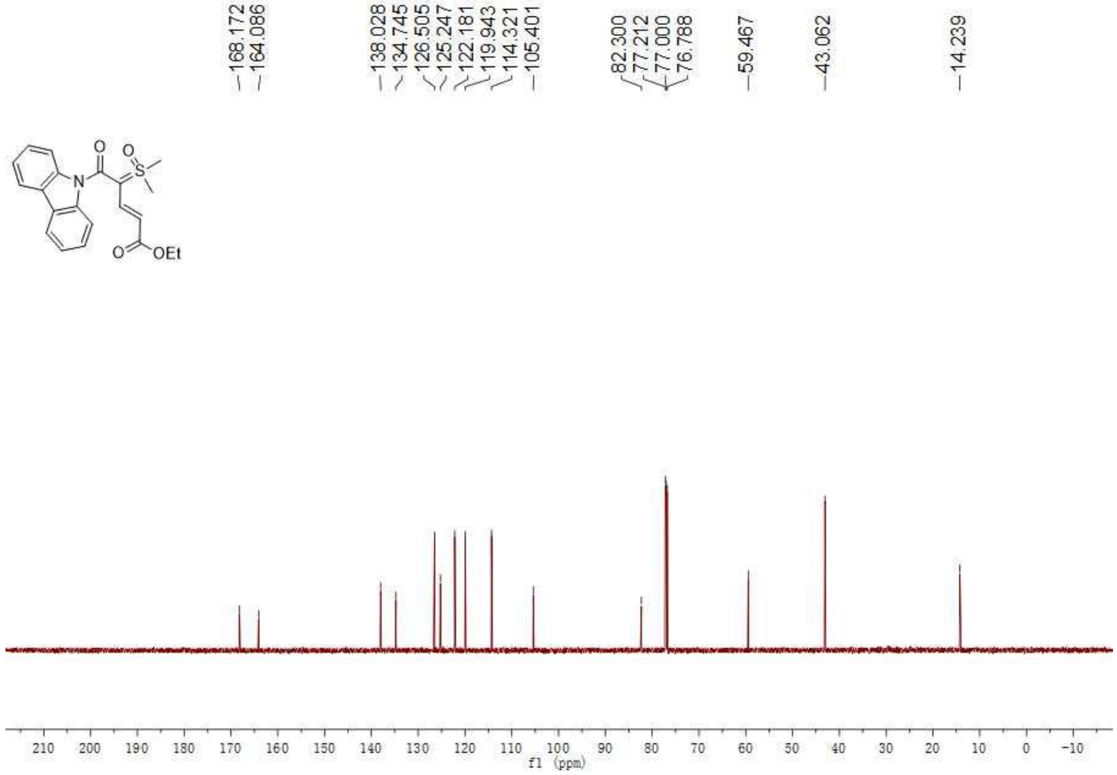

^{1}H NMR (600 MHz, $CDCl_3$) Spectrum of **22**

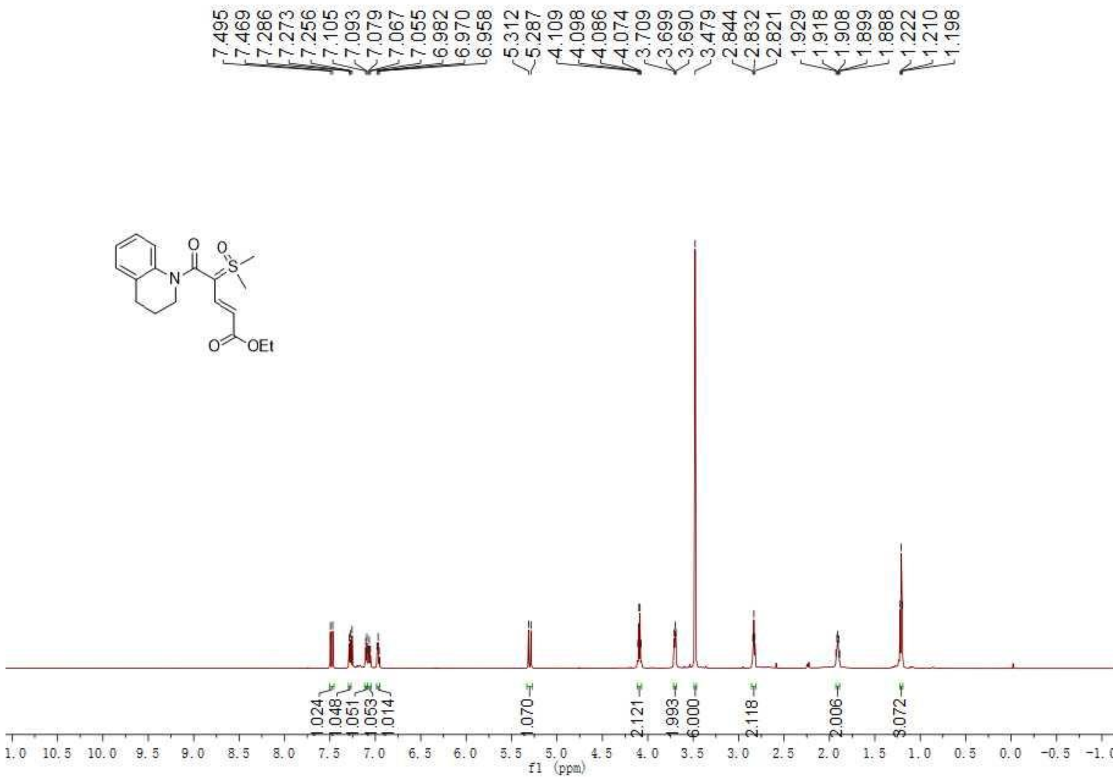

^{13}C NMR (150 MHz, $CDCl_3$) Spectrum of **22**

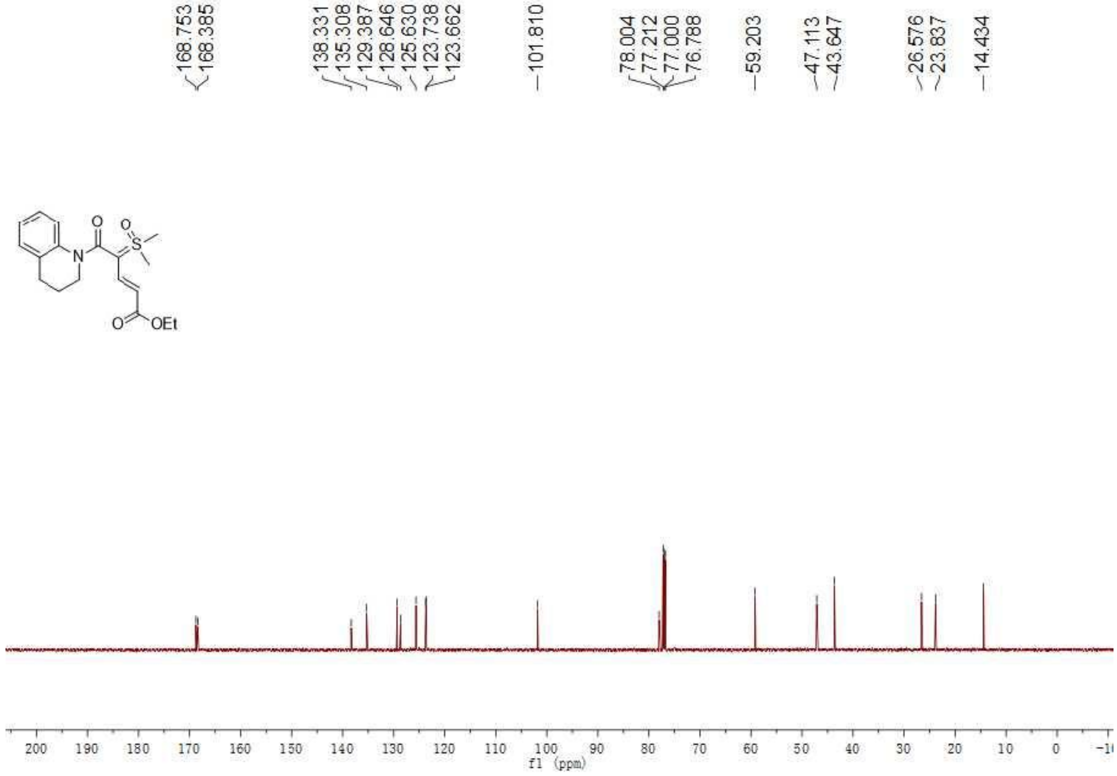

^{1}H NMR (600 MHz, $CDCl_3$) Spectrum of **23**

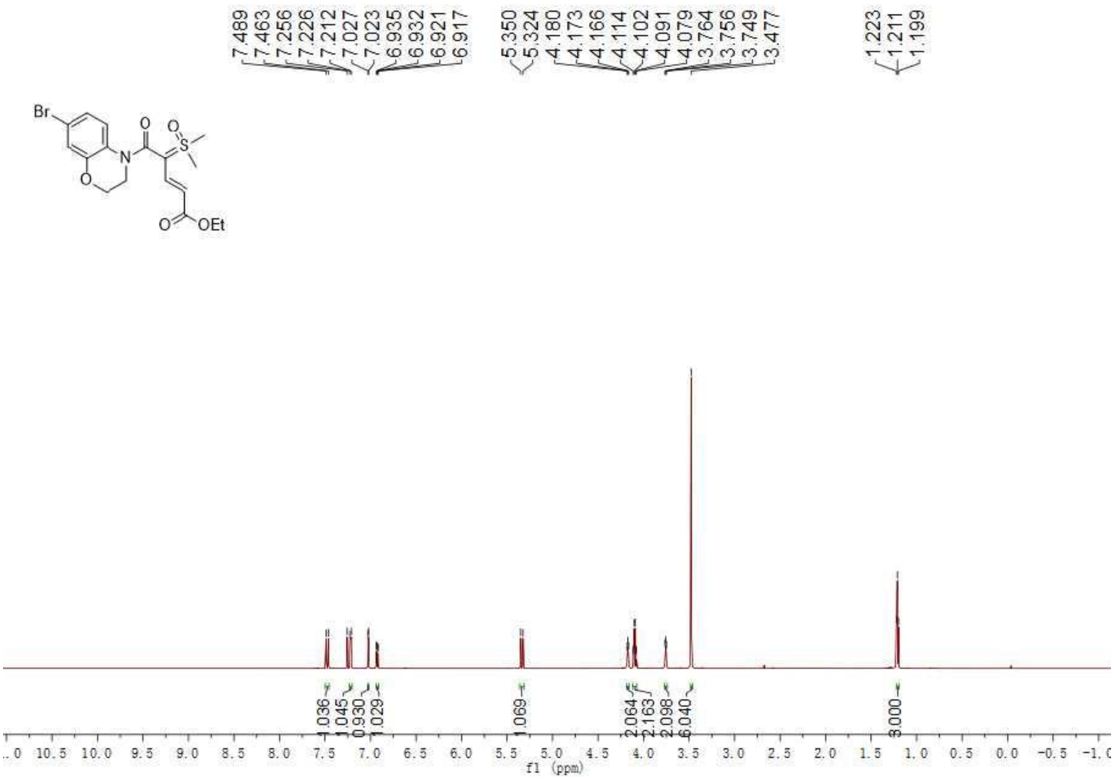

^{13}C NMR (150 MHz, $CDCl_3$) Spectrum of **23**

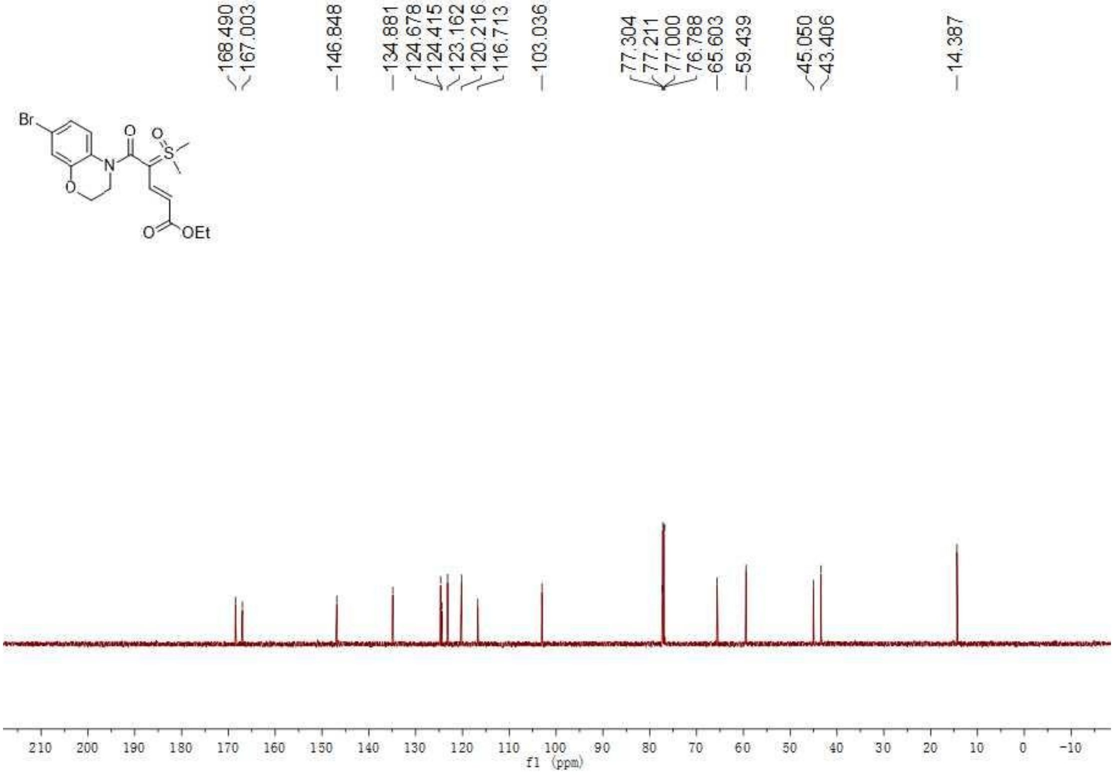

^{1}H NMR (600 MHz, $CDCl_3$) Spectrum of **24**

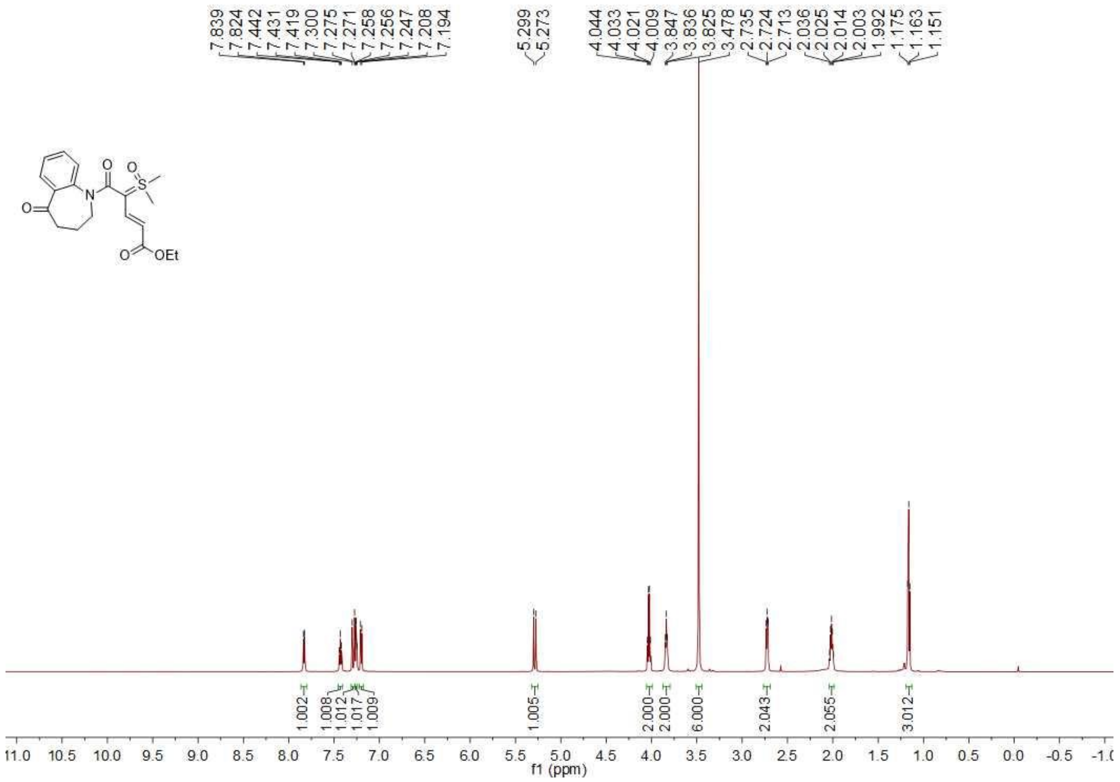

^{13}C NMR (150 MHz, $CDCl_3$) Spectrum of **24**

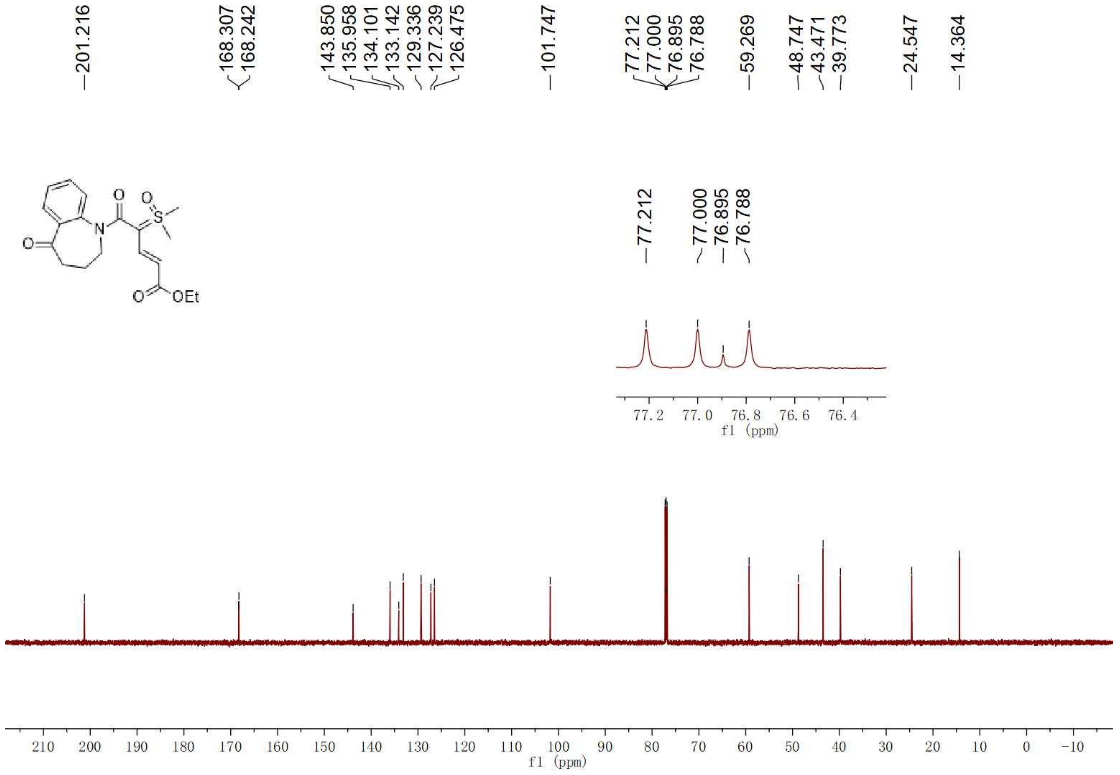

^{1}H NMR (600 MHz, $CDCl_3$) Spectrum of **25**

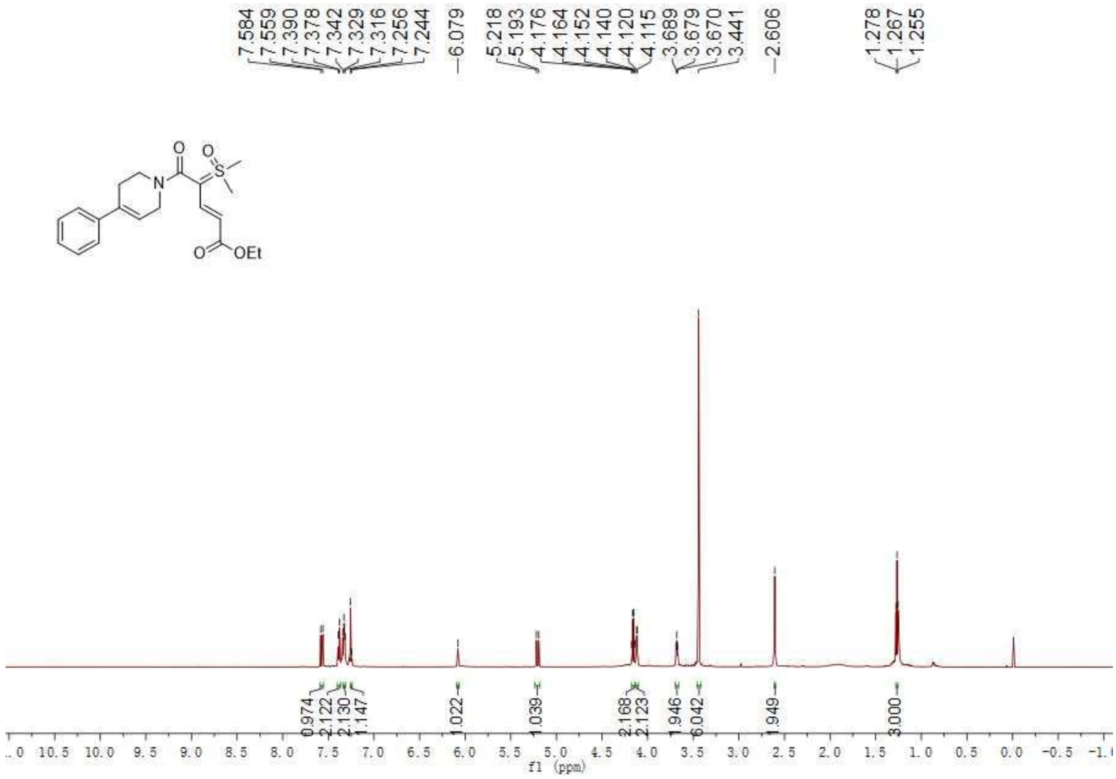

^{13}C NMR (150 MHz, $CDCl_3$) Spectrum of **25**

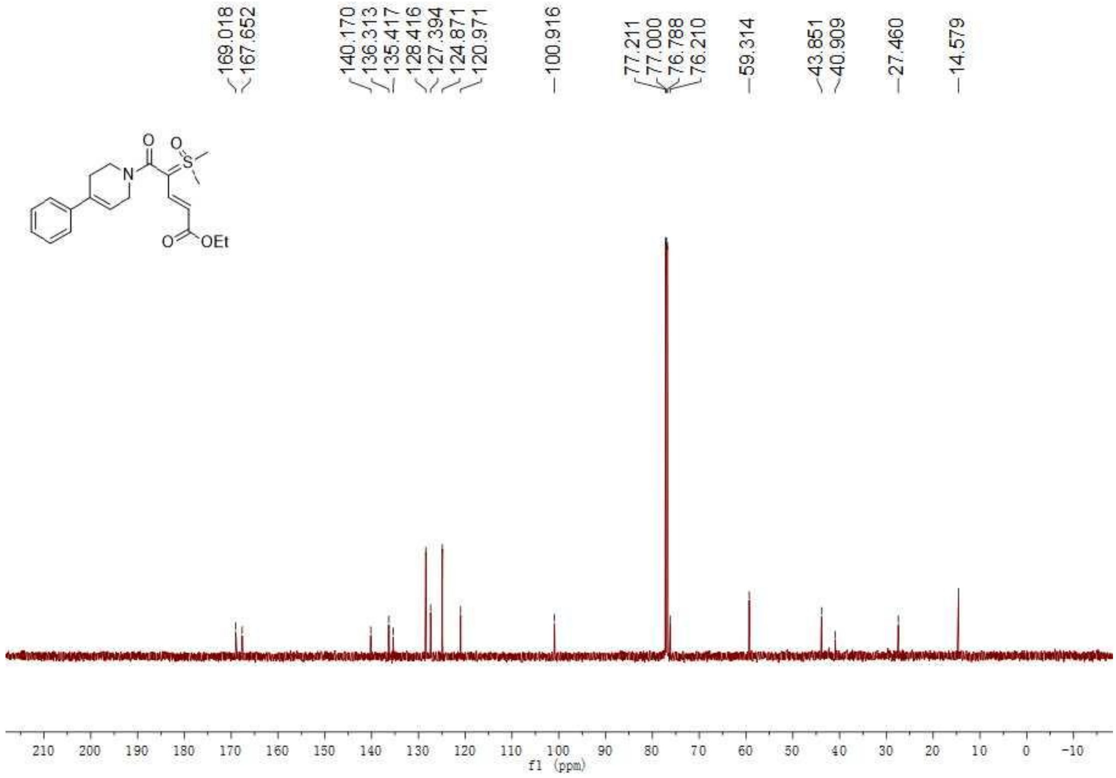

^{1}H NMR (600 MHz, $CDCl_3$) Spectrum of **26**

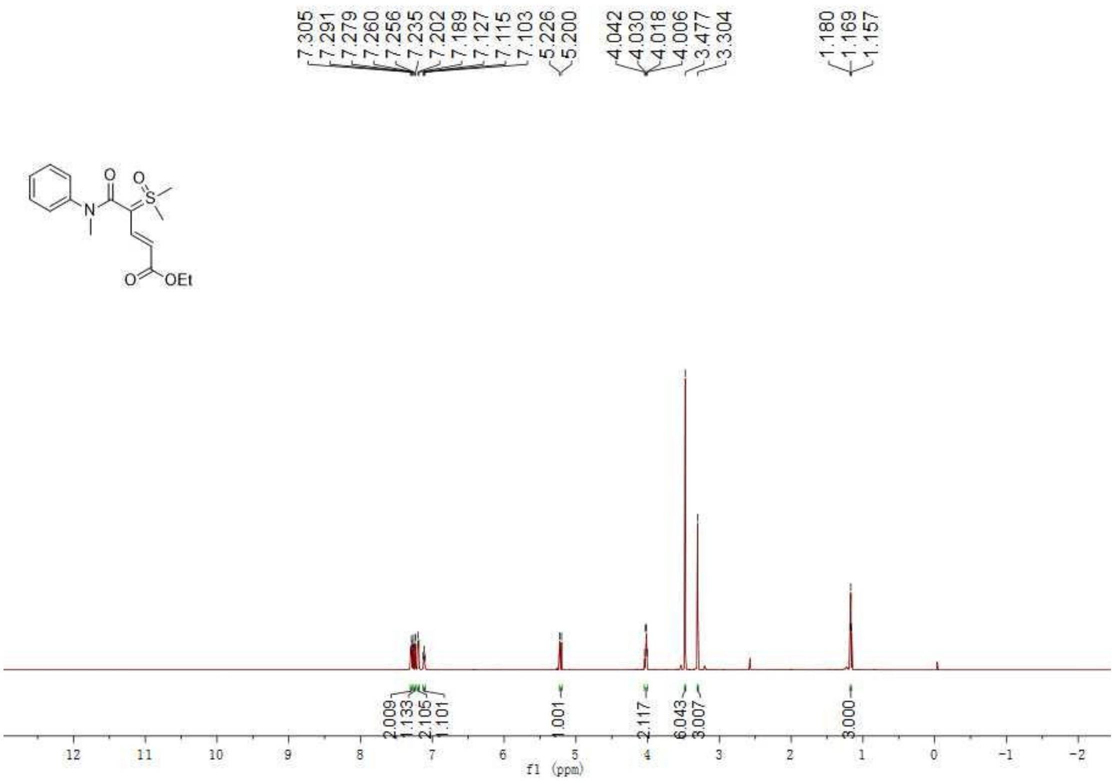

^{13}C NMR (150 MHz, $CDCl_3$) Spectrum of **26**

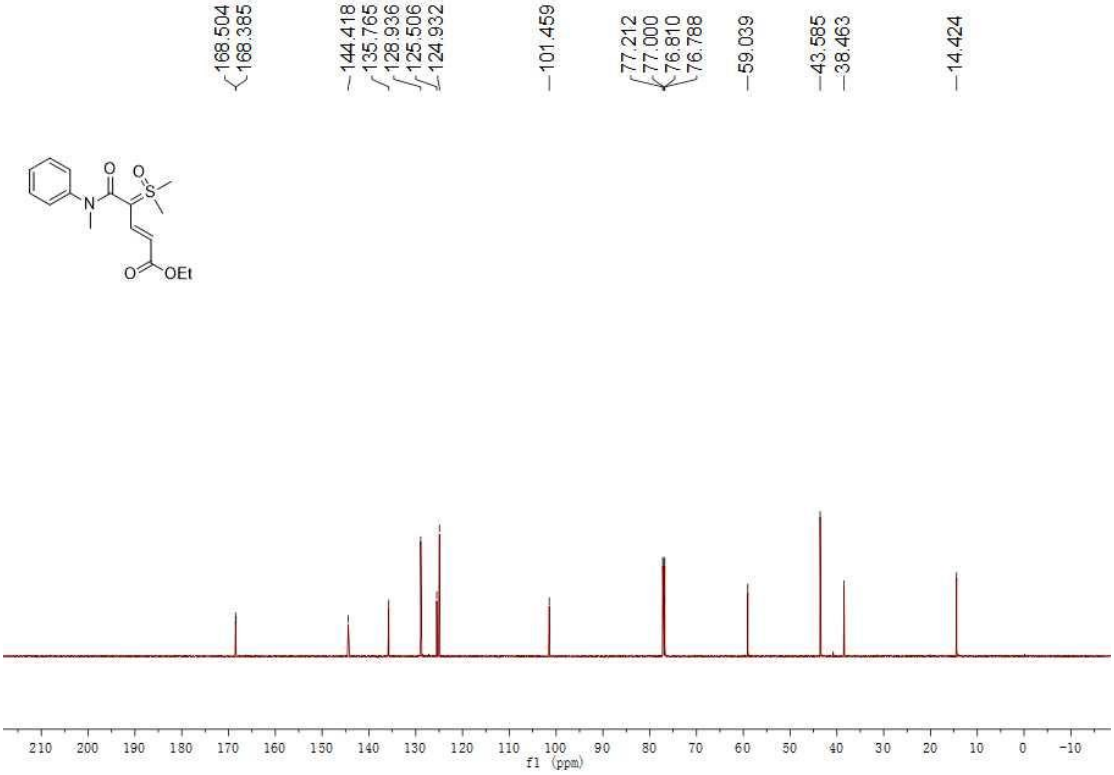

^{1}H NMR (600 MHz, $CDCl_3$) Spectrum of **27**

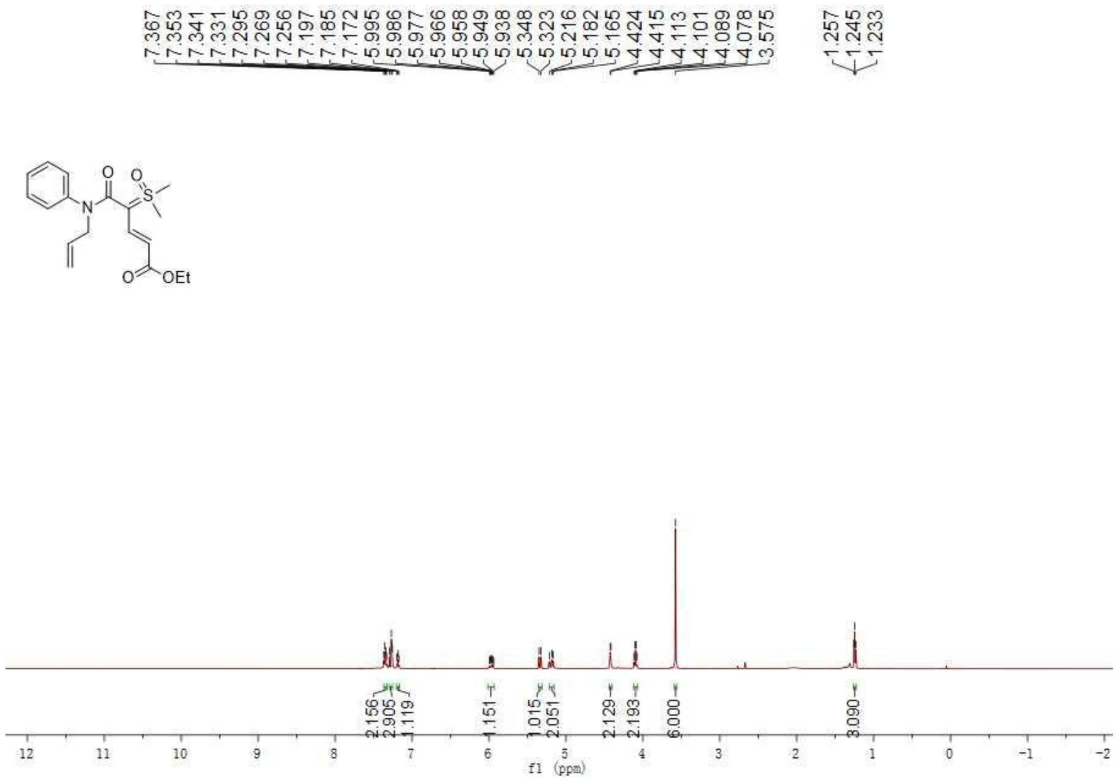

^{13}C NMR (150 MHz, $CDCl_3$) Spectrum of **27**

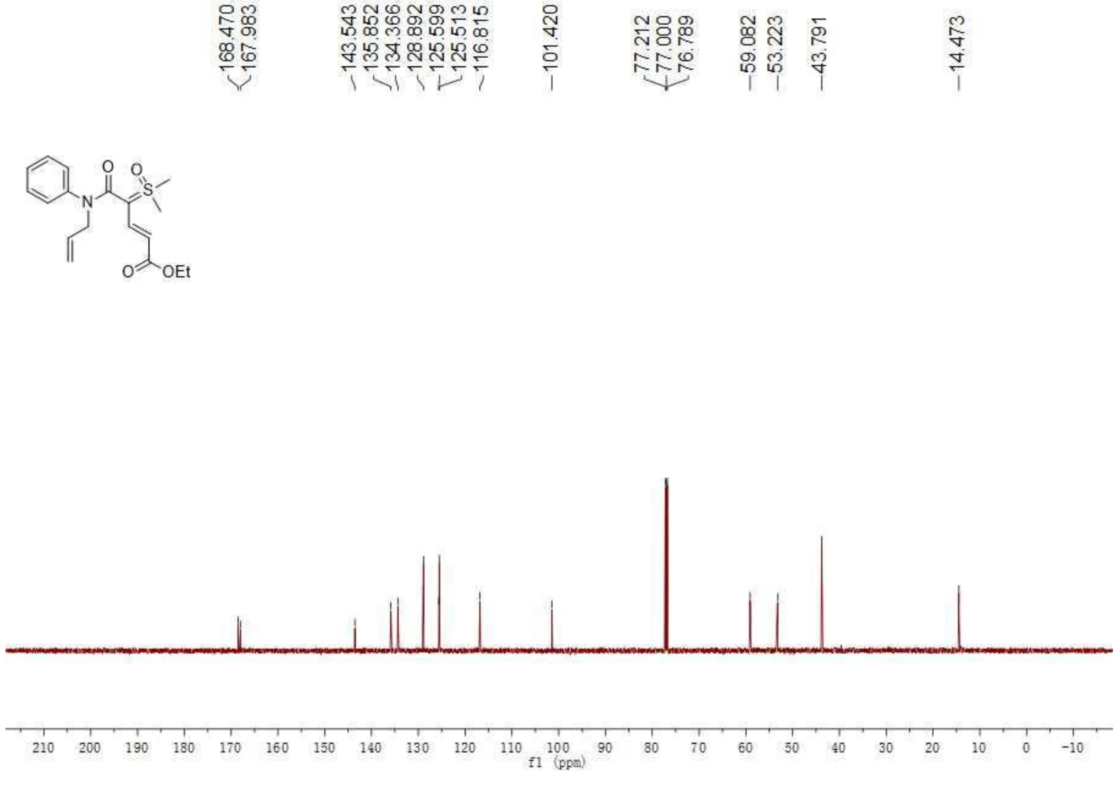

^{1}H NMR (600 MHz, $CDCl_3$) Spectrum of **28**

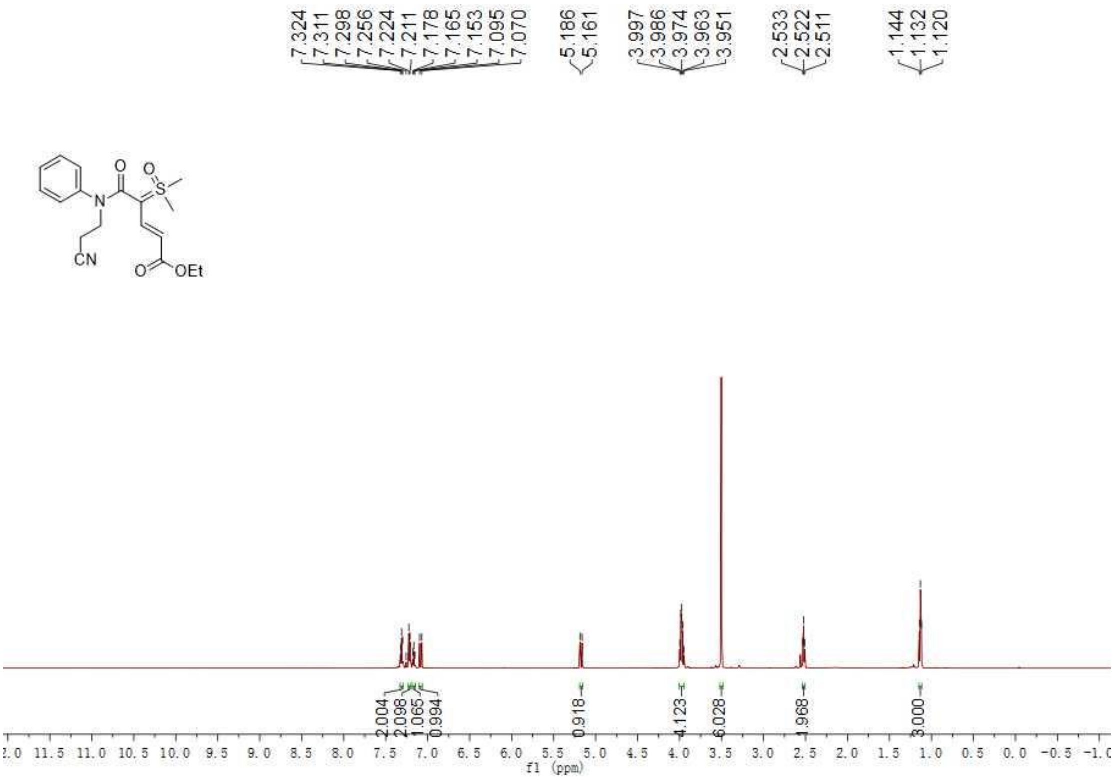

^{13}C NMR (150 MHz, $CDCl_3$) Spectrum of **28**

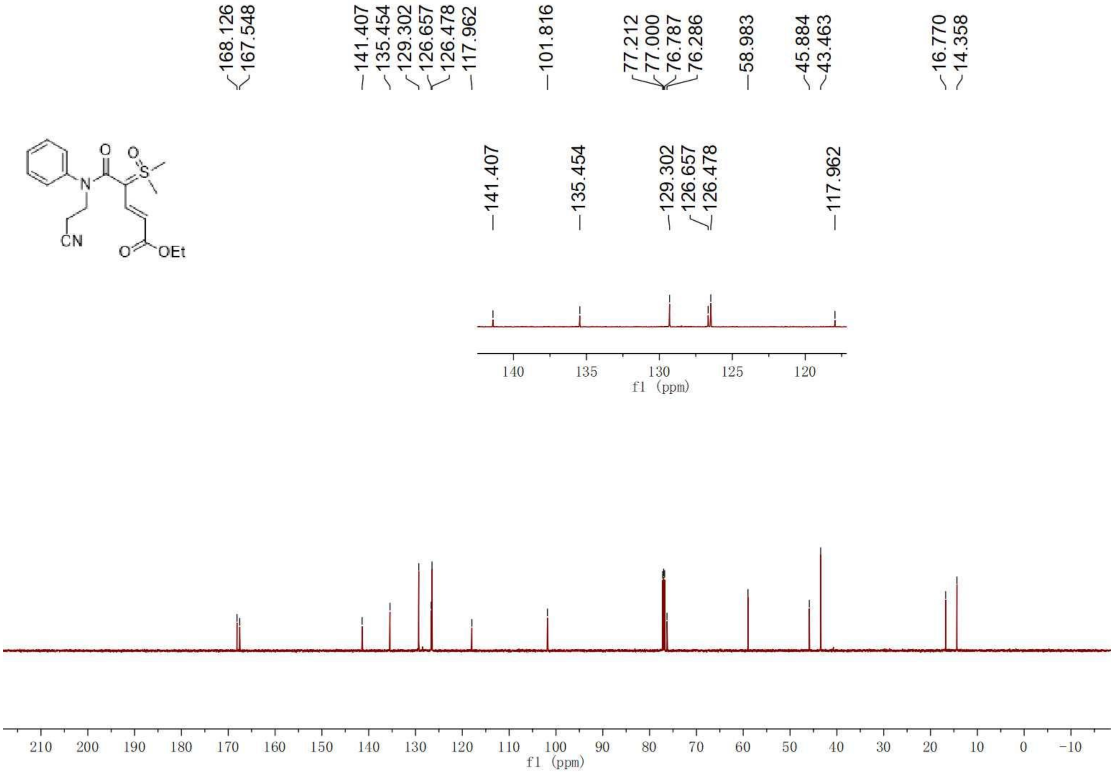

^{1}H NMR (600 MHz, $CDCl_3$) Spectrum of **29**

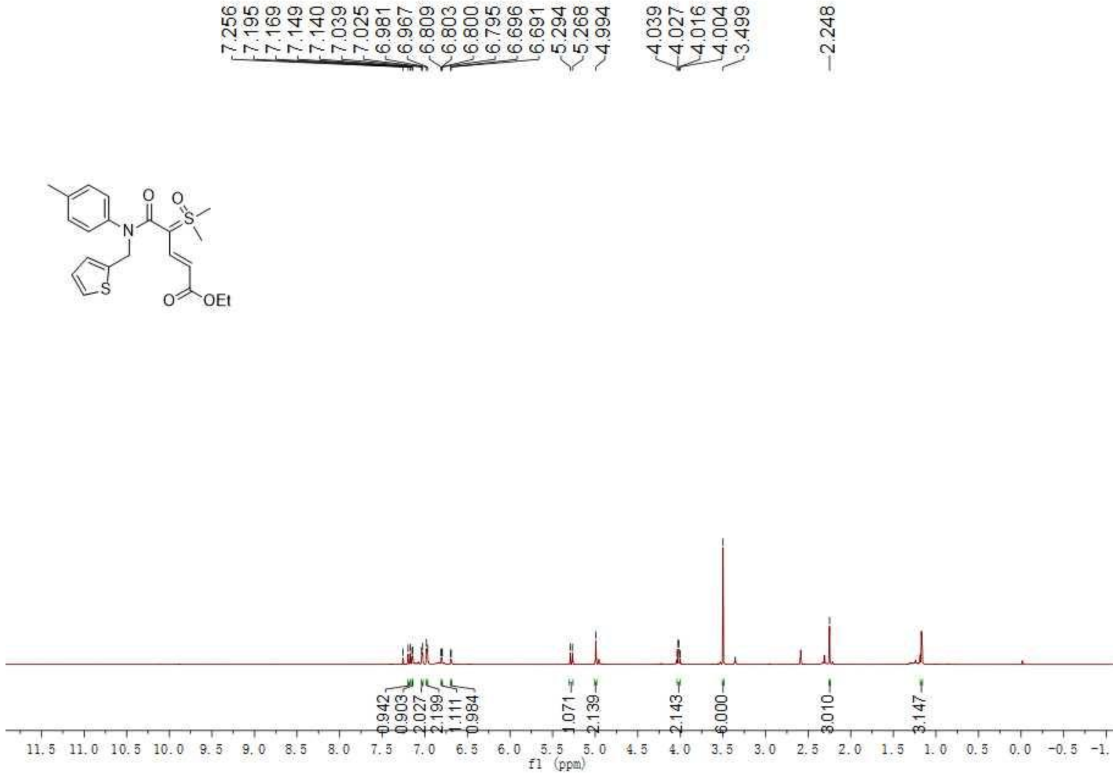

^{13}C NMR (150 MHz, $CDCl_3$) Spectrum of **29**

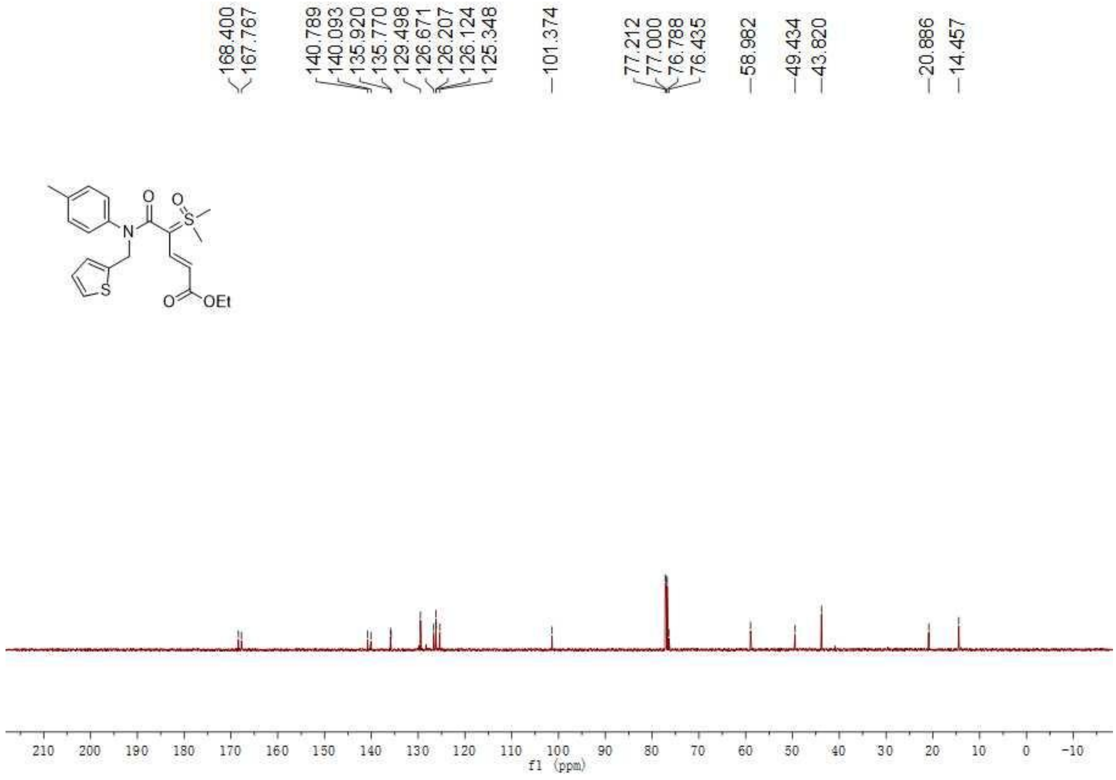

^{1}H NMR (600 MHz, $CDCl_3$) Spectrum of **30**

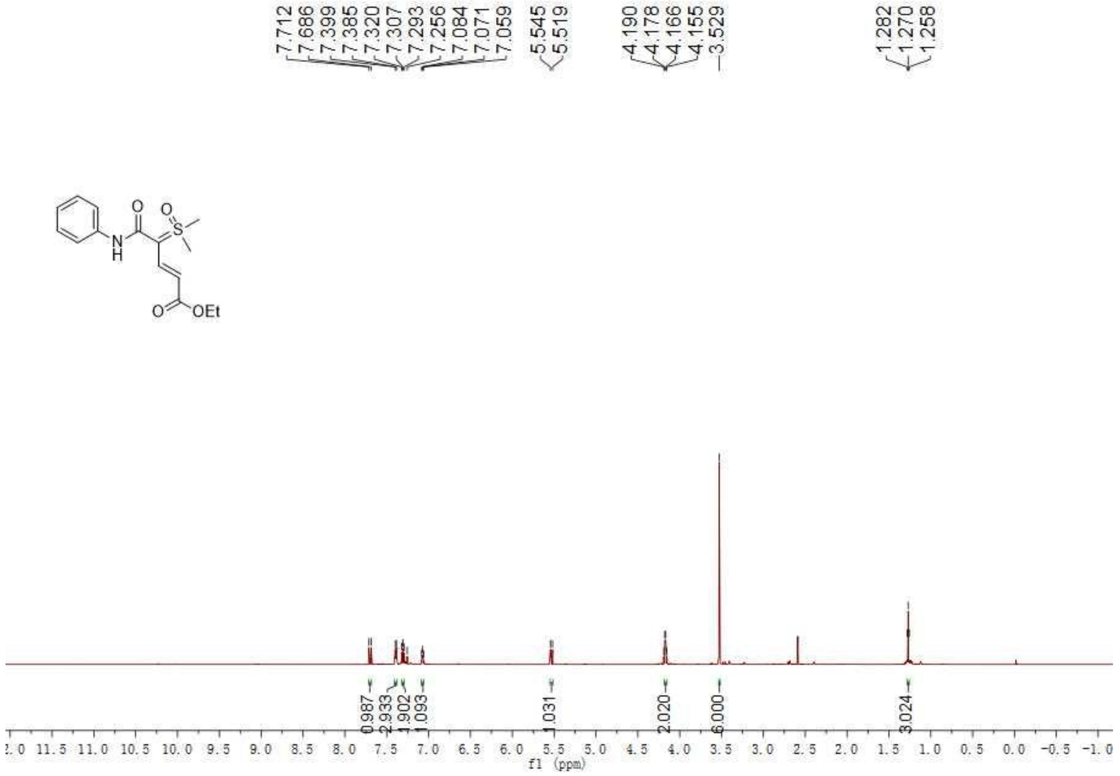

^{13}C NMR (150 MHz, $CDCl_3$) Spectrum of **30**

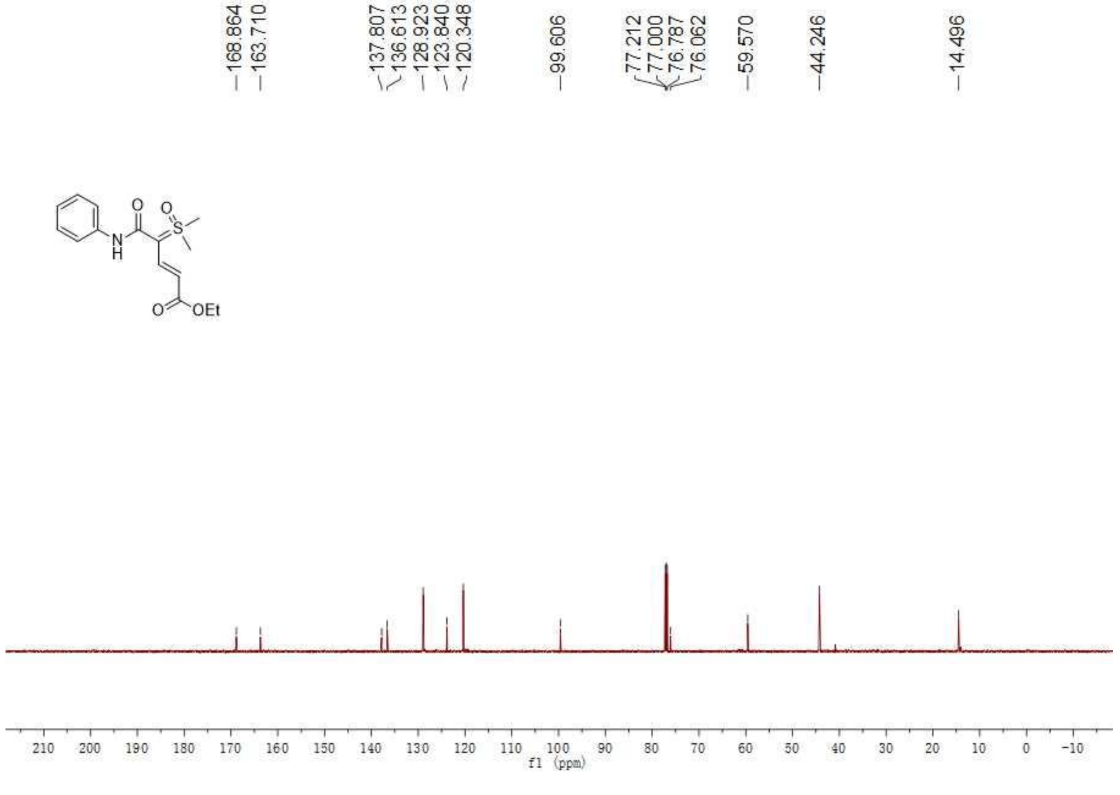

^{1}H NMR (600 MHz, $CDCl_3$) Spectrum of **31**

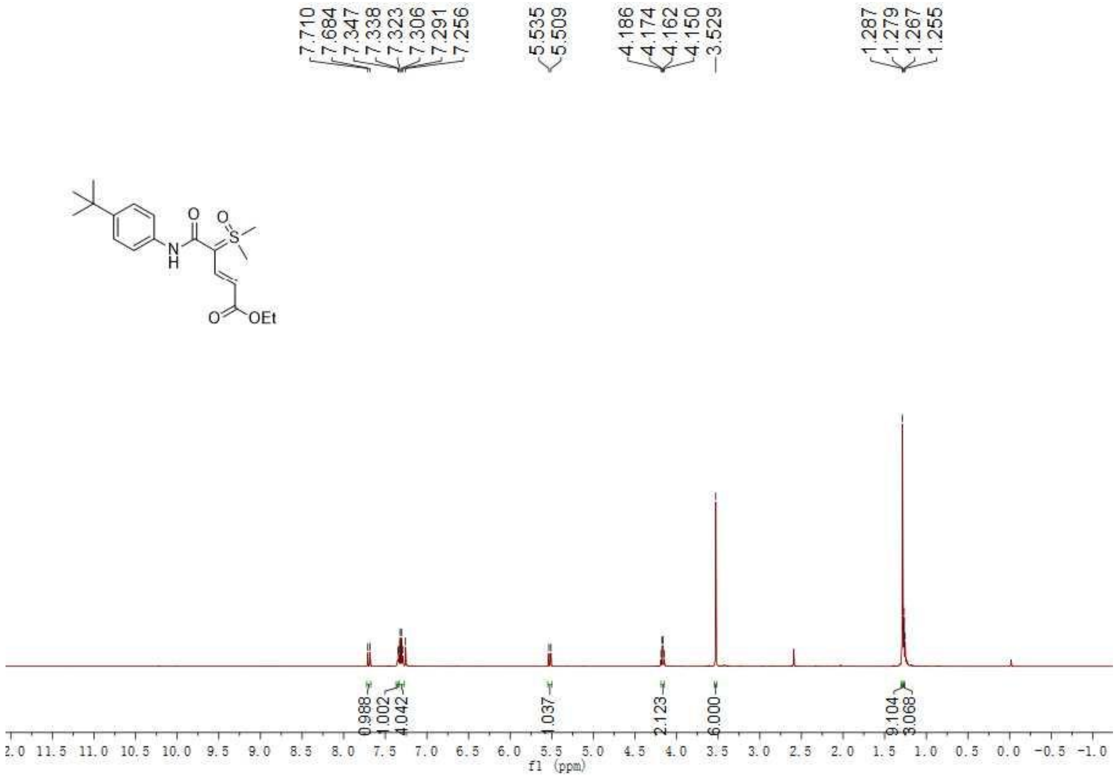

^{13}C NMR (150 MHz, $CDCl_3$) Spectrum of **31**

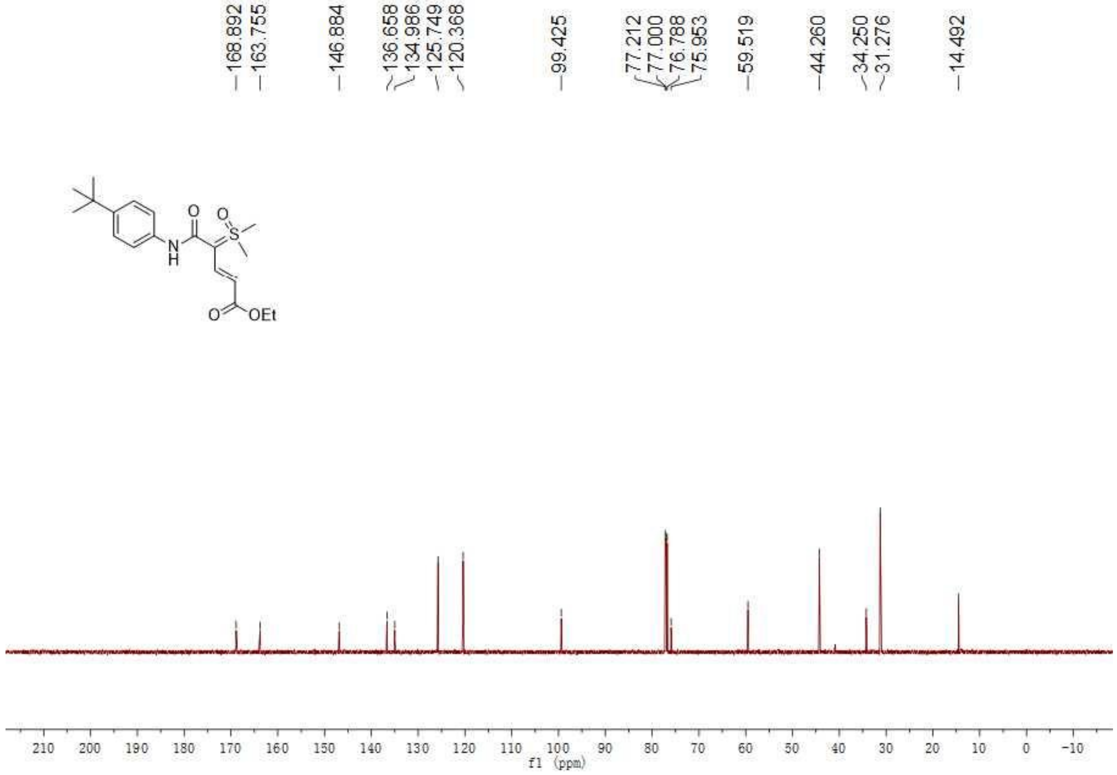

^{1}H NMR (600 MHz, $CDCl_3$) Spectrum of **32**

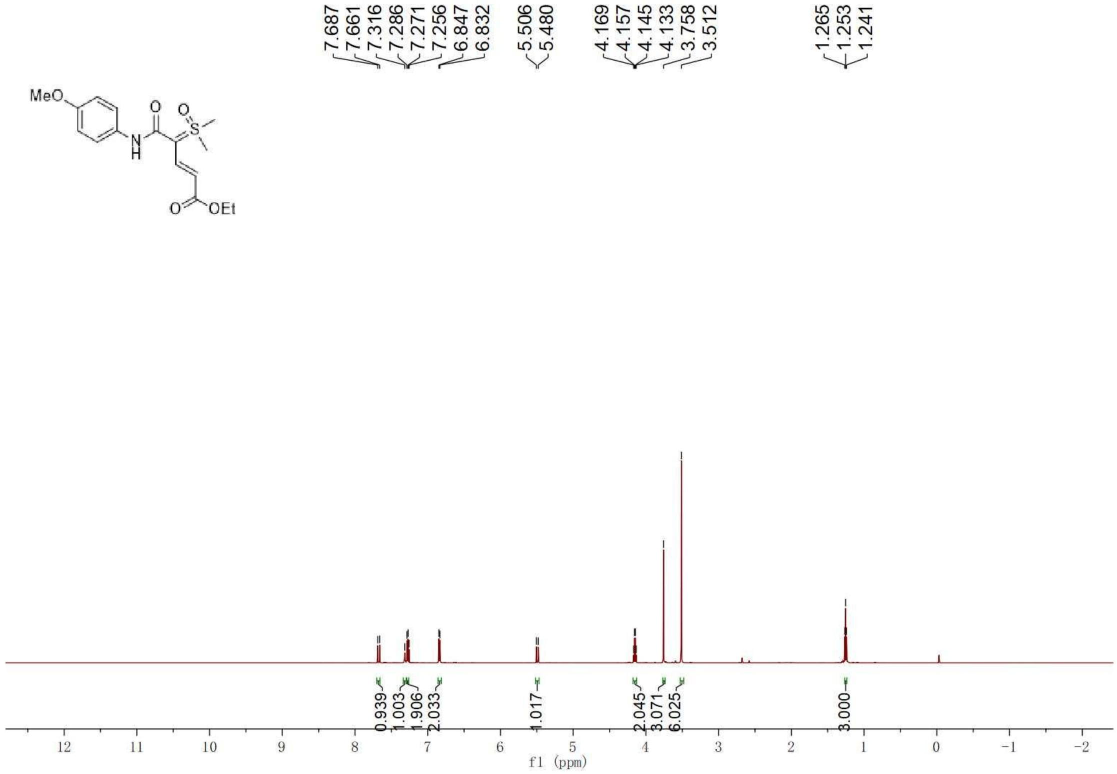

^{13}C NMR (150 MHz, $CDCl_3$) Spectrum of **32**

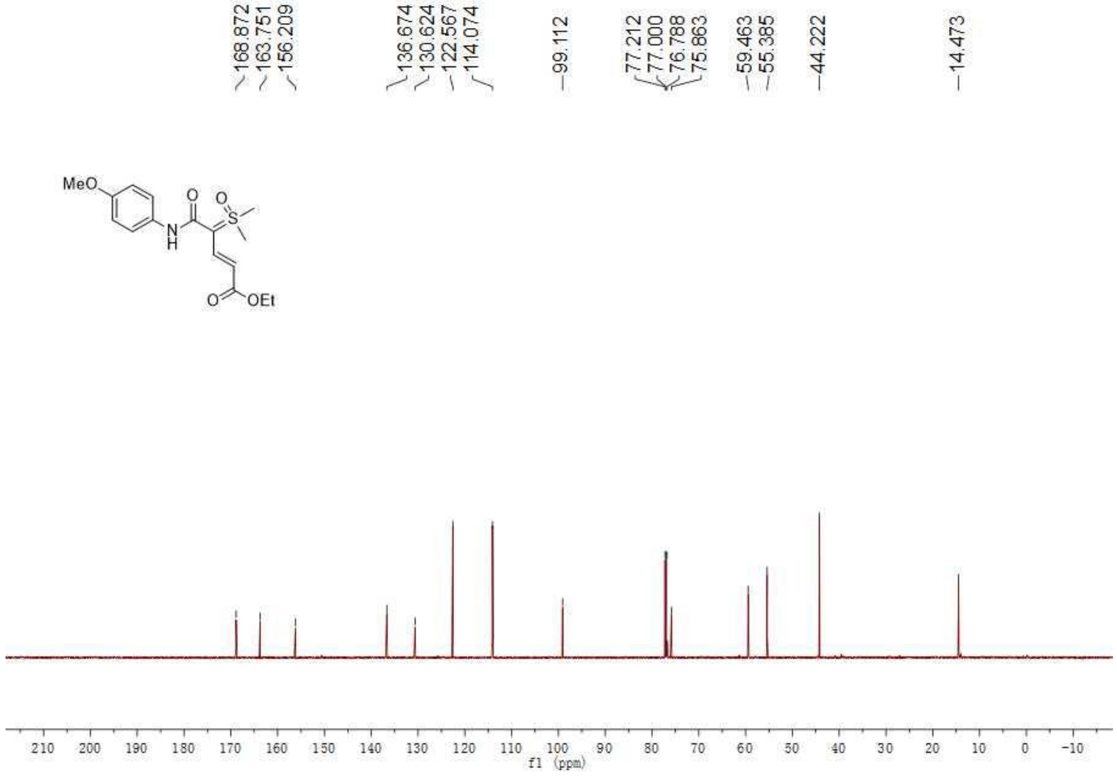

^{1}H NMR (600 MHz, $CDCl_3$) Spectrum of **33**

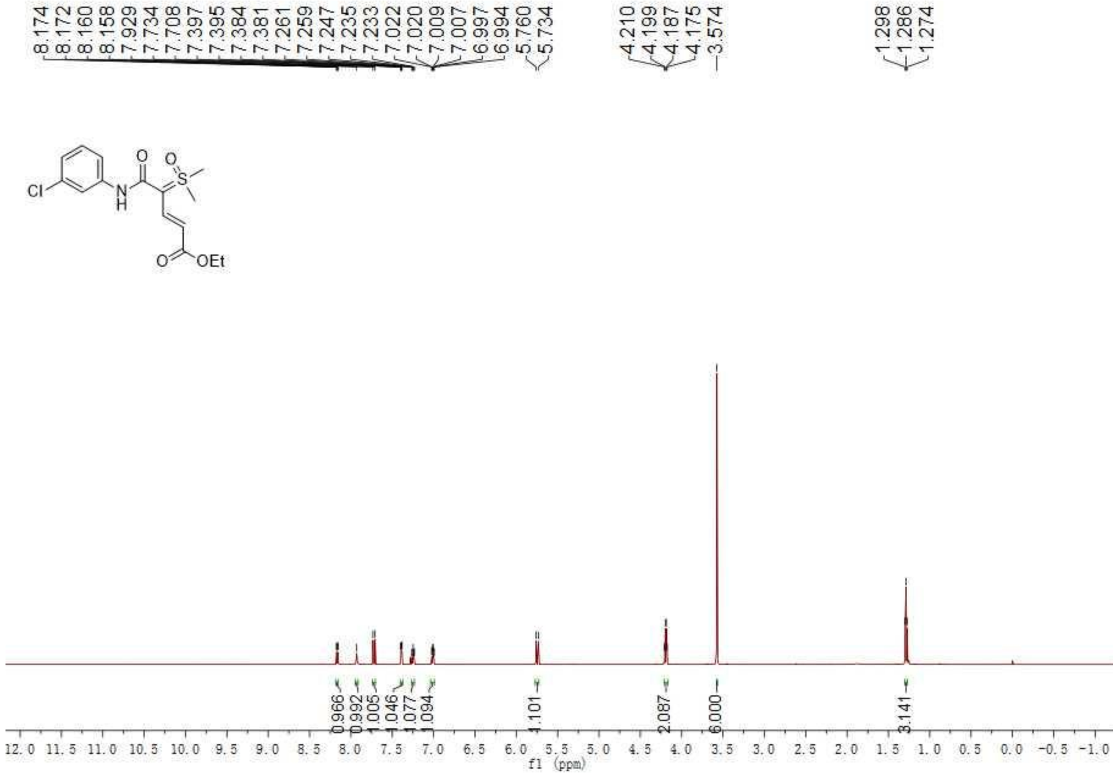

^{13}C NMR (150 MHz, $CDCl_3$) Spectrum of **33**

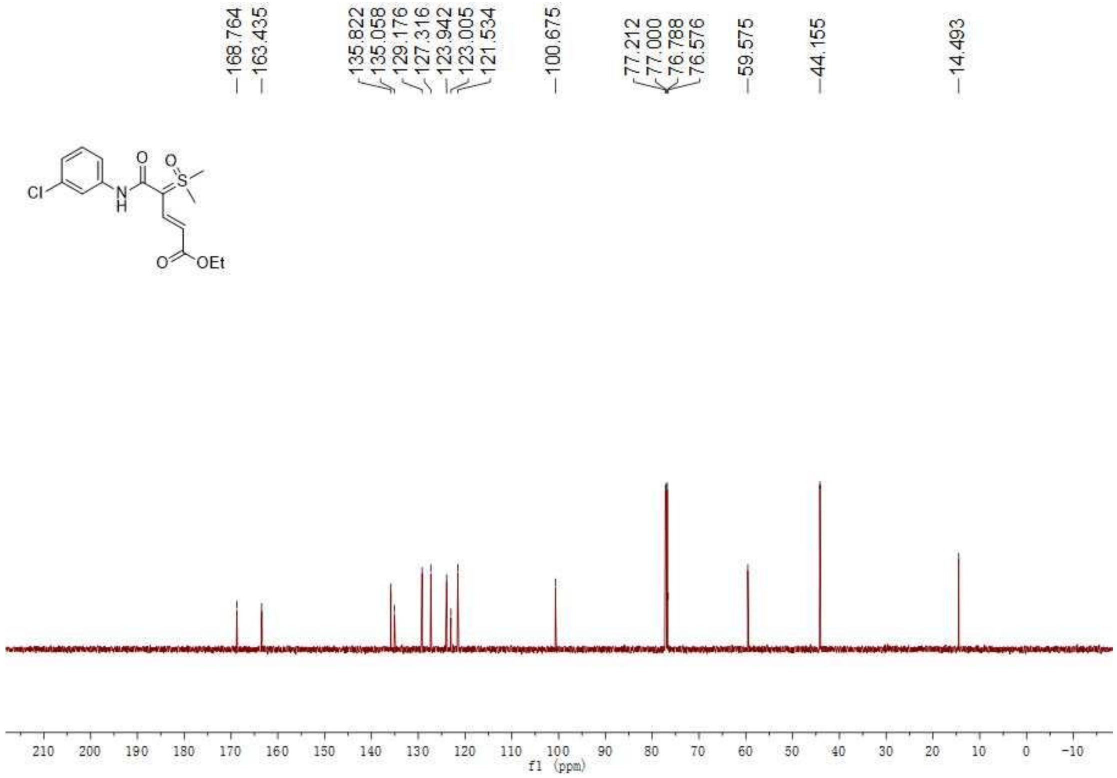

^{1}H NMR (600 MHz, $CDCl_3$) Spectrum of **34**

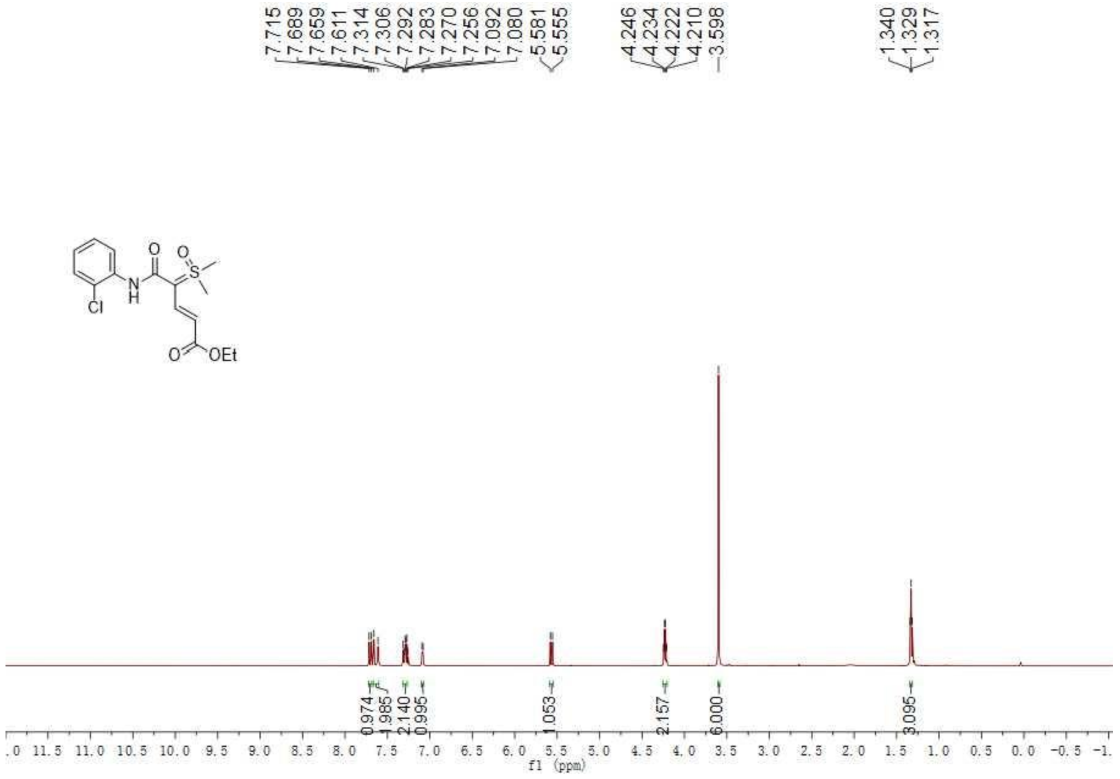

^{13}C NMR (150 MHz, $CDCl_3$) Spectrum of **34**

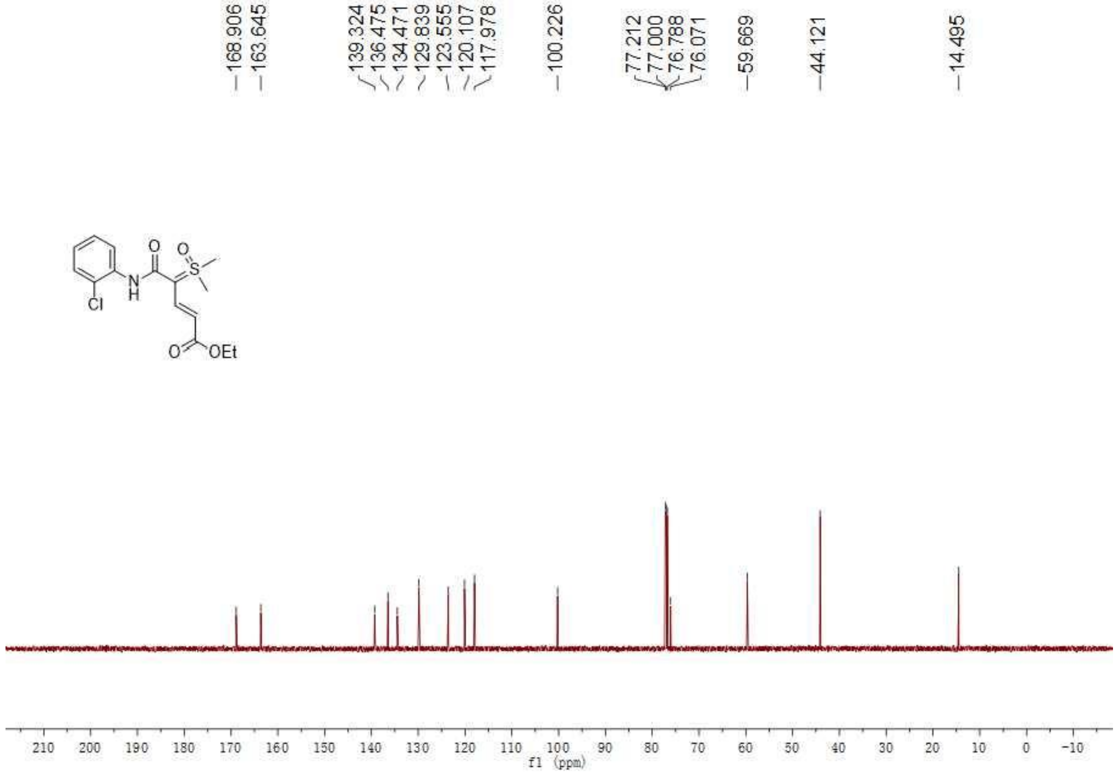

^{1}H NMR (600 MHz, $CDCl_3$) Spectrum of **35**

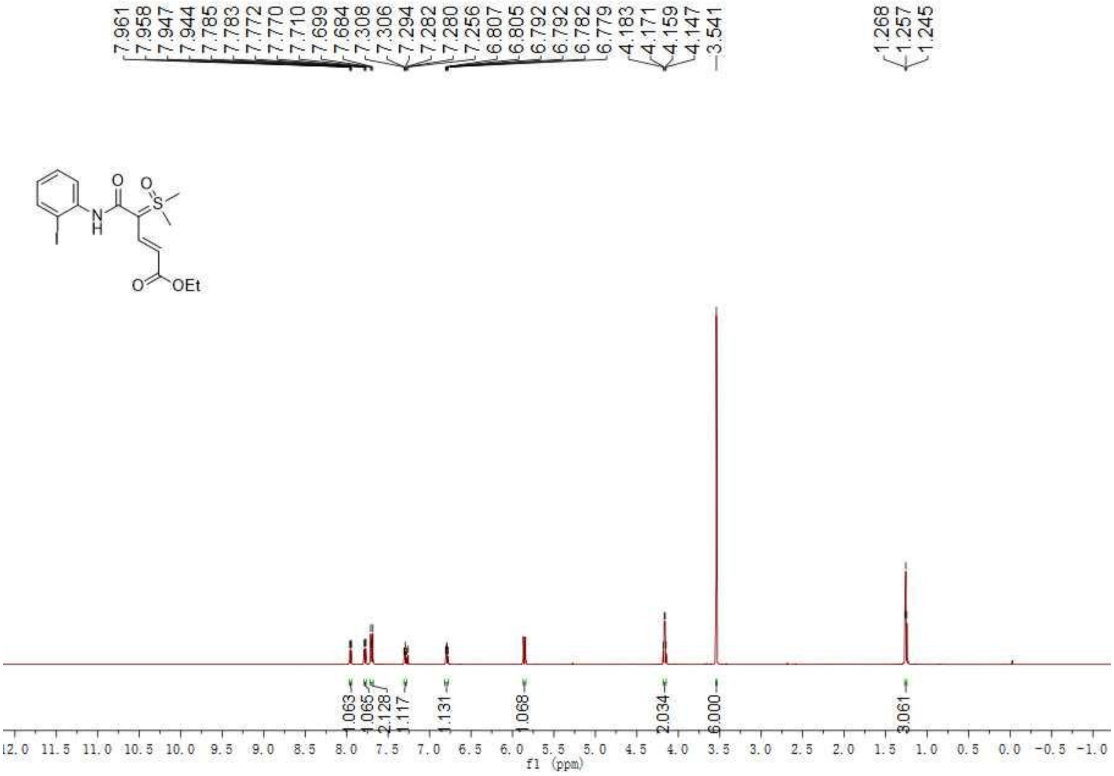

^{13}C NMR (150 MHz, $CDCl_3$) Spectrum of **35**

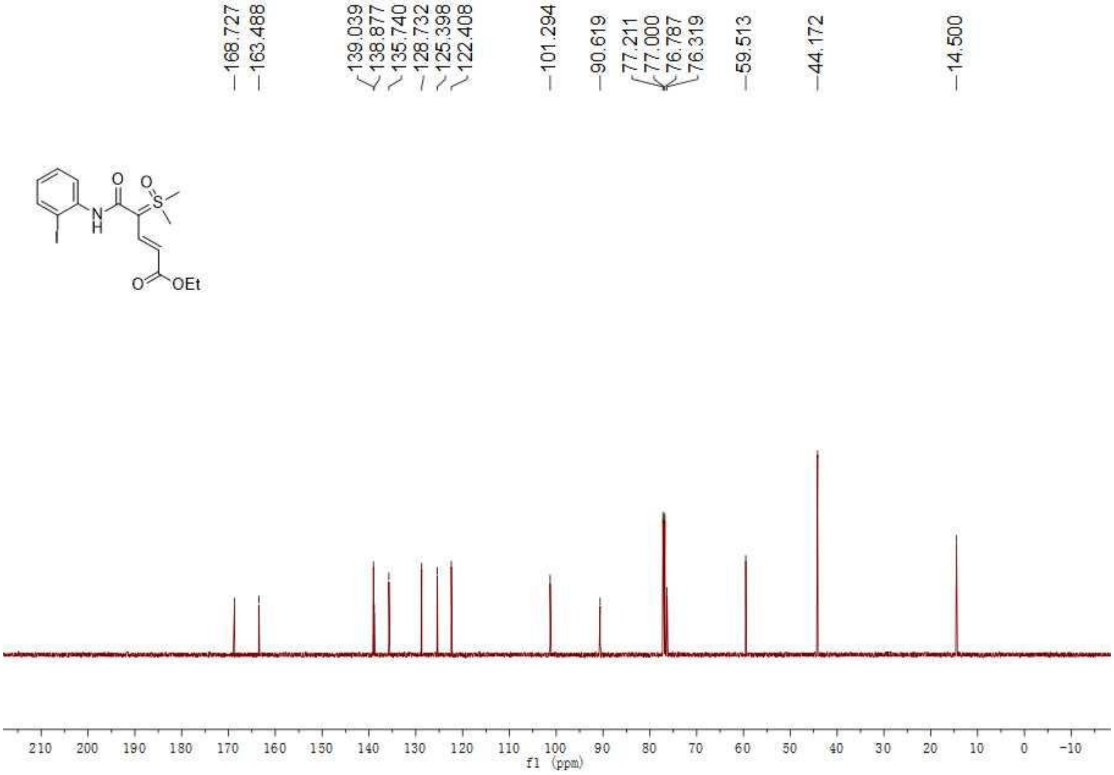

^{1}H NMR (600 MHz, $CDCl_3$) Spectrum of **36**

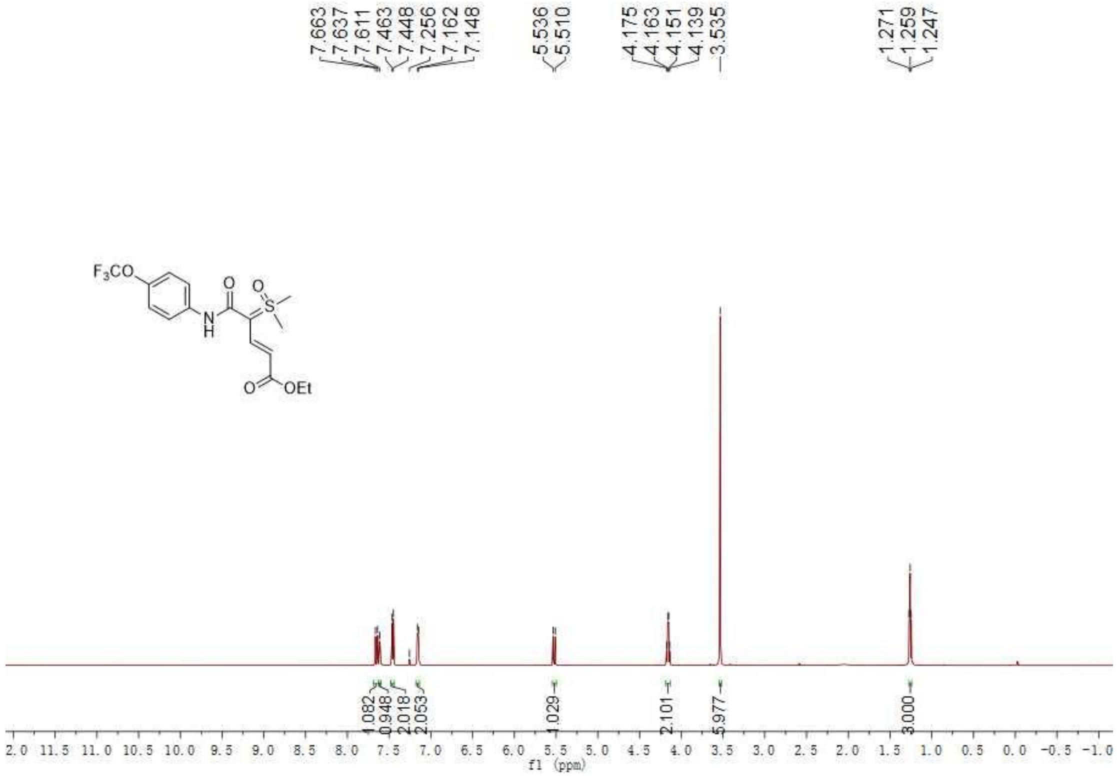

^{13}C NMR (150 MHz, $CDCl_3$) Spectrum of **36**

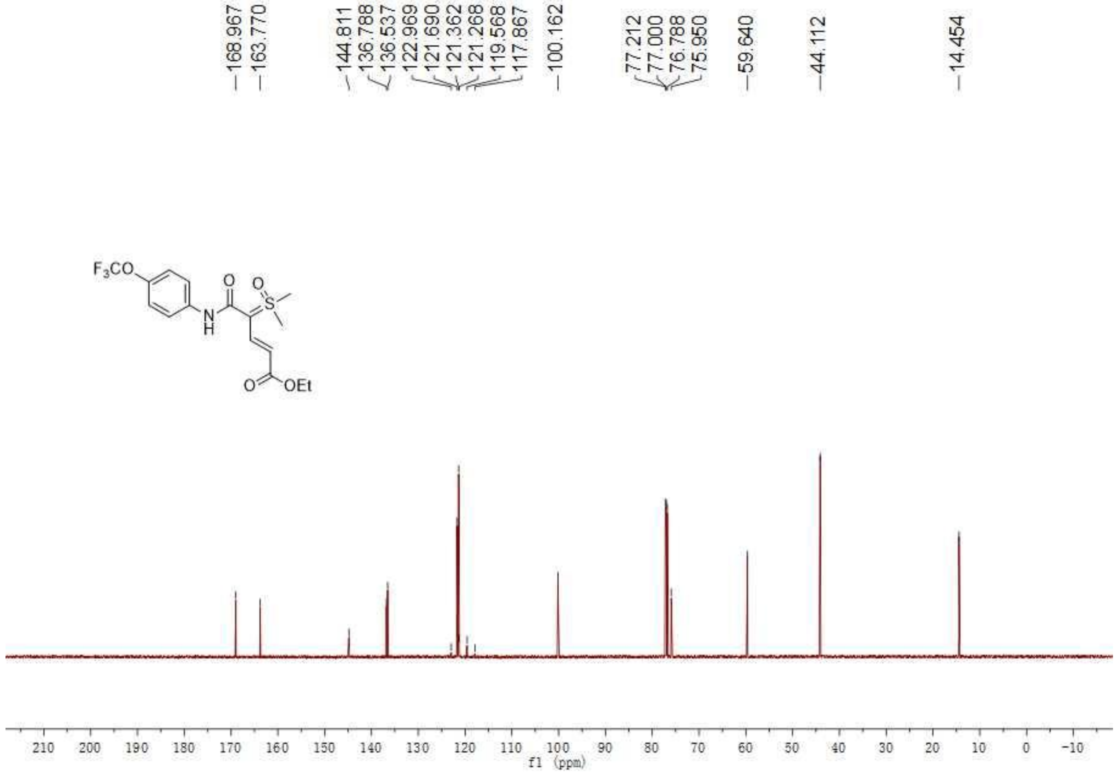

^{19}F NMR (564 MHz, $CDCl_3$) Spectrum of **36**

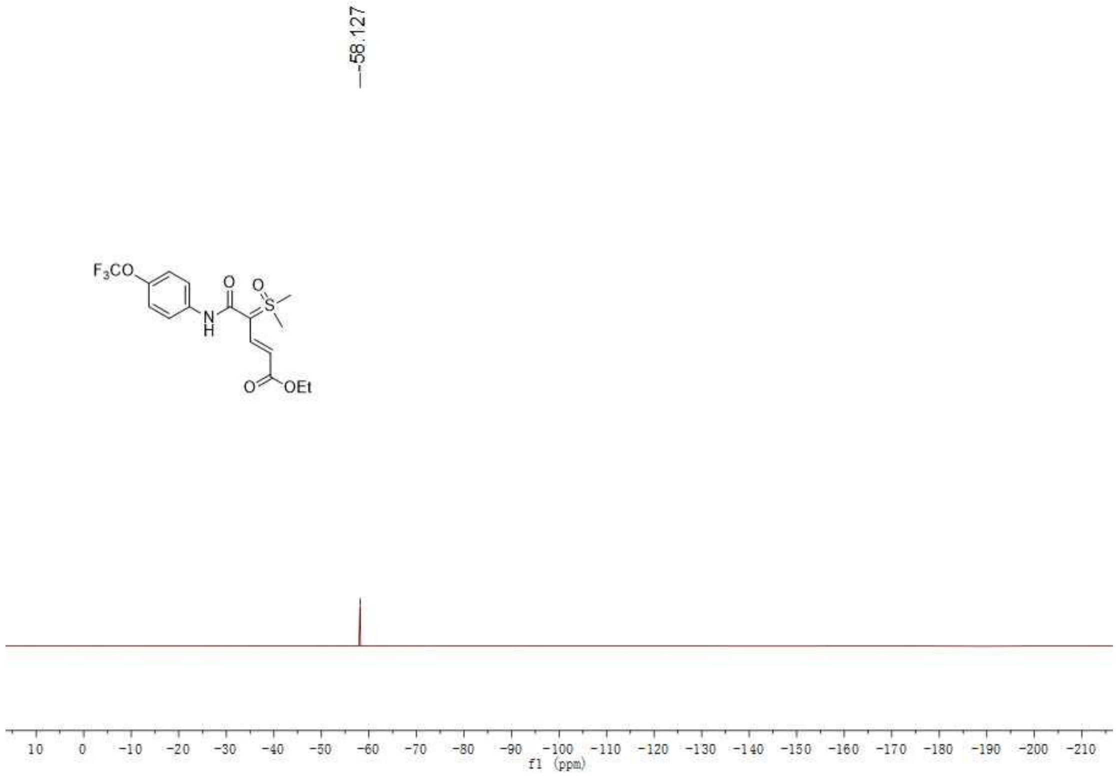

^{1}H NMR (600 MHz, $CDCl_3$) Spectrum of **37**

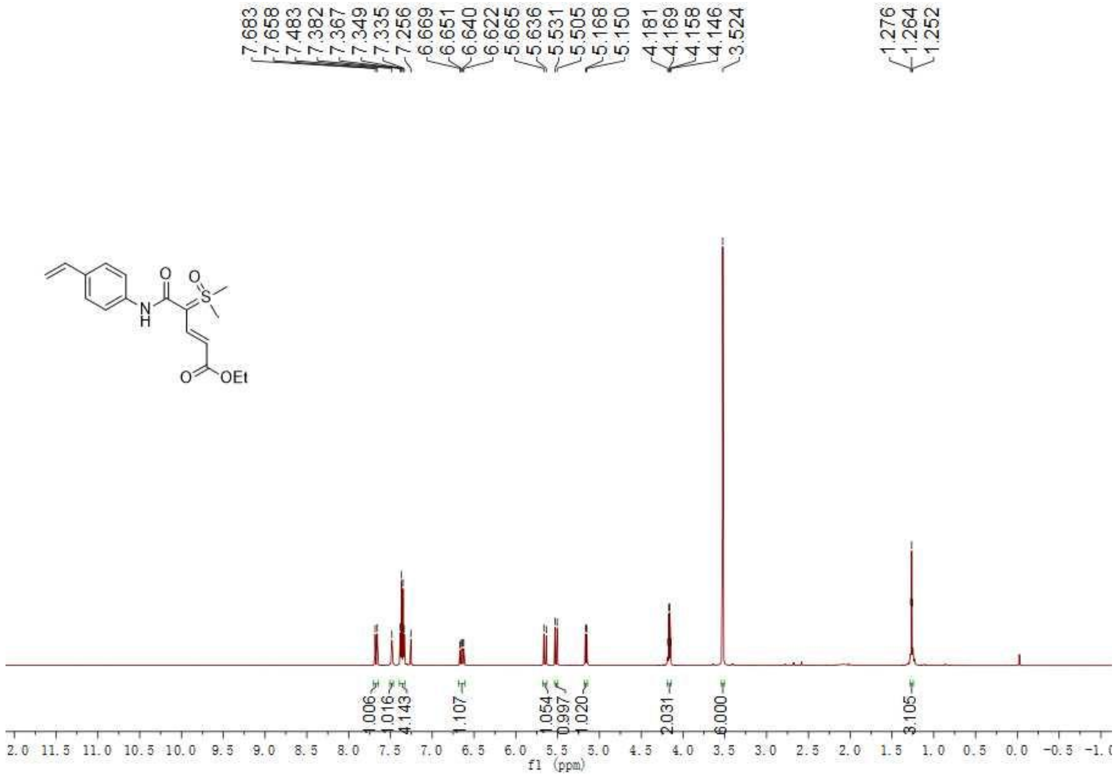

^{13}C NMR (150 MHz, $CDCl_3$) Spectrum of **37**

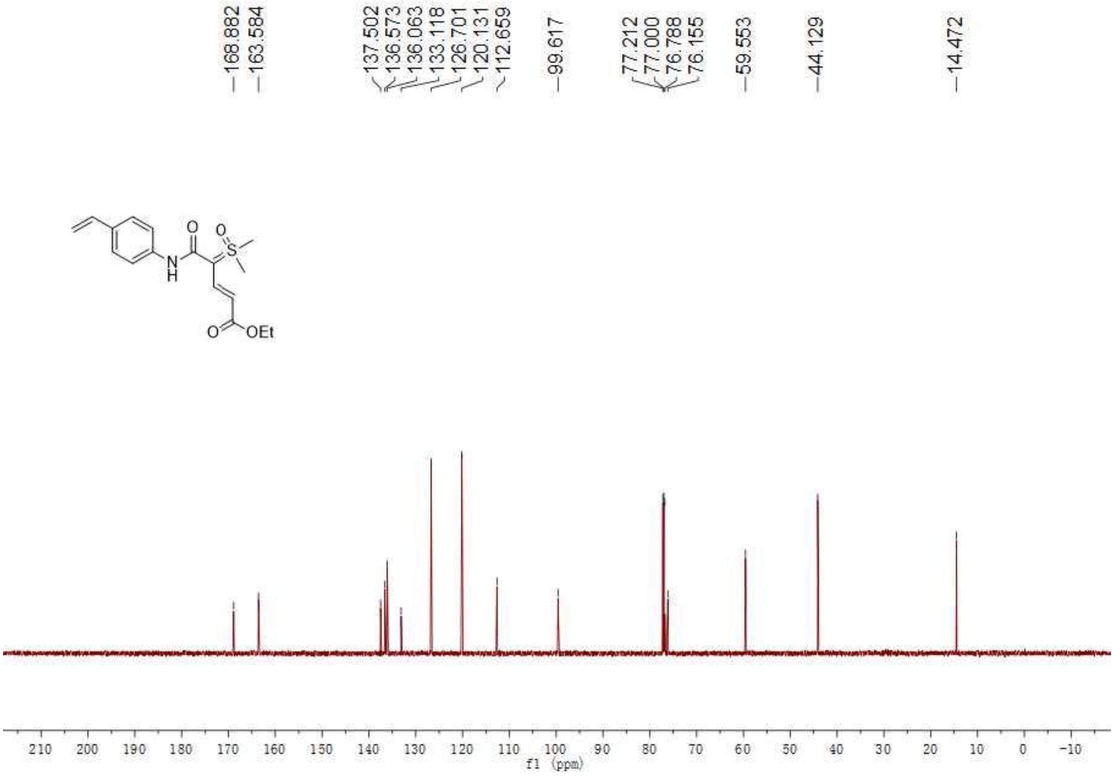

^{1}H NMR (600 MHz, $CDCl_3$) Spectrum of **38**

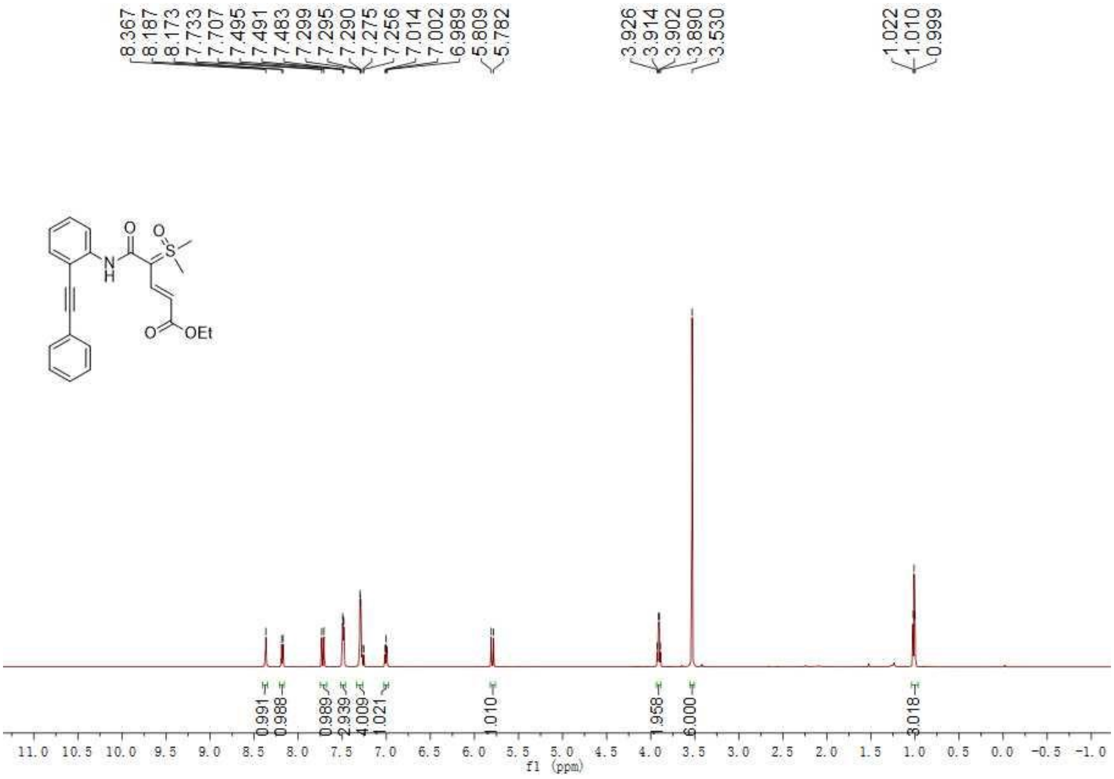

^{13}C NMR (150 MHz, $CDCl_3$) Spectrum of **38**

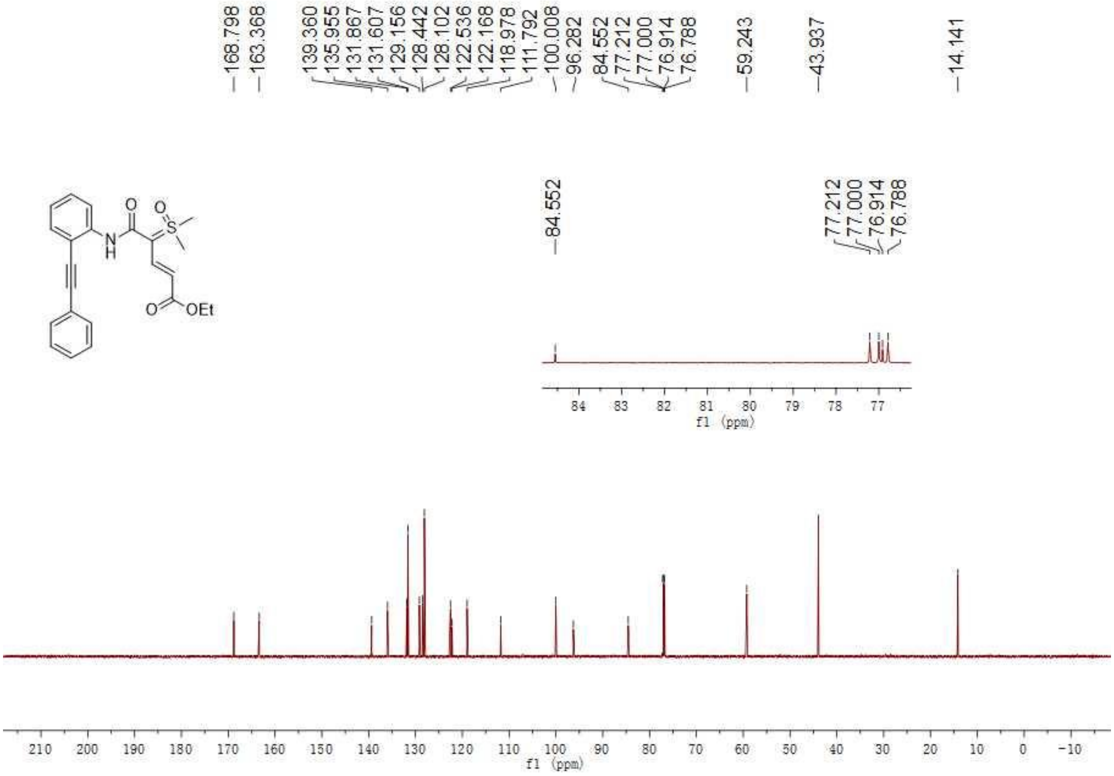

^{1}H NMR (600 MHz, $CDCl_3$) Spectrum of **39**

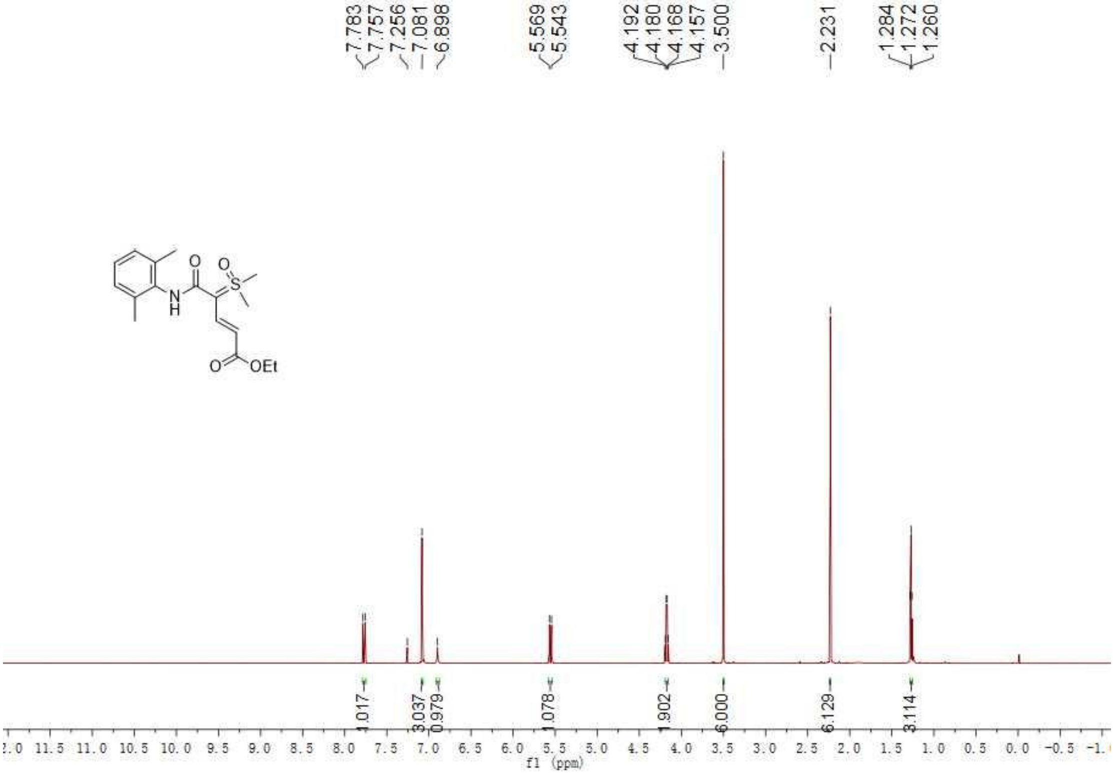

[13] C NMR (150 MHz, $CDCl_3$) Spectrum of **39**

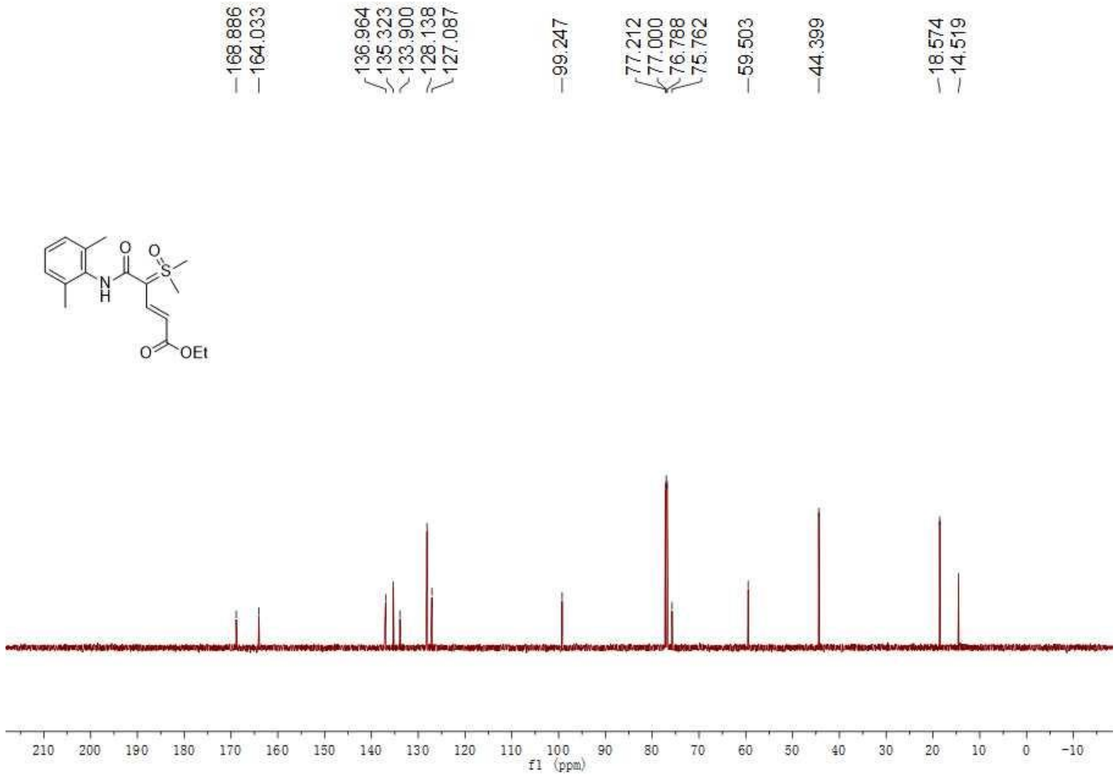

[1] H NMR (600 MHz, $CDCl_3$) Spectrum of **40**

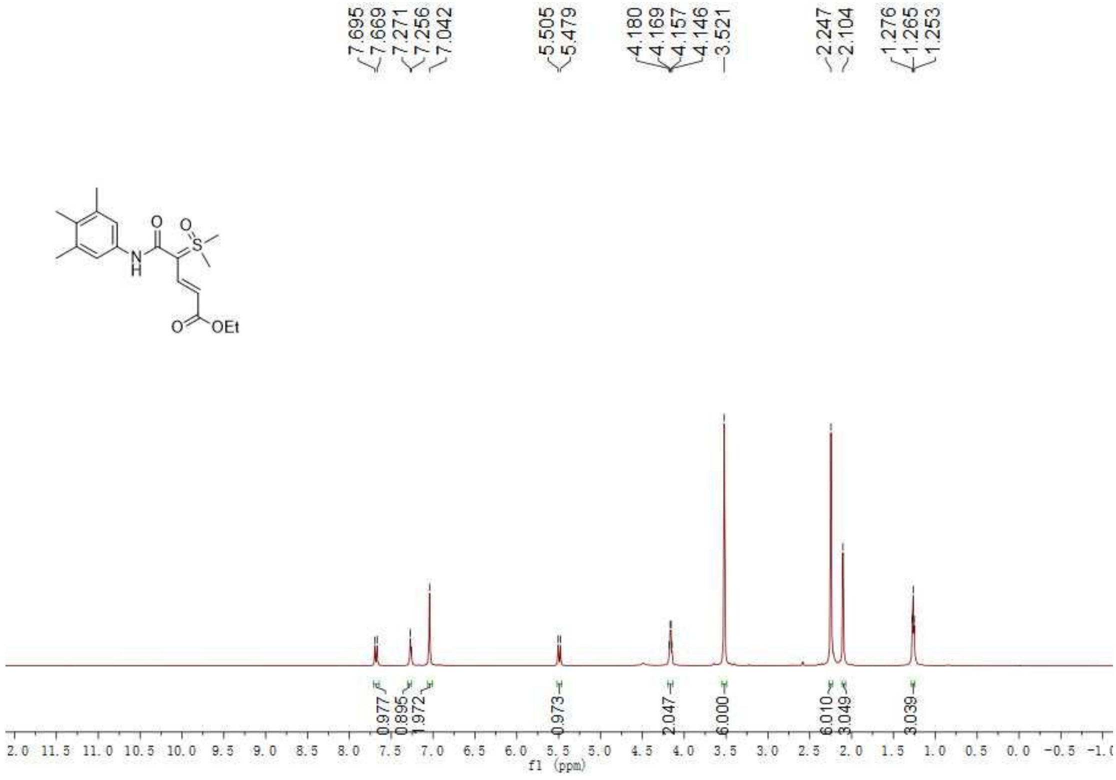

^{13}C NMR (150 MHz, $CDCl_3$) Spectrum of **40**

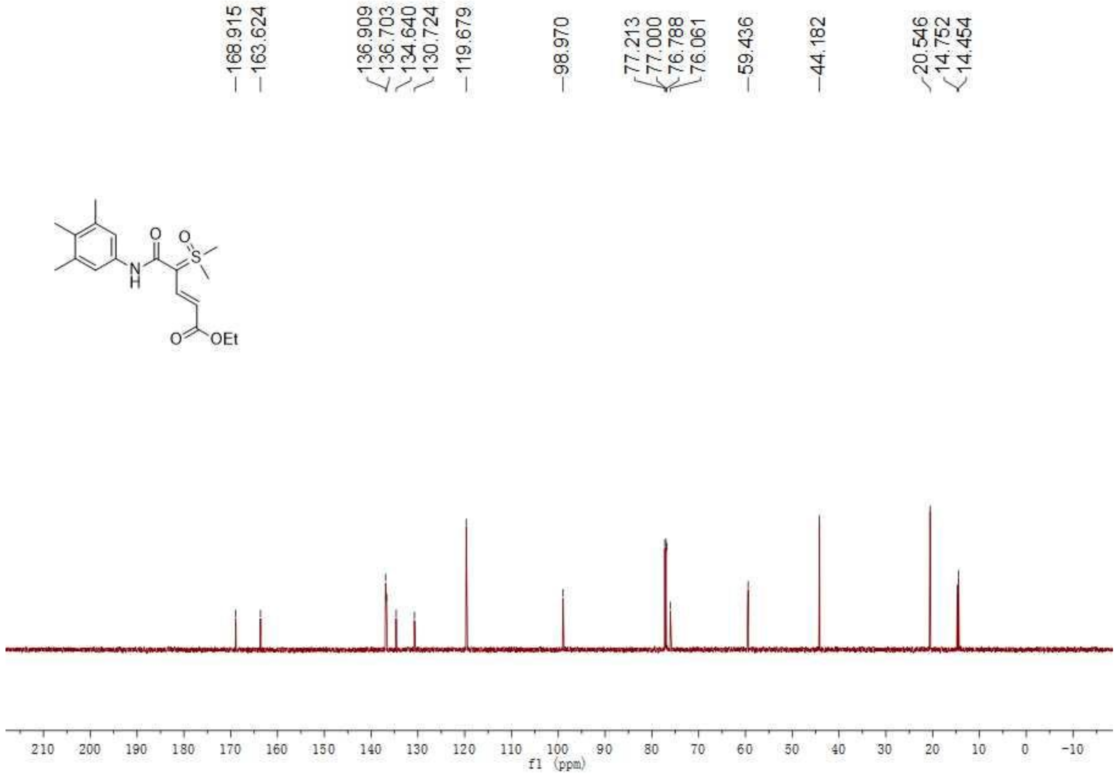

^{1}H NMR (600 MHz, $CDCl_3$) Spectrum of **41**

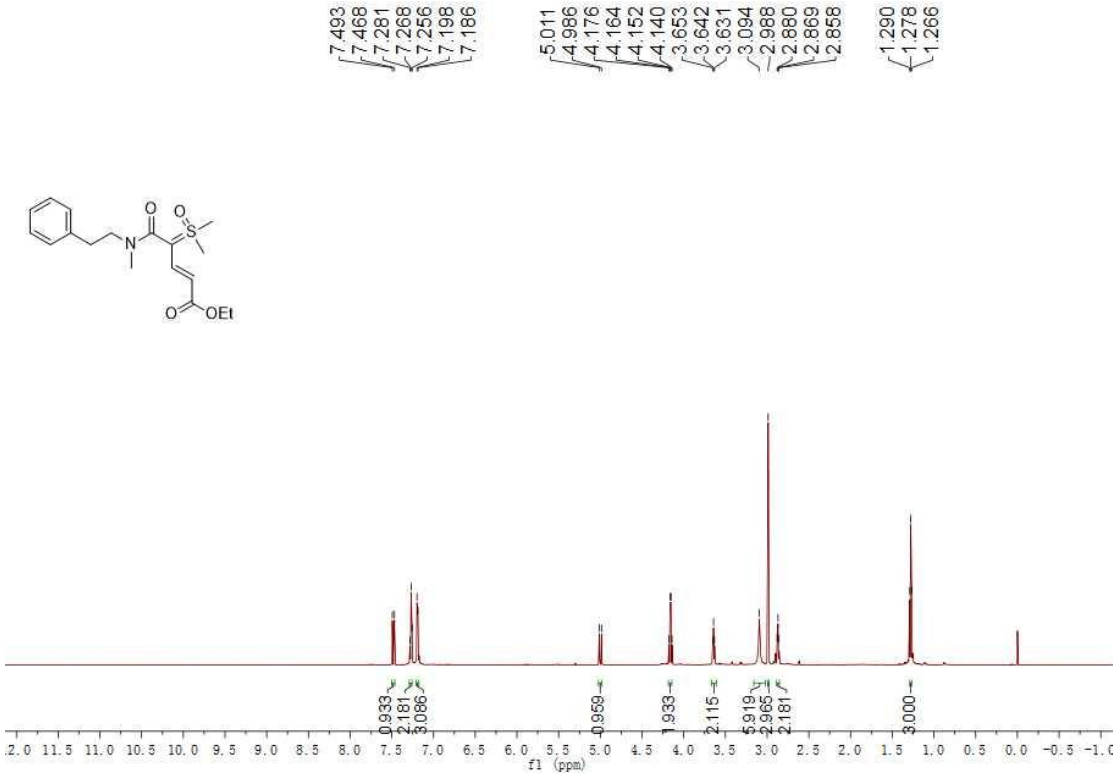

^{13}C NMR (150 MHz, $CDCl_3$) Spectrum of **41**

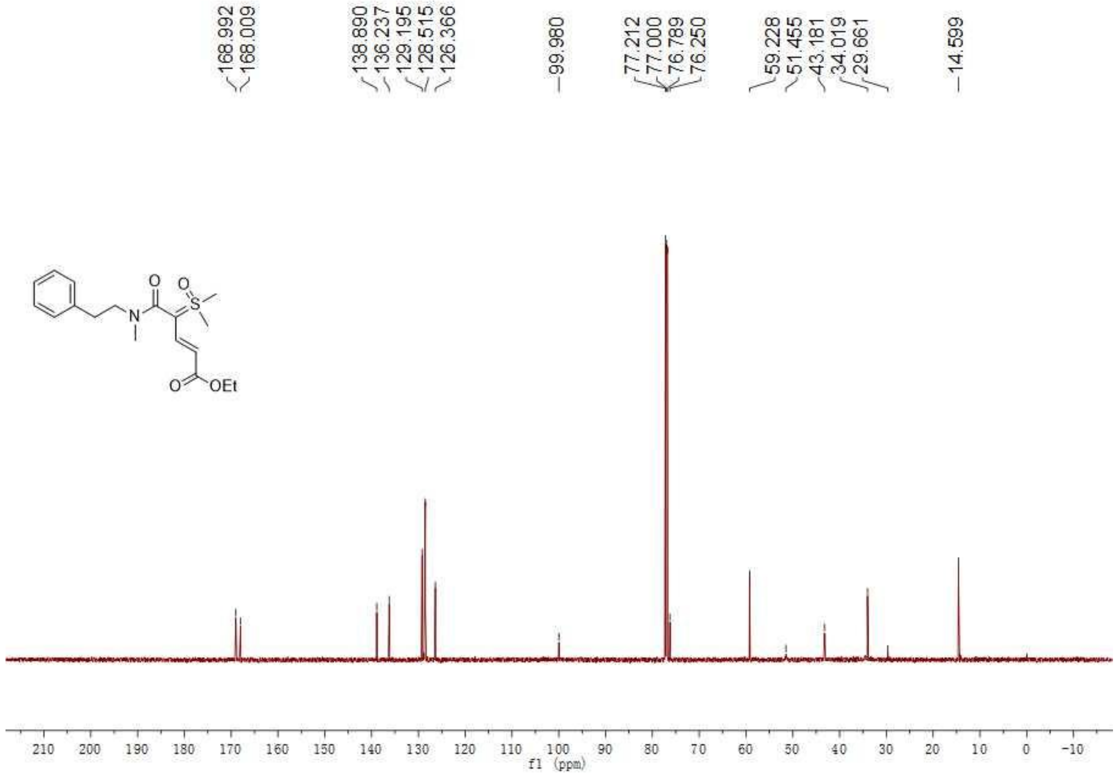

^{1}H NMR (600 MHz, $CDCl_3$) Spectrum of **42**

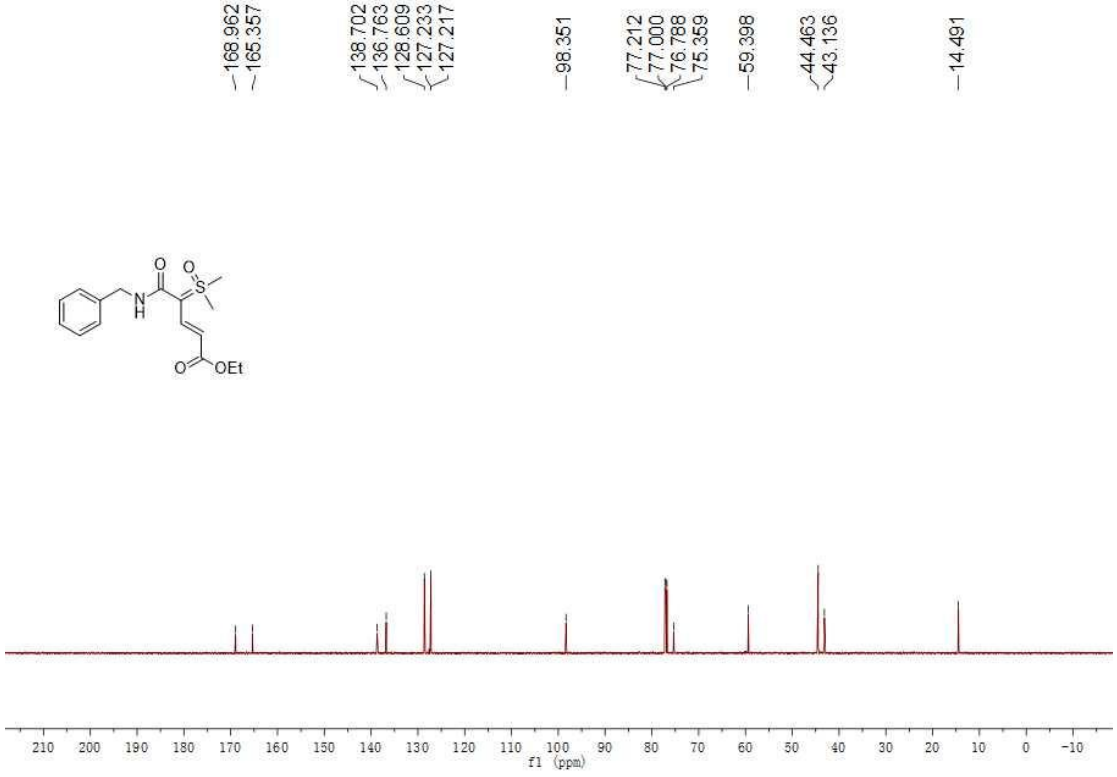

^{13}C NMR (150 MHz, $CDCl_3$) Spectrum of **42**

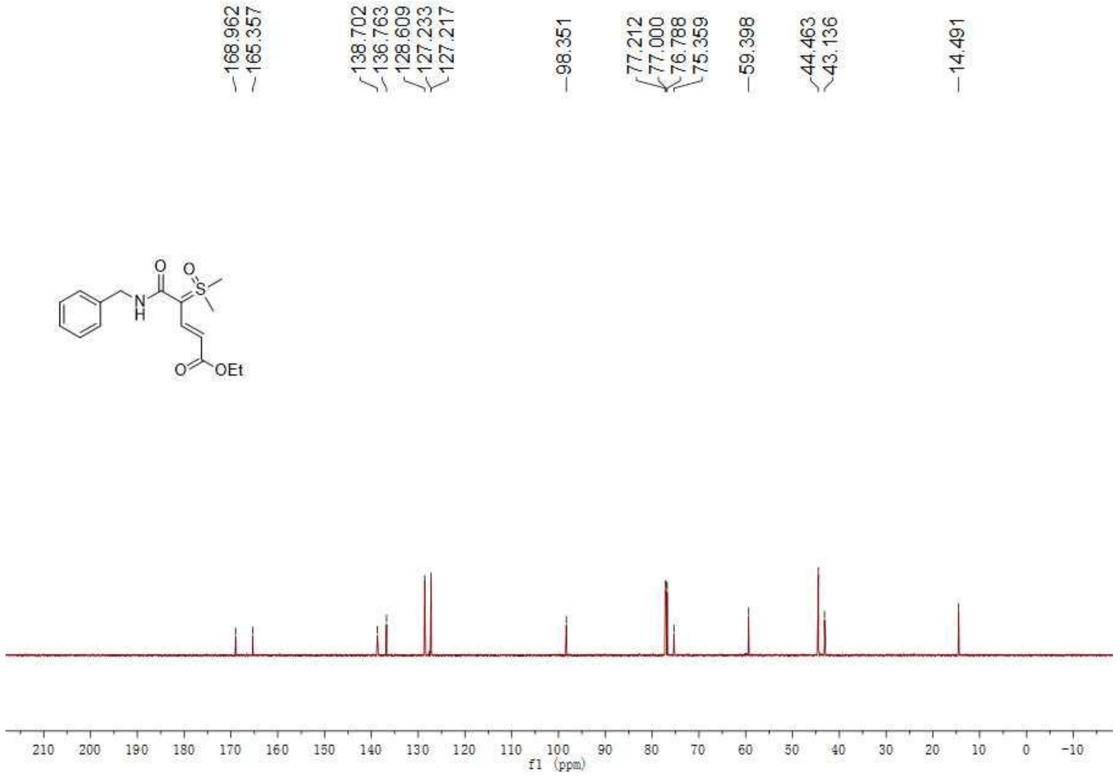

^{1}H NMR (600 MHz, $CDCl_3$) Spectrum of **43**

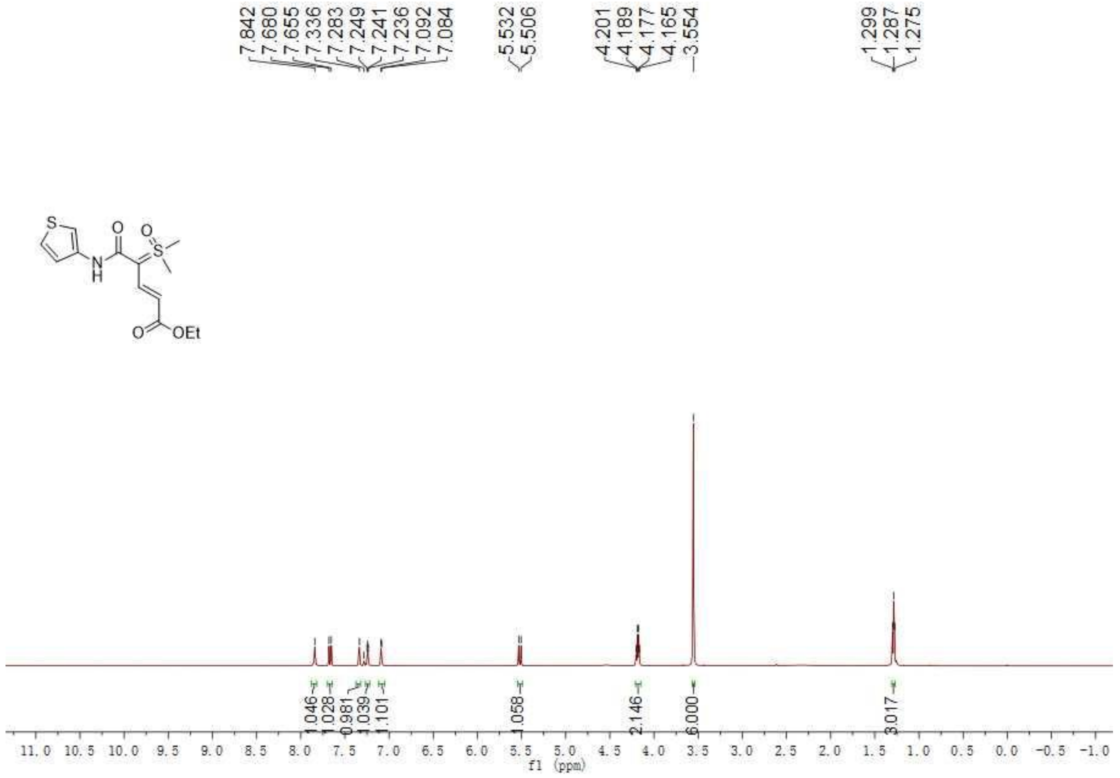

^{13}C NMR (150 MHz, $CDCl_3$) Spectrum of **43**

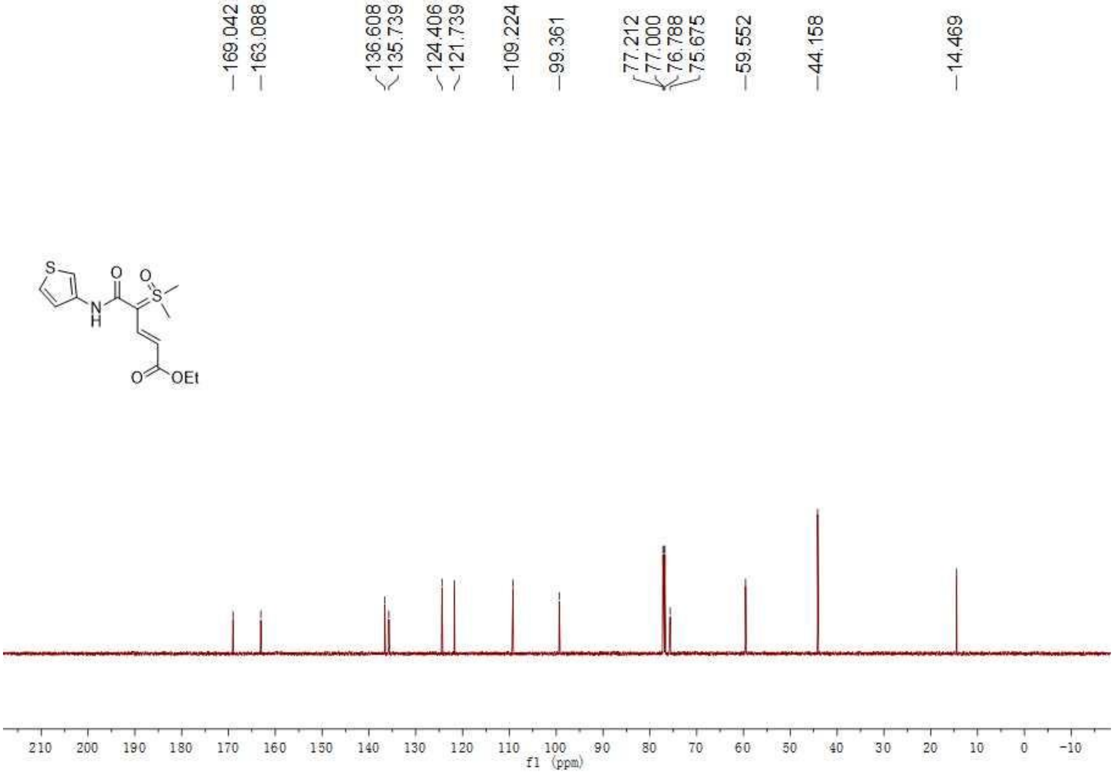

^{1}H NMR (600 MHz, $CDCl_3$) Spectrum of **44**

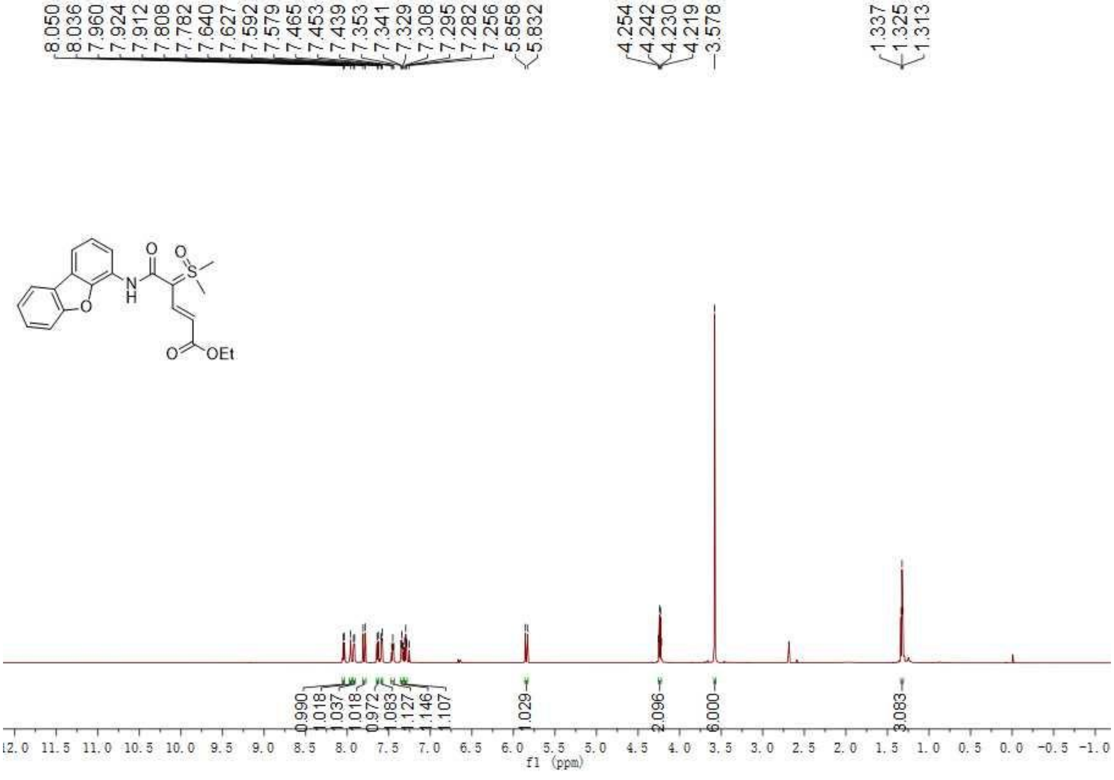

^{13}C NMR (150 MHz, $CDCl_3$) Spectrum of **44**

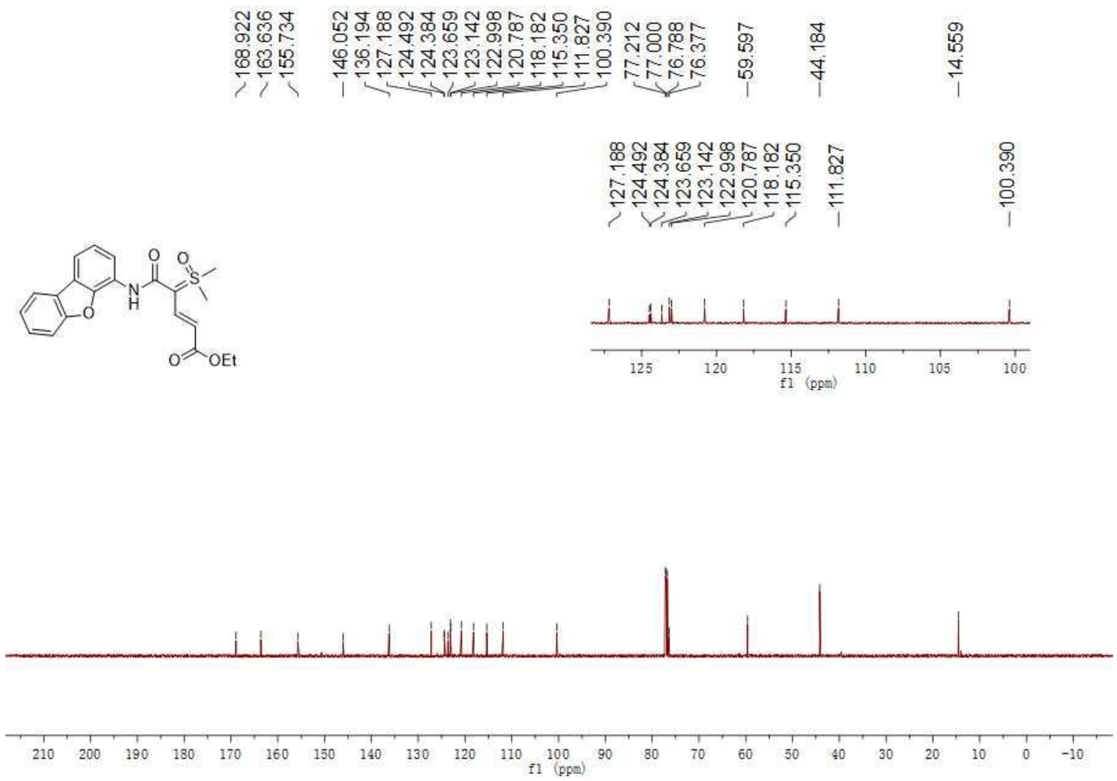

^{1}H NMR (600 MHz, $CDCl_3$) Spectrum of **45**

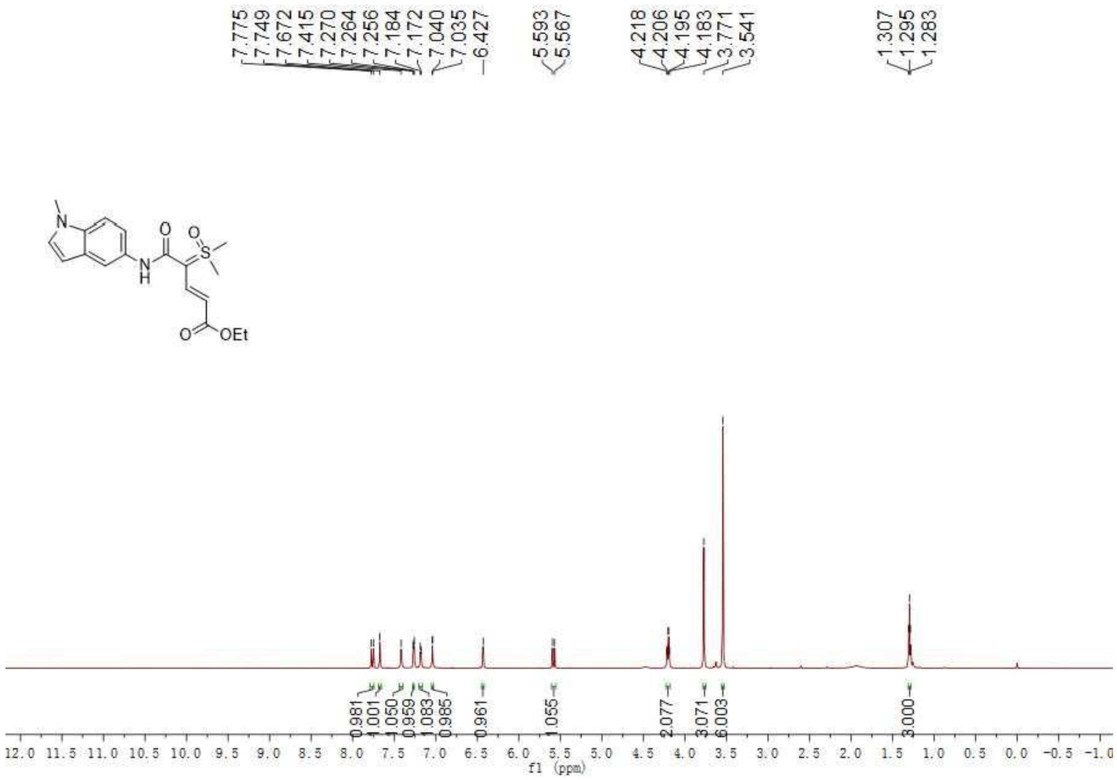

^{13}C NMR (150 MHz, $CDCl_3$) Spectrum of **45**

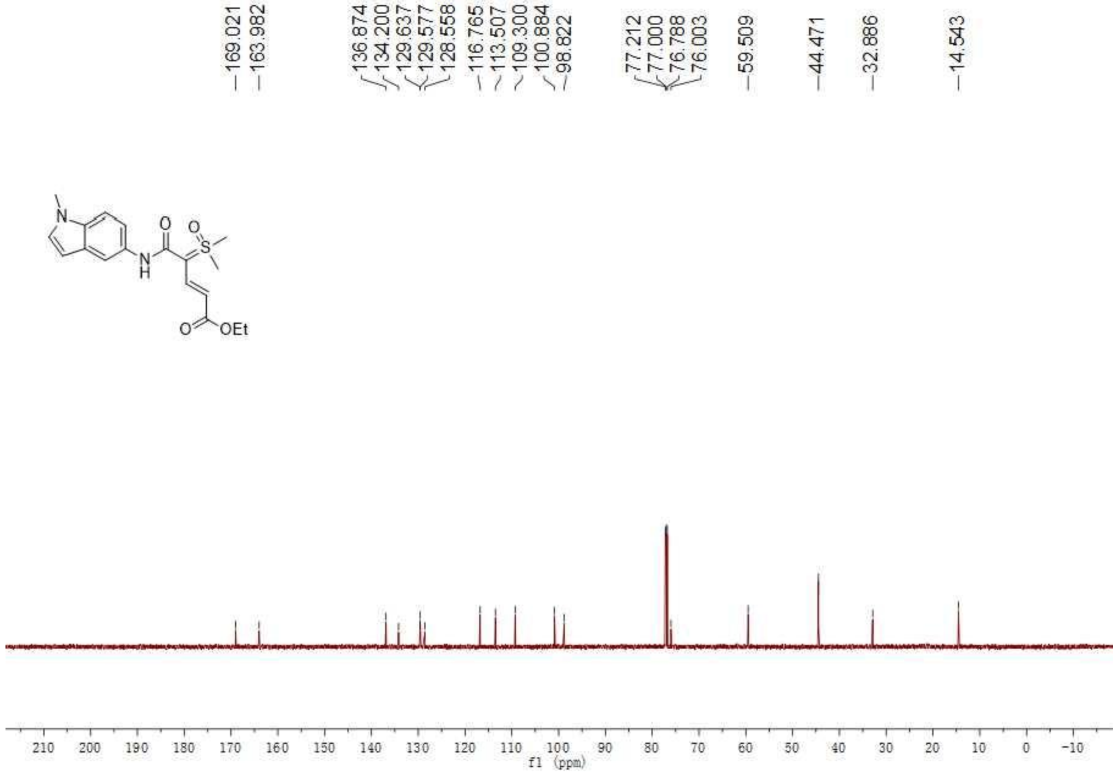

^{1}H NMR (600 MHz, $CDCl_3$) Spectrum of **46**

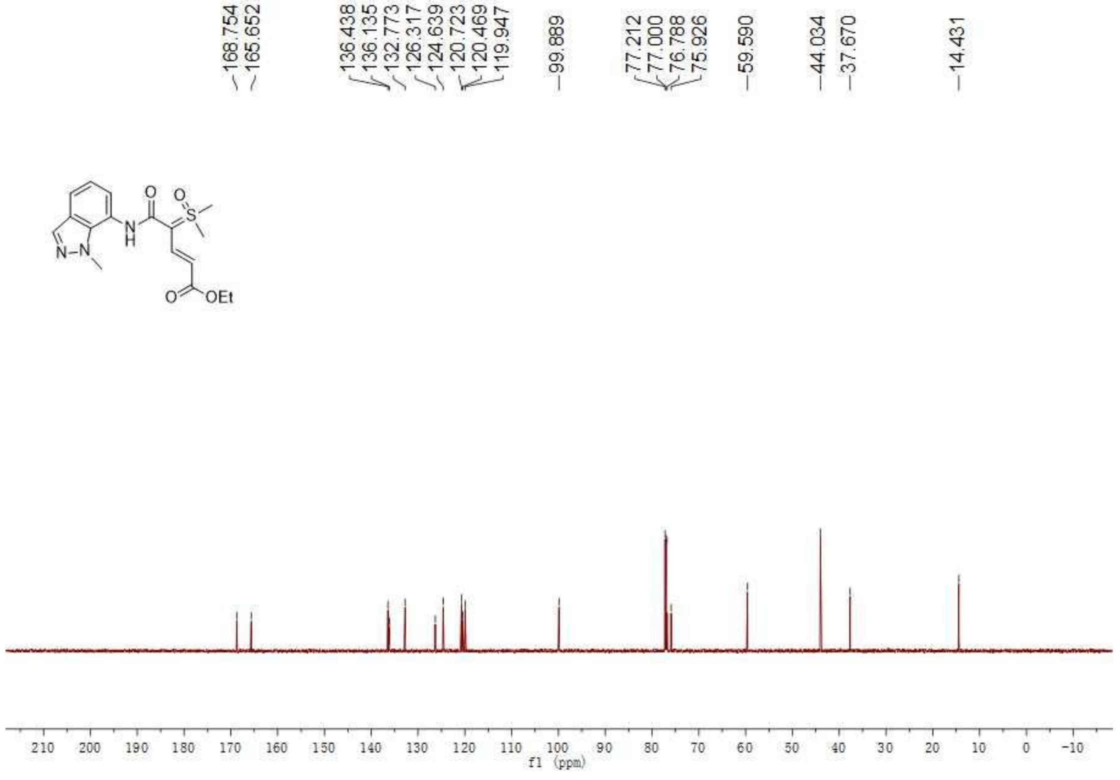

^{13}C NMR (150 MHz, $CDCl_3$) Spectrum of **46**

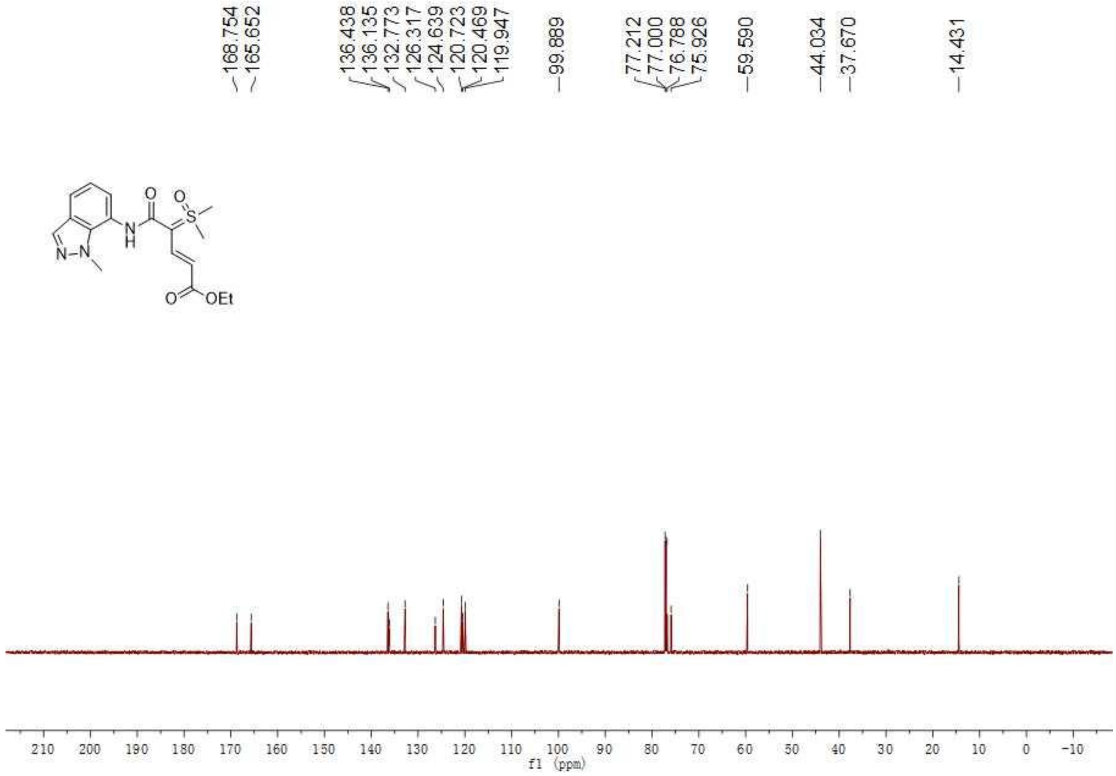

^{1}H NMR (600 MHz, $CDCl_3$) Spectrum of **47**

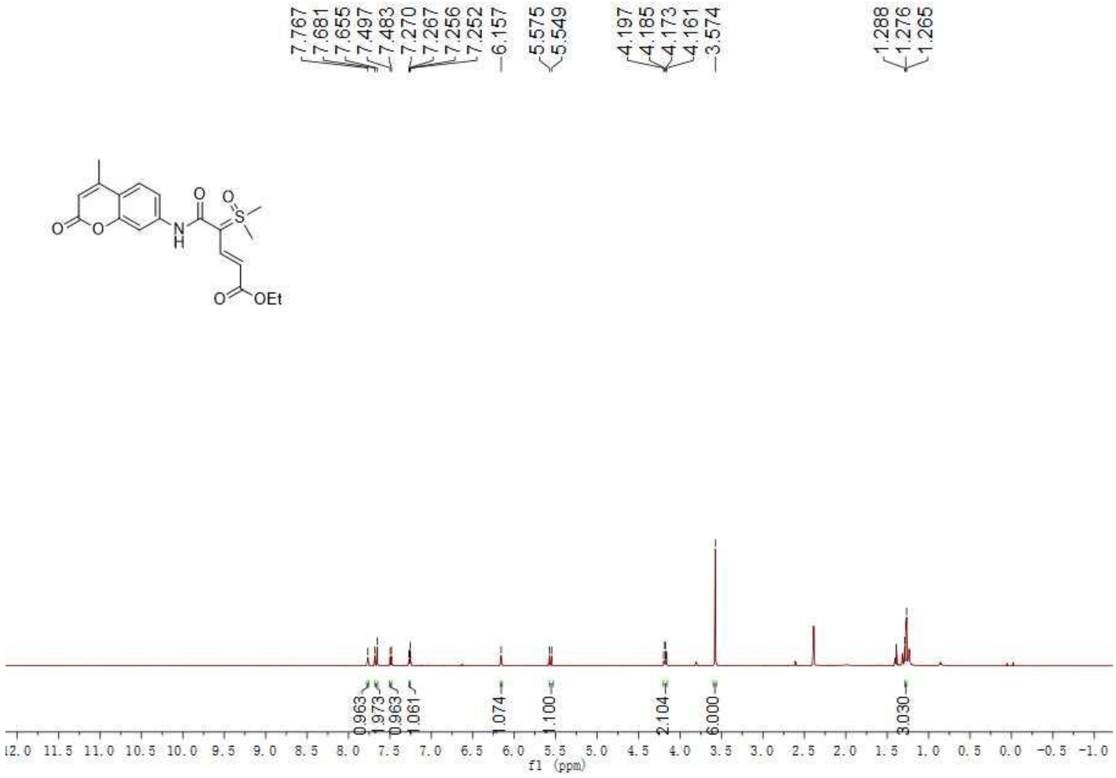

^{13}C NMR (150 MHz, $CDCl_3$) Spectrum of **47**

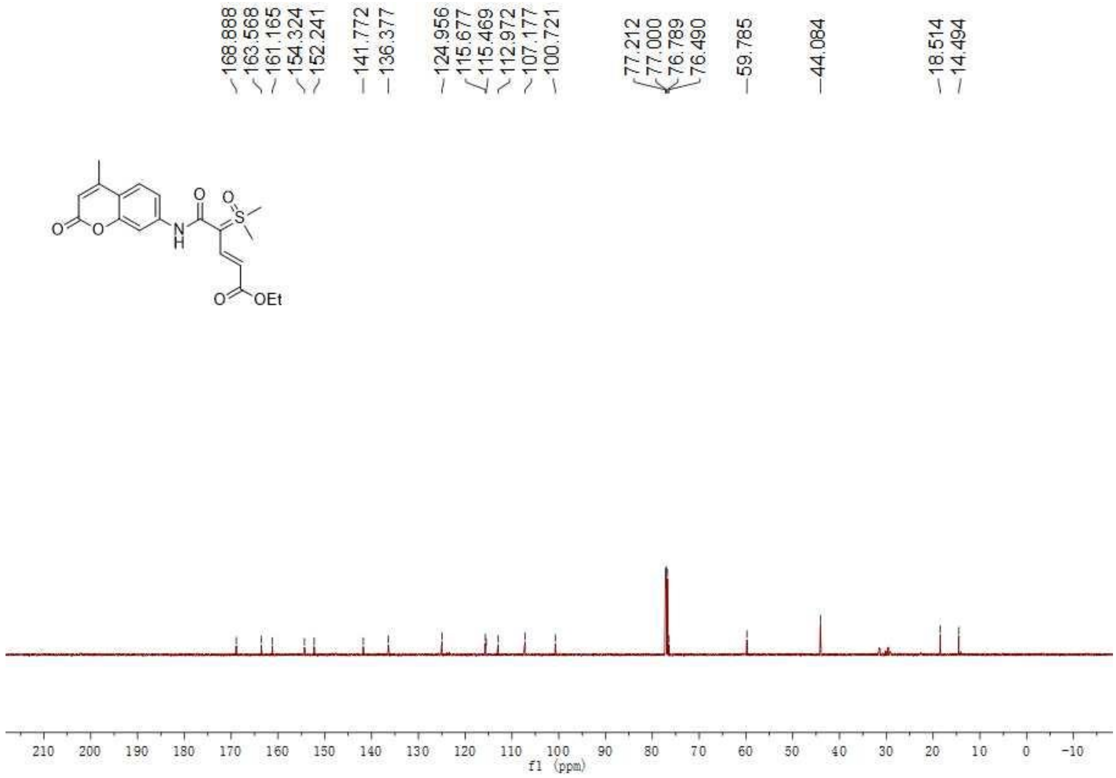

^{1}H NMR (600 MHz, $CDCl_3$) Spectrum of **48**

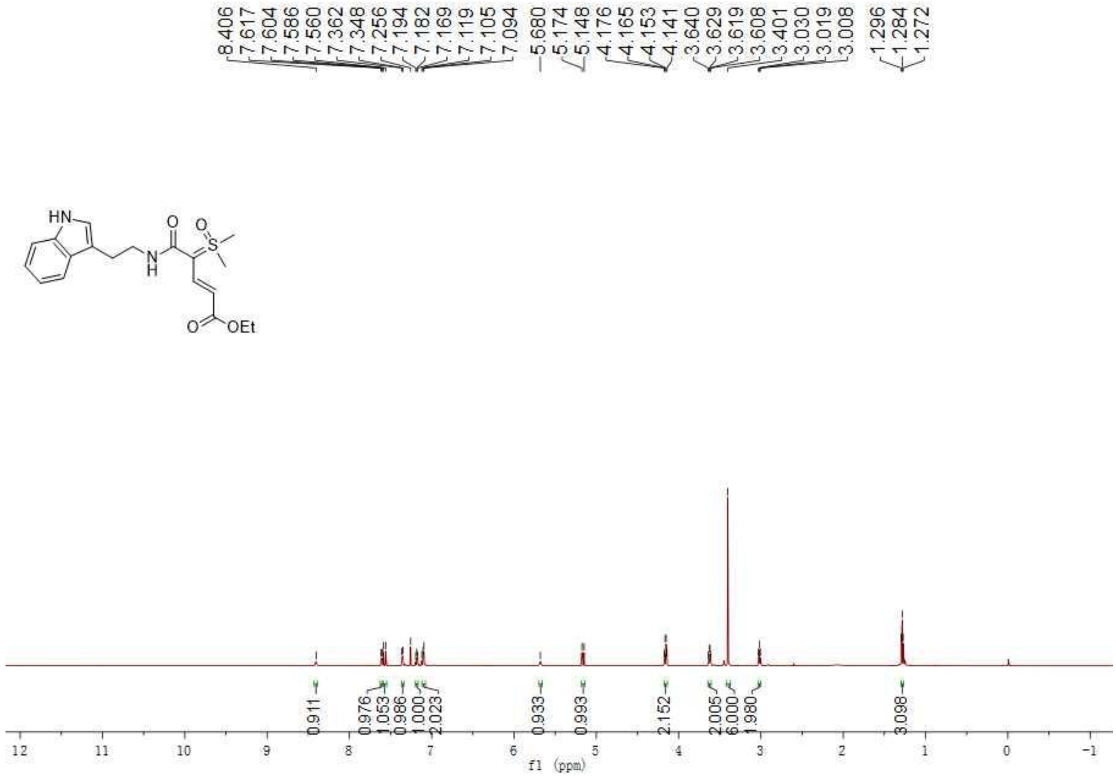

^{13}C NMR (150 MHz, $CDCl_3$) Spectrum of **48**

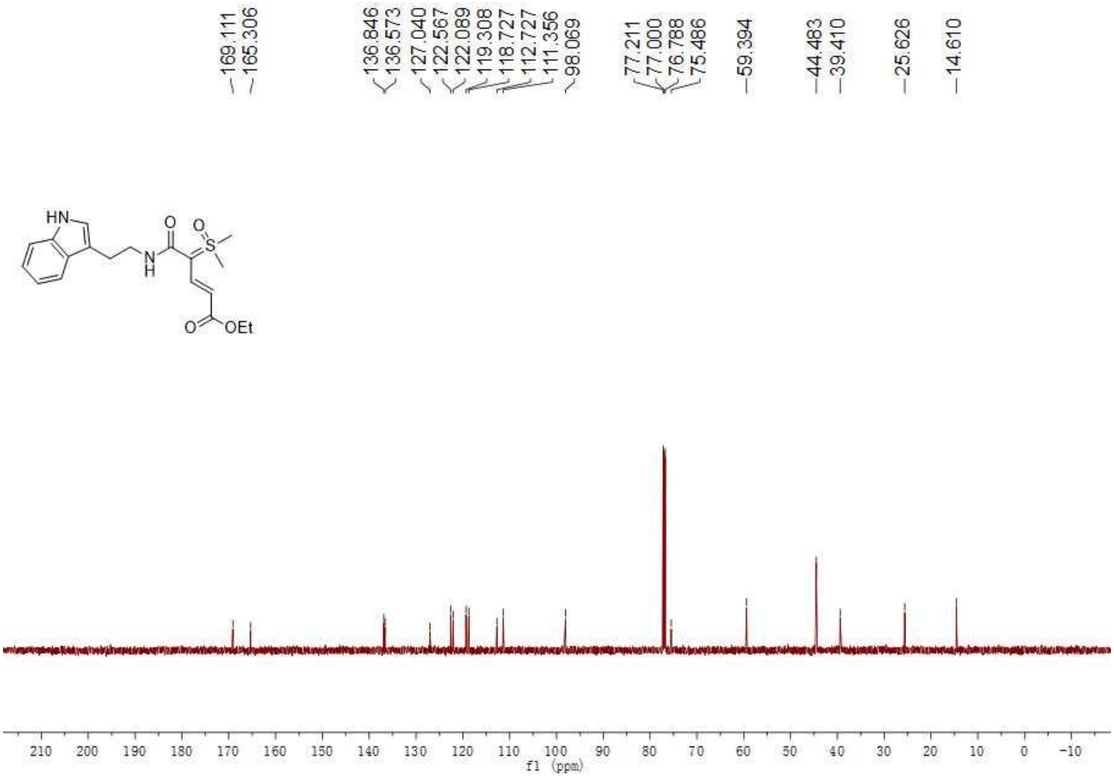

^{1}H NMR (600 MHz, $CDCl_3$) Spectrum of **49**

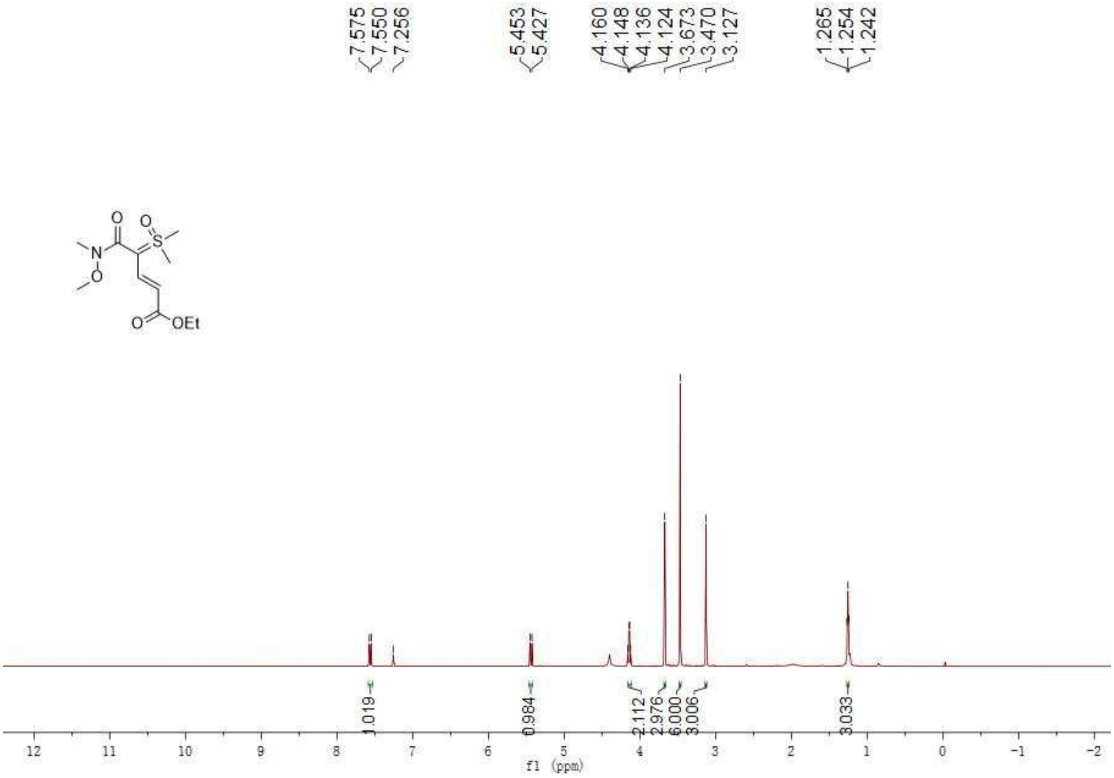

^{13}C NMR (150 MHz, $CDCl_3$) Spectrum of **49**

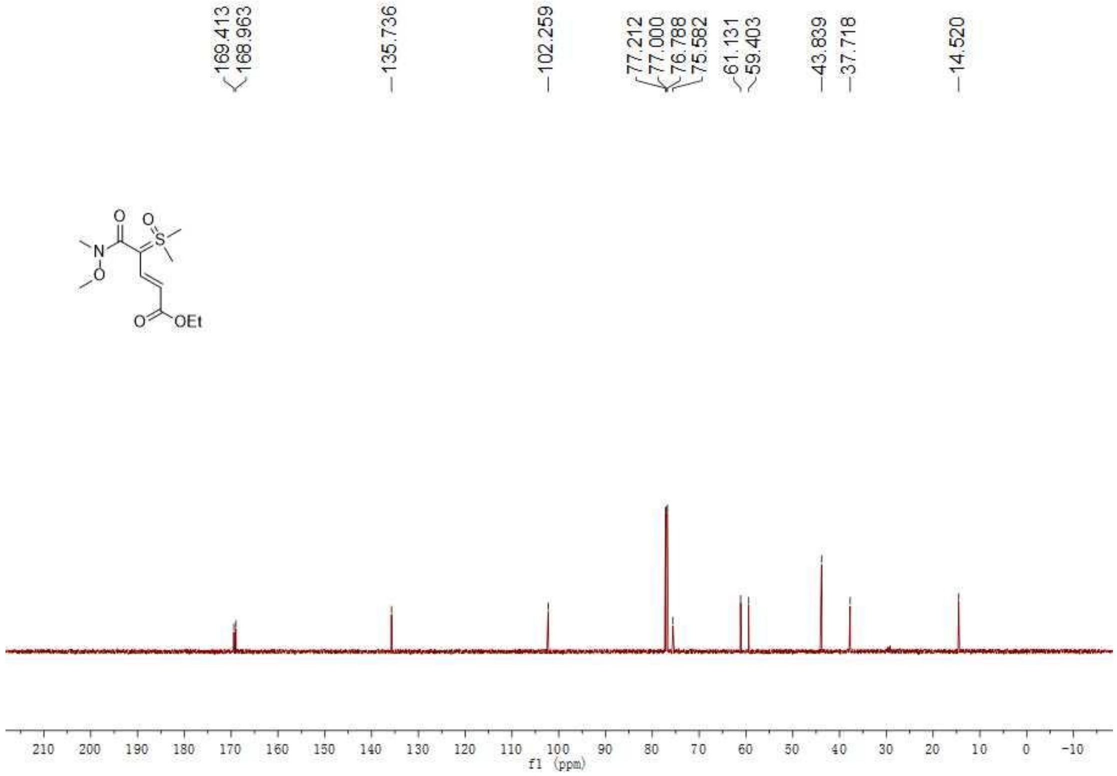

^{1}H NMR (600 MHz, $CDCl_3$) Spectrum of **50**

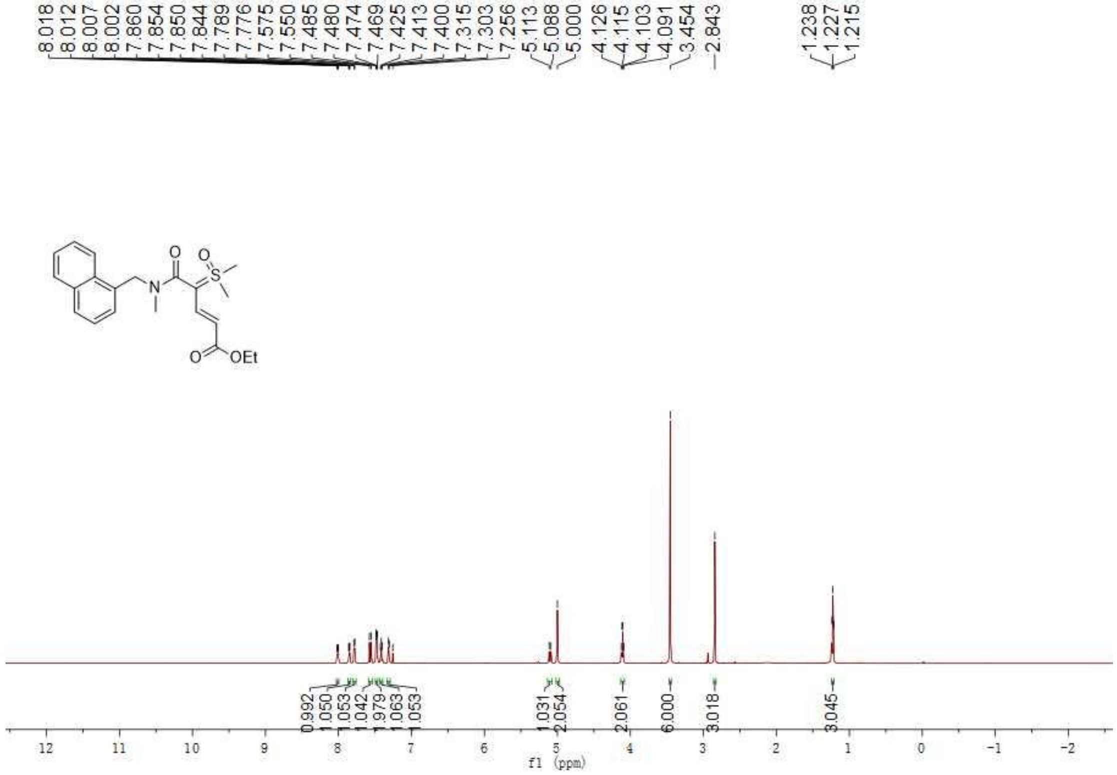

^{13}C NMR (150 MHz, $CDCl_3$) Spectrum of **50**

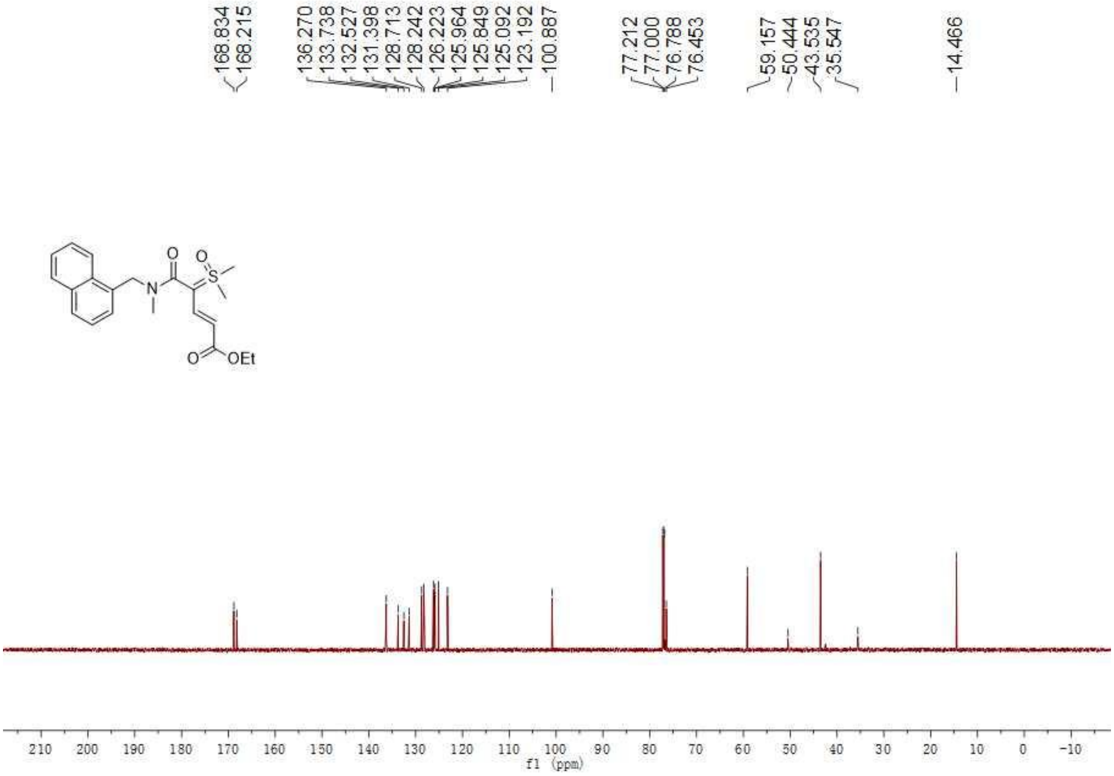

^1H NMR (600 MHz, $CDCl_3$) Spectrum of **51**

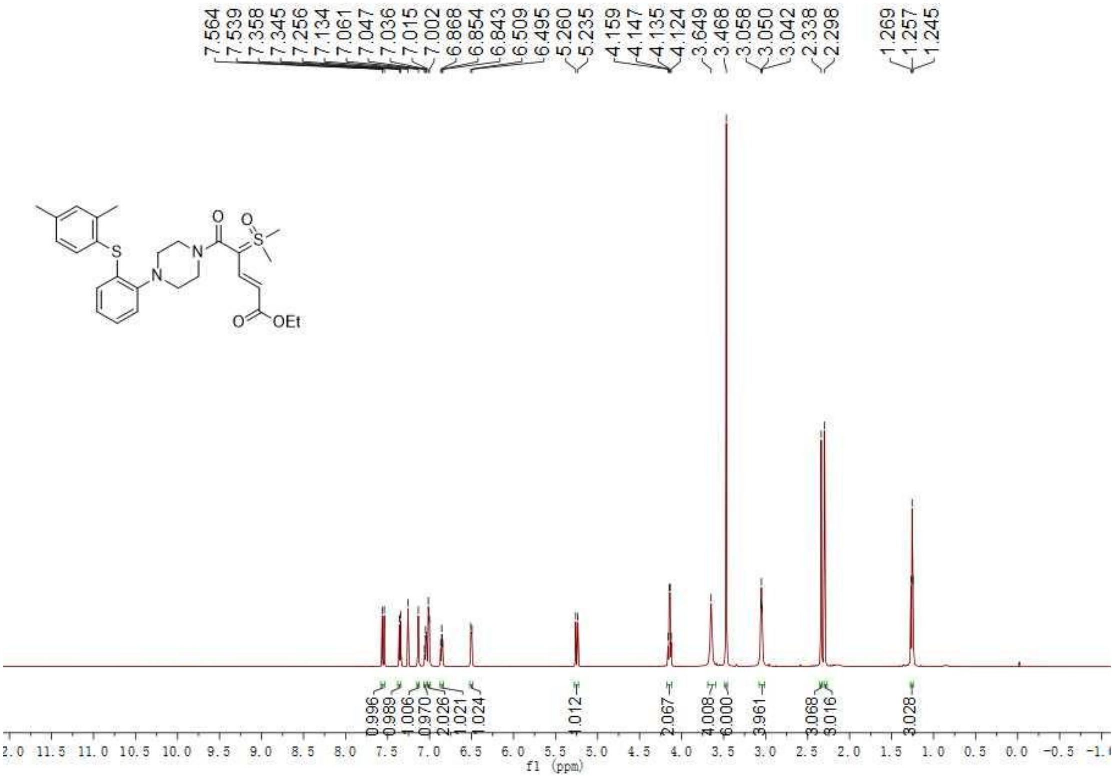

^{13}C NMR (150 MHz, $CDCl_3$) Spectrum of **51**

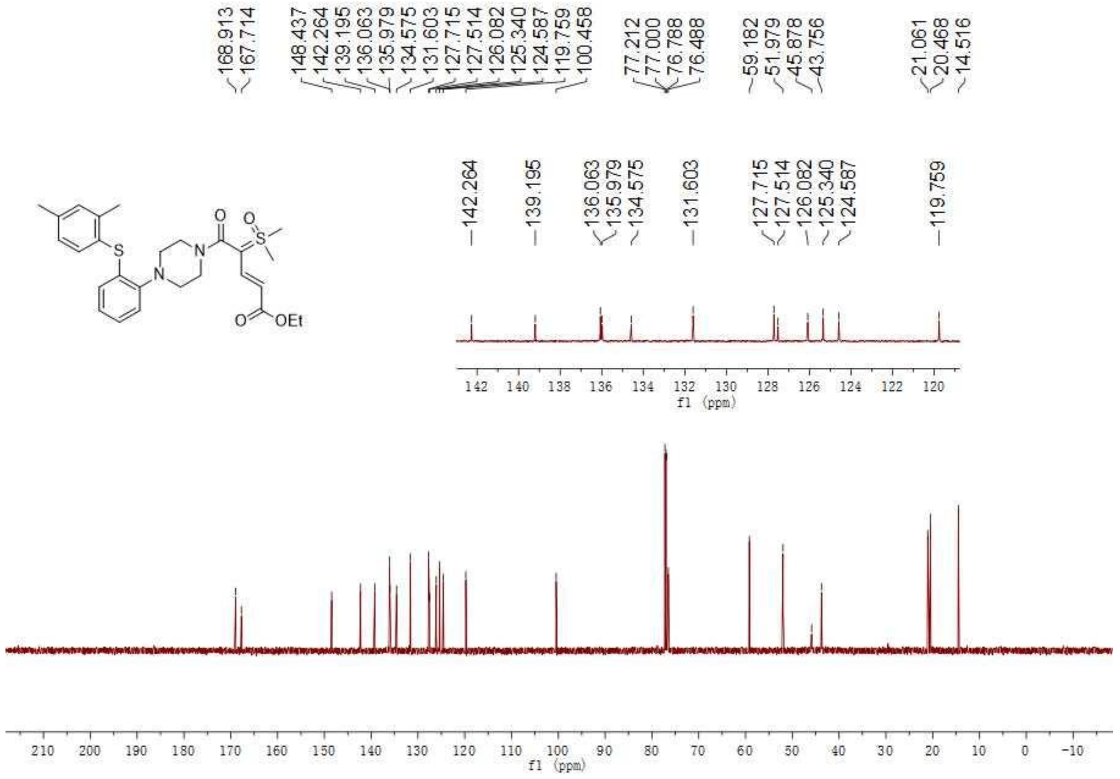

^{1}H NMR (600 MHz, $CDCl_3$) Spectrum of **52**

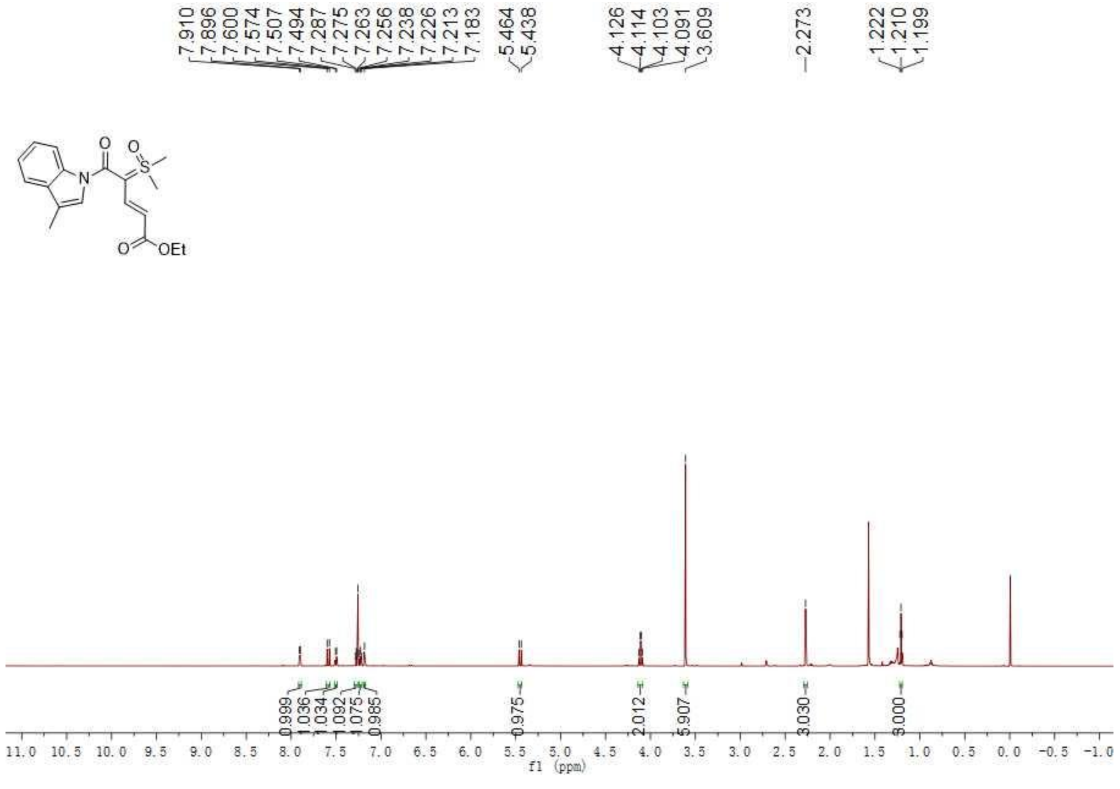

^{13}C NMR (150 MHz, $CDCl_3$) Spectrum of **52**

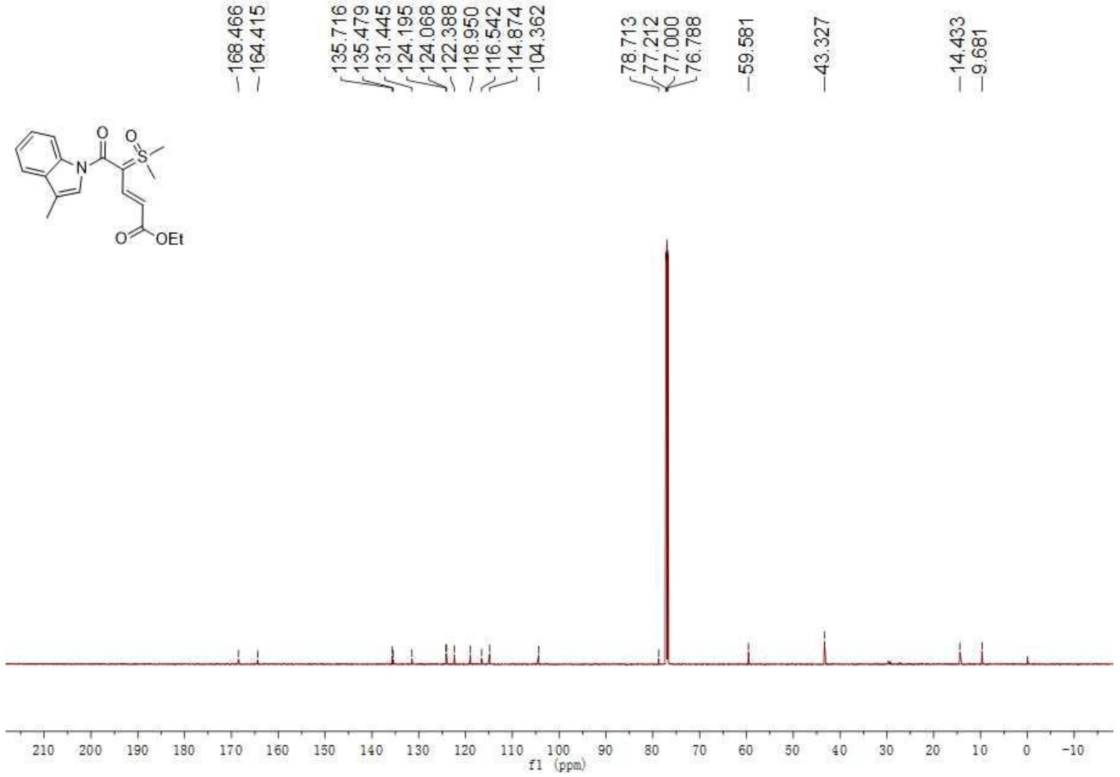

^{1}H NMR (600 MHz, $CDCl_3$) Spectrum of **53**

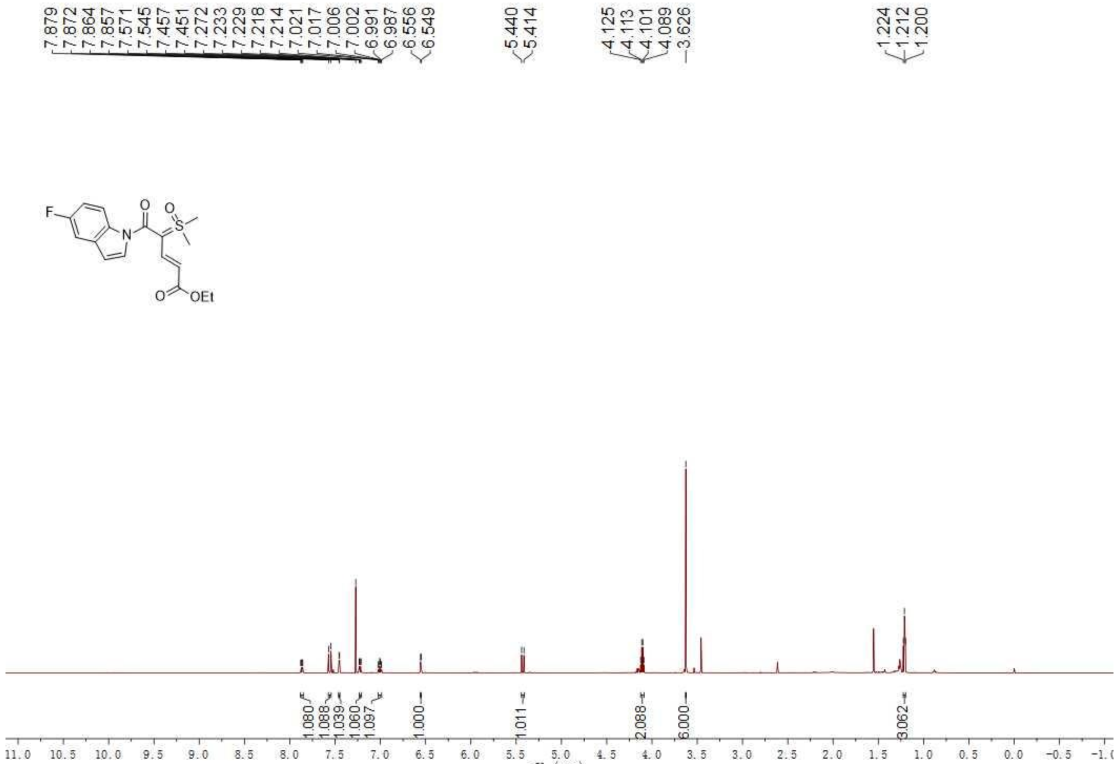

^{13}C NMR (150 MHz, $CDCl_3$) Spectrum of **53**

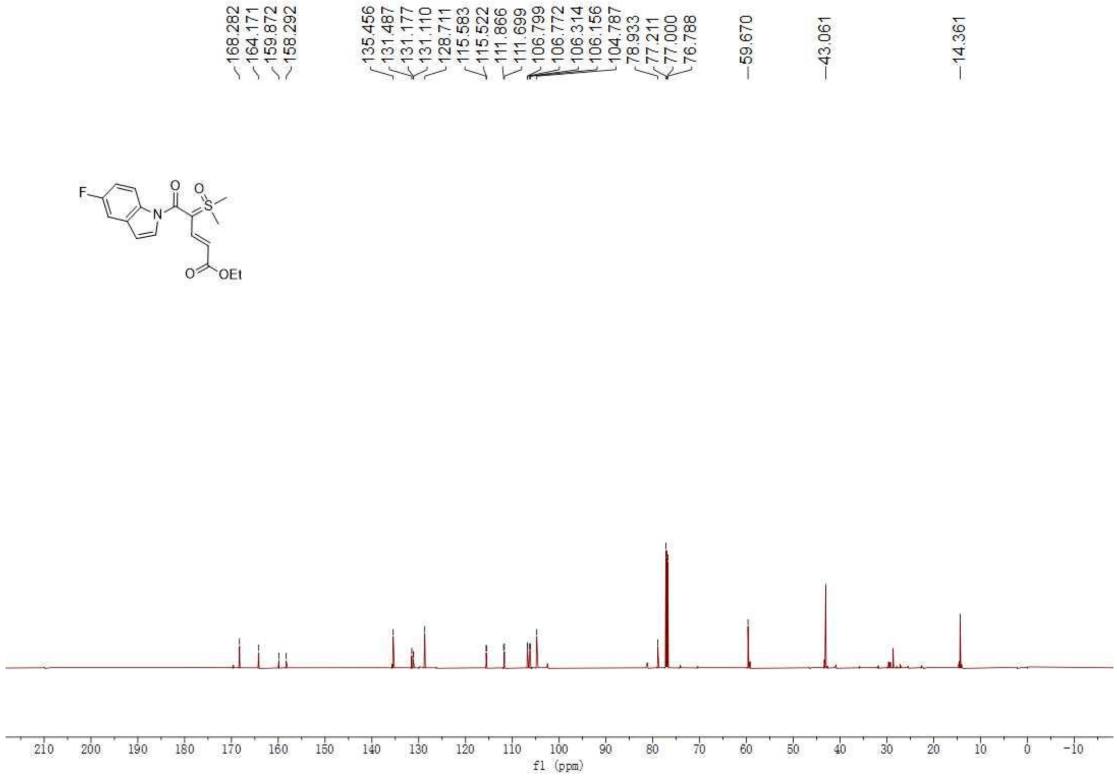

^{19}F NMR (564 MHz, $CDCl_3$) Spectrum of **53**

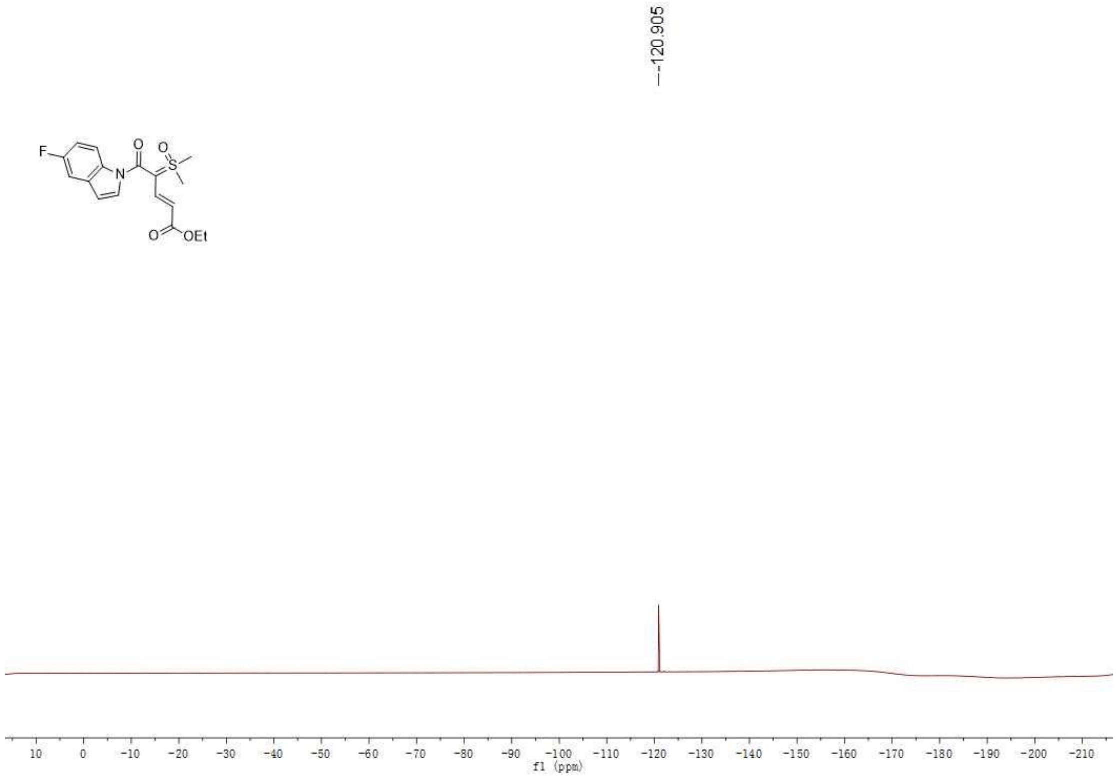

^{1}H NMR (600 MHz, $CDCl_3$) Spectrum of **54**

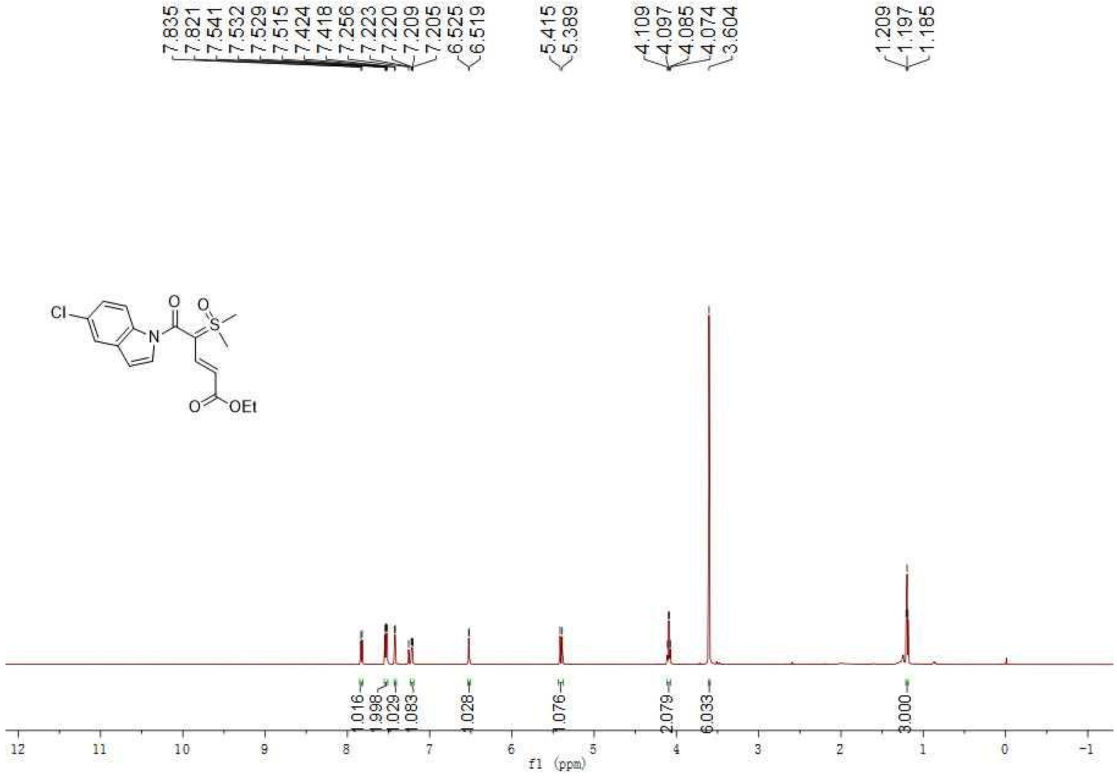

^{13}C NMR (150 MHz, $CDCl_3$) Spectrum of **54**

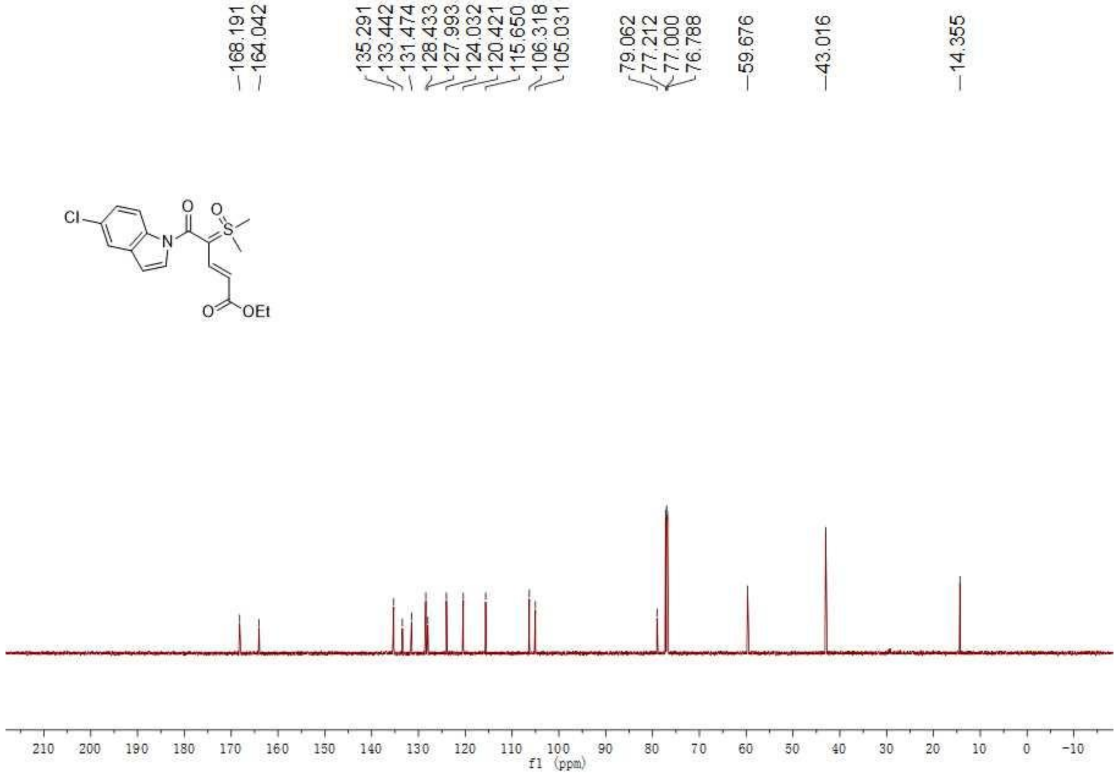

^{1}H NMR (600 MHz, $CDCl_3$) Spectrum of **55**

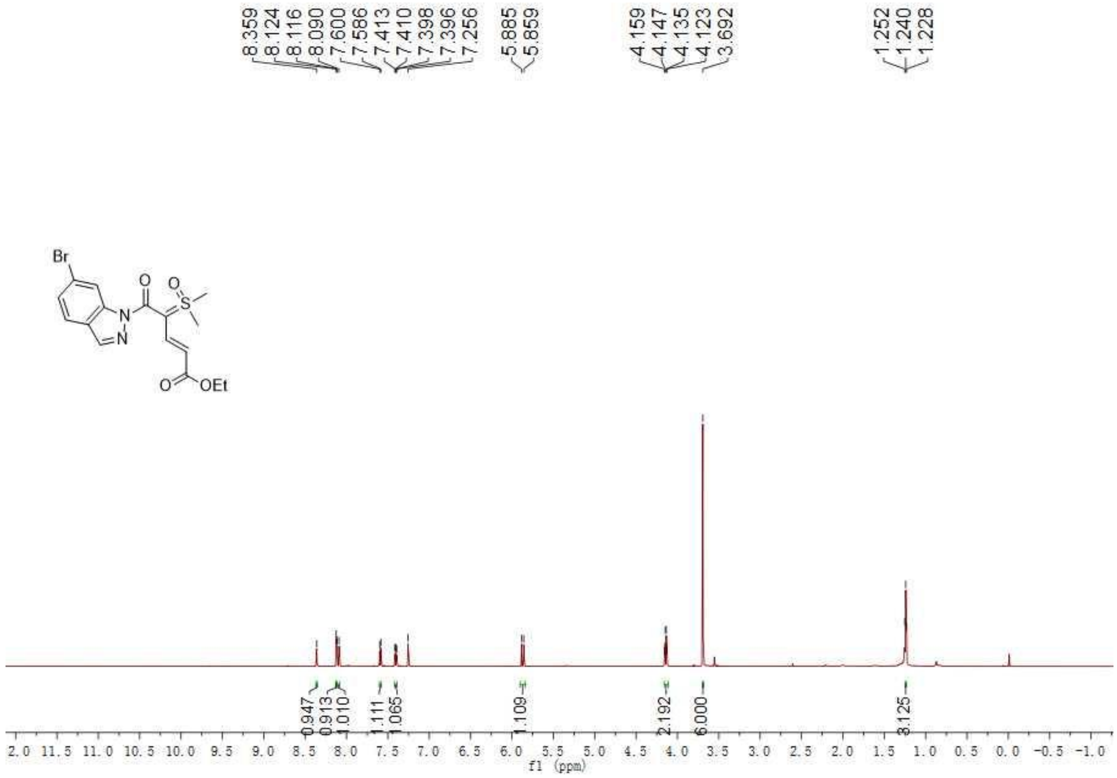

^{13}C NMR (150 MHz, $CDCl_3$) Spectrum of **55**

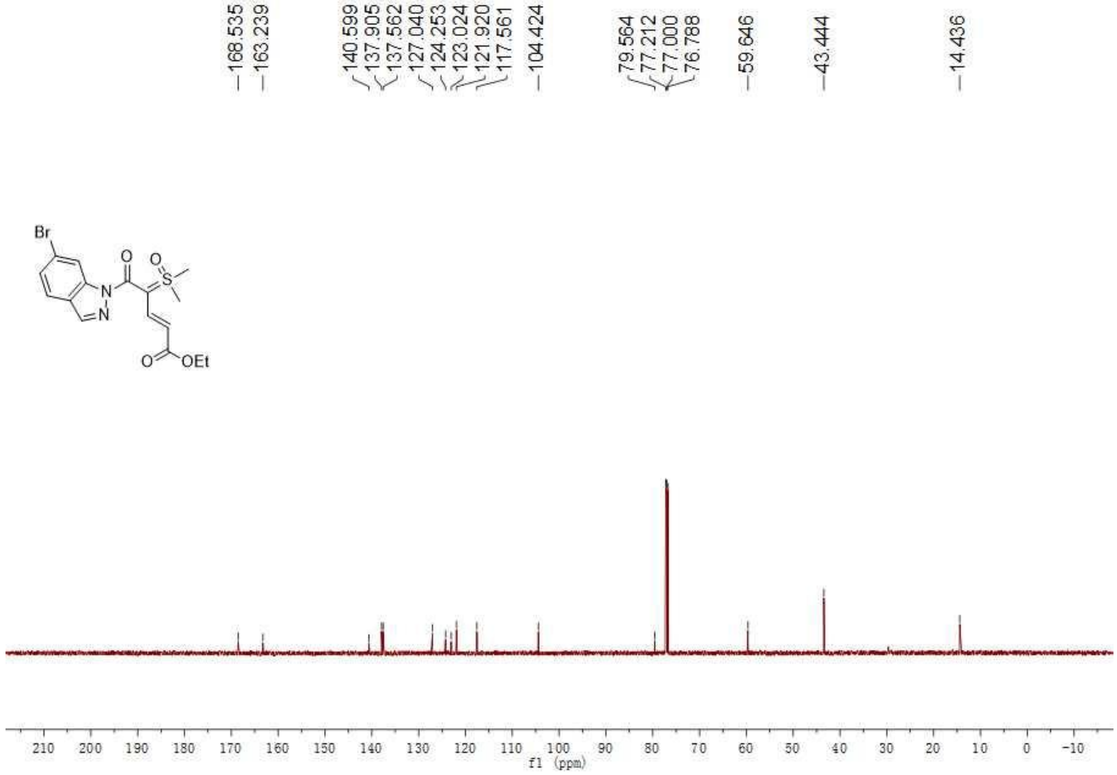

^{1}H NMR (600 MHz, $CDCl_3$) Spectrum of **56**

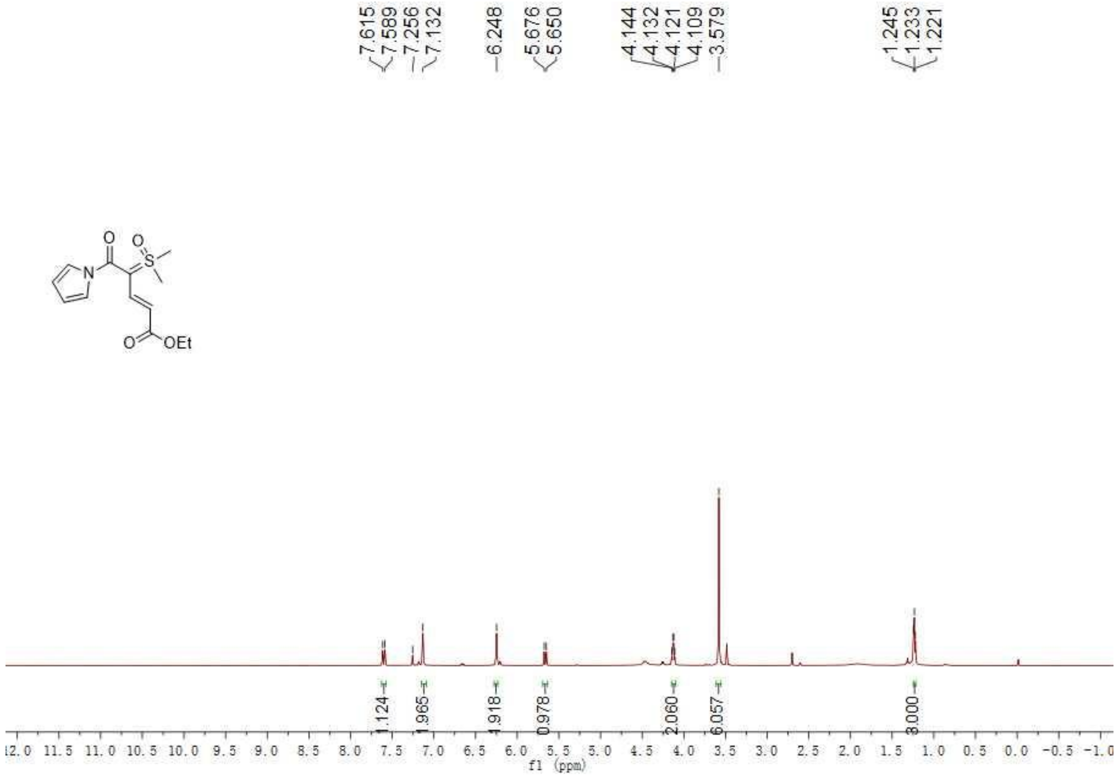

^{13}C NMR (150 MHz, $CDCl_3$) Spectrum of **56**

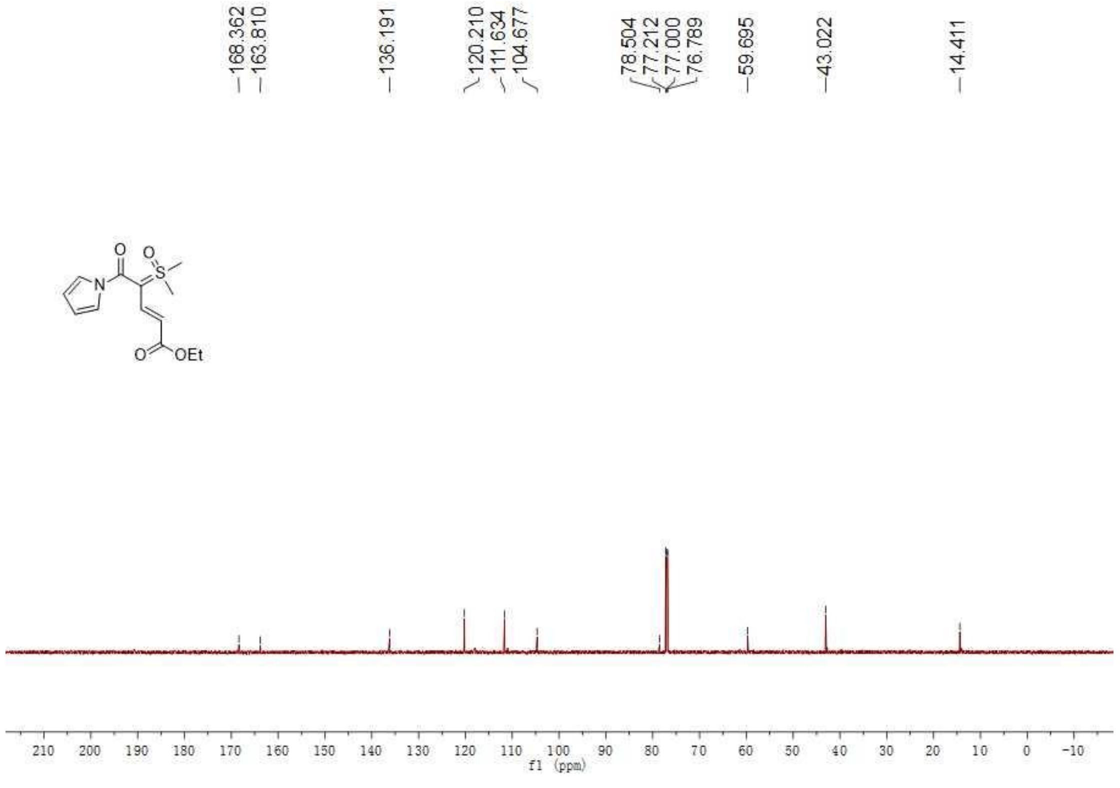

^{1}H NMR (600 MHz, $CDCl_3$) Spectrum of **57**

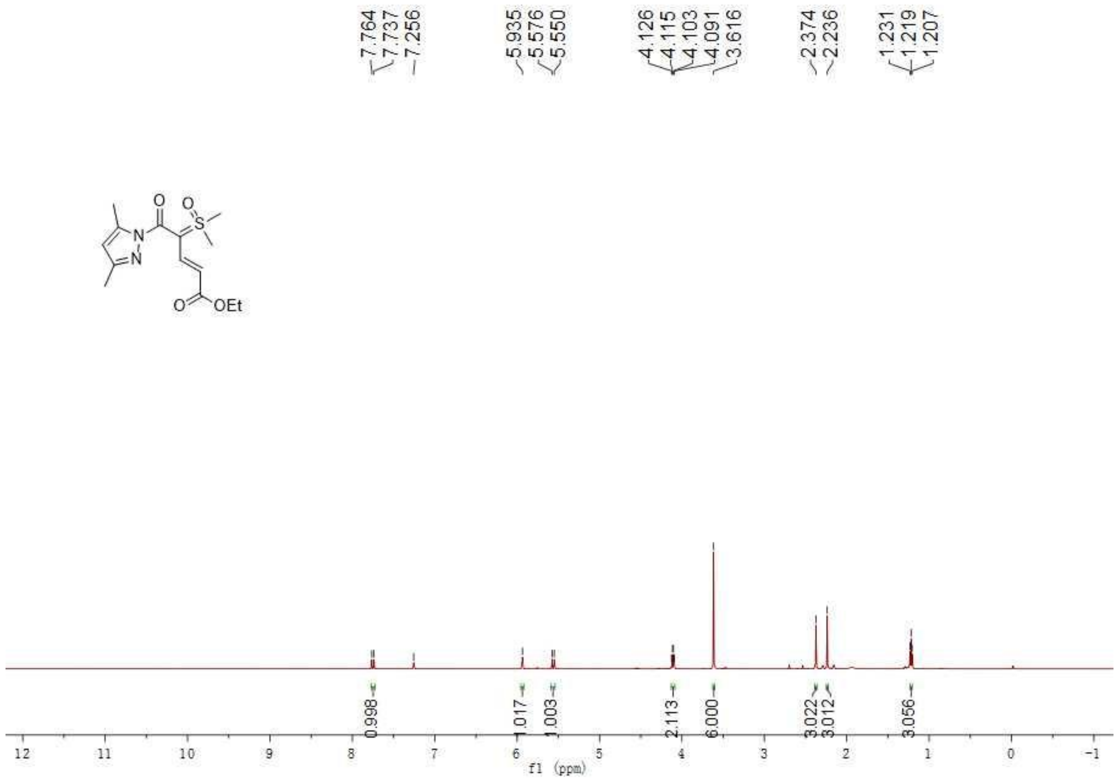

^{13}C NMR (150 MHz, $CDCl_3$) Spectrum of **57**

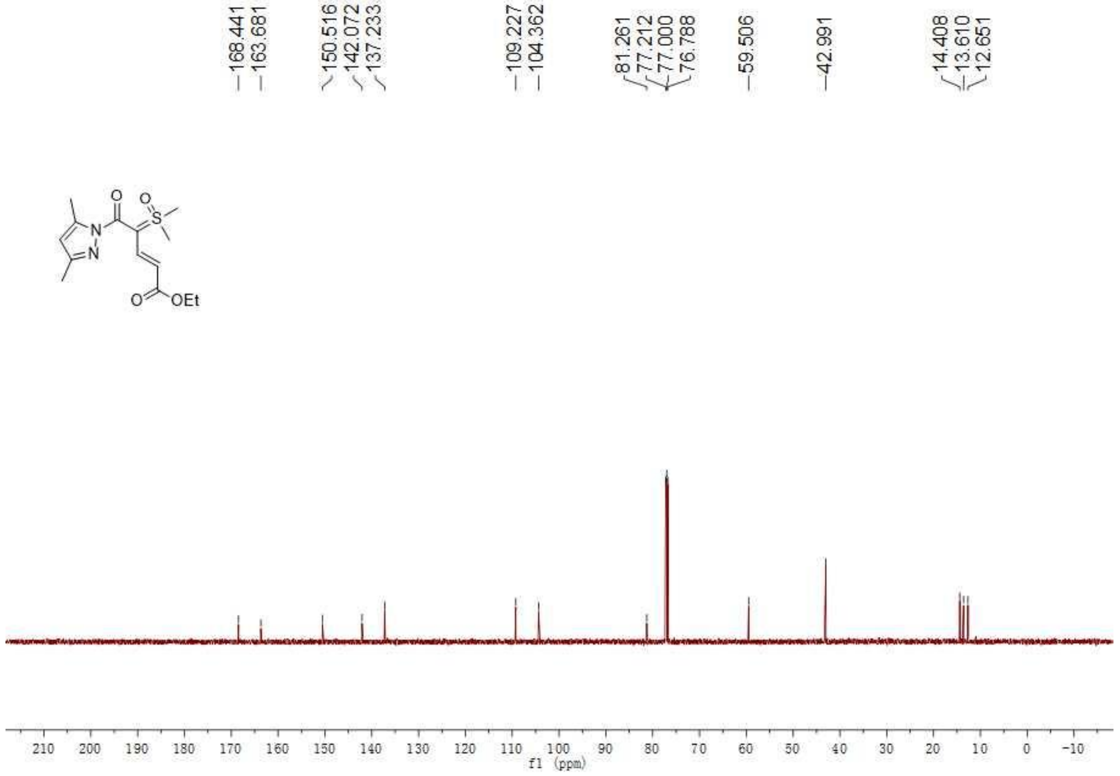

^{1}H NMR (600 MHz, $CDCl_3$) Spectrum of **58**

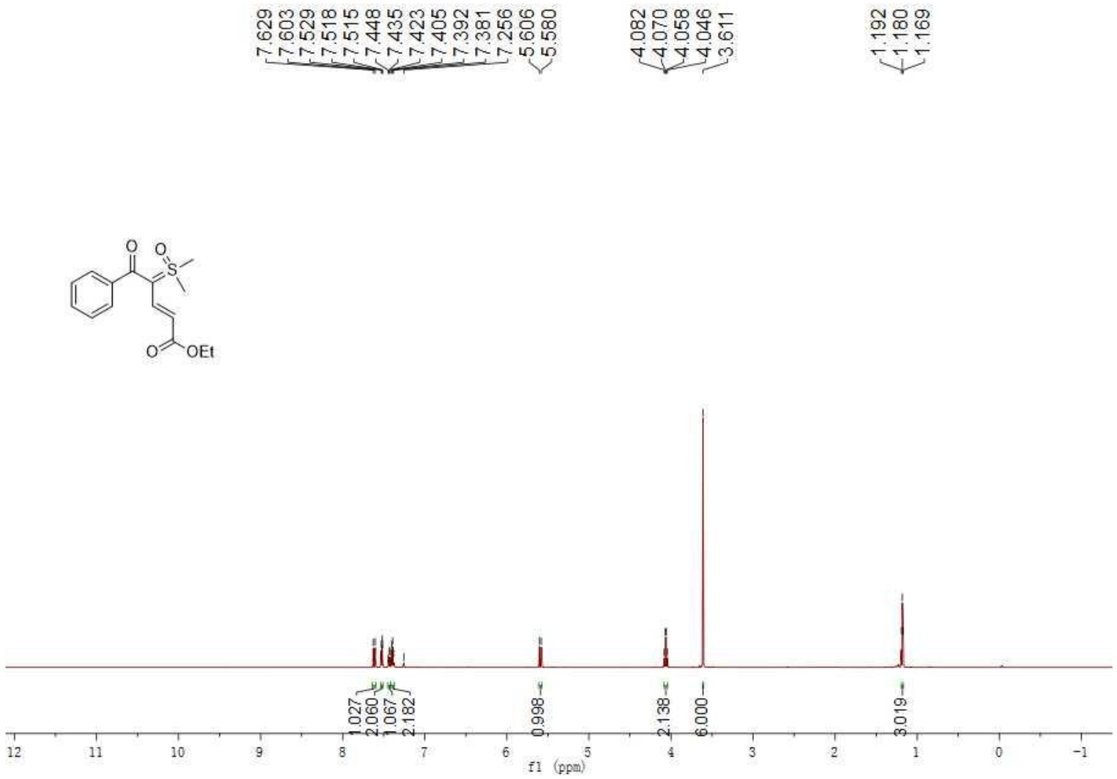

^{13}C NMR (150 MHz, $CDCl_3$) Spectrum of **58**

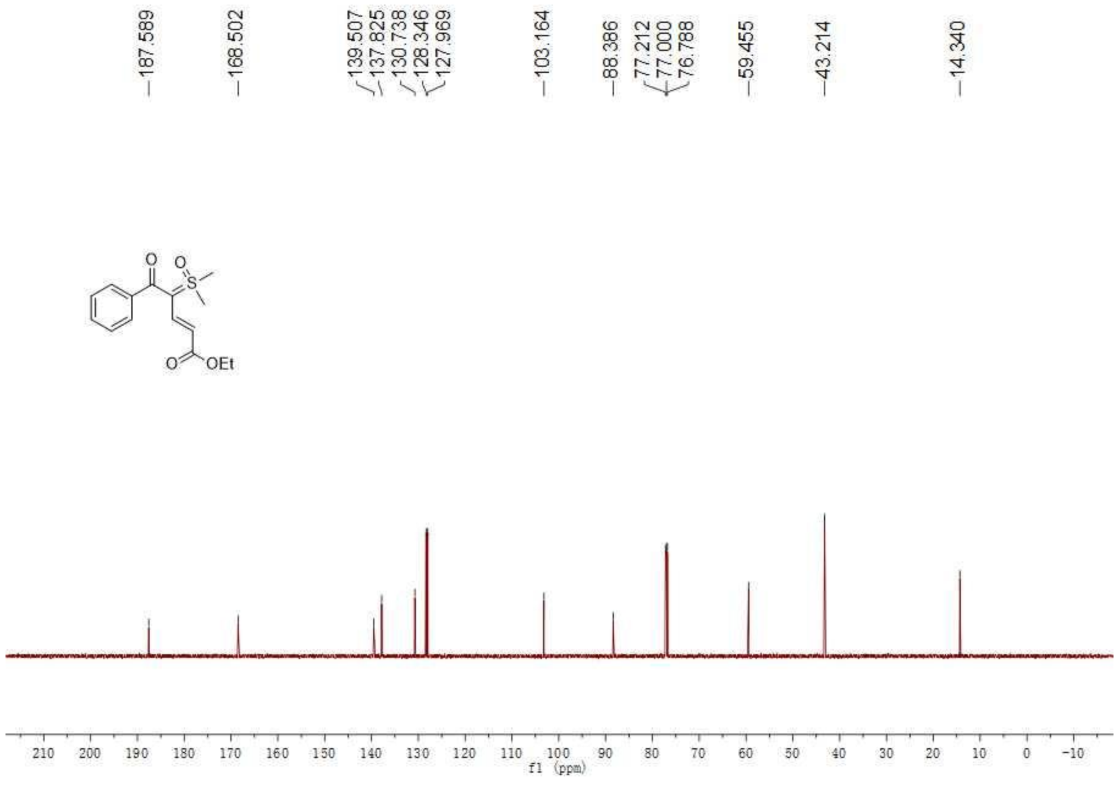

^{1}H NMR (600 MHz, $CDCl_3$) Spectrum of **59**

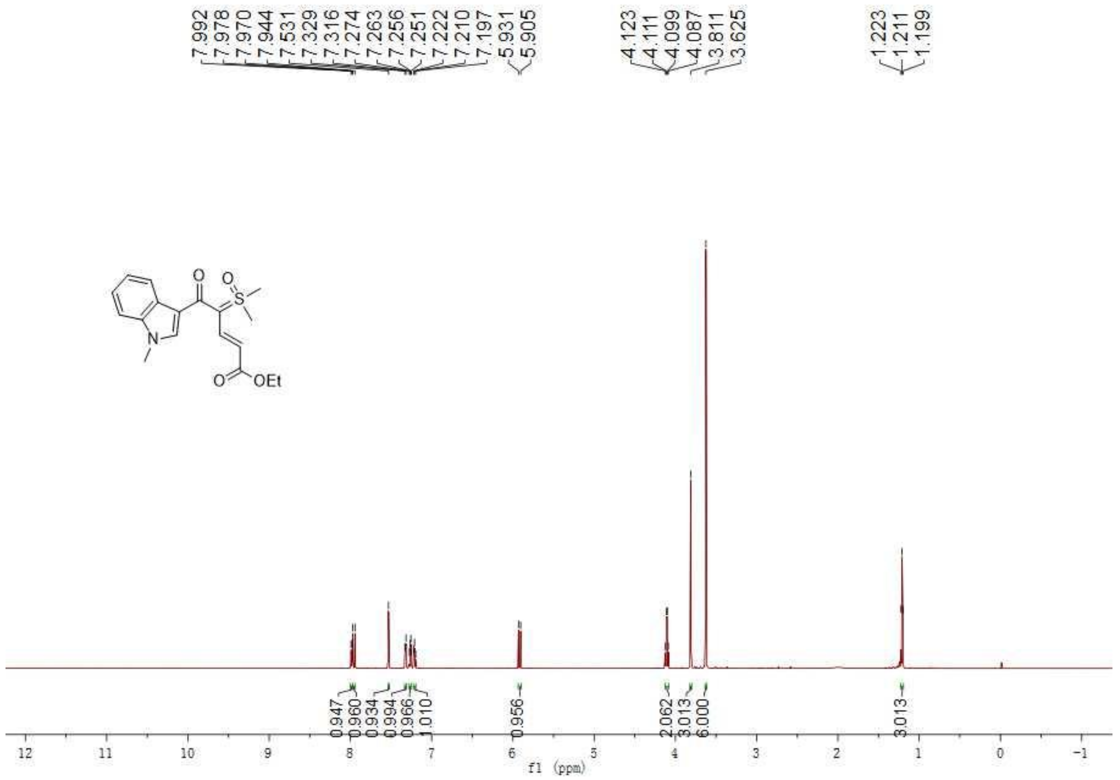

^{13}C NMR (150 MHz, $CDCl_3$) Spectrum of **59**

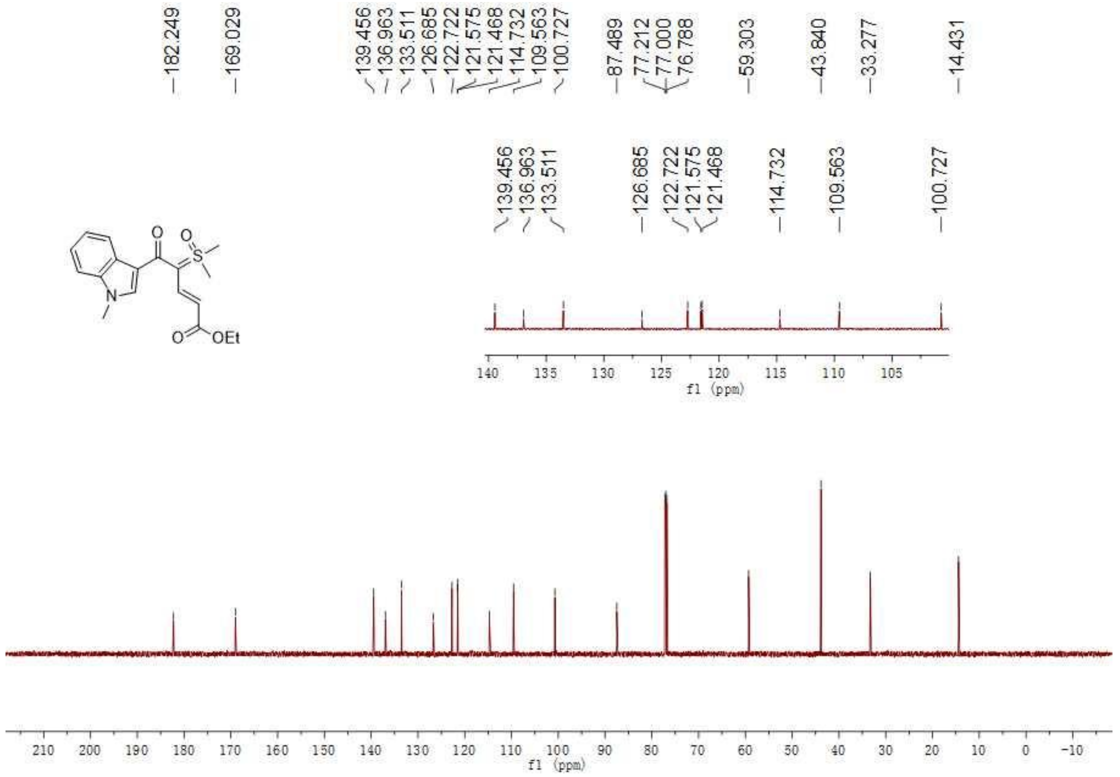

^{1}H NMR (600 MHz, $CDCl_3$) Spectrum of **60**

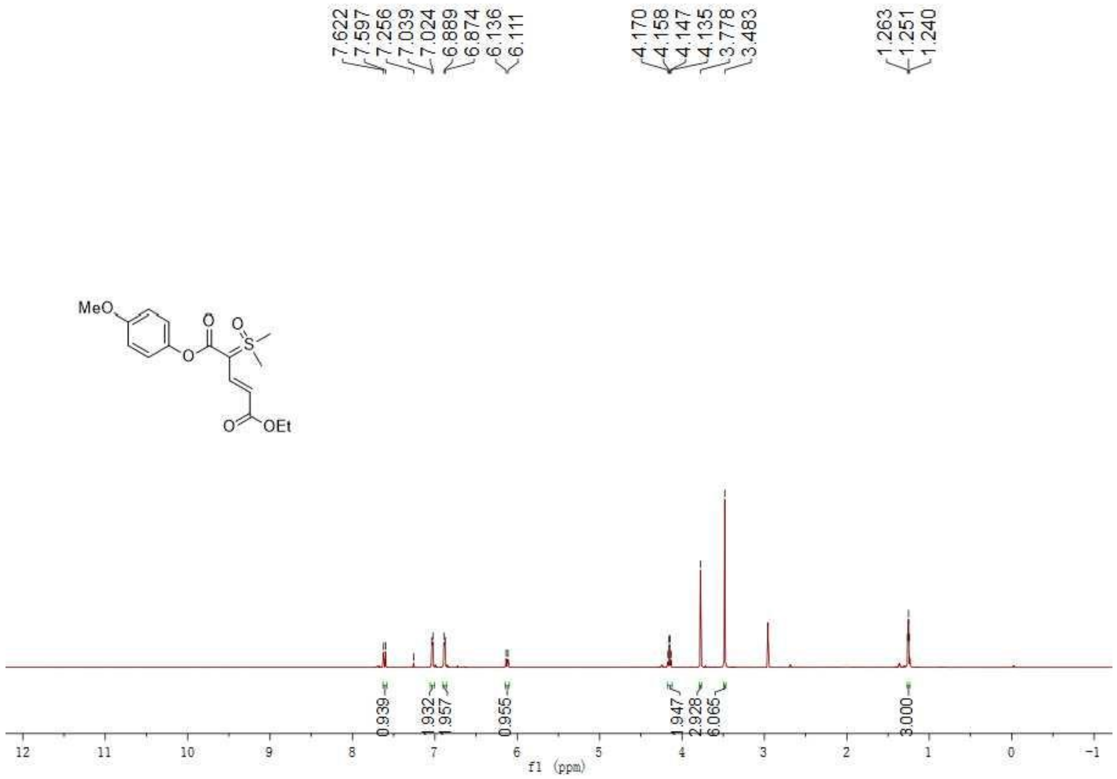

^{13}C NMR (150 MHz, $CDCl_3$) Spectrum of **60**

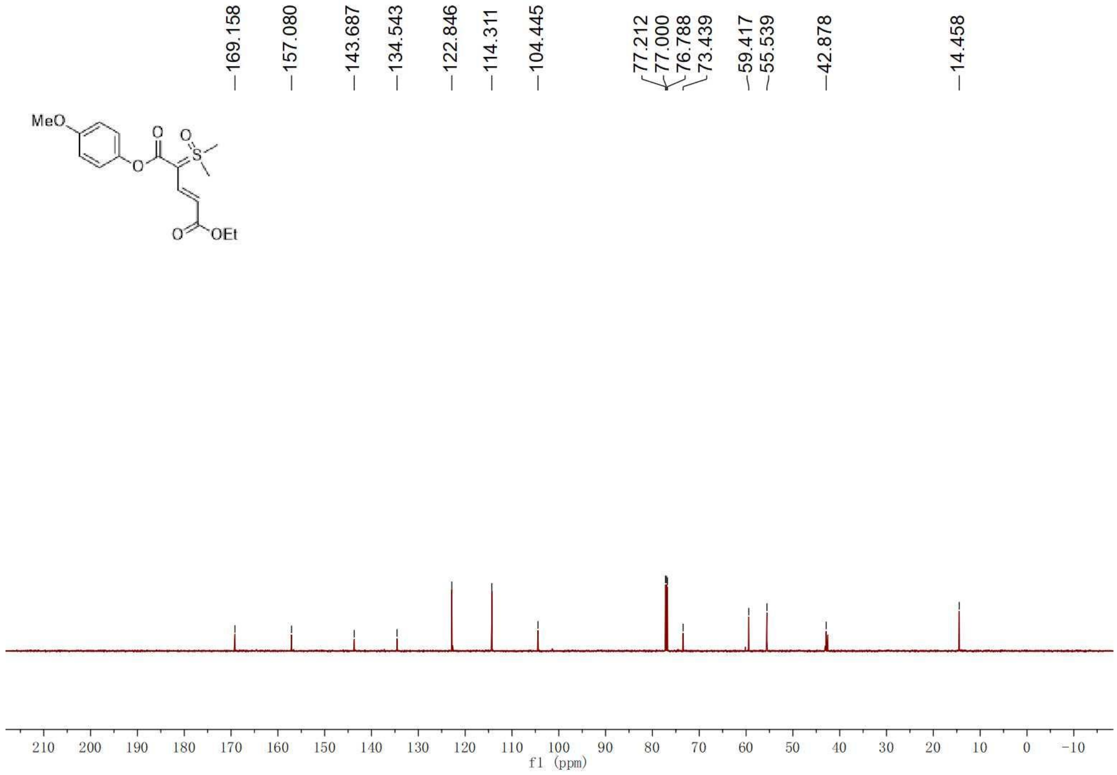

^{1}H NMR (600 MHz, $CDCl_3$) Spectrum of **61**

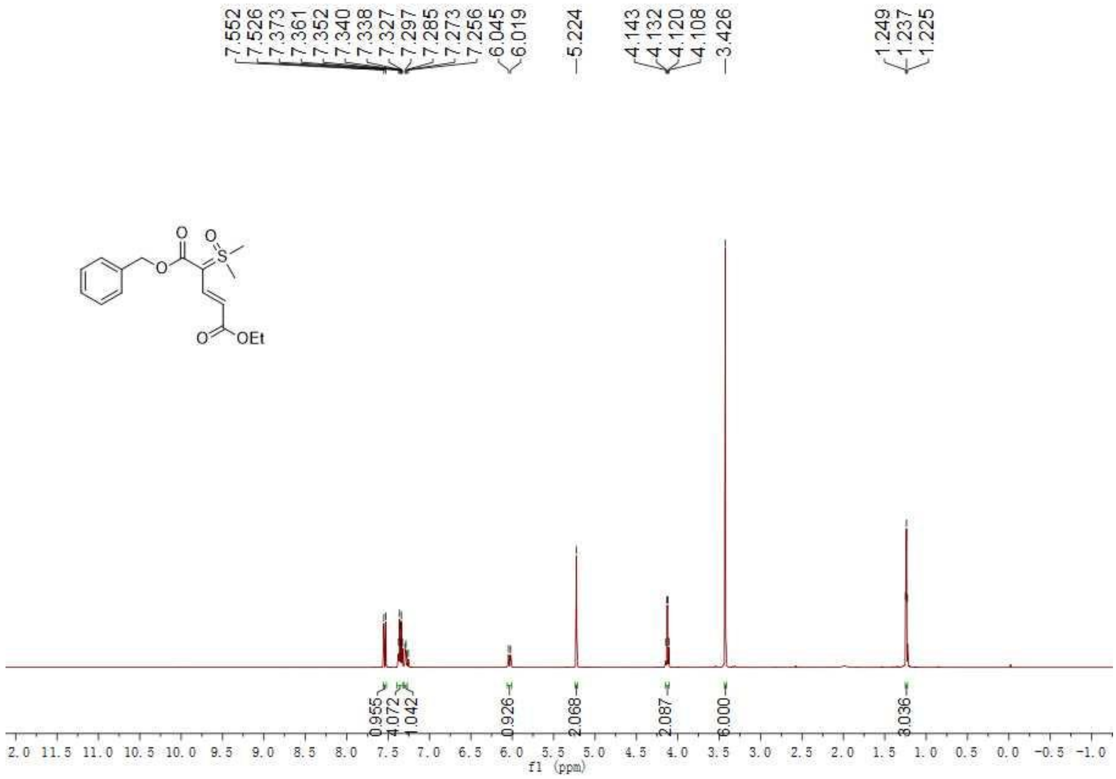

^{13}C NMR (150 MHz, $CDCl_3$) Spectrum of **61**

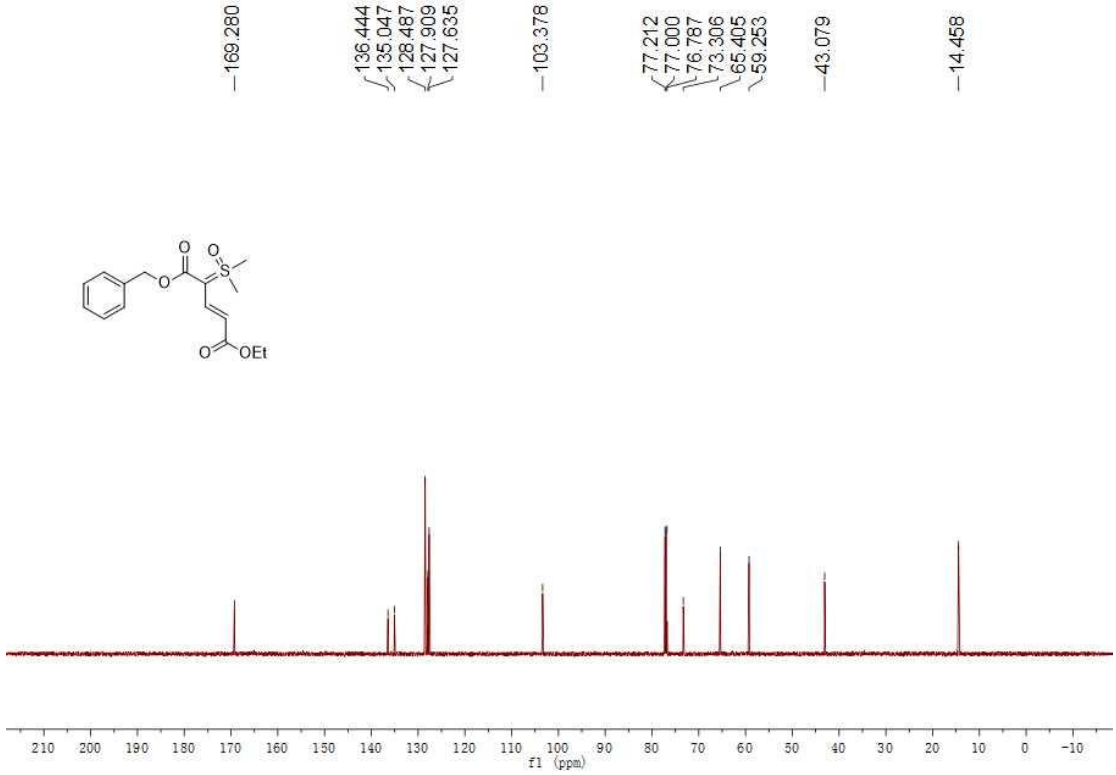

^{1}H NMR (600 MHz, $CDCl_3$) Spectrum of **62**

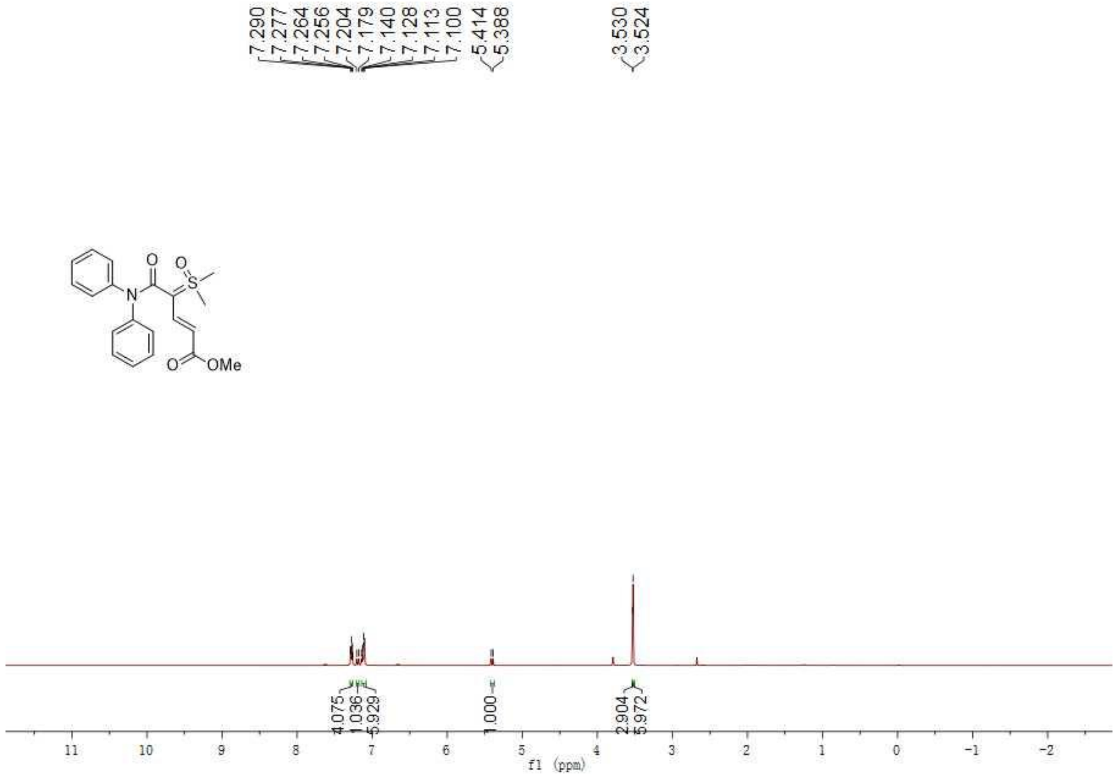

^{13}C NMR (150 MHz, $CDCl_3$) Spectrum of **62**

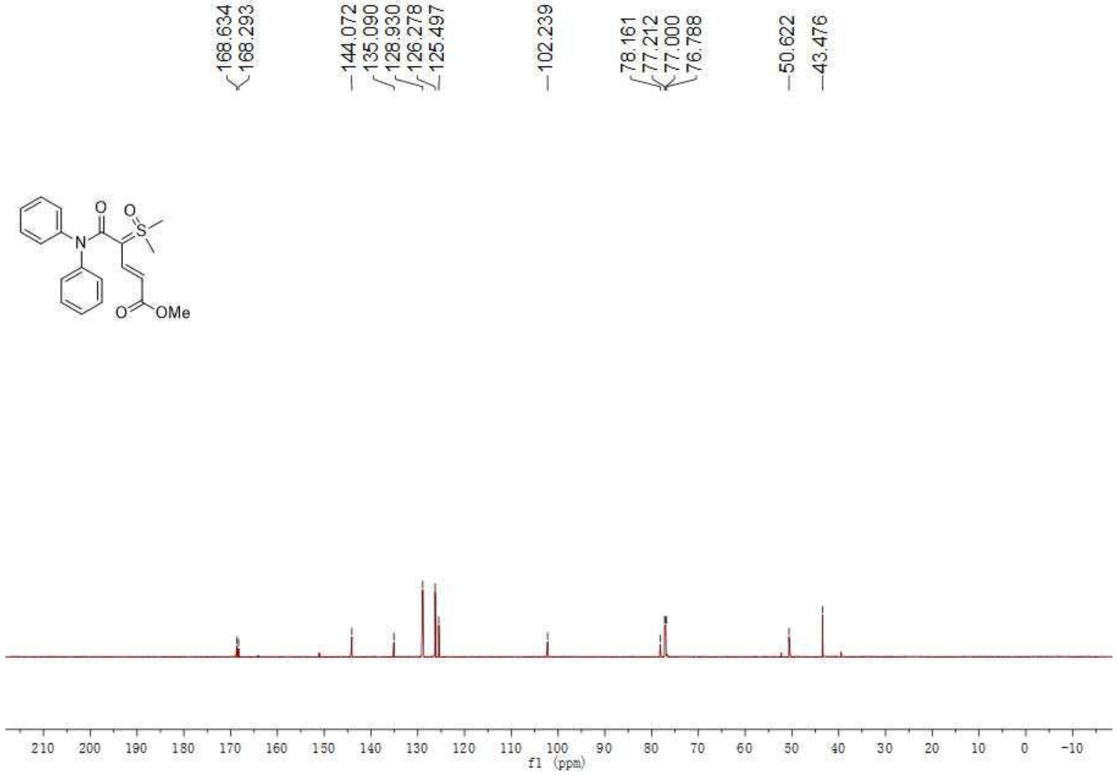

^{1}H NMR (600 MHz, $CDCl_3$) Spectrum of **63**

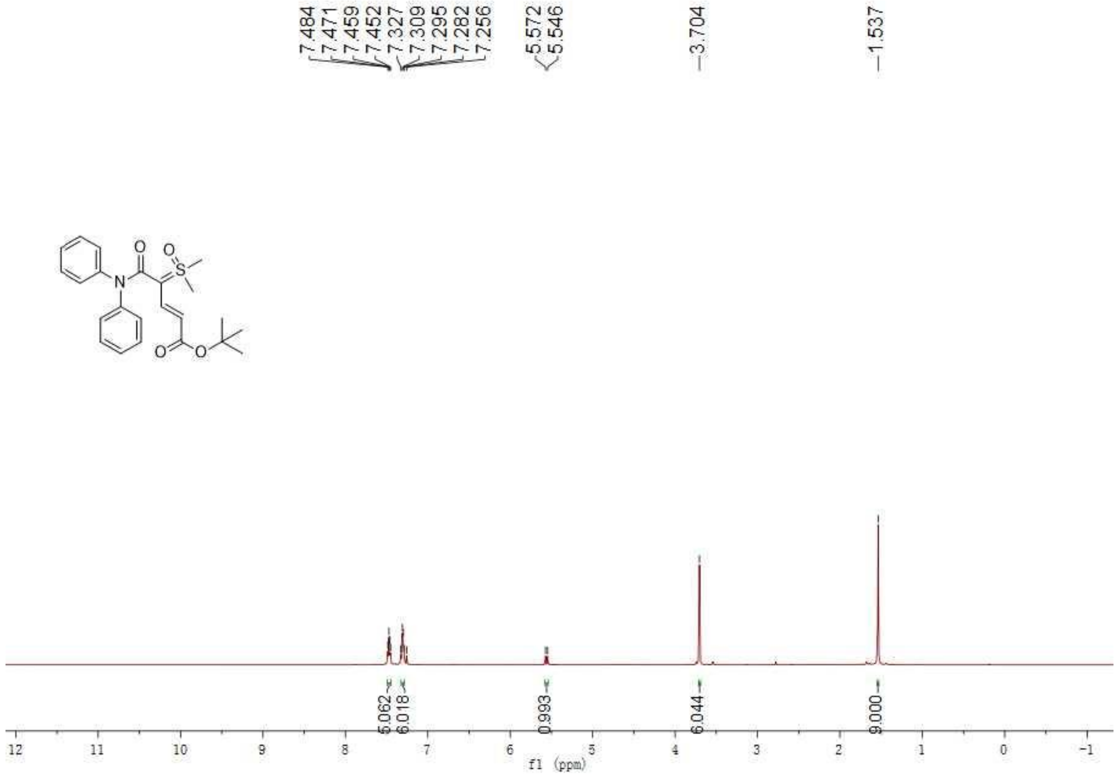

^{13}C NMR (150 MHz, $CDCl_3$) Spectrum of **63**

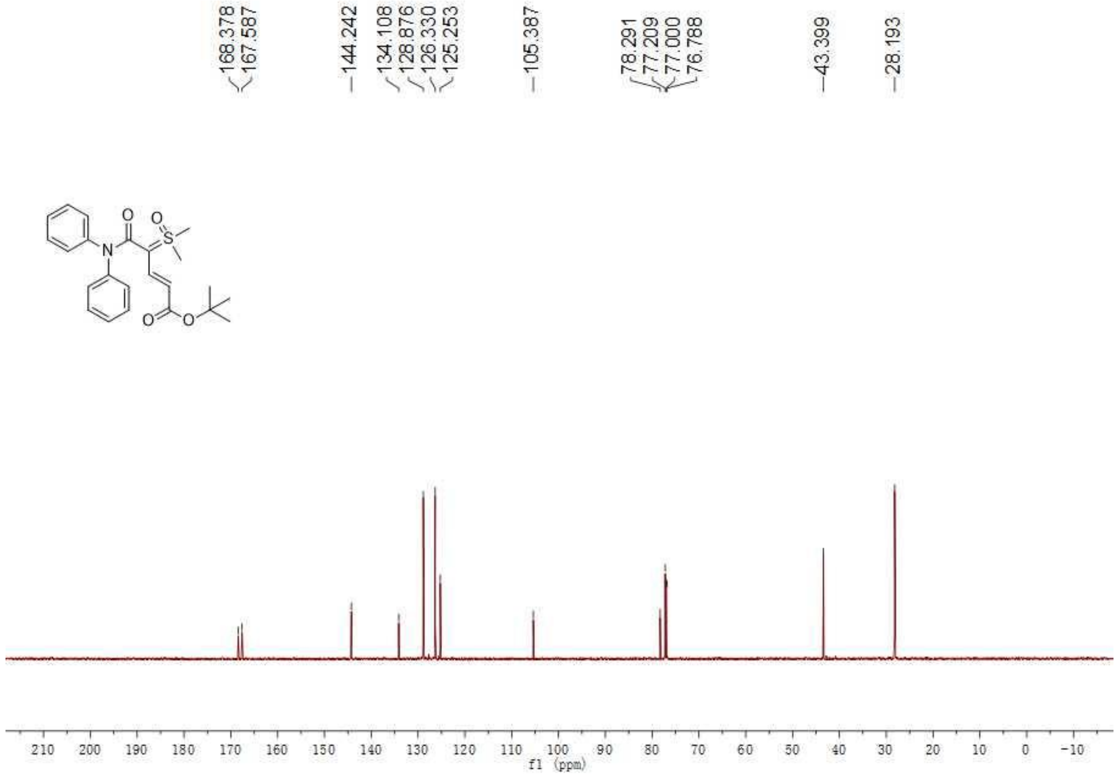

^{1}H NMR (600 MHz, $CDCl_3$) Spectrum of **64**

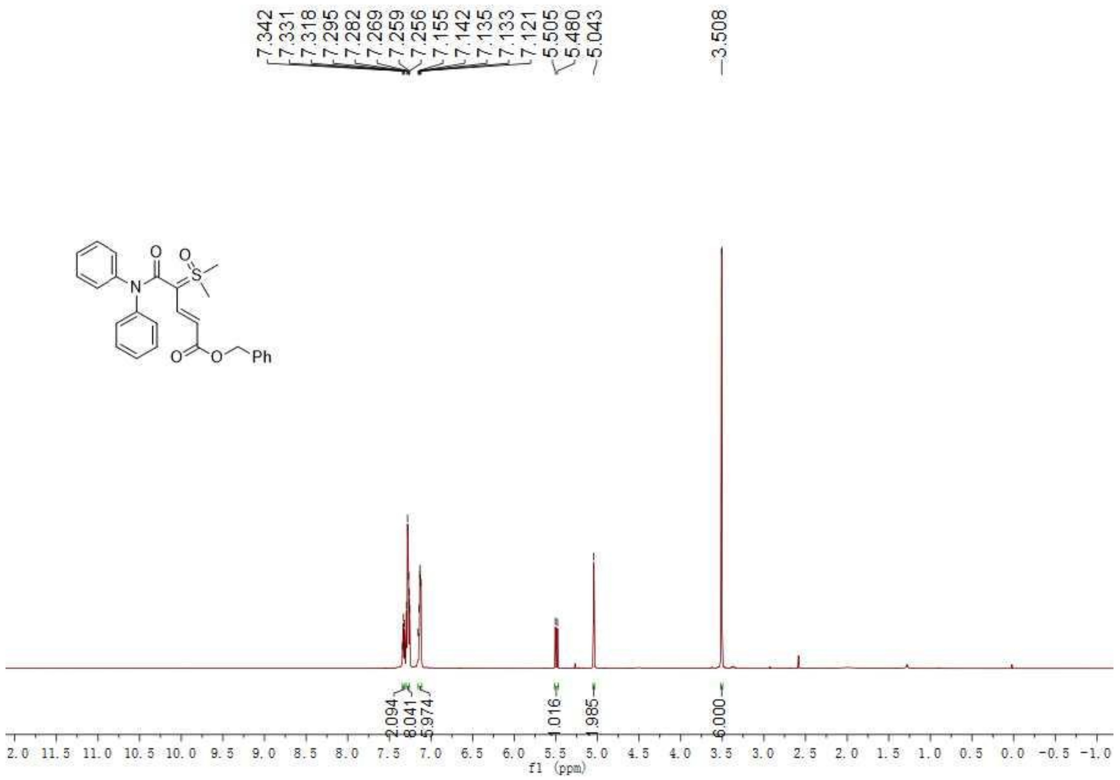

^{13}C NMR (150 MHz, $CDCl_3$) Spectrum of **64**

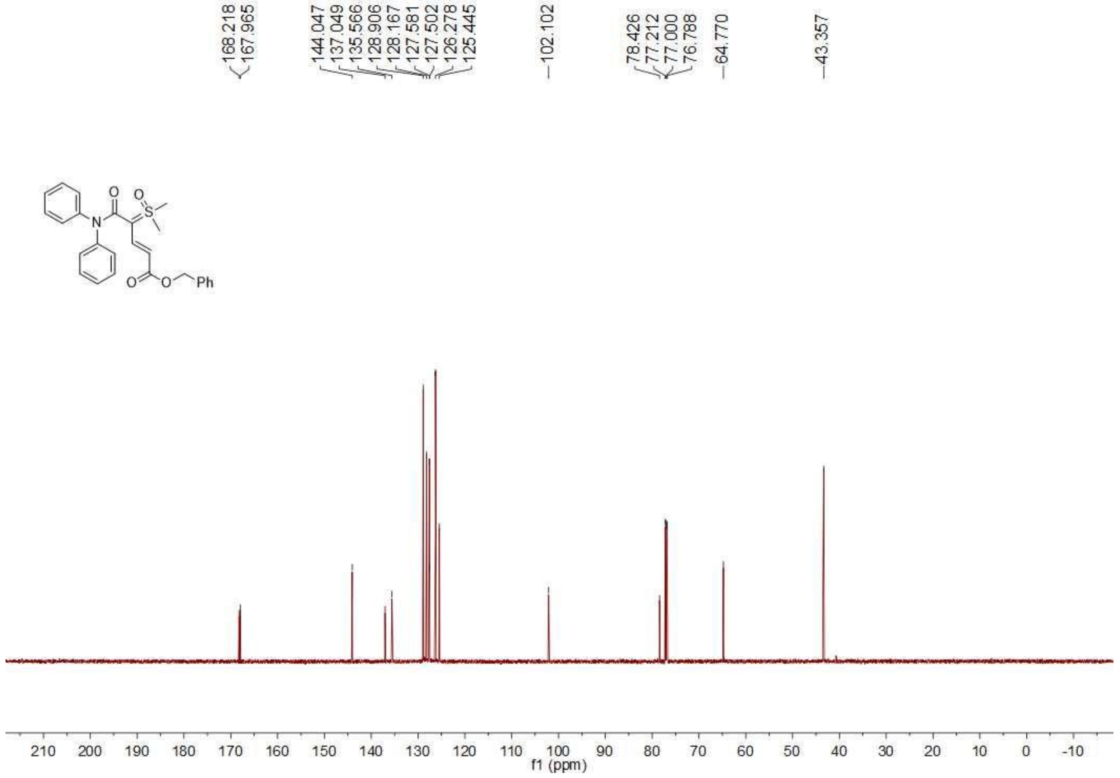

^{1}H NMR (600 MHz, $CDCl_3$) Spectrum of **65**

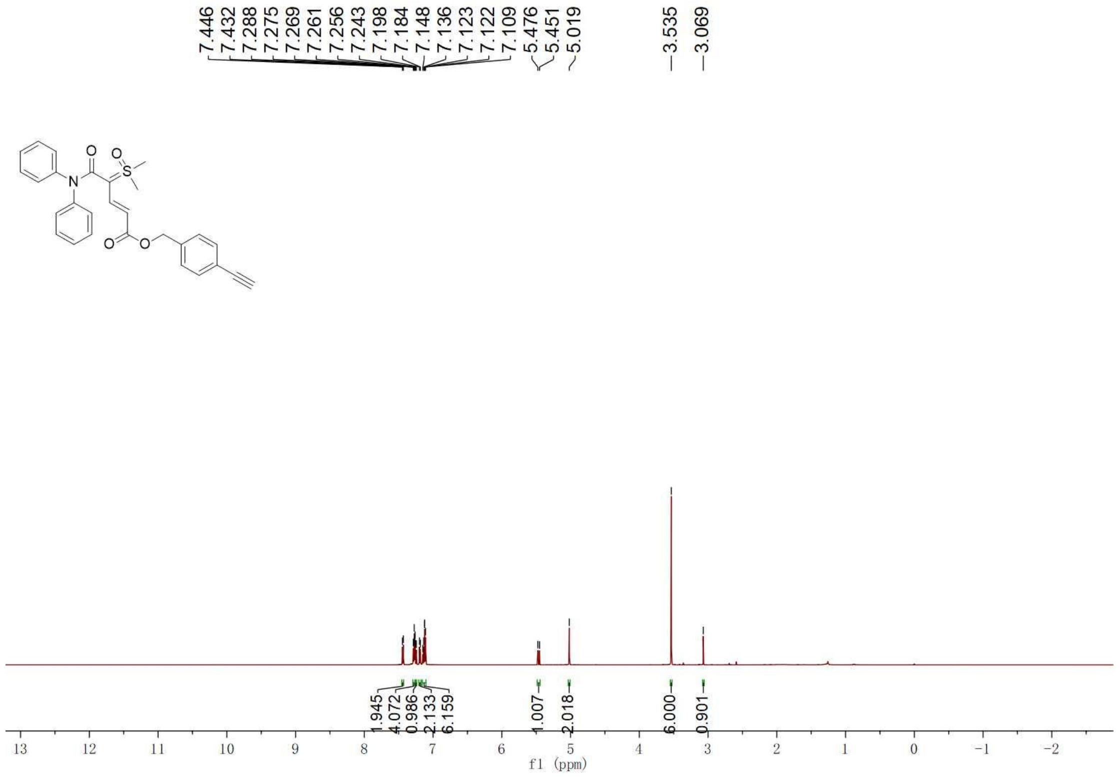

^{13}C NMR (150 MHz, $CDCl_3$) Spectrum of **65**

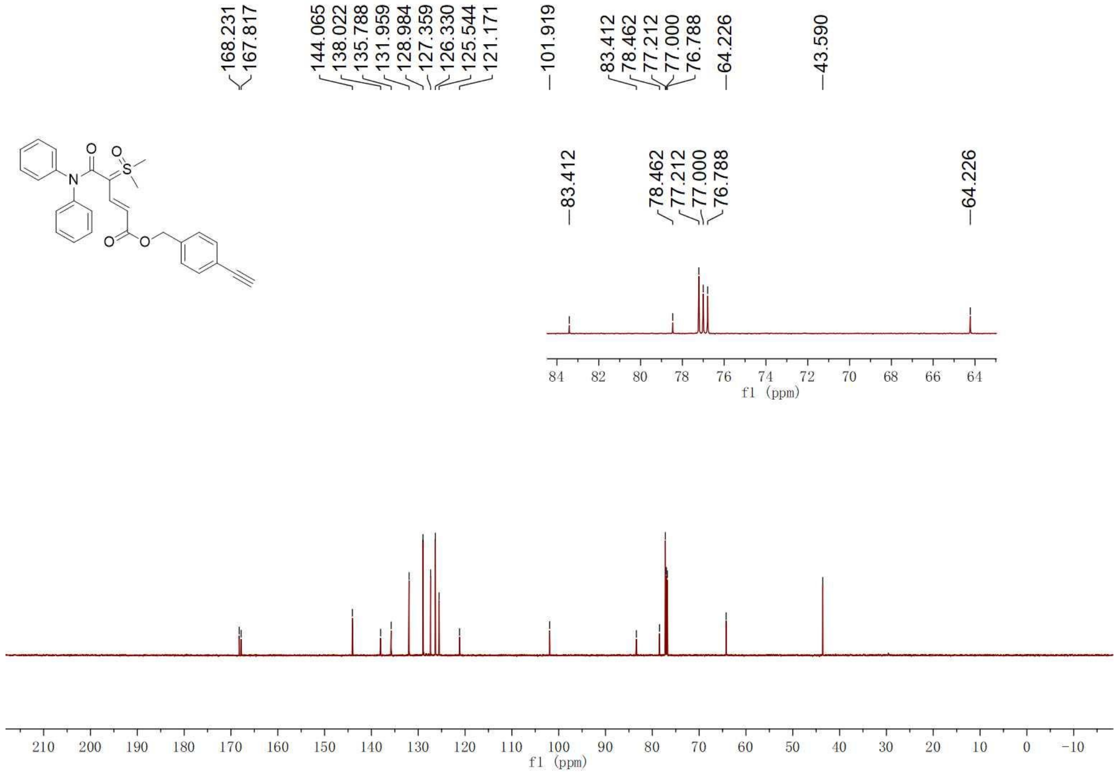

^{1}H NMR (600 MHz, $CDCl_3$) Spectrum of **66**

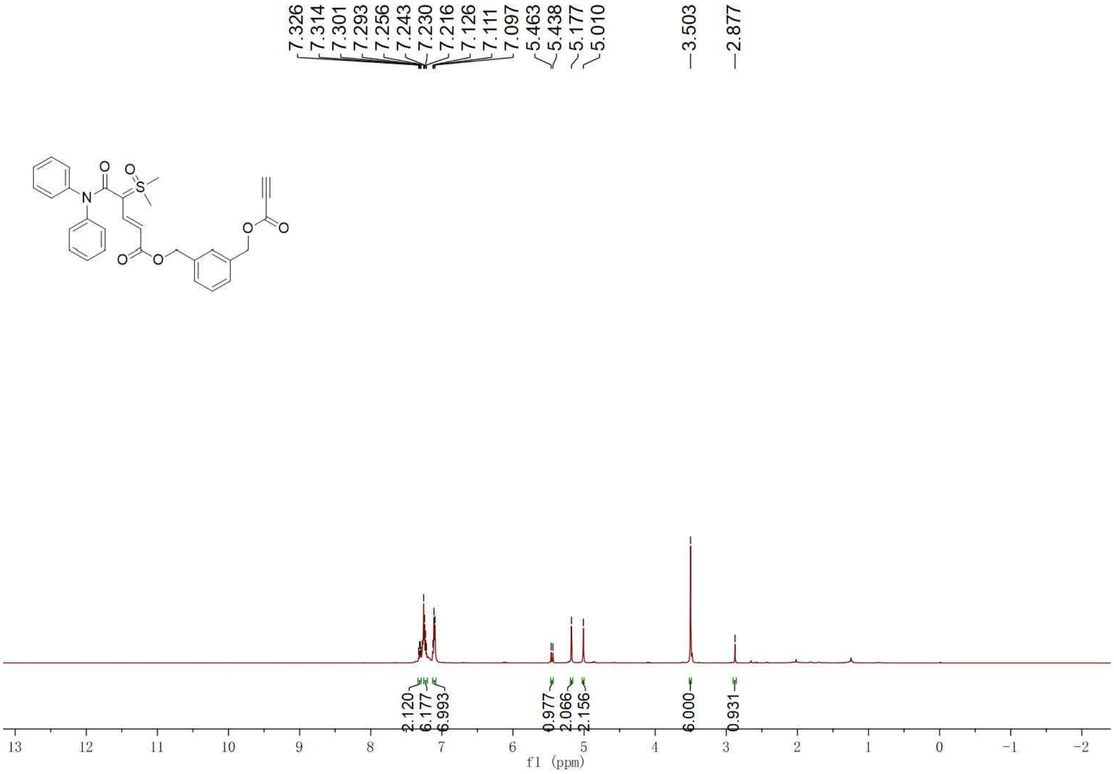

^{13}C NMR (150 MHz, $CDCl_3$) Spectrum of **66**

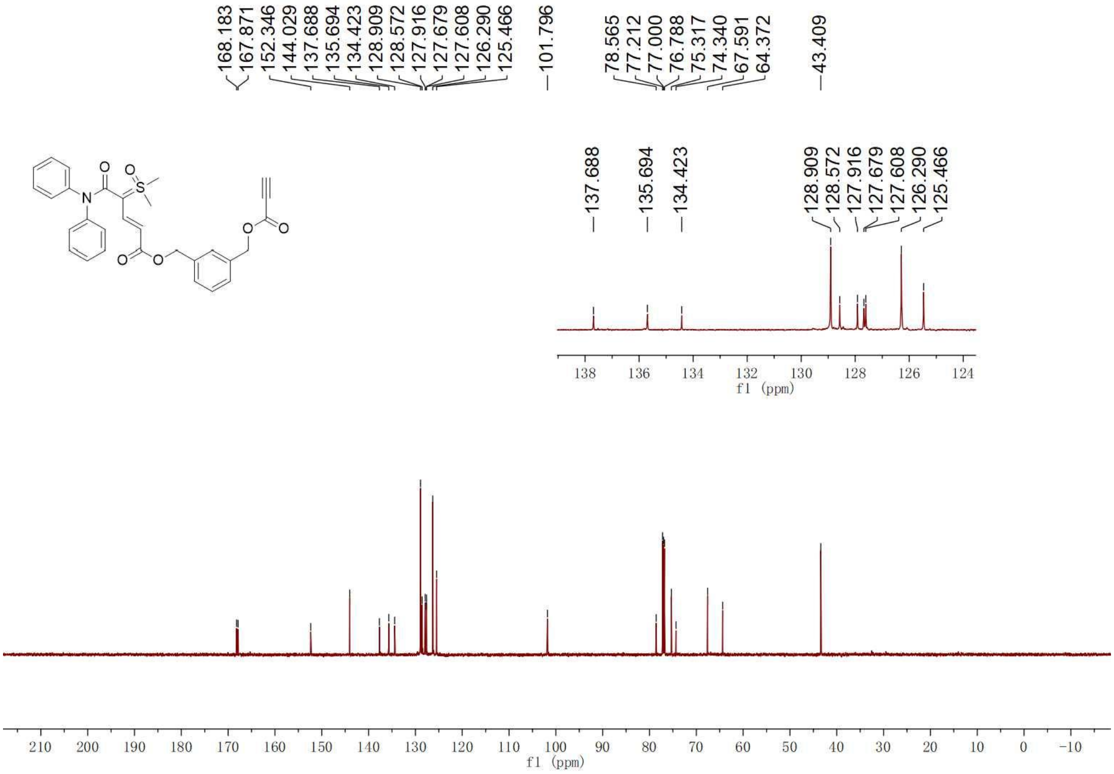

^{1}H NMR (600 MHz, $CDCl_3$) Spectrum of **67**

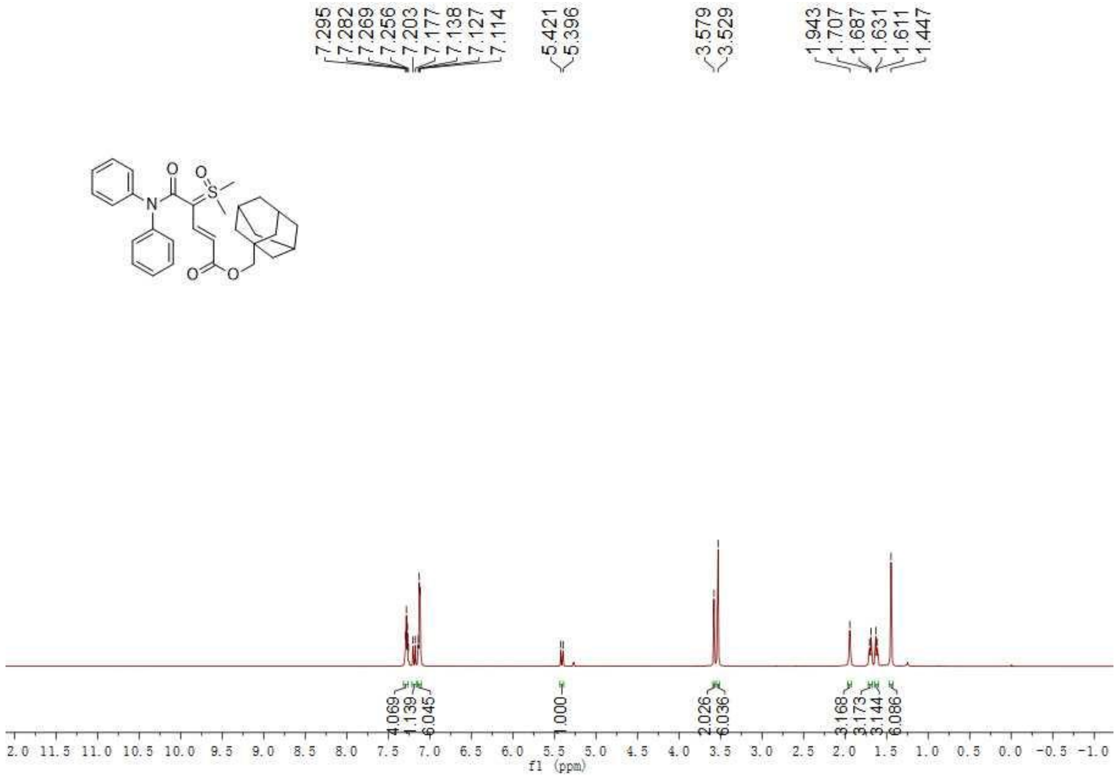

^{13}C NMR (150 MHz, $CDCl_3$) Spectrum of **67**

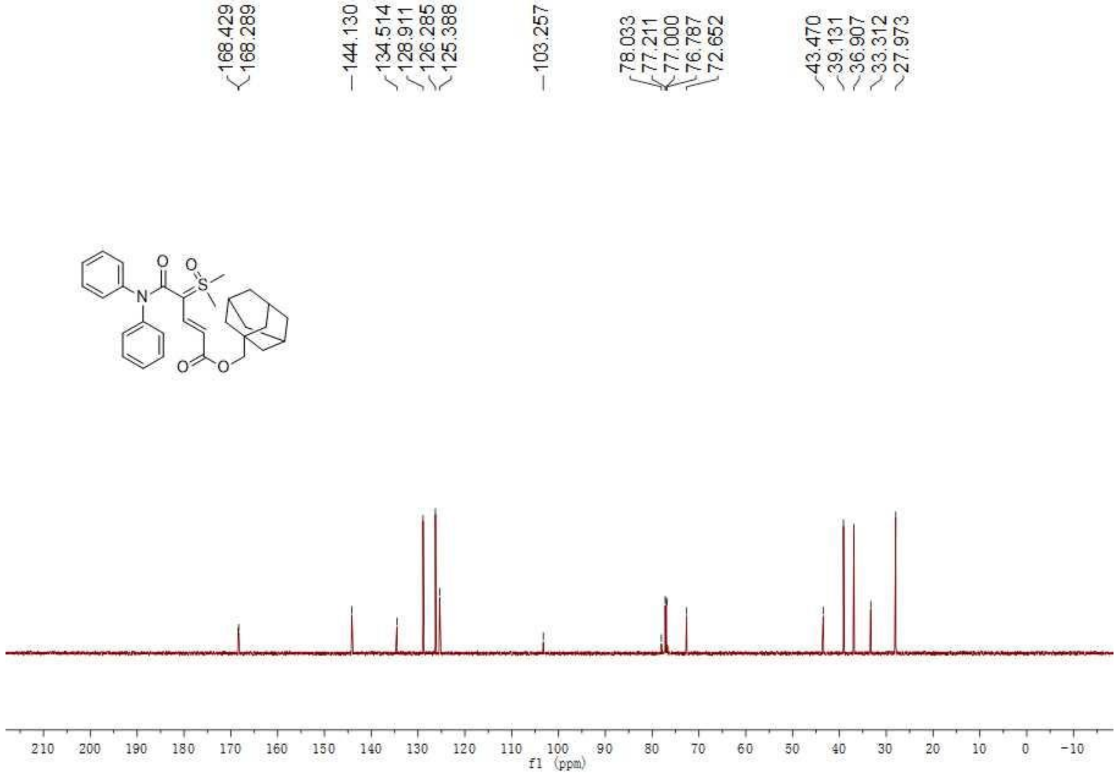

^{1}H NMR (600 MHz, $CDCl_3$) Spectrum of **68**

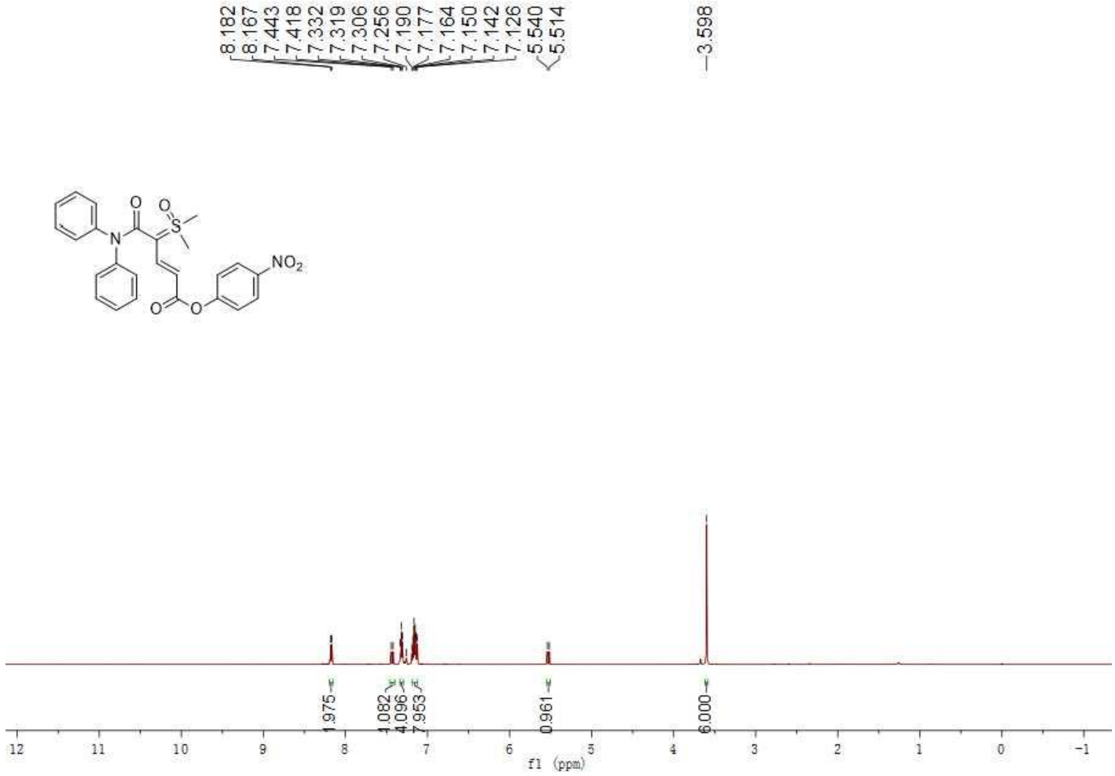

^{13}C NMR (150 MHz, $CDCl_3$) Spectrum of **68**

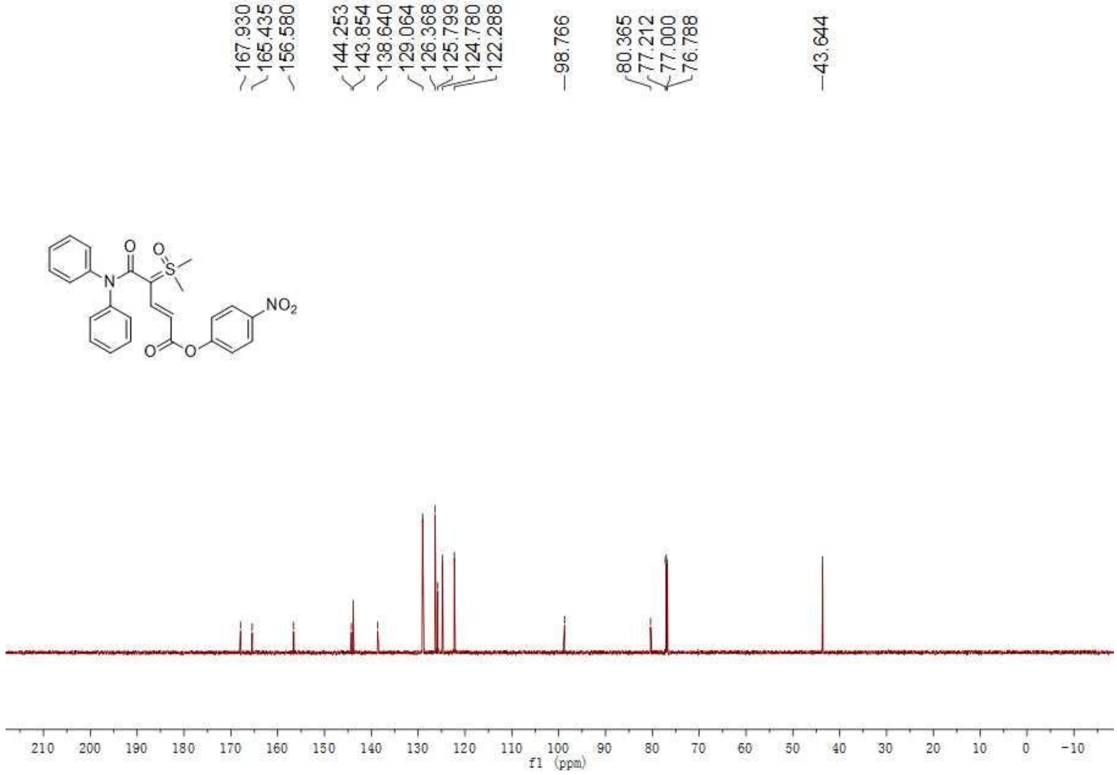

^{1}H NMR (600 MHz, $CDCl_3$) Spectrum of **69**

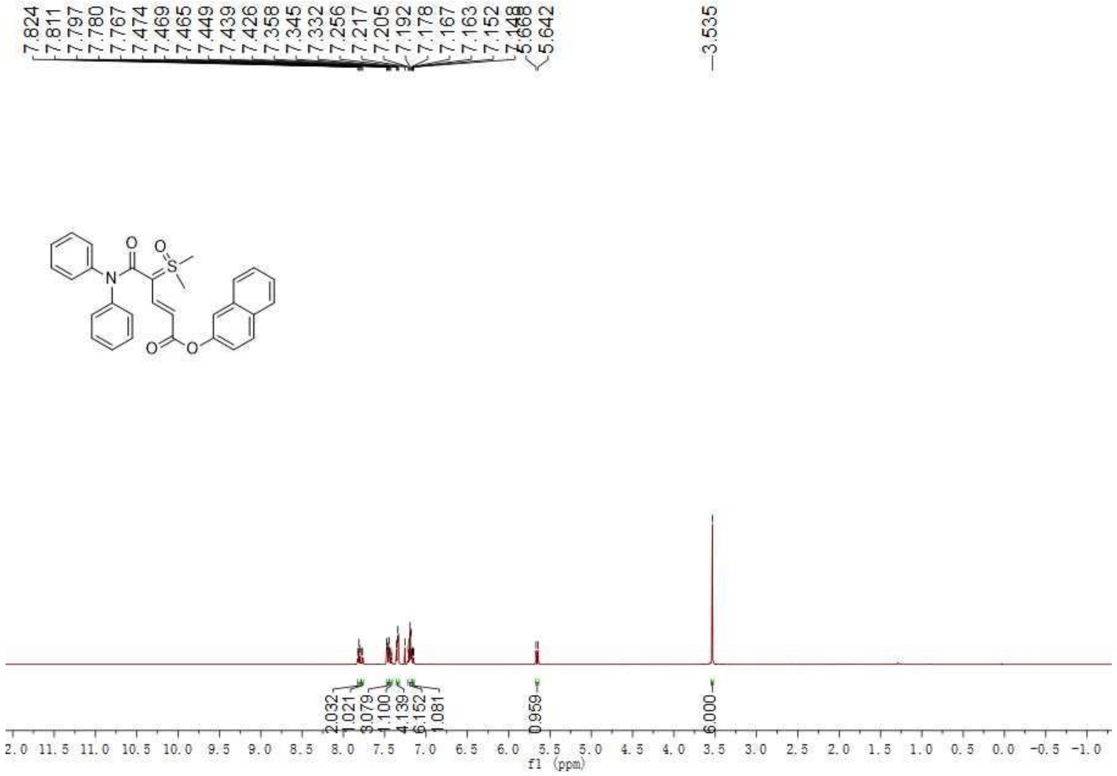

^{13}C NMR (150 MHz, $CDCl_3$) Spectrum of **69**

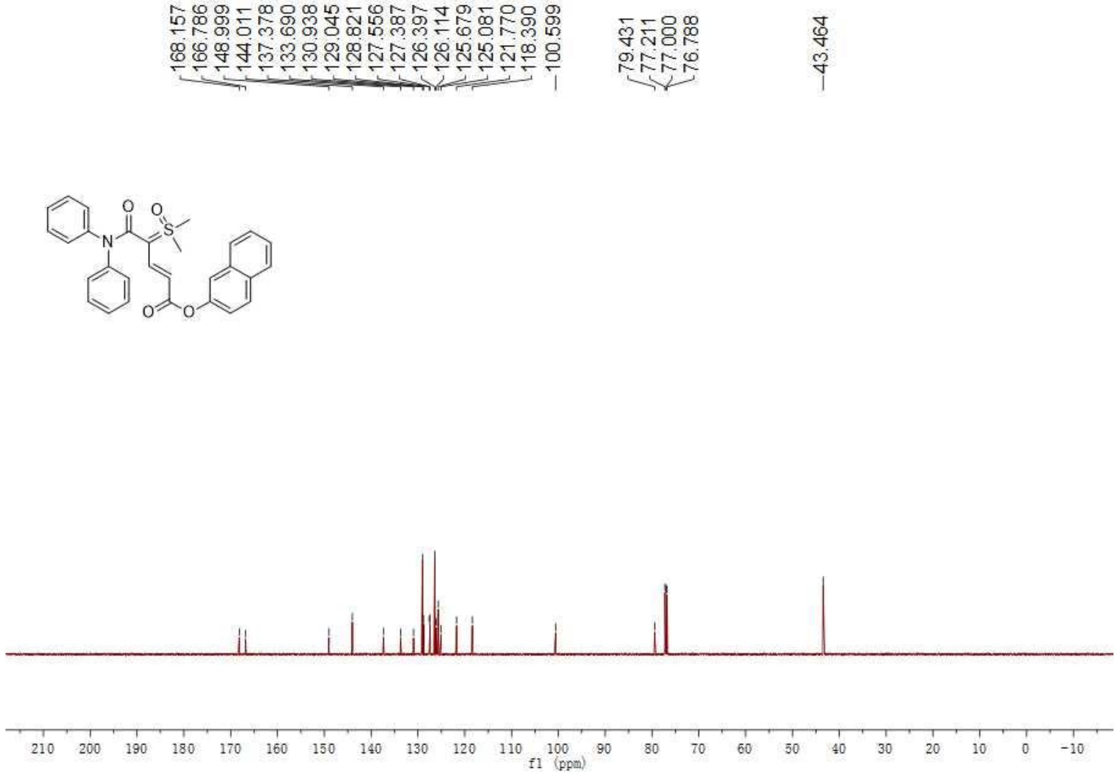

^{1}H NMR (600 MHz, $CDCl_3$) Spectrum of **70**

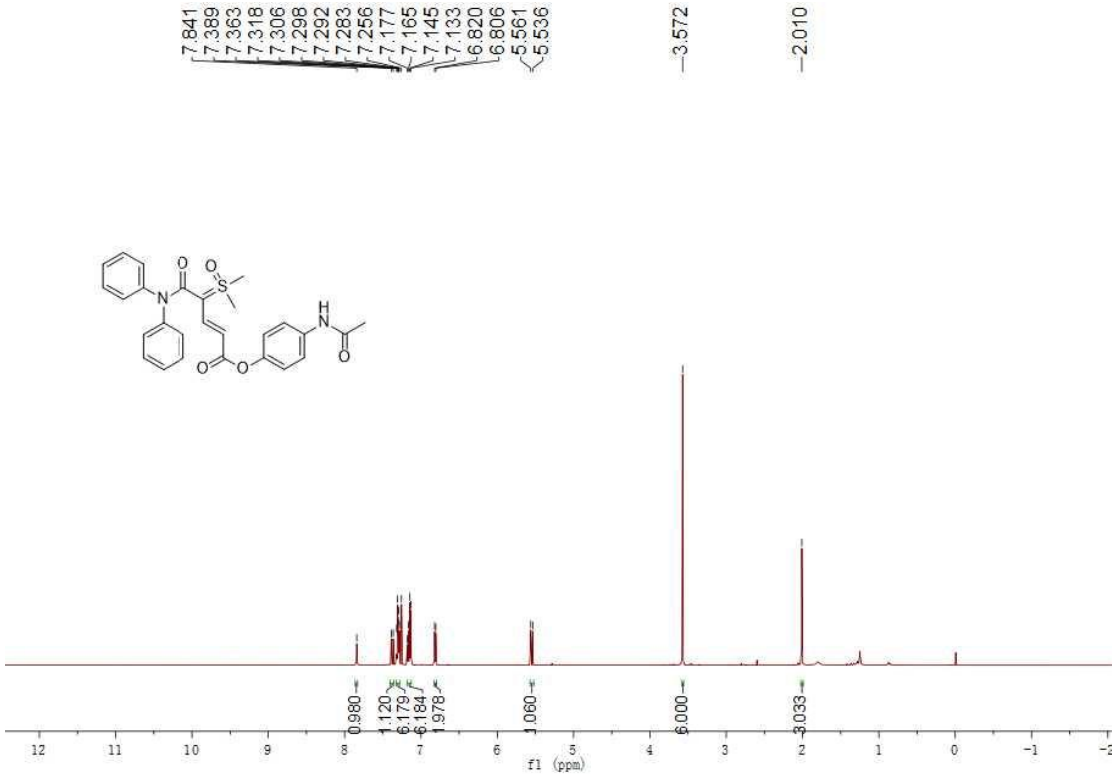

^{13}C NMR (150 MHz, $CDCl_3$) Spectrum of **70**

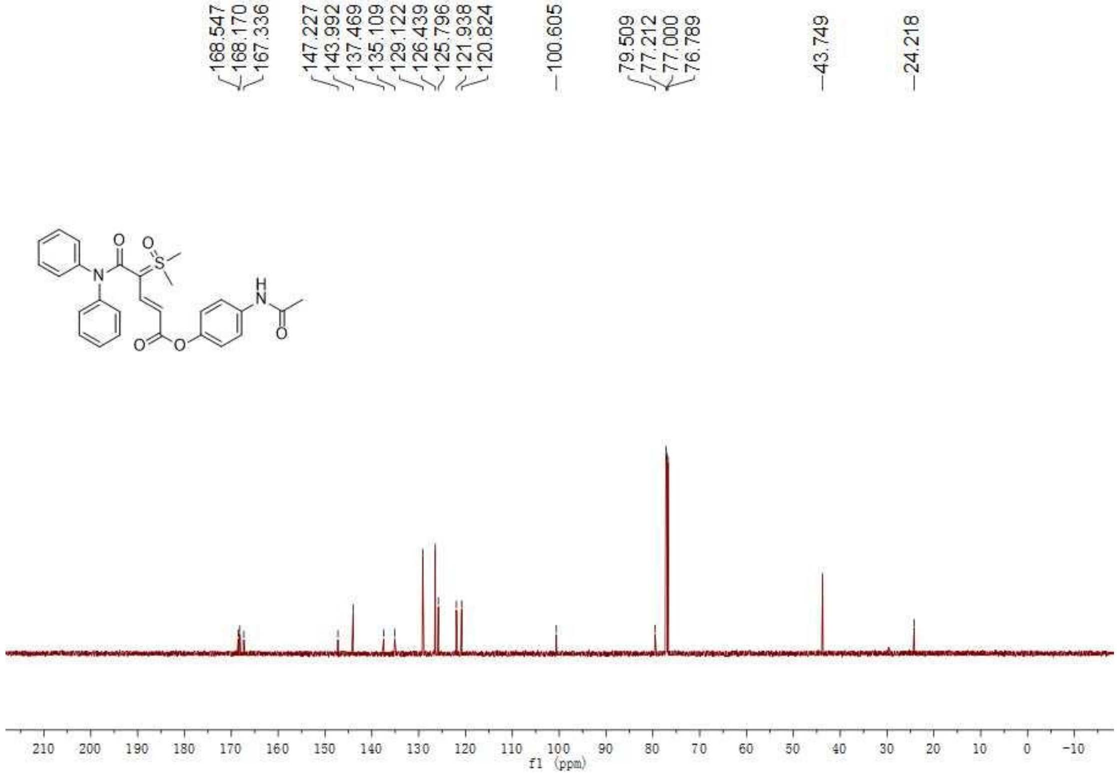

^{1}H NMR (600 MHz, $CDCl_3$) Spectrum of **71**

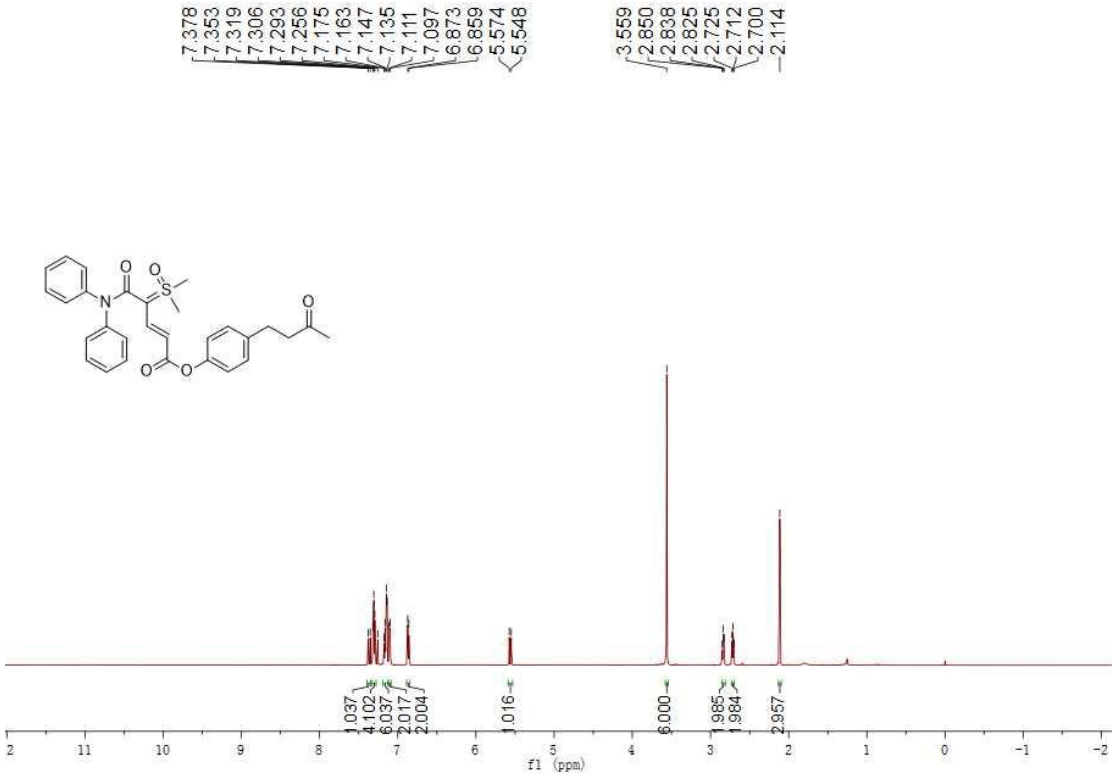

^{13}C NMR (150 MHz, $CDCl_3$) Spectrum of **71**

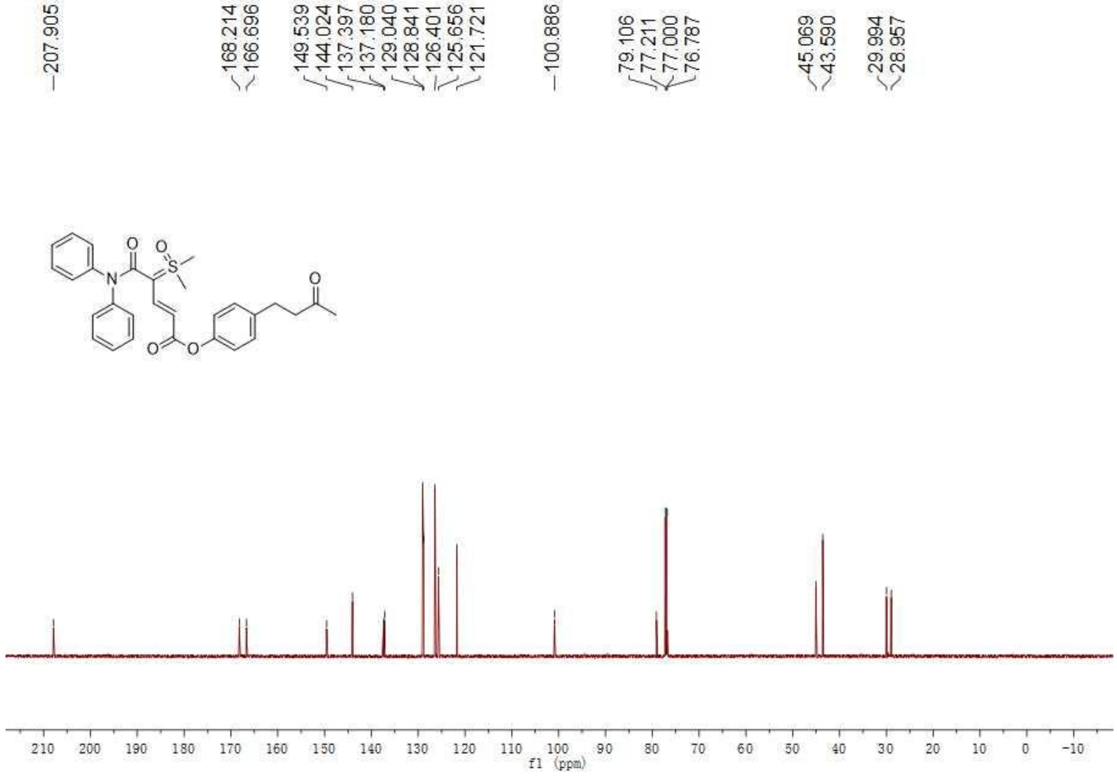

^{1}H NMR (600 MHz, CDCl$_3$) Spectrum of **72**

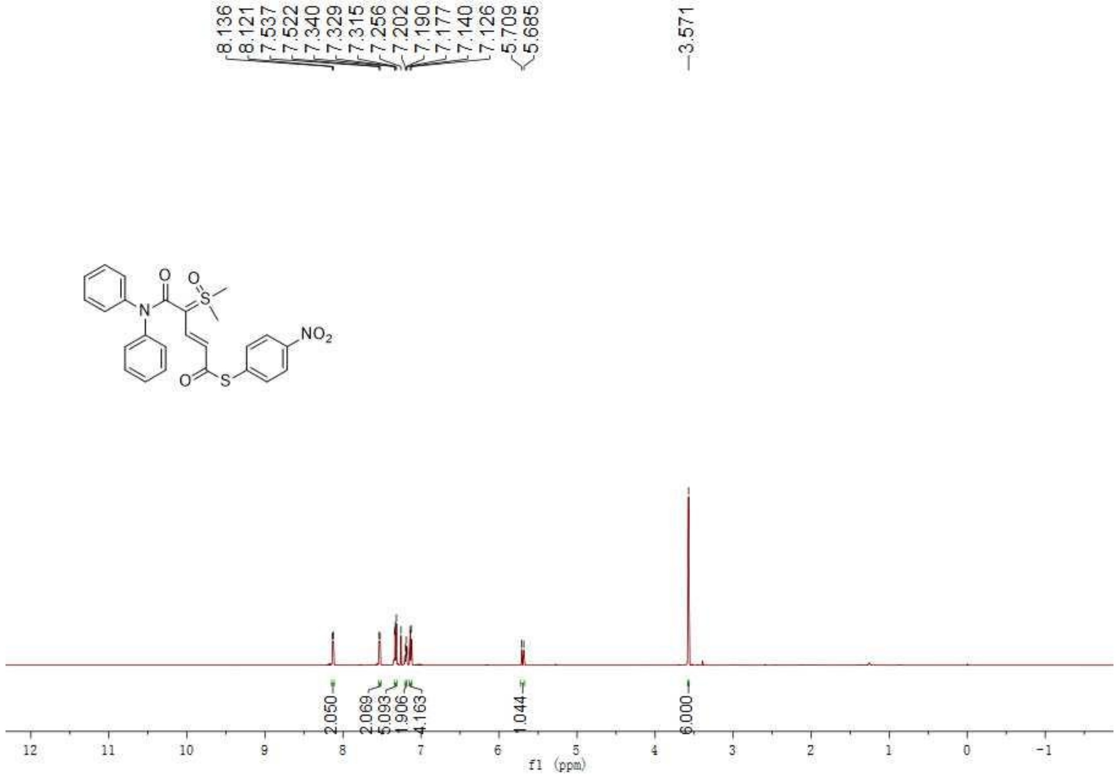

^{13}C NMR (150 MHz, CDCl$_3$) Spectrum of **72**

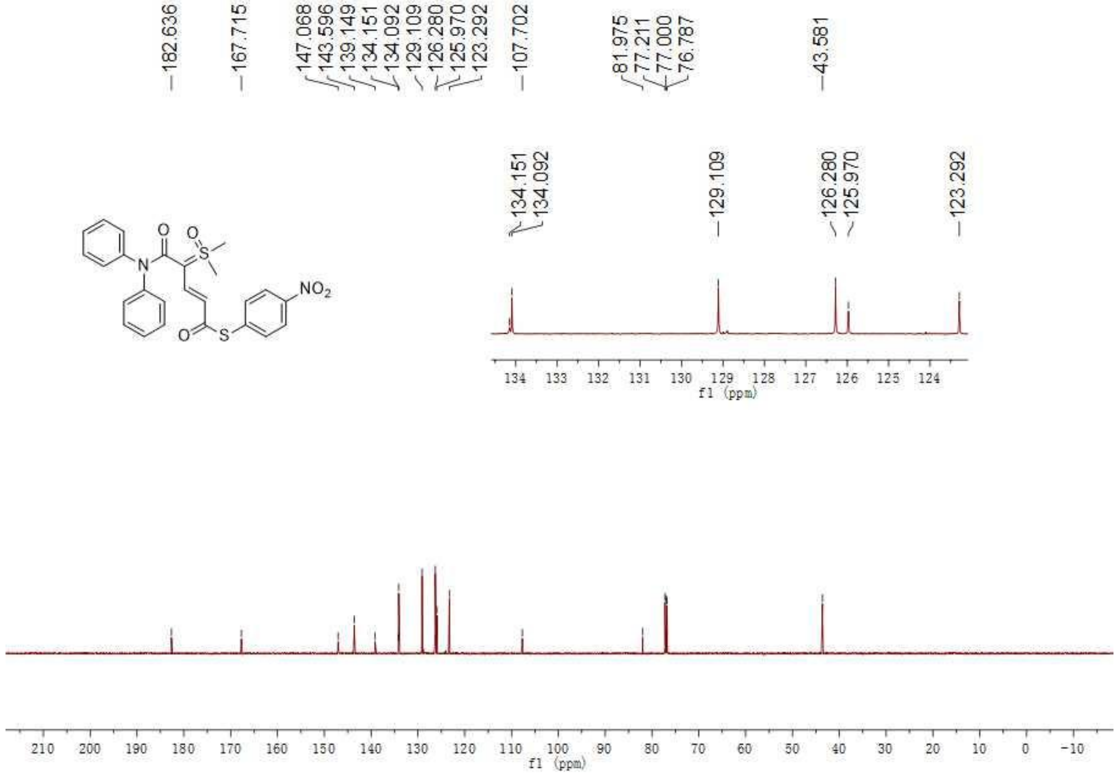

^{1}H NMR (600 MHz, $CDCl_3$) Spectrum of **73**

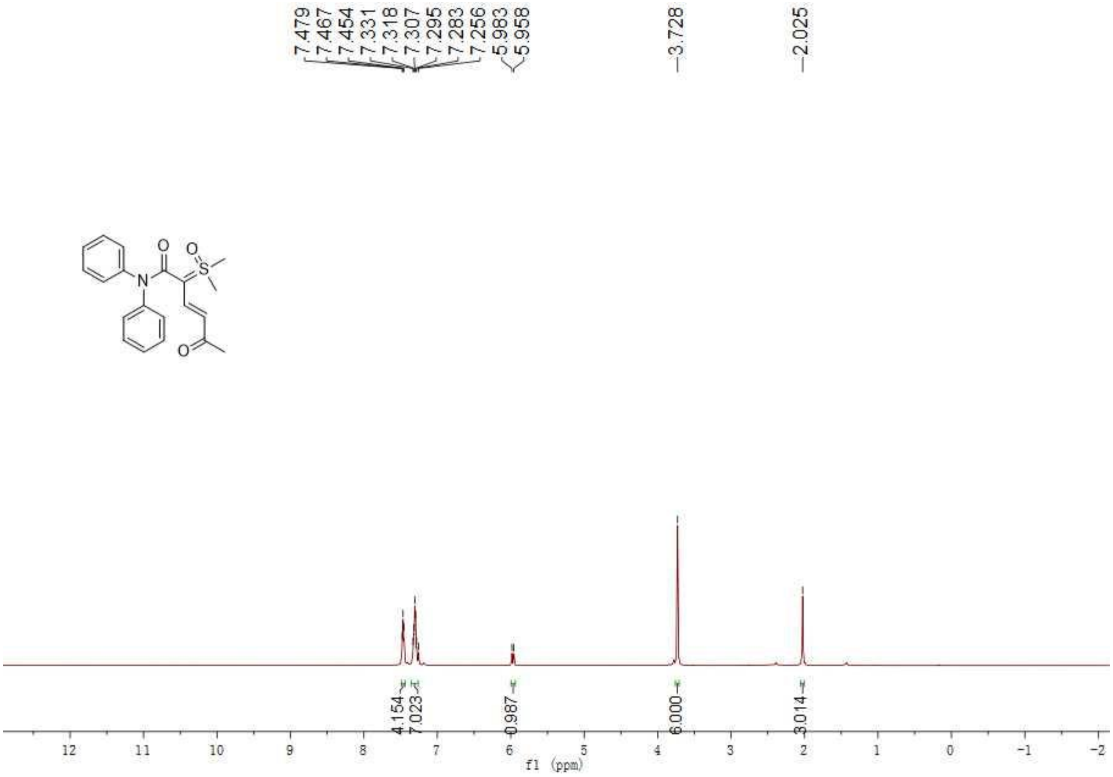

^{13}C NMR (150 MHz, $CDCl_3$) Spectrum of **73**

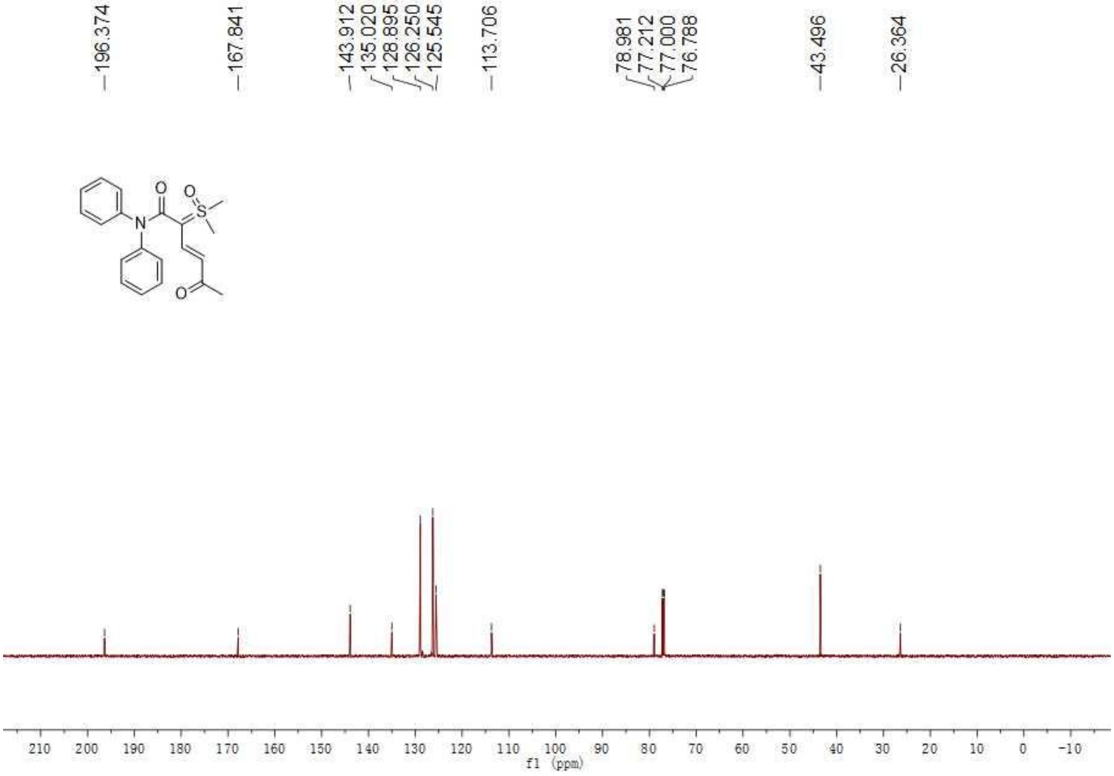

^{1}H NMR (600 MHz, $CDCl_3$) Spectrum of **74**

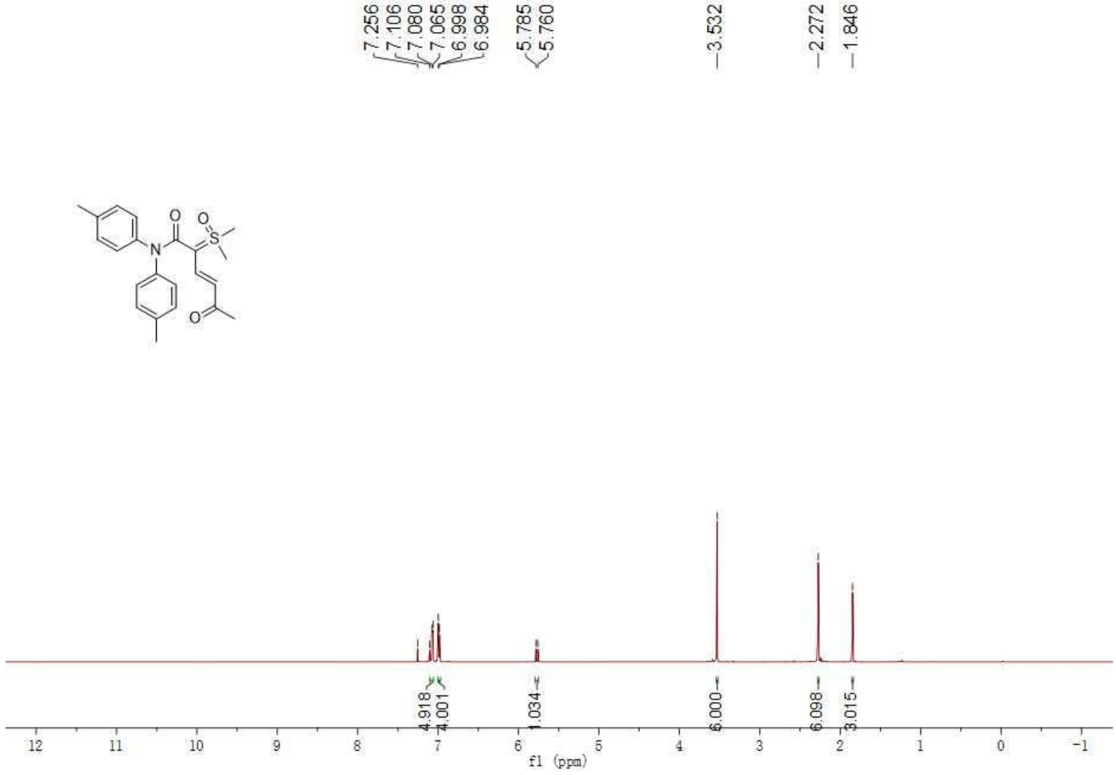

^{13}C NMR (150 MHz, $CDCl_3$) Spectrum of **74**

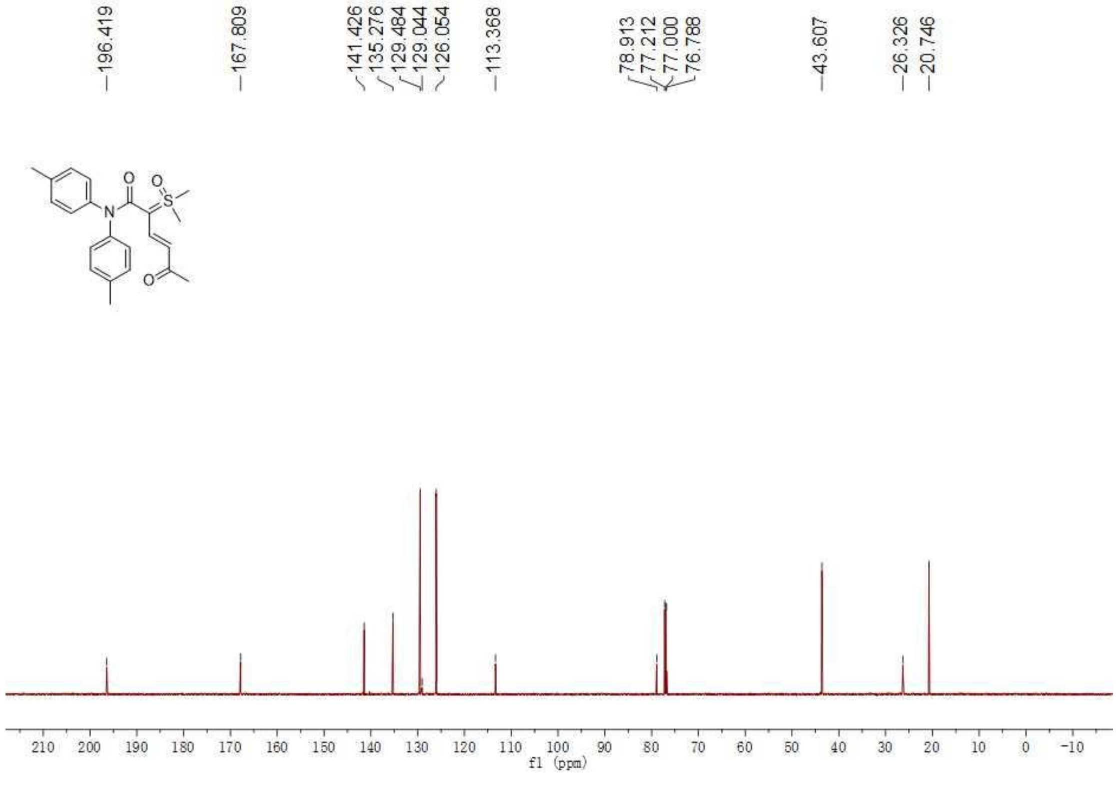

^{1}H NMR (600 MHz, $CDCl_3$) Spectrum of **75**

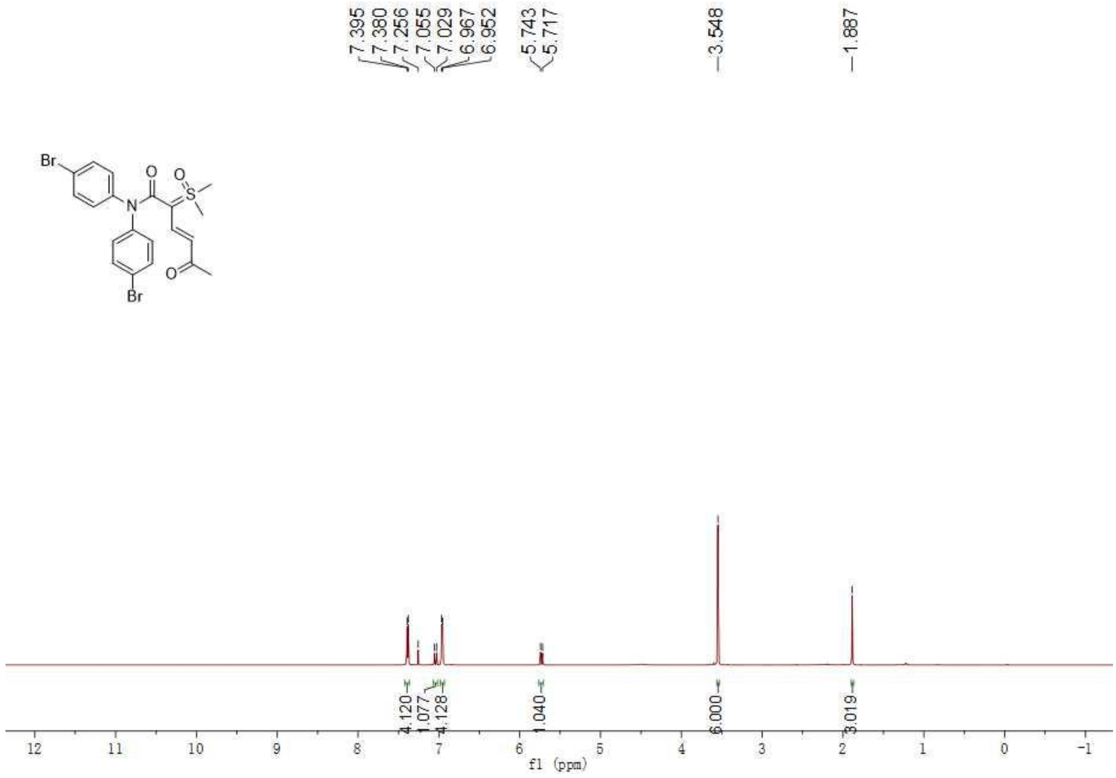

^{13}C NMR (150 MHz, $CDCl_3$) Spectrum of **75**

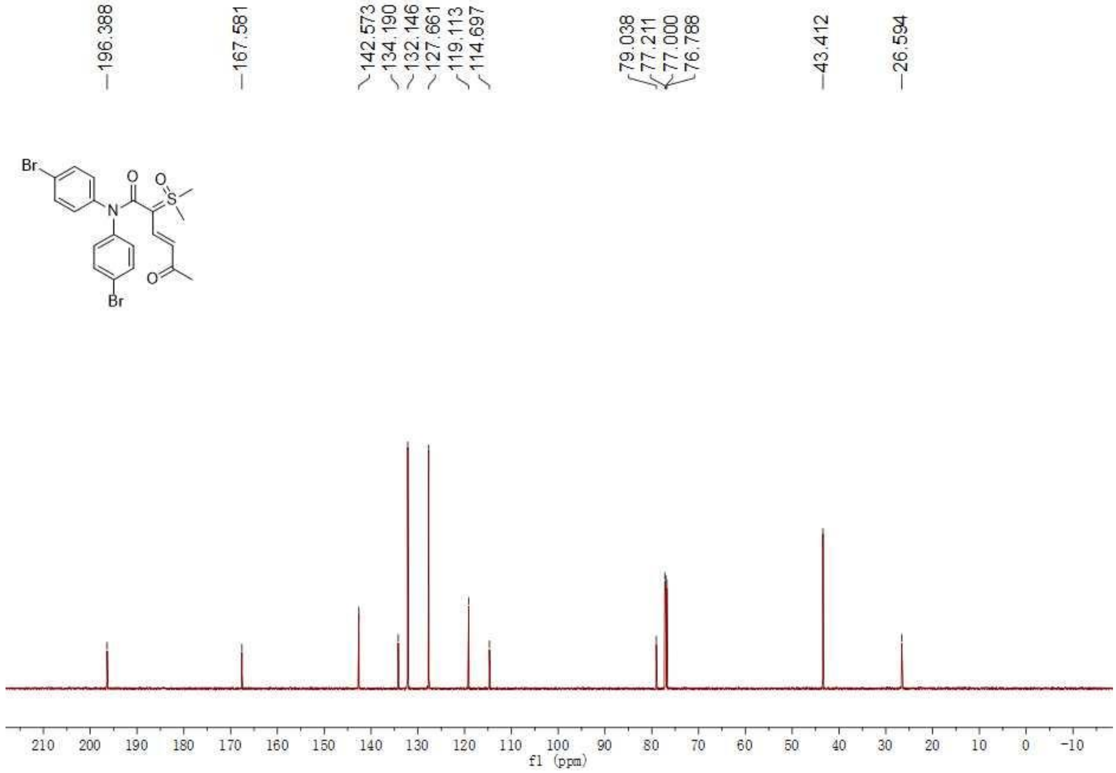

^{1}H NMR (600 MHz, $CDCl_3$) Spectrum of **76**

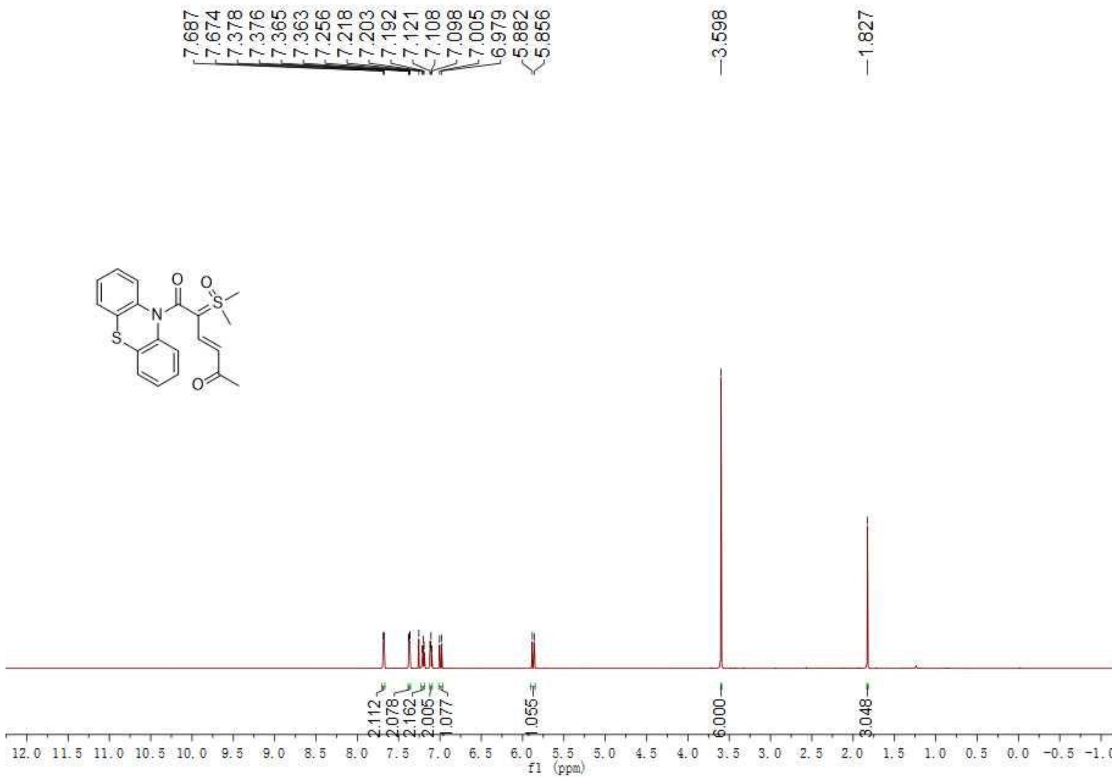

^{13}C NMR (150 MHz, $CDCl_3$) Spectrum of **76**

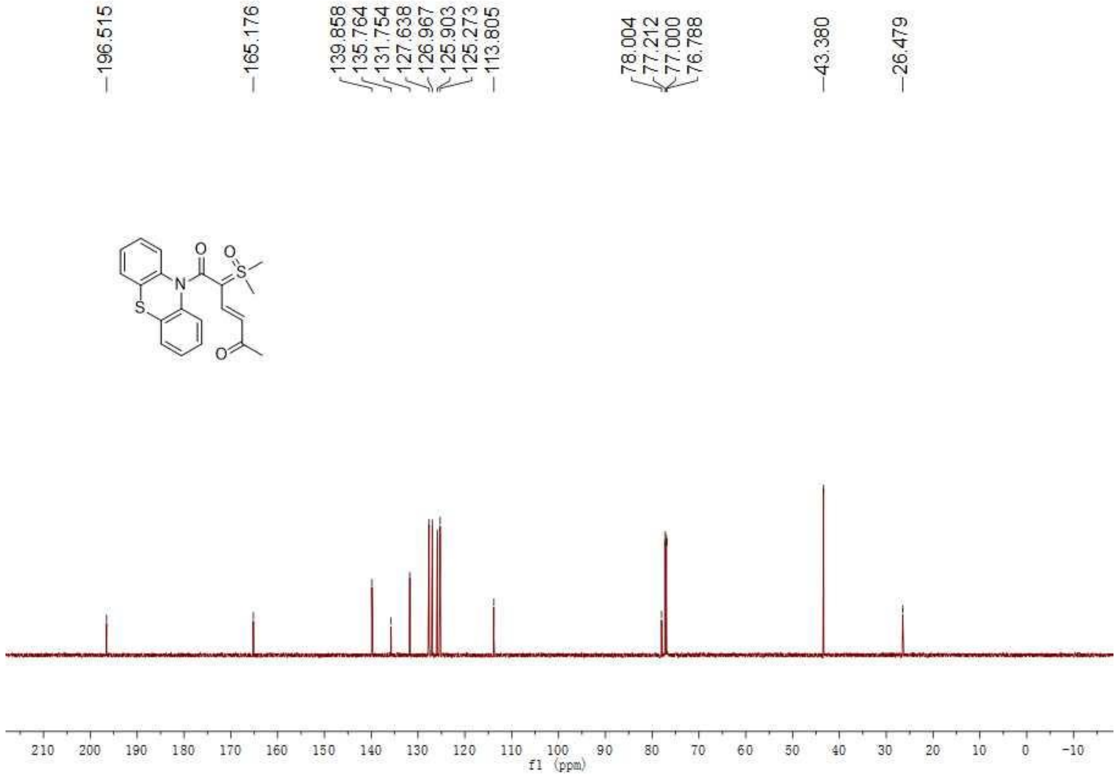

^{1}H NMR (600 MHz, $CDCl_3$) Spectrum of **77**

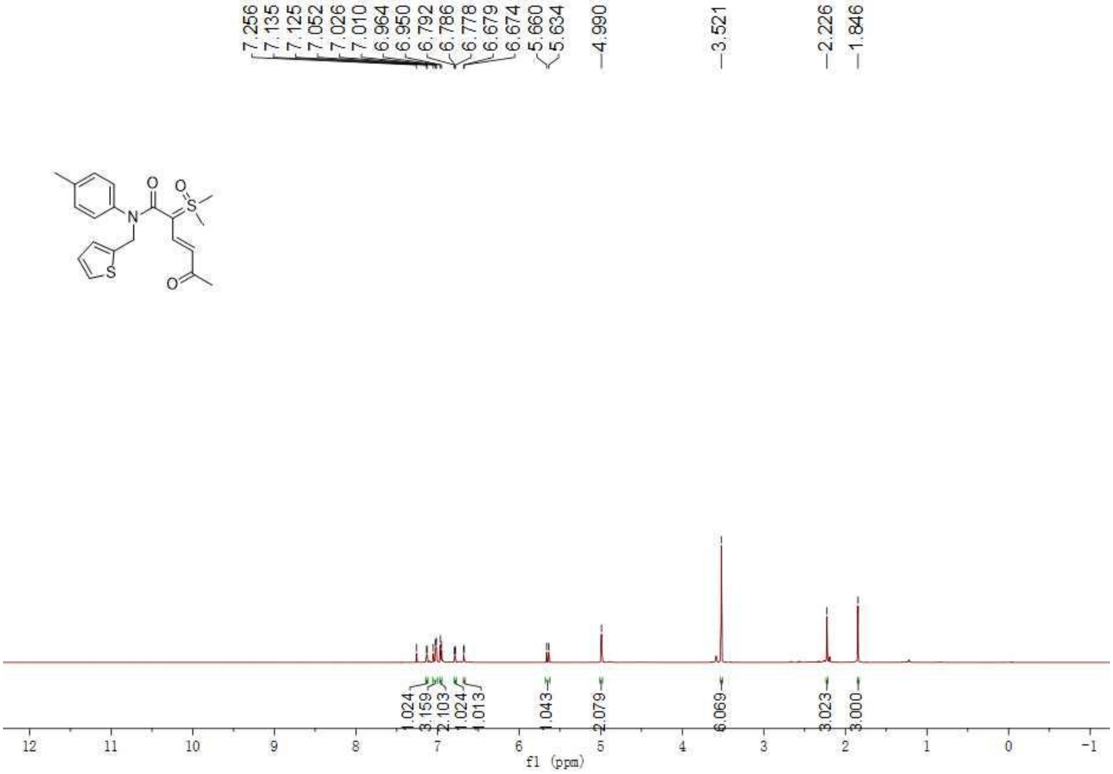

^{13}C NMR (150 MHz, $CDCl_3$) Spectrum of **77**

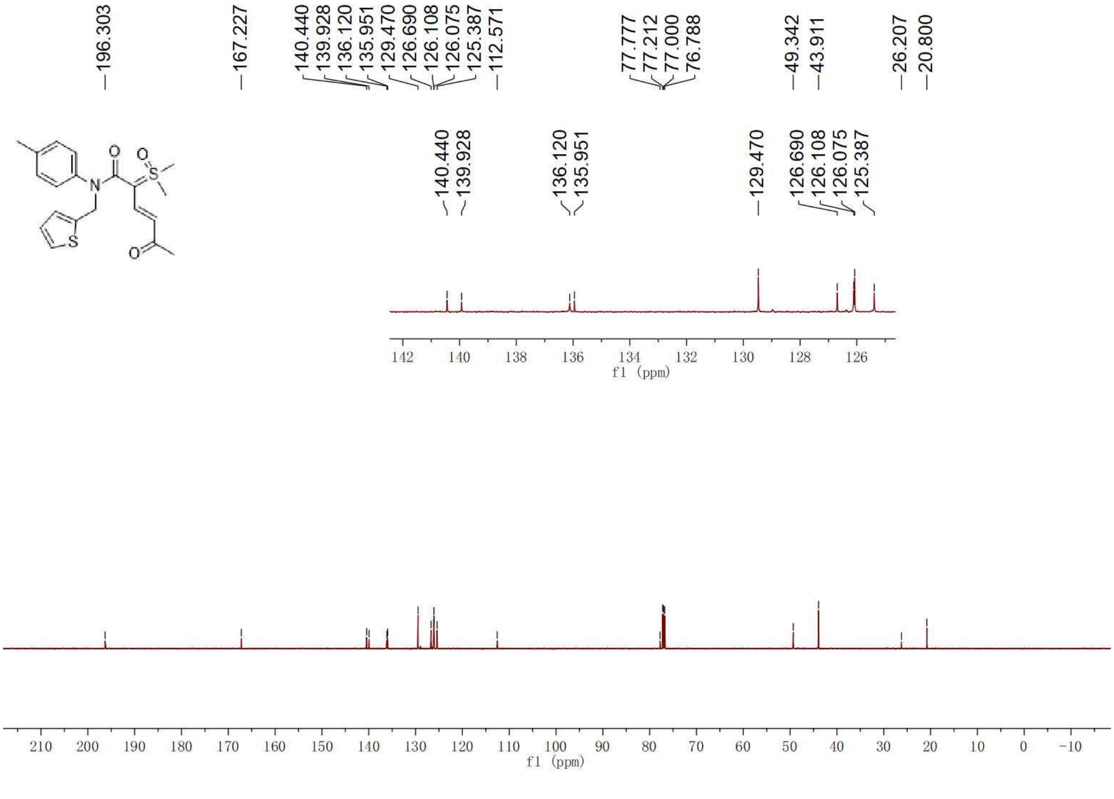

^{1}H NMR (600 MHz, $CDCl_3$) Spectrum of **78**

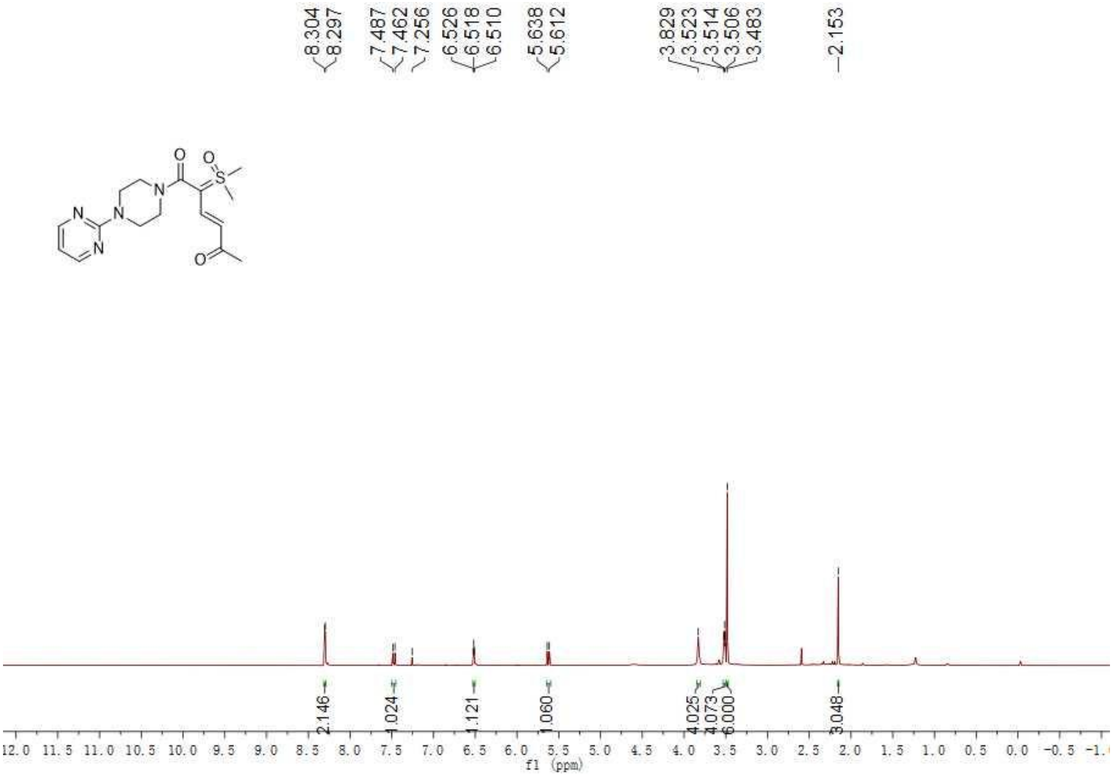

^{13}C NMR (150 MHz, $CDCl_3$) Spectrum of **78**

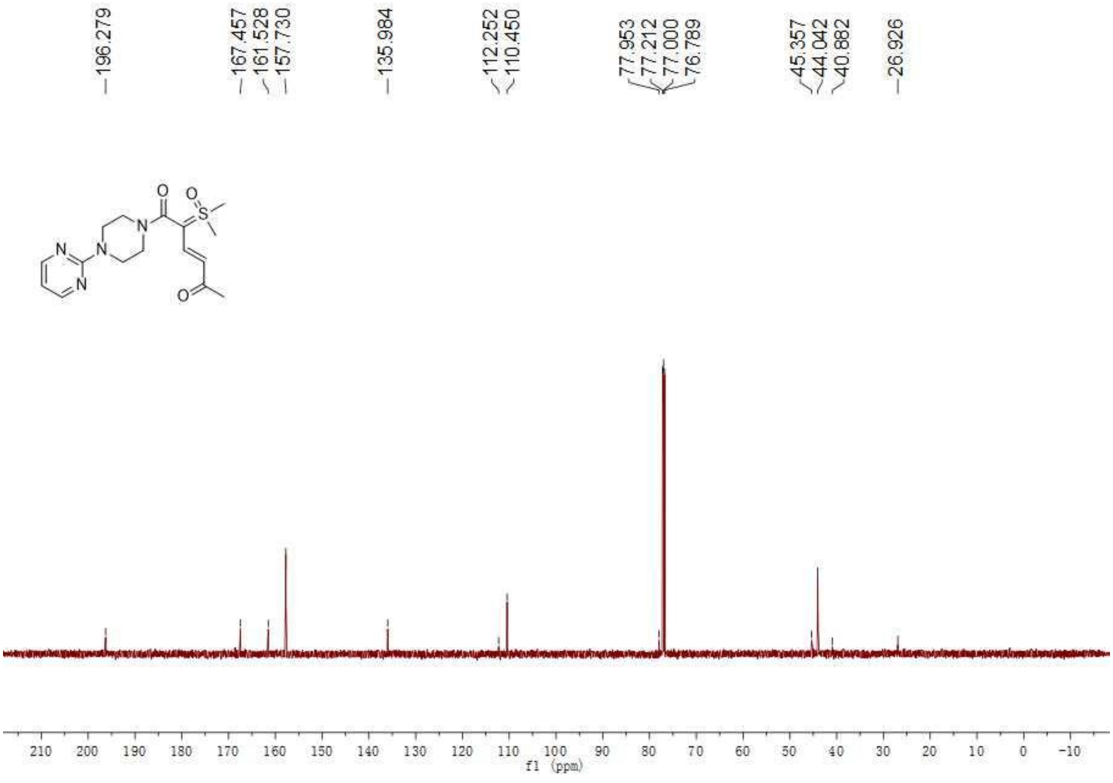

^{1}H NMR (600 MHz, $CDCl_3$) Spectrum of **79**

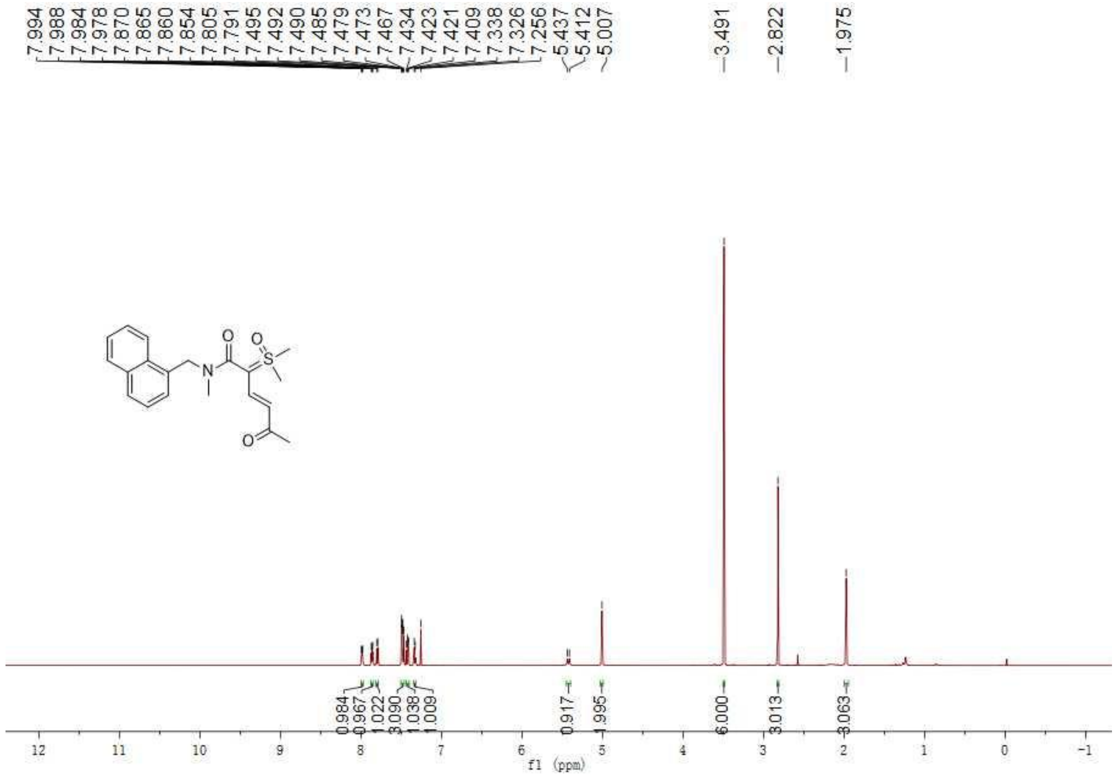

^{13}C NMR (150 MHz, $CDCl_3$) Spectrum of **79**

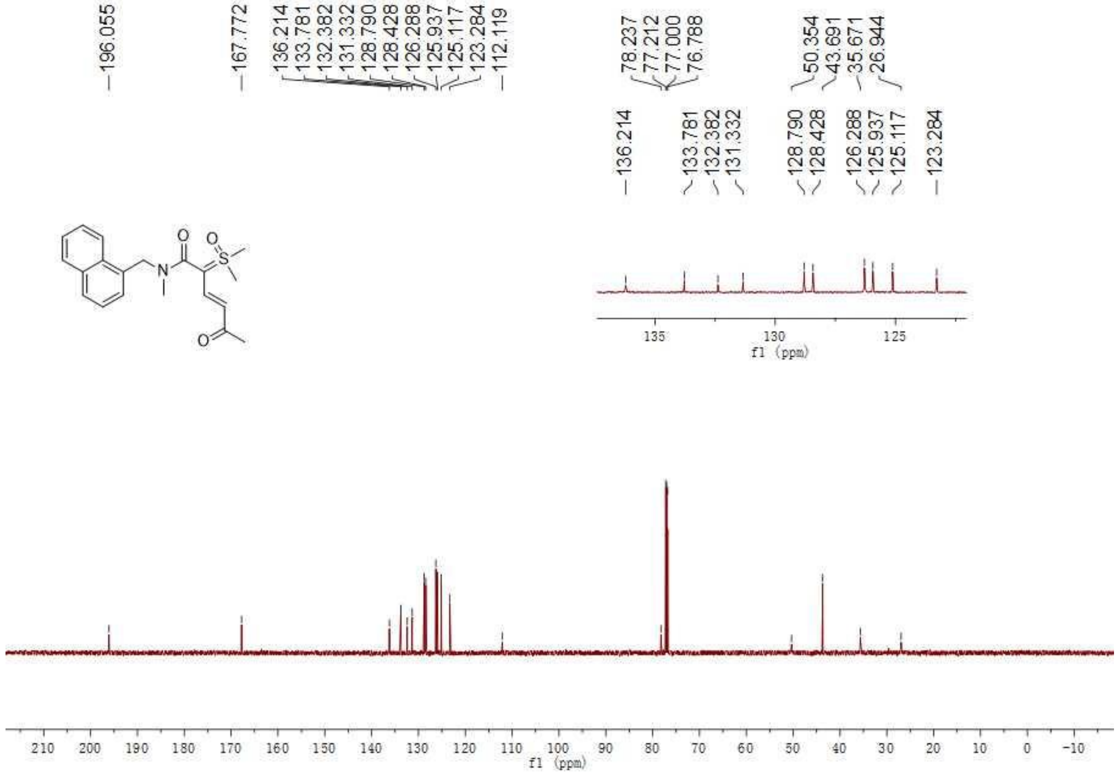

^{1}H NMR (600 MHz, $CDCl_3$) Spectrum of **80**

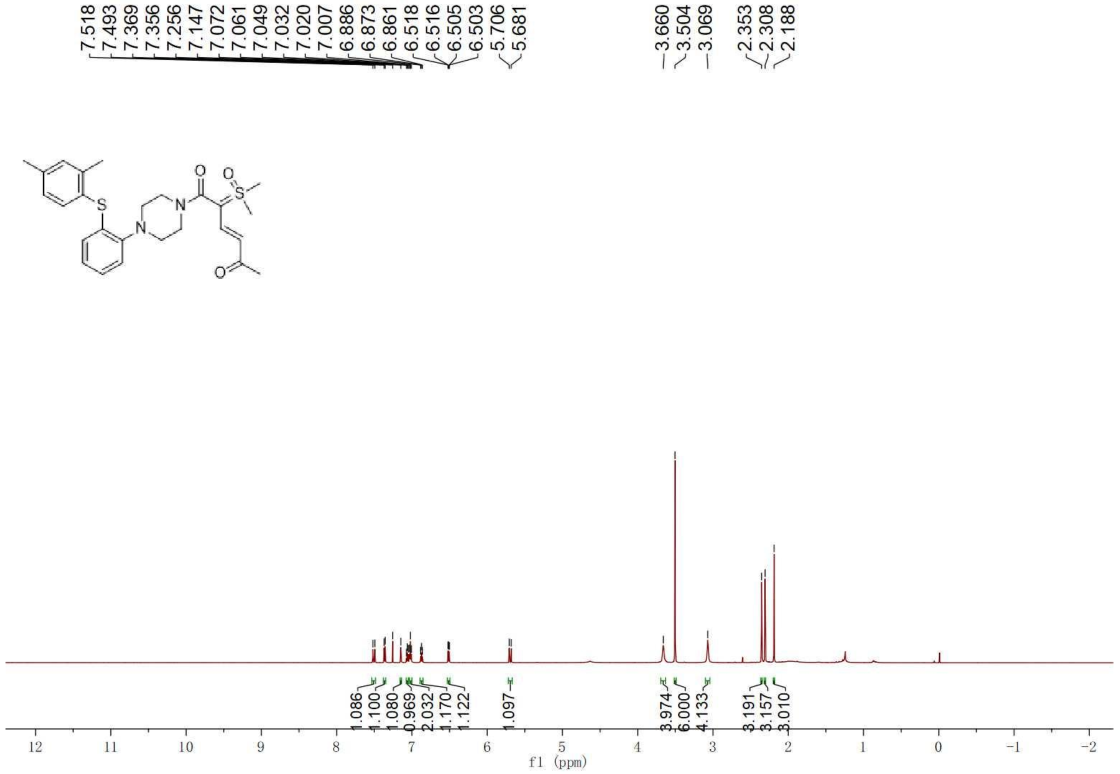

^{13}C NMR (150 MHz, $CDCl_3$) Spectrum of **80**

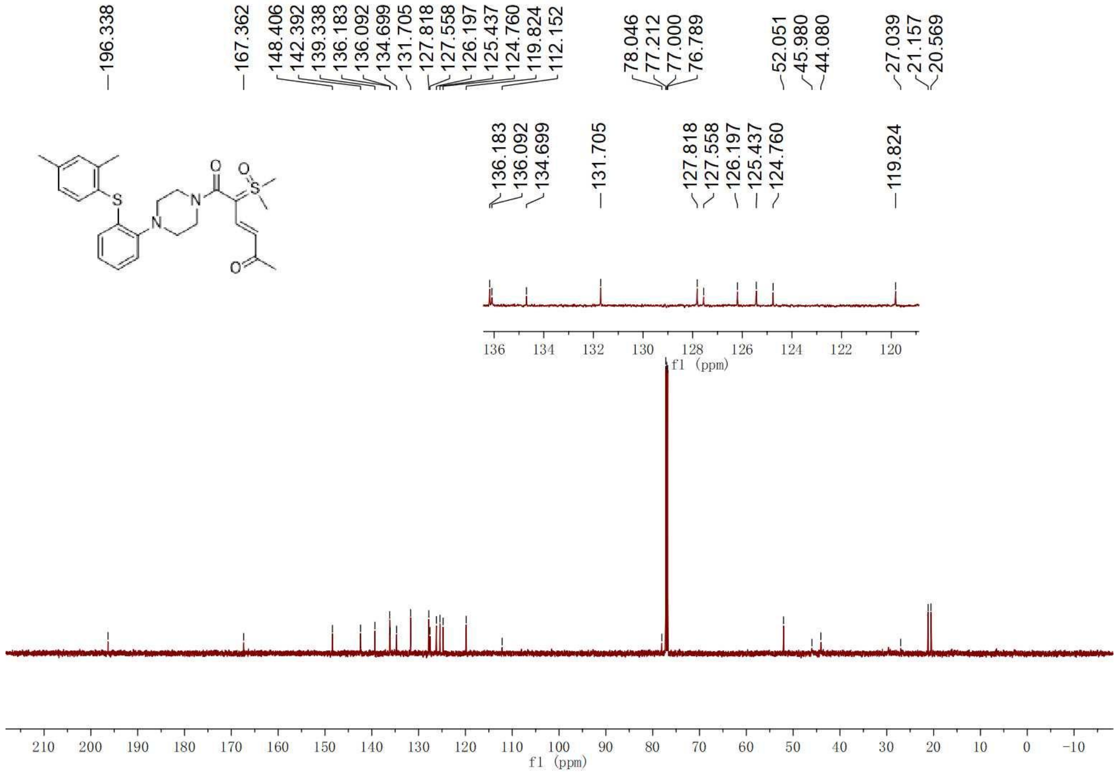

^{1}H NMR (600 MHz, $CDCl_3$) Spectrum of **81**

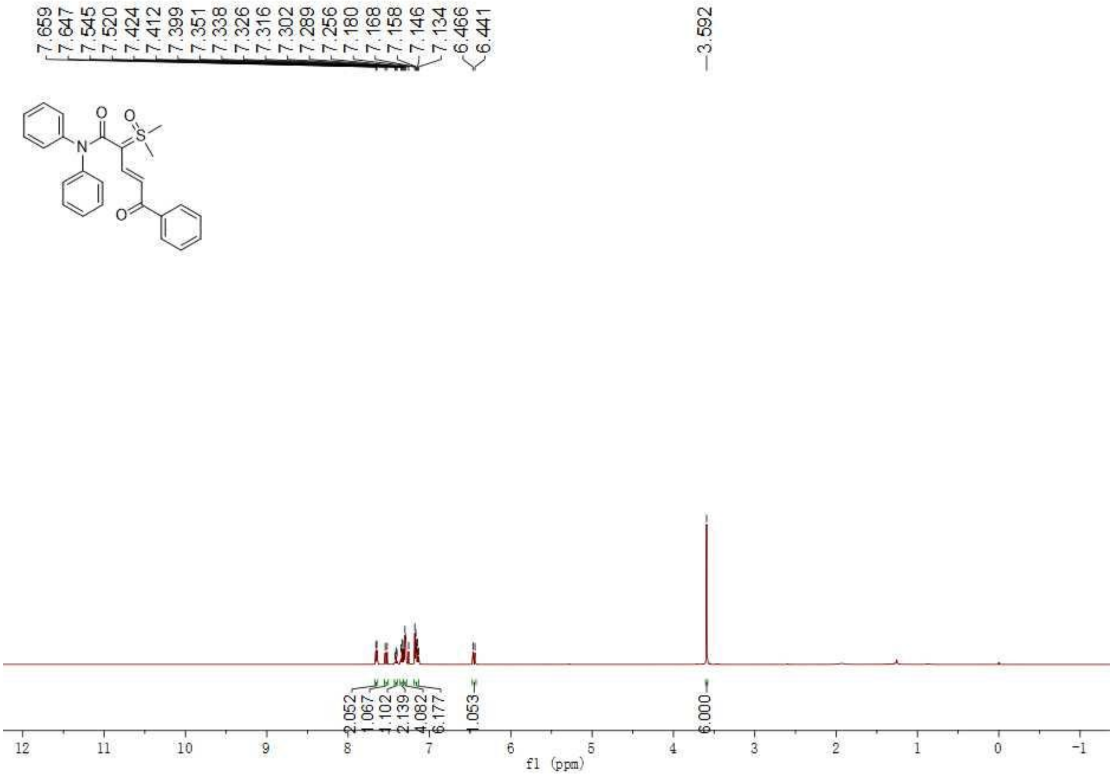

^{13}C NMR (150 MHz, $CDCl_3$) Spectrum of **81**

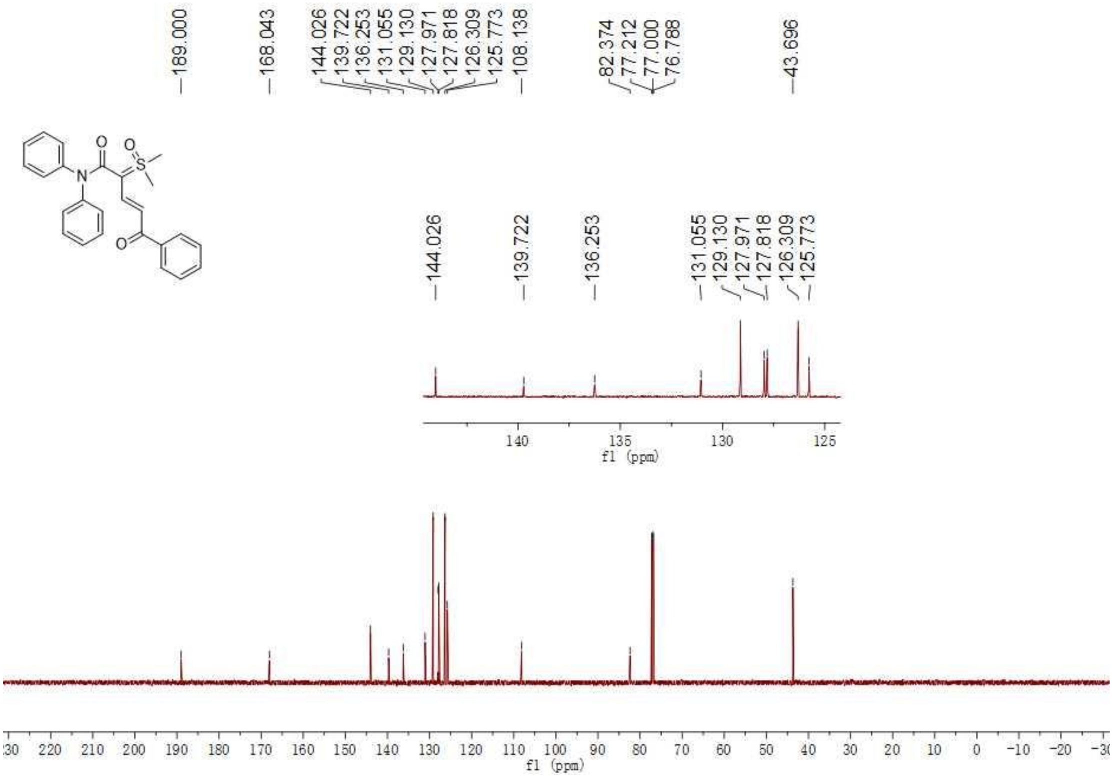

^{1}H NMR (600 MHz, $CDCl_3$) Spectrum of **83**

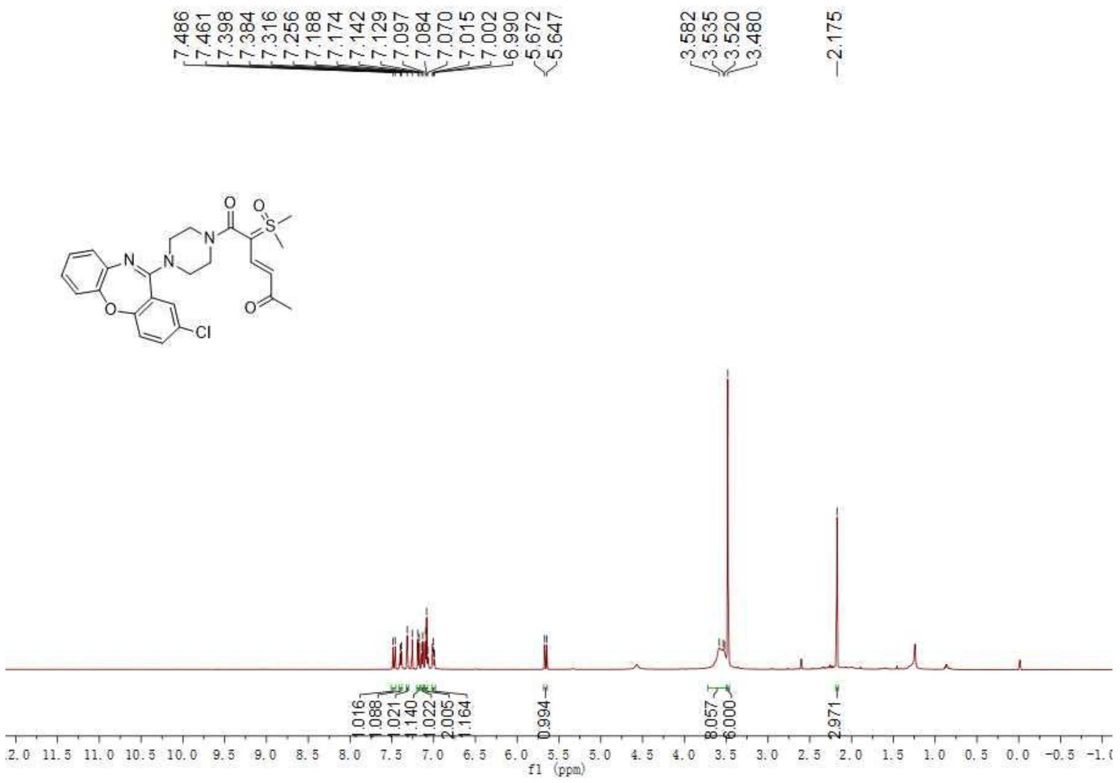

^{13}C NMR (150 MHz, $CDCl_3$) Spectrum of **83**

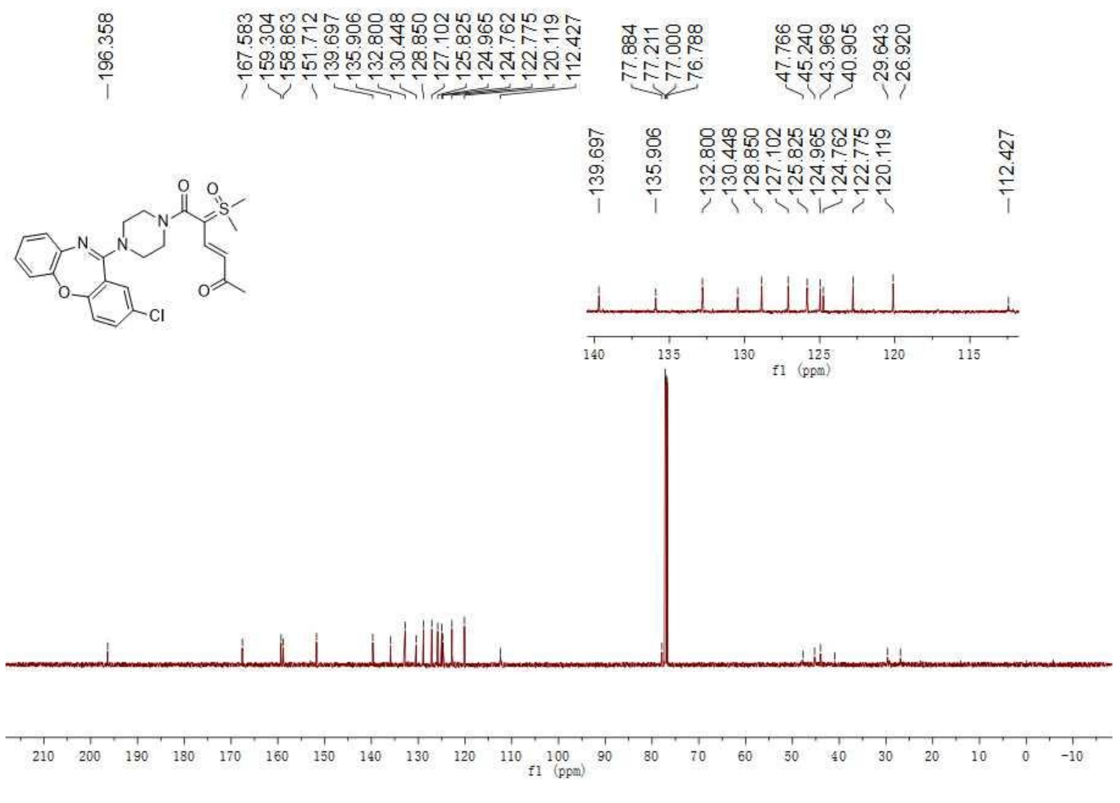

^{1}H NMR (600 MHz, $CDCl_3$) Spectrum of **83**

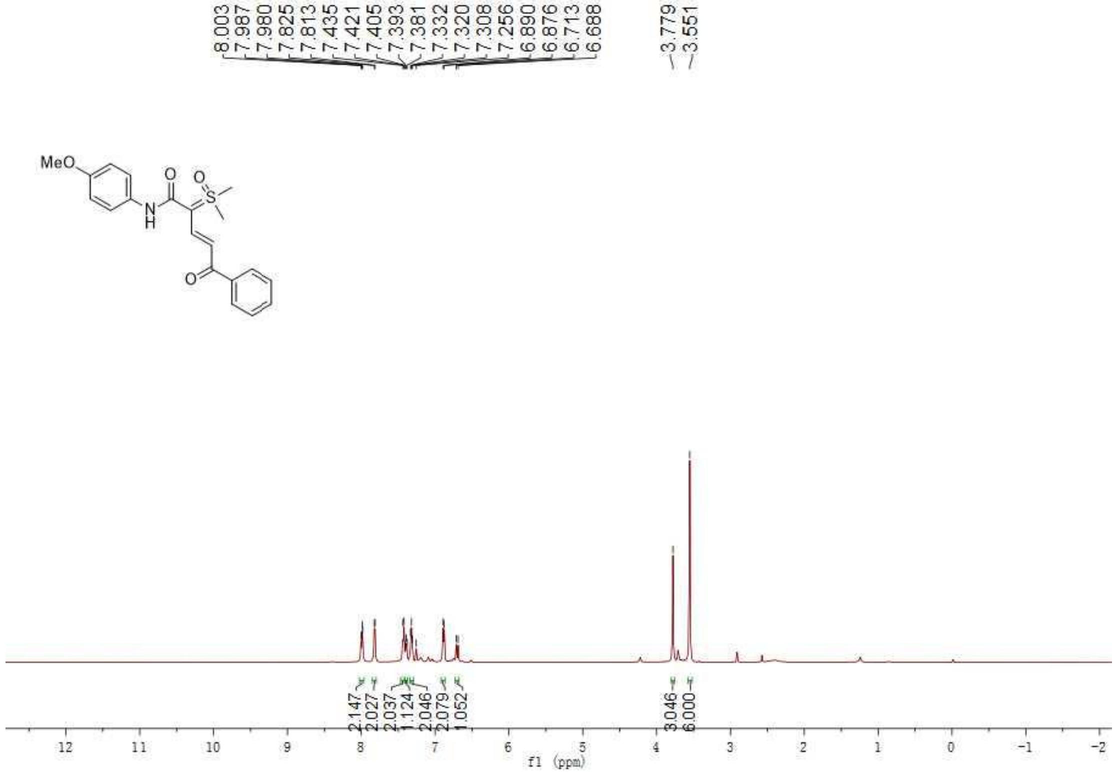

^{13}C NMR (150 MHz, $CDCl_3$) Spectrum of **83**

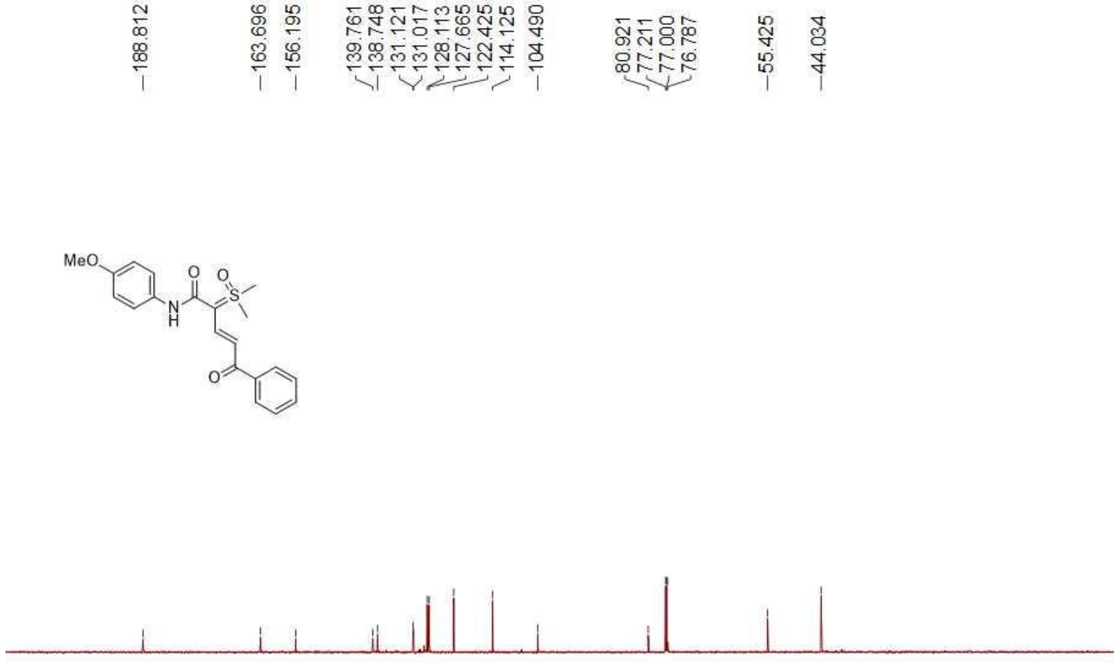

^{1}H NMR (600 MHz, $CDCl_3$) Spectrum of **84**

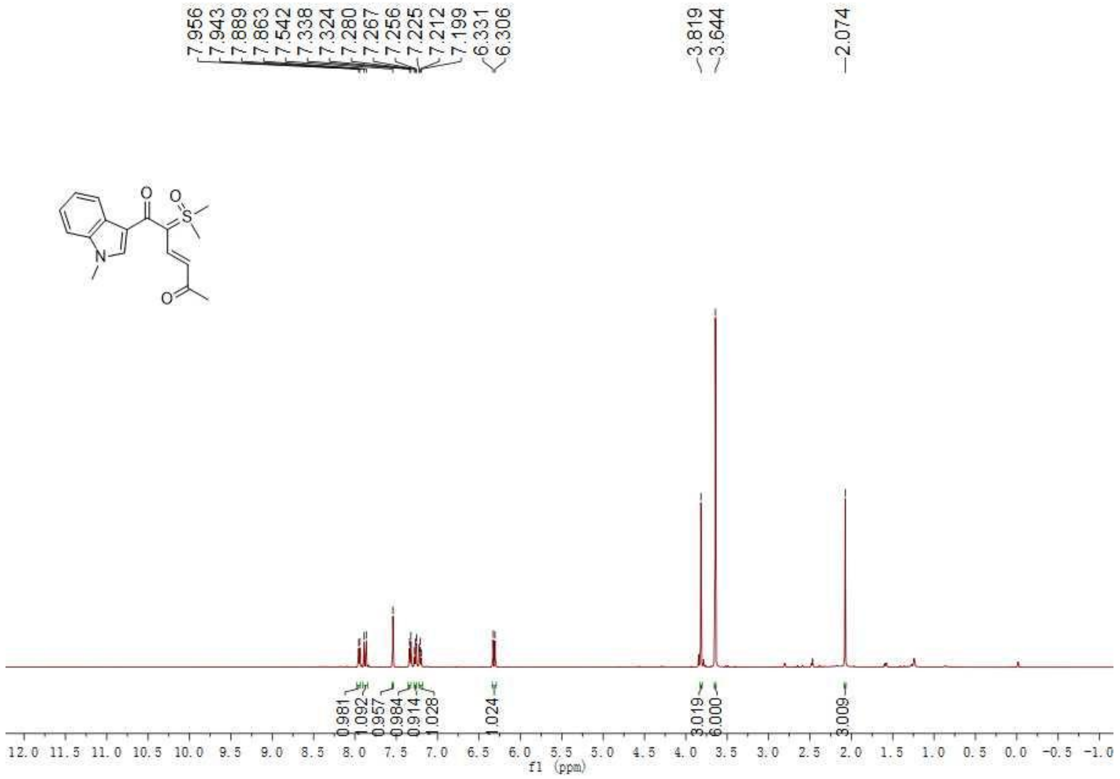

^{13}C NMR (150 MHz, $CDCl_3$) Spectrum of **84**

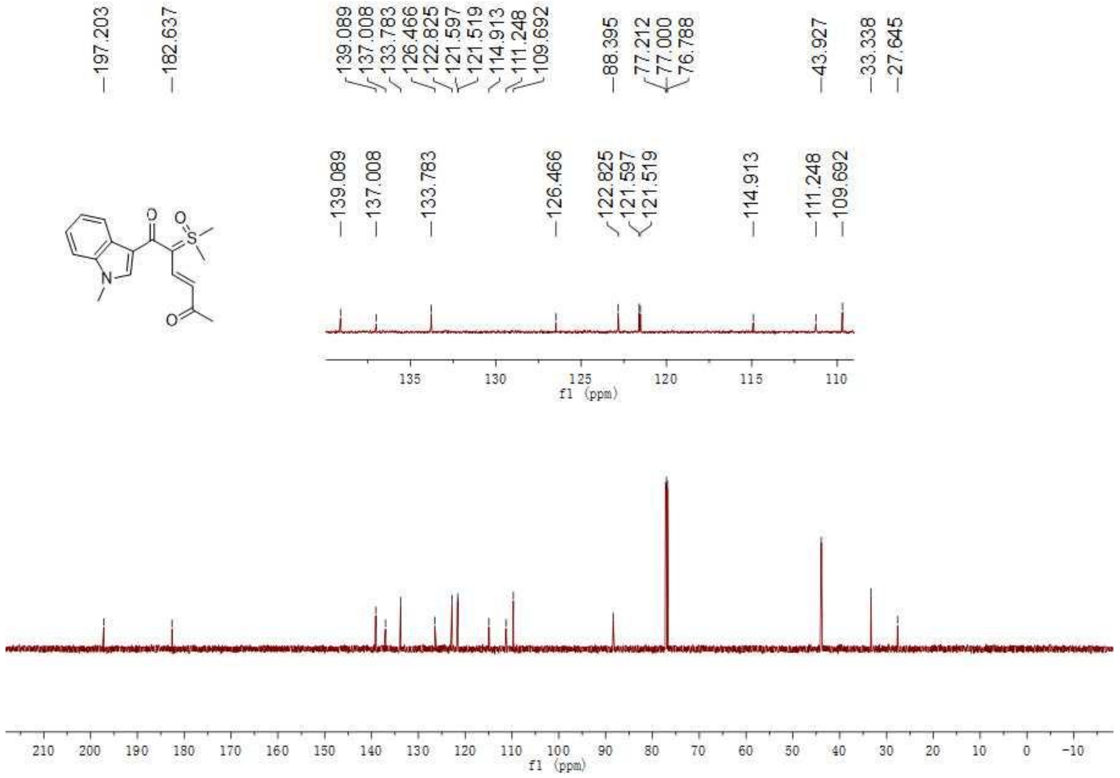

^{1}H NMR (600 MHz, $CDCl_3$) Spectrum of **85**

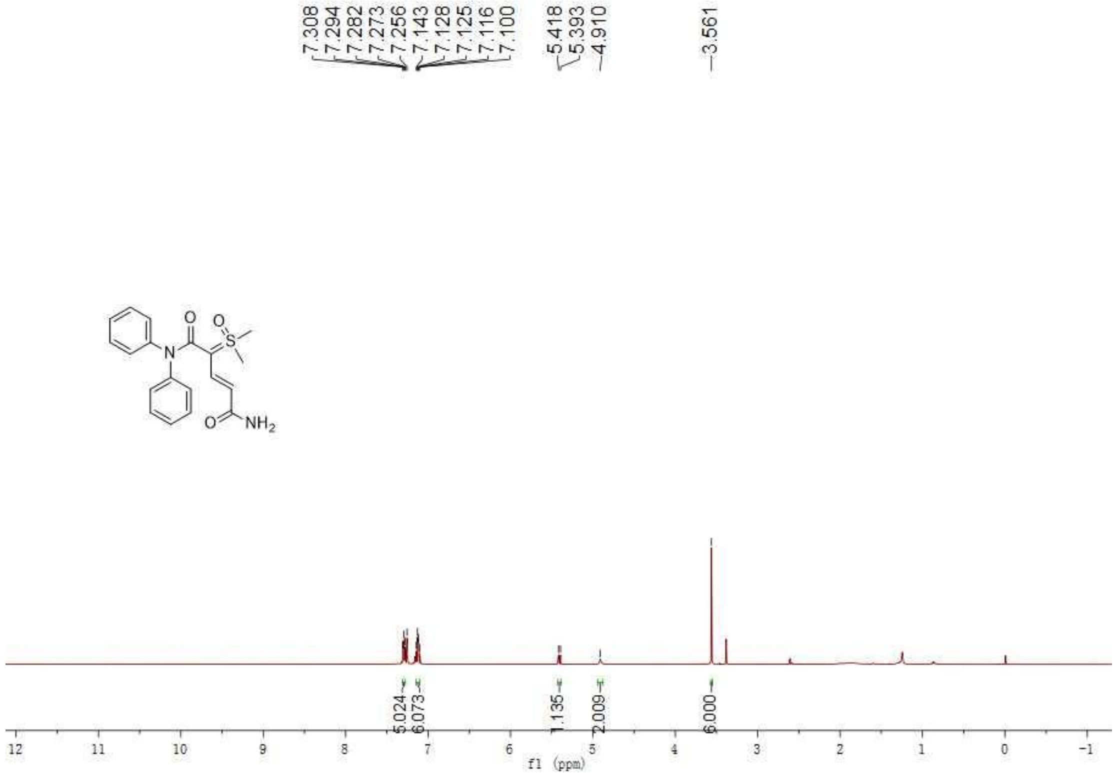

^{13}C NMR (150 MHz, $CDCl_3$) Spectrum of **85**

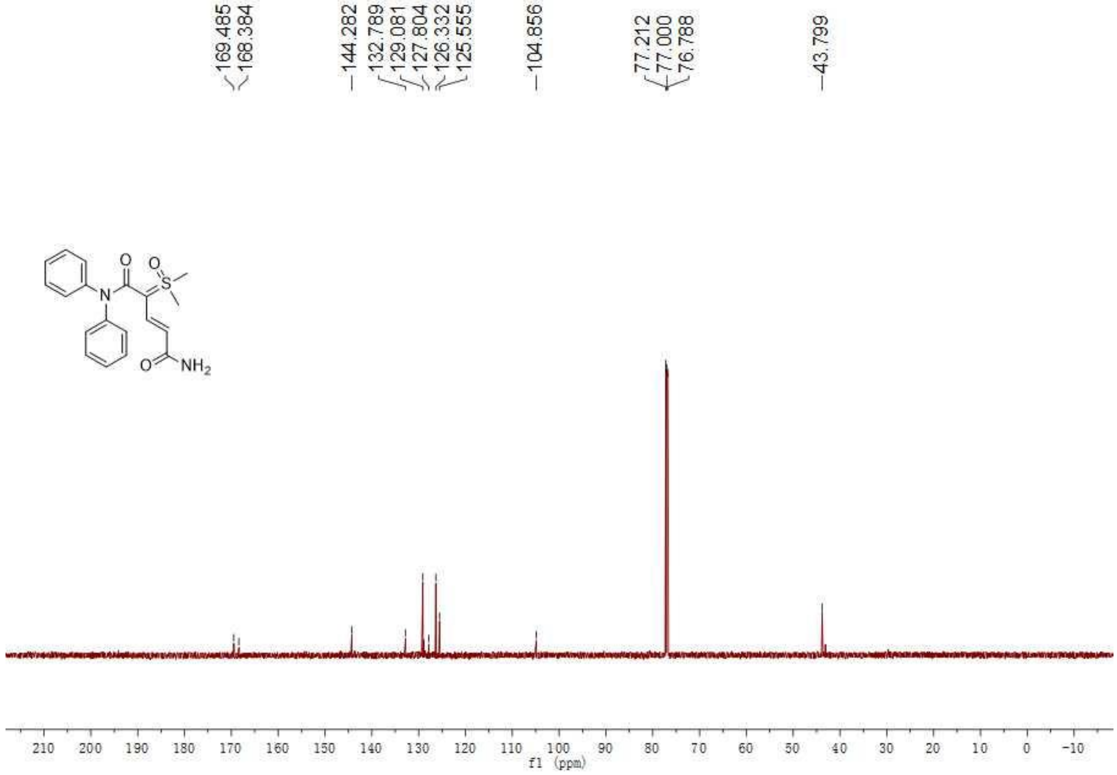

^{1}H NMR (600 MHz, DMSO-d_6) Spectrum of **86**

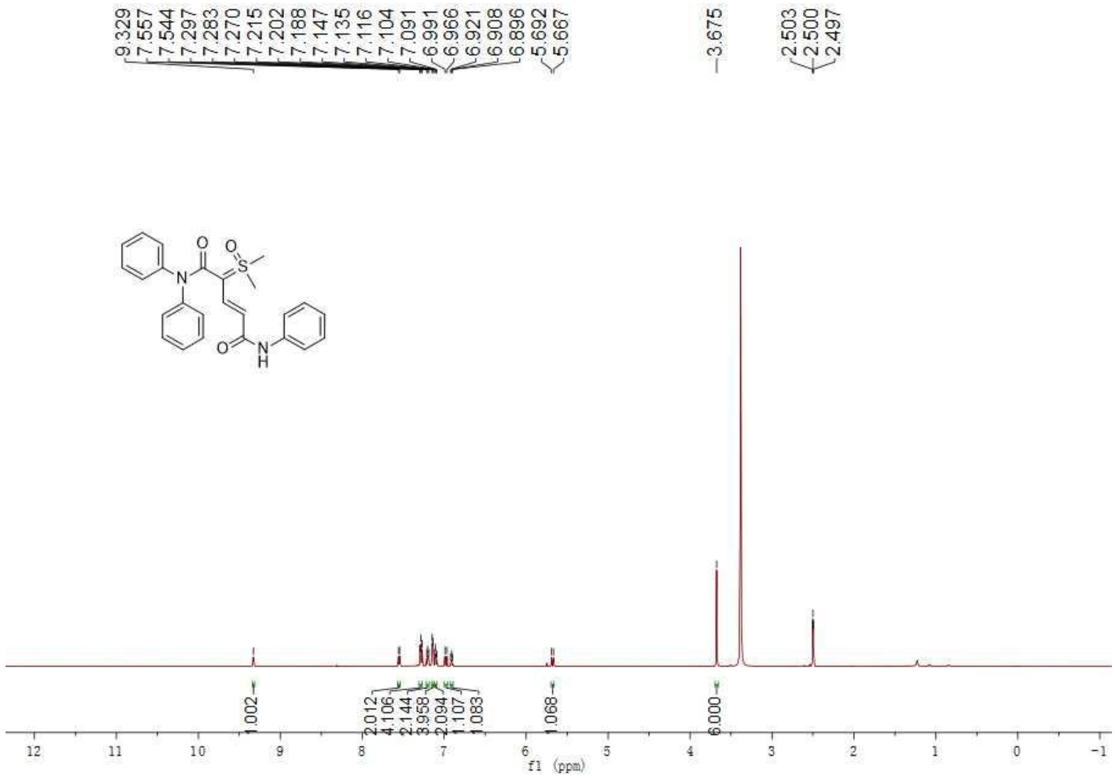

^{13}C NMR (150 MHz, DMSO-d_6) Spectrum of **86**

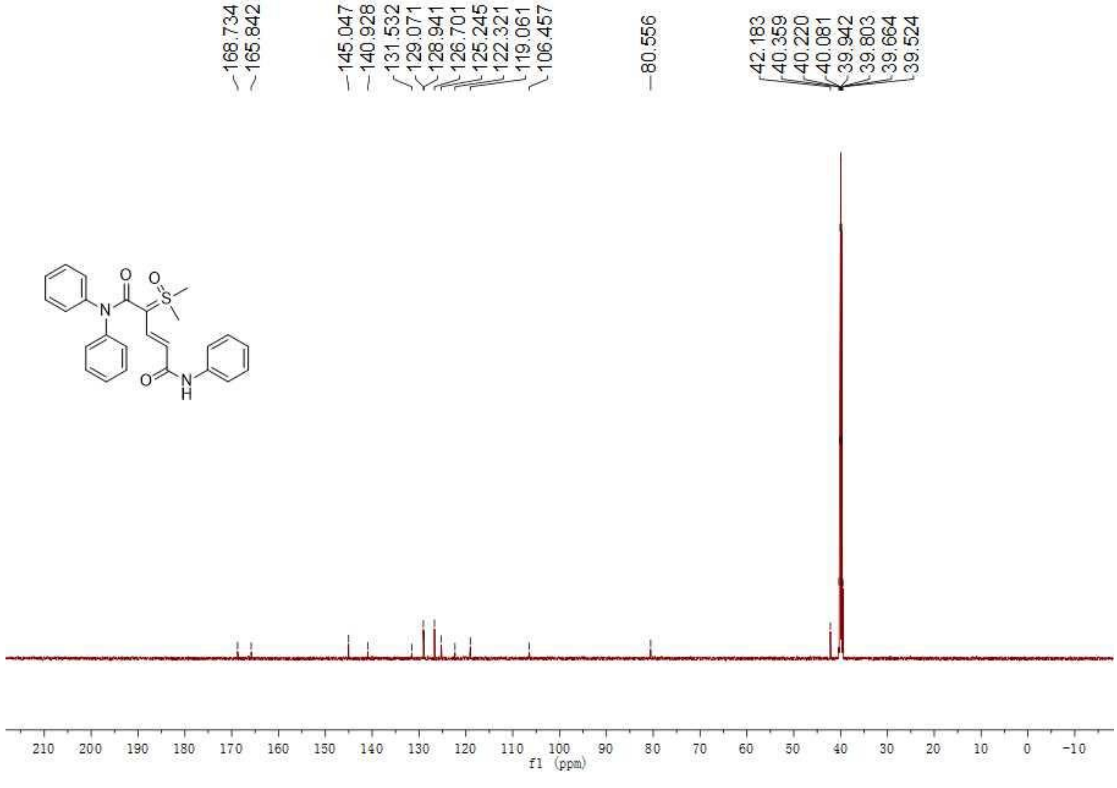

^{1}H NMR (600 MHz, $CDCl_3$) Spectrum of **87**

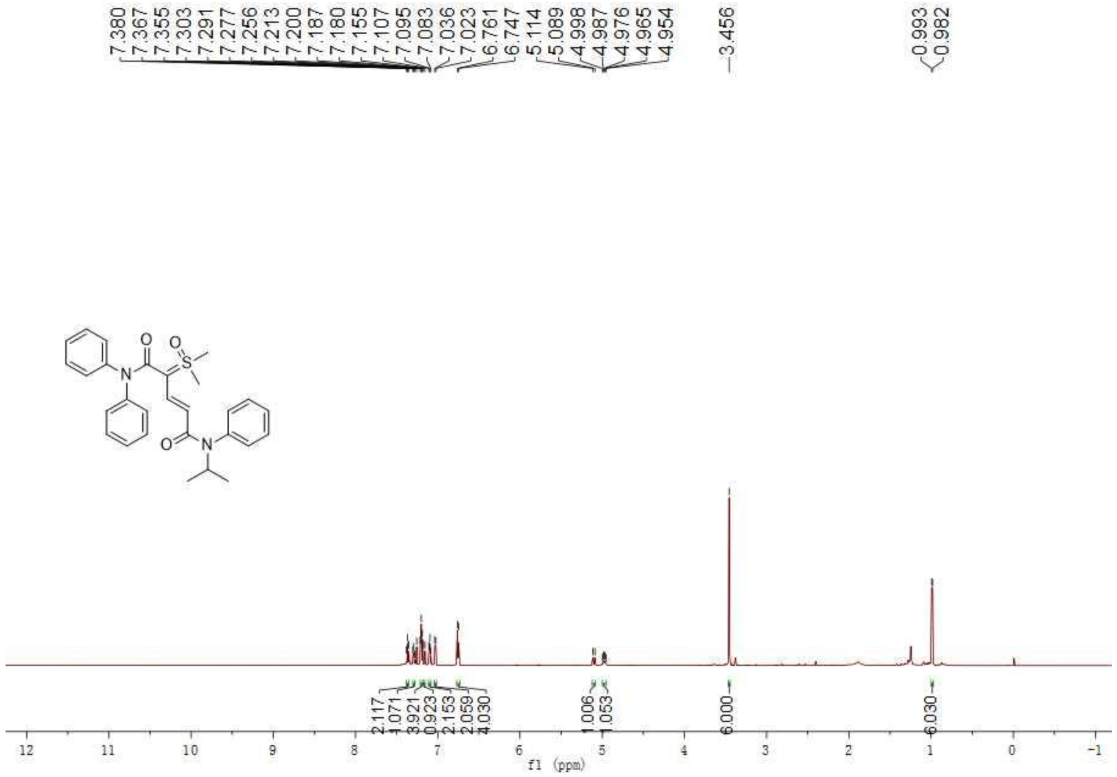

^{13}C NMR (150 MHz, $CDCl_3$) Spectrum of **87**

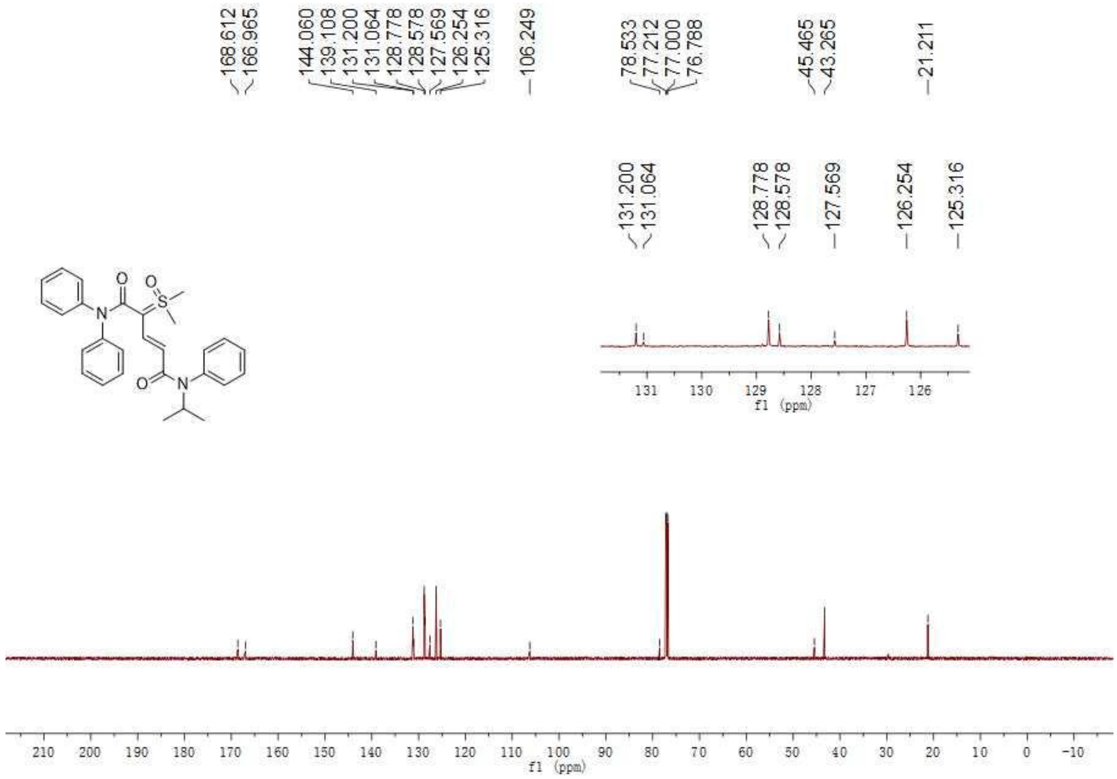

^{1}H NMR (600 MHz, $CDCl_3$) Spectrum of **88**

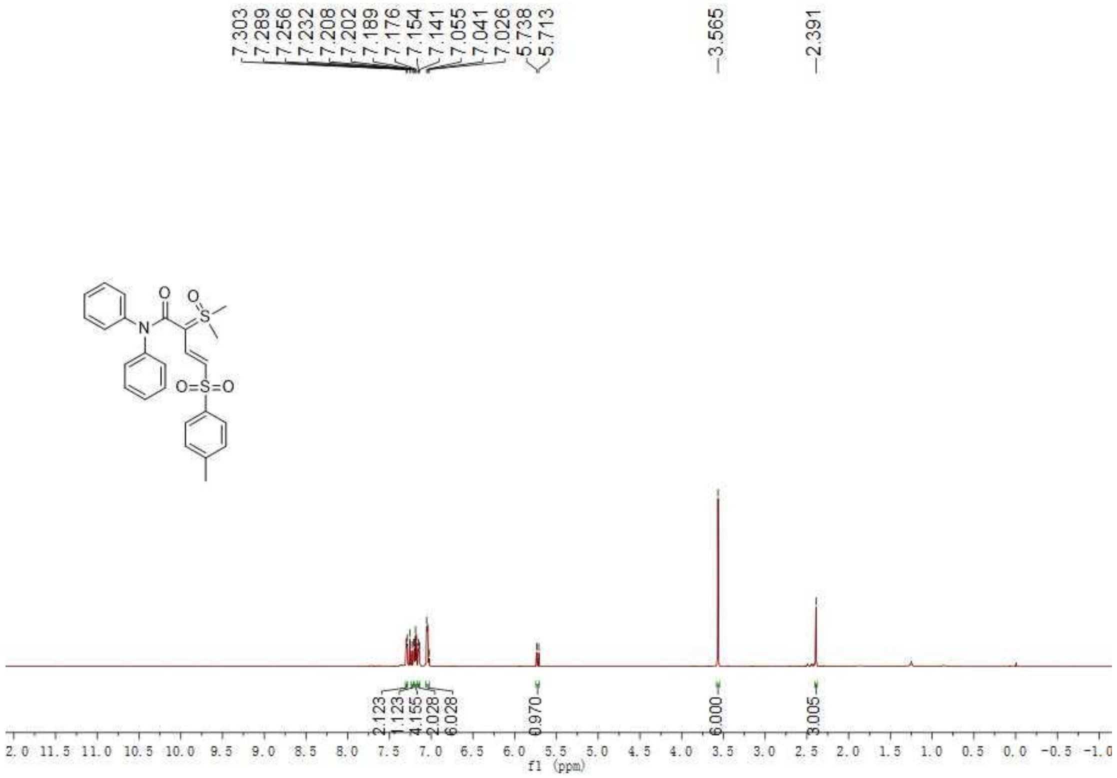

^{13}C NMR (150 MHz, $CDCl_3$) Spectrum of **88**

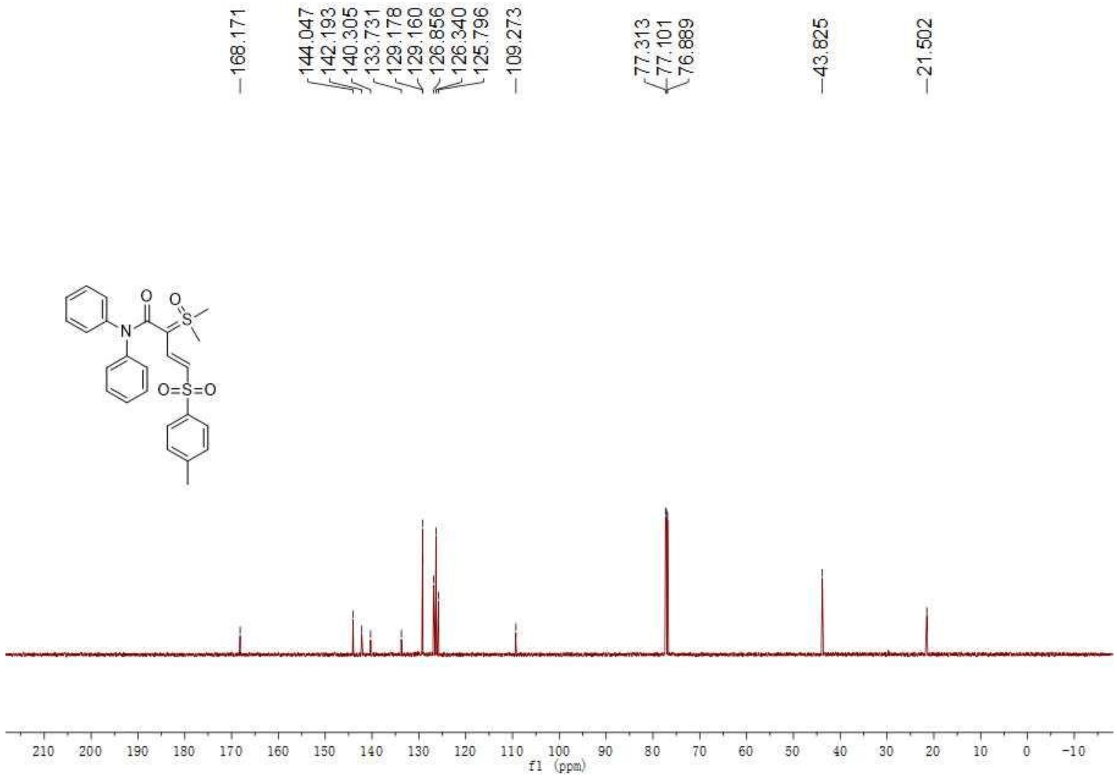

^{1}H NMR (600 MHz, $CDCl_3$) Spectrum of **89**

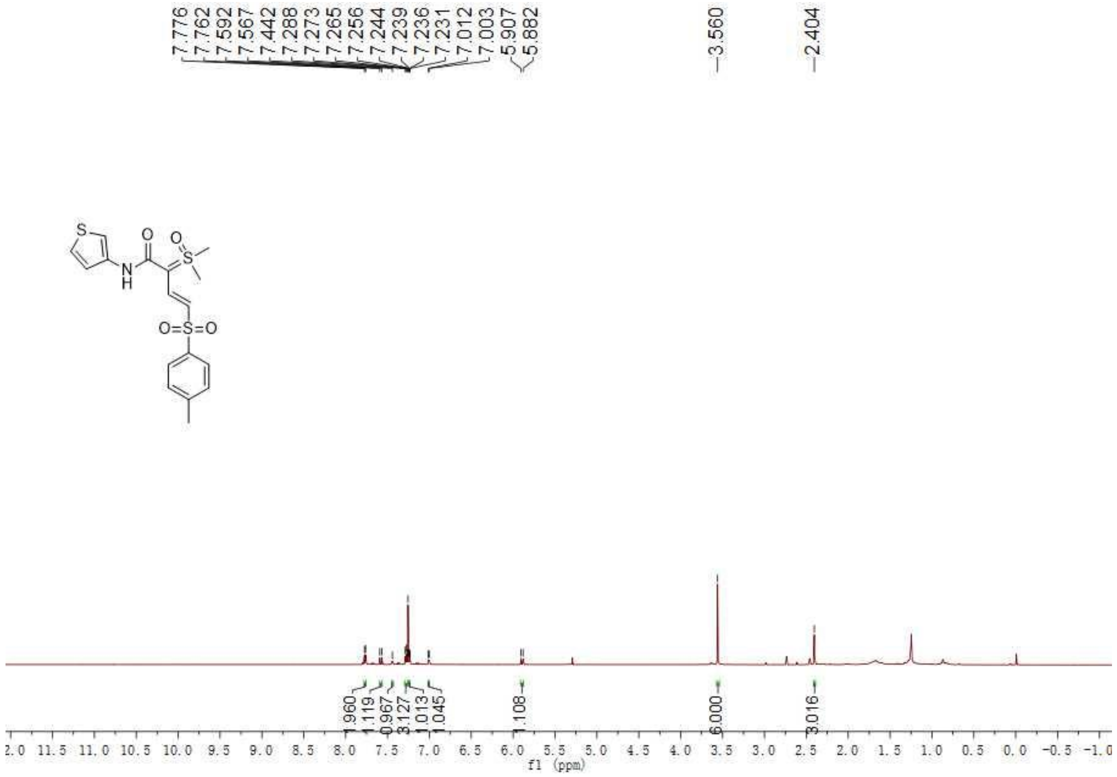

^{13}C NMR (150 MHz, $CDCl_3$) Spectrum of **89**

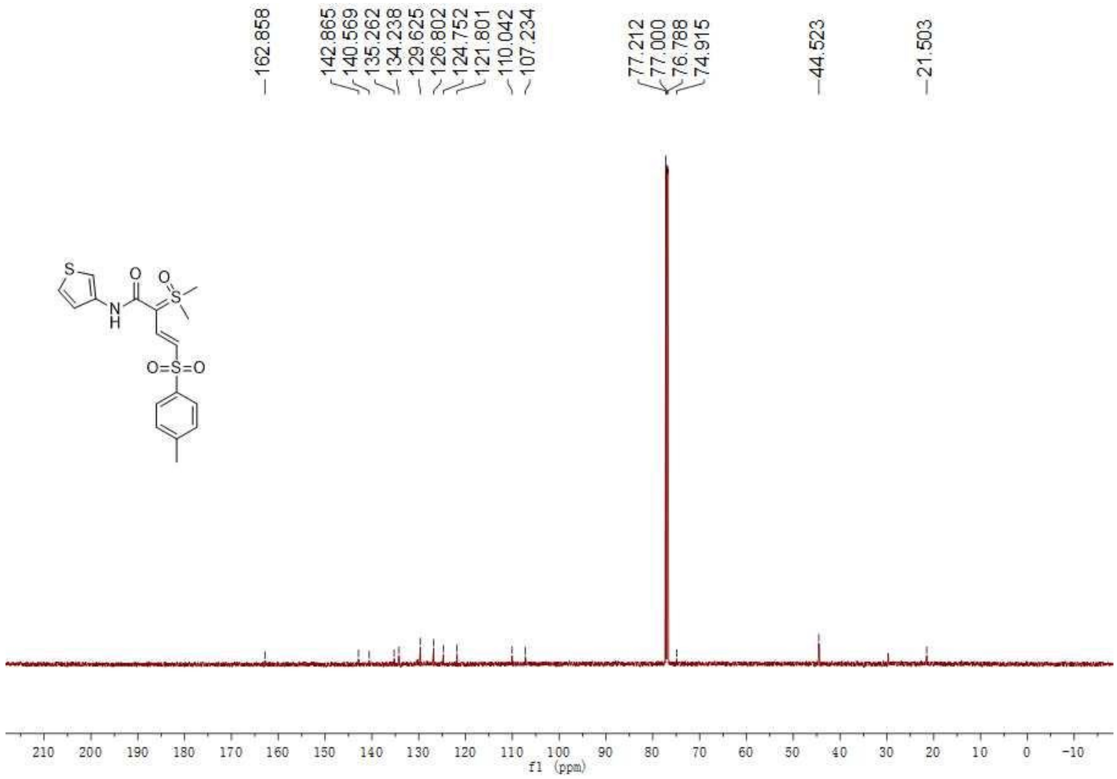

^{1}H NMR (600 MHz, $CDCl_3$) Spectrum of **90**

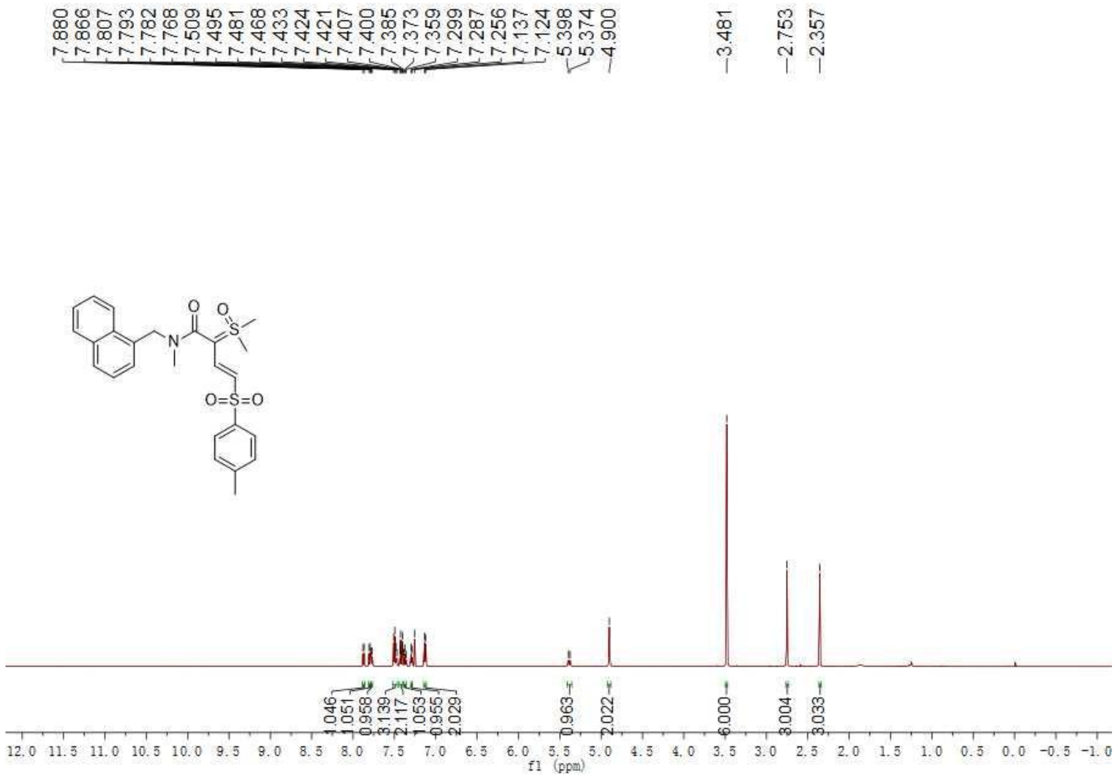

^{13}C NMR (150 MHz, $CDCl_3$) Spectrum of **90**

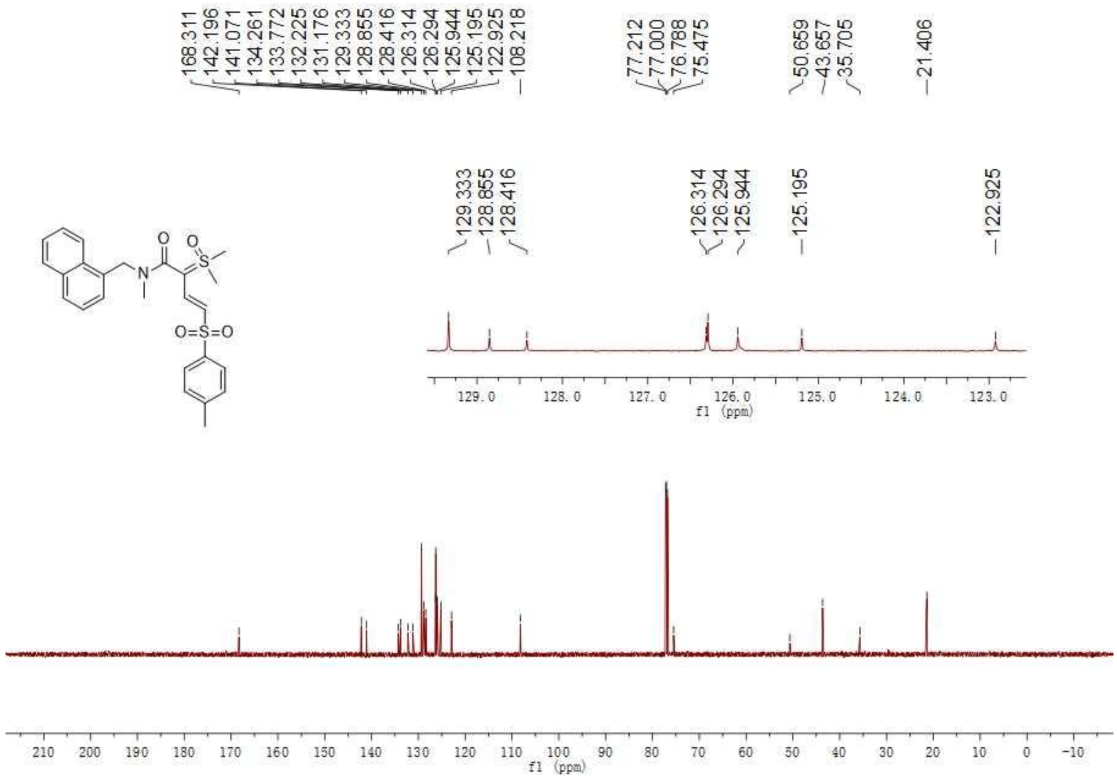

^{1}H NMR (600 MHz, $CDCl_3$) Spectrum of **91**

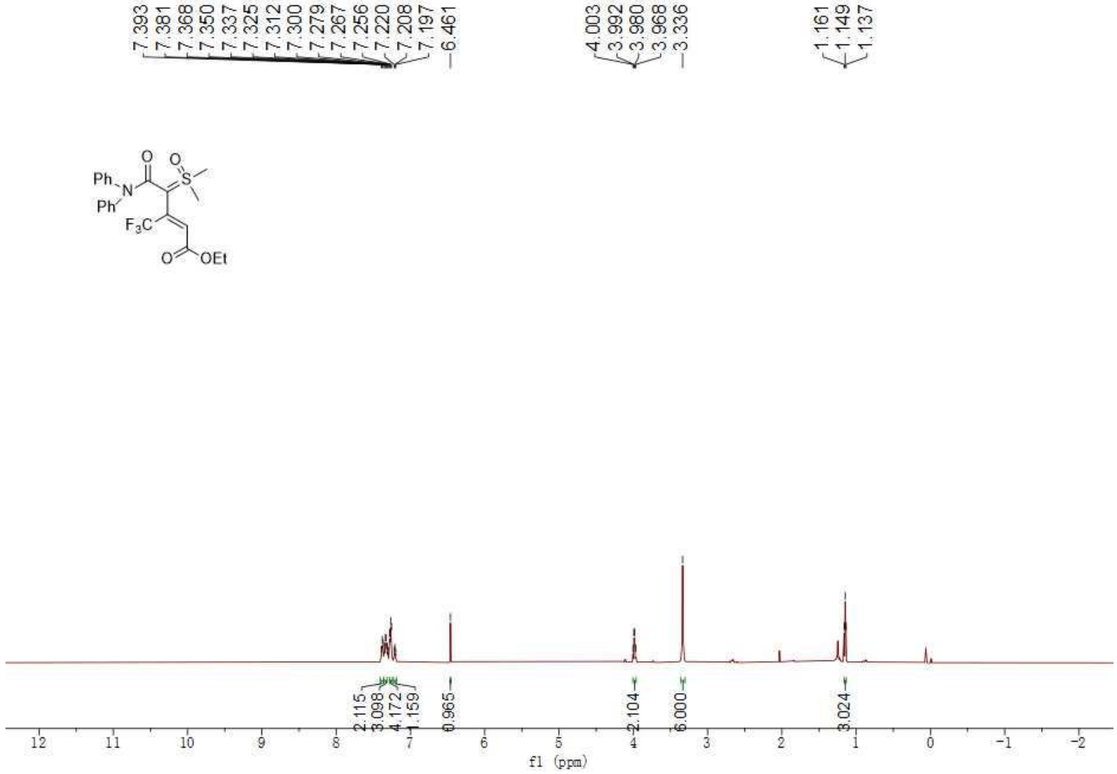

^{13}C NMR (150 MHz, $CDCl_3$) Spectrum of **91**

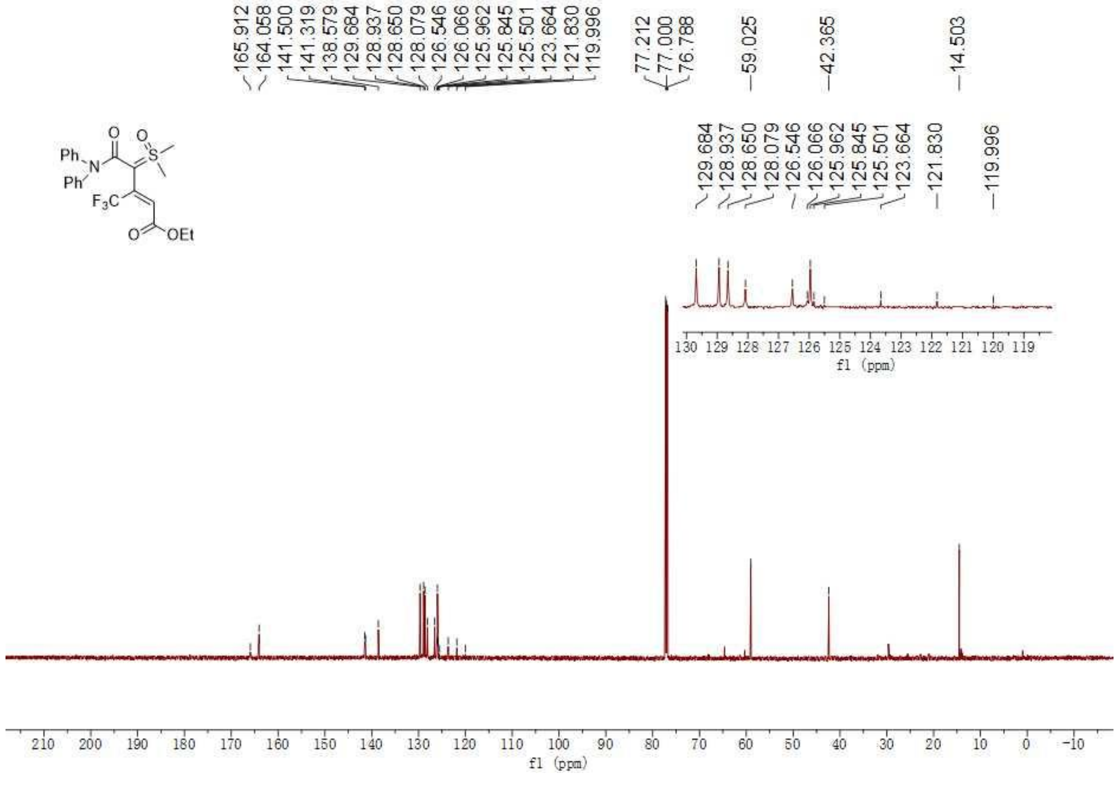

^{19}F NMR (564 MHz, $CDCl_3$) Spectrum of **91**

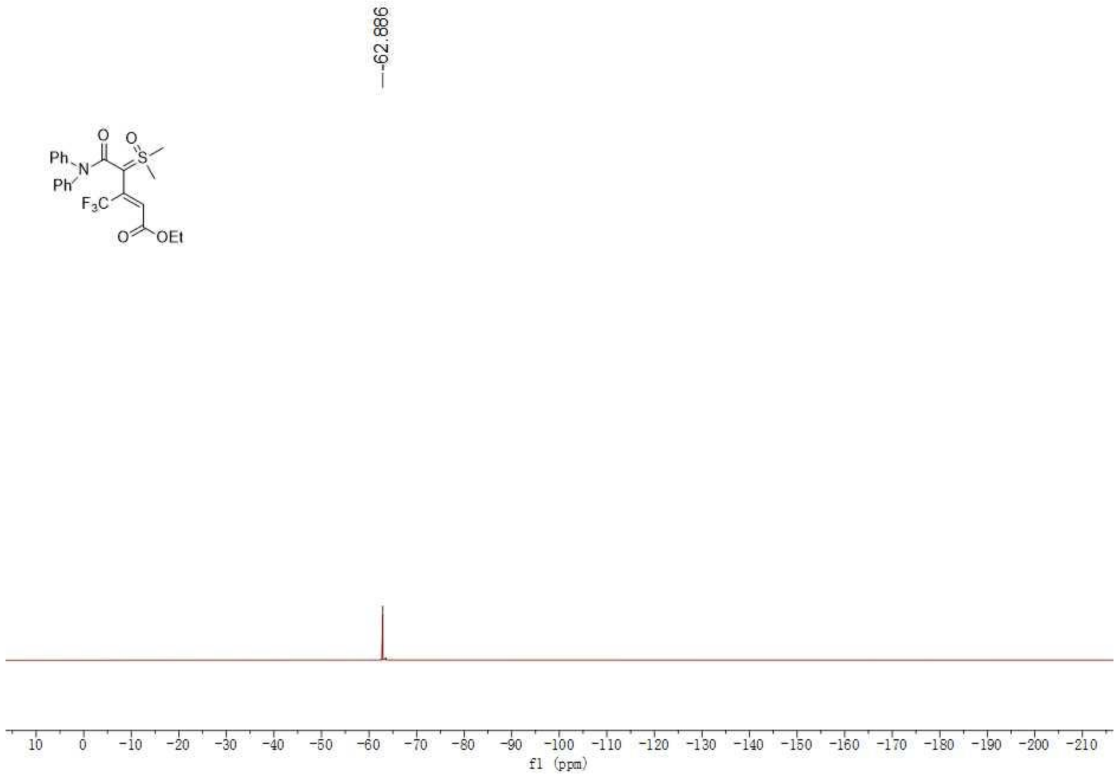

^{1}H NMR (600 MHz, $CDCl_3$) Spectrum of **93**

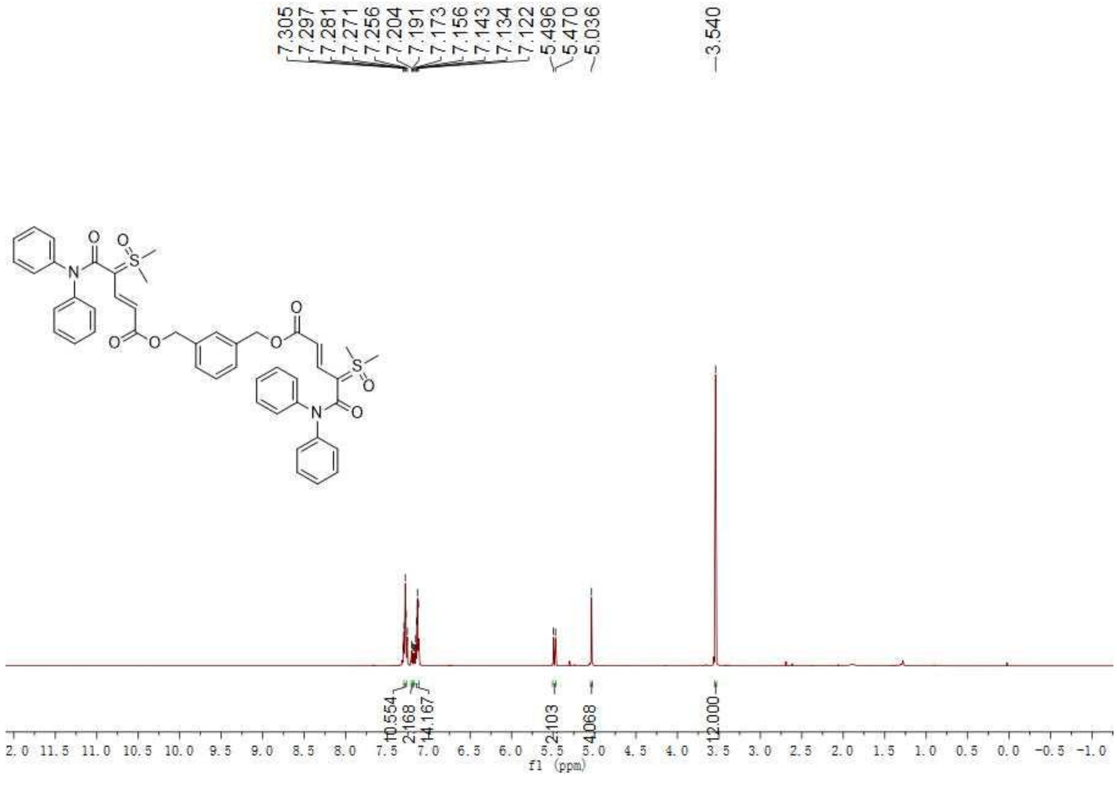

^{13}C NMR (150 MHz, $CDCl_3$) Spectrum of **93**

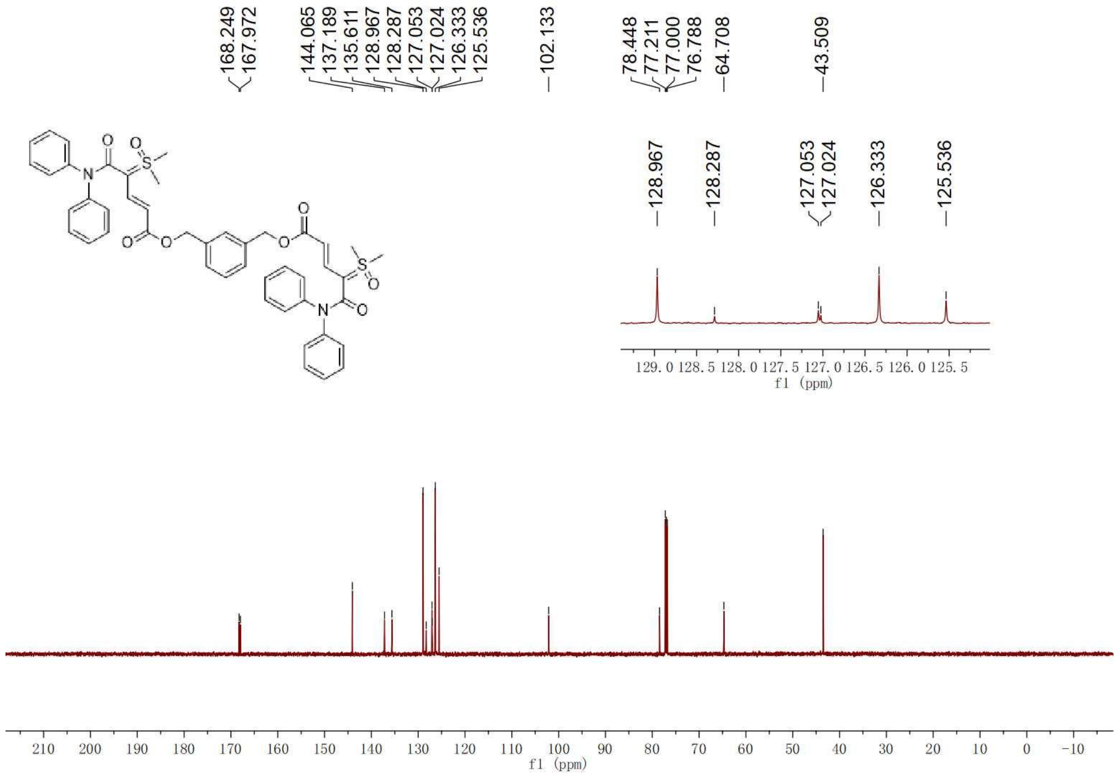

^{1}H NMR (600 MHz, $CDCl_3$) Spectrum of **94**

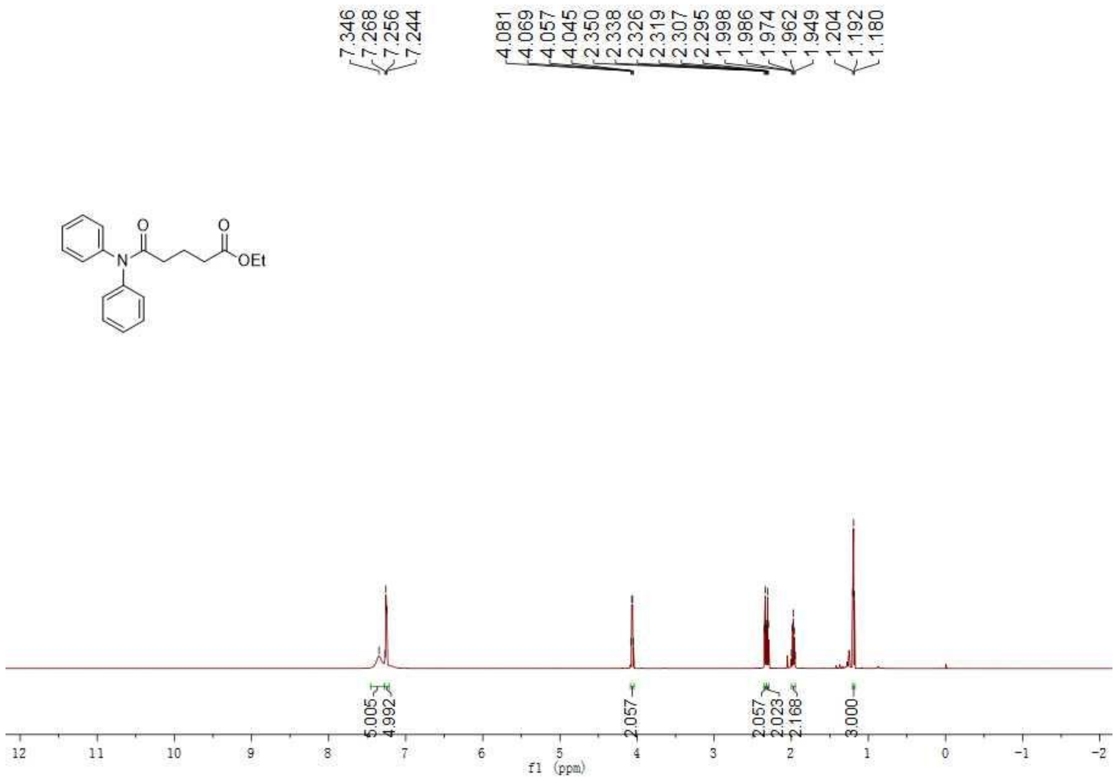

^{13}C NMR (150 MHz, $CDCl_3$) Spectrum of **94**

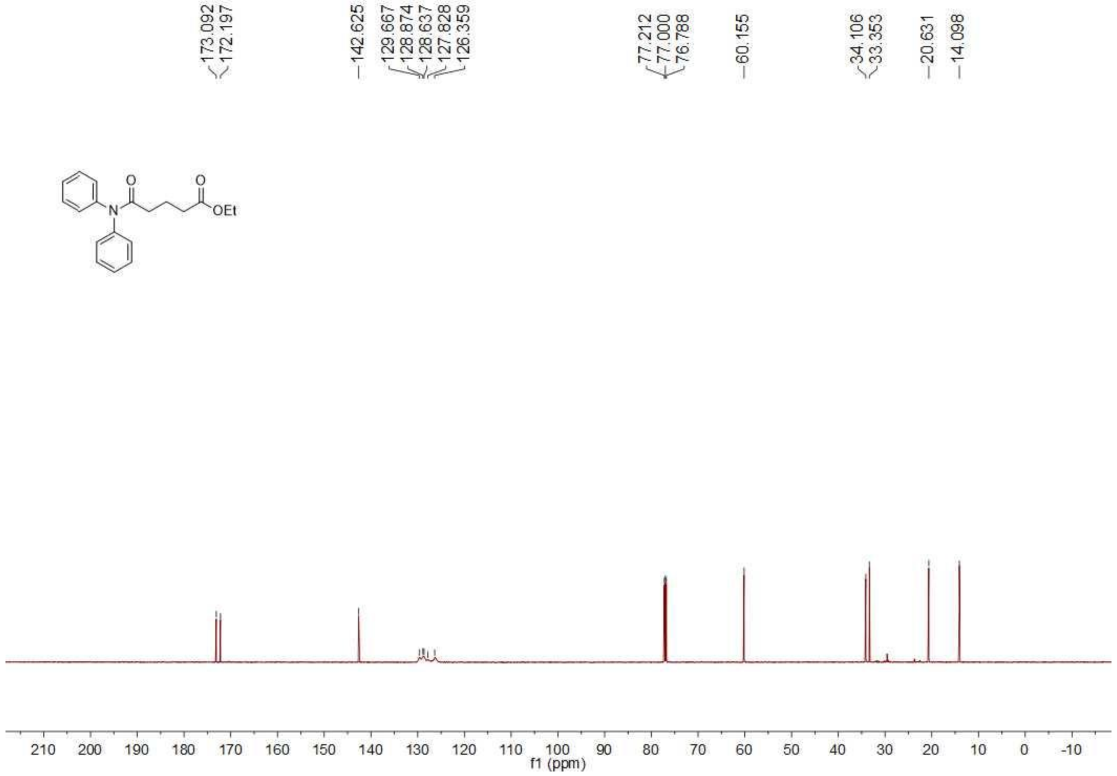

^{1}H NMR (600 MHz, $CDCl_3$) Spectrum of **95**

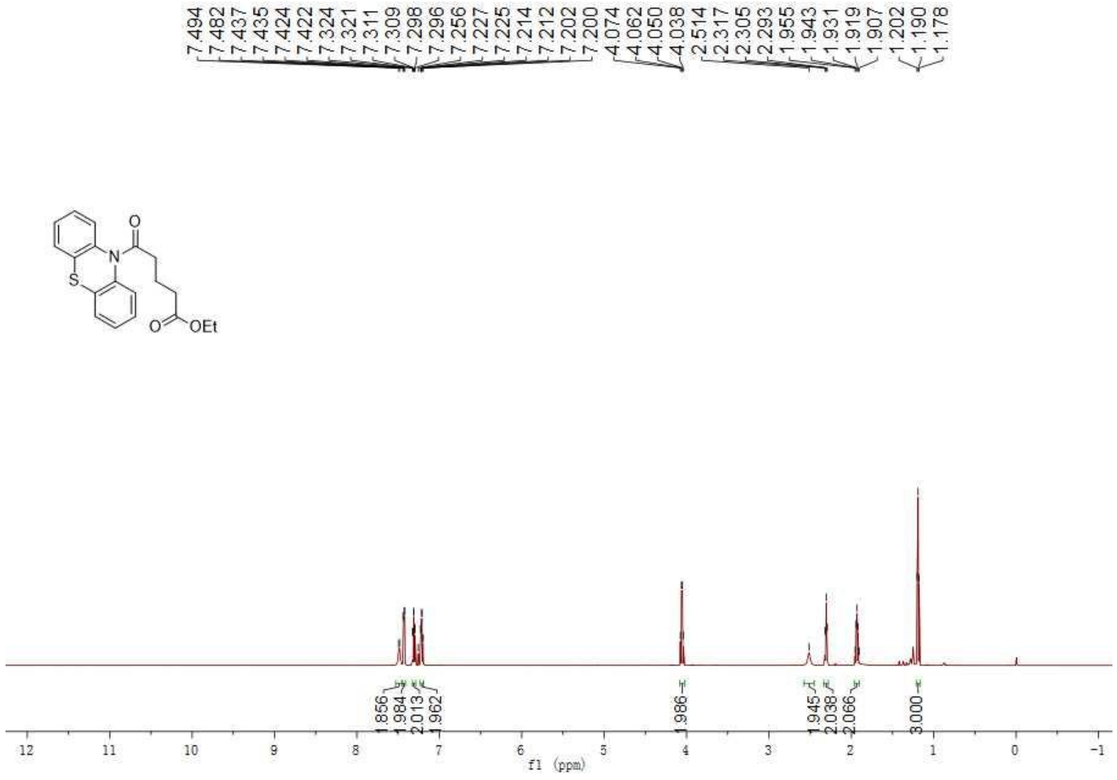

^{13}C NMR (150 MHz, $CDCl_3$) Spectrum of **95**

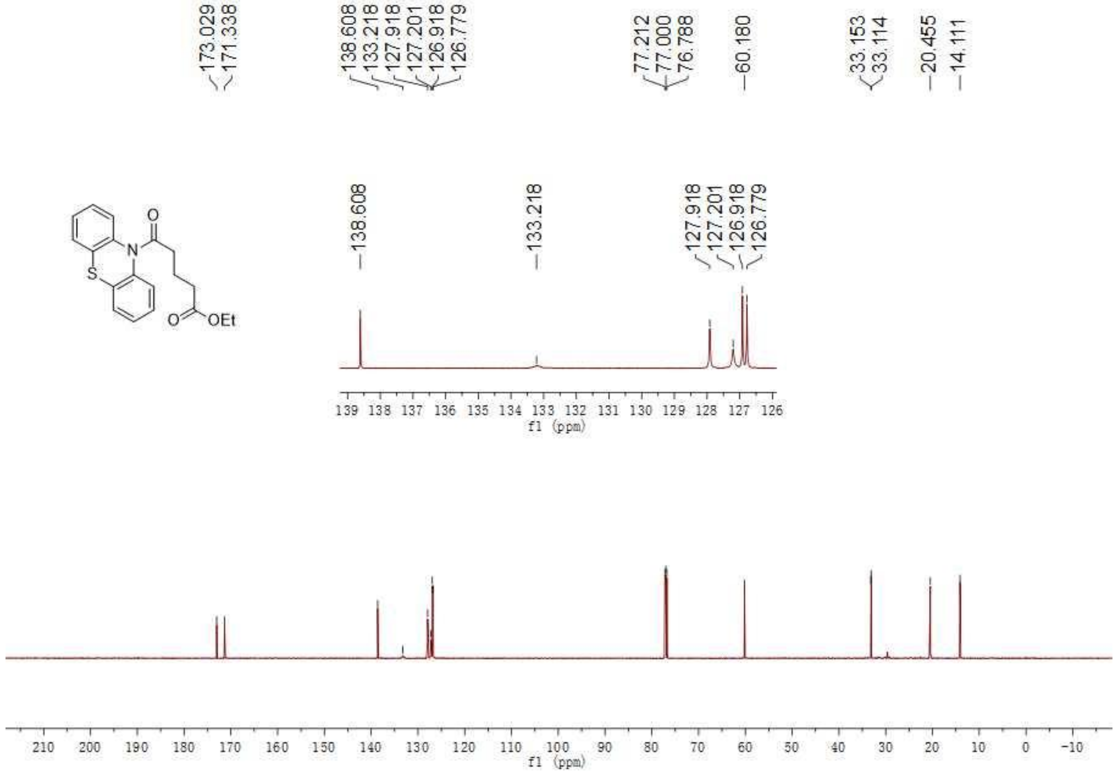

^{1}H NMR (600 MHz, $CDCl_3$) Spectrum of **96**

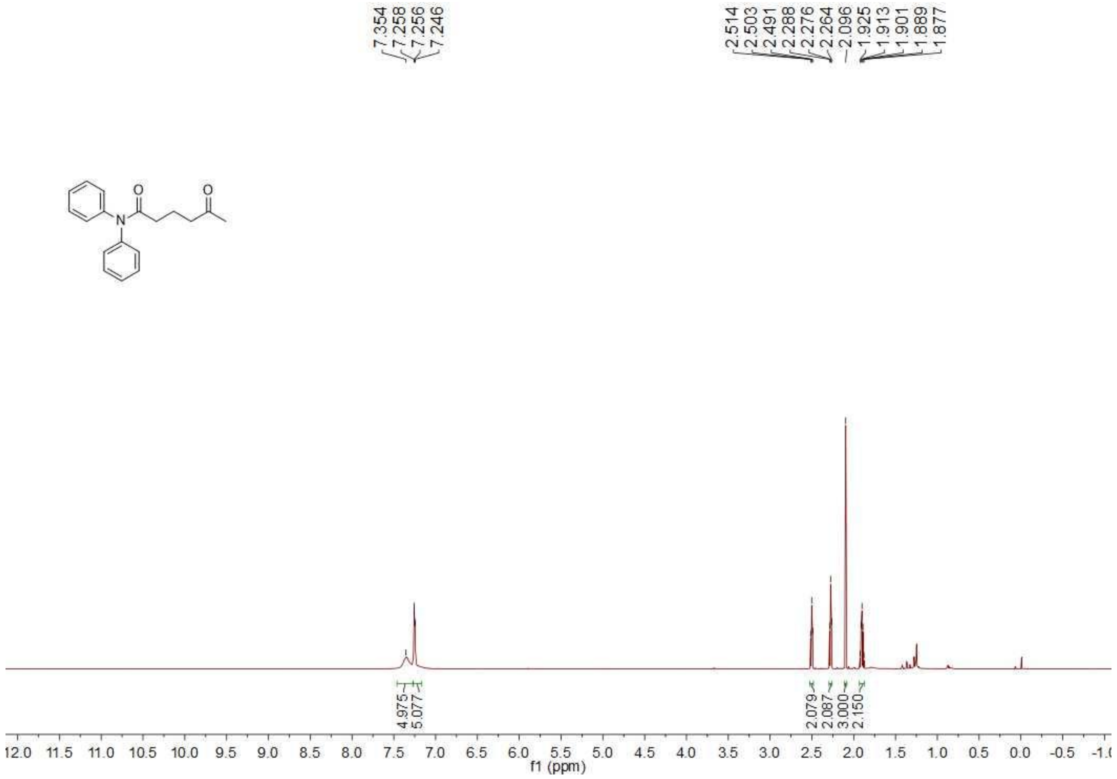

^{13}C NMR (150 MHz, $CDCl_3$) Spectrum of **96**

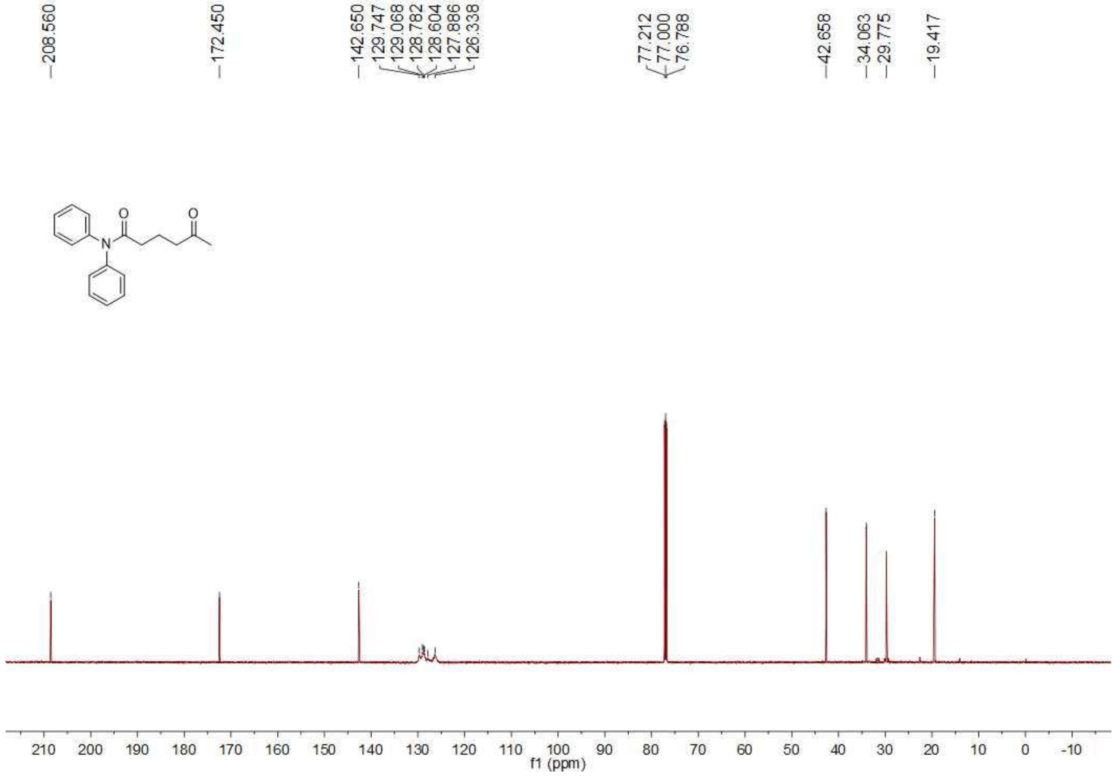

^{1}H NMR (600 MHz, $CDCl_3$) Spectrum of **98**

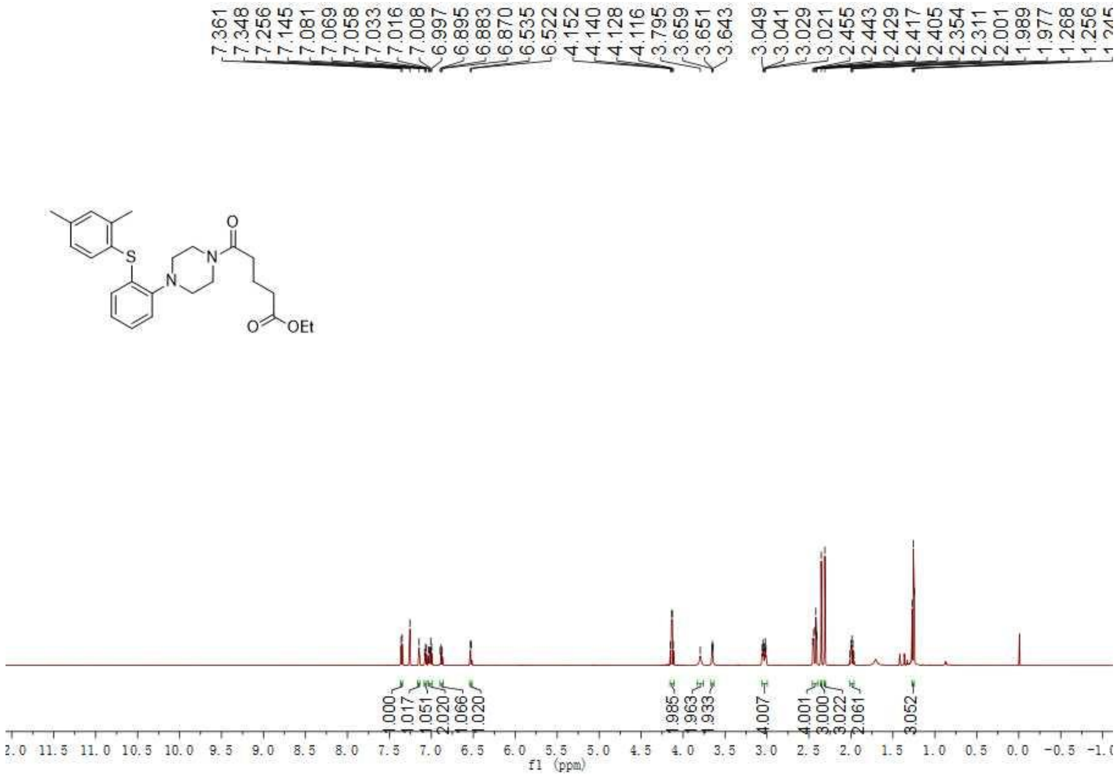

^{13}C NMR (150 MHz, $CDCl_3$) Spectrum of **98**

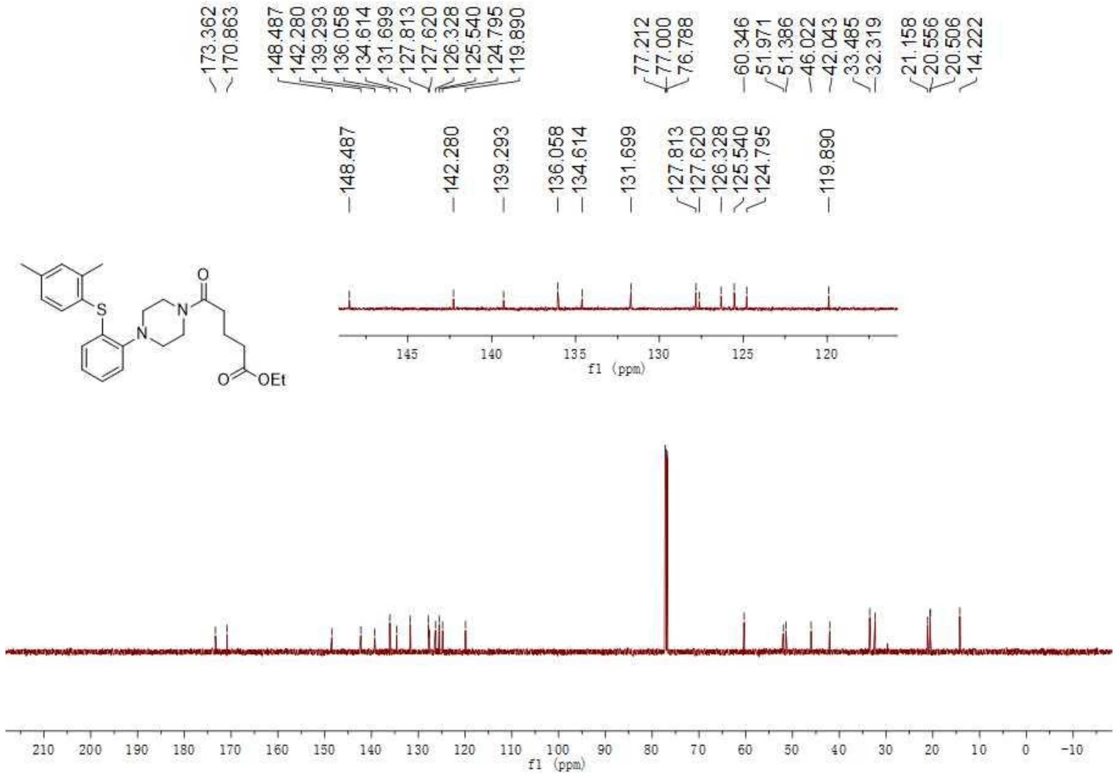

^{1}H NMR (600 MHz, $CDCl_3$) Spectrum of **100**

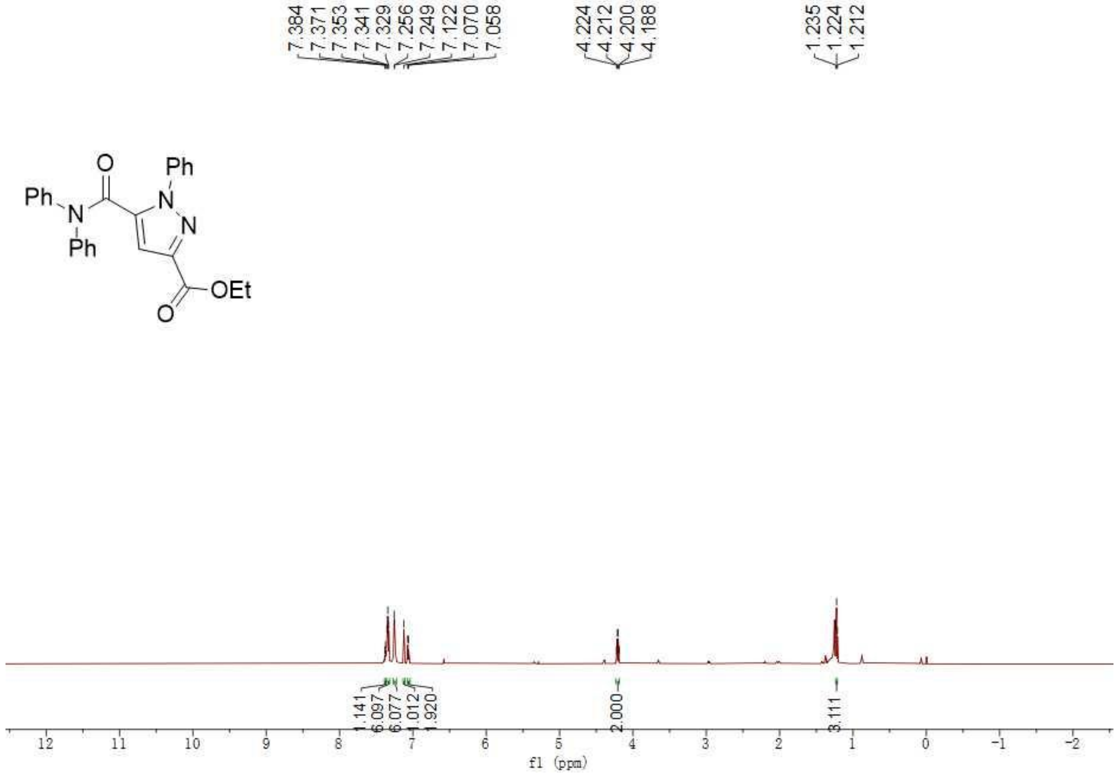

^{13}C NMR (150 MHz, $CDCl_3$) Spectrum of **100**

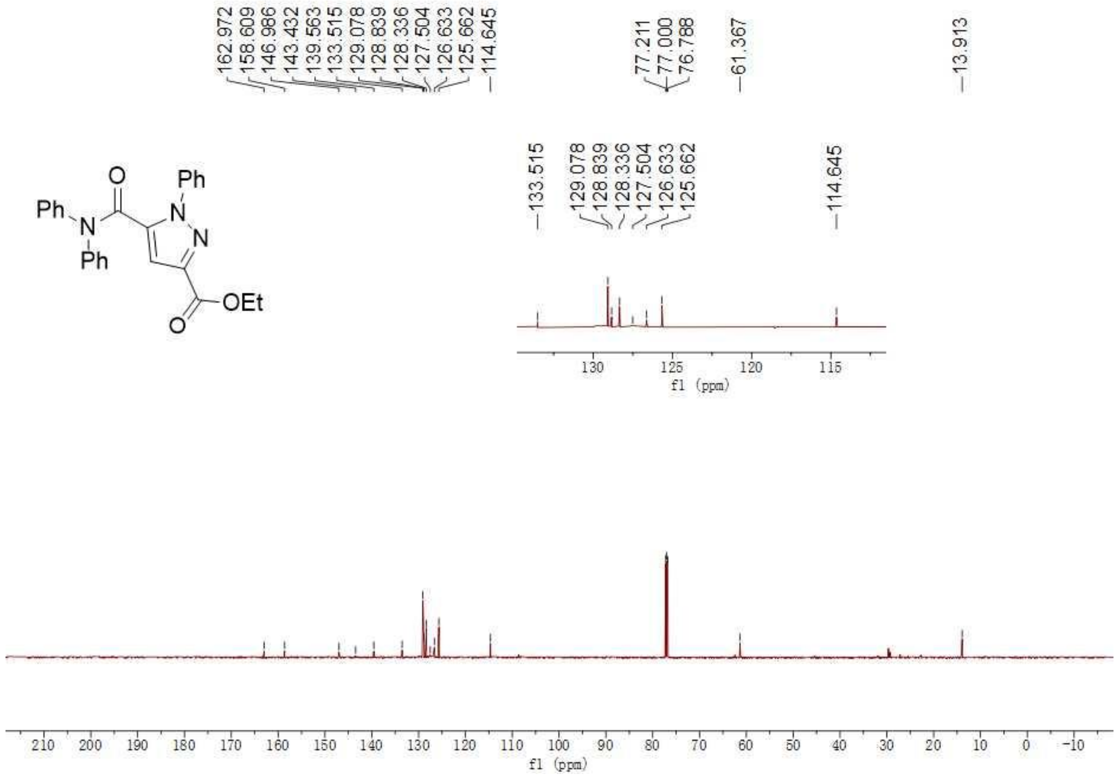

^{1}H NMR (600 MHz, $CDCl_3$) Spectrum of **101**

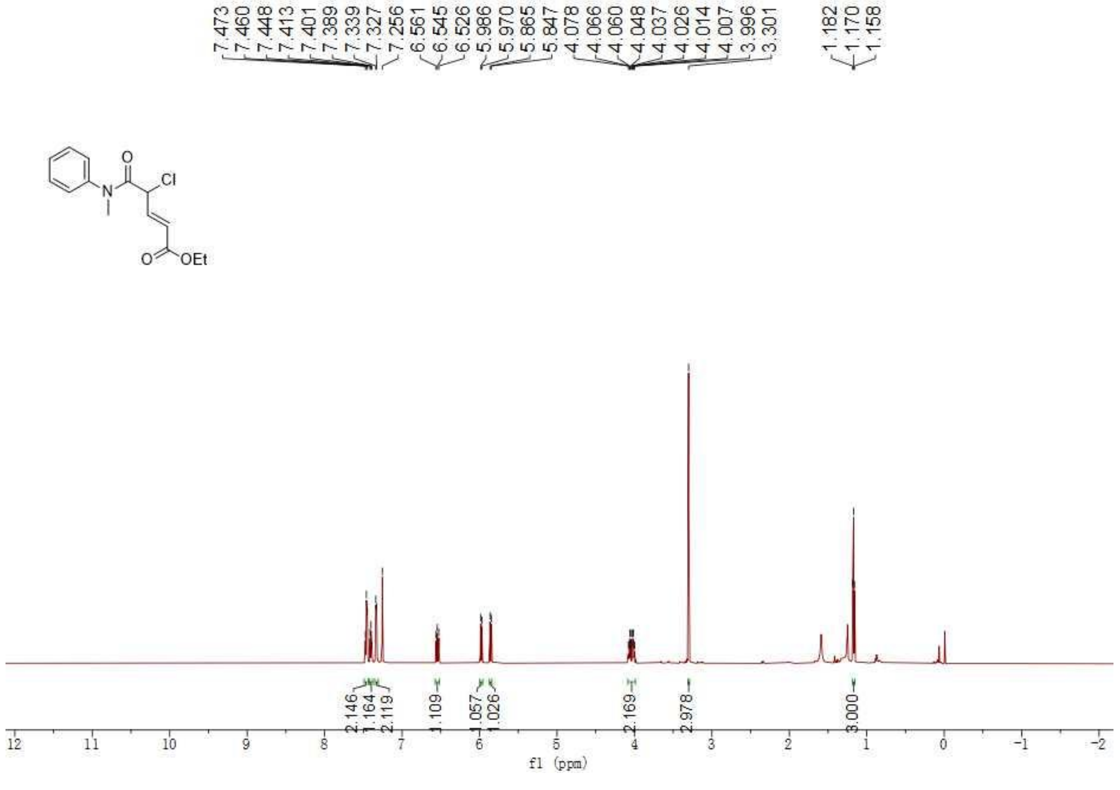

^{13}C NMR (150 MHz, $CDCl_3$) Spectrum of **101**

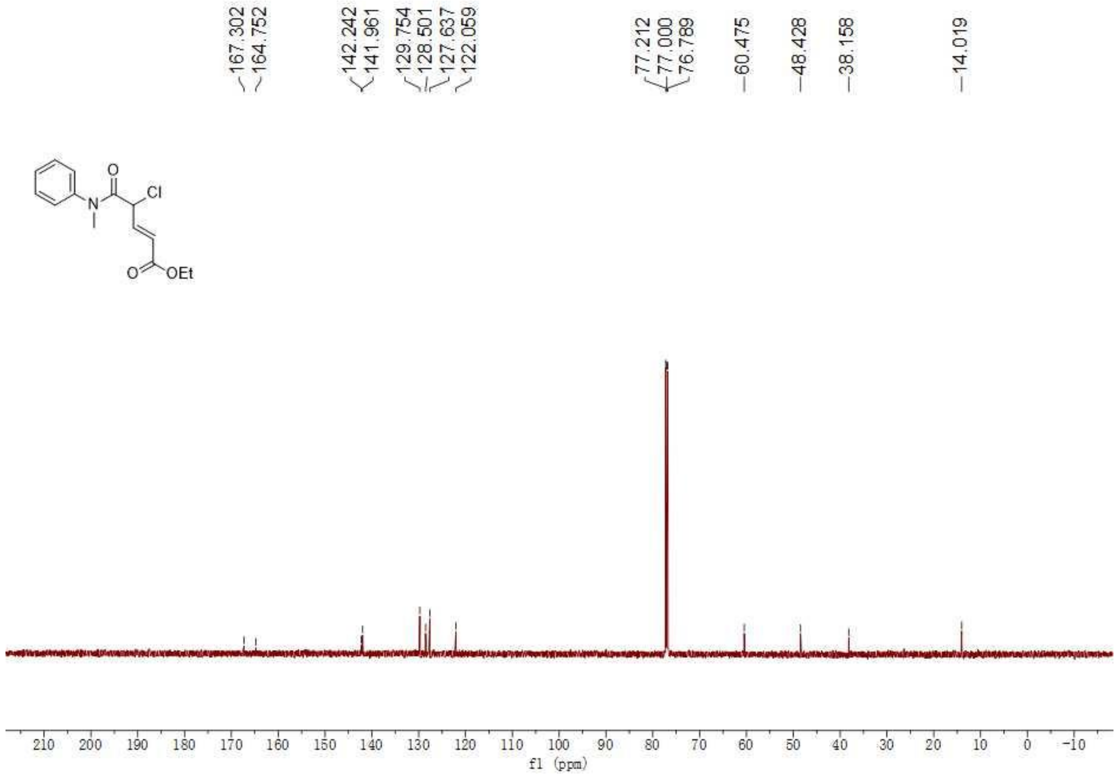

^{1}H NMR (600 MHz, $CDCl_3$) Spectrum of **102**

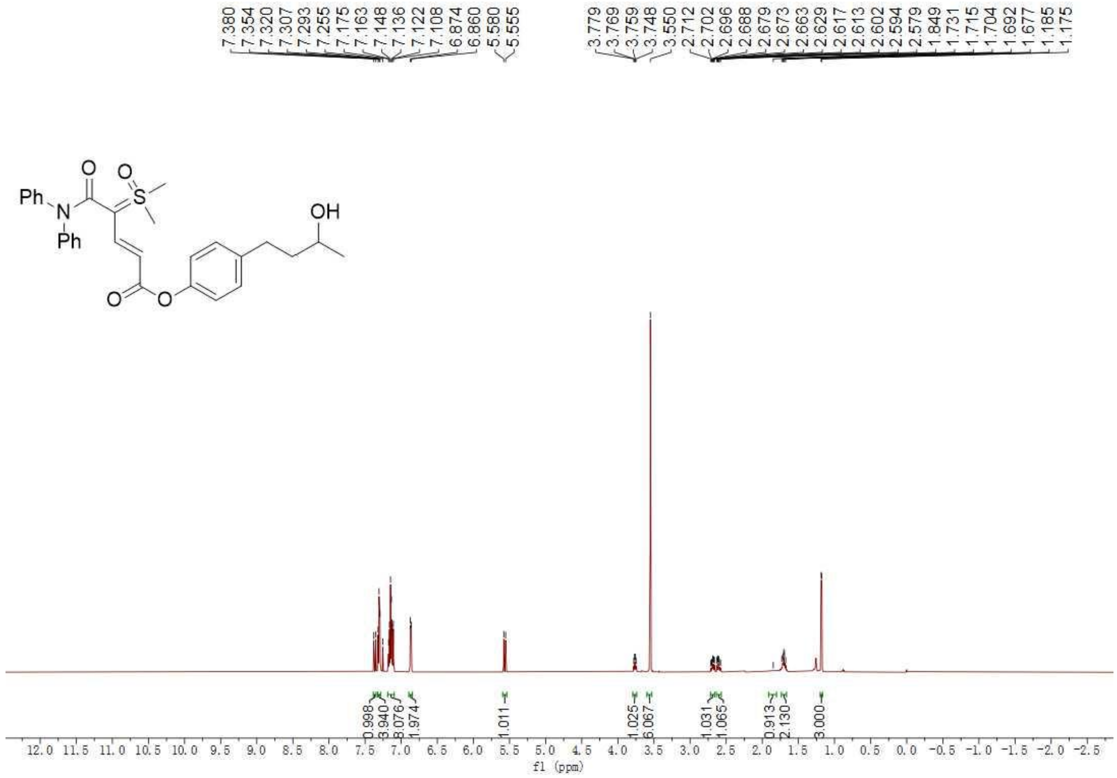

^{13}C NMR (150 MHz, $CDCl_3$) Spectrum of **102**

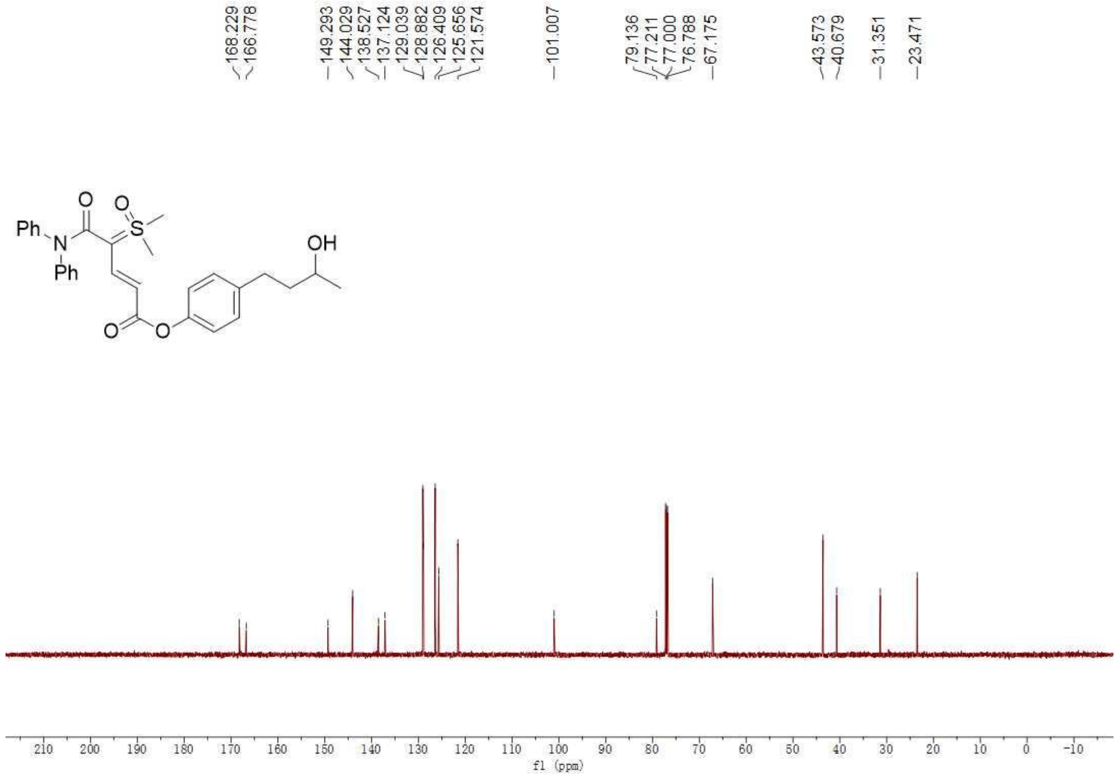

^{1}H NMR (600 MHz, $CDCl_3$) Spectrum of **103**

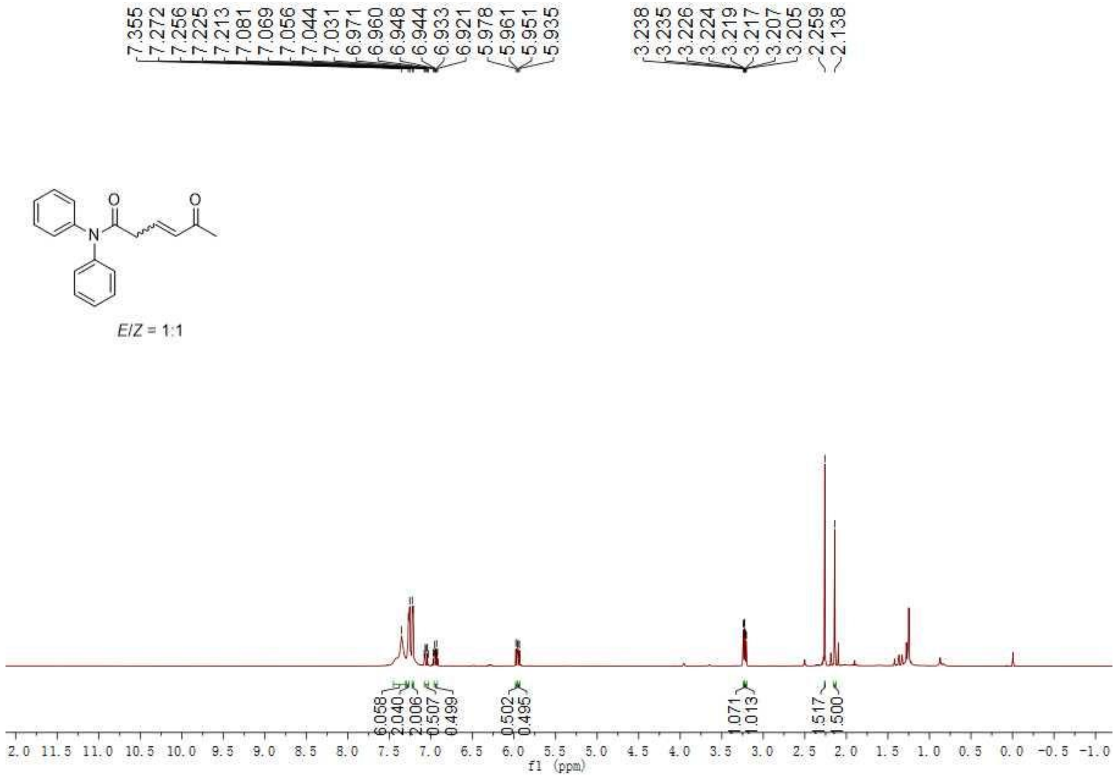

^{13}C NMR (150 MHz, $CDCl_3$) Spectrum of **103**

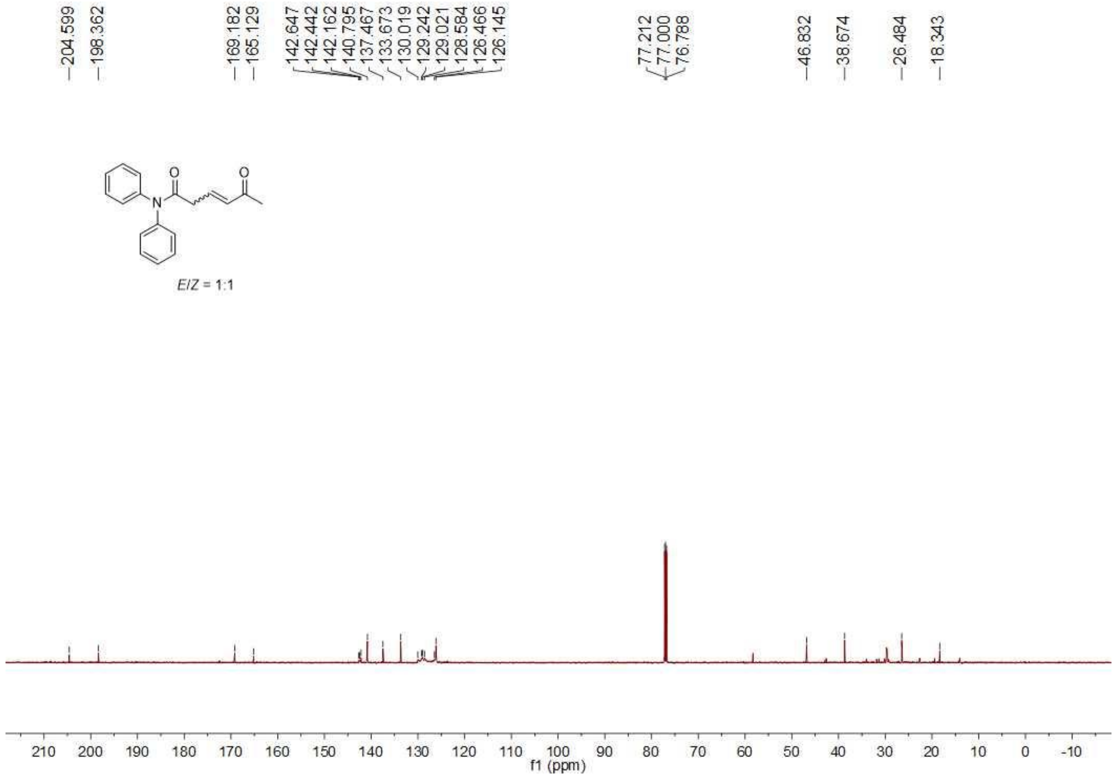

^{1}H NMR (600 MHz, $CDCl_3$) Spectrum of **104**

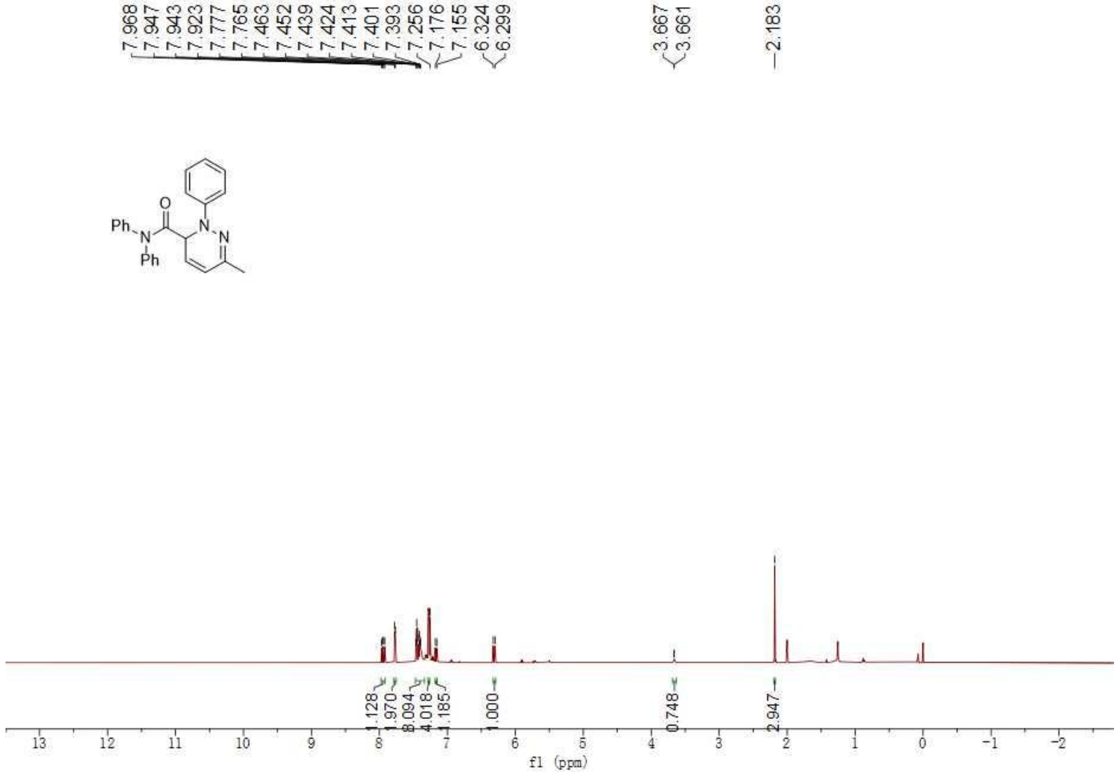

^{13}C NMR (150 MHz, $CDCl_3$) Spectrum of **104**

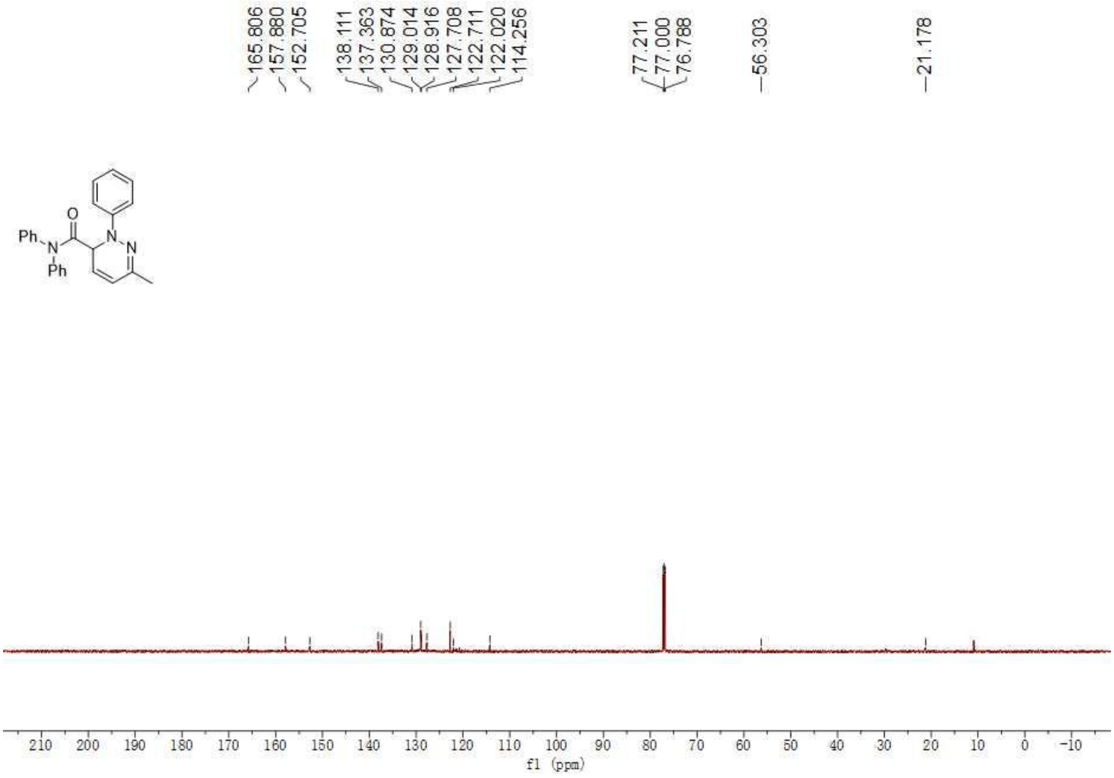

^{1}H NMR (600 MHz, $CDCl_3$) Spectrum of **105**

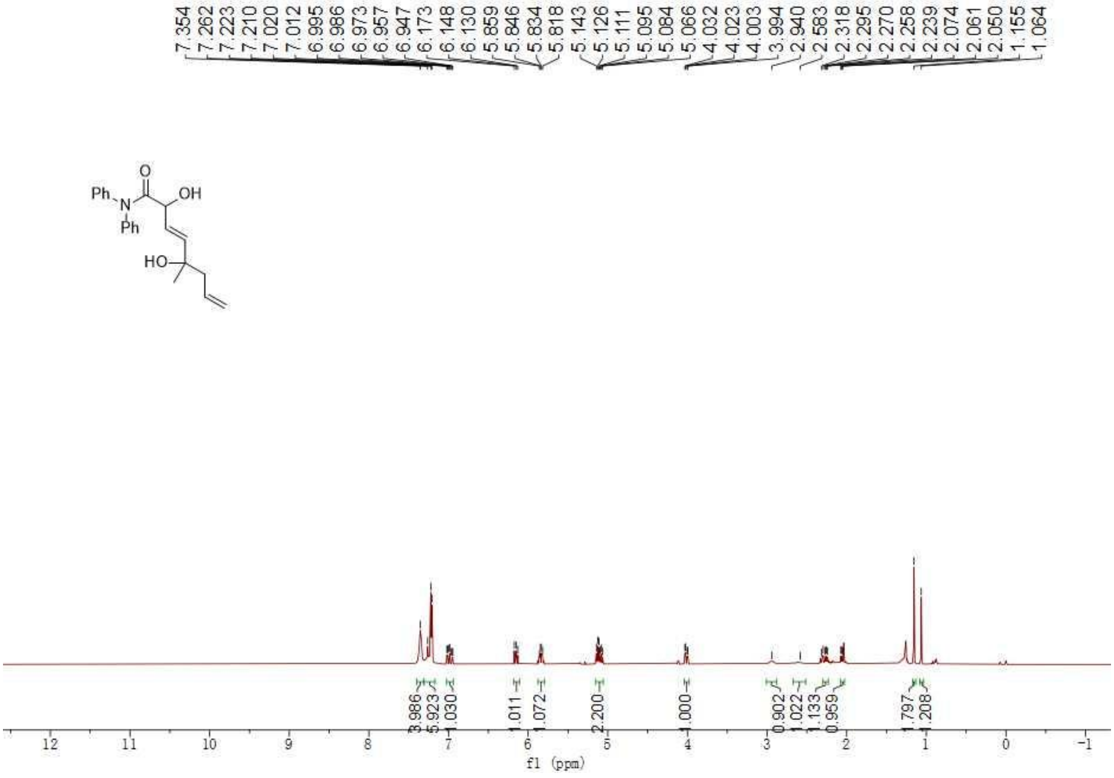

^{13}C NMR (150 MHz, $CDCl_3$) Spectrum of **105**

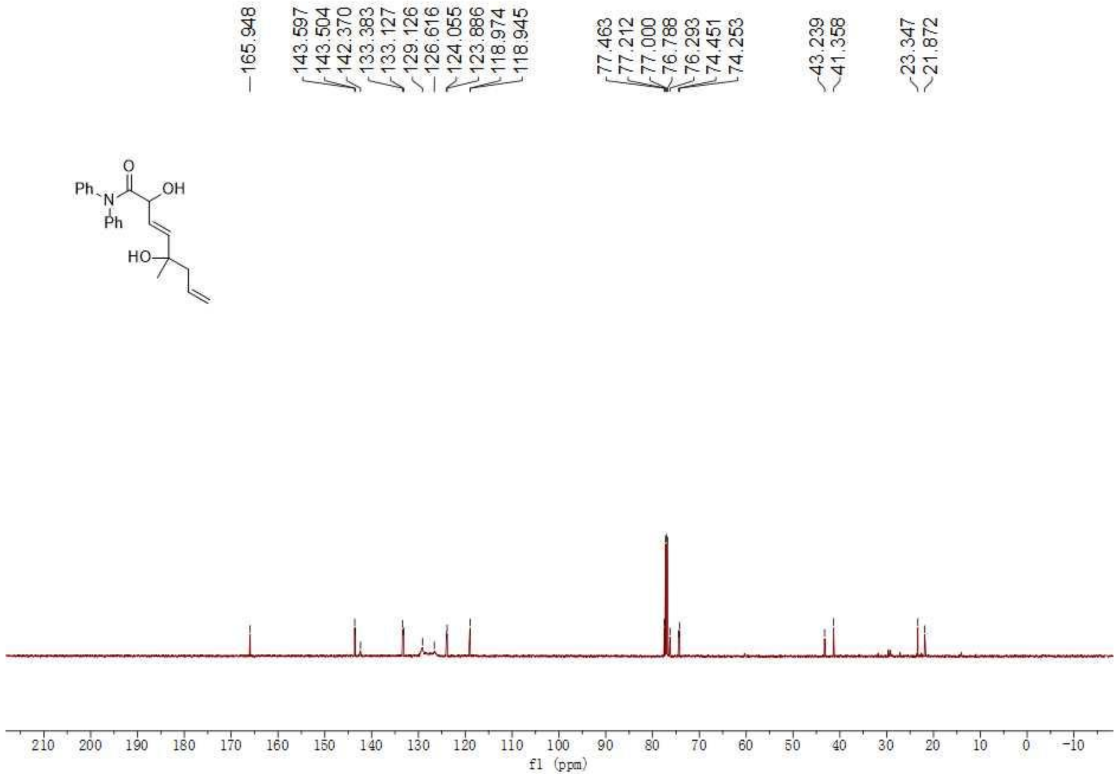

^{1}H NMR (600 MHz, $CDCl_3$) Spectrum of **106**

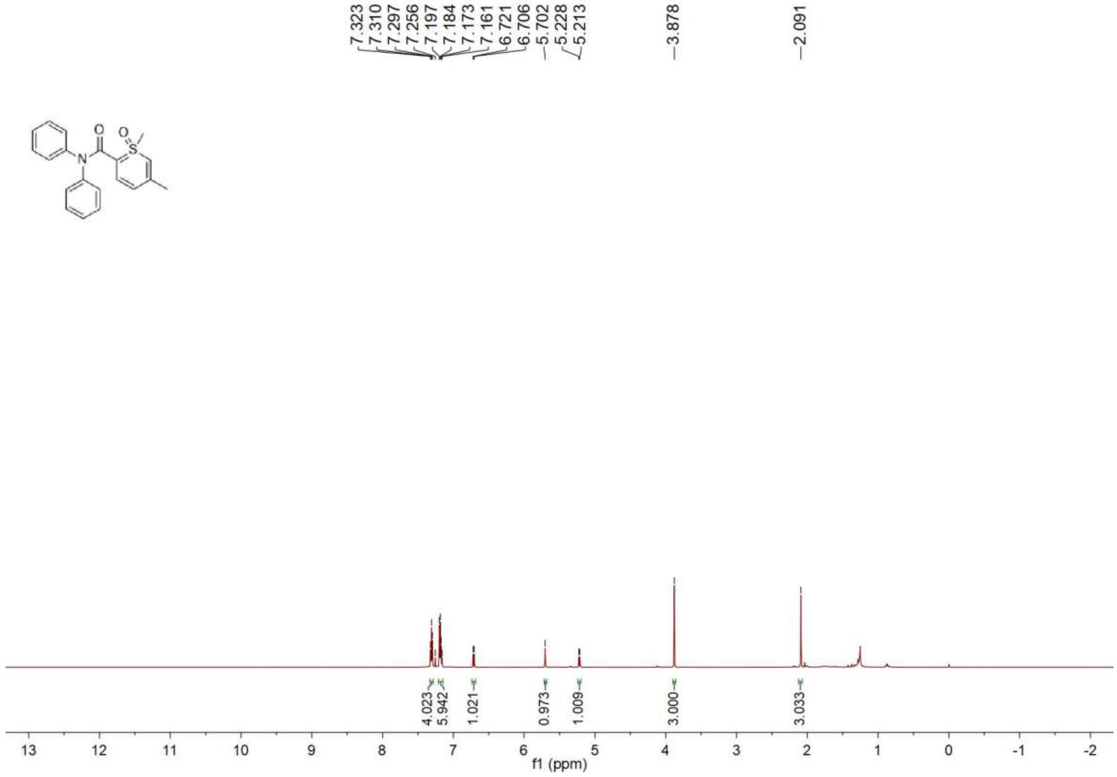

^{13}C NMR (150 MHz, $CDCl_3$) Spectrum of **106**

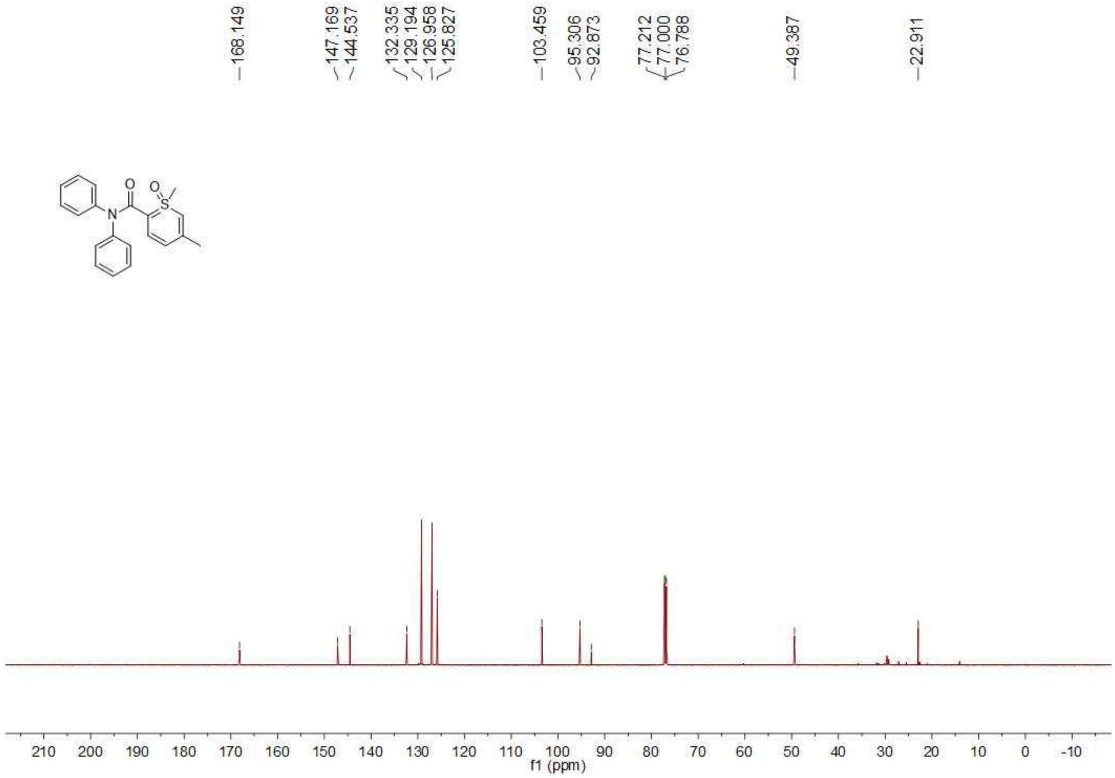

^{1}H NMR (600 MHz, $CDCl_3$) Spectrum of **107**

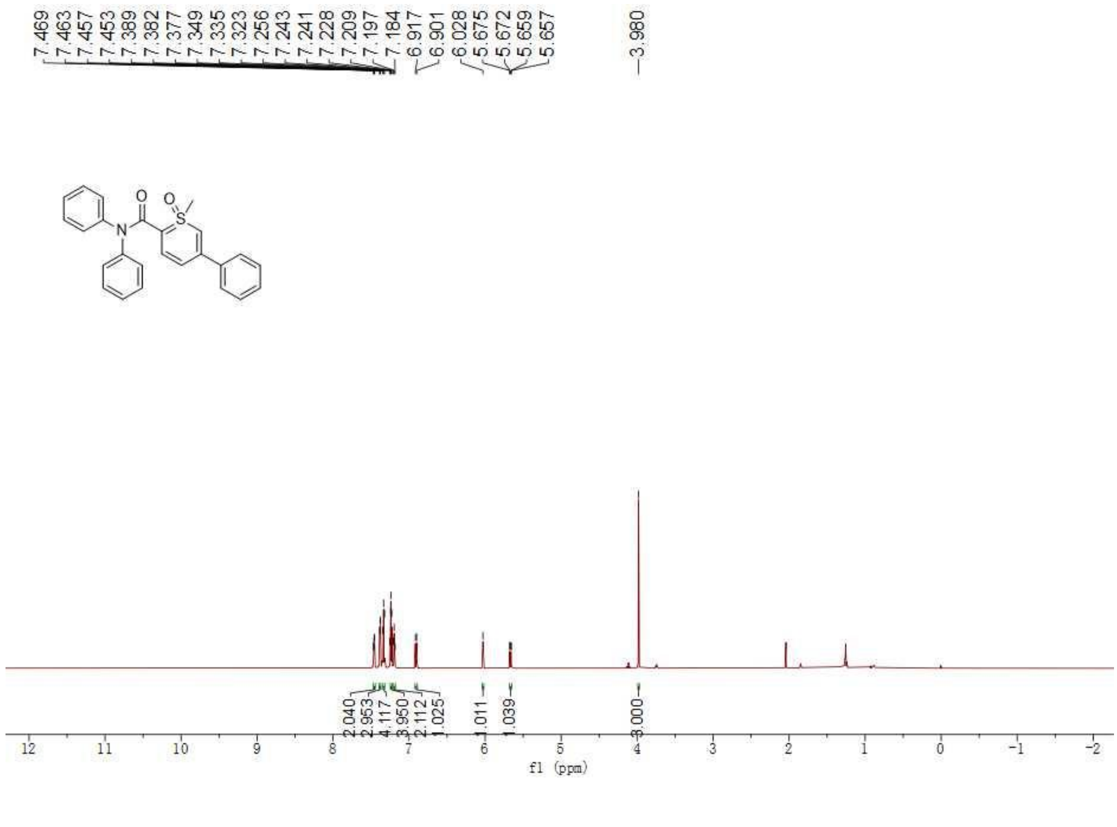

^{13}C NMR (150 MHz, $CDCl_3$) Spectrum of **107**

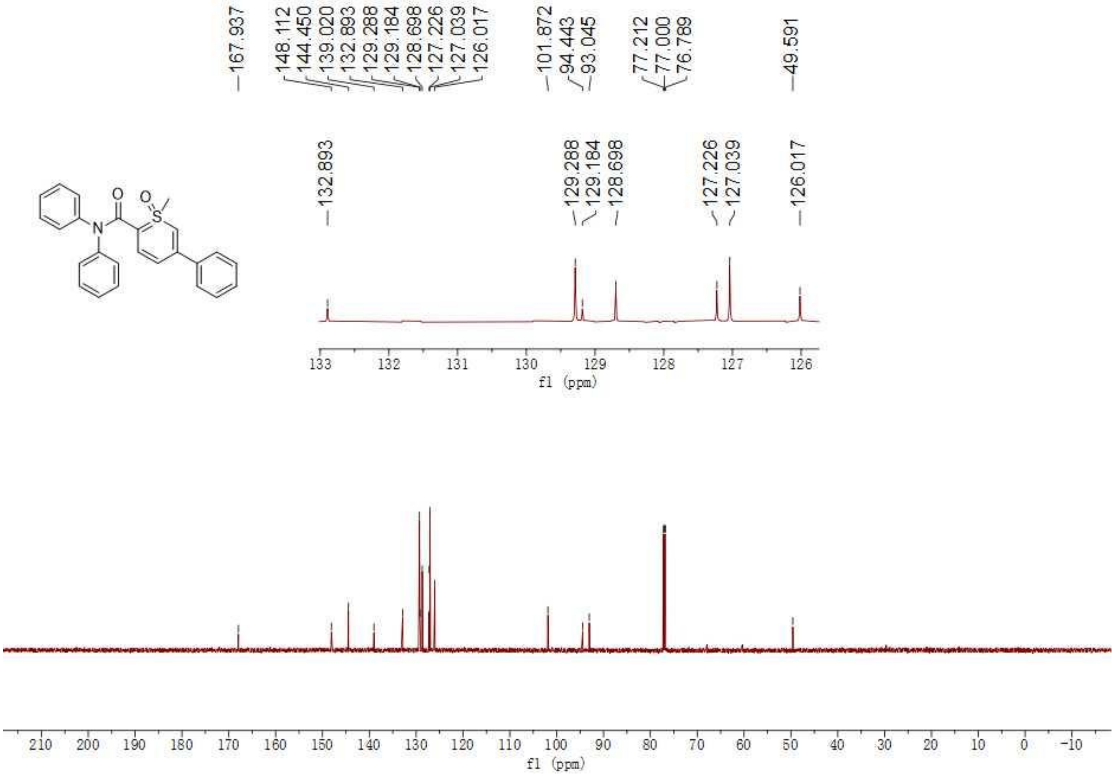

^{1}H NMR (600 MHz, $CDCl_3$) Spectrum of **108**

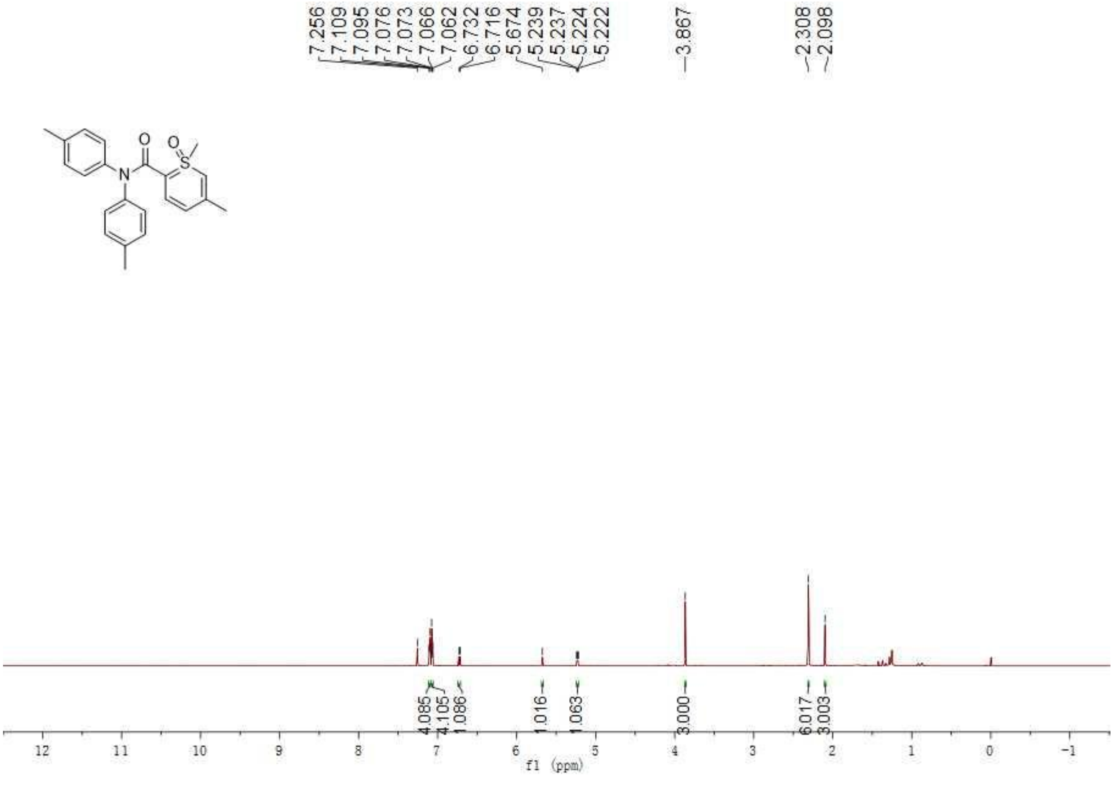

^{13}C NMR (150 MHz, $CDCl_3$) Spectrum of **108**

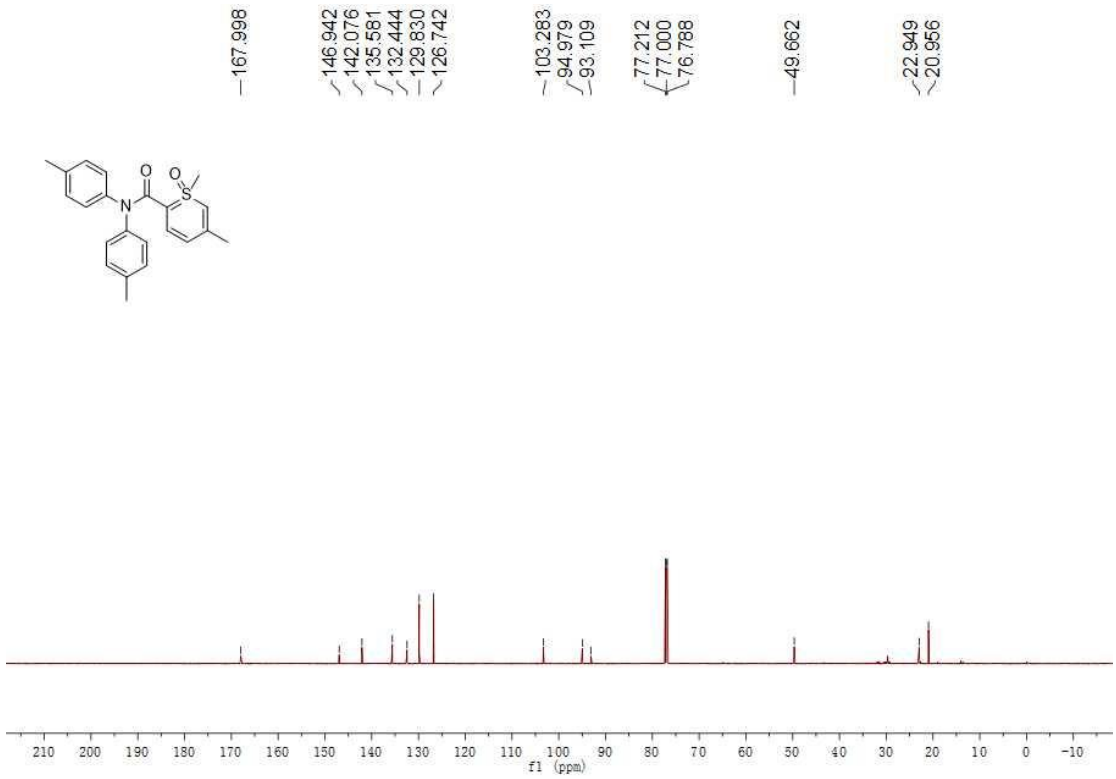

^{1}H NMR (600 MHz, $CDCl_3$) Spectrum of **109**

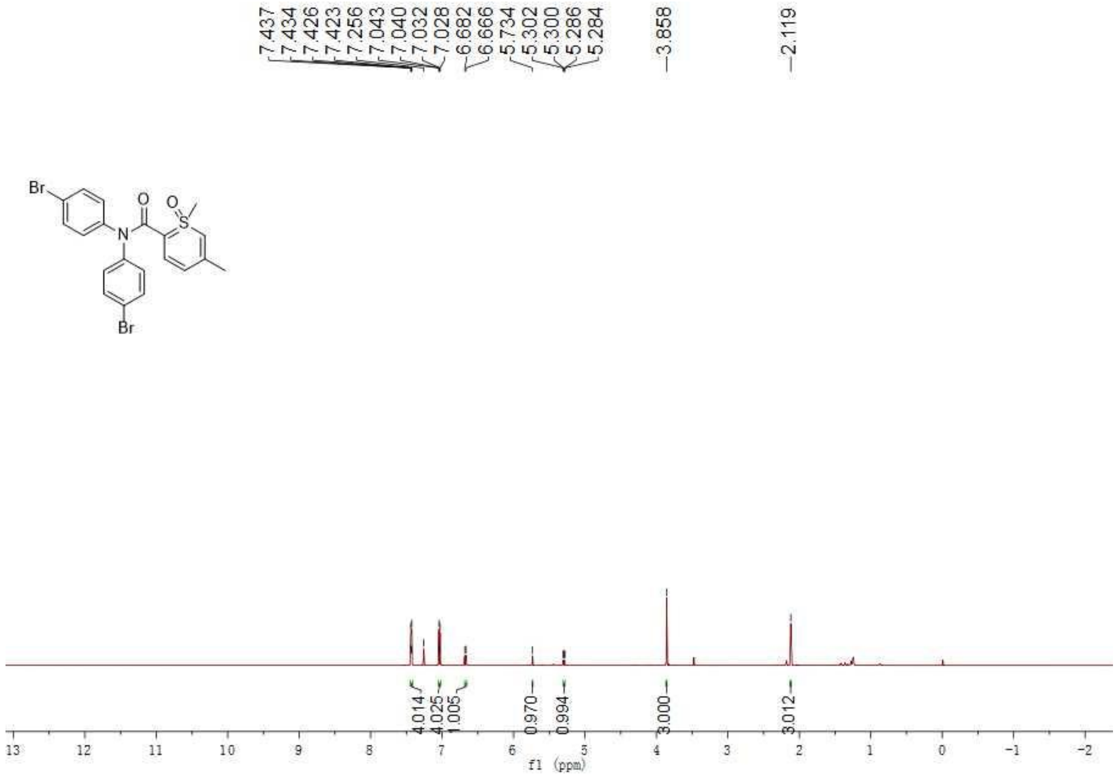

^{13}C NMR (150 MHz, $CDCl_3$) Spectrum of **109**

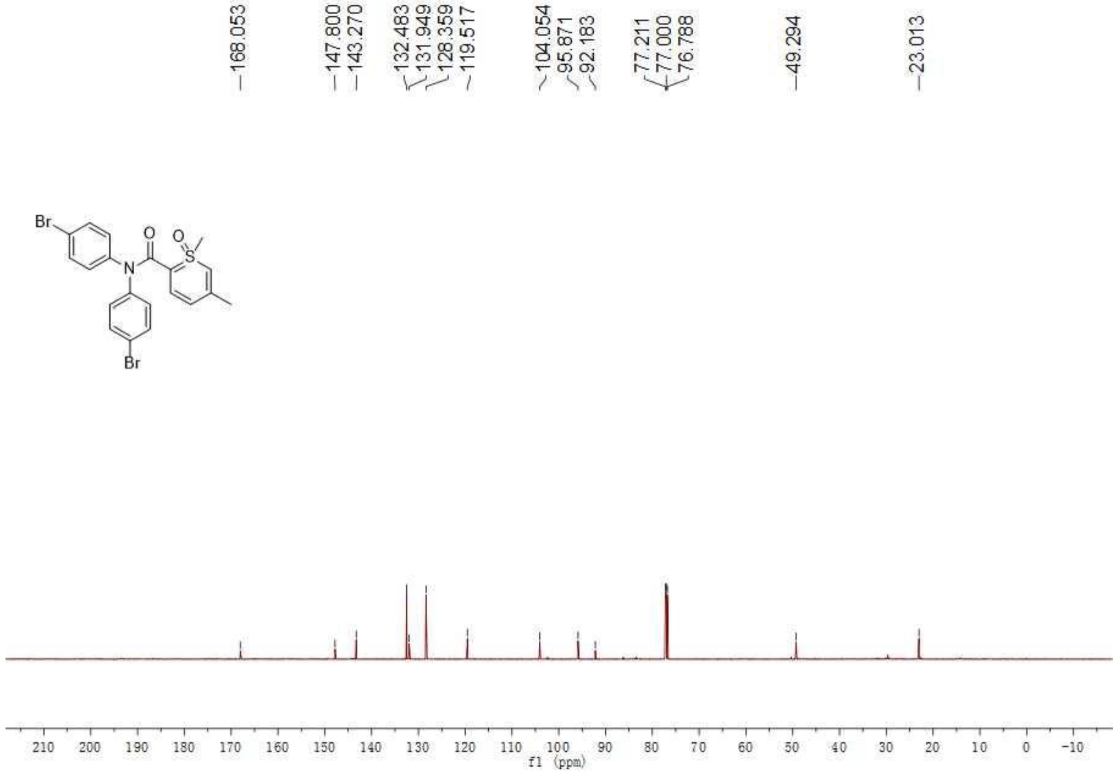

^{1}H NMR (600 MHz, $CDCl_3$) Spectrum of **110**

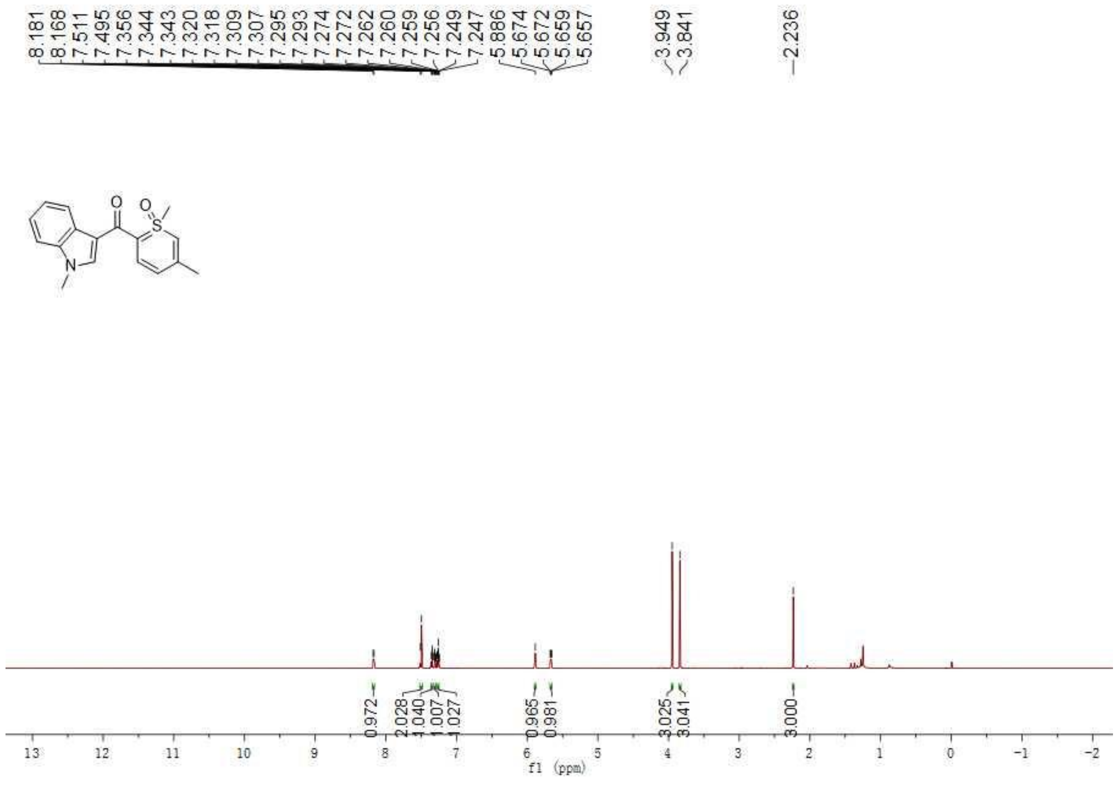

^{13}C NMR (150 MHz, $CDCl_3$) Spectrum of **110**

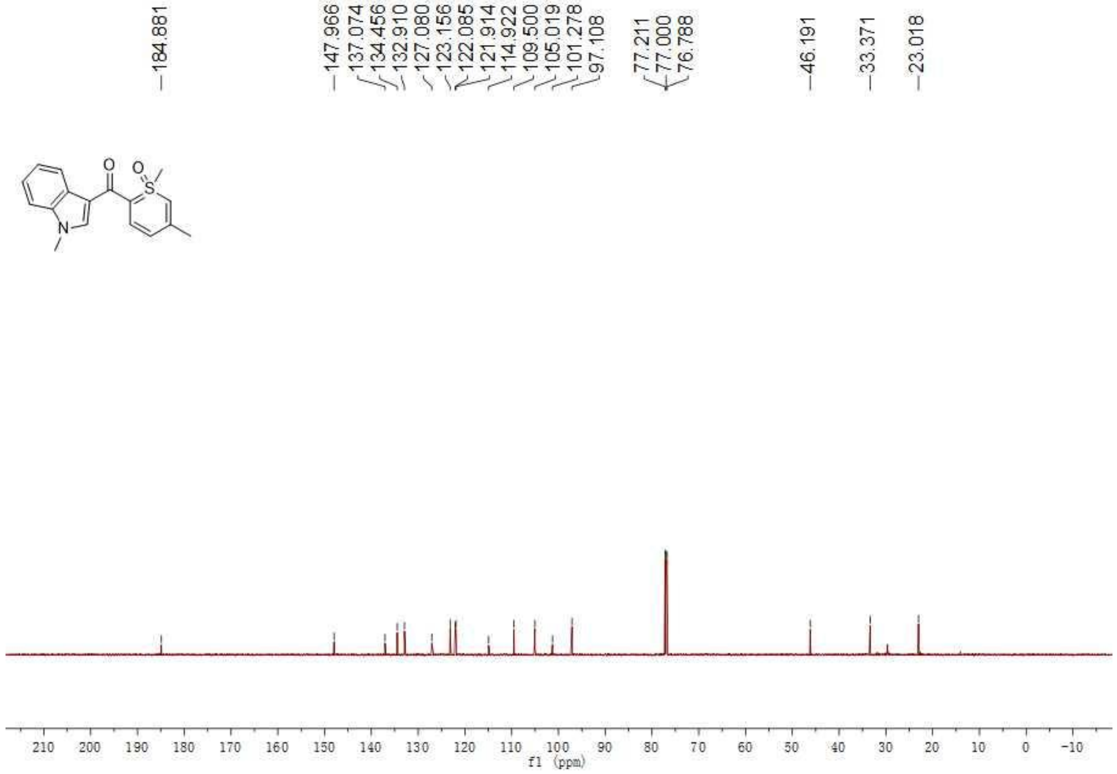

^{1}H NMR (600 MHz, $CDCl_3$) Spectrum of **S4**

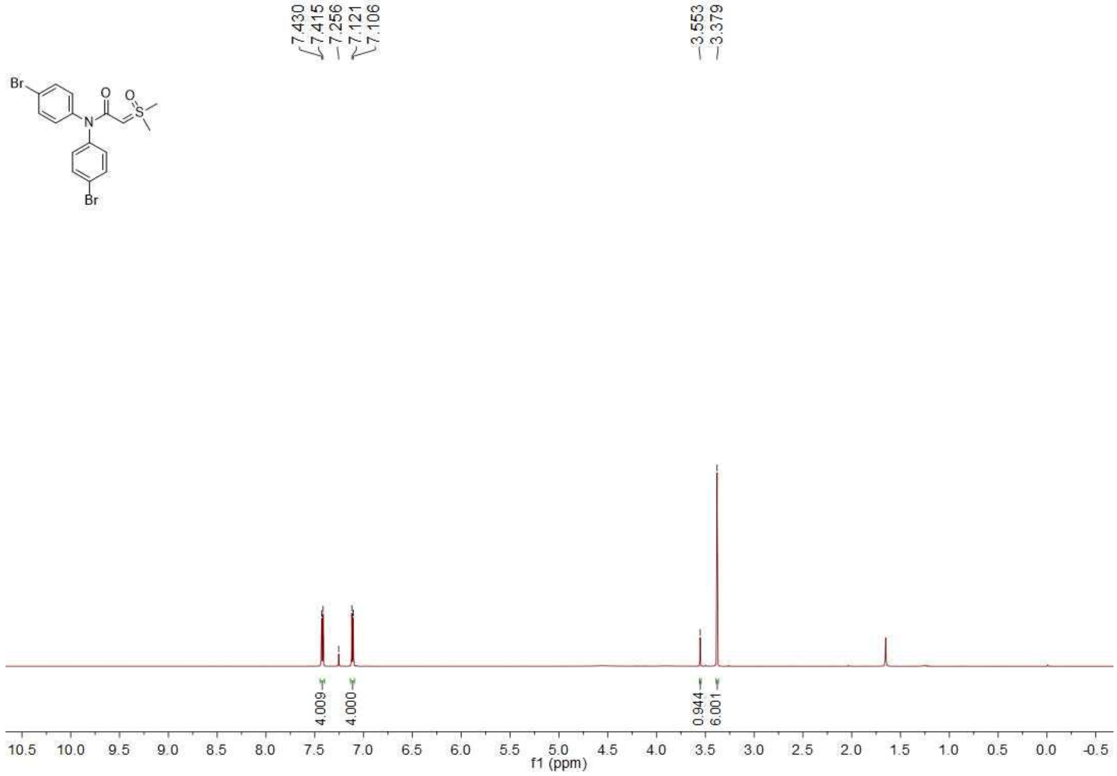

^{13}C NMR (150 MHz, $CDCl_3$) Spectrum of **S4**

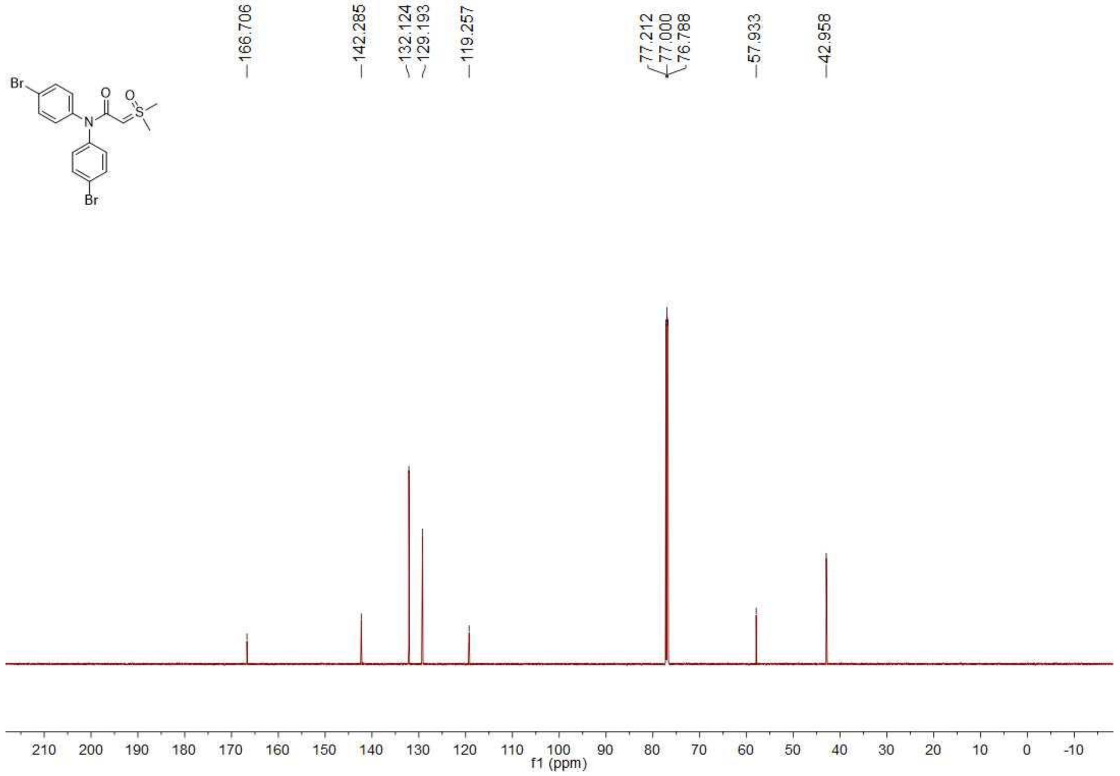

^{1}H NMR (600 MHz, $CDCl_3$) Spectrum of **S5**

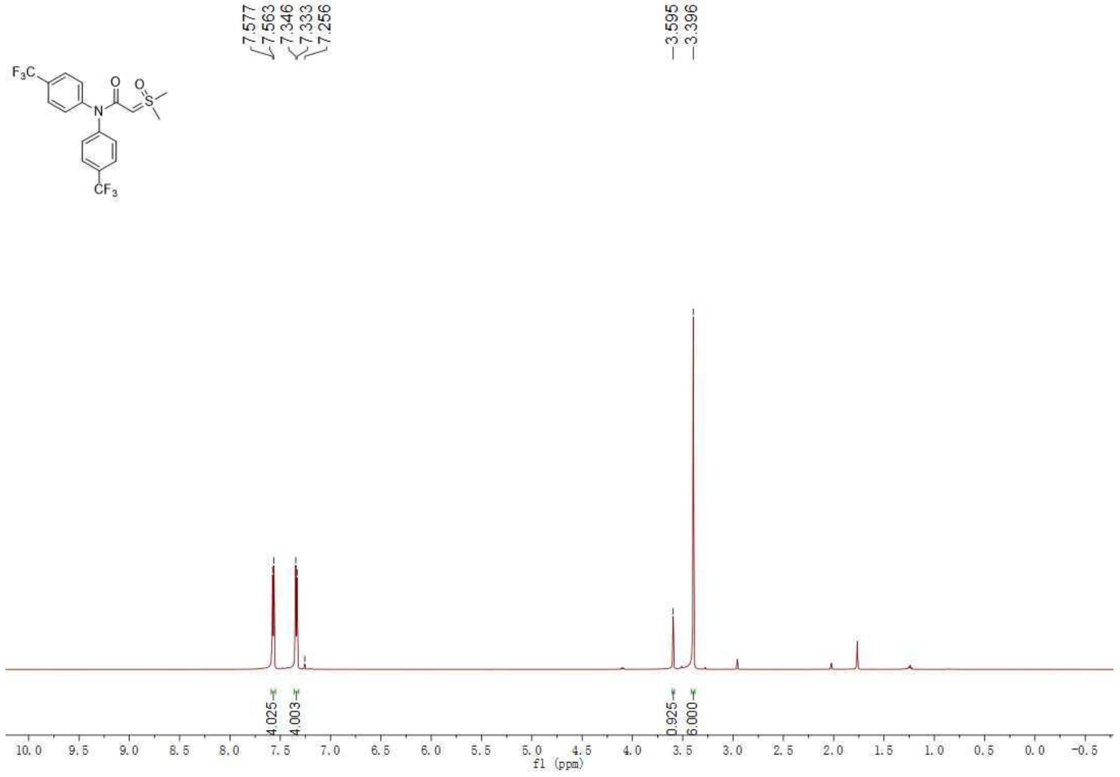

^{13}C NMR (150 MHz, $CDCl_3$) Spectrum of **S5**

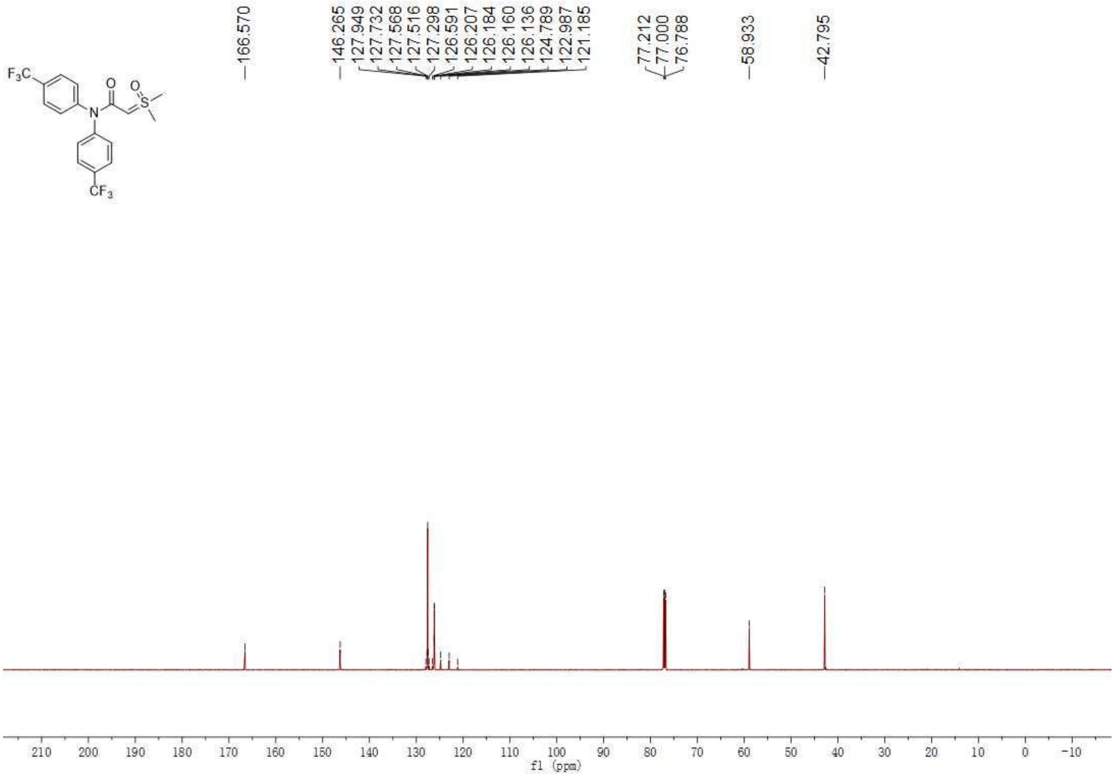

^{19}F NMR (564 MHz, $CDCl_3$) Spectrum of **S5**

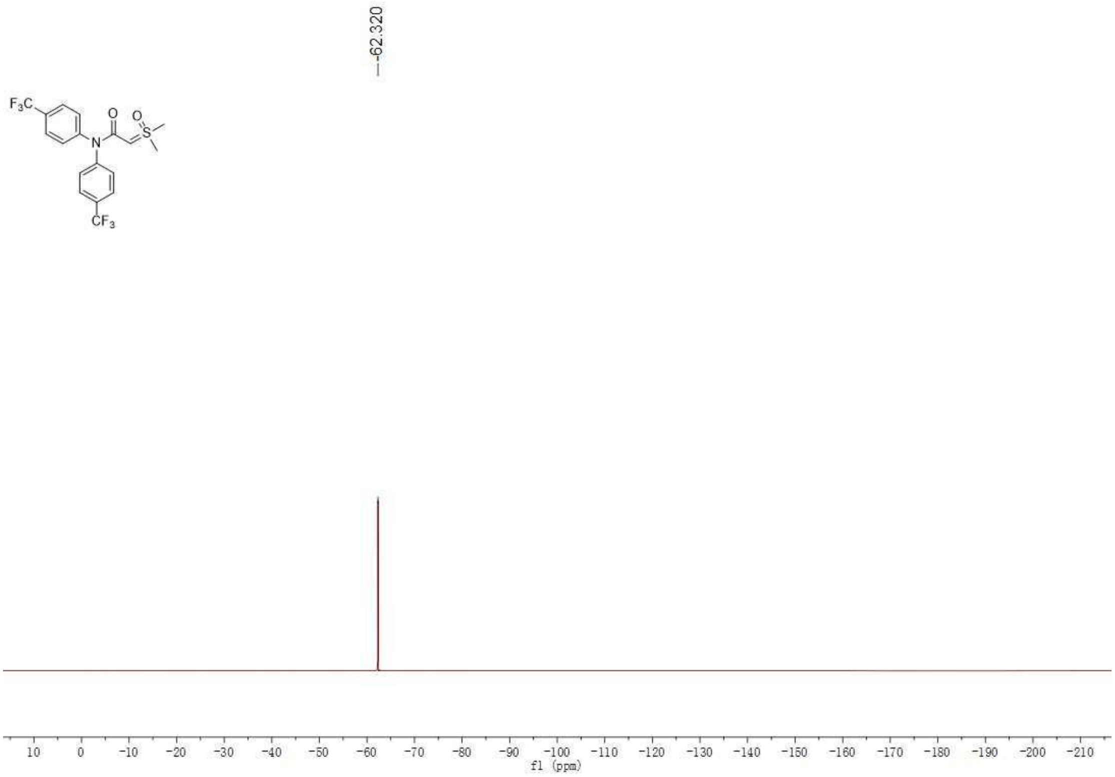

^{1}H NMR (600 MHz, $CDCl_3$) Spectrum of **S7**

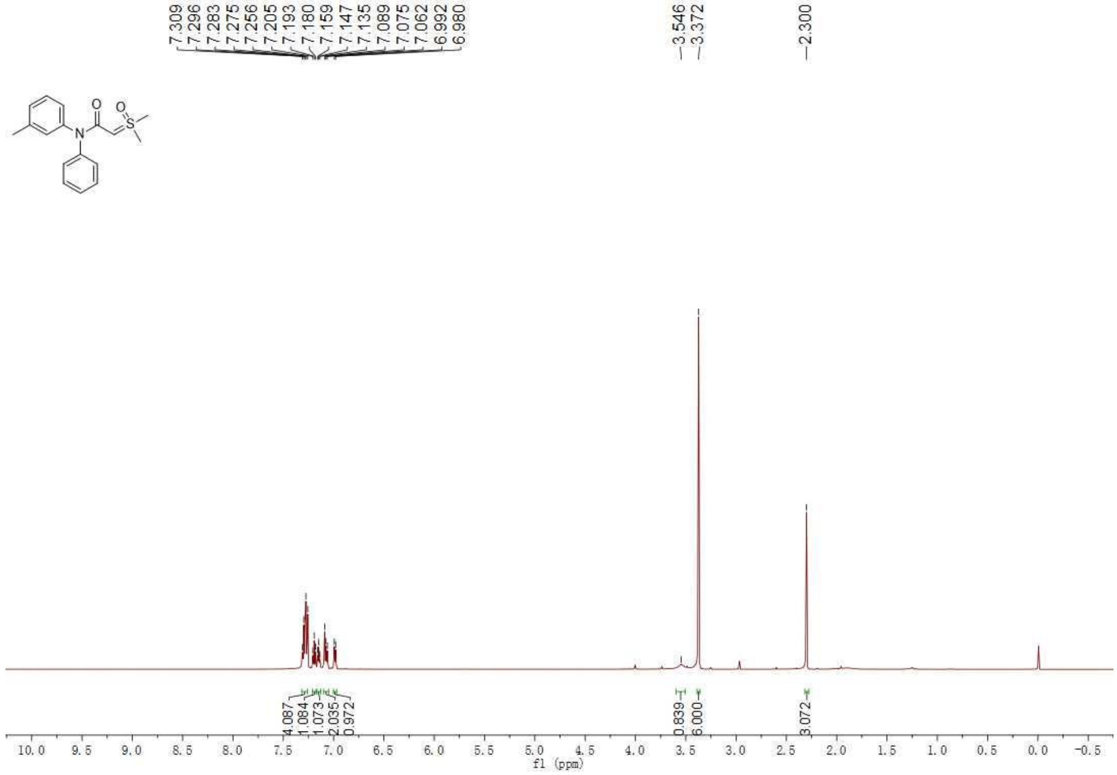

^{13}C NMR (150 MHz, $CDCl_3$) Spectrum of **S7**

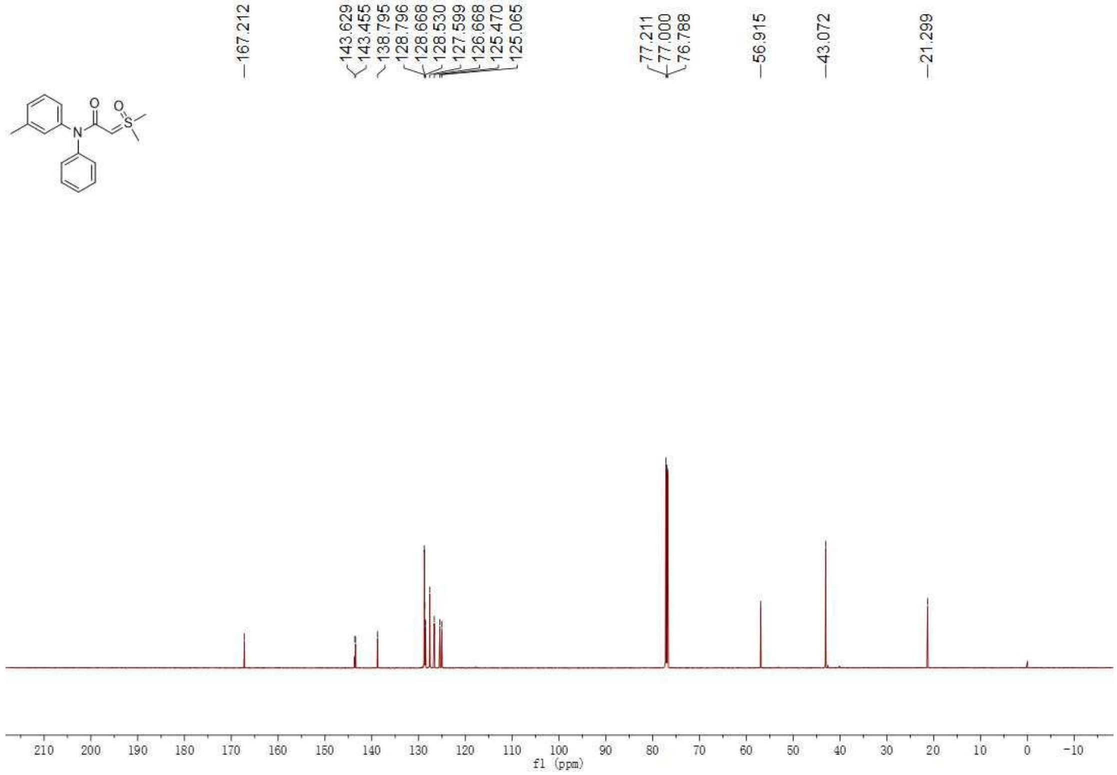

^{1}H NMR (600 MHz, $CDCl_3$) Spectrum of **S8**

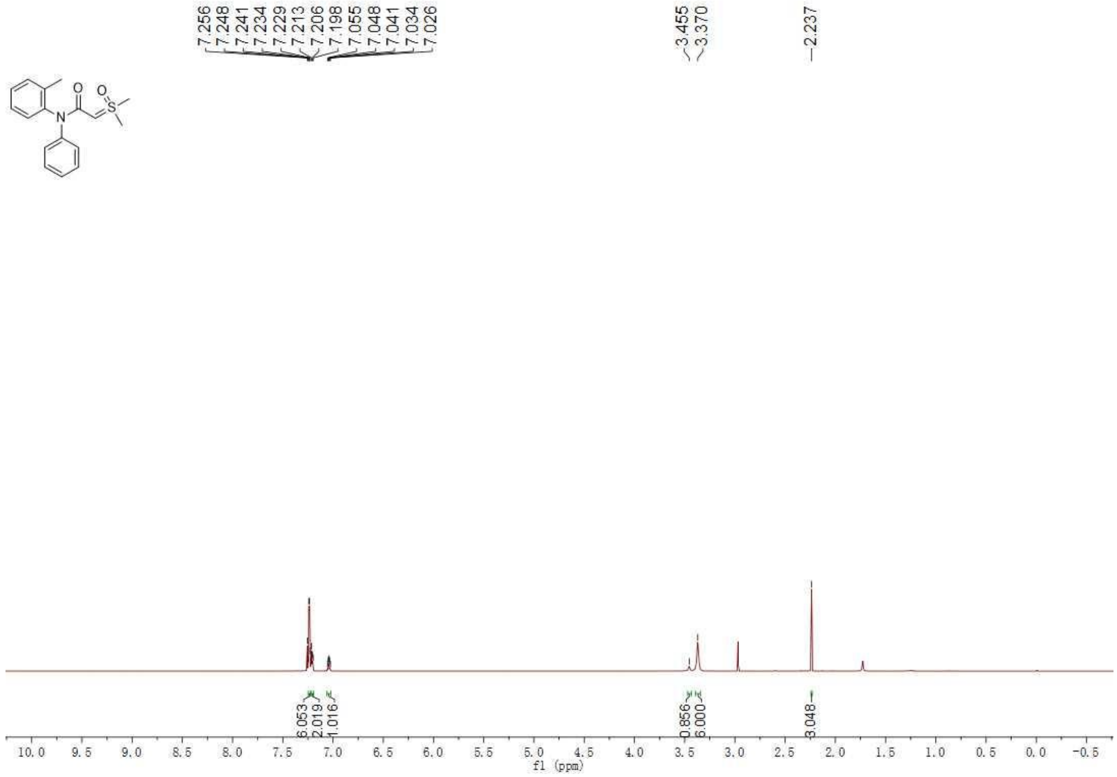

^{13}C NMR (150 MHz, $CDCl_3$) Spectrum of **S8**

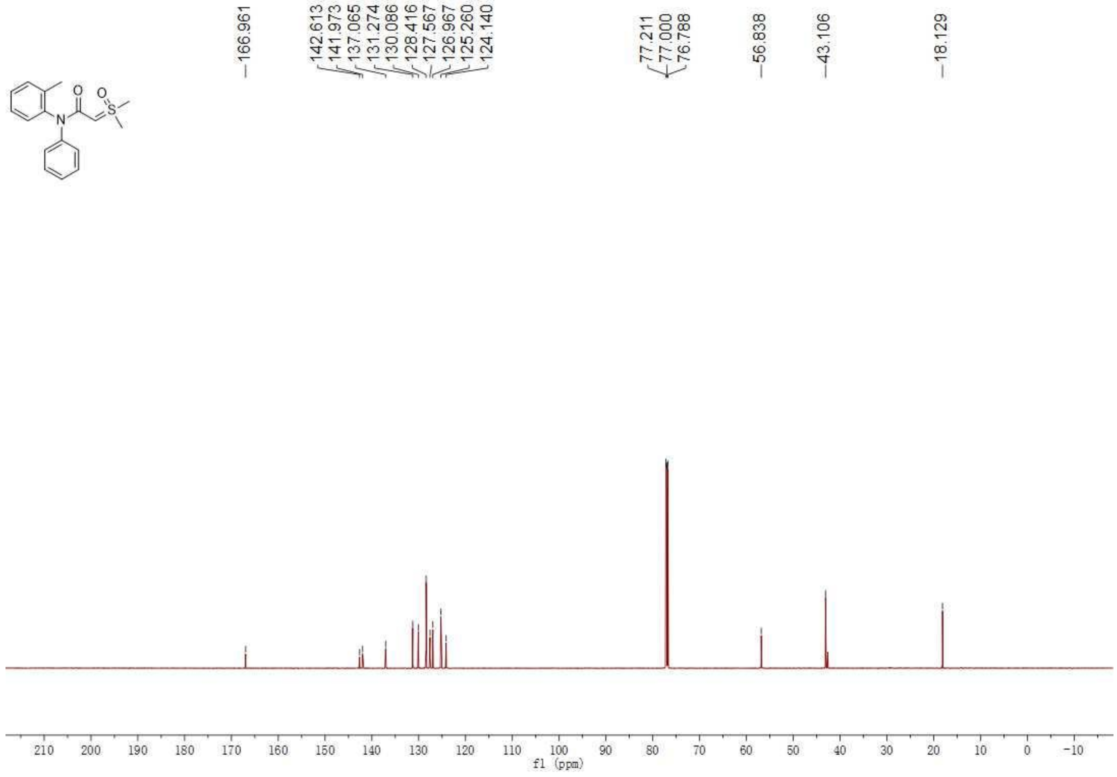

^{1}H NMR (600 MHz, $CDCl_3$) Spectrum of **S9**

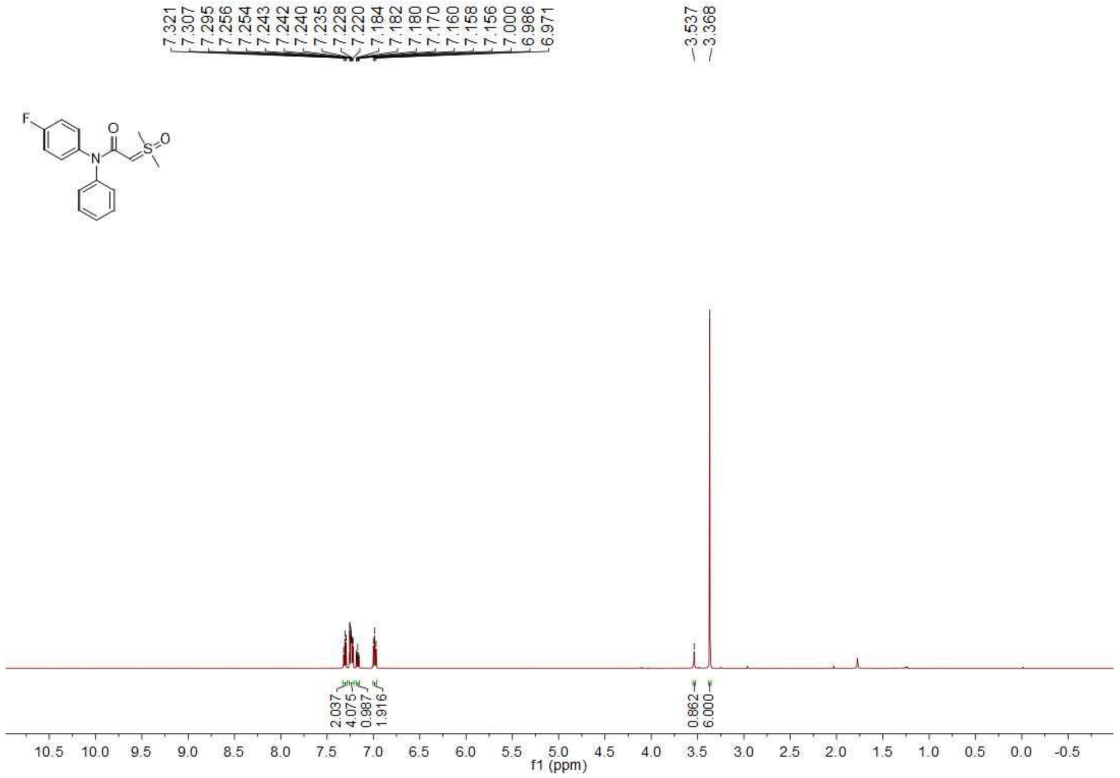

^{13}C NMR (150 MHz, $CDCl_3$) Spectrum of **S9**

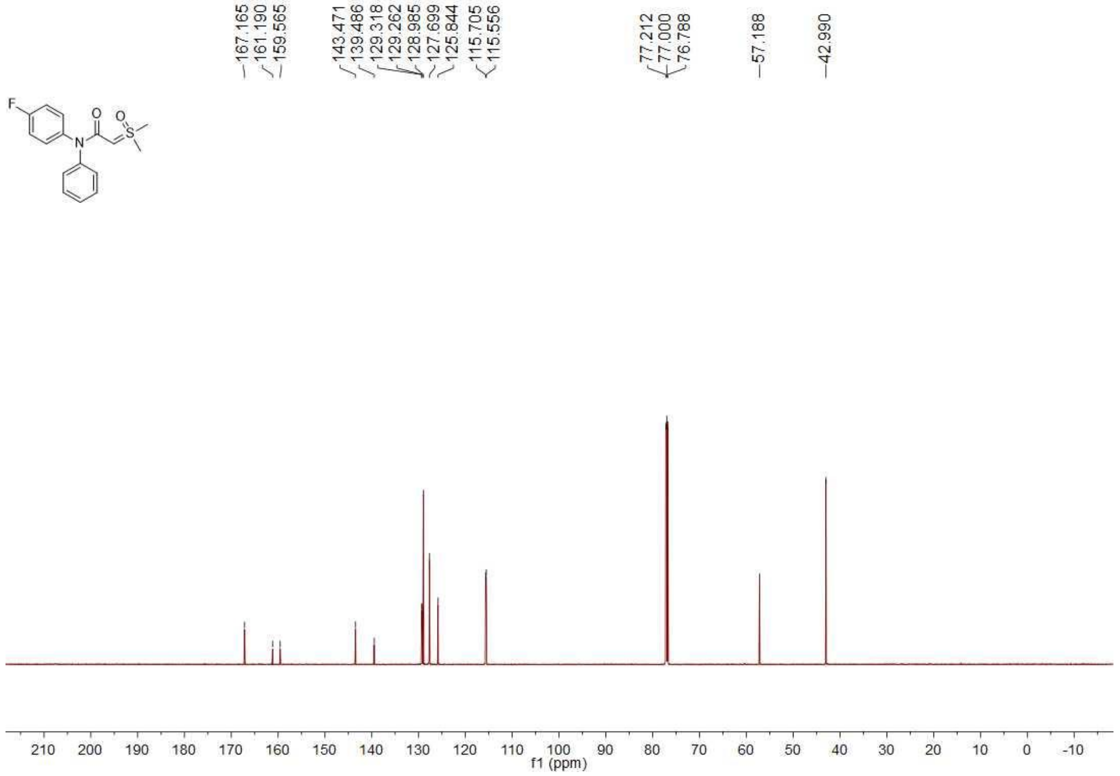

^{19}F NMR (564 MHz, $CDCl_3$) Spectrum of **S9**

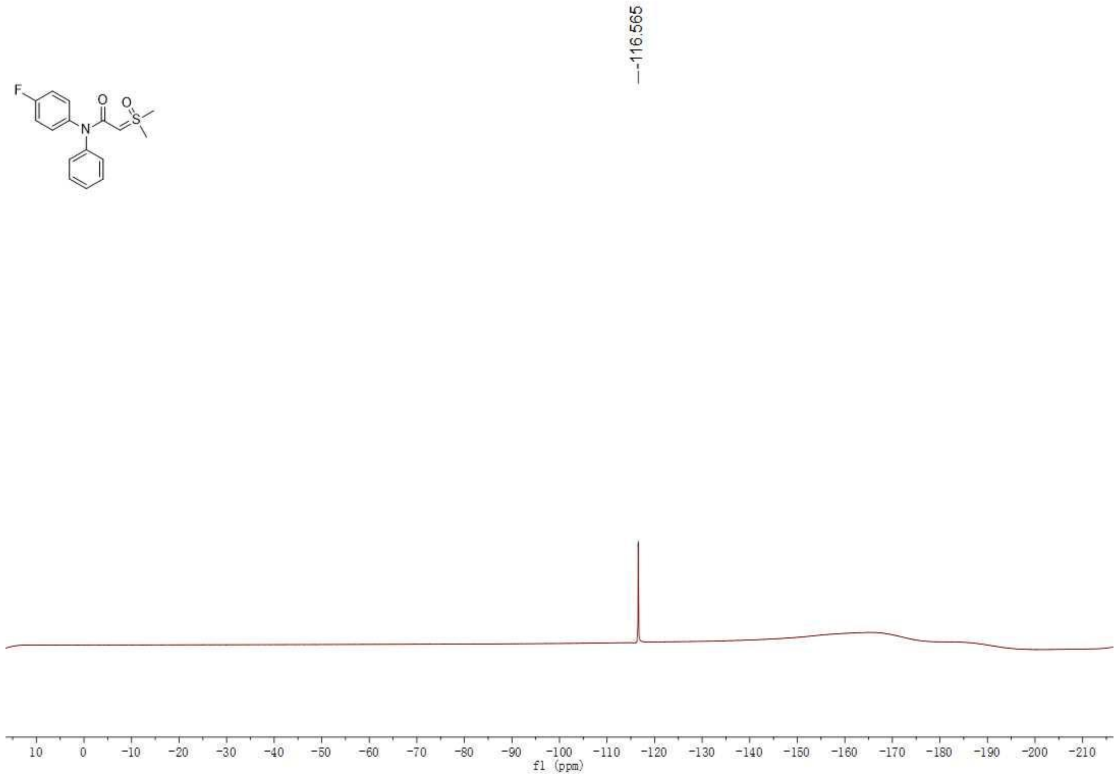

^{1}H NMR (600 MHz, $CDCl_3$) Spectrum of **S10**

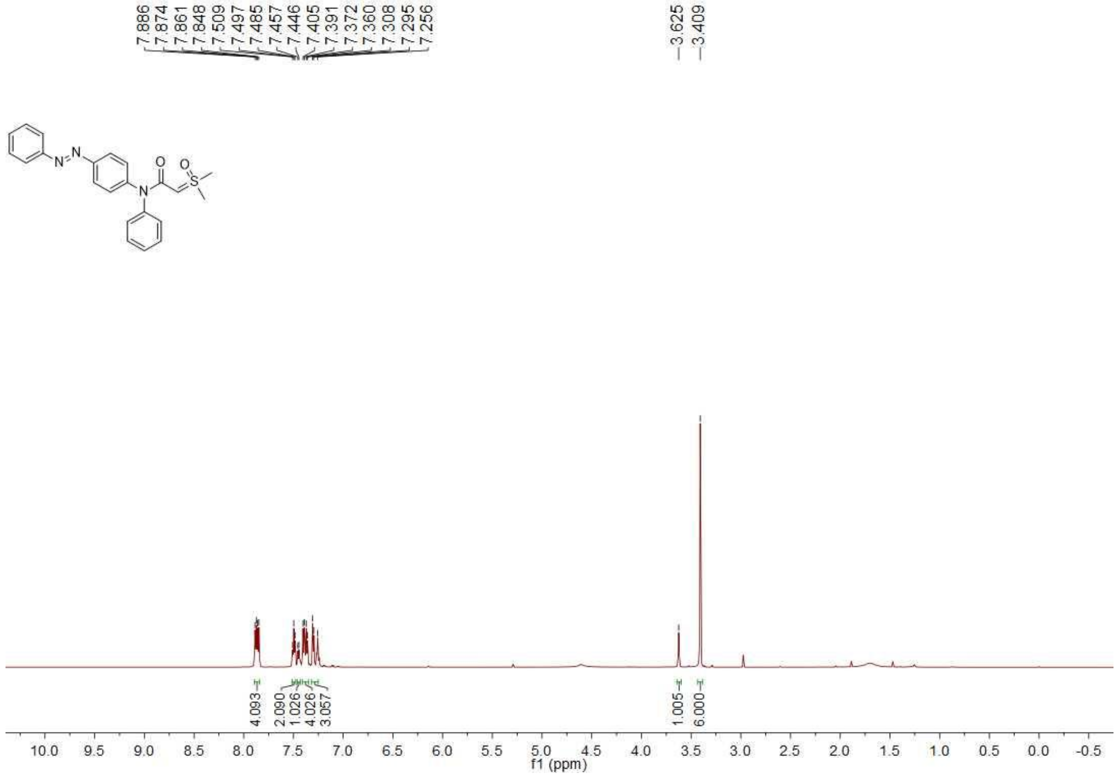

^{13}C NMR (150 MHz, $CDCl_3$) Spectrum of **S10**

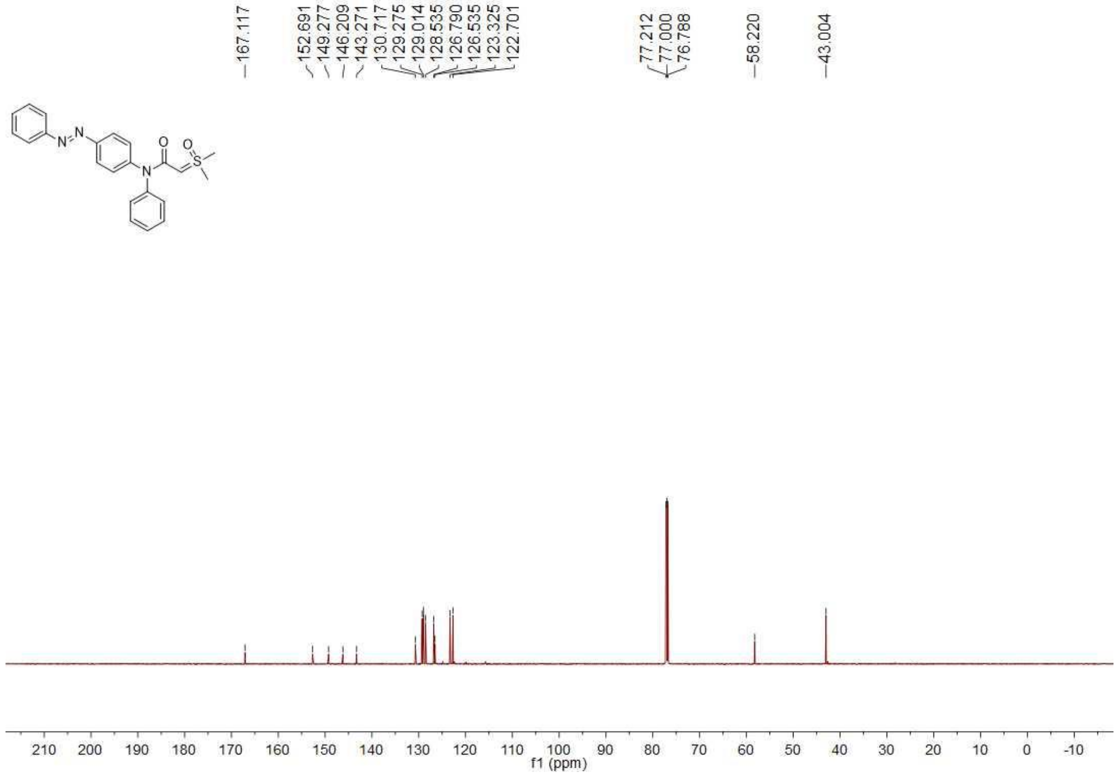

^{1}H NMR (600 MHz, $CDCl_3$) Spectrum of **S11**

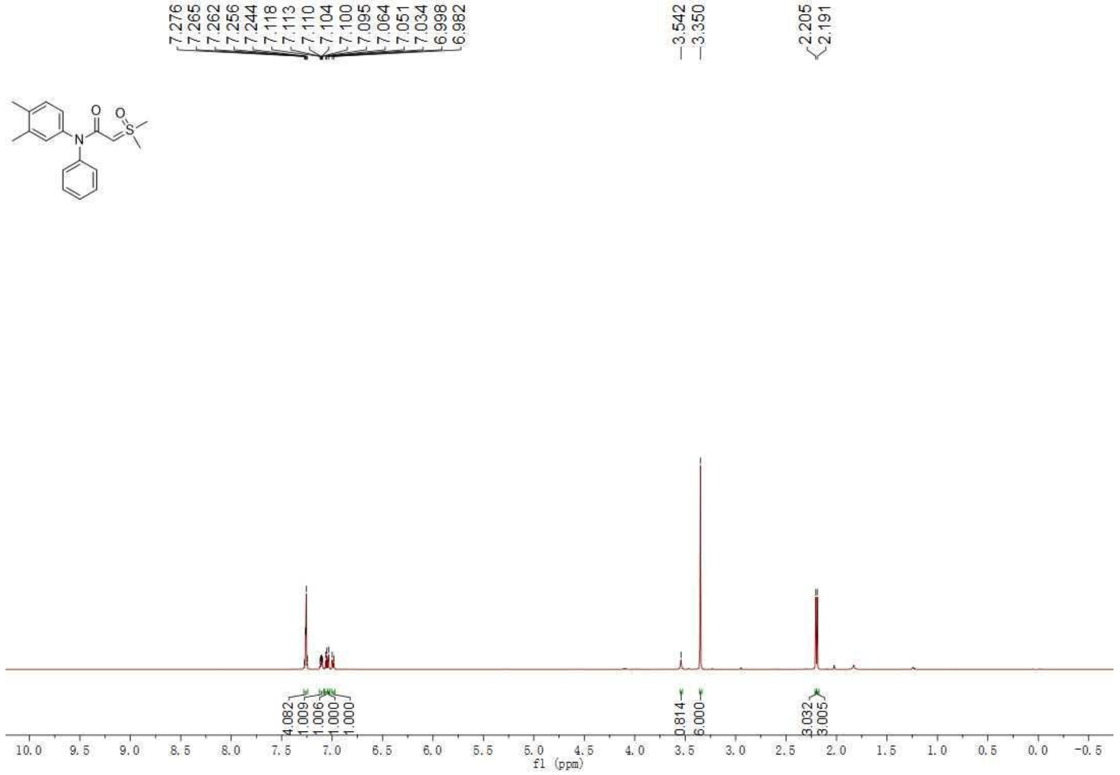

^{13}C NMR (150 MHz, $CDCl_3$) Spectrum of **S11**

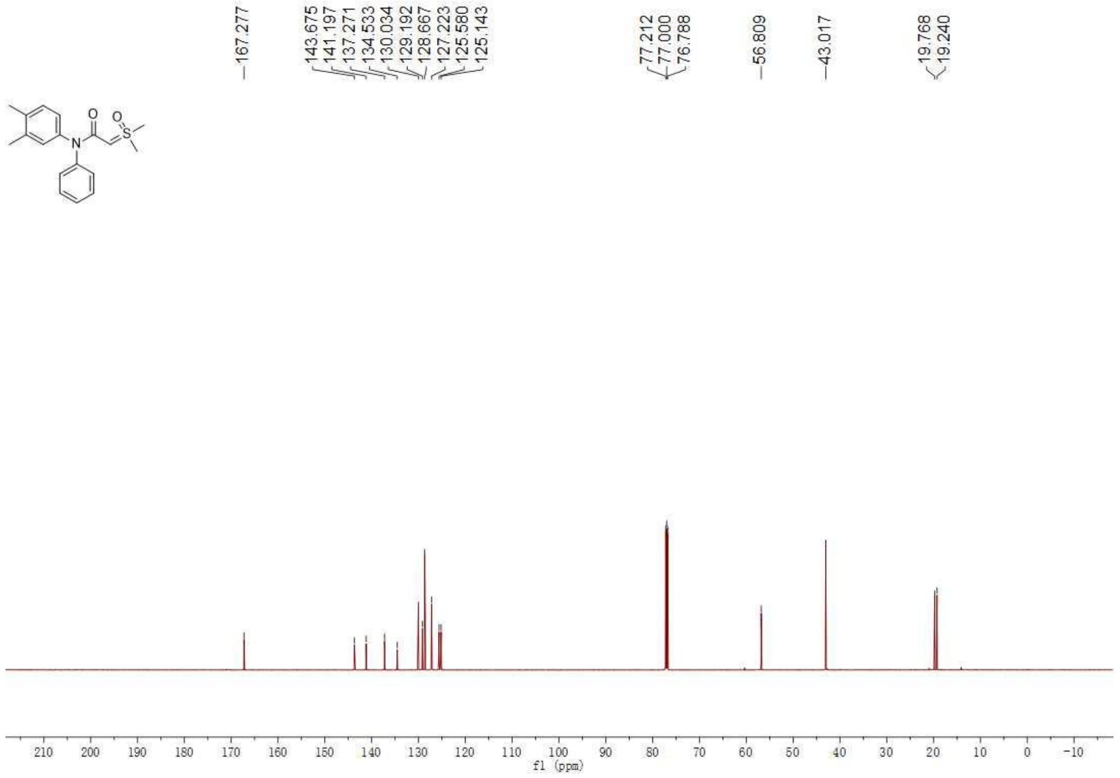

^{1}H NMR (600 MHz, $CDCl_3$) Spectrum of **S13**

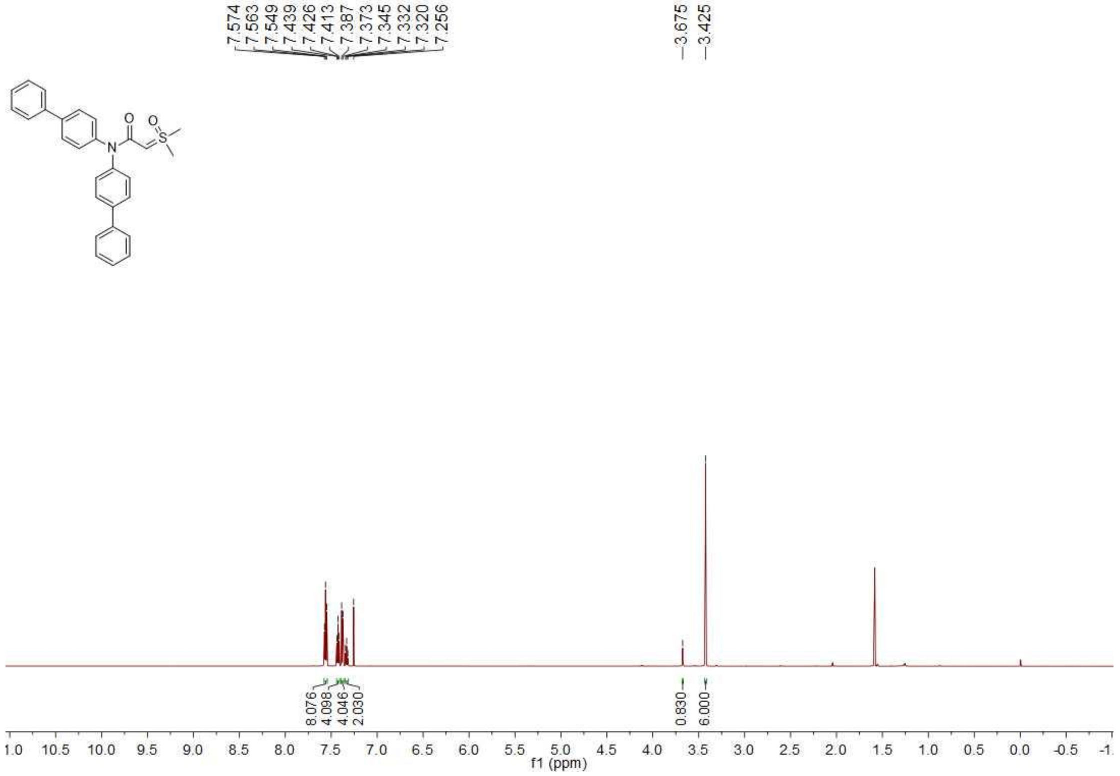

^{13}C NMR (150 MHz, $CDCl_3$) Spectrum of **S13**

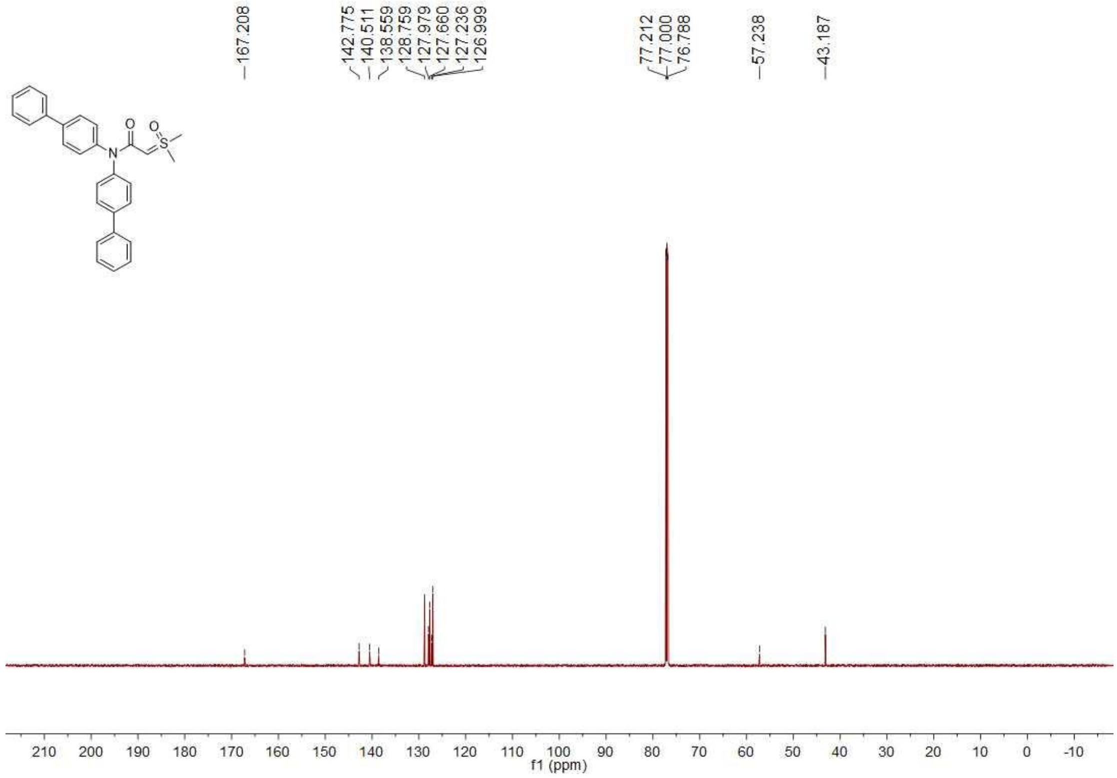

^{1}H NMR (600 MHz, $CDCl_3$) Spectrum of **S18**

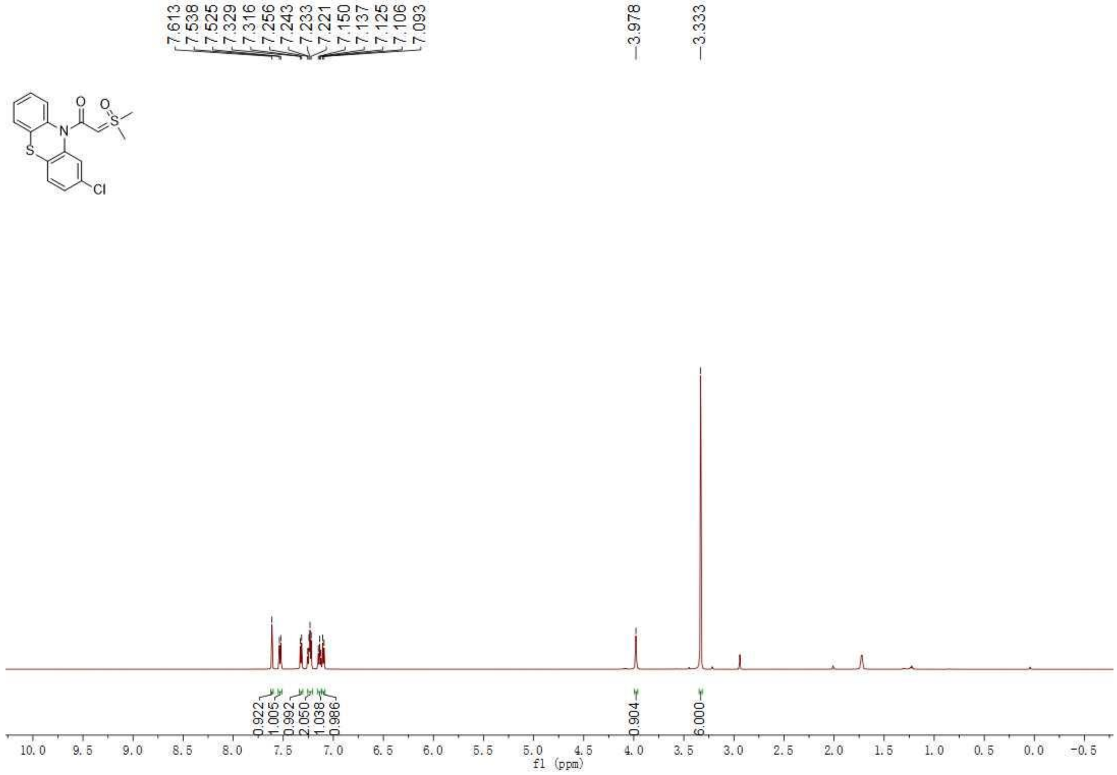

^{13}C NMR (150 MHz, $CDCl_3$) Spectrum of **S18**

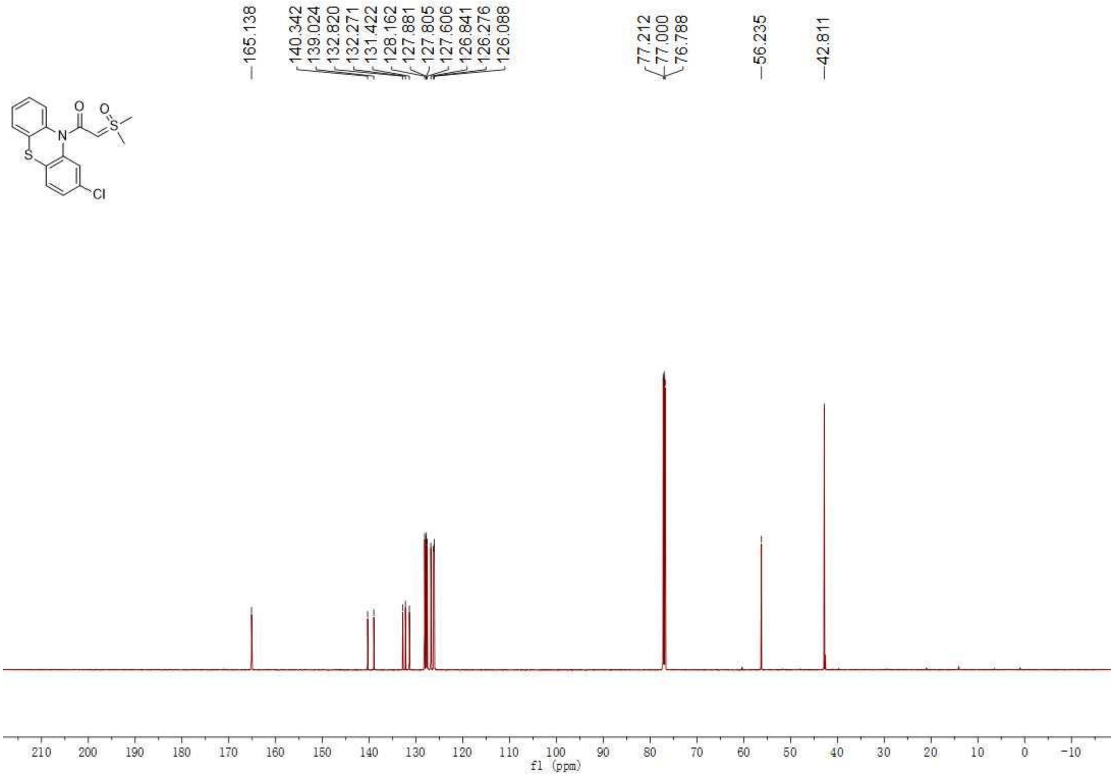

^{1}H NMR (600 MHz, DMSO-d_6) Spectrum of **S19**

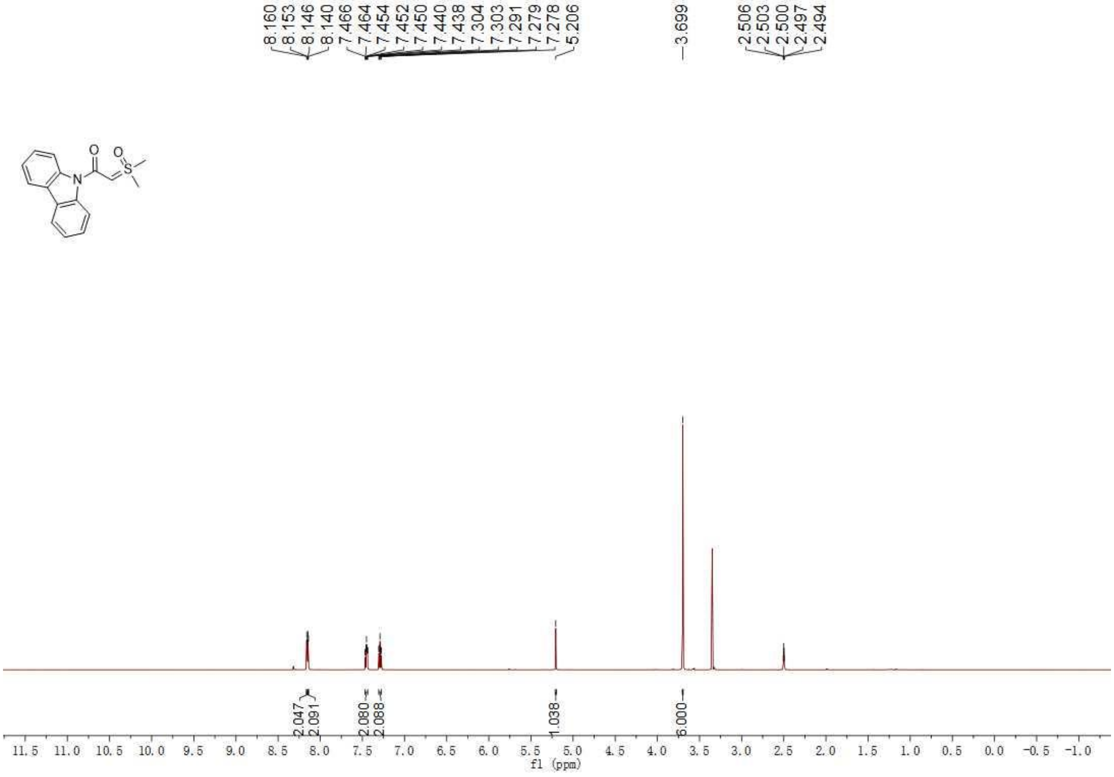

^{13}C NMR (150 MHz, DMSO-d_6) Spectrum of **S19**

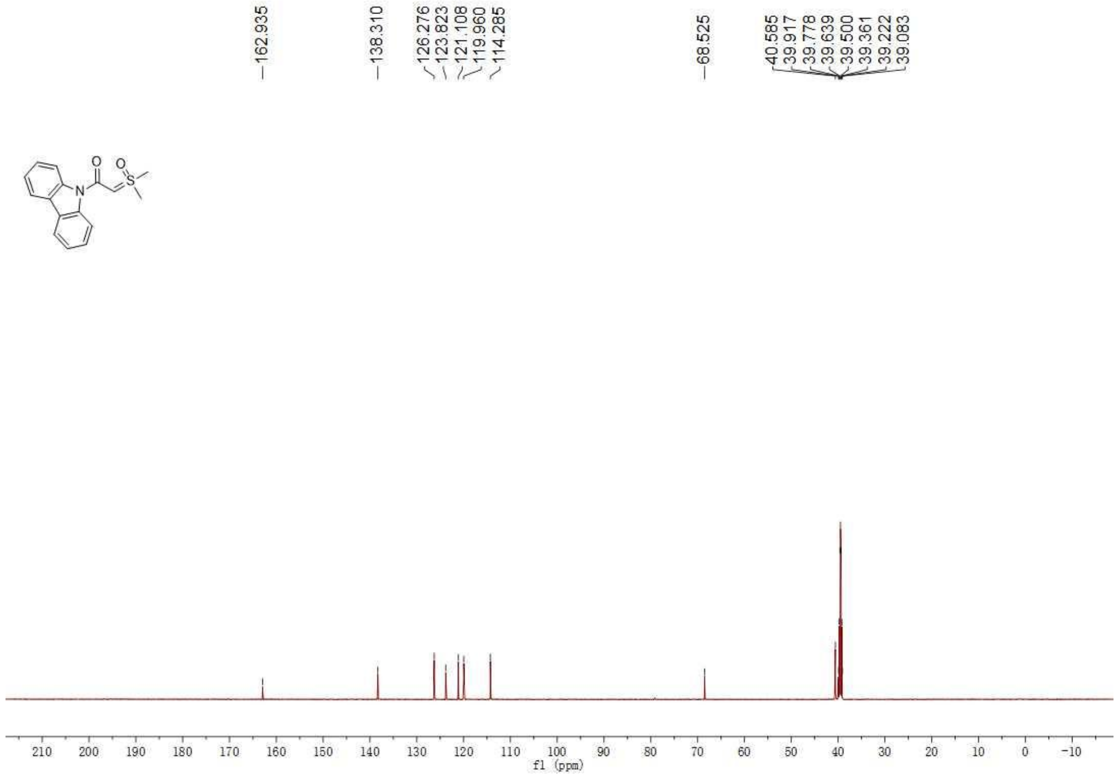

^{1}H NMR (600 MHz, $CDCl_3$) Spectrum of **S21**

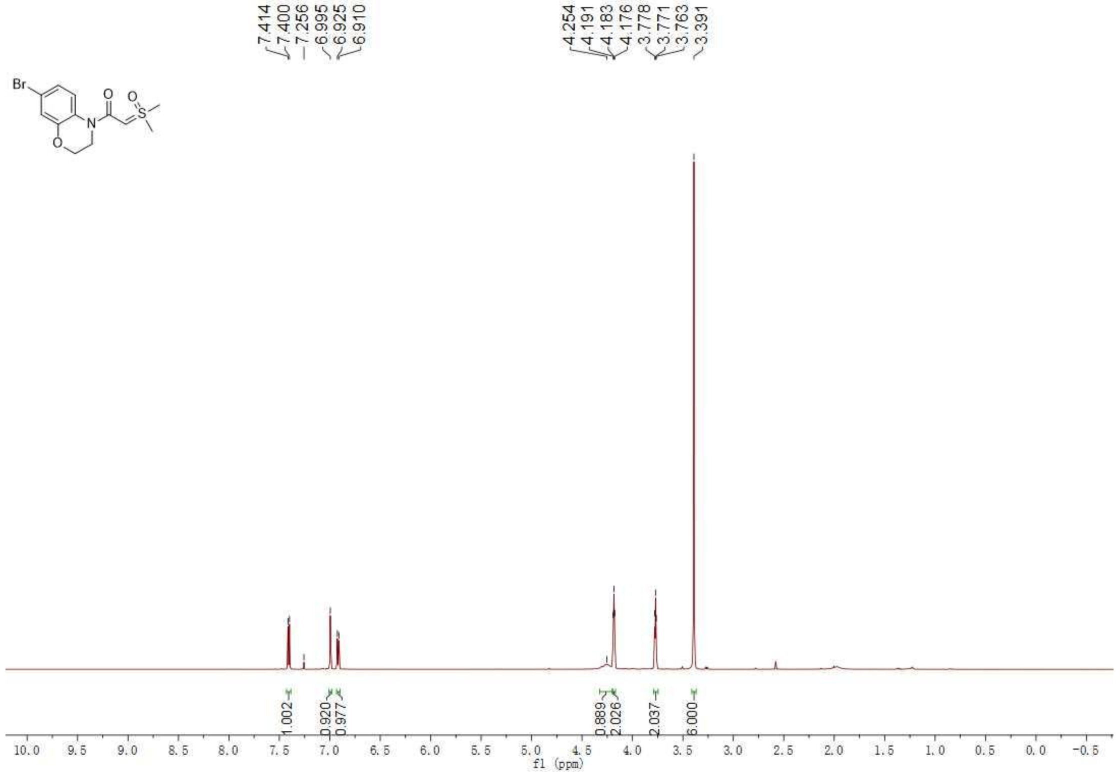

^{13}C NMR (150 MHz, $CDCl_3$) Spectrum of **S21**

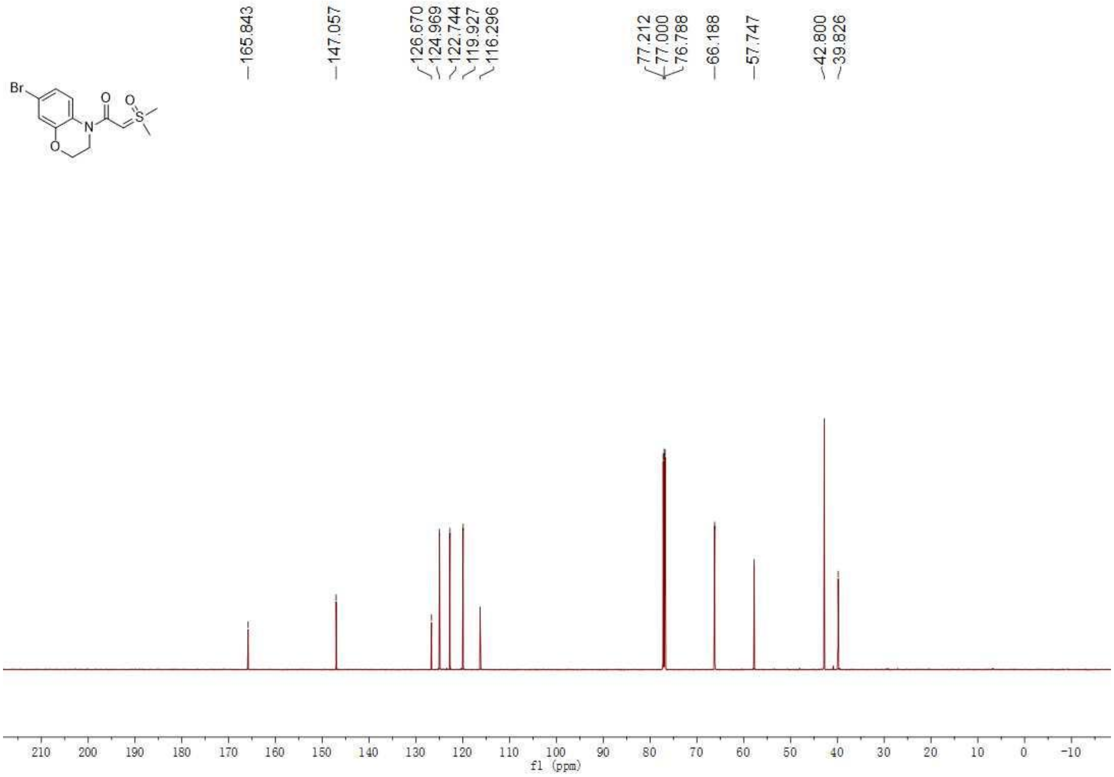

^1H NMR (600 MHz, $CDCl_3$) Spectrum of **S22**

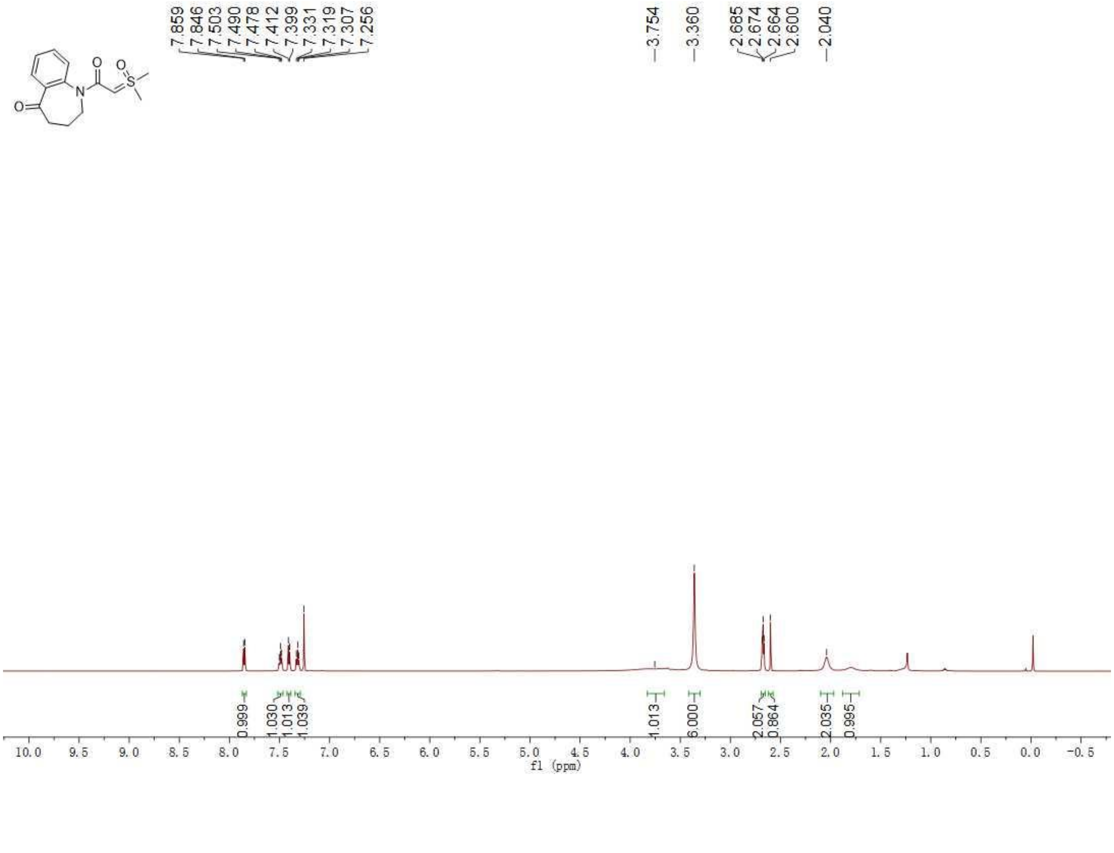

^{13}C NMR (150 MHz, $CDCl_3$) Spectrum of **S22**

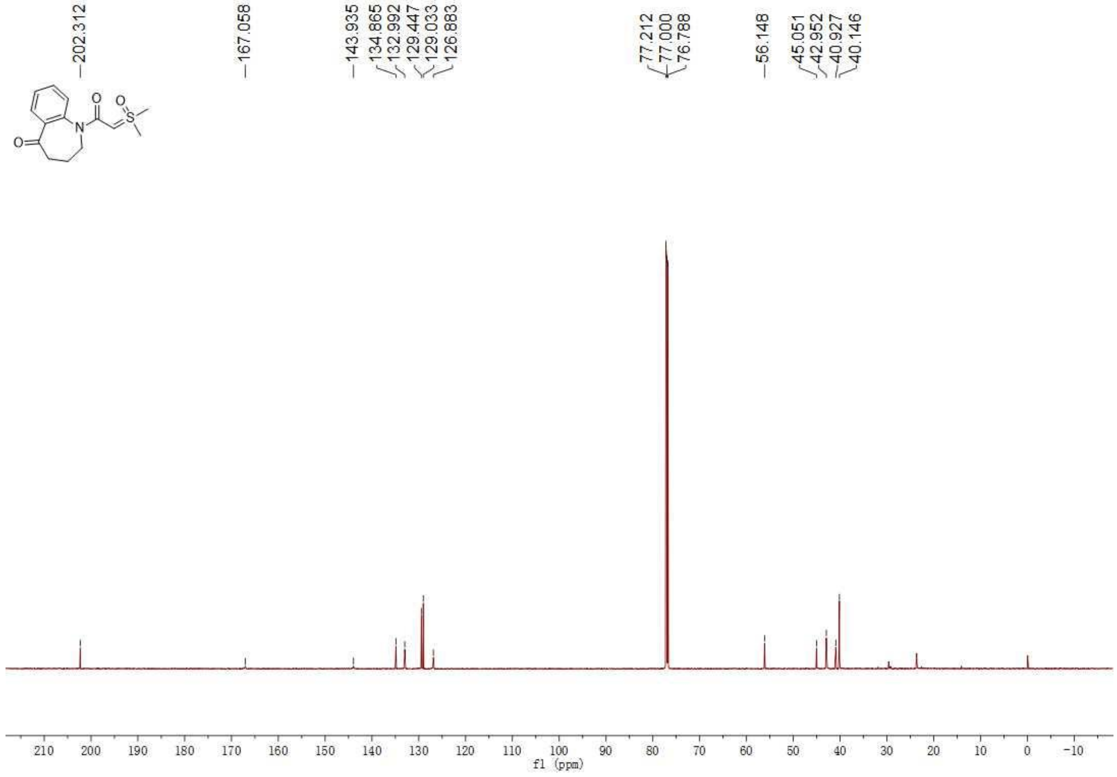

^{1}H NMR (600 MHz, $CDCl_3$) Spectrum of **S23**

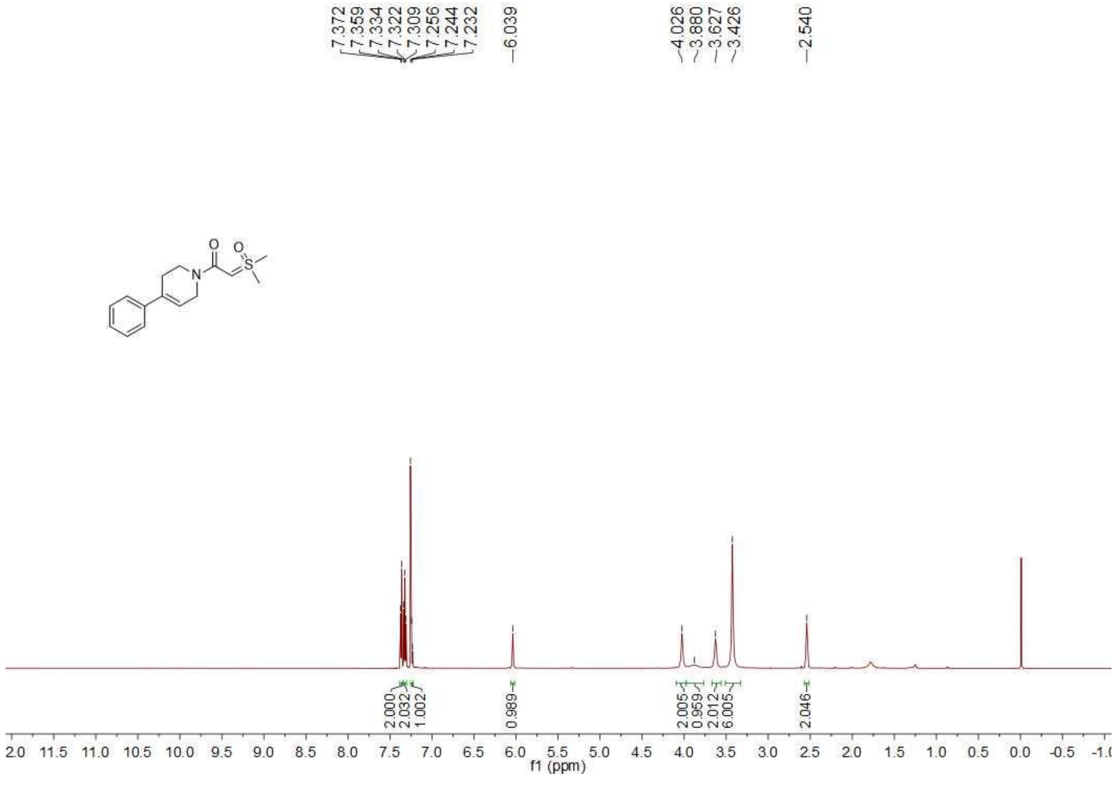

^{13}C NMR (150 MHz, $CDCl_3$) Spectrum of **S23**

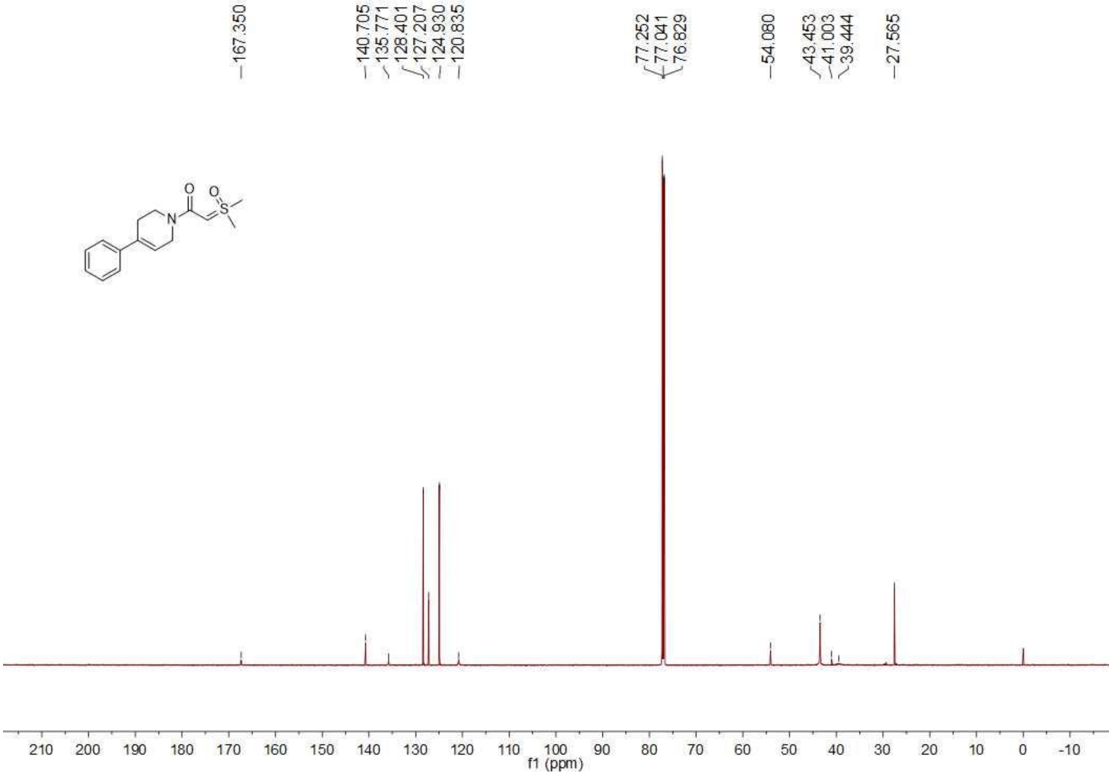

^{1}H NMR (600 MHz, $CDCl_3$) Spectrum of **S25**

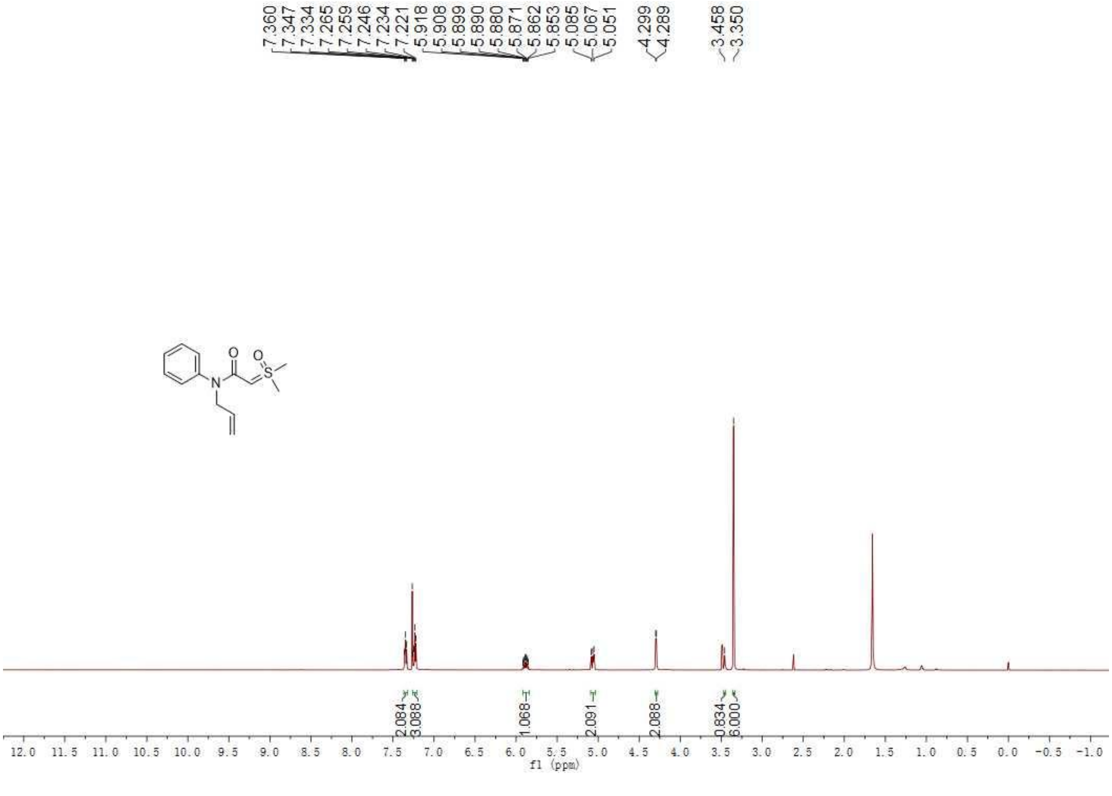

^{13}C NMR (150 MHz, $CDCl_3$) Spectrum of **S25**

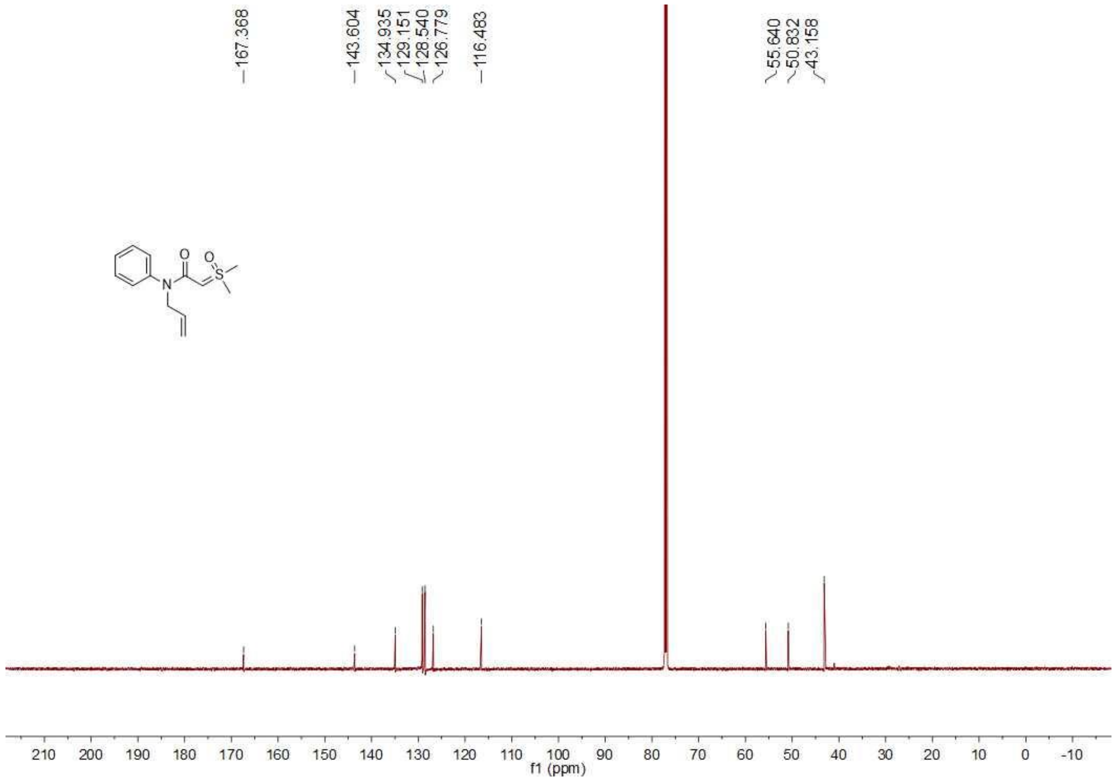

^{1}H NMR (600 MHz, $CDCl_3$) Spectrum of **S26**

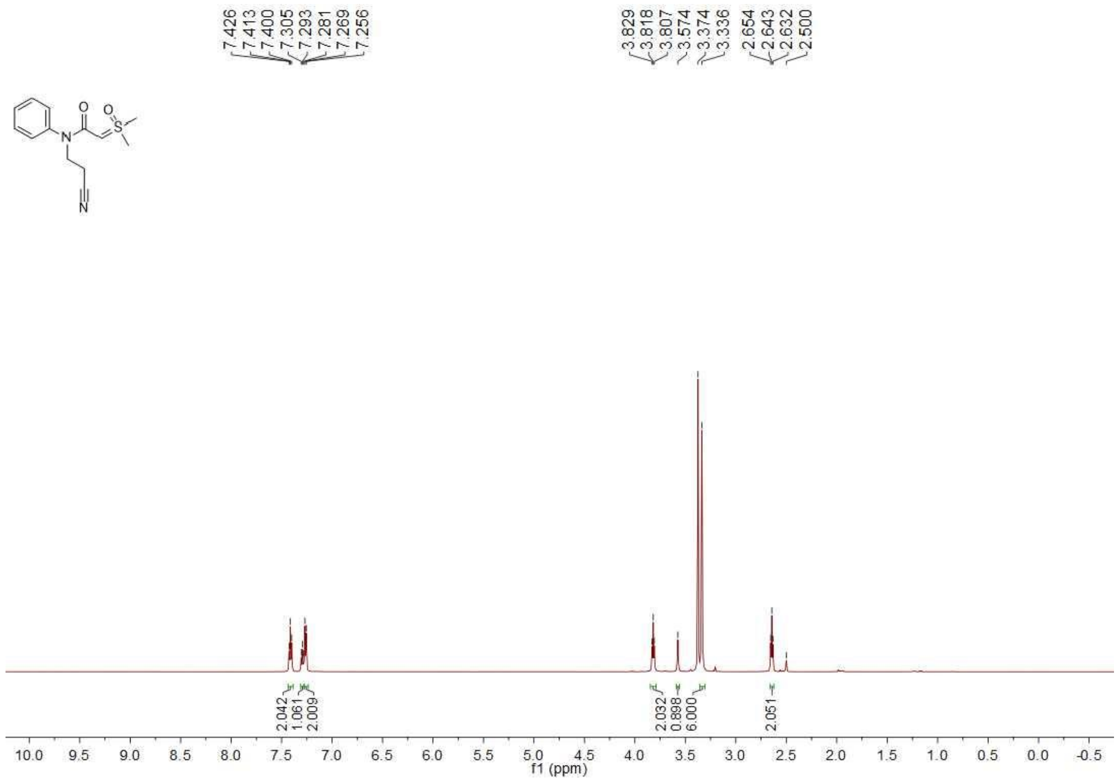

^{13}C NMR (150 MHz, $CDCl_3$) Spectrum of **S26**

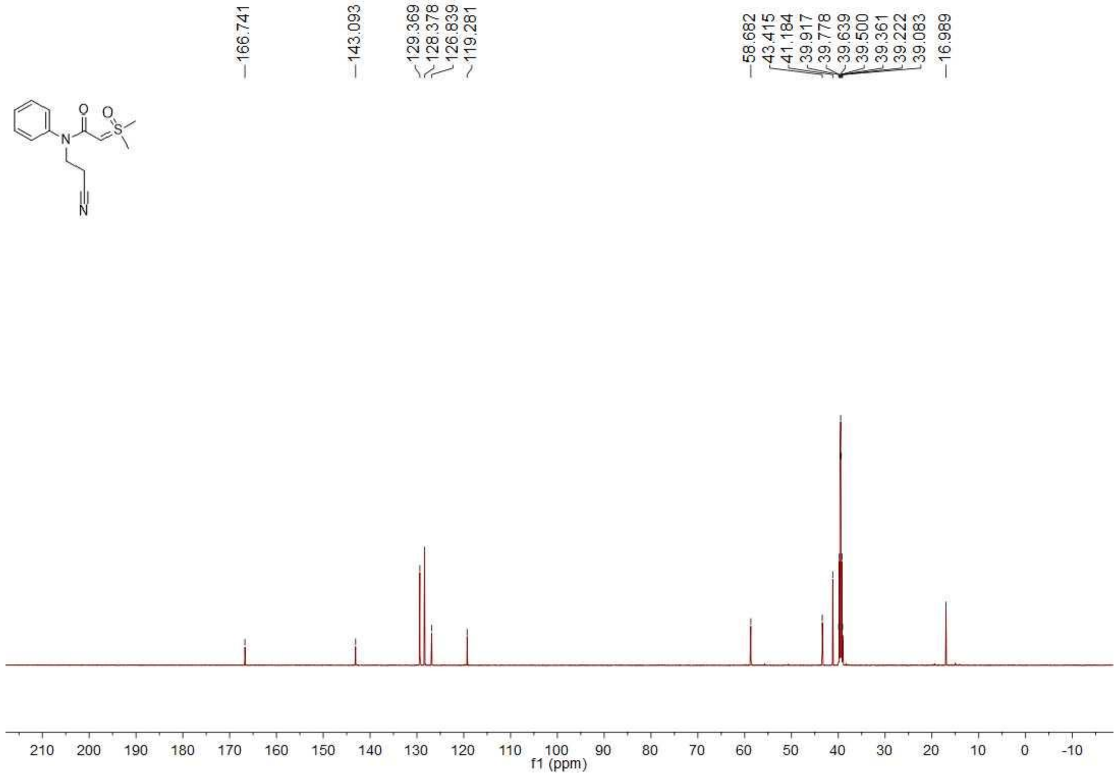

^{1}H NMR (600 MHz, $CDCl_3$) Spectrum of **S27**

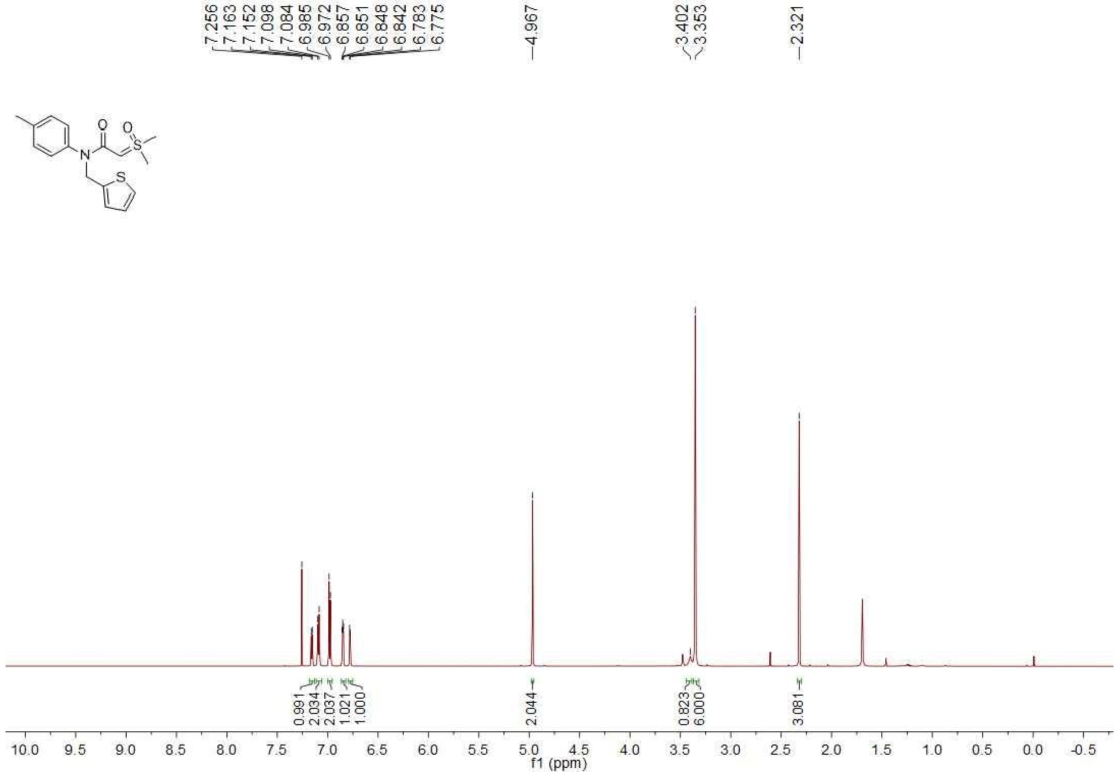

^{13}C NMR (150 MHz, $CDCl_3$) Spectrum of **S27**

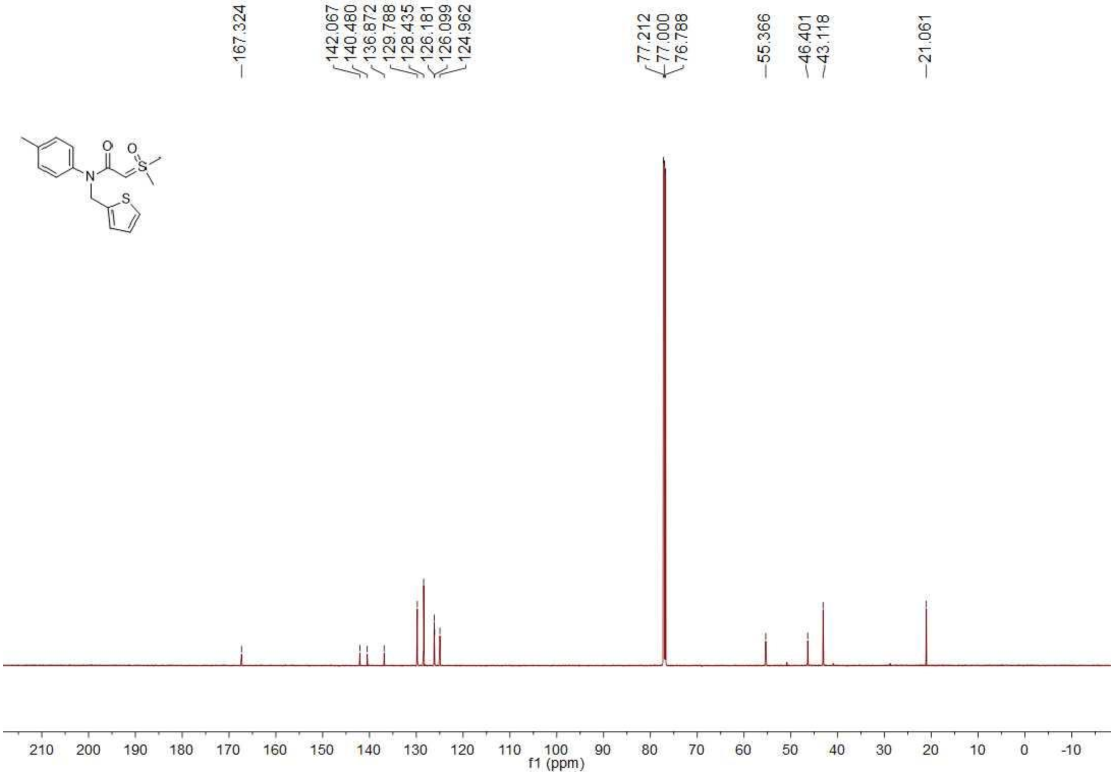

^{1}H NMR (600 MHz, $CDCl_3$) Spectrum of **S29**

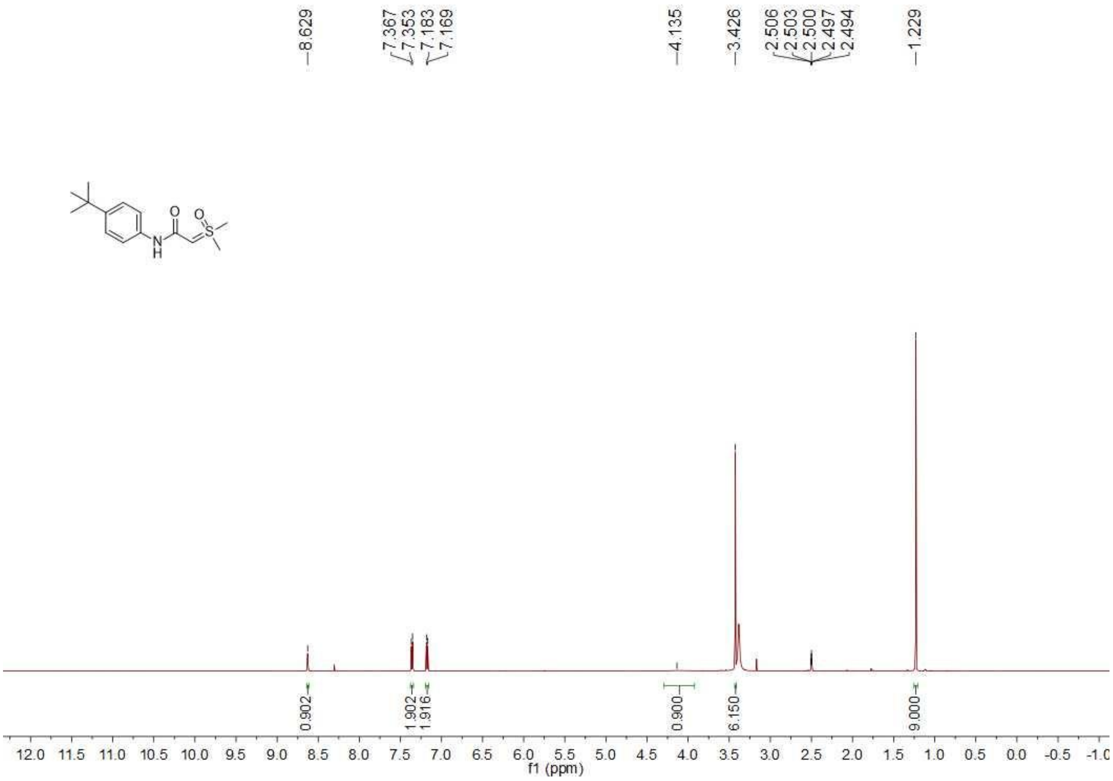

^{13}C NMR (150 MHz, $CDCl_3$) Spectrum of **S29**

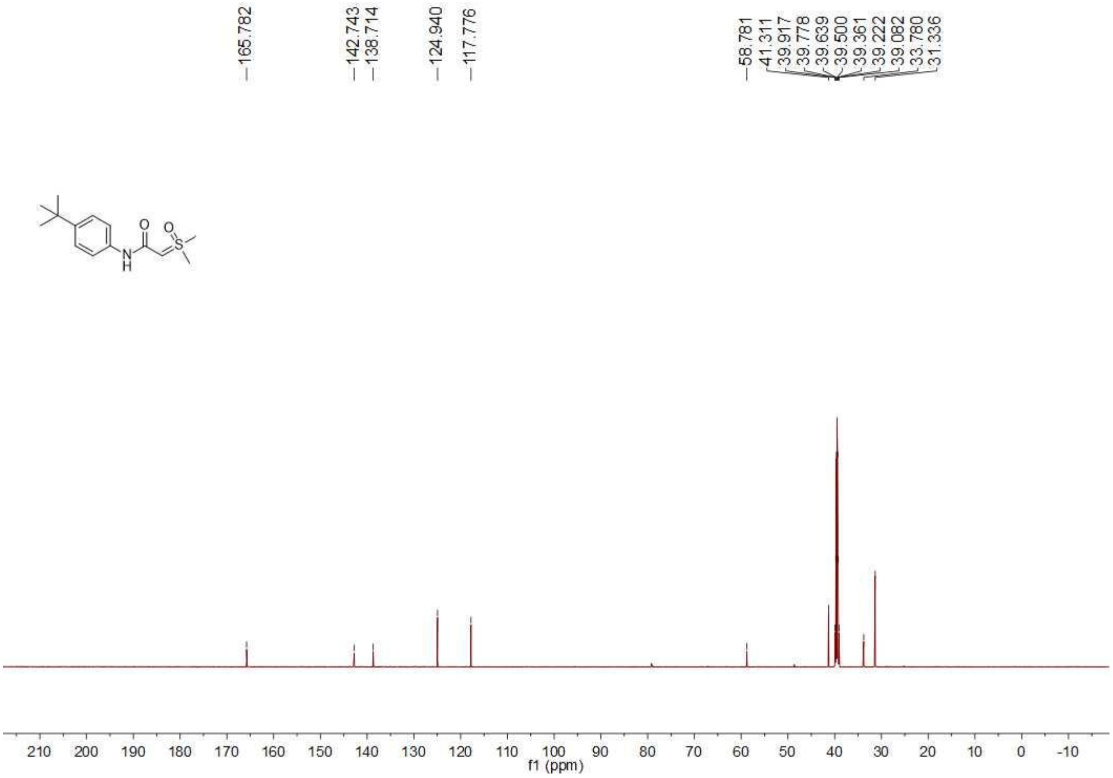

^{1}H NMR (600 MHz, $CDCl_3$) Spectrum of **S30**

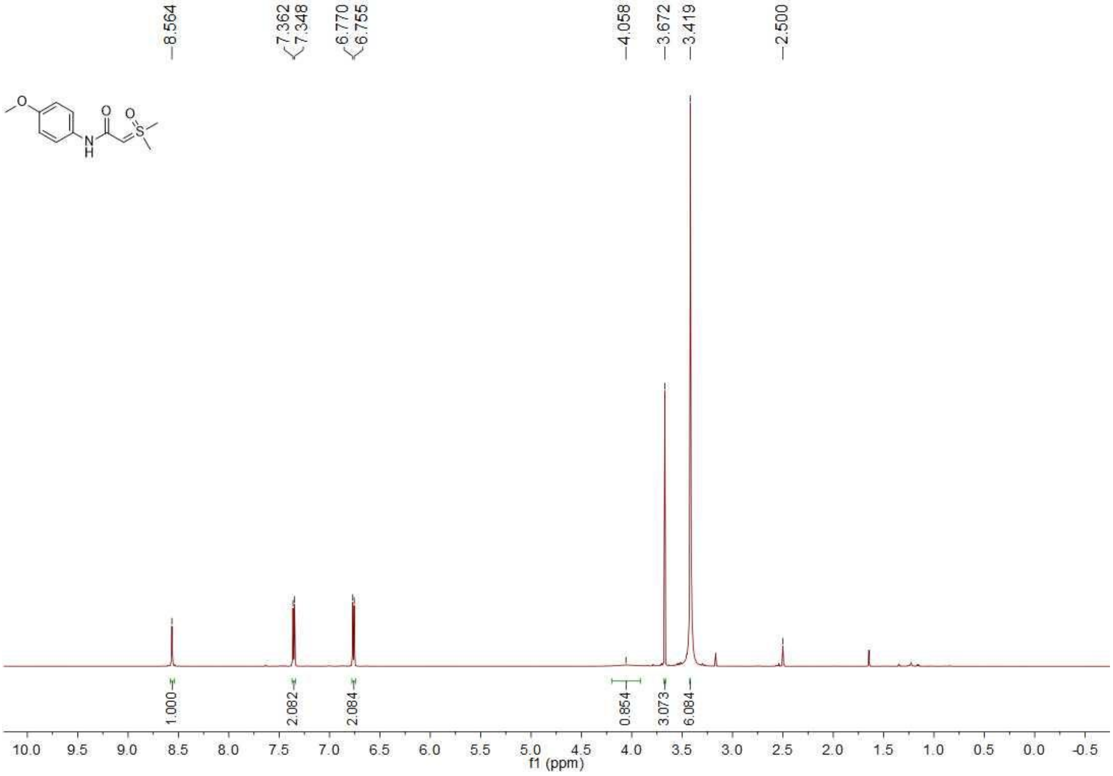

^{13}C NMR (150 MHz, $CDCl_3$) Spectrum of **S30**

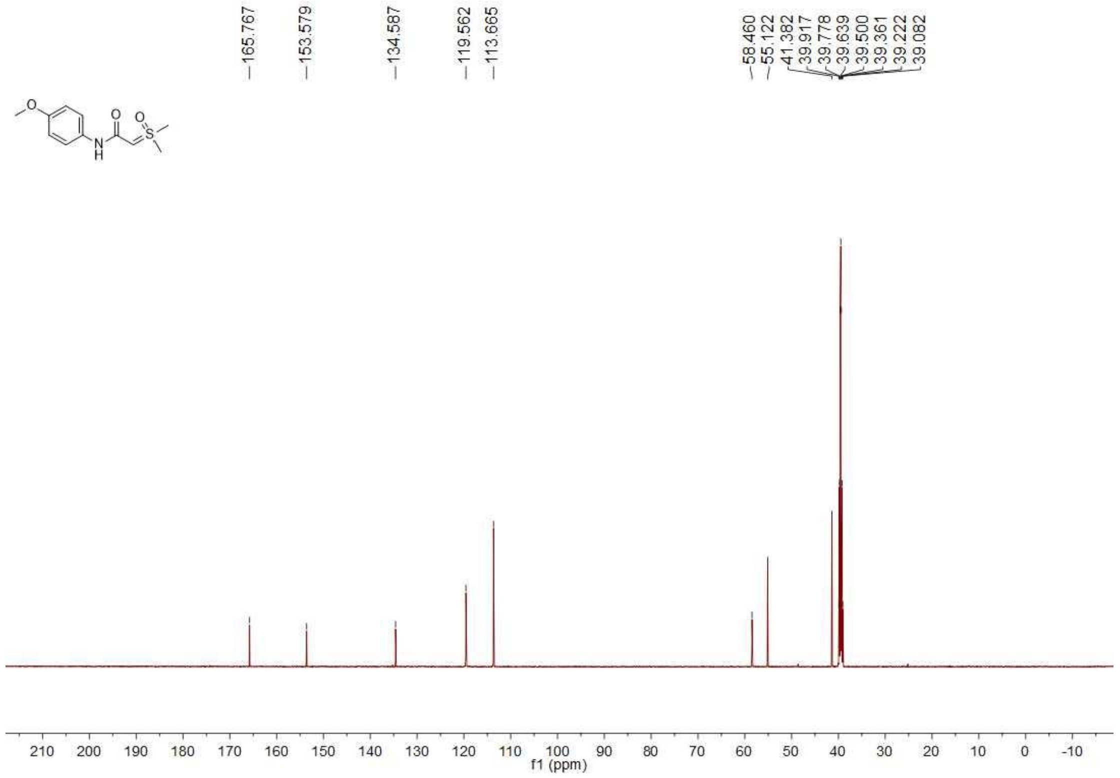

^{1}H NMR (600 MHz, $CDCl_3$) Spectrum of **S31**

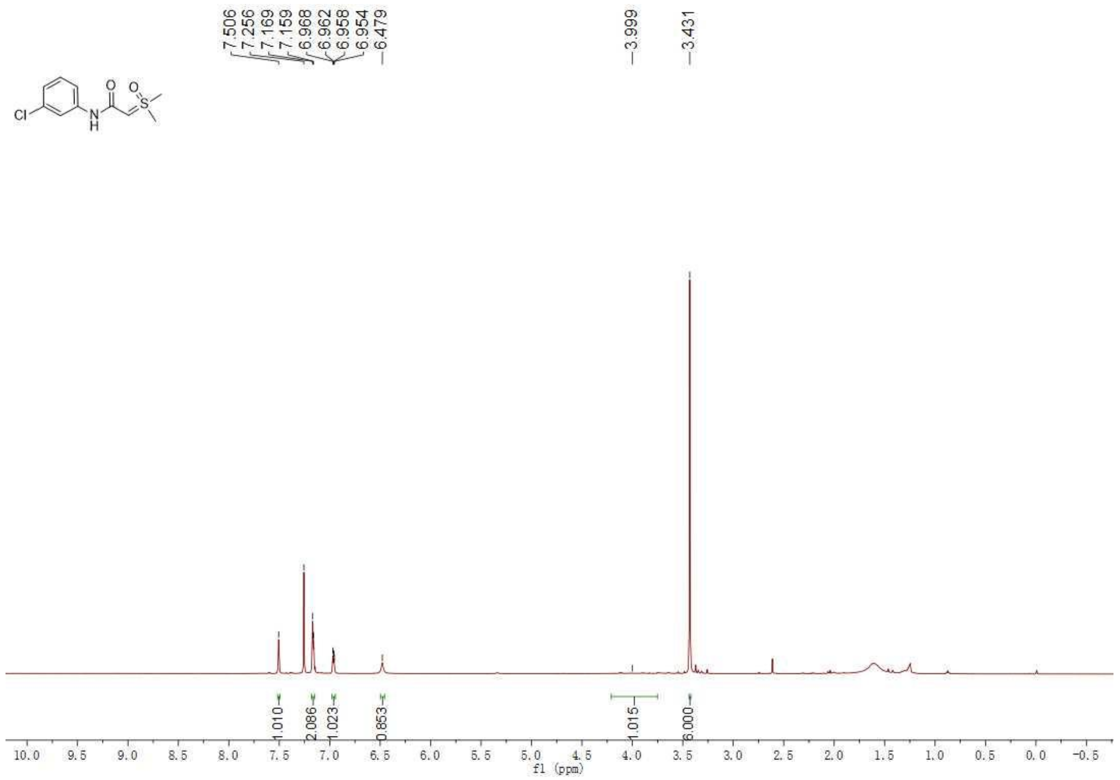

^{13}C NMR (150 MHz, $CDCl_3$) Spectrum of **S31**

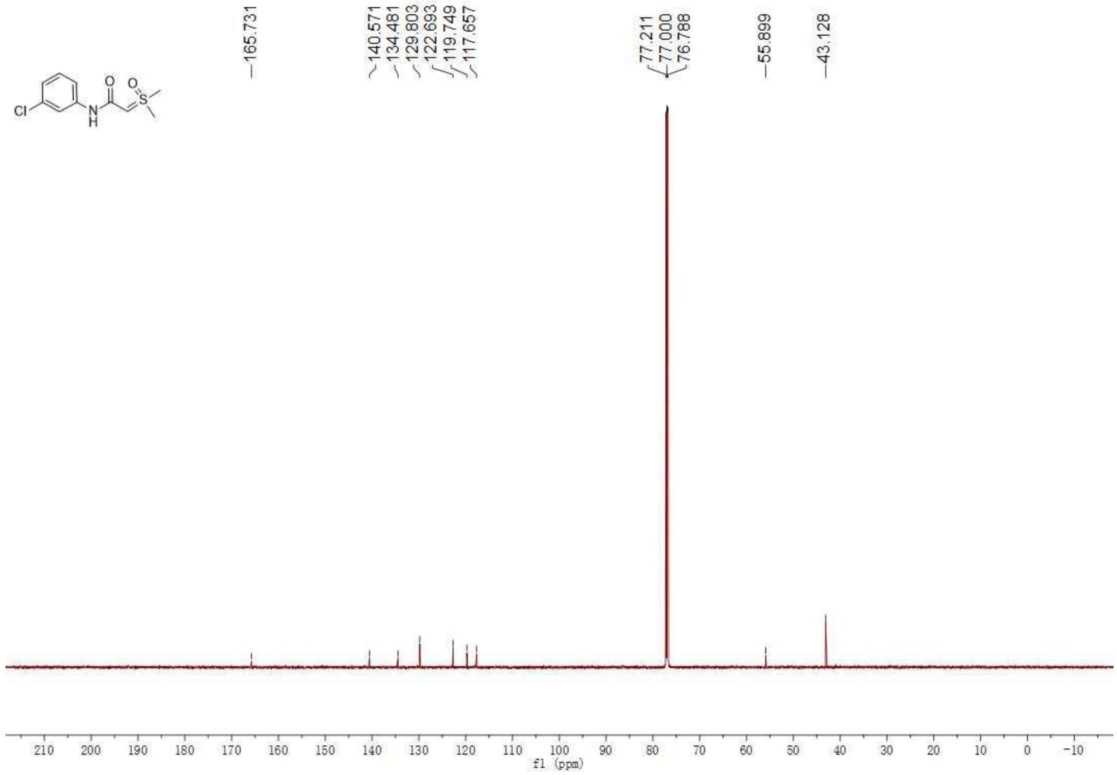

^{1}H NMR (600 MHz, $CDCl_3$) Spectrum of **S32**

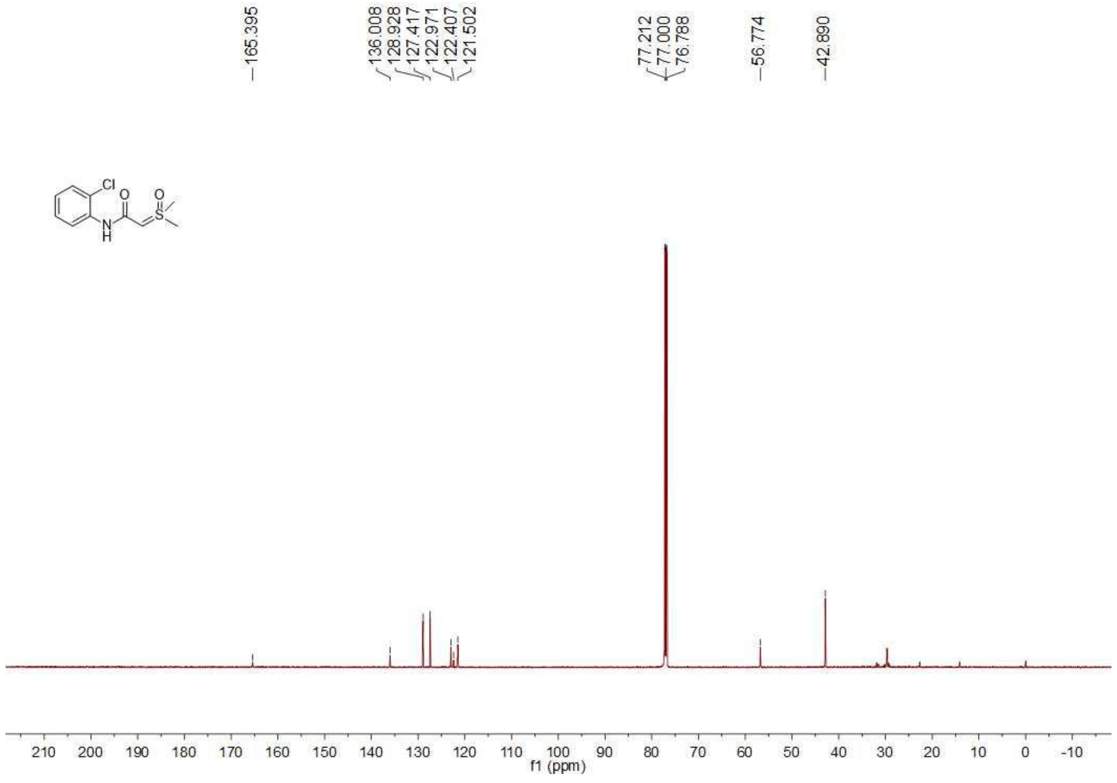

^{13}C NMR (150 MHz, $CDCl_3$) Spectrum of **S32**

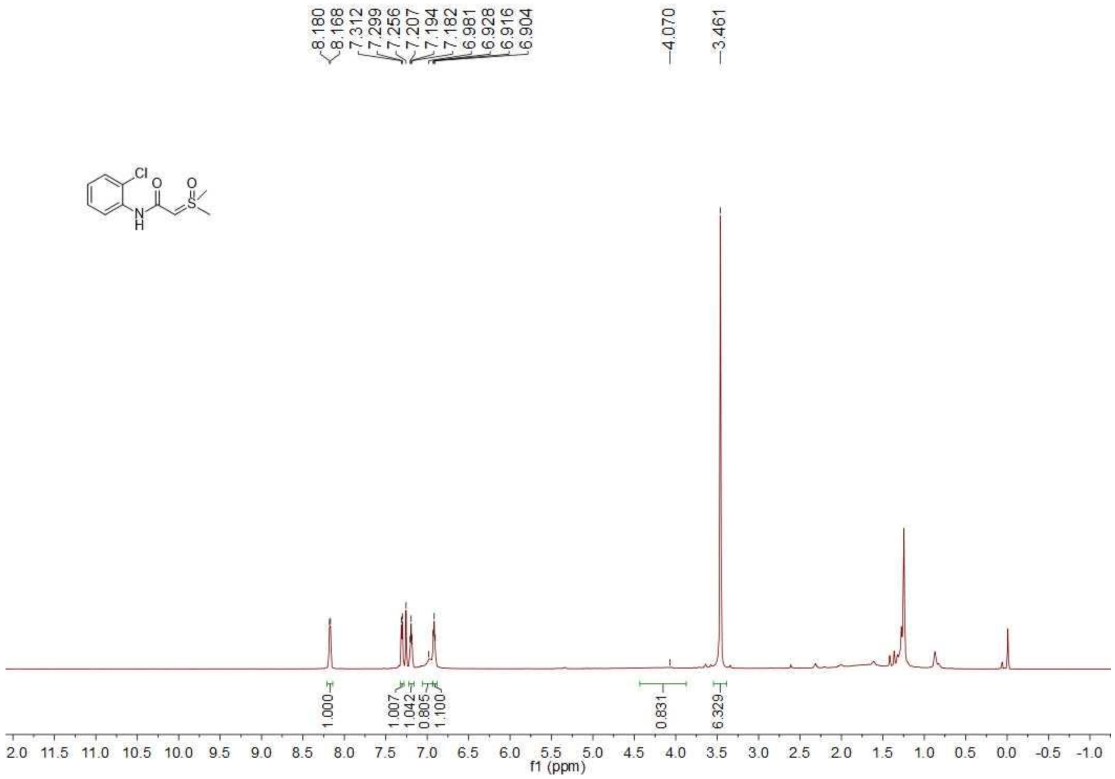

^{1}H NMR (600 MHz, $CDCl_3$) Spectrum of **S33**

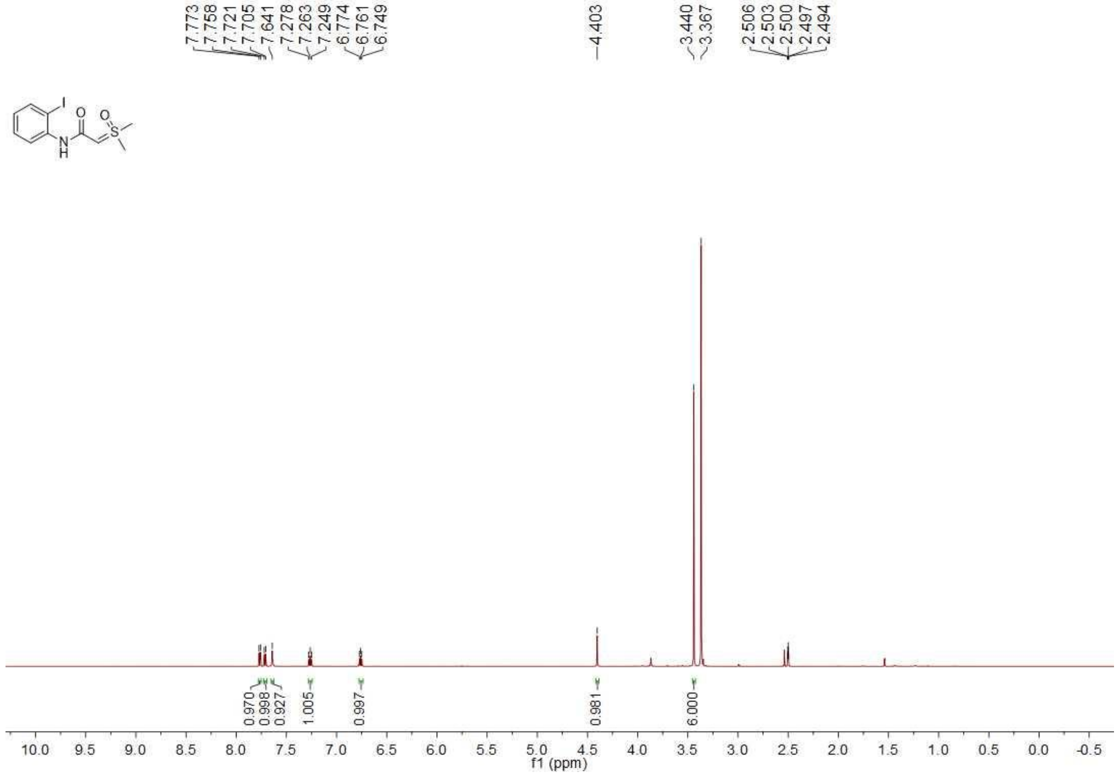

^{13}C NMR (150 MHz, $CDCl_3$) Spectrum of **S33**

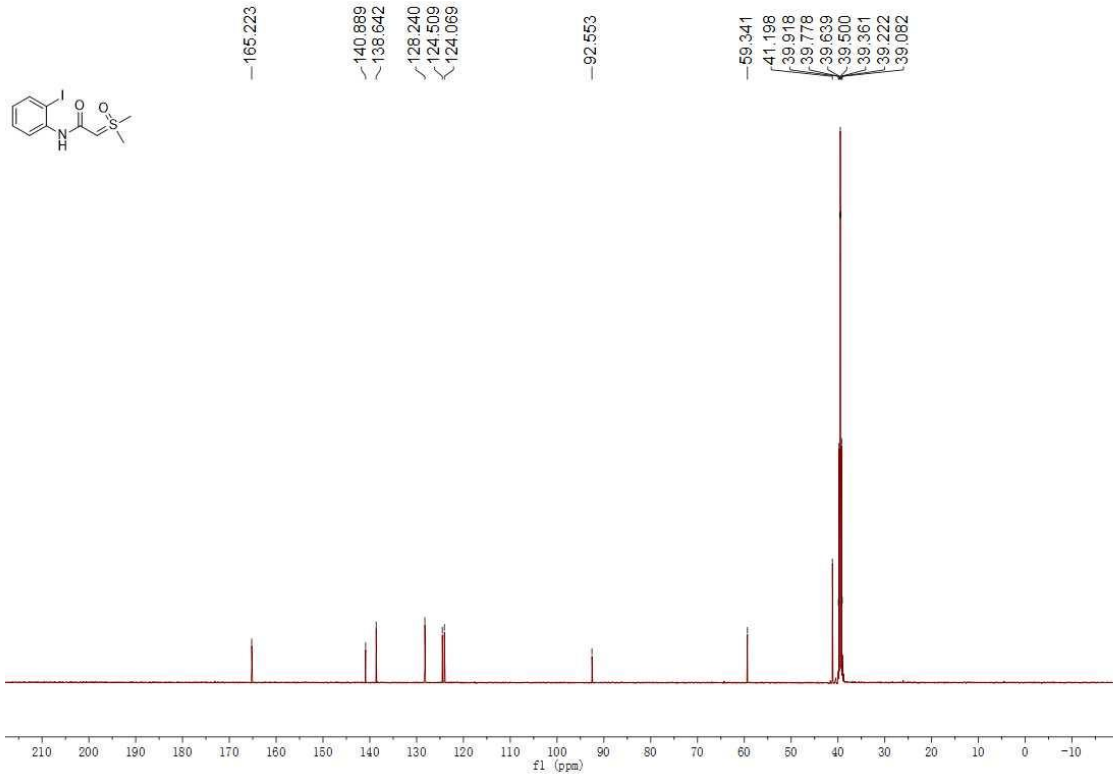

^{1}H NMR (600 MHz, DMSO-d_6) Spectrum of **S34**

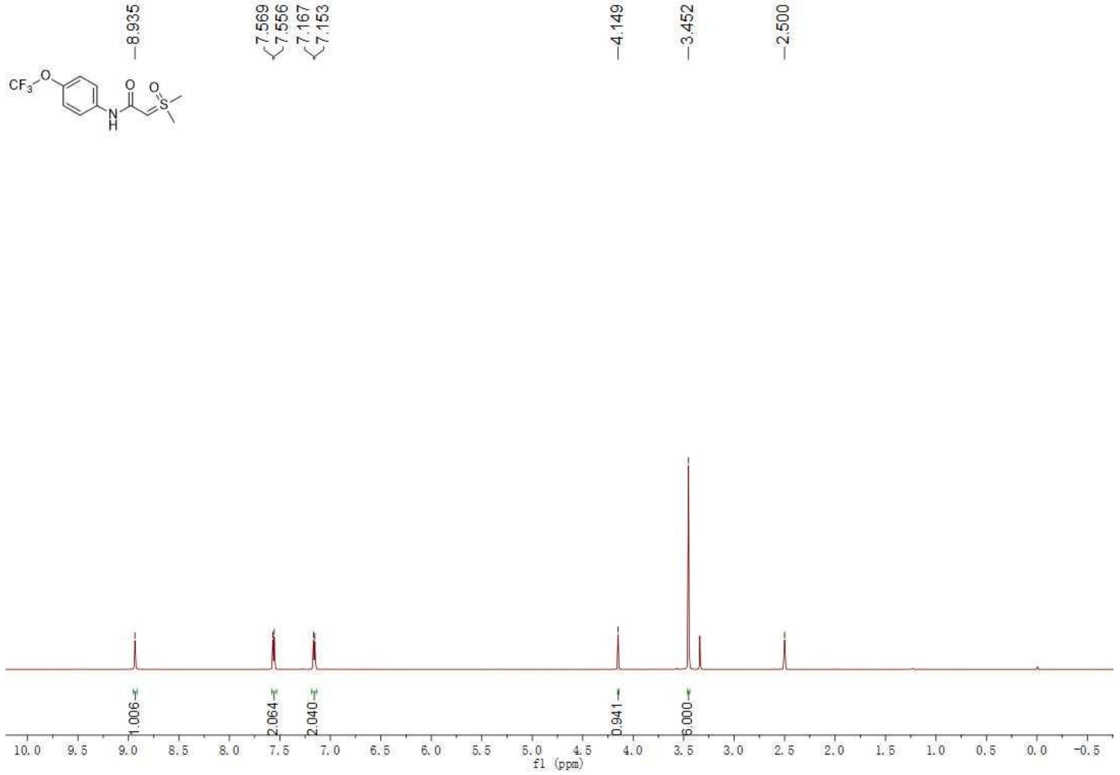

^{13}C NMR (150 MHz, DMSO-d_6) Spectrum of **S34**

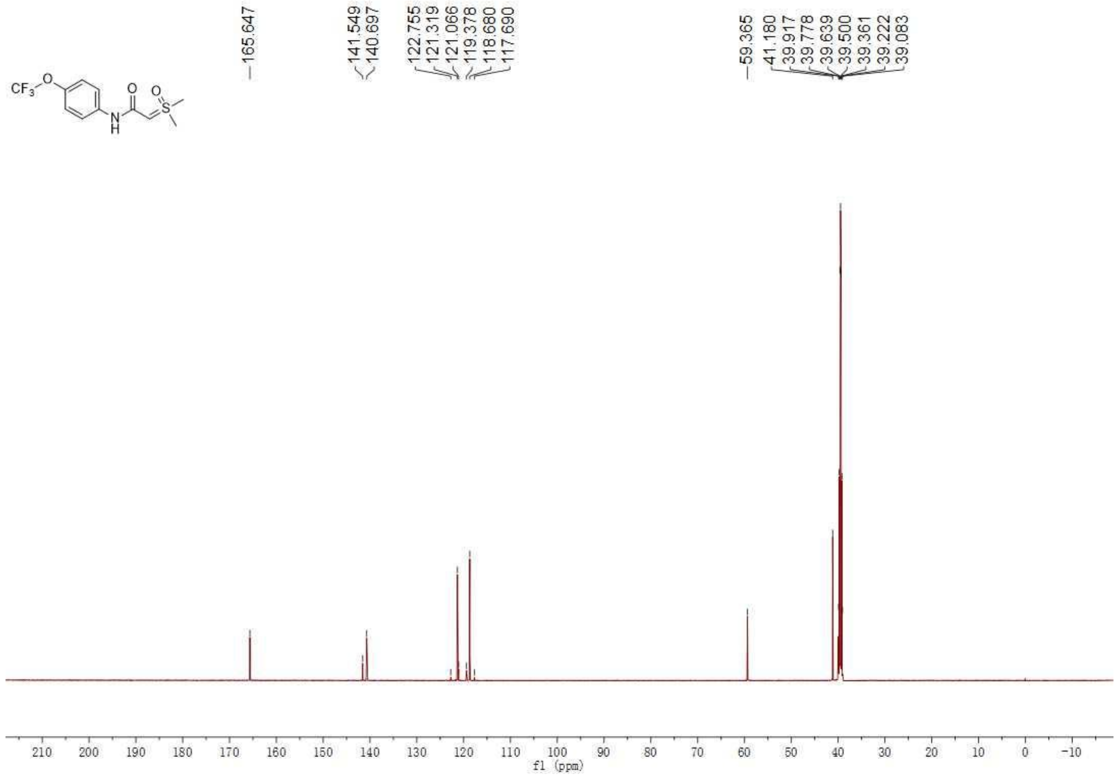

^{19}F NMR (564 MHz, DMSO-d_6) Spectrum of **S34**

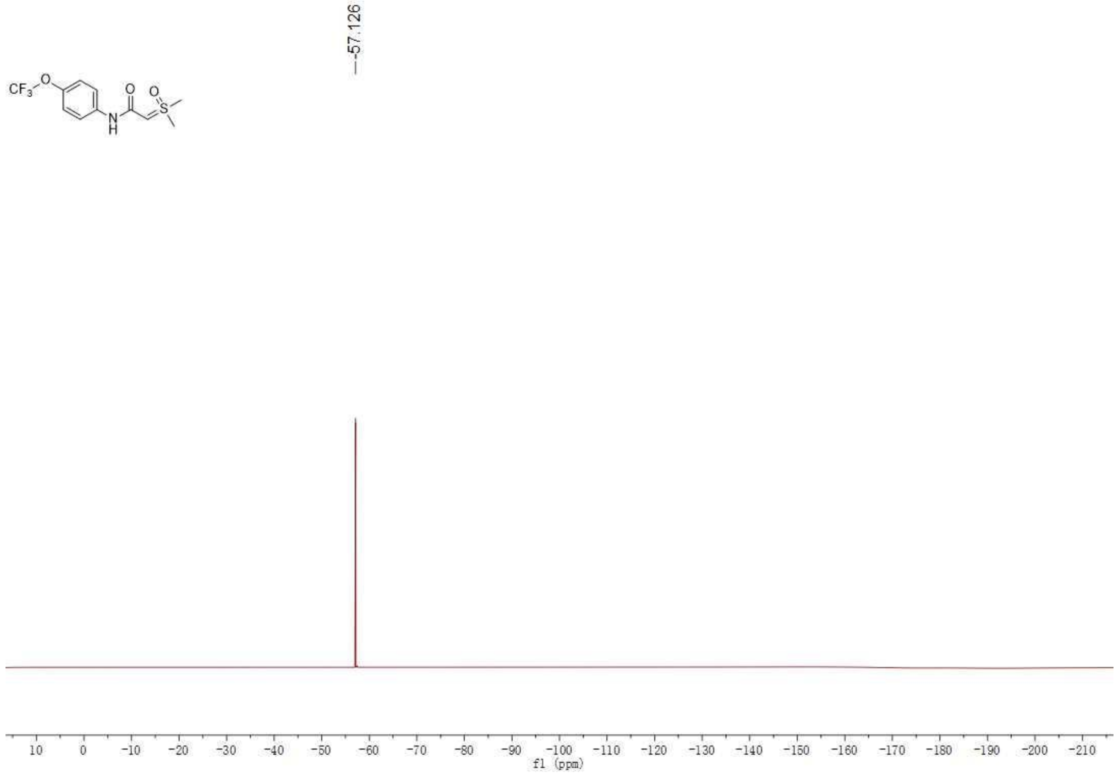

^{1}H NMR (600 MHz, DMSO-d_6) Spectrum of **S35**

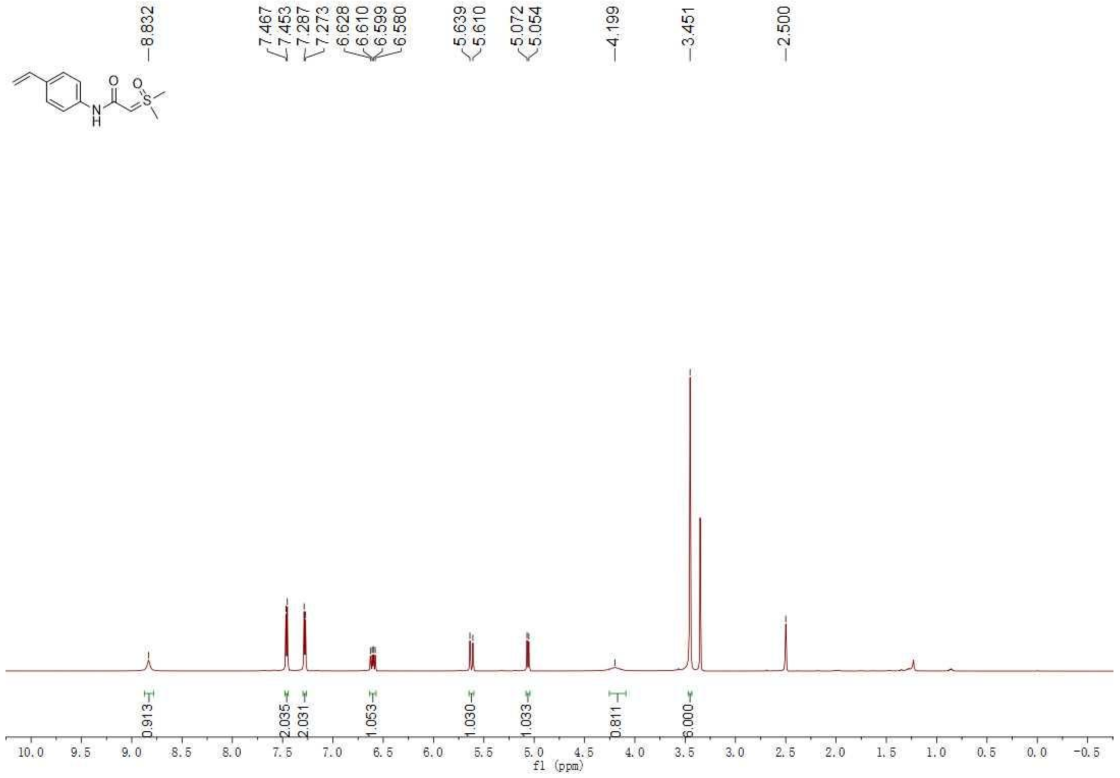

^{13}C NMR (150 MHz, DMSO-d_6) Spectrum of **S35**

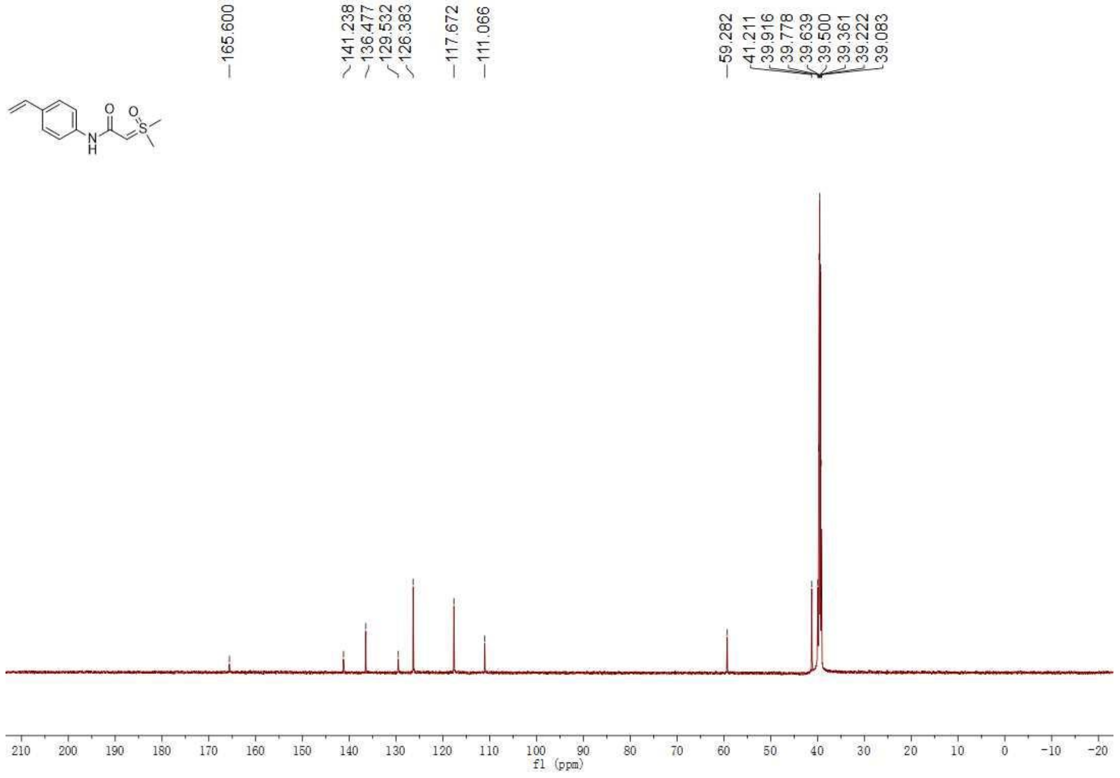

^{1}H NMR (600 MHz, $CDCl_3$) Spectrum of **S36**

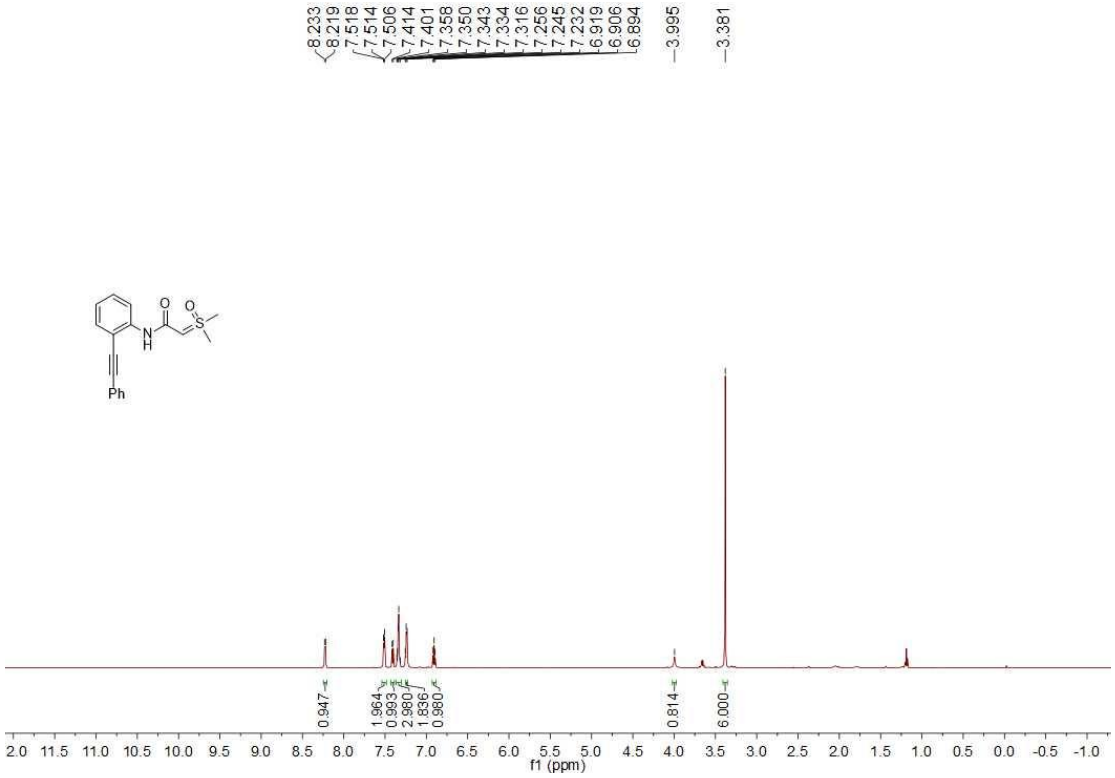

^{13}C NMR (150 MHz, $CDCl_3$) Spectrum of **S36**

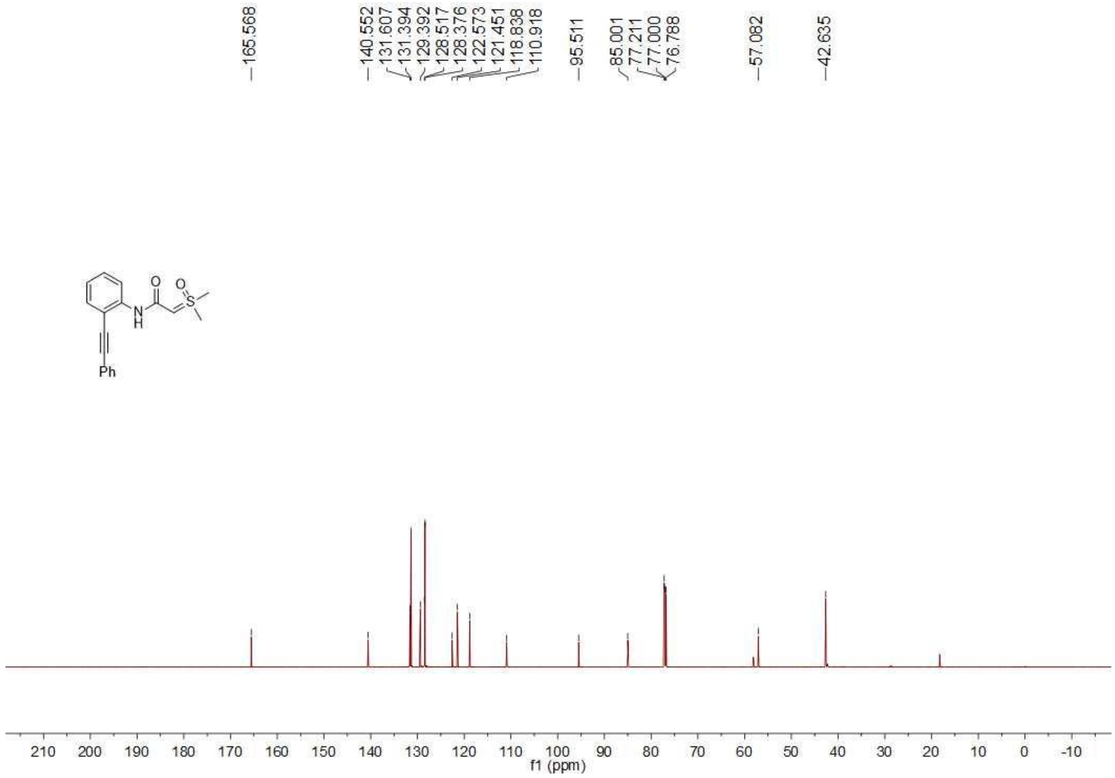

^{1}H NMR (600 MHz, DMSO-d_6) Spectrum of **S37**

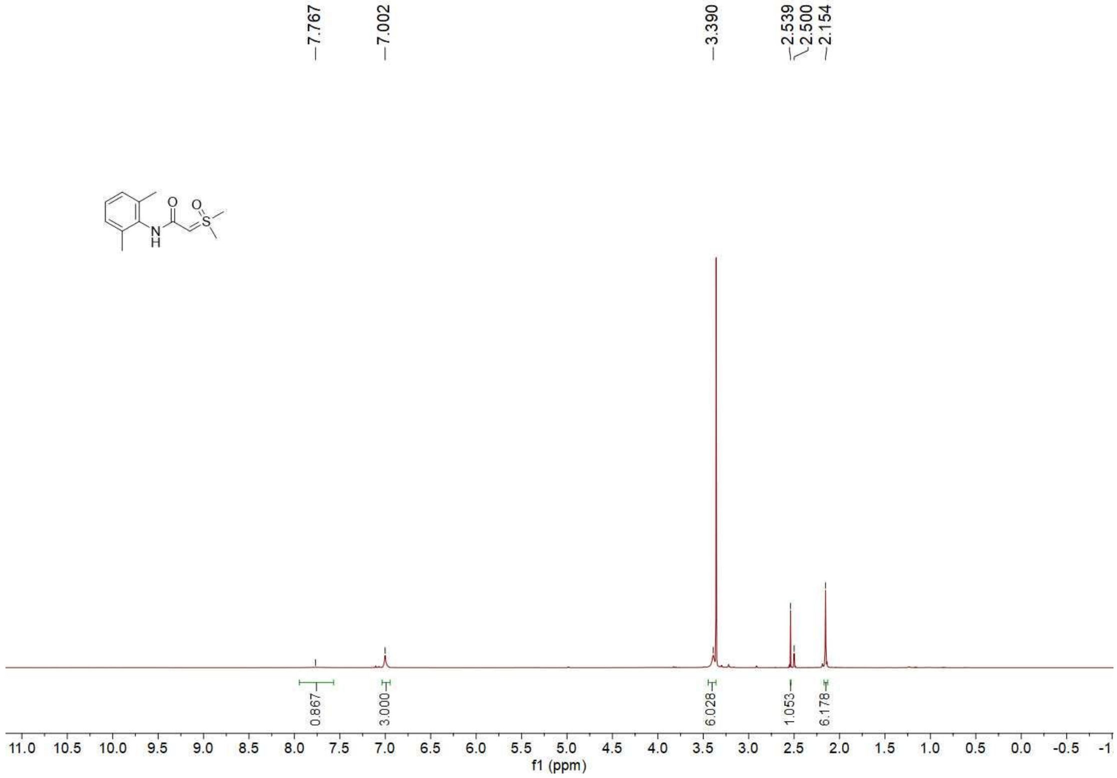

^{13}C NMR (150 MHz, DMSO-d_6) Spectrum of **S37**

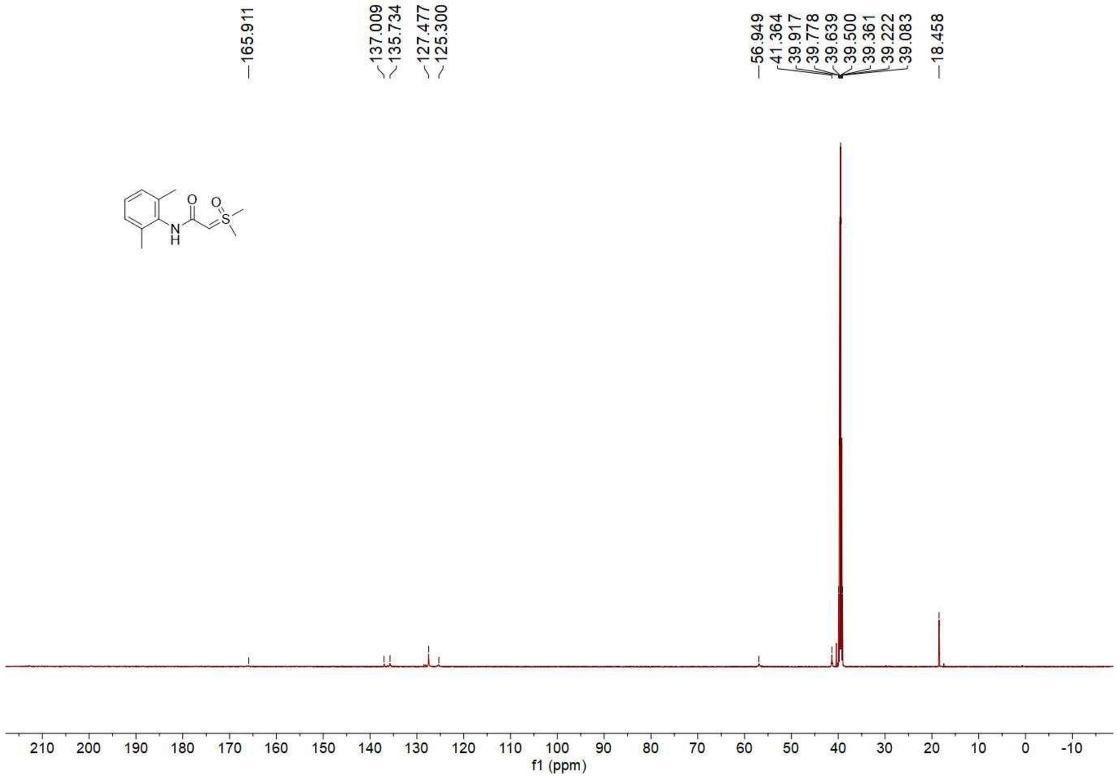

^{1}H NMR (600 MHz, $CDCl_3$) Spectrum of **S38**

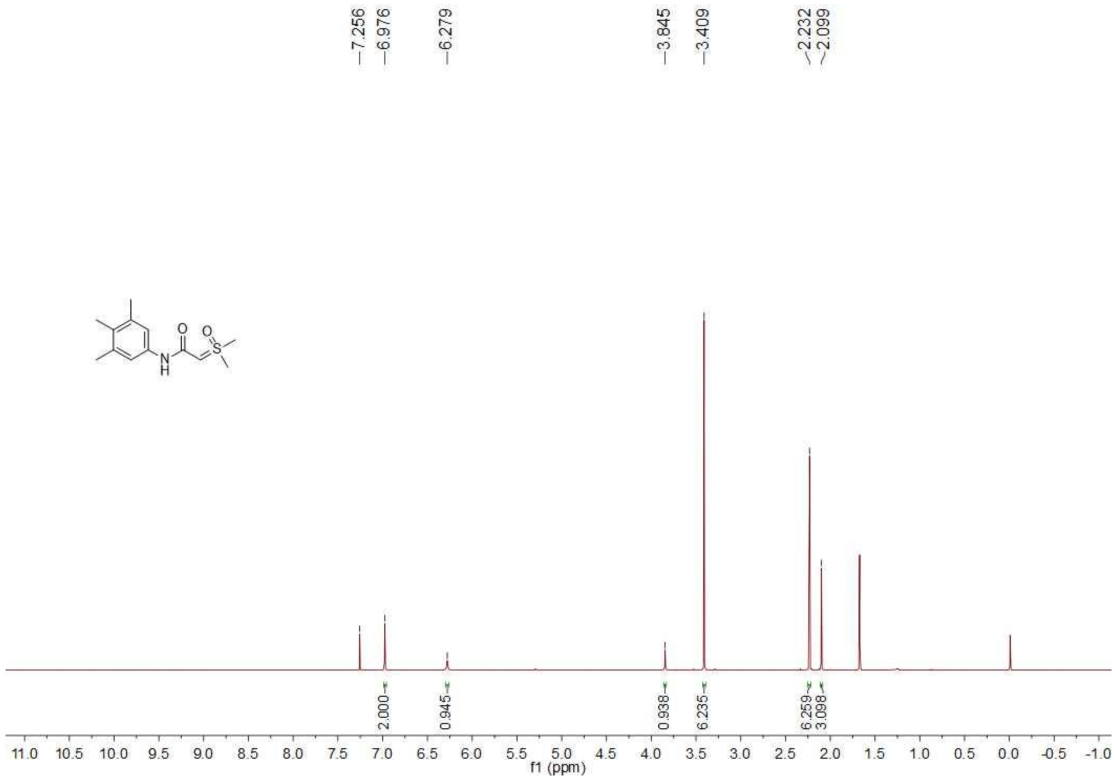

^{13}C NMR (150 MHz, $CDCl_3$) Spectrum of **S38**

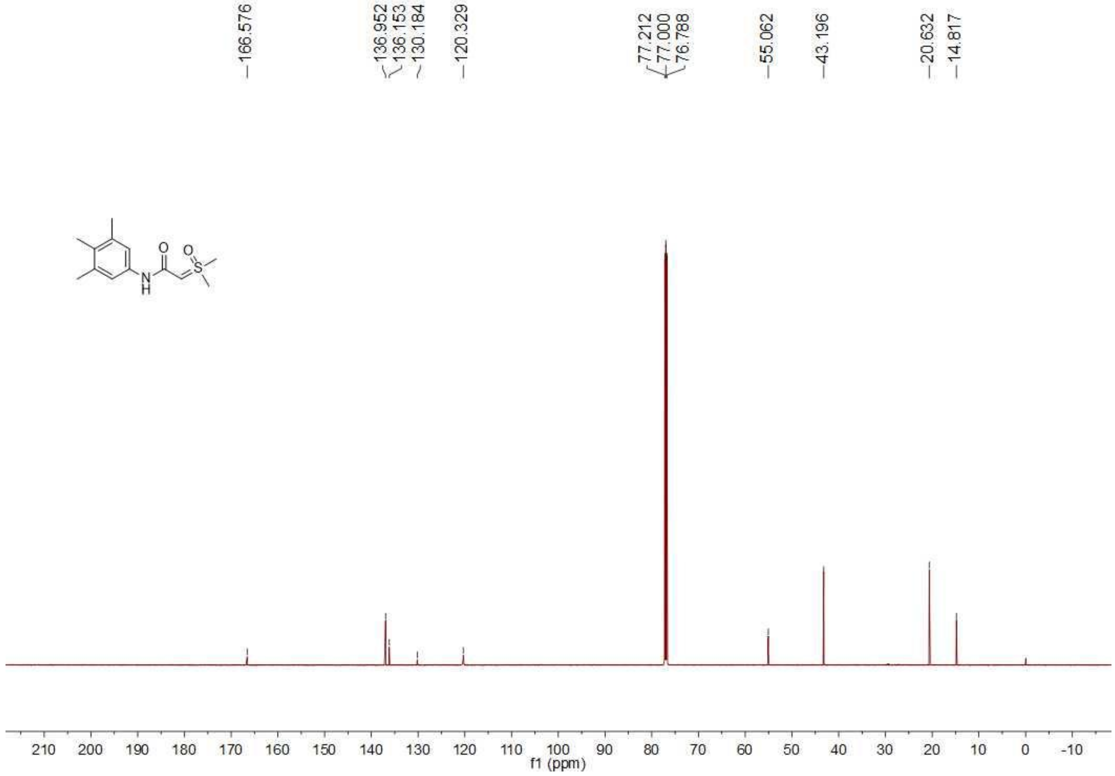

^{1}H NMR (600 MHz, $CDCl_3$) Spectrum of **S39**

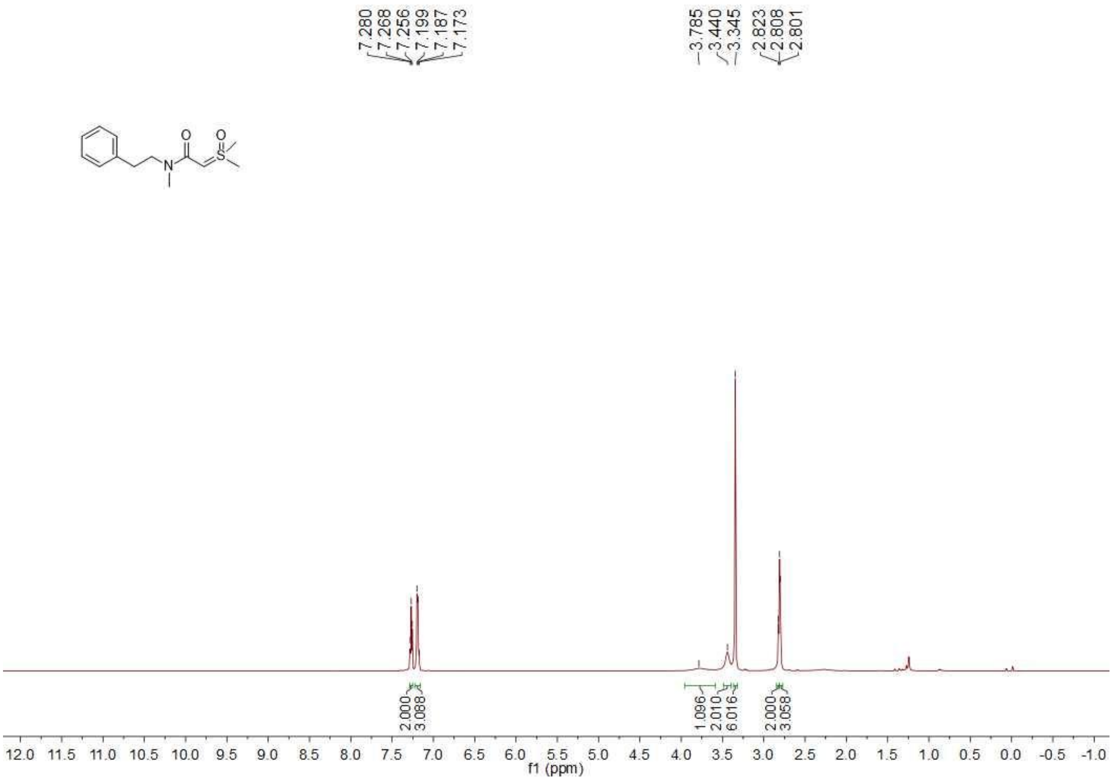

^{13}C NMR (150 MHz, $CDCl_3$) Spectrum of **S39**

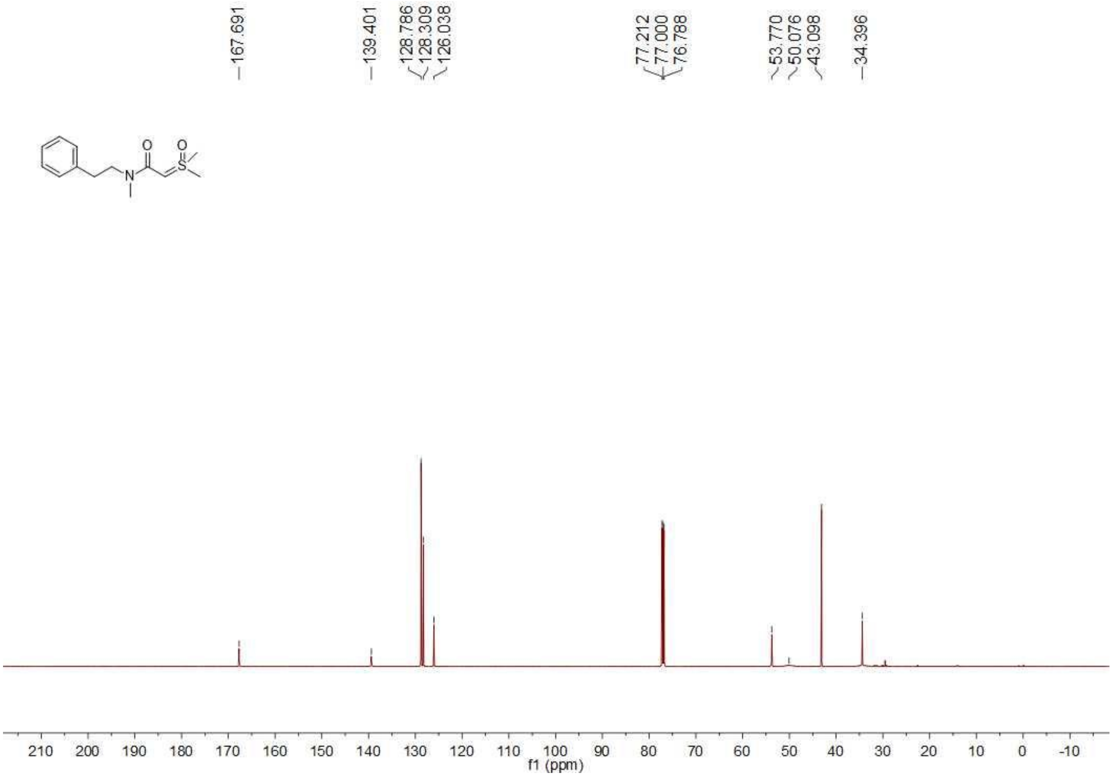

^{1}H NMR (600 MHz, DMSO-d_6) Spectrum of **S40**

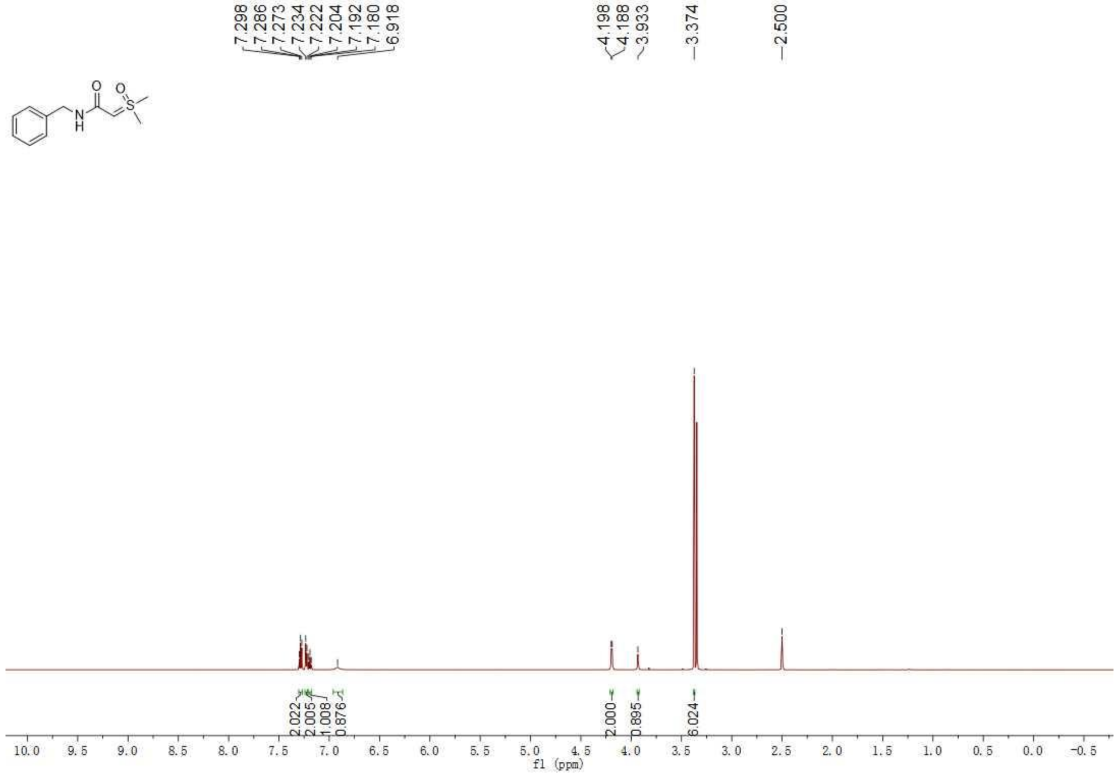

^{13}C NMR (150 MHz, DMSO-d_6) Spectrum of **S40**

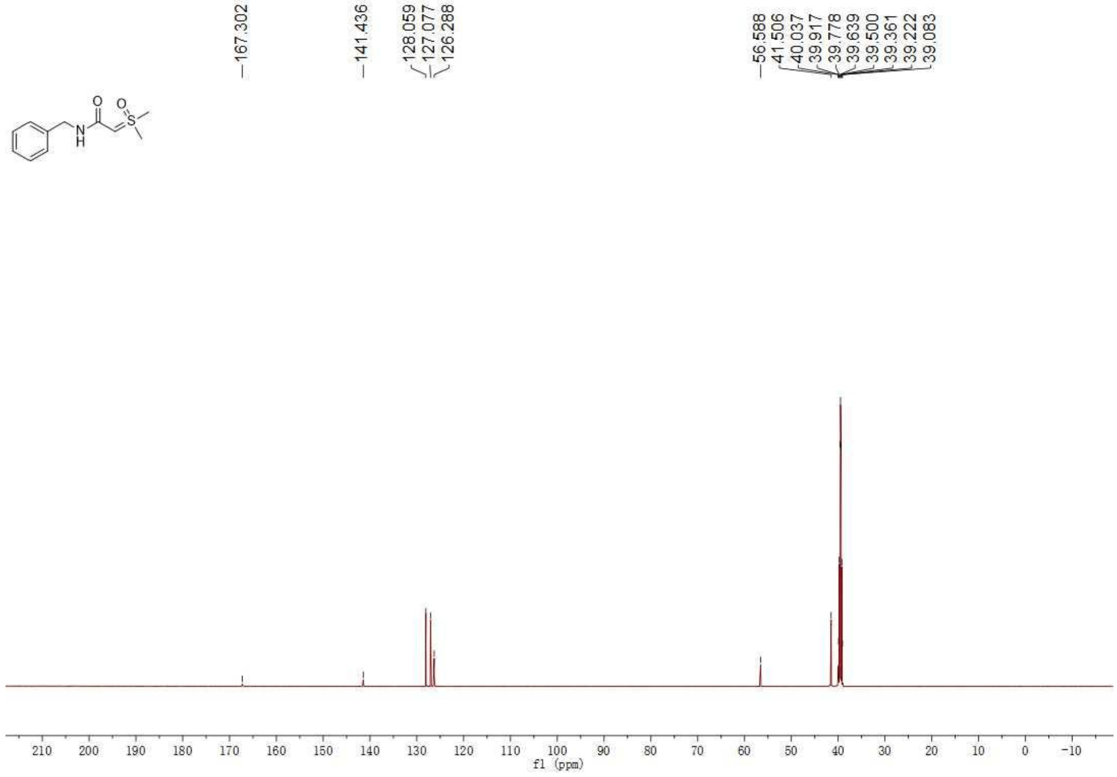

^{1}H NMR (600 MHz, DMSO-d_6) Spectrum of **S41**

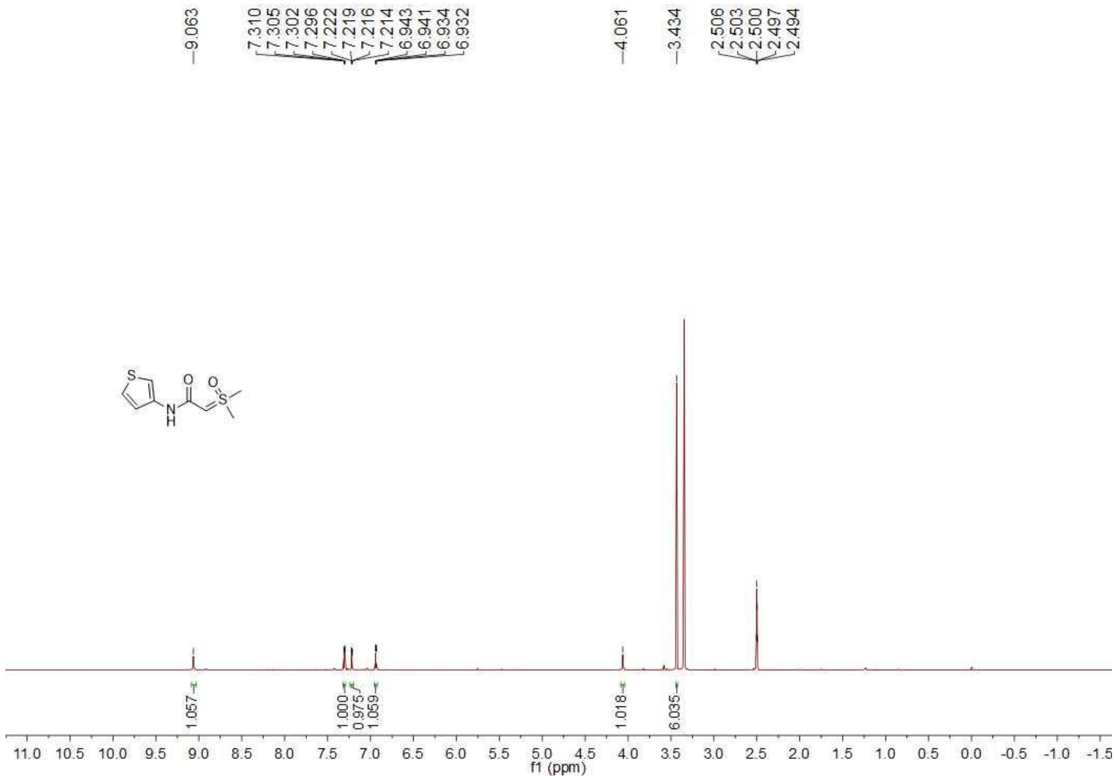

^{13}C NMR (150 MHz, DMSO-d_6) Spectrum of **S41**

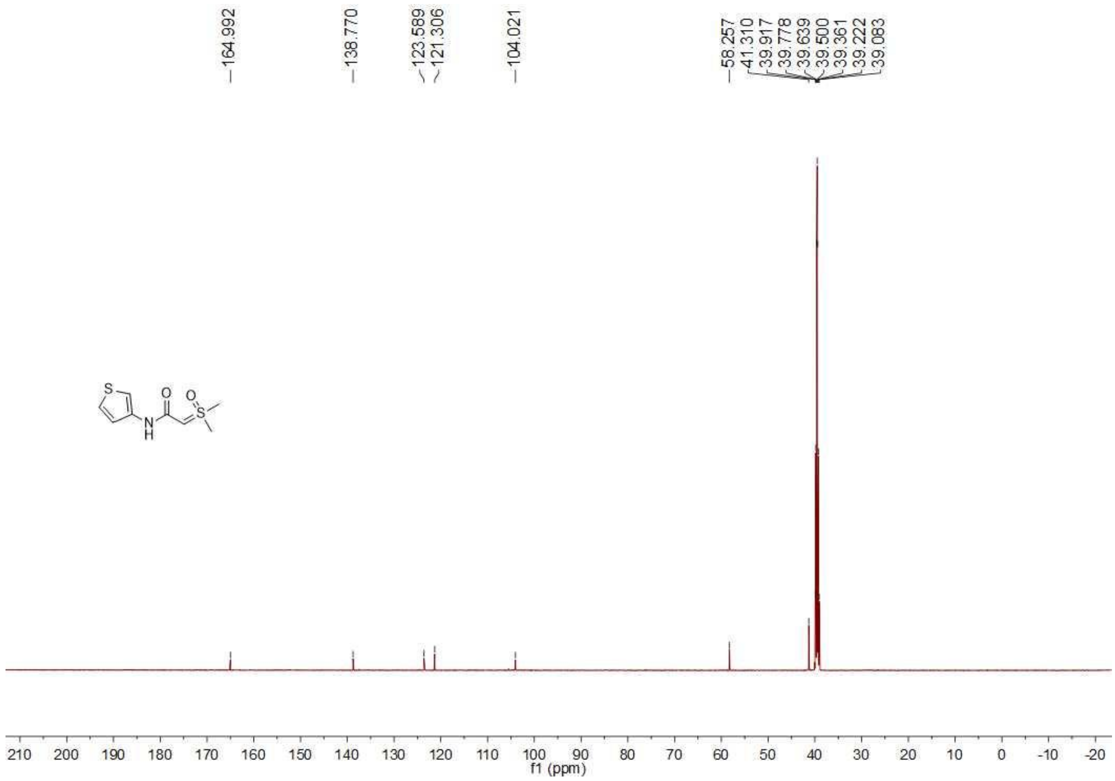

^{1}H NMR (600 MHz, $CDCl_3$) Spectrum of **S42**

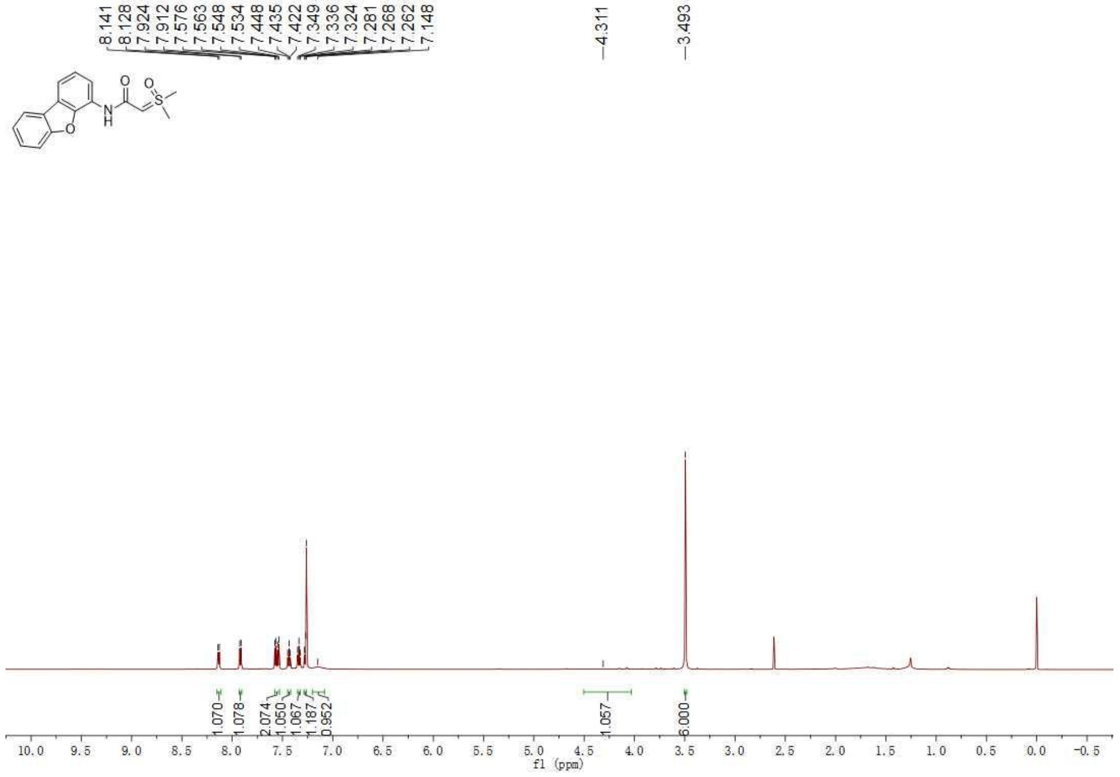

^{13}C NMR (150 MHz, $CDCl_3$) Spectrum of **S42**

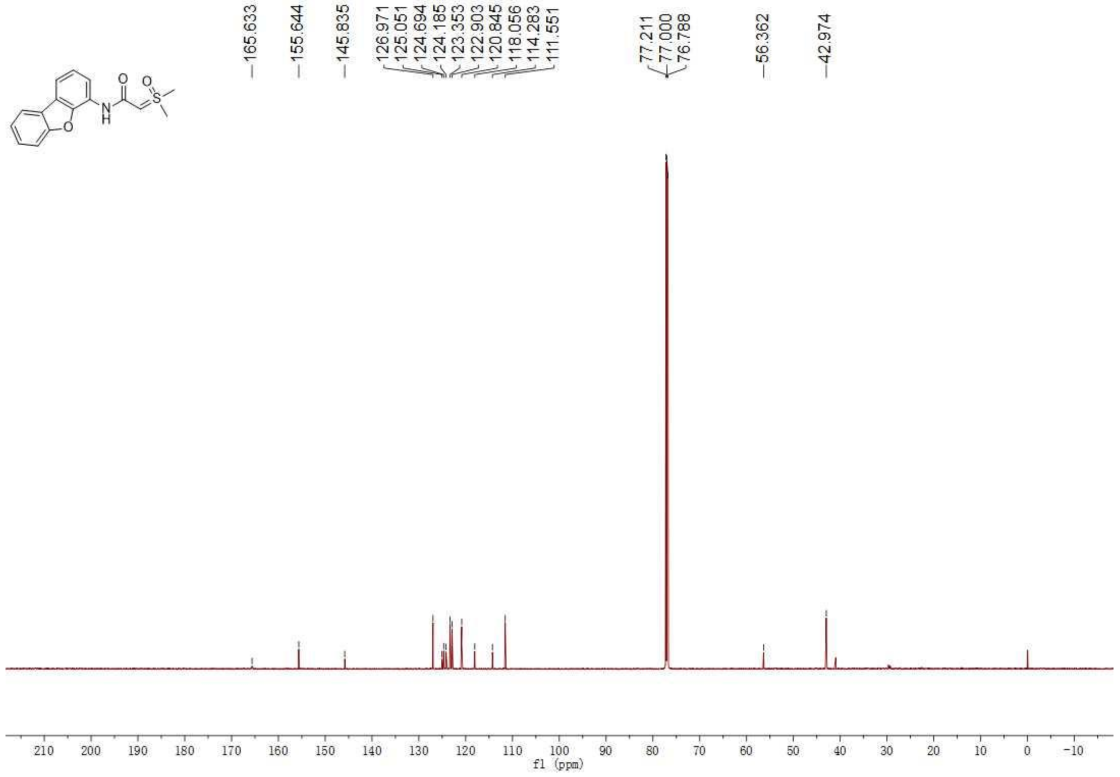

^{1}H NMR (600 MHz, $CDCl_3$) Spectrum of **S43**

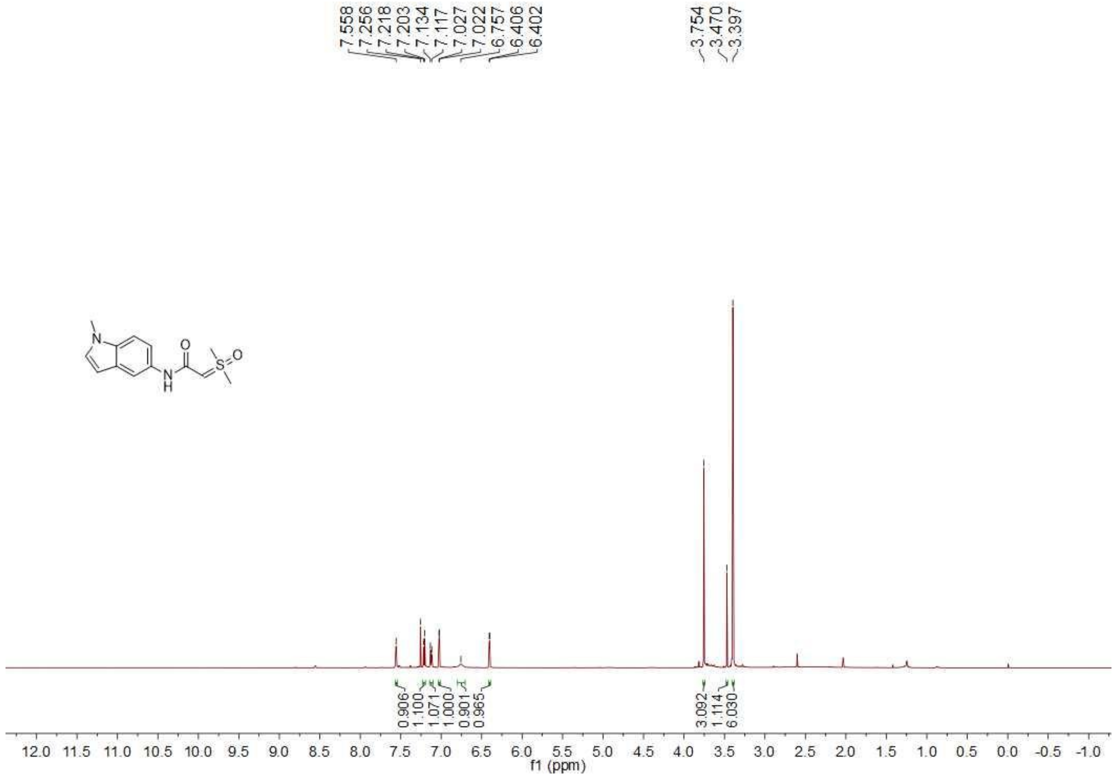

^{13}C NMR (150 MHz, $CDCl_3$) Spectrum of **S43**

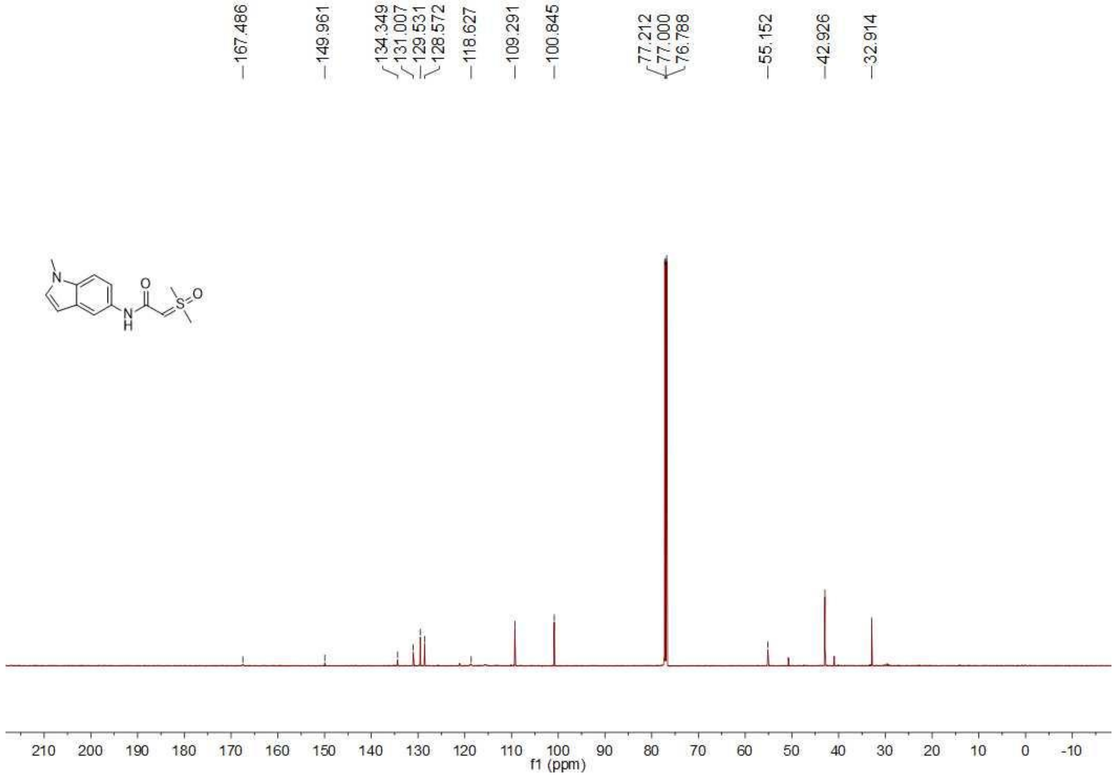

^{1}H NMR (600 MHz, $CDCl_3$) Spectrum of **S44**

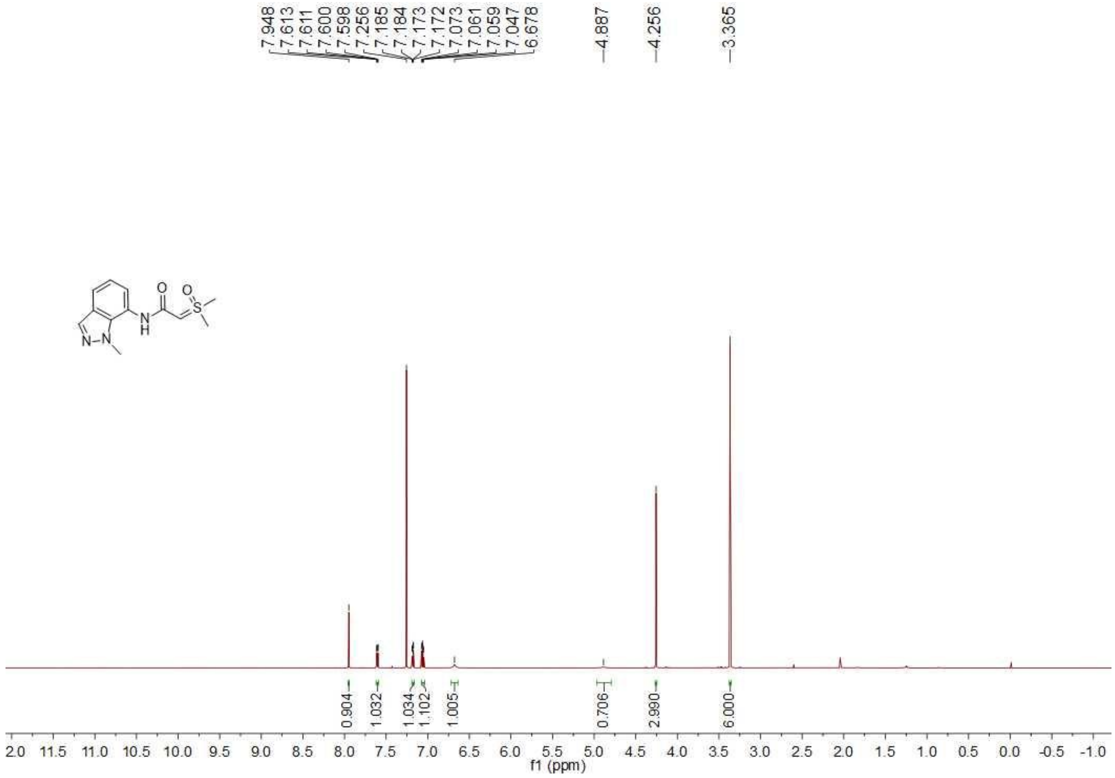

^{13}C NMR (150 MHz, $CDCl_3$) Spectrum of **S44**

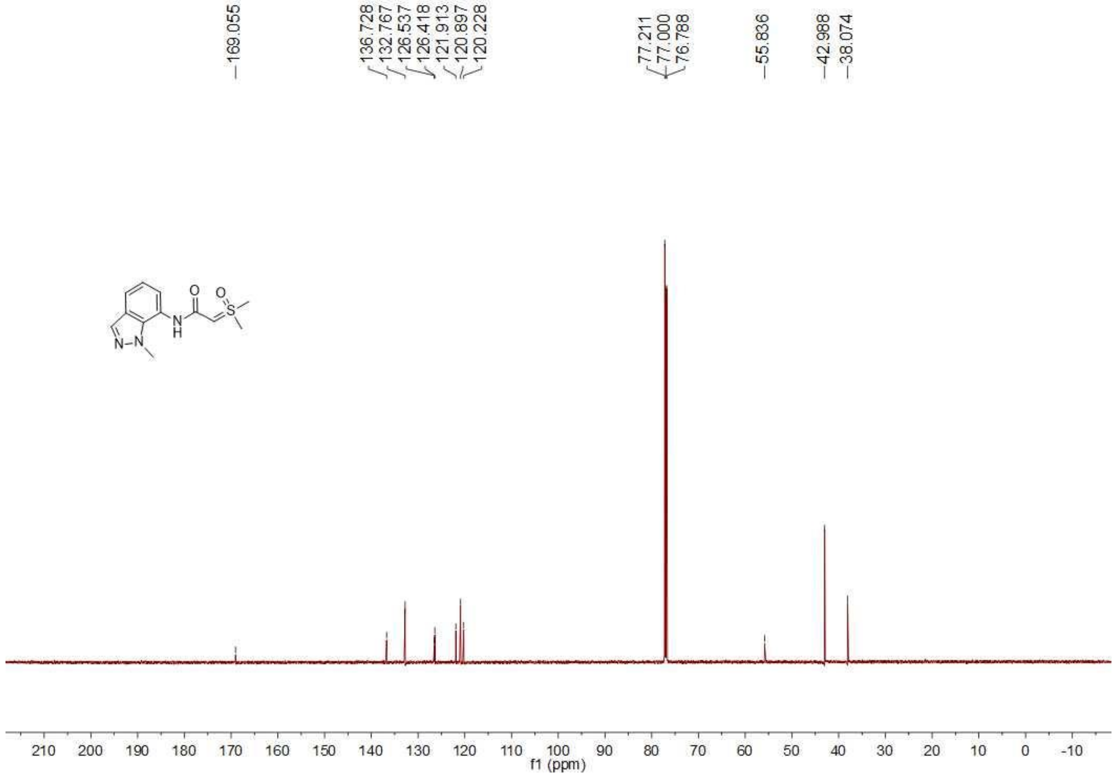

^{1}H NMR (600 MHz, DMSO-d_6) Spectrum of **S45**

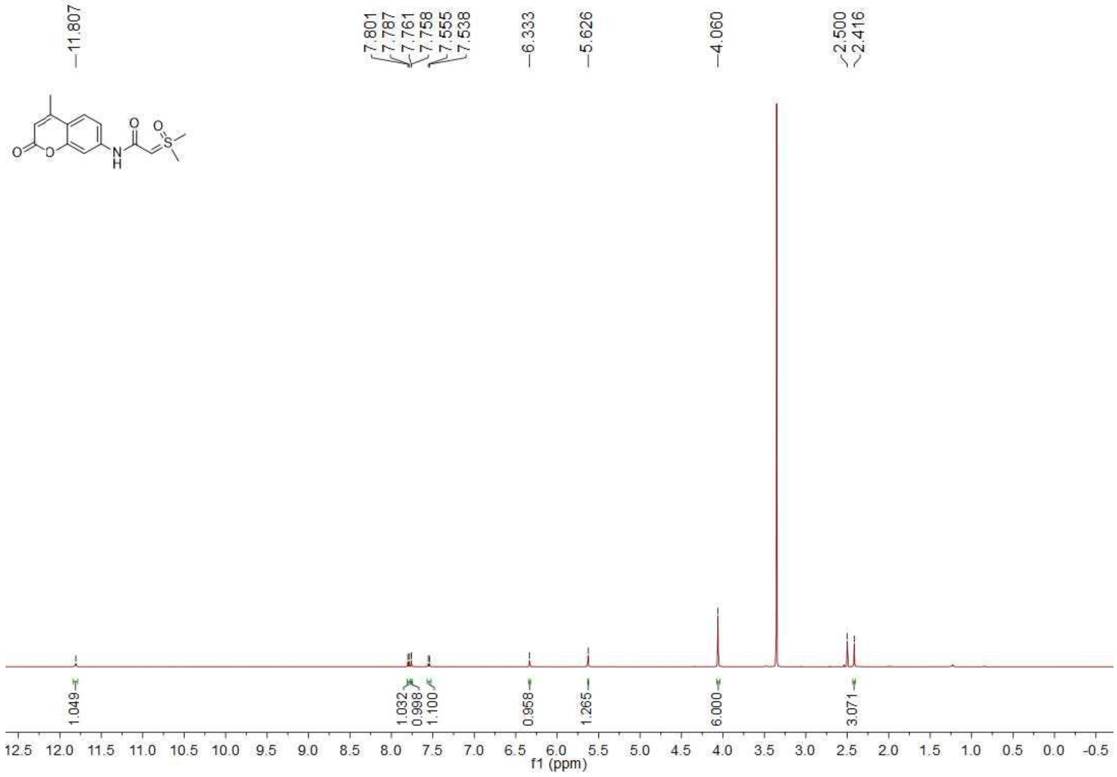

^{13}C NMR (150 MHz, DMSO-d_6) Spectrum of **S45**

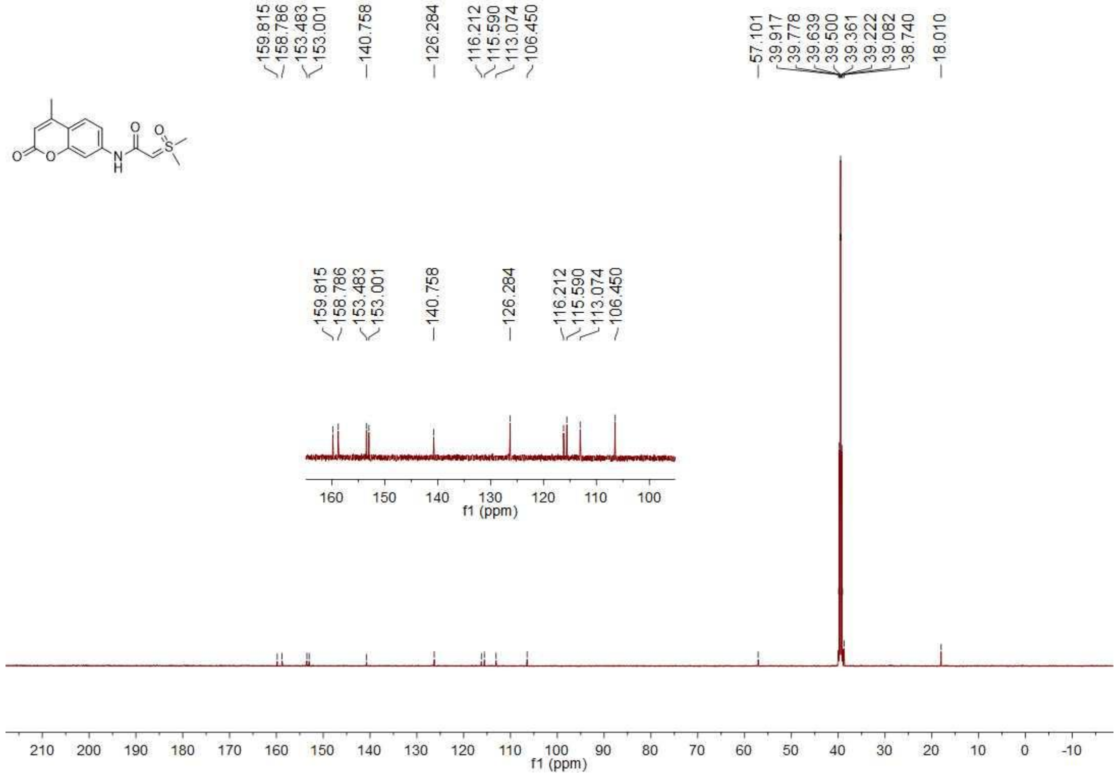

^{1}H NMR (600 MHz, DMSO-d_6) Spectrum of **S46**

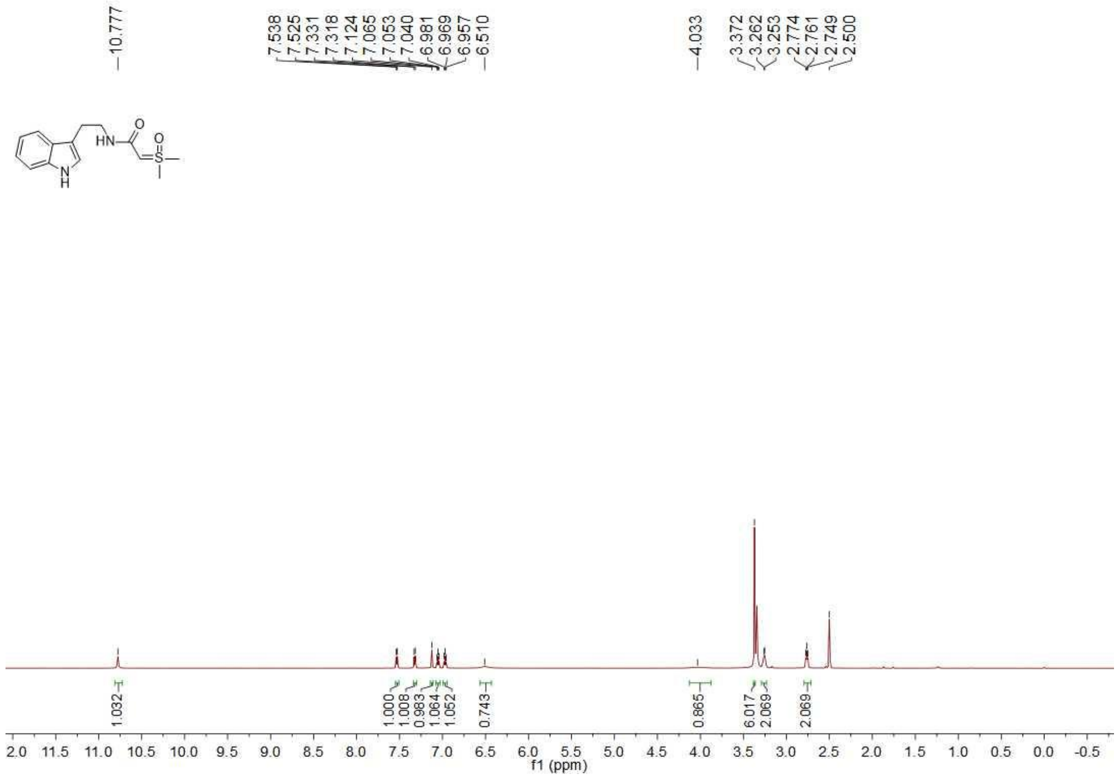

^{13}C NMR (150 MHz, DMSO-d_6) Spectrum of **S46**

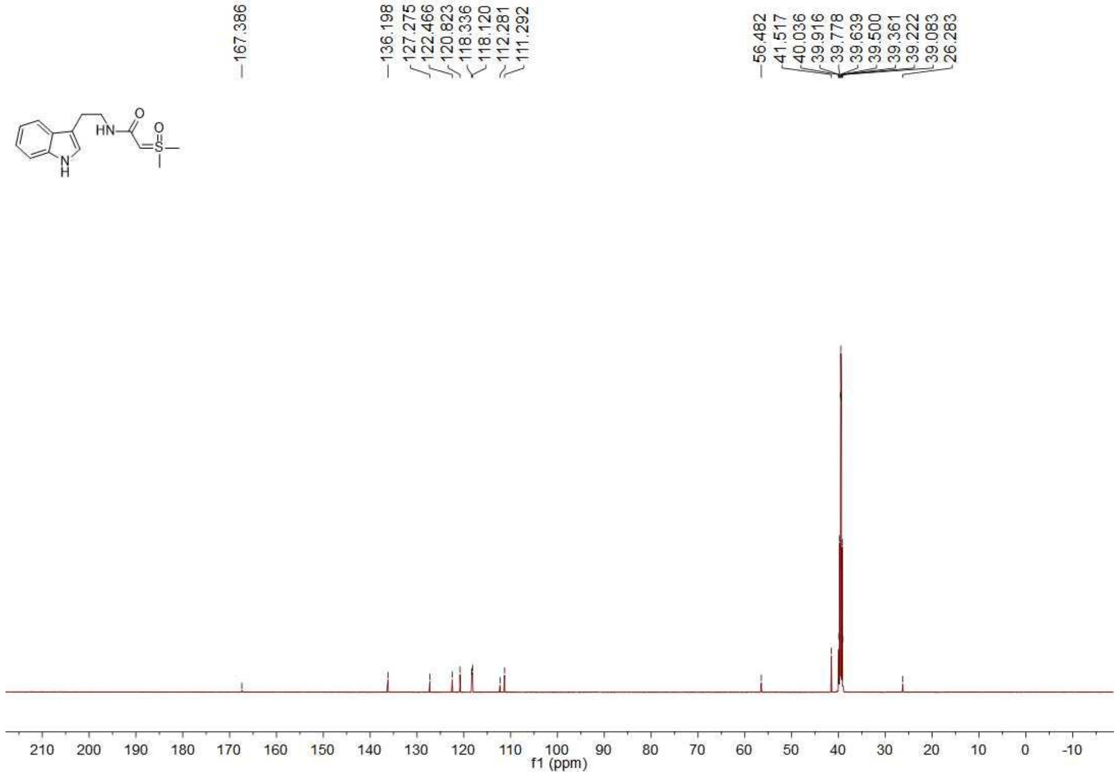

^{1}H NMR (600 MHz, DMSO-d_6) Spectrum of **S47**

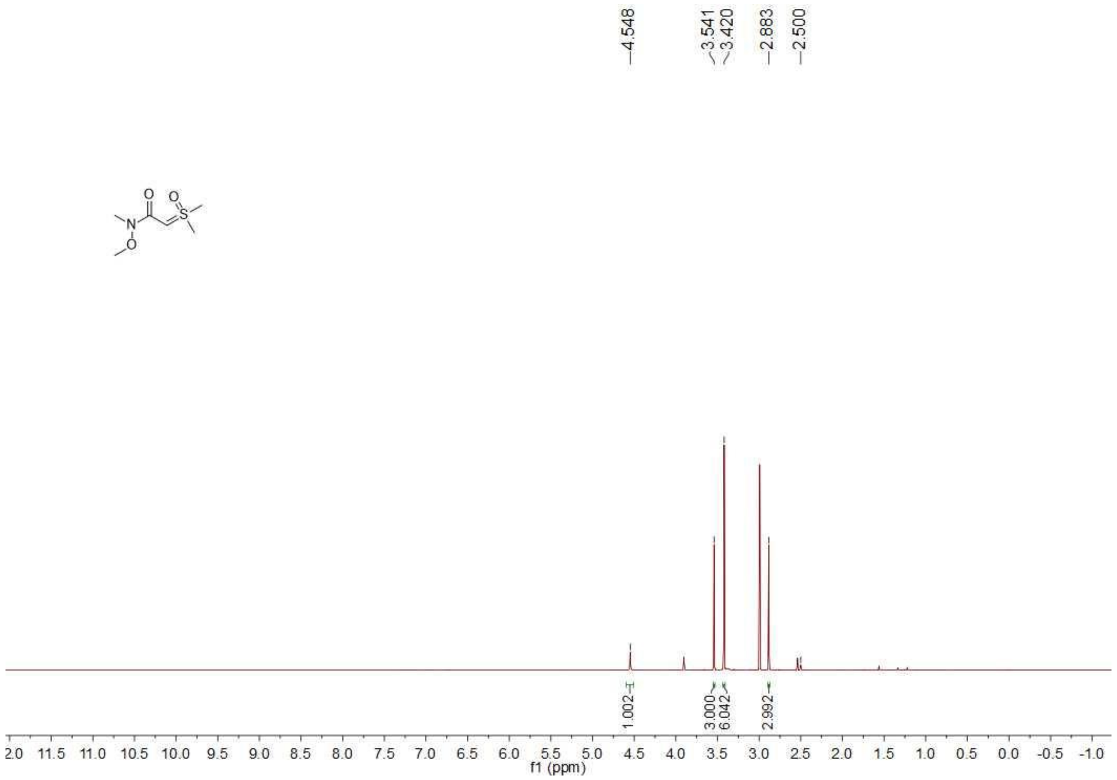

^{13}C NMR (150 MHz, DMSO-d_6) Spectrum of **S47**

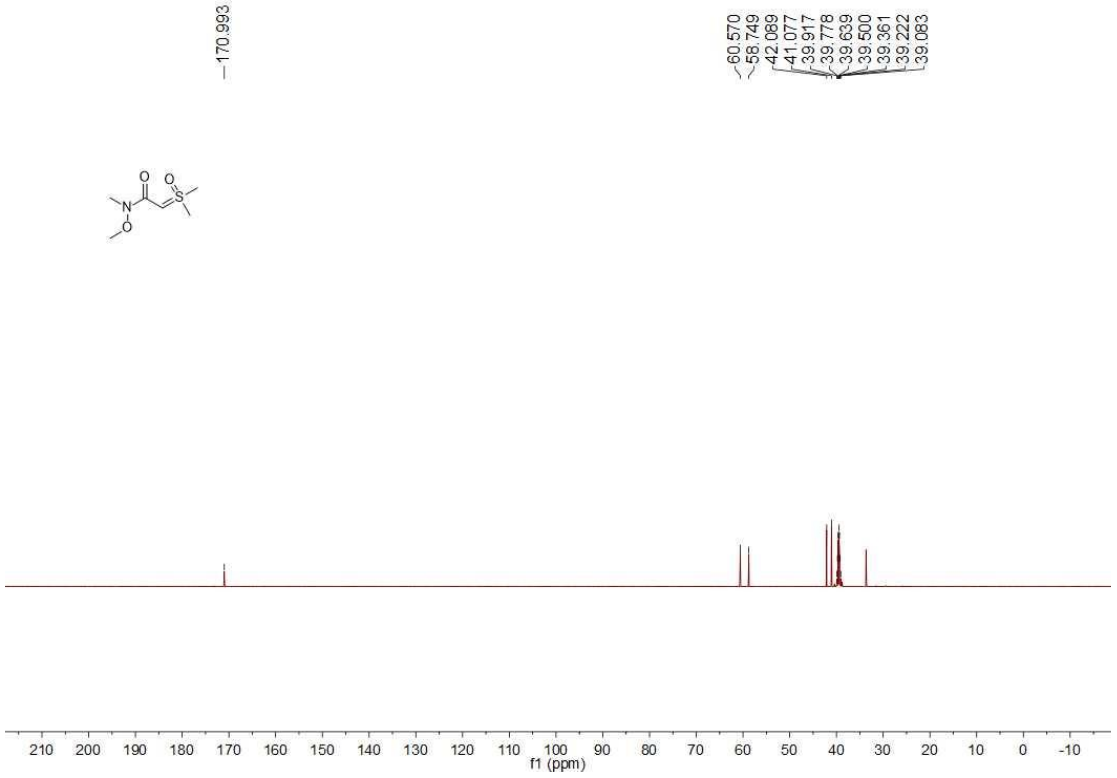

^{1}H NMR (600 MHz, DMSO-d_6) Spectrum of **S48**

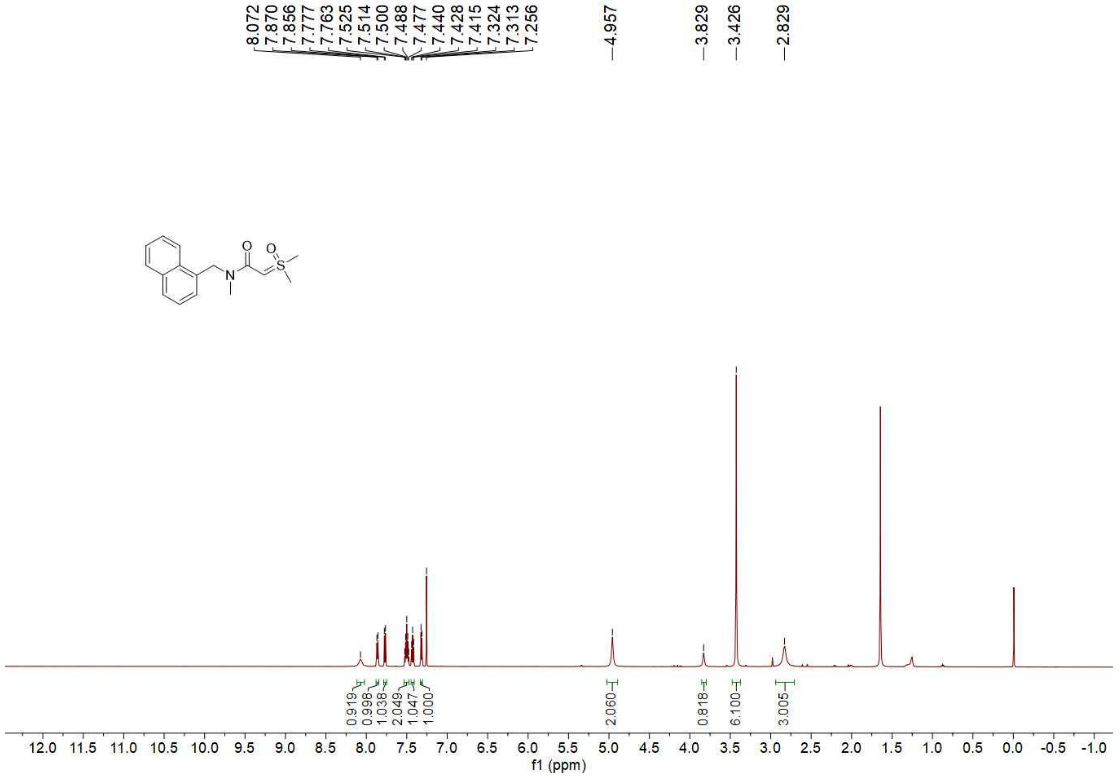

^{13}C NMR (150 MHz, DMSO-d_6) Spectrum of **S48**

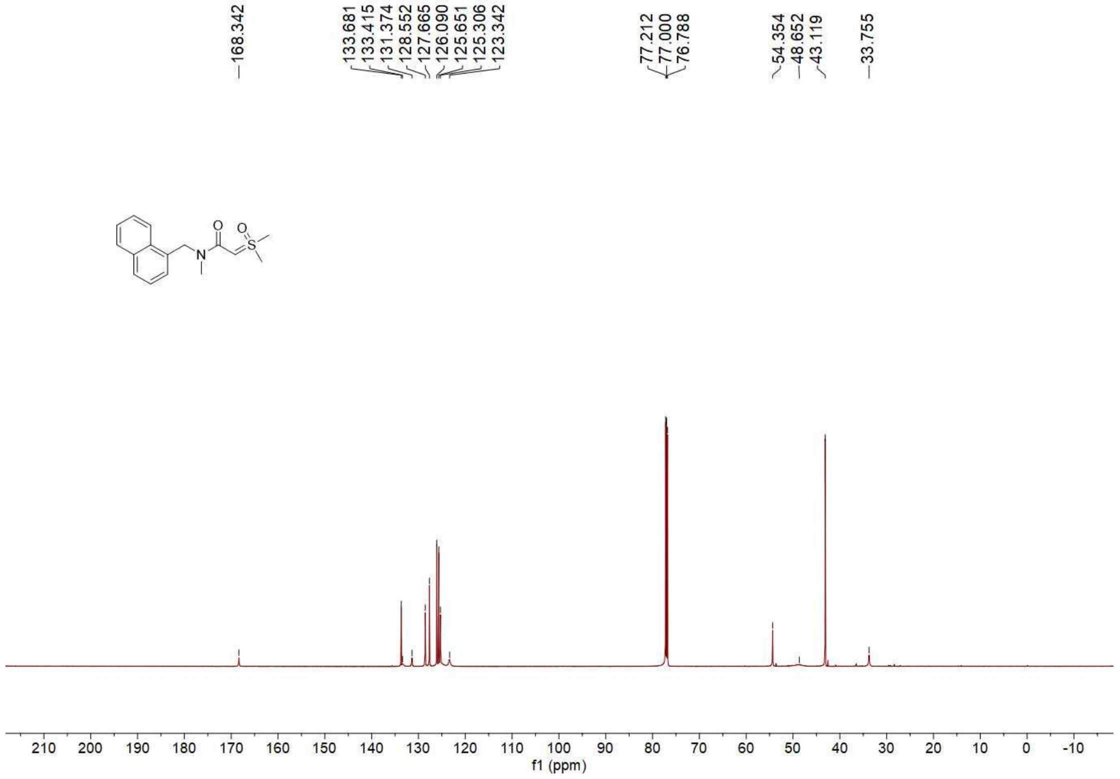

^{1}H NMR (600 MHz, DMSO-d_6) Spectrum of **S49**

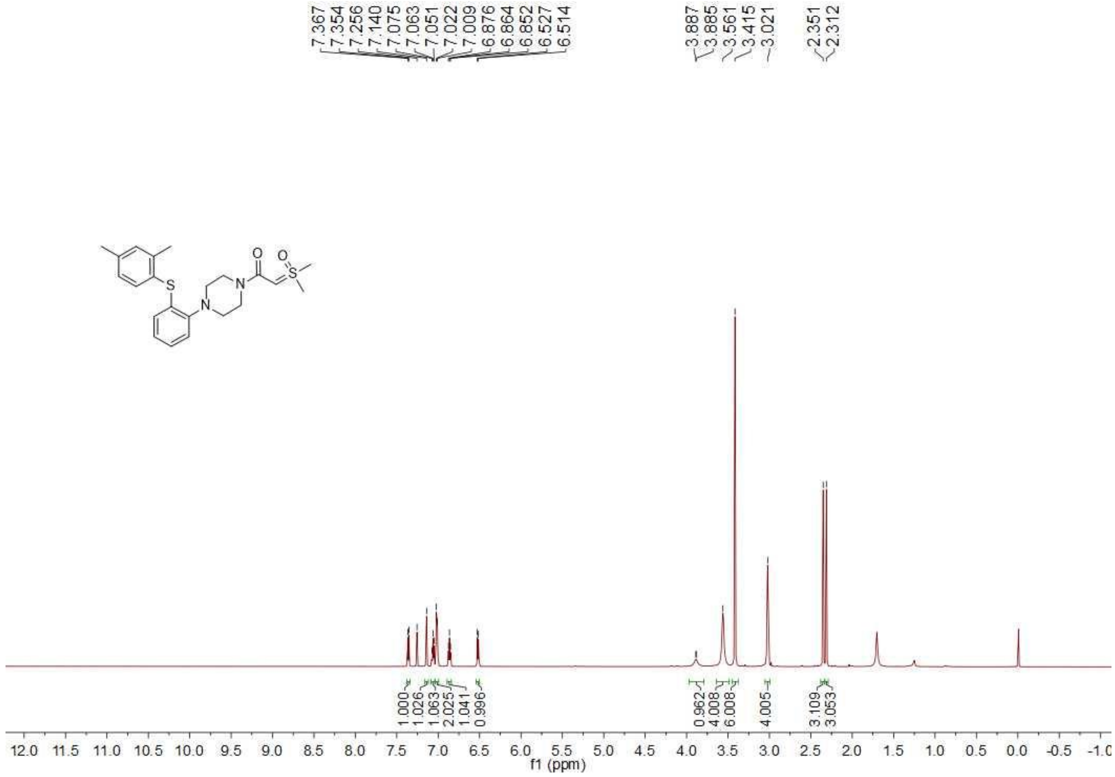

^{13}C NMR (150 MHz, DMSO-d_6) Spectrum of **S49**

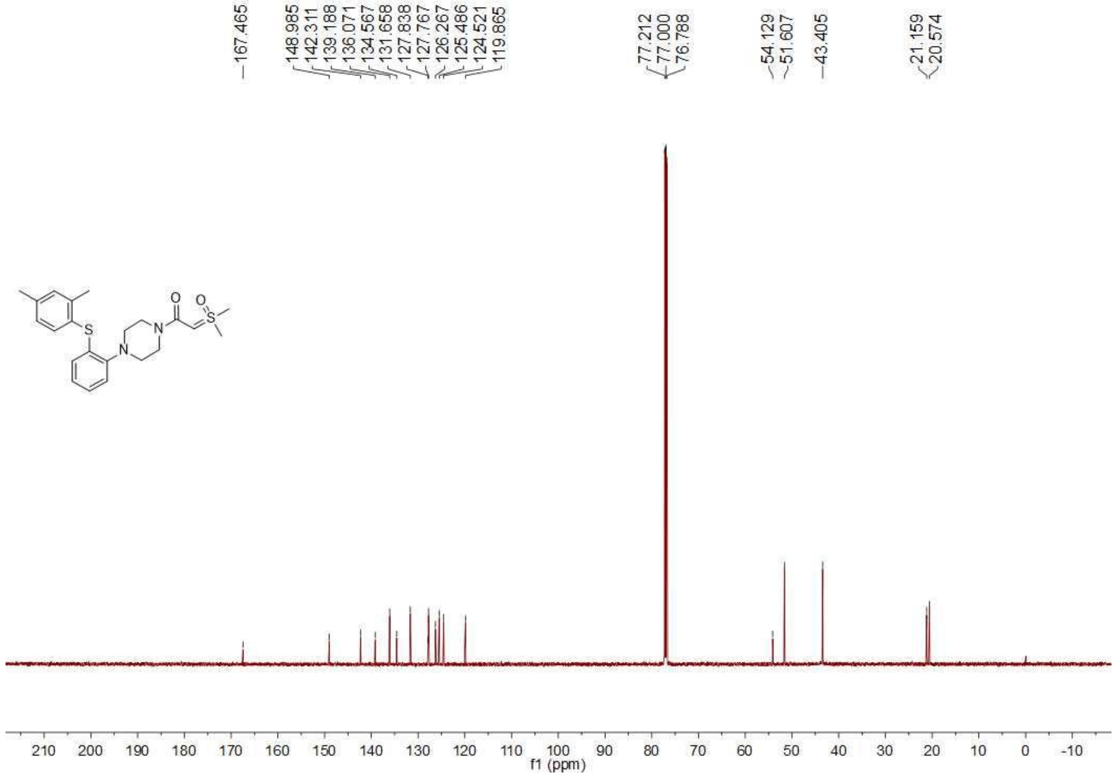

^{1}H NMR (600 MHz, $CDCl_3$) Spectrum of **S50**

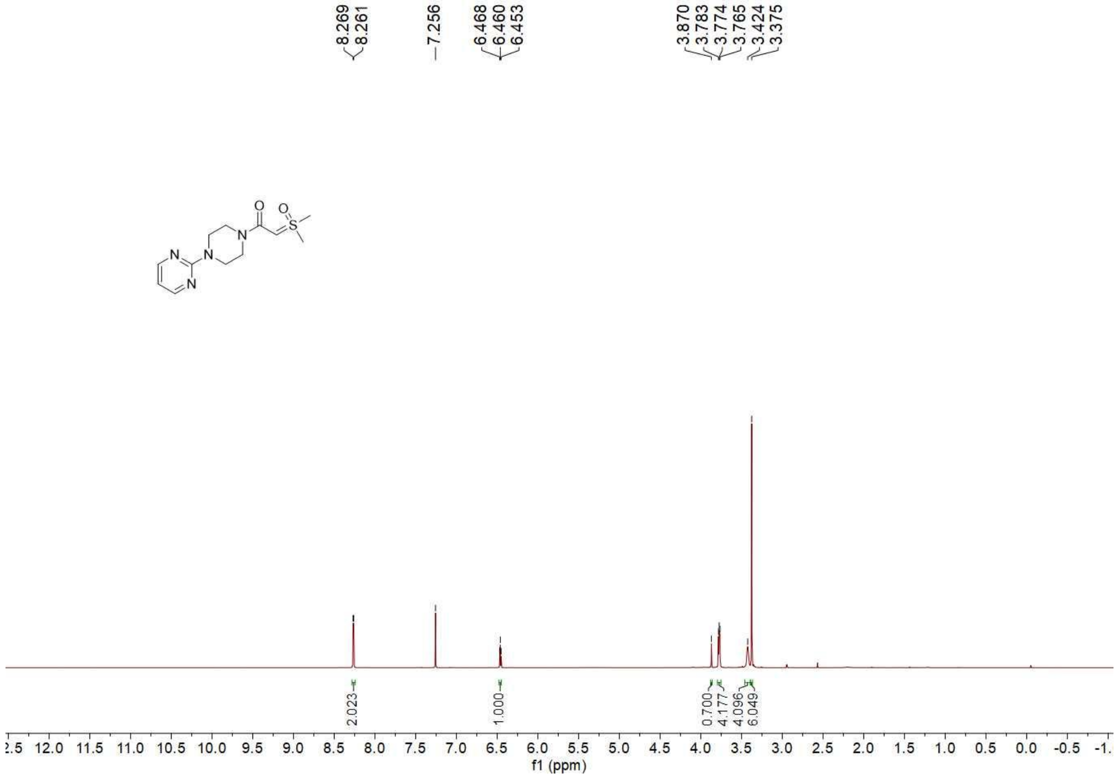

^{13}C NMR (150 MHz, $CDCl_3$) Spectrum of **S50**

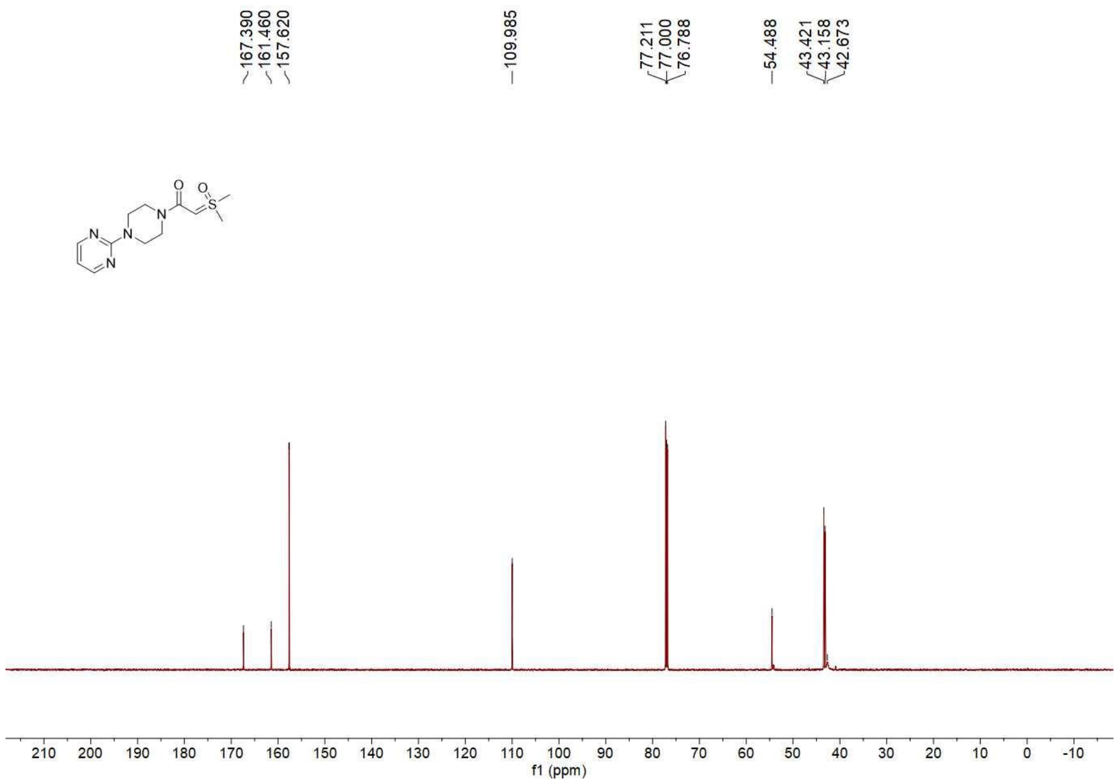

^{1}H NMR (600 MHz, $CDCl_3$) Spectrum of **S51**

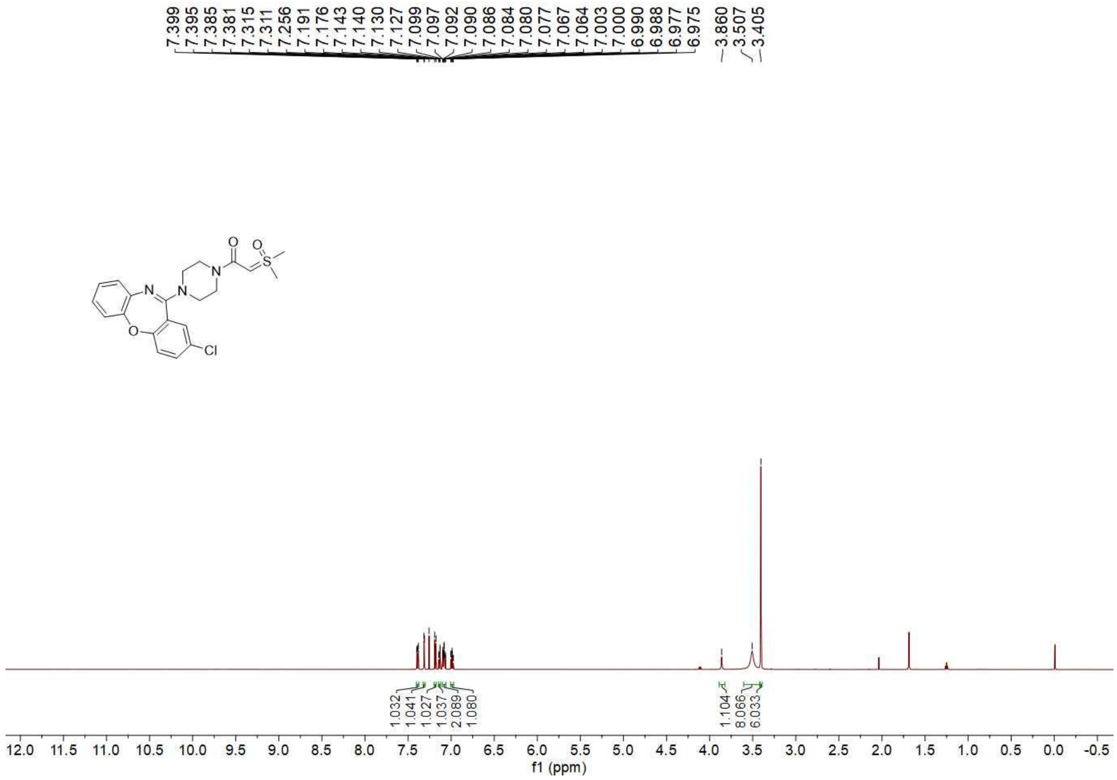

^{13}C NMR (150 MHz, $CDCl_3$) Spectrum of **S51**

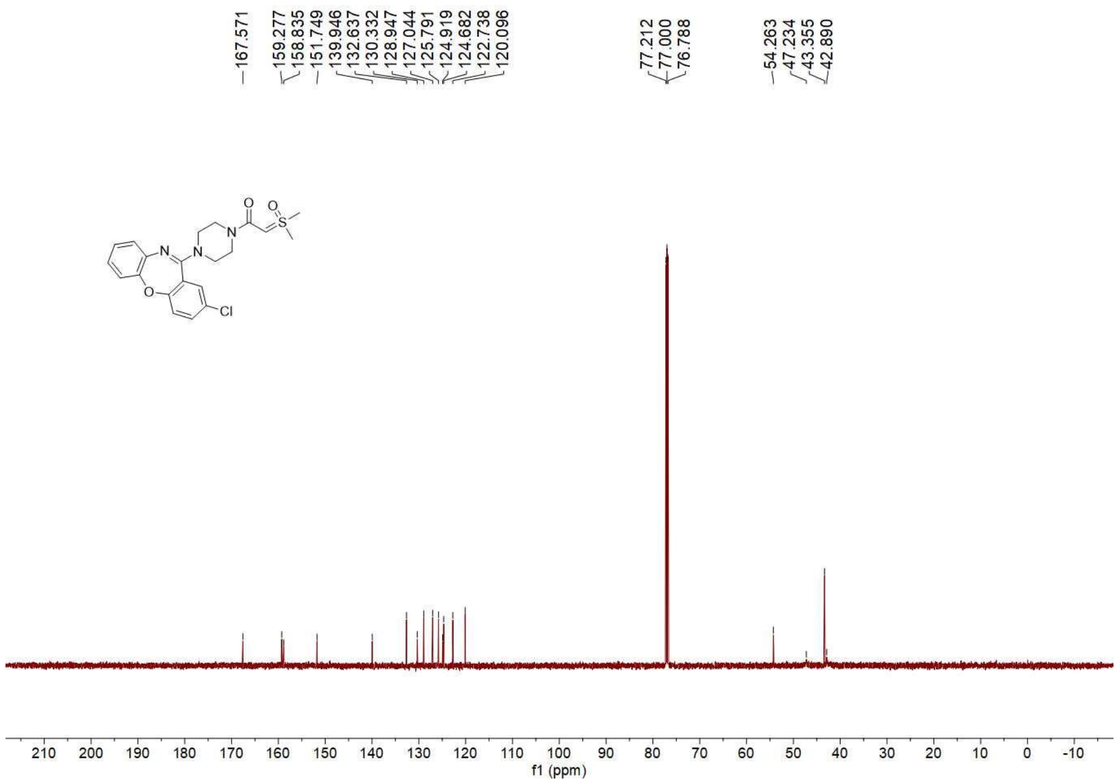

^{1}H NMR (600 MHz, DMSO-d_6) Spectrum of **S56**

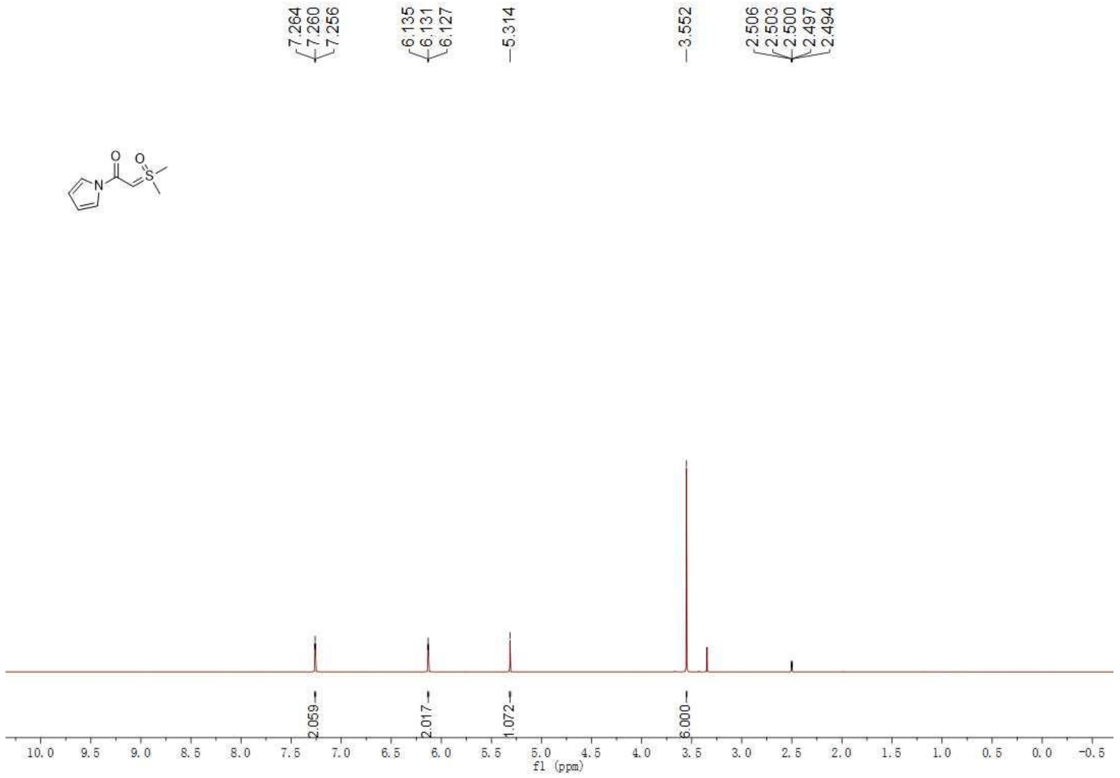

^{13}C NMR (150 MHz, DMSO-d_6) Spectrum of **S56**

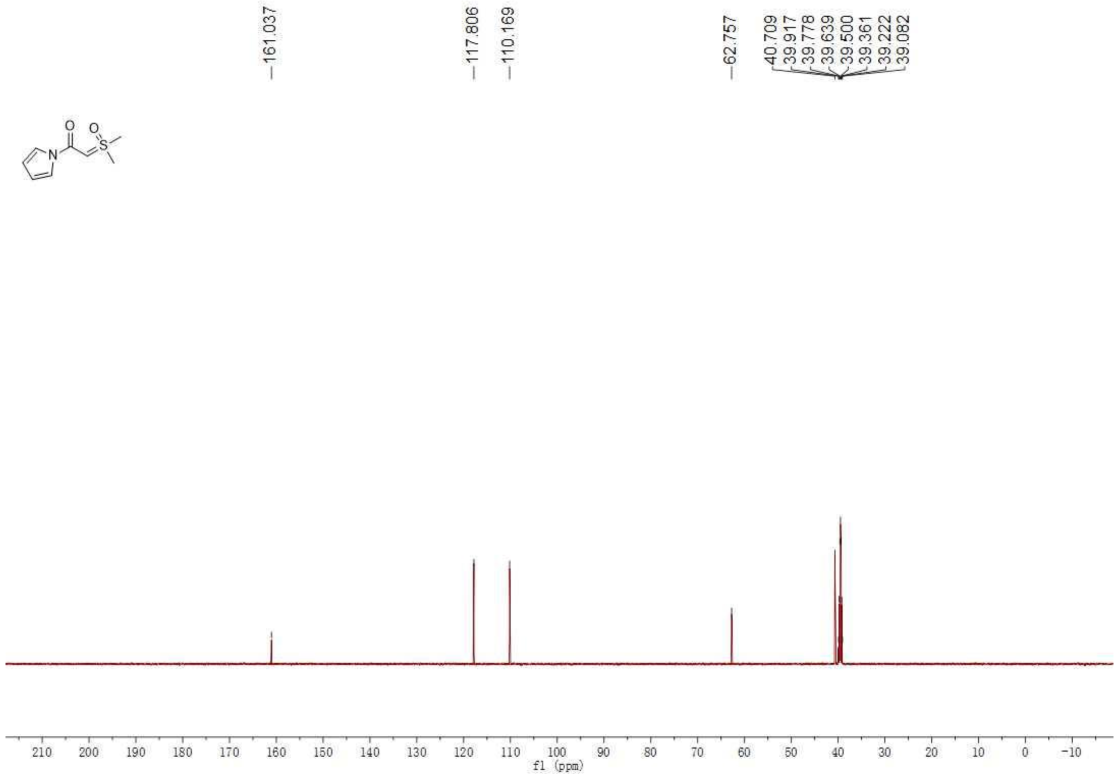

^{1}H NMR (600 MHz, $CDCl_3$) Spectrum of **S57**

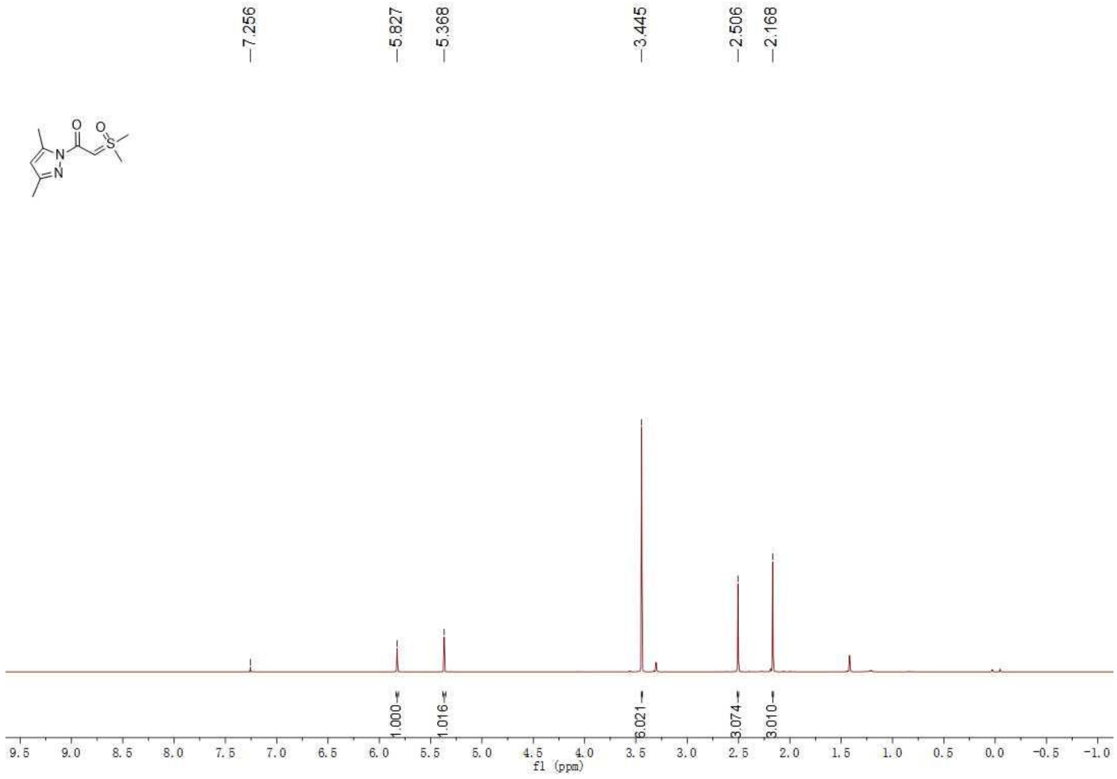

^{13}C NMR (150 MHz, $CDCl_3$) Spectrum of **S57**

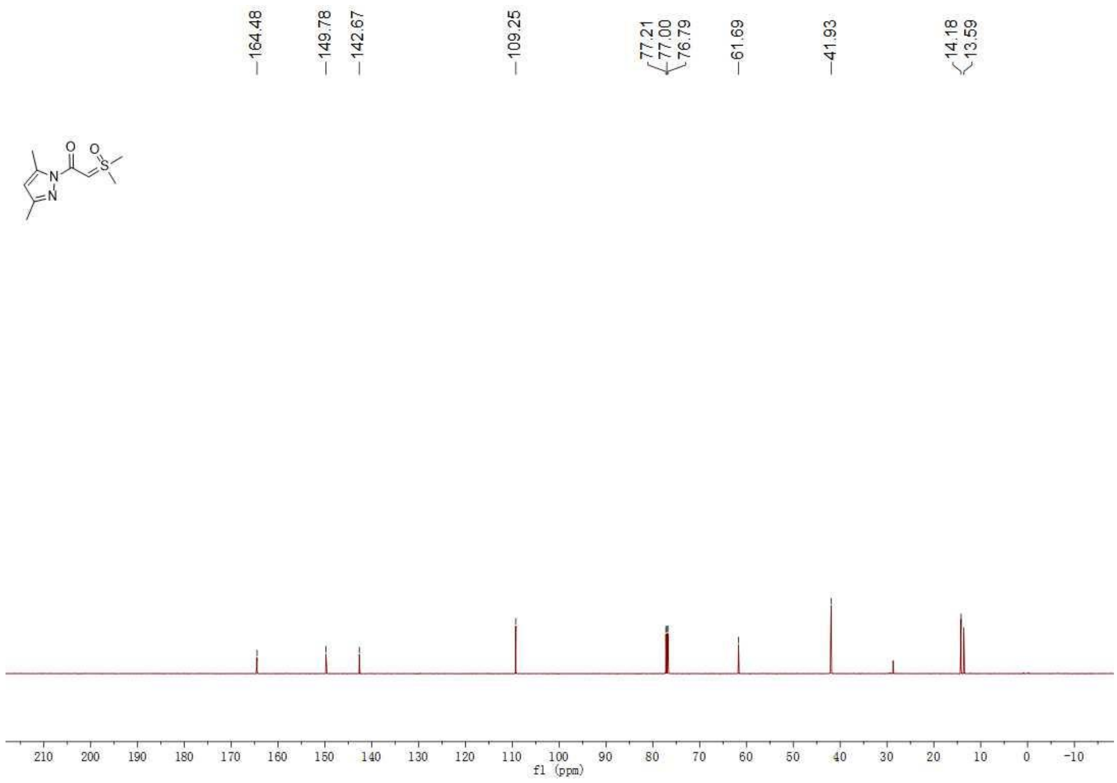

^{1}H NMR (600 MHz, DMSO-d_6) Spectrum of **S59**

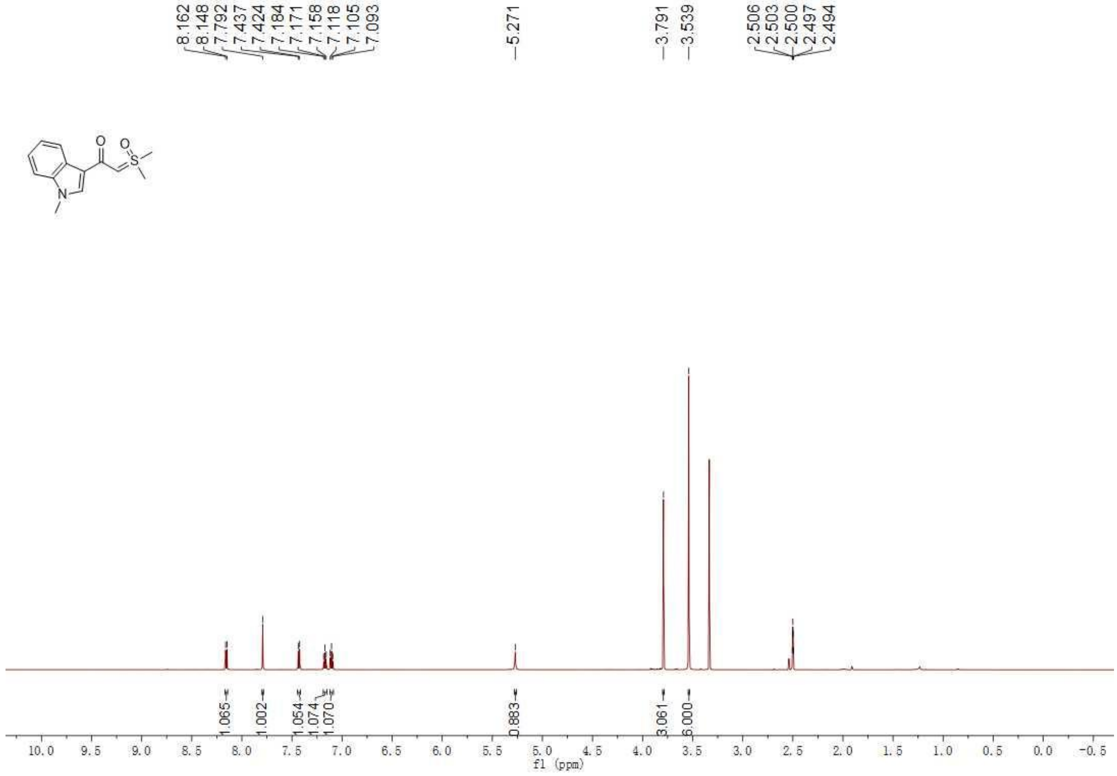

^{13}C NMR (150 MHz, DMSO-d_6) Spectrum of **S59**

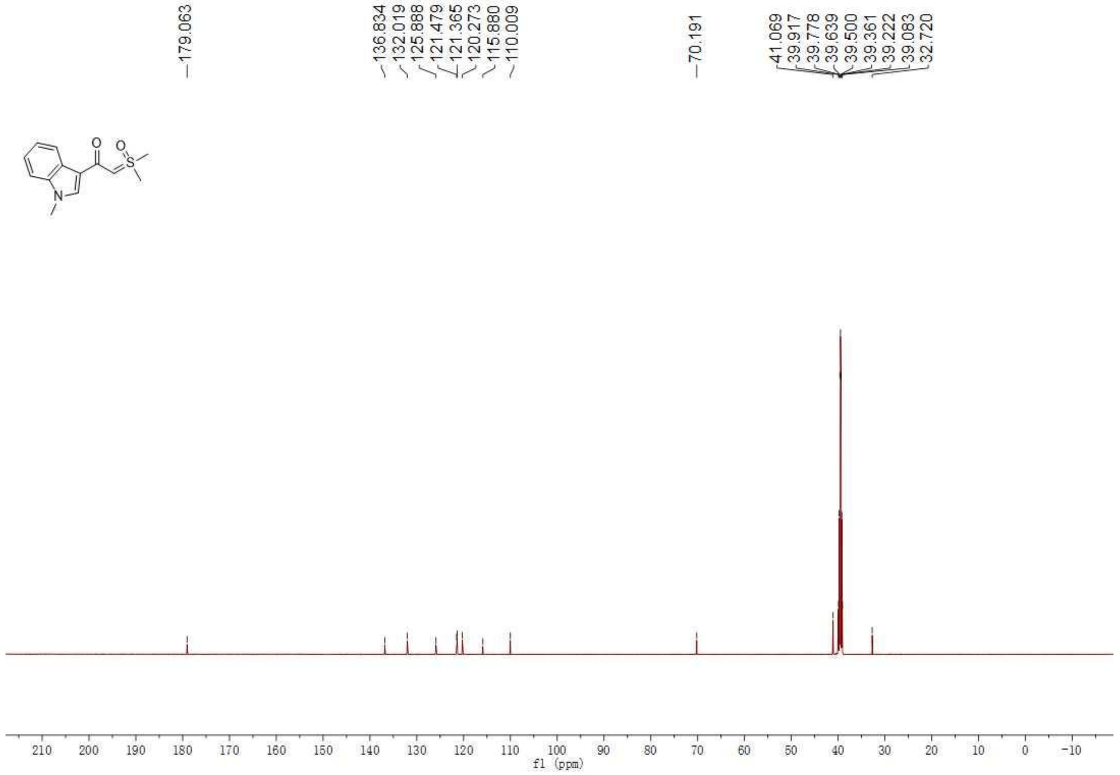

^{1}H NMR (600 MHz, $CDCl_3$) Spectrum of **1a-*d*$_7$**

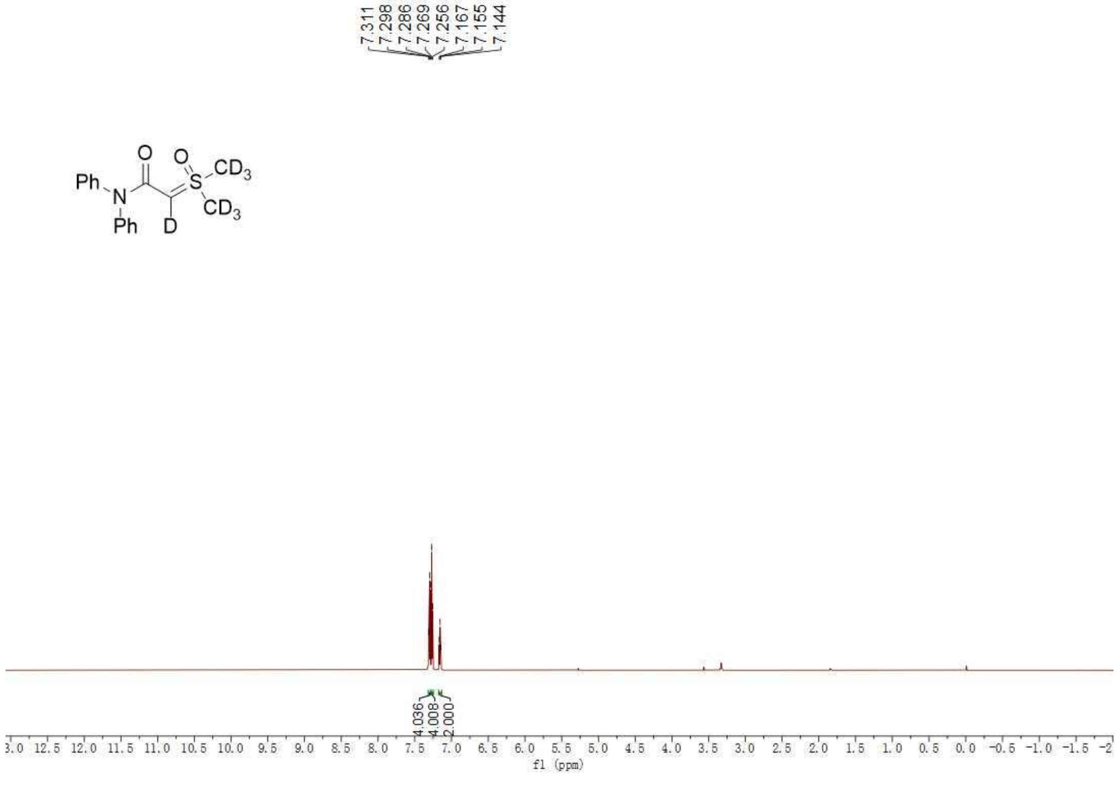

^{13}C NMR (150 MHz, $CDCl_3$) Spectrum of **1a-*d*$_7$**

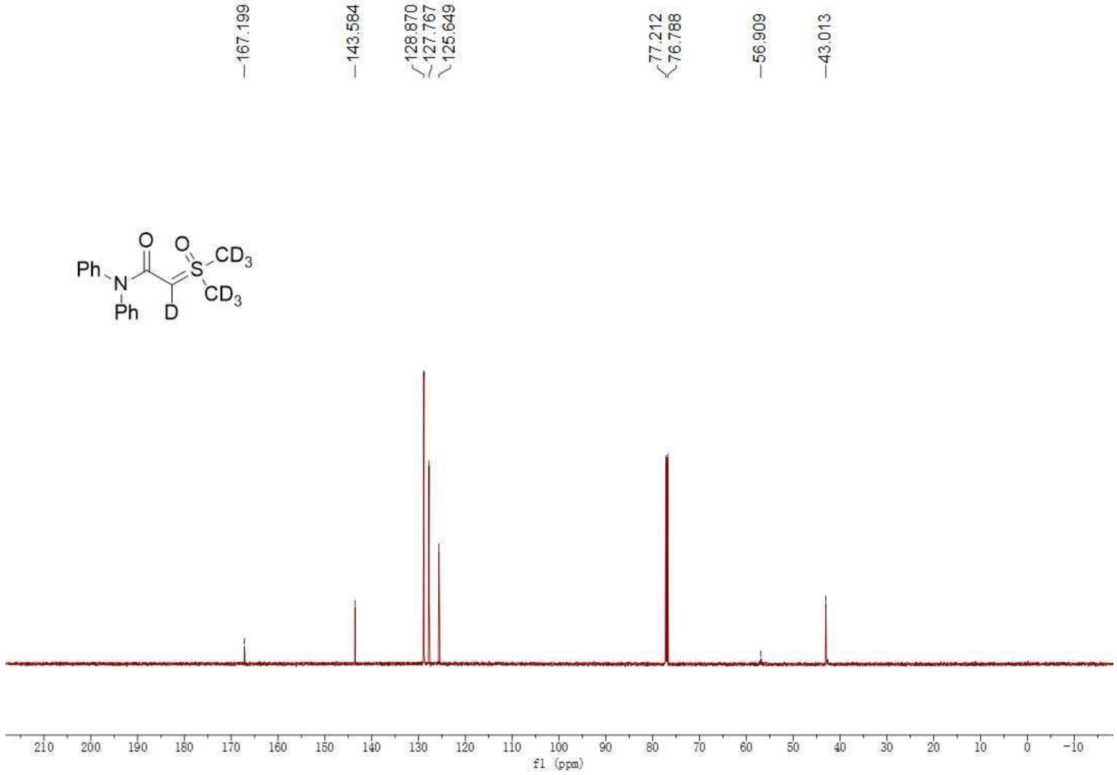

Supplement: Supplementary file 1 — Supporting Information [file ADVS-12-2417362-s001.pdf]
